# Genome-Based Taxonomic Classification of the Phylum *Actinobacteria*

**DOI:** 10.3389/fmicb.2018.02007

**Published:** 2018-08-22

**Authors:** Imen Nouioui, Lorena Carro, Marina García-López, Jan P. Meier-Kolthoff, Tanja Woyke, Nikos C. Kyrpides, Rüdiger Pukall, Hans-Peter Klenk, Michael Goodfellow, Markus Göker

**Affiliations:** ^1^School of Natural and Environmental Sciences, Newcastle University, Newcastle upon Tyne, United Kingdom; ^2^Department of Microorganisms, Leibniz Institute DSMZ – German Collection of Microorganisms and Cell Cultures, Braunschweig, Germany; ^3^Department of Energy, Joint Genome Institute, Walnut Creek, CA, United States

**Keywords:** G+C content, genome size, Genome BLAST Distance Phylogeny, chemotaxonomy, morphology, phylogenetic systematics, phylogenomics

## Abstract

The application of phylogenetic taxonomic procedures led to improvements in the classification of bacteria assigned to the phylum *Actinobacteria* but even so there remains a need to further clarify relationships within a taxon that encompasses organisms of agricultural, biotechnological, clinical, and ecological importance. Classification of the morphologically diverse bacteria belonging to this large phylum based on a limited number of features has proved to be difficult, not least when taxonomic decisions rested heavily on interpretation of poorly resolved 16S rRNA gene trees. Here, draft genome sequences of a large collection of actinobacterial type strains were used to infer phylogenetic trees from genome-scale data using principles drawn from phylogenetic systematics. The majority of taxa were found to be monophyletic but several orders, families, and genera, as well as many species and a few subspecies were shown to be in need of revision leading to proposals for the recognition of 2 orders, 10 families, and 17 genera, as well as the transfer of over 100 species to other genera. In addition, emended descriptions are given for many species mainly involving the addition of data on genome size and DNA G+C content, the former can be considered to be a valuable taxonomic marker in actinobacterial systematics. Many of the incongruities detected when the results of the present study were compared with existing classifications had been recognized from 16S rRNA gene trees though whole-genome phylogenies proved to be much better resolved. The few significant incongruities found between 16S/23S rRNA and whole genome trees underline the pitfalls inherent in phylogenies based upon single gene sequences. Similarly good congruence was found between the discontinuous distribution of phenotypic properties and taxa delineated in the phylogenetic trees though diverse non-monophyletic taxa appeared to be based on the use of plesiomorphic character states as diagnostic features.

## Introduction

Prokaryotic systematics is the study of the kinds and diversity of *Archaea* and *Bacteria* and relationships within and between them. It encompasses the related disciplines of classification, nomenclature and identification, the concept of the taxonomic trinity (Cowan, 1955). Classification and identification are core scientific disciplines that are practiced by few but their applications are relevant to most, if not all, of the microbiological community. Nomenclature is also central to all aspects of microbiology as it deals with the correct use of names by following rules embodied in the *International Code of Nomenclature of Prokaryotes*, notably the nomenclatural type concept and the requirement to deposit type strains in two public culture collections in different countries (Stackebrandt et al., 2014; Parker et al., 2015). Classification and identification are markedly data-dependent and thereby in a perpetual state of development driven by new taxonomic concepts and practices.

Early classification of prokaryotes, including actinomycetes, which are now known as actinobacteria, was based on morphology and a few biochemical and physiological properties, as exemplified by *Nocardia*, the oldest name in current use for an aerobic actinomycete genus (Lechevalier, 1976). However, the subsequent application of chemotaxonomic, molecular systematic and numerical taxonomic procedures (Goodfellow et al., 1985) showed that classifications dependent on form and function tended to result in the delineation of heterogeneous taxa. *Nocardia*, for instance, as morphologically defined in the eighth edition of *Bergey's Manual of Determinative Bacteriology* (McClung, 1974) contained species that were later assigned taxonomically to diverse genera, including *Actinomadura, Amycolatopsis, Oerskovia, Rhodococcus*, and *Rothia* (Goodfellow and Minnikin, 1977; Lechevalier et al., 1986). Similarly, a series of more broadly based studies showed that coryneform bacteria, at the time an ill-assorted group in search of a taxonomic home, were actinomycetes (Bousfield and Callely, 1978). Conversely, *Thermoactinomyces*, which had, long been classified with the actinomycetes, was found to be related to aerobic, endospore-forming bacilli (Cross and Unsworth, 1981).

The new advances, especially in molecular systematics, led to the view that classifications at all levels in the taxonomic hierarchy should be based on the integrated use of genotypic and phenotypic data (Wayne et al., 1987; Stackebrandt, 1992). This approach, known as polyphasic taxonomy (Colwell, 1970), has become the lodestone of prokaryotic systematics (Vandamme et al., 1996; Gillis et al., 2005; Kämpfer and Glaeser, 2012). Methods selected for polyphasic studies tend to be somewhat subjective as they reflect the biology of taxa under study and available facilities. However, a prevailing theme in such studies is the comparative analysis of 16S rRNA gene sequences designed (Kim and Chun, 2014; Yarza and Munoz, 2014) to infer relationships between strains at generic and suprageneric ranks (Ludwig et al., 2012).

The widespread application of polyphasic taxonomy led to sweeping changes in the classification of prokaryotes (Brenner et al., 2005b,c; De Vos et al., 2009), including actinomycetes, as exemplified by the current classification of the phylum *Actinobacteria*, as shown in *Bergey's Manual of Systematic Bacteriology* (Goodfellow et al., 2012a). Despite the success of 16S rRNA gene sequencing in providing the phylogenetic backbone for the classification of prokaryotes (Brenner et al., 2005a; Ludwig and Klenk, 2005) problems remain in circumscribing actinobacterial taxa, including at family and genus levels. *Nocardiaceae* (Castellani and Chalmers, 1919), for instance, encompass the type genus *Nocardia* as well as *Gordonia, Millisia, Rhodococcus, Skermania*, and *Smaragdicoccus* (Goodfellow, 2012a) though a case was also made for the continued recognition of *Gordoniaceae* (Stackebrandt et al., 1997) to include not only the type genus, *Gordonia*, but also *Millisia, Skermania*, and *Williamsia* (Goodfellow, 2012a). Other families considered to be problematic include *Cellulomonadaceae, Dermacoccaceae, Dermatophilaceae, Intrasporangiaceae*, and *Nocardioidaceae* (Goodfellow, 2012a). The current assignment of genera to such families should be seen as staging posts to better classifications.

The situation at the genus level is not dissimilar to that outlined above as 16S rRNA gene trees often also lack the resolution to distinguish between closely related genera, as shown by *Lechevalieria* (Labeda et al., 2001) and *Lentzea* (Yassin et al., 1995), which are difficult to distinguish when assigning new species to either genus (Okoro et al., 2010; Idris et al., 2017). In turn, the observation that *Kitasatospora* (Omura et al., 1982) and *Streptacidiphilus* (Kim et al., 2003) fall within the *Streptomyces* 16S rRNA gene tree led some to question their taxonomic status (Kämpfer, 2012a). Along the same lines, the monospecific genera *Jiangella* (Song et al., 2005), *Plantactinospora* (Qin et al., 2009a), and *Polymorphospora* (Tamura et al., 2006) were interspersed within the *Micromonospora* 16S rRNA gene tree together with authentic representatives of *Salinispora* (Maldonado et al., 2005) and *Verrucosispora* (Rheims et al., 1998) in a recent study (Trujillo et al., 2014). It is also difficult to unravel the internal structure of complex actinobacterial genera using data acquired from polyphasic taxonomic studies, notable examples include *Actinomyces* (Schaal and Yassin, 2012a), *Amycolatopsis* (Tan and Goodfellow, 2012), *Arthrobacter* (Busse et al., 2012), *Micromonospora* (Carro et al., 2018), *Nocardioides* (Evtushenko et al., 2012), *Rhodococcus* (Jones and Goodfellow, 2012), and *Streptomyces* (Labeda et al., 2012). Polyphasic studies as currently conducted largely depend on the 16S rRNA gene (Montero-Calasanz et al., 2017) but despite its usefulness for resolving taxonomic questions in the past, the gene contains only a limited number of characters and thus, much like any other single gene, can yield trees with many statistically unsupported branches (Klenk and Göker, 2010; Breider et al., 2014).

There is clearly a compelling need to develop an improved framework for the classification of actinobacteria, a goal in its own right (Klenk and Göker, 2010) but also one driven by biotechnological, bioprospecting, and ecological imperatives (Bull et al., 2016; Katz and Baltz, 2016; Benito et al., 2017) and by fundamental research interests (Chandra and Chater, 2014; Barka et al., 2016). Given the rapid and ongoing progress in sequencing technologies (Mavromatis et al., 2012), classifications based on whole genome sequences and associated bioinformatic tools are addressing these needs as they are based on millions of unit characters and thereby provide a step change in reliability, as evidenced by significantly high average bootstrap support in phylogenomic trees (Breider et al., 2014; Meier-Kolthoff et al., 2014a). In contrast, trees based on a few thousand nucleotides (Tang et al., 2016) tend to have branches with low bootstrap values; the same limitation applies, albeit to a lesser extent, to multi-locus sequence analyses of conserved housekeeping genes (Glaeser and Kämpfer, 2015), which can hardly be called genome-scale (Klenk and Göker, 2010). Phylogenomic methods have already been applied to elucidate the classification of complex actinobacterial taxa, such as the genera *Amycolatopsis, Micromonospora, Rhodococcus*, and *Salinispora* (Sangal et al., 2016; Tang et al., 2016; Jensen, 2017; Carro et al., 2018), and in some cases has led to marked reclassification (Montero-Calasanz et al., 2017). However, a comprehensive analysis of the phylum *Actinobacteria* based on truly genome-scale methods has not been undertaken.

Experimental methods that indirectly determine DNA G+C composition (Mesbah et al., 1989; Moreira et al., 2011) can now be replaced by calculating it directly from accurate genome sequences. Claims in the literature that the variation in G+C content within bacterial species is at most 3 mol% (Mesbah et al., 1989) or even 5% (Rosselló-Mora and Amann, 2001) can be attributed to experimental error as within-species variation is at most 1% when G+C content is calculated from genome sequences (Meier-Kolthoff et al., 2014c). The G+C values cited in many species descriptions are often out of sync with those directly calculated from genome sequences, which also have a significantly better fit to the phylogeny than the former (Hahnke et al., 2016). Emendations to species descriptions are needed where estimates from experimental methods differ by more than 1% from *in silico* derived values (Meier-Kolthoff et al., 2014c). Similarly, description of higher ranked taxa should be emended if the range of G+C values are shown to be in conflict with corresponding values determined from genome sequences. In the same vein, digital DNA:DNA hybridization (DDH) values provide a more accurate way of establishing relationships between closely related species than corresponding data derived from experimental methods (Auch et al., 2010a,b; Meier-Kolthoff et al., 2013a), whereas genome size has as yet been less well elucidated as taxonomic marker.

Results from incorporating new technologies into systematics often raise the question whether discrepancies to earlier outcomes, if any, are caused by conflicts in the data themselves, such as between the phenotype, single genes or entire genomes, or by conflicting approaches of data interpretation such as those between the distinct schools of taxonomy (Klenk and Göker, 2010). In phylogenetic systematics only monophyletic taxa can be accepted in taxonomic classifications as they are designed to summarize the phylogeny of the classified organisms (Hennig, 1965; Wiley and Lieberman, 2011). While principles of phylogenetic systematics should also be used to guide microbial classification (Klenk and Göker, 2010; Hahnke et al., 2016), they have specific implications on how single characters, such as phenotypic ones, should be interpreted, even though this has not been widely considered in current taxonomic practice in microbiology (Montero-Calasanz et al., 2017).

The present study was designed to provide an improved framework for the classification of actinobacteria based on the principles of phylogenetic systematics, in line with our earlier study on *Bacteroidetes* (Hahnke et al., 2016). To this end, a comprehensive sampling of publicly available whole genome sequences of actinobacterial type strains was used to construct genome-scale phylogenetic trees and to address the following questions: (i) to what extent are phylogenies calculated from whole genome sequences in conflict with current actinobacterial classification and with 16S (or 23S) rRNA gene phylogenies? (ii) Which taxa need to be revised because they are evidently non-monophyletic? (iii) What is the historical cause of these discrepancies? (iv) Which taxon descriptions should be modified because of inaccurate or missing G+C values? and (v) How does genome size relate to phylogeny and genomic G+C content and how can it serve as a taxonomic marker?

## Materials and methods

A total number of 1,142 actinobacterial (ingroup) and 25 chloroflexi (outgroup) type-strain genomes were downloaded mainly from GenBank and to a smaller degree from the Integrated Microbial Genomes platform (Markowitz et al., 2009), that originated from the Genomic Encyclopedia of *Archaea* and *Bacteria* pilot phase or phase 1 of the One Thousand Microbial Genomes project (Kyrpides et al., 2014; Mukherjee et al., 2017). GenBank sequences comprising more than 500 contigs were discarded. The complete genome list is found in Supplementary Table 1. Further processing of these genome sequences closely followed an earlier study on genome-based classification (Hahnke et al., 2016). The high-throughput version (Meier-Kolthoff et al., 2014a) of the Genome BLAST Distance Phylogeny (GBDP) approach (Auch et al., 2006) was used to infer genome-scale phylogenies from whole proteomes in conjunction with BLAST+ (v2.2.30) (Camacho et al., 2009) in BLASTP mode with default parameters except for an e-value filter of 10^−8^ (Meier-Kolthoff et al., 2014a). The greedy-with-trimming GBDP algorithm was applied in conjunction with formula *d*_5_ and subjected to 100 pseudo-bootstrap replicates (Meier-Kolthoff et al., 2013a, 2014a). FastME (Lefort et al., 2015) was used to infer phylogenetic trees from the original and pseudo-bootstrapped intergenomic distance matrices, and trees and support values were visualized using Interactive Tree Of Life (Letunic and Bork, 2016) in conjunction with the script found at https://github.com/mgoeker/itol for facilitating tree annotation. Digital DNA:DNA hybridization (dDDH) was conducted with the recommended settings of the Genome-To-Genome Distance Calculator (GGDC) version 2.1 (Meier-Kolthoff et al., 2013a) to clarify species and subspecies affiliations.

Genomic G+C content values and genome sizes (approximate genome sizes in the case of incompletely sequenced genomes) were calculated as previously described (Hahnke et al., 2016); they were also recorded for single contigs to detect potential contamination of genome sequences. For the subsequent statistical tests, which were conducted without the outgroup, G+C content values were logit-transformed and genome sizes were log-transformed to account for the dependence of the variance on the mean in the case of proportion and count data (Crawley, 2007). Phylogenetic conservation was determined using the phylosignal function from the *picante* package (Kembel et al., 2010) for the R statistical environment (R Development Core Team, 2014) with 9,999 permutations. Phylogenetically independent contrasts (Felsenstein, 1985) were determined using the *pic* function of the *ape* package (Paradis et al., 2004). The relationship between G+C content and genome size was analyzed with Pearson and Kendall correlations. Loess-based bootstrap aggregating, that is bagging (Breiman, 1996), was applied in R with 100 replicates to increase stability and reduce overfitting.

Comprehensive, aligned, near full-length 16S rRNA gene sequence data for the *Actinobacteria* and *Chloroflexi* were initially taken from the All-Species Living Tree Project (Yarza et al., 2008) and considerably augmented by screening the more recent taxonomic literature (the complete DSMZ reference collection comprises c. 16,000 type-strain 16S rRNA gene sequences of *Archaea* and *Bacteria*). All of the rRNA genes were extracted from the genome sequences using RNAmmer version 1.2 (Lagesen et al., 2007) and the extracted 16S rRNA genes were compared using BLAST search (and in ambiguous cases also with phylogenetic trees) with the 16S rRNA gene reference data base (Chun et al., 2018). Non-matching genome sequences were discarded. Sequences were aligned using MAFFT version 7.271 with the “localpair” option (Katoh et al., 2005). Depending on the length and number of ambiguous bases, either the sequences extracted from the genome sequences or the previously published 16S sequences were used. Trees were inferred from the alignment with RAxML under the maximum-likelihood (ML) criterion using GTR+CAT as model approximation (Stamatakis, 2014) and with TNT under maximum-parsimony (MP) (Goloboff et al., 2008), as previously described (Hahnke et al., 2016). In addition to these unconstrained, comprehensive 16S rRNA gene trees (UCT), constrained comprehensive trees (CCT) were inferred with ML and MP using the bipartitions of the GBDP tree with ≥95% support as backbone constraint. Finally, unconstrained 16S rRNA gene trees reduced to genome-sequenced strains were inferred, as well as unconstrained 23S (i.e., large subunit) rRNA gene trees (ULT).

All of the trees were compared with the current classification used in LTP version s123, which was cleaned from inconsistencies such as mismatches between species and genus names and subsequently modified manually by incorporating newer literature sources. For instance, we followed the recent abandonment of subclass and suborder categories in *Bergey's Manual of Systematic Bacteriology* (Ludwig et al., 2012). Taxa were checked to determine whether they were monophyletic, paraphyletic or polyphyletic (Farris, 1974; Wood, 1994) as previously described (Hahnke et al., 2016).

Taxa non-monophyletic according to the GBDP tree were tested for evidence for their monophyly in the ULT, UCT, and the 16S rRNA gene trees, if any, in the original publication. In the case of a significant conflict (i.e., high support values for contradicting bipartitions) or low support in the GBDP tree, additional phylogenomic analyses of selected taxa were conducted. To this end, the reciprocal best hits from GBDP/BLAST were clustered with MCL under default settings and an *e*-value filter of 10^−5^ in analogy to OrthoMCL (Li et al., 2003a). The resulting sets of orthologous proteins were aligned with MAFFT and concatenated to form a supermatrix. The few clusters that still contained more than a single protein for at least one genome were discarded. Core-genome supermatrices were constructed for the orthologs that occurred in all of the genomes, whereas comprehensive supermatrices were compiled from all the orthologs that occurred in at least four genomes. Supermatrices were analyzed with TNT, and with RAxML under the PROTCATLGF model (Le and Gascuel, 2008), in conjunction with 100 partition bootstrap replicates, i.e., by sampling (with replacement) the orthologs instead of the single alignment positions (Siddall, 2010; Simon et al., 2017).

Unambiguously non-monophyletic taxa according to the genome-scale analyses were screened for published phenotypic evidence of their monophyly. Published evidence was judged as inconclusive when based on probably homoplastic characters or on probable plesiomorphic character states. Importantly, “diagnostic” features alone are insufficient in phylogenetic systematics, as plesiomorphies might well be diagnostic but just for paraphyletic groups (Hennig, 1965; Wiley and Lieberman, 2011). Finally, taxonomic consequences were proposed to fix all obviously non-monophyletic taxa by new taxon delineations sufficiently supported by the CCT, i.e., not hindered by the uncertain phylogenetic placement of taxa whose genome sequences were not available at the time of writing.

## Results

**Figure 1:**
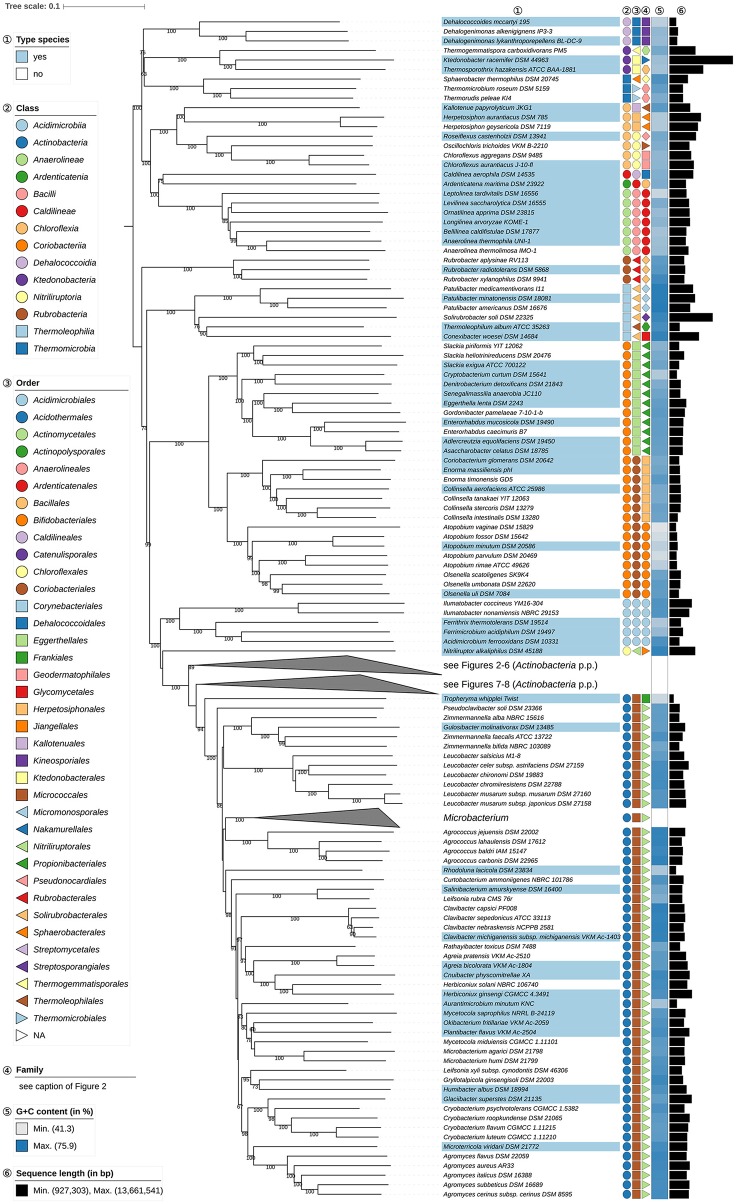
First part of the phylogenomic tree inferred with GBDP. Tree inferred with FastME from GBDP distances calculated from whole proteomes. The numbers above branches are GBDP pseudo-bootstrap support values from 100 replications. Tip colors indicate type species of genera, colors to the right of the tips indicate, from left to right, class, order and family (see the embedded legend for details, Figure 2 for the families). The blue gradient toward the far right indicates the exact G+C content as calculated from the genome sequences, followed by black bars indicating the (approximate) genome size in bp. The parts of the tree which have been collapsed here are shown in Figures 2–8.

**Figure 2:**
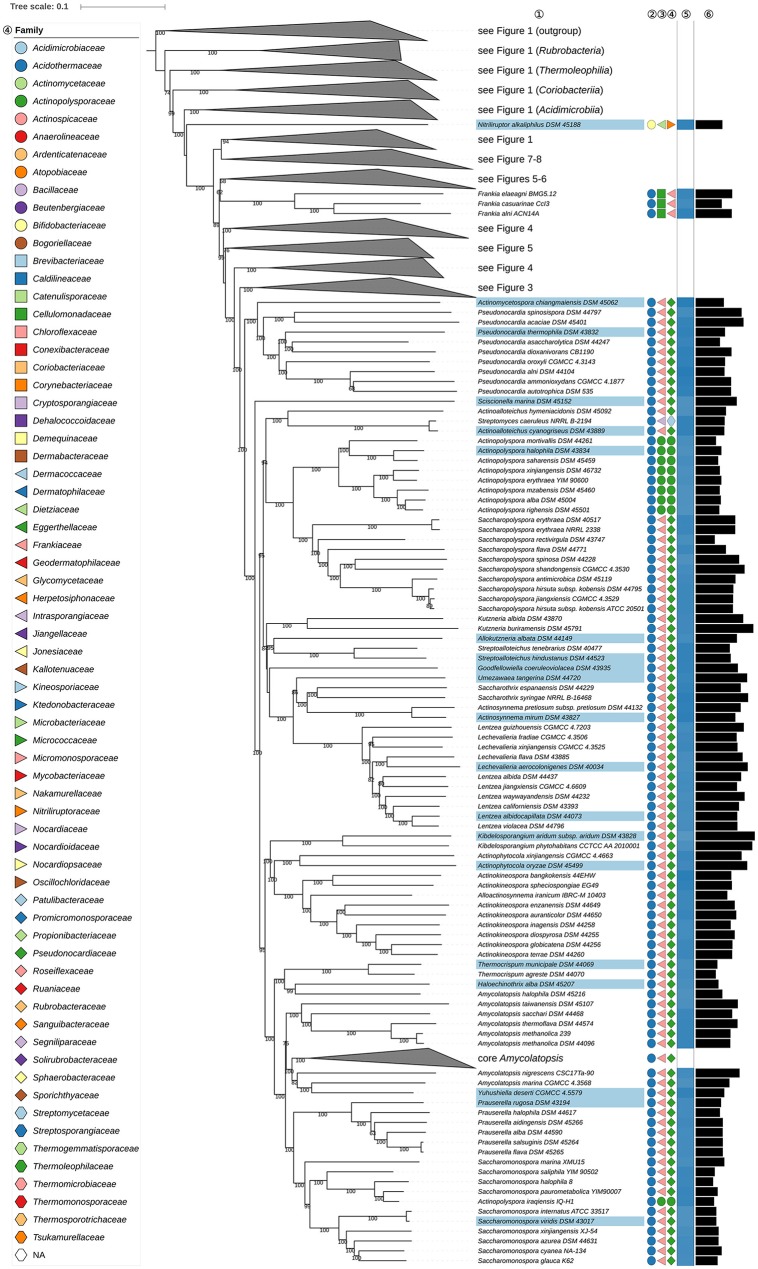
Second part of the phylogenomic tree inferred with GBDP. A detailed description is provided in the caption of Figure 1. The parts of the tree which have been collapsed here are shown in other figures as indicated.

**Figure 3:**
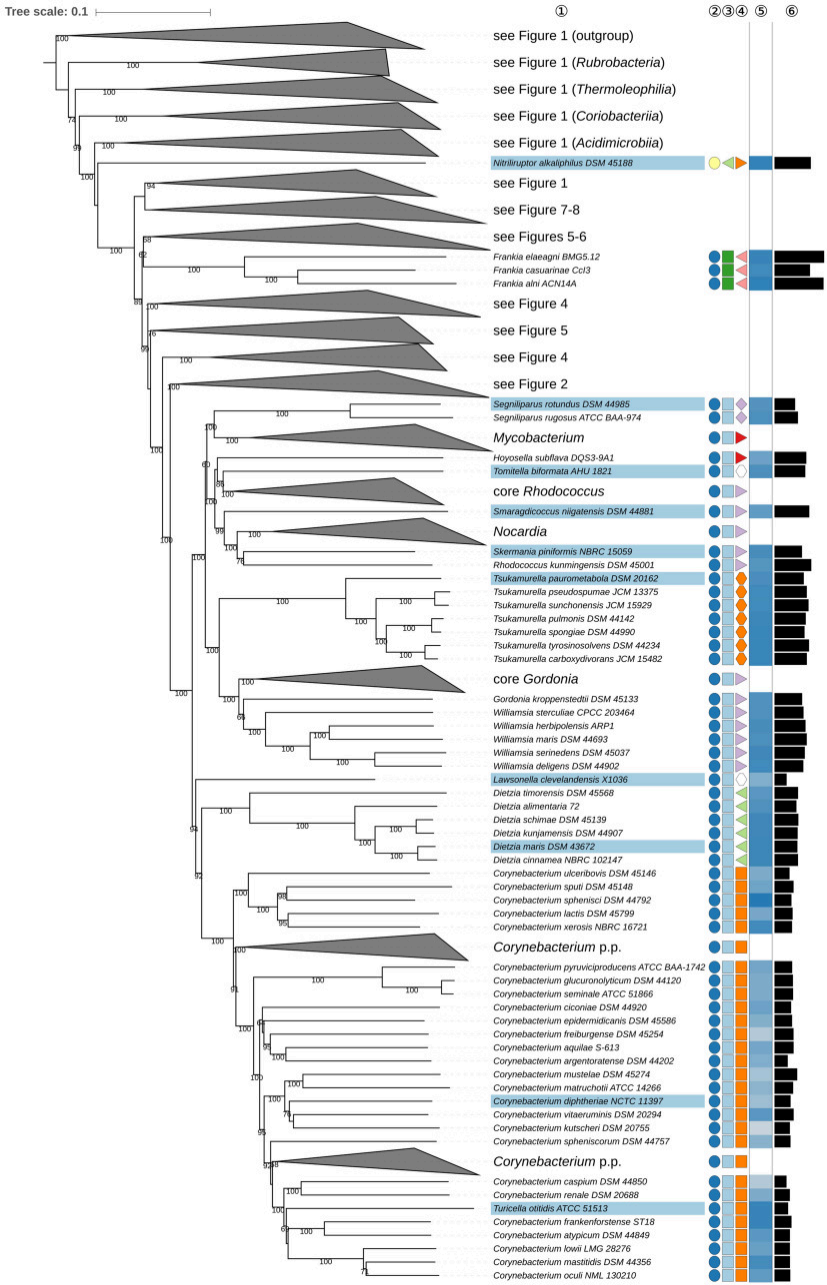
Third part of the phylogenomic tree inferred with GBDP. A detailed description is provided in the caption of Figure 1. The parts of the tree which have been collapsed here are shown in other figures as indicated.

**Figure 4:**
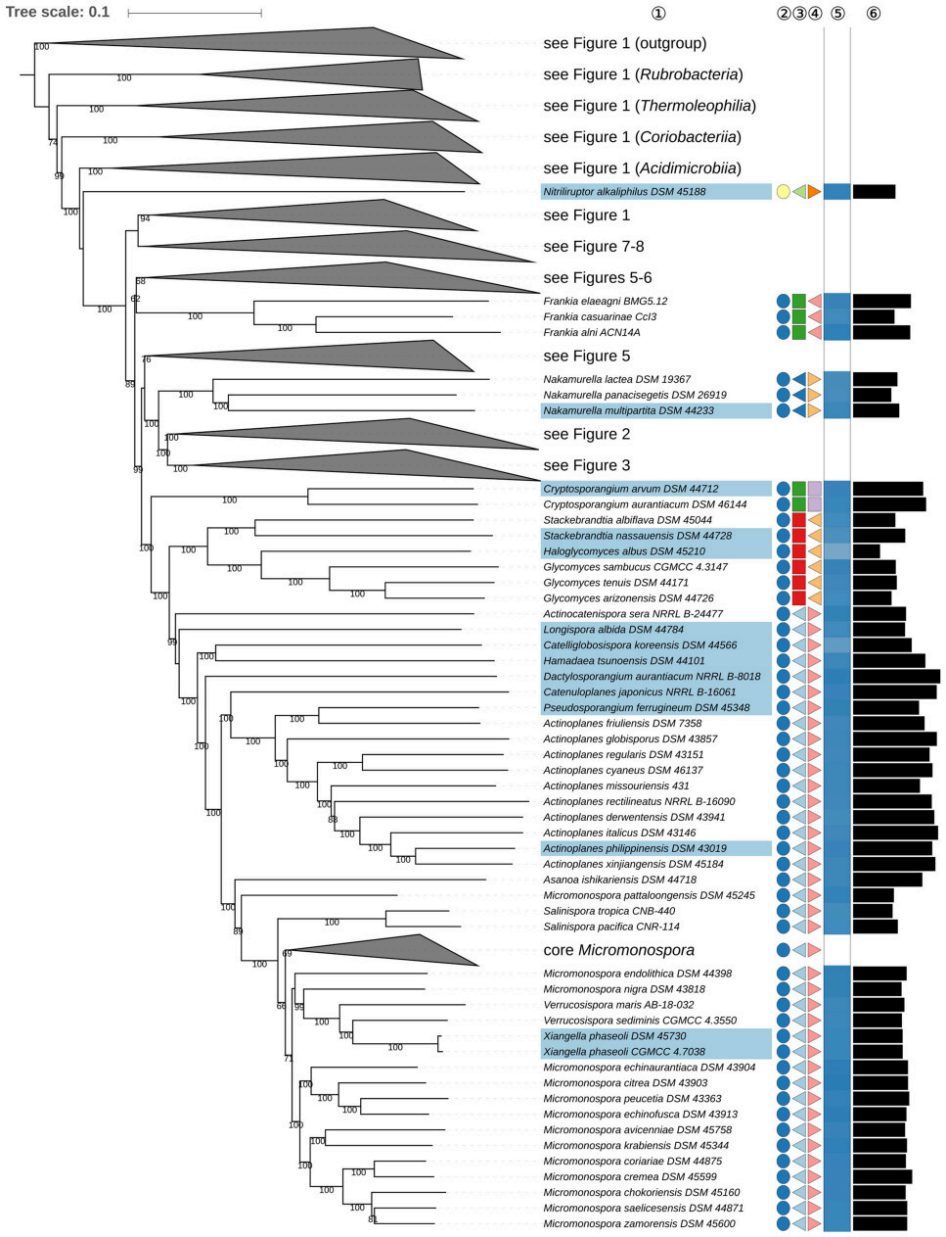
Fourth part of the phylogenomic tree inferred with GBDP. A detailed description is provided in the caption of Figure 1. The parts of the tree which have been collapsed here are shown in other figures as indicated.

**Figure 5:**
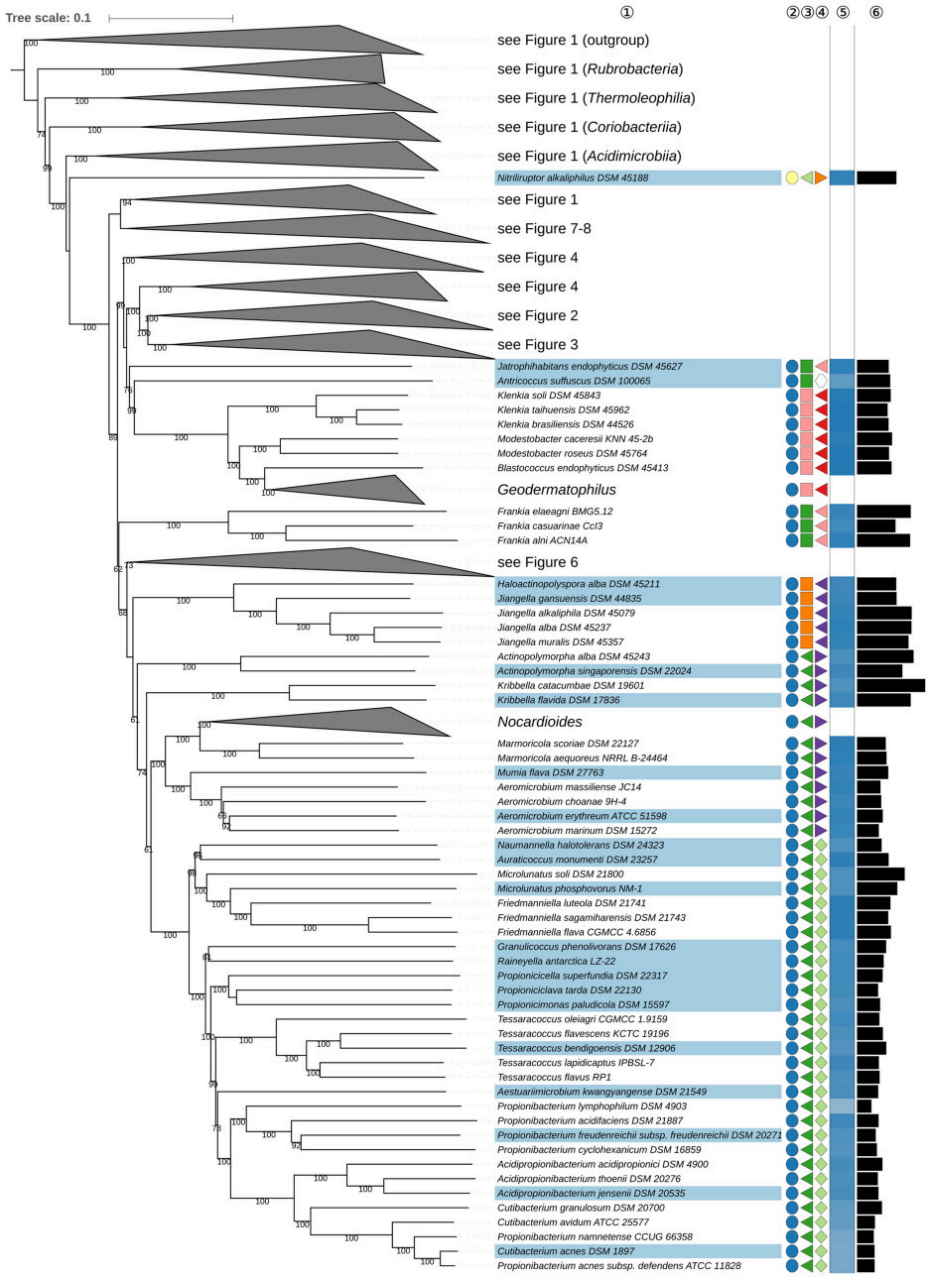
Fifth part of the phylogenomic tree inferred with GBDP. A detailed description is provided in the caption of Figure 1. The parts of the tree which have been collapsed here are shown in other figures as indicated.

**Figure 6:**
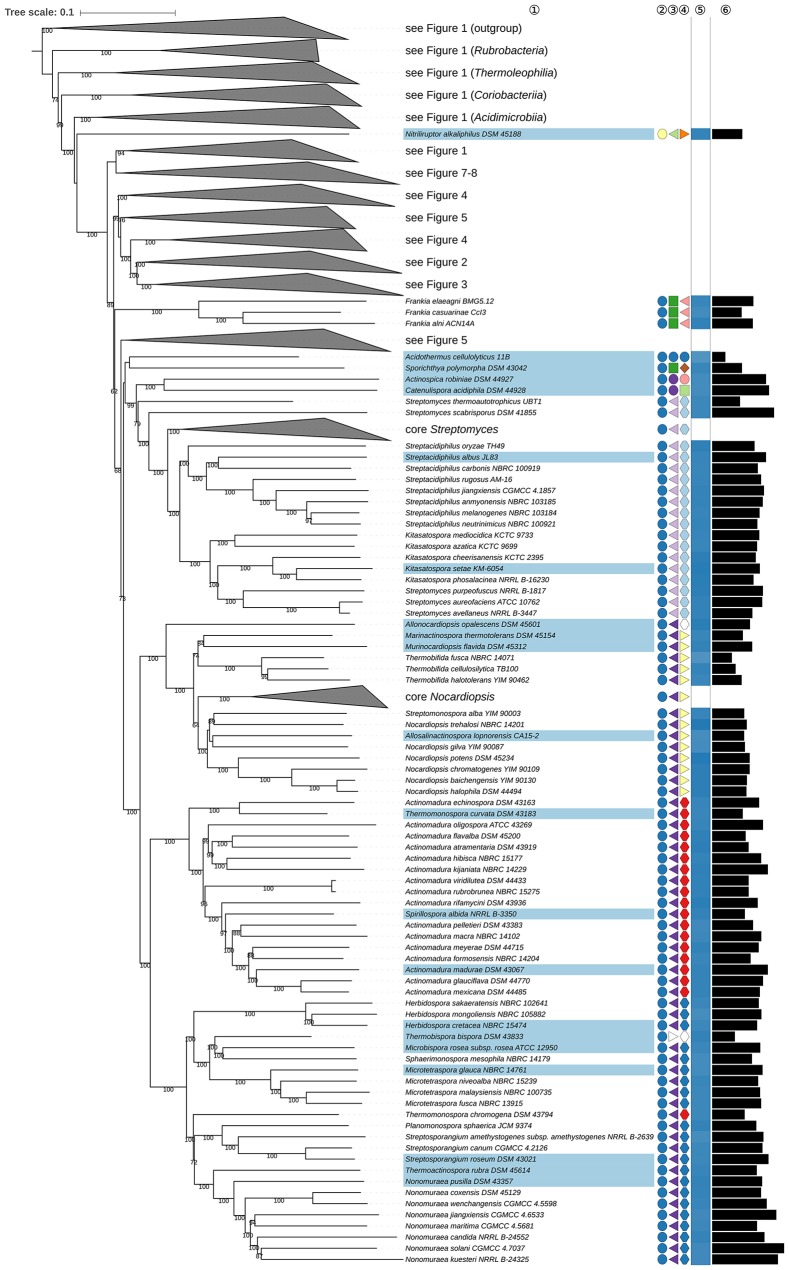
Sixth part of the phylogenomic tree inferred with GBDP. A detailed description is provided in the caption of Figure 1. The parts of the tree which have been collapsed here are shown in other figures as indicated.

**Figure 7:**
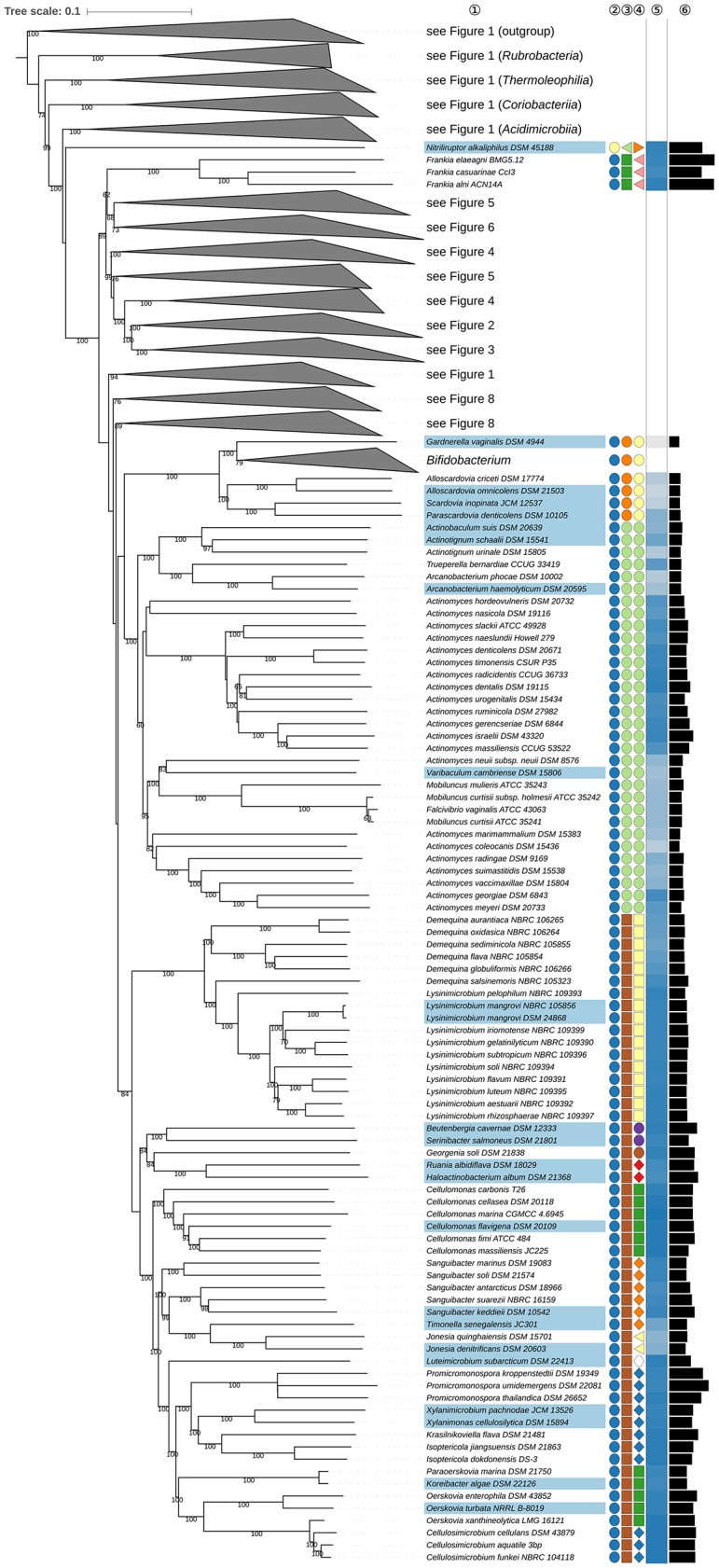
Seventh part of the phylogenomic tree inferred with GBDP. A detailed description is provided in the caption of Figure 1. The parts of the tree which have been collapsed here are shown in other figures as indicated.

**Figure 8:**
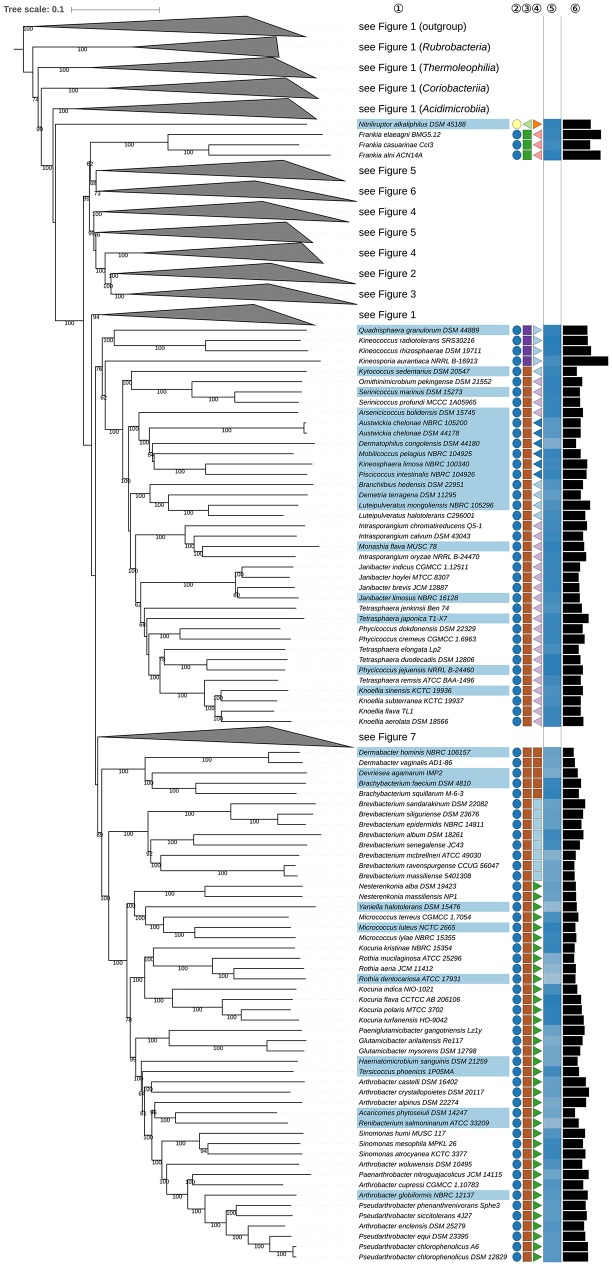
Eighth part of the phylogenomic tree inferred with GBDP. A detailed description is provided in the caption of Figure 1. The parts of the tree which have been collapsed here are shown in other figures as indicated.

The phylogenetic tree inferred with GBDP is shown in Figures 1–**8**. The tree with all of the branches expanded is shown in Supplementary File 2 together with results from additional 16S rRNA gene (UCT, CCT, reduced tree), 23S rRNA gene (ULT), and supermatrix analyses, as well as a juxtaposition of GBDP and 16S rRNA gene support values. Supplementary Table 1 also contains a tabular overview of taxonomically relevant phenotypic features taken from the literature for taxa of interest. All of the classes and most of the orders, families, and genera appear to be monophyletic, mainly with high support but other taxa are in need of taxonomic revision according to the GBDP analysis. These discrepancies are considered below in decreasing order of taxonomic rank.

### Orders

*Solirubrobacterales* (Reddy and Garcia-Pichel, 2009) appear to be paraphyletic because of the position of *Thermoleophilum album* (Zarilla and Perry, 1984) of *Thermoleophilales* (Figure 1). However, the two branches responsible for this arrangement are poorly supported whereas the CCT, ULT, and an additional supermatrix analysis show that *Solirubrobacterales* are monophyletic (Supplementary File 2). It is evident that these taxa have phenotypic features in common, albeit unspecific ones (Supplementary Table 1). It can be concluded that taxonomic changes are not presently needed with respect to *Solirubrobacterales* and *Thermoleophilales*.

The position of *Actinopolysporales* (and *Actinopolysporaceae*) in the phylogenomic tree (Figure 2) causes *Pseudonocardiales* (and *Pseudonocardiaceae*) to be paraphyletic, as *Actinopolyspora* (Gochnauer et al., 1975), apart for *A. iraqiensis* (Ruan et al., 1994), appears to be a highly supported as sister group to *Saccharopolyspora* (Lacey and Goodfellow, 1975). *Actinopolysporales* arose from the elevation of *Actinopolysporineae* which in turn was based on signature nucleotides and the original 16S rRNA gene tree though the latter was defined with rather low backbone support; it was not clear whether these properties represent apomorphies (Zhi et al., 2009). In the CCT *Bounagaea* (Meklat et al., 2015) of *Pseudonocardiales* and *Halopolyspora* (Lai et al., 2014) of *Actinopolysporaceae* were also found to be part of the clade encompassing representative *Actinopolysporaceae* and *Pseudonocardiaceae* for which the UCT provided reasonable support. *Mzabimyces algeriensis*, for which *Mzabimycetaceae* fam. nov. had been suggested based on a largely unresolved 16S rRNA gene tree (Saker et al., 2014), was later regarded as a heterotypic synonym of *Halopolyspora algeriensis* (Lai et al., 2017). The phylogenetic results, even including those based only on the 16S rRNA gene, indicate that *Actinopolysporaceae* and *Mzabimycetaceae* should be classified in *Pseudonocardiaceae*, a taxonomically most conservative solution, and that *Actinopolysporales* be disbanded. It is interesting in this context that *Actinopolyspora* was initially classified in *Pseudonocardiaceae* (Embley et al., 1988), and that the phenotypic differences between *Actinopolysporaceae* and *Pseudonocardiaceae* are not pronounced (Supplementary Table 1).

*Frankiales*, as effectively published (Normand and Benson, 2012), encompasses in addition to *Fodinicola* (genus *incertae sedis*) nine genera, which are classified into *Frankiaceae* (Becking, 1970), *Acidothermaceae* (Stackebrandt et al., 1997), *Cryptosporangiaceae* (Zhi et al., 2009), *Geodermatophilaceae* (Normand, 2006), *Motilibacteraceae* (Lee, 2013b), *Nakamurellaceae* (Tao et al., 2004), and *Sporichthyaceae* (Stackebrandt et al., 1997). A phylogenomic study (Sen et al., 2014) assigned six of these families to four new orders, *Frankiales, Acidothermales, Geodermatophilales*, and *Nakamurellales*. The two remaining genera were *Cryptosporangium* (Tamura et al., 1998) and *Sporichthya* (Lechevalier et al., 1968). In the phylogenetic tree (**Figures 4**, **6**), these genera were found to be only distantly related to the type genus of *Frankiales, Frankia* (Nouioui et al., 2016). *Cryptosporangium* forms a well-supported clade together with *Glycomycetale*s (Labeda, 2012) and *Micromonosporale*s (Genilloud, 2012) hence raising the prospect that the genus be assigned to a new order, *Cryptosporangiales*, a move in line with the isolated position of the genus in the CCT and ULT (Supplementary File 2). As detailed in Supplementary Table 1, there are no common phenotypic features between *Cryptosporangium, Frankia, Glycomycetales*, and *Micromonosporales*, apart from the unspecified peptidoglycan component DL-A_2_pm, i.e., *mes*o-diaminopimelic acid (Genilloud, 2012; Labeda, 2012; Normand and Benson, 2012).

It is evident from Figure **5** that the position of *Jatrophihabitans* (Madhaiyan et al., 2013) is at variance with its assignment to *Frankiales*. Phenotypical synapomorphies of *Frankia* and *Jatrophihabitans* are not known. *Jatrophihabitans* forms short rods while frankiae form hyphae, multilocular sporangia and vesicles in which nitrogen is fixed (Supplementary Table 1). The position of *Jatrophihabitans* as the sister group to the clade comprising *Antricoccus* (Lee, 2015) and *Geodermatophilaceae* is only weakly supported (**Figure 5**). In the ULT (Supplementary File 2), *Antricoccus* and *Jatrophihabitans* were found to be sister groups albeit with weak support within *Geodermatophilales*. In light of these results, it is proposed that *Jatrophihabitans* be recognized as the type genus of *Jatrophihabitandaceae* fam. nov. without an assignment to an order. This proposal is not in conflict with the 16S rRNA gene tree shown in the initial description of the genus because the tree was poorly resolved.

*Antricoccus* (Lee, 2015), which is currently in search of a family, was found to be more closely related in the phylogenomic tree to *Geodermatophilales* than to *Frankiales* (**Figure 5**). Indeed, given its distinct position in the tree, it is best seen as a separate family within *Geodermatophilales* rather than placed within *Geodermatophilaceae*, from which it also differs in genomic G+C content (**Figure 5**). This proposition is not contradicted by the poorly resolved 16S rRNA gene tree presented in the original description of *Antricoccus*. Phenotypical synapomorphies of *Antricoccus*, which forms non-motile, asporogenous cocci, and *Frankia* are not known either (Supplementary Table 1).

It is evident from the GBDP tree (**Figure 6**) that *Sporichthya* (Lechevalier et al., 1968) of *Sporichthyaceae* (Stackebrandt et al., 1997) is separated from *Frankiaceae. Sporichthya* appears more closely related to *Acidothermus cellulolyticus* (Mohagheghi et al., 1986) of *Acidothermaceae* but without support. The sister-group relationship between *Acidothermus* and *Sporichthya* is not supported by either the CCT or ULT (Supplementary File 2) nor are synapomorphies of these taxa known. *Sporichthya* is motile and has LL-A_2_pm in the peptidoglycan whereas *Acidothermus* is non-motile, thermophilic with DL-A_2_pm in the peptidoglycan. Given these findings it is proposed that *Sporichthya* be recognized as the type genus of *Sporichthyales* ord. nov.

The relationship between *Fodinicola* (Carlsohn et al., 2008) and *Cryptosporangiaceae* was not resolved in the UCT (Supplementary File 2); the CCT showed it to have an isolated position, albeit with weak support. Likewise, the affiliation of *Motilibacter* (Lee, 2012, 2013b) to *Frankiales* is questionable; the UCT and CCT place this genus close to *Streptomycetales*, but with low support. *Fodinicola* and *Motilibacter*, originally assigned to *Frankiales*, might best be classified as genera *incertae sedis* until their position is clarified once more genome sequences become available.

*Kineosporiales* (Kämpfer, 2012b) and *Micrococcales* (Prévot, 1940) did not appear to be monophyletic though support to this effect is low in all the phylogenetic analyses (Figures 1–**8**, Supplementary File 2). The taxonomy of these orders needs to be revisited once more genome sequences become available.

### Families

Within *Corynebacteriales* (Goodfellow and Jones, 2012), *Mycobacteriaceae* (Chester, 1897; Stackebrandt et al., 1997; Zhi et al., 2009) contains the type genus *Mycobacterium* (Lehmann and Neumann, 1896) and *Hoyosella* (Jurado et al., 2009). *Mycobacteriaceae* appear to be paraphyletic in the phylogenomic tree (Figure 3) given the position of *H. subflava* (Hamada et al., 2016). Support for the monophyly of *Mycobacteriaceae* is strong in the 16S rRNA gene tree reduced to strains with genome sequences, but decreases when more sequences are considered; neither the UCT nor the ULT resolve these relationships (Supplementary File 2). Additional supermatrix analyses of *Corynebacteriales* type species confirm the non-monophyly of *Mycobacteriaceae*, as indicated in the GBDP tree. *Tomitella* (Katayama et al., 2010), currently in search of a family, was, like *H. subflava*, found within *Nocardiaceae* with high support in the phylogenomic tree (Figure 3). The proposed transfer of *Hoyosella* and *Tomitella* to *Nocardiaceae* is not contradicted by phenotypic features (Supplementary Table 1); for instance, mycolic acid carbon chain lengths of these genera are within the range known for the family.

The unresolved phylogenetic relationships of *Gordonia* (Tsukamura, 1971; Stackebrandt et al., 1988) and *Williamsia* (Kämpfer et al., 1999) relative to other *Nocardiaceae* as inferred from 16S rRNA gene sequences were noted previously (Goodfellow and Jones, 2012). In the present study, *Gordonia* and *Williamsia* form a well-supported clade together with *Tsukamurella* (Collins et al., 1988b) which was distinct from core *Nocardiaceae* (Figure 3). In the UCT and the ULT (Supplementary File 2) the backbone of the *Corynebacteriales* subtree was not resolved. The supermatrix analyses of the type species of *Corynebacteriales* confirmed the sister-group relationship of the *Gordonia-Williamsia* clade with *Tsukamurella*. A case can be made for assigning *Gordonia* and *Williamsia* to *Tsukamurellaceae*, but this prospect is less conservative than reassigning them to *Gordoniaceae* (Stackebrandt et al., 1997). Moreover, their inclusion in *Nocardiaceae* was mainly based on a 16S rRNA gene tree with low backbone support and on signature nucleotides but it was not clear whether these properties represented apomorphies (Zhi et al., 2009). *Gordonia* and *Williamsia* share phenotypic features with *Tsukamurellaceae* (Supplementary Table 1) though such properties are not necessarily apomorphic. Consequently, it is proposed that *Gordonia* and *Williamsia* be classified, as before, in *Gordoniaceae* (Stackebrandt et al., 1997).

The monospecific genus *Lawsonella* was proposed previously (Bell et al., 2016) for a clade that appeared most closely related in 16S rRNA gene trees to a clade that encompassed *Corynebacterium, Dietzia*, and *Tsukamurella*. It is evident from the GBDP tree (Figure 3) that *L. clevelandensis* (Bell et al., 2016) does not belong to any of the known *Corynebacteriales* families. It forms, with moderate support, the sister group of a clade that includes *Corynebacteriaceae* (Lehmann and Neumann, 1907; Stackebrandt et al., 1997; Zhi et al., 2009) and *Dietziaceae* (Stackebrandt et al., 1997), relationships underpinned by supermatrix analyses of *Corynebacteriales* type species (Supplementary File 2). Given these phylogenetic results it is proposed that *Lawsonella* be seen as the type genus of *Lawsonellaceae* fam. nov.

*Propionibacteriales* (Patrick and McDowell, 2012) contain *Nocardioidaceae* (Nesterenko et al., 1985; Stackebrandt et al., 1997; Zhi et al., 2009) and *Propionibacteriaceae* (Delwiche, 1957; Stackebrandt et al., 1997; Zhi et al., 2009). In addition to *Nocardioides* (Prauser, 1976), the type genus, *Nocardioidaceae* encompass genera such as *Actinopolymorpha* (Wang et al., 2001) *Flindersiella* (Kaewkla and Franco, 2011), *Kribbella* (Park et al., 1999; Sohn et al., 2003; Everest et al., 2013), *Tenggerimyces* (Sun et al., 2015), and *Thermasporomyces* (Yabe et al., 2011). *Nocardioidaceae* appear to be paraphyletic with respect to *Propionibacteriaceae* given the placing of *Actinopolymorpha* and *Kribbella* (**Figure 5**) though the branch support for the distant positions of these genera in the GBDP tree is not pronounced. However, there is a need to revise the classification of the *Nocardioidaceae* due to the genetic divergence it contains.

The positions of *Actinopolymorpha* and *Kribbella* are underpinned with relatively high support in the ULT (Supplementary File 2). The CCT and the UCT show *Propionibacteriaceae* and core *Nocardioidaceae* to be sister groups given the exclusion of *Actinopolymorpha* and *Kribbella*. In this context, it is interesting that *Actinopolymorpha* and *Kribbella* have much larger genomes than the clade composed of core *Nocardioidaceae* and *Propionibacteriaceae* indicating that reduced genome size is a synapomorphy in the latter (**Figure 5**). The heterogeneity of the *Nocardioidaceae* with respect to genomic and phenotypic properties has been noted (Evtushenko and Ariskina, 2012): *Actinopolymorpha, Kribbella, Flindersiella, Tenggerimyces*, and *Thermasporomyces* are non-motile and form branched hyphae whereas most of the other *Nocardioidaceae* form motile rods or cocci; *Kribbella* is the only one of these genera in which teichuronic and teichulosonic acids have been detected (Tul'skaya et al., 2011). Finally, a highly supported clade in the CCT (Supplementary File 2) includes *Actinopolymorpha, Flindersiella, Tenggerimyces* and *Thermasporomyces*, but not *Kribbella*. Given this wealth of genomic and phenotypic data, it is proposed that two additional families be recognized within *Propionibacteriales*.

The monospecific genus *Thermobispora* (Wang et al., 1996) appears as a distinct lineage within *Streptosporangiales* based on an analysis of 16S rRNA gene sequences, but without a clear association to any of the described families (Ludwig et al., 2012) hence its assignment to an order *incertae sedis* in the current edition of *Bergey's Manual of Systematic Bacteriology* (Goodfellow et al., 2012a). It is apparent from the phylogenomic tree (**Figure 6**) that *T. bispora* (Henssen, 1957; Wang et al., 1996) forms the sister group to a clade encompassing *Microbispora* (Nonomura and Ohara, 1957) and *Sphaerimonospora* (Katayama et al., 2010), together these taxa form the sister group to *Microtetraspora* (Thiemann et al., 1968). All three of these genera belong to *Streptosporangiaceae* (Goodfellow et al., 1990; Stackebrandt et al., 1997). *Thermobispora* can be distinguished from the current *Streptosporangiaceae* genera given its thermophilic nature, formation of pairs of spores on aerial hyphae and presence of MK-9(H_0_) as the predominant menaquinone (Wang et al., 1996). In contrast, *Sphaerimonospora mesophila* (Mingma et al., 2016), the type species of the genus, forms single spores on aerial hyphae and has MK-9(H_4_) as the predominant isoprenologue (Supplementary Table 1). Given these developments it is proposed that *Thermobispora* be classified in *Streptosporangiaceae* but kept in a genus of its own.

*Sanguibacteraceae* (Stackebrandt and Schumann, 2000; Zhi et al., 2009) appear paraphyletic because *Jonesiaceae* (Stackebrandt et al., 1997; Zhi et al., 2009), represented by *Jonesia* (Rocourt et al., 1987), were more closely related to *Timonella* (Mishra et al., 2013), and to some *Sanguibacter* species (Fernández-Garayzábal et al., 1995) than to other species in this genus with high support in the GBDP tree (**Figure 7**). The *Jonesia*-*Timonella* clade also shows lower genomic G+C content and genome size compared to the surrounding genera, a probable synapomorphy. Additional supermatrix analyses of selected species confirmed these relationships (Supplementary File 2). In the CCT the *Jonesia* and *Timonella* clade also includes *Populibacterium* (Li et al., 2016) from the same family and *Rarobacter* (Yamamoto et al., 1988; Li et al., 2016) of *Rarobacteraceae* (Stackebrandt and Schumann, 2000). *Jonesiaceae* and *Sanguibacteraceae* share key chemotaxonomic markers, as exemplified by the presence of L-Lysine as the cell wall diamino acid, MK-9 or MK-9(H_4_) as the predominant isoprenologue and *anteiso*-C_15:0_ and C_16:0_ as major fatty acids (Supplementary Table 1). In light of the genomic and phenotypic data, it is proposed that *Sanguibacteraceae* be reduced to a synonym of *Jonesiaceae*. It seems likely that further comparative taxonomic studies will show *Rarobacteraceae*, which like the other two families was based on signature nucleotides, for which it remained unclear whether they are apomorphies, and a largely unresolved 16S rRNA tree (Stackebrandt and Schumann, 2000), to belong to *Jonesiaceae* (Zhi et al., 2009). The presence of L-ornithine in the *Rarobacter* peptidoglycan does not preclude this, as its absence in the other genera might simply be the plesiomorphic state in the *Jonesiaceae-Rarobacteraceae-Sanguibacteraceae* clade.

*Cellulomonadaceae* (Stackebrandt and Prauser, 1991; Zhi et al., 2009) appear polyphyletic because *Paraoerskovia* (Khan et al., 2009) and *Oerskovia* (Prauser et al., 1970) are phylogenetically located within *Promicromonosporaceae* (Stackebrandt et al., 1997; Zhi et al., 2009), forming a highly supported clade together with *Cellulosimicrobium* (Schumann et al., 2001), whereas *Tropheryma* (La Scola et al., 2001) is placed in a completely different position as sister group to *Microbacteriaceae* (Park et al., 1993) albeit with not particularly high support (Figures 1, **7**). The ULT does not resolve the position of *Oerskovia* and *Paraoerskovia* but shows the *Microbacteriaceae-Tropheryma* clade with maximum support (Supplementary File 2). Thus, none of the three genera appear phylogenetically closely related to the type genus *Cellulomonas* (Bergey et al., 1923). The transfer of *Paraoerskovia* (including *Koreibacter*, a known later heterotypic) and *Oerskovia* (excluding *O. xanthineolytica*, also a later heterotypic synonym) to *Promicromonosporaceae* is supported by several common phenotypic and genotypic features (Supplementary Table 1). *Luteimicrobium* (Hamada et al., 2010), currently in search of a family, is shown in Figure **7** as the sister group of an accordingly revised *Promicromonosporaceae* with strong support; hence we propose to also include it in this family. Except for a minor deviation regarding the isoprenologues their phenotypic features are in good agreement (Supplementary Table 1). The enigmatic, poorly characterized *Tropheryma* should, given its isolated phylogenetic position, best be transferred to it own family, currently without an assignment to an order.

*Dermacoccaceae* (Stackebrandt and Schumann, 2000; Zhi et al., 2009; Ruckmani et al., 2011) is not shown as monophyletic because of the position of *Kytococcus* (Stackebrandt et al., 1995) in the GBDP tree (Figure **8**). The CCT (Supplementary File 2) shows the type genus, *Dermacoccus*, the genomes of which have not yet been sequenced, located, with high support, within the *Branchiibius*-*Demetria*-*Luteipulveratus* clade whereas *Kytococcus* does not form a well-supported sister group to this taxon. The ULT does not resolve the position of *Kytococcus* and hence does not support the monophyly of *Dermacoccaceae*. The probable misclassification of *Kytococcus* within *Dermacoccaceae* has been noted (Stackebrandt and Schumann, 2014) based on chemotaxonomic evidence (Supplementary Table 1) and on its position within the 16S rRNA gene tree. In light of all these results, it is proposed that *Kytococcus* be classified in *Kytococcaceae* fam. nov., within *Micrococcales* (Prévot, 1940).

*Intrasporangiaceae* (Stackebrandt et al., 1997; Zhi et al., 2009) also belong to *Micrococcales*, currently encompassing genera such as *Intrasporangium* (Kalakoutskii et al., 1967), the type genus, *Arsenicicoccus* (Collins et al., 2004b), *Kribbia* (Jung et al., 2006), *Ornithinibacter* (Xiao et al., 2011a), *Ornithinicoccus* (Groth et al., 1999a; Zhang et al., 2016b), *Serinicoccus* (Yi et al., 2004; Traiwan et al., 2011; Xiao et al., 2011b), and *Ornithinimicrobium* (Groth et al., 2001). It is evident from the GBDP tree (**Figure 8**) that the family is paraphyletic as *Serinicoccus* and *Ornithinimicrobium* appear to form the sister group to *Kytococcus* and are more distantly related to other *Intrasporangiaceae*; similar relationships are apparent in the ULT, albeit without strong support. The CCT (Supplementary File 2) shows that *O. humiphilum* (Groth et al., 2001), the type species of the genus, also belongs to the *Ornithinimicrobium-Serinicoccus* clade. These genera have ornithine as the diamino acid of the peptidoglycan, apart from *S. chungangensis* (Traiwan et al., 2011), which contains DL-A_2_pm whereas, in the main, *Intrasporangiaceae* lack ornithine (Supplementary Table 1). Consequently, it is proposed that *Ornithinimicrobium* and *Serinicoccus* be classified in *Ornithinimicrobiaceae* fam. nov.; it remains to be seen whether the other ornithine-containing genera, *Ornithinibacter* (Xiao et al., 2011a) and *Ornithinicoccus* (Groth et al., 1999a; Zhang et al., 2016b) belong to this family; the CCT does not settle this question.

In **Figure 8** *Arsenicicoccus* (Collins et al., 2004b) appears to form the sister group to *Dermatophilaceae* (Austwick, 1958; Stackebrandt et al., 1997; Stackebrandt and Schumann, 2000; Zhi et al., 2009) (**Figure 8**). These taxa share a number of phenotypic properties (Supplementary Table 1), notably a facultatively anaerobic lifestyle (Jung et al., 2006). All of the other *Intrasporangiacae* are aerobic, apart from *Kribbia* which is a facultative anaerobe. The assignment of *Arsenicicoccus* to *Intrasporangiaceae* was based on chemotaxonomic data, notably the presence of LL-A_2_pm in the peptidoglycan, and on a poorly resolved 16S rRNA gene tree. In line with the recent evidence, it is proposed that *Arsenicicoccus* be classified in *Dermatophilaceae*. It is possible that *Kribbia* may be recovered in this family when genomic data are generated for this genus.

### Genera

It can be seen from the phylogenomic tree (Figures 1–**8**) that a broad range of genera are non-monophyletic, as exemplified by *Corynebacterium* (Lehmann and Neumann, 1896; Bernard et al., 2010), *Kocuria* (Stackebrandt et al., 1995), *Lechevalieria* (Labeda et al., 2001), *Kitasatospora* (Omura et al., 1982; Wellington et al., 1992; Zhang et al., 1997), *Streptomyces* (Waksman and Henrici, 1943; Witt and Stackebrandt, 1990; Wellington et al., 1992), *Actinomyces* (Harz, 1877), *Lawsonella* (Bell et al., 2016), *Rhodococcus* (Zopf, 1891), *Actinomadura* (Lechevalier and Lechevalier, 1970; Zhao et al., 2015), *Thermomonospora* (Henssen, 1957; Zhang et al., 1998), *Zimmermannella* (Lin et al., 2004), and *Amycolatopsis* (Lechevalier et al., 1986; Lee, 2009; Tang et al., 2010a). In most of these cases, 16S rRNA gene sequence analyses (Supplementary File 2) had revealed the same taxonomic problems albeit with low support for delineated clades (Supplementary File 2).

In the GBDP tree the position of *Gulosibacter* (Manaia et al., 2004) makes *Zimmermannella* (Lin et al., 2004) appear paraphyletic (Figure 1). However, the name *Zimmermannella* has no standing in nomenclature as it is a later homotypic synonym of *Pseudoclavibacter* (Manaia et al., 2004) because its type species, *Z. helvola* (Lin et al., 2004) is a homotypic synonym of *P. helvolus* (Manaia et al., 2004) which means that *Z. alba* (Lin et al., 2004), *Z. bifida* (Lin et al., 2004), and *Z. faecalis* (Lin et al., 2004) and *Z. helvola* are also illegitimate. The UCT did not resolve the status of *Zimmermannella* species, but the CCT showed an unambiguously supported clade composed of *G. molinativorax* (Manaia et al., 2004), *Z. bifida*, and *Z. faecalis* though the placement of the other *Zimmermannella* species was uncertain (Supplementary File 2); the reported phenotypic differences between the species (Manaia et al., 2012) are not pronounced (Supplementary Table 1). Given these clear-cut phylogenetic relationships and the need to provide legitimate names for *Zimmermannella* species, it is proposed that *Z. bifida* and *Z. faecalis* be transferred to *Gulosibacter*. The taxonomic status of *Z. alba* may be resolved when genomic sequences become available for *Pseudoclavibacter*.

*Atopobium* (Collins and Wallbanks, 1992; Cools et al., 2014), the type genus of *Atopobiaceae* (Gupta et al., 2013) appears to be paraphyletic in the phylogenomic tree (Figure 1) as *Olsenella* (Dewhirst et al., 2001; Kraatz et al., 2011) forms the sister group to *A. parvulum* and *A. rimae* with high support*;* this relationship is also apparent in 16S rRNA gene phylogenies (Cools et al., 2014), in the latter case with insufficient resolution unless constrained (Supplementary File 2). The CCT shows a core *Atopobium* composed of *A. minutum*, the type species, *A. deltae* and *A. fossor* (the genome of *A. deltae* has yet to be sequenced); this clade was highly supported in the UCT. The ULT underpins the distinctness of the three *Atopobium* clades, but surprisingly shows *Atopobium* to be monophyletic and *Olsenella* paraphyletic. Supermatrix analyses undertaken to resolve this situation showed a different topology to that in the GBDP tree, but confirmed *Atopobium* as paraphyletic, whereas monophyly of *Olsenella* was unresolved due to the uncertain location of *O. scatoligenes* (Supplementary File 2). Consequently, it seems that the ULT rather than the GBDP topology is anomalous, a situation that offers two taxonomic ways of achieving monophyletic taxa, namely merging the genera *Atopobium* and *Olsenella* or splitting *Atopobium*. Differences in genomic G+C content and genome size (Figure 1) and oxygen requirements (Supplementary Table 1) argue against combining the two genera, as does the overall genomic divergence encompassed by the clade compared to its sister group. We thus will propose one new genus to accommodate *A. vaginae* and another new genus to accommodate *A. parvulum* and *A. rimae*. In contrast to the genus description, *A. vaginae* was described as facultatively anaerobic (Rodriguez Jovita et al., 1999). The culture condition used in that study point to microaerophilic preferences, however, while the type strain is successfully cultivated at DSMZ under anoxic conditions (DSMZ; unpublished data).

*Eggerthellaceae* (Gupta et al., 2013) encompass several genera, including the type genus *Eggerthella* (Wade et al., 1999), *Adlercreutzia* (Maruo et al., 2008), *Asaccharobacter* (Minamida et al., 2008), *Enterorhabdus* (Clavel et al., 2009), and *Parvibacter* (Clavel et al., 2013). The GBDP tree (Figure 1) shows that *Adlercreutzia, Asaccharobacter*, and *Enterorhabdus* form a clade which stands out as its genetic divergence is lower than that of adjacent clades, including closely related ones corresponding to individual genera, as exemplified by *Collinsella* and *Slackia*. In the 16S rRNA gene tree, a fourth genu*s, Parvibacter*, was assigned to this clade with *Enterorhabdus* appearing to be paraphyletic, albeit with low support. All four genera have an anaerobic lifestyle and share chemotaxonomic features, notably with respect to major fatty acids and predominant menaquinones. Indeed, *Adlercreutzia* and *Asaccharobacter*, were undistinguishable at the species level with respect to their chemotaxonomic, genomic and physiological features (Table 1, Supplementary Table 1)*;* these taxa also have similar G+C values (Figure 1). Given these similarities, it is proposed that *Asaccharobacter, Enterorhabdus* and *Parvibacter* be seen as synonyms of the earlier described *Adlercreutzia*.

**Table 1:** Outcome of applying GGDC to calculate intergenomic dDDH values.

**Strain 1**	**Strain 2**	**dDDH**	**Consequence**
*Actinomadura viridilutea* DSM 44433	*Actinomadura rubrobrunea* NBRC 15275	100.0	LHT: A. viridilutea^*^
*Adlercreutzia equolifaciens* DSM 19450	*Asaccharobacter celatus* DSM 18785	71.3	New subspecies of *A. equolifaciens* from species A. celatus^*^
*Amycolatopsis keratiniphila* subsp. *nogabecina* DSM 44586	*Amycolatopsis keratiniphila* subsp. *keratiniphila* DSM 44409	90.3	LHT: *A. keratiniphila* subsp. nogabecina^*^
*Bifidobacterium adolescentis* ATCC 15703	*Bifidobacterium stercoris* DSM 24849	80.5	LHT: *B. stercoris*
*Bifidobacterium animalis* subsp. *animalis* ATCC 25527	*Bifidobacterium animalis* subsp. *lactis* DSM 10140	65.4	Reinstate B. lactis^*^
*Bifidobacterium catenulatum* JCM 1194	*Bifidobacterium kashiwanohense* JCM 15439	72.8	New subspecies of *B. catenulatum* from species B. kashiwanohense^*^
*Bifidobacterium coryneforme* LMG 18911	*Bifidobacterium indicum* LMG 11587	85.0	LHT: B. coryneforme^*^
*Bifidobacterium gallinarum* LMG 11586	*Bifidobacterium pullorum* LMG 21816	74.5	New subspecies of *B. pullorum* from species B. gallinarum^*^
*Bifidobacterium longum* subsp. *longum* DSM 20219	*Bifidobacterium longum* subsp. *infantis* ATCC 15697	62.4	Reinstate B. infantis^*^
*Bifidobacterium longum* subsp. *longum* JCM 1217	*Bifidobacterium longum* subsp. *infantis* ATCC 15697	62.3	Reinstate B. infantis^*^
*Bifidobacterium pseudolongum* subsp. *pseudolongum* LMG 11571	*Bifidobacterium pseudolongum* subsp. *globosum* DSM 20092	52.0	Reinstate B. globosum^*^
*Bifidobacterium pullorum* LMG 21816	*Bifidobacterium saeculare* LMG 14934	75.5	New subspecies of *B. pullorum* from species B. saeculare^*^
*Bifidobacterium thermacidophilum* subsp. *thermacidophilum* DSM 15837	*Bifidobacterium thermacidophilum* subsp. *porcinum* LMG 21689	64.5	New species from subspecies^*^
*Cellulosimicrobium aquatile* 3bp	*Cellulosimicrobium funkei* NBRC 104118	86.9	LHT: C. aquatile^*^
*Cellulosimicrobium aquatile* 3bp	*Oerskovia xanthineolytica* LMG 16121	77.5	(see section Discussion)^*^
*Cellulosimicrobium funkei* NBRC 104118	*Oerskovia xanthineolytica* LMG 16121	77.6	(see section Discussion)^*^
*Clavibacter michiganensis* subsp. *capsici* PF008	*Clavibacter michiganensis* subsp. *michiganensis* VKM Ac-1403	40.0	New species from subspecies
*Clavibacter michiganensis* subsp. *michiganensis* VKM Ac-1403	*Clavibacter michiganensis* subsp. *sepedonicus* ATCC 33113	45.6	New species from subspecies
*Clavibacter michiganensis* subsp. *nebraskensis* NCPPB 2581	*Clavibacter michiganensis* subsp. *michiganensis* VKM Ac-1403	47.9	New species from subspecies
*Dietzia maris* DSM 43672	*Dietzia cinnamea* NBRC 102147	79.1	LHT: D. cinnamea^*^
*Dietzia schimae* DSM 45139	*Dietzia kunjamensis* DSM 44907	72.0	New subspecies of *D. kunjamensis* from species D. schimae^*^
*Falcivibrio vaginalis* ATCC 43063	*Mobiluncus curtisii* ATCC 35241	15.2	Non-LHT: F. vaginalis^*^
*Falcivibrio vaginalis* ATCC 43063	*Mobiluncus curtisii* subsp. *holmesii* ATCC 35242	84.6	LHT: F. vaginalis^*^
*Lentzea albidocapillata* DSM 44073	*Lentzea violacea* DSM 44796	71.0	New subspecies of *L. albidocapillata* from species L. violacea^*^
*Leucobacter musarum* subsp. *musarum* DSM 27160	*Leucobacter musarum* subsp. *japonicus* DSM 27158	62.3	New species from subspecies^*^
*Mobiluncus curtisii* ATCC 35241	*Mobiluncus curtisii* subsp. *holmesii* ATCC 35242	14.3	New species from subspecies^*^
*Mycobacterium bouchedurhonense* DSM 45439	*Mycobacterium avium* subsp. *avium* ATCC 25291	91.1	LHT: M. bouchedurhonense^*^
*Mycobacterium chimaera* DSM 44623	*Mycobacterium yongonense* 05-1390	80.8	LHT: M. yongonense^*^
*Mycobacterium intracellulare* ATCC 13950	*Mycobacterium chimaera* DSM 44623	78.8	New subspecies of *M. intracellulare* from species M. chimaera^*^
*Mycobacterium intracellulare* ATCC 13950	*Mycobacterium paraintracellulare* KCTC 29084	89.8	LHT: M. paraintracellulare^*^
*Mycobacterium intracellulare* ATCC 13950	*Mycobacterium yongonense* 05-1390	78.0	New subspecies of *M. intracellulare* from species M. yongonense^*^
*Mycobacterium vanbaalenii* PYR-1	*Mycobacterium austroafricanum* DSM 44191	88.8	LHT: M. vanbaalenii^*^
*Nocardia cummidelens* NBRC 100378	*Nocardia salmonicida* NBRC 13393	78.8	New subspecies of *N. salmonicida* from species N. cummidelens^*^
*Nocardia cummidelens* NBRC 100378	*Nocardia soli* NBRC 100376	92.9	LHT: N. soli^*^
*Nocardia salmonicida* NBRC 13393	*Nocardia soli* NBRC 100376	79.3	LHT: N. soli^*^
*Nocardiopsis baichengensis* YIM 90130	*Nocardiopsis halophila* DSM 44494	82.9	LHT: N. baichengensis^*^
*Nocardiopsis dassonvillei* subsp. *albirubida* NBRC 13392	*Nocardiopsis dassonvillei* subsp. *dassonvillei* DSM 43111	53.7	Reinstate N. alborubida^*^
*Oerskovia xanthineolytica* LMG 16121	*Cellulosimicrobium cellulans* DSM 43879	63.5	(see section Discussion)^*^
*Paraoerskovia marina* DSM 21750	*Koreibacter algae* DSM 22126	84.8	LHT: *K. algae*
*Prauserella salsuginis* DSM 45264	*Prauserella flava* DSM 45265	100.0	LHT: P. flava^*^
*Rhodococcus enclensis* DSM 45688	*Rhodococcus qingshengii* JCM 15477	88.0	LHT: R. enclensis^*^
*Rhodococcus hoagii* DSM 20295	*Rhodococcus equi* NBRC 101255	91.1	LHT: *R. hoagii*
*Saccharomonospora internatus* ATCC 33517	*Saccharomonospora viridis* DSM 43017	96.3	LHT: *S. internatus*
*Saccharomonospora paurometabolica* YIM90007	*Actinopolyspora iraqiensis* IQ-H1	73.1	New subspecies of *Saccharomonospora iraqiensis* comb. nov. from species S. paurometabolica^*^
*Saccharopolyspora jiangxiensis* CGMCC 4.3529	*Saccharopolyspora hirsuta* subsp. *kobensis* ATCC 20501	100.0	LHT: S. jiangxiensis^*^
*Saccharopolyspora jiangxiensis* CGMCC 4.3529	*Saccharopolyspora hirsuta* subsp. *kobensis* DSM 44795	98.5	LHT: S. jiangxiensis^*^
*Streptomyces aureofaciens* ATCC 10762	*Streptomyces avellaneus* NRRL B-3447	99.2	LHT: *S. avellaneus*
*Streptomyces caeruleus* NRRL B-2194	*Actinoalloteichus cyanogriseus* DSM 43889	94.9	LHT: *A. cyanogriseus*
*Streptomyces californicus* NRRL B-2098	*Streptomyces floridae* NRRL 2423	87.5	LHT: *S. floridae*
*Streptomyces fluorescens* NRRL B-2873	*Streptomyces citreofluorescens* NRRL B-3362	99.9	LHT: *S. fluorescens*
*Streptomyces griseolus* NRRL B-2925	*Streptomyces halstedii* NRRL-ISP 5068	99.9	LHT: *S. griseolus*
*Streptomyces griseorubiginosus* DSM 40469	*Streptomyces phaeopurpureus* DSM 40125	86.1	LHT: *S. phaeopurpureus*
*Tsukamurella pseudospumae* JCM 13375	*Tsukamurella sunchonensis* JCM 15929	82.3	LHT: *T. sunchonensis*
*Tsukamurella pulmonis* DSM 44142	*Tsukamurella spongiae* DSM 44990	92.8	LHT: *T. spongiae*
*Tsukamurella tyrosinosolvens* DSM 44234	*Tsukamurella carboxydivorans* JCM 15482	90.4	LHT: *T. carboxydivorans*

*Only results that yield taxonomic consequences are shown, including the genome-sequence based confirmation of heterotypic synonyms indicated in the literature. Each taxonomic consequence is indicated in addition to whether it was a new result or confirmed literature data. LHT, later heterotypic synonym; asterisk, new result (see the main text for literature references)*.

Within *Pseudonocardiaceae* (Embley et al., 1988; Stackebrandt et al., 1997; Zhi et al., 2009; Labeda et al., 2011), *Lentzea* (Yassin et al., 1995) appears to be paraphyletic in the GBDP tree (Figure 2) due to the inclusion of *Lechevalieria* (Labeda et al., 2001). The CCT does not differentiate between these taxa and shows that they are closely associated with other genera, such as *Actinorectispora* and *Actinosynnema* (Supplementary File 2). It can also be seen from Figure 2 that the genetic diversity encompassed by the *Lechevalieria* and *Lentzea* clade is comparable to that shown by adjacent clades corresponding to single genera, as exemplified by *Actinopolyspora* and *Actinosynnema. Lechevalieria* and *Lentzea* have chemotaxonomic features in common, including DL-A_2_pm in the peptidoglycan, galactose, mannose, and ribose as whole cell sugars, MK-9(H_4_) as the predominant isoprenologue and PE (diagnostic component), DPG, PG, and PI as major polar lipids (Supplementary Table 1). In contrast, *Lechevalieria* is rich in saturated and mono-unsaturated *iso-* and *anteiso-* fatty acids and *Lentzea* in saturated *iso*- and *anteiso*-components and tuberculostearic acid though it is not clear whether these characters states are apomorphies. *Lechevalieria* and *Lentzea* have genomes of a similar size with G+C values within a narrow range (Figure 2); estimates based on experimental methods were much wider: 68.0–71.4% (*Lechevalieria*) and 68.6–79.6% (*Lentzea*). In light of these genomic and phenotypic data, it is proposed that *Lechevalieria* be seen as a subjective synonym of *Lentzea*.

*Amycolatopsis* (*Pseudonocardiaceae*) does not appear to be monophyletic (Figure 2) as *A. halophila* (Tang et al., 2010a) forms a clade together with *Haloechinothrix* and *Thermocrispum* (Korn-Wendisch et al., 1995) Indeed, genera such as *Saccharomonospora* (Nonomura and Ohara, 1971b) and *Prauserella* (Kim and Goodfellow, 1999) appear to be more closely related to core *Amycolatopsis* including *A. orientalis* (Lechevalier et al., 1986), the type species, than to *A. halophila*. In addition, *Yuhushiella* (Mao et al., 2011) forms the sister group to *A. marina* (Bian et al., 2009).

Given the genomic divergence encompassed by these genera it does not make sense to classify them into a single genus, nor would this be the most conservative solution taxonomically. In the CCT (Supplementary File 2), *A. halophila* (Tang et al., 2010a) and *A. salitolerans* (Guan et al., 2012) form a highly supported clade which forms the sister group to *Haloechinothrix* with high support; this clade forms the sister group of *Thermocrispum* (Figure 2), confirmed by the ULT. The genome sizes of *A. halophila, Haloechinothrix* and *Thermocrispum* show more similarity to one another than to core *Amycolatopsis*; increased genome size appears to be an apomorphy of the latter (Figure 2). Available phenotypic features of *A. halophila* and *A. salitolerans* are in good agreement with those of *Haloechinothrix alba;* all of these species are halophilic and filamentous with similar chemotaxonomic traits (Supplementary Table 1). Moreover, support for the inclusion of A. *halophila* and *A. salitolerans* within *Amycolatopsis* was uniformly low at the backbone of the respective 16S rRNA gene trees nor were these taxa compared with *H. alba* which was described around the same time. Given all the these results, it is proposed that *A. halophila* and *A. salitolerans* be transferred to the genus *Haloechinothrix*. The original description of *A. halophila* had made it necessary to emend the description of *Amycolatopsis*; these changes can now be reversed. Again, the original descriptions of *A. halophila* and *A. salitolerans* had not considered *Haloechinothrix*, and support was uniformly low at the backbone of the 16S rRNA gene trees.

In the original description of *Yuhushiella* (Mao et al., 2011) it was compared with a restricted number of *Amycolatopsis* species which did not include *A. marina*, while support for the backbone of the 16S rRNA gene tree was uniformly low. *Yuhushiella* has many features in common with *Amycolatopsis*, notably with respect to fatty-acid and polar-lipid profiles (Supplementary Table 1) and genomic G+C content (Figure 2). Consequently, it is proposed that *Yuhushiella* be regarded as a subjective synonym of *Amycolatopsis*. This solution is safer and more conservative taxonomically than further splitting *Amycolatopsis*, which is difficult because the genus is large and despite the strong resolution of the phylogenomic tree there were hardly any well-supported subgroups in the CCT (Supplementary File 2).

*Actinokineospora* (Hasegawa, 1988; Labeda et al., 2010; Tang et al., 2012) appears to be paraphyletic in the phylogenomic tree (Figure 2) given the position of *Alloactinosynnema iranicum* (Nikou et al., 2014). The ULT confirmed the paraphyly of *Actinokineospora*, albeit with moderate support (Supplementary File 2), while the CCT shows that the type species of *Alloactinosynnema, A. album* (Yuan et al., 2010), is the sister group to *A. iranicum*. The relationship between these species was not clear either in the UCT or in the original description of *A. album*. The chemotaxonomic characteristics of *A. iranicum* are more in line with those of *Actinokineospora*, as it contains arabinose, galactose and ribose as whole-organism sugars and PE (diagnostic component) in its polar-lipid profile whereas *A. album* is rich in PC and contains only arabinose as whole-organism sugar (Nikou et al., 2014). However, the value of these chemotaxonomic features in separating these genera is doubtful as the character states of *A. album* are most likely to be its autapomorphies; this means that the other states are plesiomorphic and hence cannot be used to separate a taxon. The CCT also shows that *A. album* falls within *Actinokineospora*. Consequently, it is proposed that *Alloactinosynnema* be seen as a subjective synonym of *Actinokineospora*.

*Corynebacterium* (Lehmann and Neumann, 1896; Bernard et al., 2010) appeared paraphyletic due to the position of *Turicella otitidis* (Funke et al., 1994a). It also appears from the CCT, UCT, and ULT (Supplementary File 2) that *Corynebacterium* is paraphyletic given the position of *Turicella otitidis*, a species found to be closely related to *C. atypicum* (Hall et al., 2003a) and *C. frankenforstense* (Wiertz et al., 2013). Both genera have DL-A_2_pm in the peptidoglycan and arabinose and galactose as whole-cell sugars (Supplementary Table 1), but can be distinguished based on menaquinone and fatty-acid composition. *Corynebacterium* has either MK-8(H_2_) or MK-9(H_2_) as predominant menaquinone whereas *T. otitidis* contains major amounts of MK-10 or MK-11 (Busse, 2012). Further, *Corynebacterium* mostly has short-chain mycolic acids and tuberculostearic acid whereas *T. otitidis* lacks these compounds, as do some species of *Corynebacterium*, as exemplified by *C. atypicum, C. caspium* (Collins et al., 2004a), and *C. lactis* (Wiertz et al., 2013). In turn, *C. frankenforstense* synthesizes mycolic acids but not tuberculostearic acid while the reverse is the case in *C. ciconiae* (Fernández-Garyzábal et al., 2004) and *C. kroppenstedtii* (Collins et al., 1998).

A recent study (Baek et al., 2018) revealed that genes that play an essential role in mycolic acid biosynthesis are absent in *Turicella* and other mycolate-less *Corynebacterium* species and proposed that *T. otitidis* be classified in the genus *Corynebacterium*. Moreover, menaquinone reductase was also found to be absent in *Turicella*. While these authors concluded from their results that chemotaxonomic features such as menaquinones and mycolic acids should be treated with caution when drawing taxonomic conclusions, they may not have taken into account the consequences of the fact that the presence of mycolic acids in *Corynebacteriales* (Goodfellow and Jones, 2012) is a synapomorphy of the taxa included in the order. It follows that this character state is plesiomorphic *within* the order and hence should not have been used to justify the separation of *Corynebacterium* and *Turicella* in the first place. The same holds for menaquinones, as the widespread distribution of MK-8(H_2_) and MK-9(H_2_) in *Corynebacteriales* (Supplementary Table 1) indicates that their presence is plesiomorphic within the order and in *Corynebacterium*, whereas their replacement by MK-10/11 is an autapomorphy of *Turicella*. If the separation of the two genera was incorrectly based on these characters, then one cannot conclude from the need to include *Turicella* in *Corynebacterium* that these characters are not trustworthy *per se*.

It appears from the GBDP tree (**Figure 8**) that *Kocuria* (Stackebrandt et al., 1995) is paraphyletic with respect to *Rothia* (Georg and Brown, 1967) as *K. kristinae* (Kloos et al., 1974) is more closely related to *Rothia* than to the remaining *Kocuria* species (**Figure 8**), an arrangement that carries maximum support in the ULT (Supplementary File 2) and is underpinned in the CCT. It is also apparent from the CCT that *K. rosea* (Flügge, 1886; Stackebrandt et al., 1995), the type species of the genus, belongs to a clade in the phylogenomic tree (**Figure 8**) that is composed of *K. flava, K. polaris*, and *K. turfanensis*, whereas *K. halotolerans, K. koreensis*, and *K. kristinae* form a clade that appears as the sister group to *Rothia*. There do not appear to be any pronounced phenotypic differences between the three *Kocuria* species and *Rothia* (Supplementary Table 1). Indeed, *Kocuria* also appears to be paraphyletic in a previously published 16S rRNA gene tree, albeit without much support (Collins et al., 2000b). In light of these observations, it is proposed that *K. halotolerans, K. koreensis*, and *K. kristinae* be classified in *Rothia*.

*Streptomycetaceae* (Waksman and Henrici, 1943; Stackebrandt et al., 1997; Kim et al., 2003; Zhi et al., 2009; Huang et al., 2017) contain four genera, namely *Streptomyces* (Waksman and Henrici, 1943; Witt and Stackebrandt, 1990; Wellington et al., 1992), the type genus, *Allostreptomyces* (Huang et al., 2017), *Kitasatospora* (Omura et al., 1982; Zhang et al., 1997), and *Streptacidiphilus* (Kim et al., 2003) though the status of *Kitasatospora* and *Streptacidiphilus* have been questioned (Kämpfer, 2012a). In the phylogenomic tree (**Figure 6**) the monophyly of *Kitasatospora* and *Streptomyces* is in conflict with the inclusion of *S. aureofaciens* (Duggar, 1948), *S. avellaneus* (Baldacci and Grein, 1966), and *S. purpeofuscus* (Yamaguchi and Saburi, 1955) within *Kitasatospora* and the insertion of *Kitasatospora* and *Streptacidiphilus* within *Streptomyces*, both with maximum support (**Figure 6**). In the 16S rRNA gene tree, the aforementioned *Streptomyces* species form a well-supported clade with *Kitasatospora* and *S. aburaviensis* (Nishimura et al., 1957), *S. herbaricolor* (Kawato and Shinobu, 1959), *S. indigoferus* (Shinobu and Kawato, 1960), *S. xanthocidicus* (Asahi et al., 1966), *S. purpureus* (Matsumae et al., 1968; Goodfellow et al., 1986c), *S. chrysomallus* (Lindenbein, 1952), *S. psammoticus* (Virgilio and Hengeller, 1960), and *S. alboverticillatus* (Witt and Stackebrandt, 1990) (Supplementary File 2); the ULT shows this group though the situation is not well-resolved. These results confirm those reported previously (Labeda et al., 2017) in a multi-locus sequence analysis of *Streptomycetaceae*, but additionally clarify the status of *S. indigoferus* and *S. xanthocidicus*. Consequently, it is proposed that these species be transferred to the genus *Kitasatospora*. However, the taxonomic status of *S. alboverticillatus* needs to be checked as the 16S rRNA gene sequence of this organism (AY999766), derived from strain JCM 5010^T^, was found within *Kitasatospora* in the CCT whereas *S. alboverticillatus* NRRL B 24281^T^ was previously placed within *Streptomyces* (Labeda et al., 2017).

The assignment of *Kitasatospora* species, including *K. cystarginea* (Kusakabe and Isono, 1988), *K. griseola* (Takahashi et al., 1984), *K. mediocidica* (Labeda, 1988), *K. phosalacinea* (Takahashi et al., 1984), and the type species *K. setae* (Omura et al., 1982) to *Streptomyces* (Wellington et al., 1992) was based on the realization that these groups share high 16 rRNA gene sequence similarities and key chemotaxonomic and morphological properties (Supplementary Table 1). However, *Kitasatospora* and *Streptacidiphilus* were considered to differ from *Streptomyces* as their whole-organism hydrolysates contain DL- and LL-A_2_pm and galactose though these features were not available for most *Streptomyces* species, including those mentioned above. However, even if *Kitasatospora* and *Streptomyces* were merged, the resulting genus would still be paraphyletic as *Streptacidiphilus* forms the sister group to the *Kitasatospora* clade with high support (**Figure 6**). The results of the present analyses are in line with those reported previously (Labeda et al., 2017), as they support the distinctiveness of *Kitasatospora* and *Streptacidiphilus* as the considerable genetic divergence of the entire group also indicates the three genera should remain distinct*;* an earlier case was made for the separation of *Kitasatospora* from *Streptomyces* (Zhang et al., 1997).

It is evident from the GBDP tree (**Figure 6**) that *Streptomyces scabrisporus* (Ping et al., 2004) branches from core *Streptomyces* before *Kitasatospora* and *Streptacidiphilus* (Kim et al., 2003). *S. scabrisporus* forms the sister group to the core *Streptomyces-Kitasatospora-Streptacidiphilus* clade in the phylogenomic tree, and also in the CCT with high support and the ULT (Supplementary File 2). In addition, the organism can be distinguished from *Kitasatospora, Streptacidiphilus*, and core *Streptomyces* by a number of phenotypic features (Supplementary Table 1). Consequently, it is proposed that S. *scabrisporus* be classified as a genus in its own right.

*S. thermoautotrophicus* (Gadkari et al., 1990) branched even earlier than *S. scabrisporus* in the phylogenomic tree (**Figure 6**); its isolated position is underpinned in the CCT and ULT analyses with high support in the former (Supplementary File 2). This species shares chemotaxonomic and morphological features with core *Streptomyces* (Supplementary Table 1) but can be distinguished from the latter and from *Kitasatospora* and *Streptacidiphilus* given its obligate thermophilic nature, its inability grow below 40°C and its chemolithotrophic lifestyle; it is able to use CO_2_ or H_2_ plus CO_2._. Indeed, this species is unique in its exclusive use of lithotrophic substrates. Given these observations, it is proposed that *S. thermoautotrophicus* merits generic status. An earlier multi-gene phylogeny (Mackellar et al., 2016) yielded the same outcome. However, the type strain of the species is currently available from a single culture collection only (DSM 100163), hence a new genus with *S. thermoautotrophicus* as type species cannot be proposed (Parker et al., 2015).

A highly supported clade composed of *S. aomiensis* (Nagai et al., 2011), *S. catbensis* (Sakiyama et al., 2014), and *S. seranimatus* (Wang et al., 2007c) was found to branch before *S. scabrisporus* but after *S. thermoautotrophicus* in the CCT. The three species share biochemical, cultural, and morphological features, notably the formation of smooth spores in straight to flexuous or looped spore chains, properties that distinguished them from their nearest phylogenetic neighbor, *S. scabrisporus*, which produces rugose ornamented spores in spiral chains (Ping et al., 2004; Wang et al., 2007c; Sakiyama et al., 2014). In turn, S. *seranimatus* can be distinguished from all other *Streptomycetaceae* given the presence of PC in its polar lipid profile (Supplementary Table 1). Given this information, it is proposed that *S. aomiensis, S. catbensis*, and *S. seranimatus* be assigned to a new genus. This is immediately feasible for *S. aomiensis* only because the type strains of the other two species are currently available from only a single culture collection (Parker et al., 2015).

The proposals for the recognition of four new genera leaves *Streptomyces* as a monophyletic taxon. Indeed, these developments provide a more taxonomically conservative way of addressing the paraphyly of *Streptomyces* than lumping *Kitasatospora, Streptacidiphilus*, and *Streptomyces sensu lato* into a single genomically diverse taxon. Moreover, the set of phenotypic character states common to the early branching *Streptomyces* and core *Streptomyces*, given their phylogenetic distribution, is probably plesiomorphic within *Streptomycetaceae* and hence do not justify retaining *Streptomyces* in its present form.

*Actinomycetaceae* (Buchanan, 1918; Stackebrandt et al., 1997; Zhi et al., 2009) encompass genera such as *Actinomyces* (Harz, 1877), the type genus, *Actinobaculum* (Lawson et al., 1997; Yassin et al., 2015), *Actinotignum* (Yassin et al., 2015), *Arcanobacterium* (Collins et al., 1982; Lehnen et al., 2006), *Mobiluncus* (Spiegel and Roberts, 1984), *Trueperella* (Yassin et al., 2011), and *Varibaculum* (Hall et al., 2003d). In the phylogenomic tree, the genomically diverse *Actinomyces* appears to be paraphyletic as it encompasses *Mobiluncus* and *Varibaculum* (**Figure 7**); the ULT shows a similar arrangement, but with lower support. The phylogenomic tree does not include *Actinomyces bovis* (Harz, 1877), the type species of the genus, as a genomic sequence of its type strain was not available at the time of analyzing the data. It is apparent from the CCT (Supplementary File 2) that *A. bovis* falls within the clade that encompasses most of the *Actinomyces* species; this clade in the phylogenomic tree ranges from *A. slackii* to *A. massiliensis* (**Figure 7**). This core *Actinomyces* clade has larger genomes and a higher genomic G+C content than is found in other *Actinomyces* species, apart for *A. hordeovulneris* and *A. nasicola*. The CCT also shows that the balance of *Actinomyces* species can be assigned to an additional seven distinct, maximally supported or monotypic clades (Supplementary File 2). *A. neuii* formed the sister group to *Varibaculum cambriense*, the type species of the genus, and *V. anthropi* with moderate support; the ULT showed strong support for the relationship between *A. neui* and *V. cambriense; V. anthropi* was not included in this analysis. The sister-group relationship between this latter group and *Mobiluncus*, including *M. curtisii* (Spiegel and Roberts, 1984), the type species of the genus, and its heterotypic synonym *Falcivibrio vaginalis* (Hammann et al., 1984) was unambiguous in the GBDP tree (**Figure 7**). The remaining six *Actinomyces* clades occupied isolated positions in the CCT (Supplementary File 2). The overall proteomic differences between several pairs of *Actinomyces* species, e.g., *A. hordeovulneris* and *A. nasicola*, are greater than those between *Arcanobacterium* and *Trueperella* and between *Actinobaculum* and *Actinotignum*. These results illustrate the inconsistencies of the current classification of *Actinomycetaceae* in quantitative terms.

In general, the results outlined above are in good agreement with those of previous studies which showed *Actinomyces* to be a heterogeneous taxon containing distinct subgroups (Schofield and Schaal, 1981; Schaal and Schofield, 1984; Schaal and Gatzer, 1985; Lawson et al., 1997; Ramos et al., 1997; Schaal et al., 2006) though two main groups were recognized based on 16S rRNA gene signature nucleotides (Schaal et al., 2006). However, in a recent phylogenomic analysis (Zhao et al., 2014) *Actinomyces* species were recovered in major groups A, B, and C, which correspond to three of the clades in the CCT (Supplementary File 2). Indeed, the taxonomic status of some of the clades defined in the CCT are supported by the discontinuous distribution of chemotaxonomic traits (Supplementary Table 1). Thus, the clade represented by *A. meyeri* to *A. radingae* in **Figure 7** contains mannose and rhamnose as whole organism sugars, MK-9(H_4_) as the predominant menaquinone and DPG and PG as major polar lipids whereas core *Actinomyces* species are characterized by the presence of 6 deoxytalose, fucose, galactose, and glucose in whole organism hydrolysates, MK-10 as the predominant manaquinone and a polar lipid pattern that features PC. In turn, *A. neuii* subsp. *anitratus* (Funke et al., 1994b), *A. neuii* subsp. *neuii* (Funke et al., 1994b), and *Varibaculum cambriense* (Hall et al., 2003d), the type species of the genus, have an A5α (L-lysine-L-lysine-D-glutmic acid) peptidoglycan and a fatty-acid profile with major proportions of oleic, palmitic and stearic acids. In contrast, little chemotaxonomic data are currently available for several *Actinomyces* species, notably *A. hongkongensis, A. marimammalium*, and *A. nasicola*. The lack of chemotaxonomic features specific to the other clades might simply be due to their combination of features representing the plesiomorphic states. Given the phylogenomic and associated data outlined here, we propose to split *Actinomyces* according to the well-supported or monotypic, genomically well-differentiated clades mentioned above, yielding seven novel genera.

*Leifsonia* (Evtushenko et al., 2000a) appears to be polyphyletic in the GBDP tree (Figure 1) as *L. rubra* (Reddy et al., 2003) is placed distantly from *L. xyli* subsp. *cynodontis* (Evtushenko et al., 2000a), as is the case with the ULT and the CCT. It is also apparent, from the CCT that *L. aquatica* (Evtushenko et al., 2000a), the type species of *Leifsonia*, forms a clade with *L. xyli* subsp. *cynodontis* while a well supported second clade includes *L. rubra* and the type species of *Rhodoglobus, R. vestalii* (Sheridan et al., 2003). The 16S rRNA gene similarity between the *L. rubra* sequence AJ438585 and the *R. vestalii* sequence AJ459101 was 99.44% when calculated using the recommended settings (Meier-Kolthoff et al., 2013b), indicating that DNA:DNA relatedness must determine whether they should or should not remain as separate species. Because genome sequence data for *R. vestalii* were not available at the time of writing, we here refrain from considering the taxonomic consequences for *L. rubra*. Traditional DDH values between *L. rubra* DSM 21193 and *R. vestalii* DSM 21947 were determined as 73.8–75.0% (DSMZ, unpublished data) but while DSM 21193 is a replacement of the incorrect type-strain deposit DSM 15304 it is not included in the DSMZ online catalog because it was not clear whether it shows the properties indicated in the species description.

The mycolic-acid containing genera *Nocardia* (Trevisan, 1889), *Skermania* (Chun et al., 1997a), and *Smaragdicoccus* (Adachi et al., 2007) formed a maximally supported clade in the GBDP tree (Figure 3), which also included *Rhodococcus kunmingensis* (Wang et al., 2008). *R. kunmingensis* was also set apart from core *Rhodococcus* (Zopf, 1891) in the CCT and ULT (Supplementary File 2). However, in both the UCT and original publication *R. kunmingensis* was found to be closely related to *R. equi* (Goodfellow and Alderson, 1977), which is now included in *R. hoagii* (Kämpfer et al., 2014a); in the original 16S rRNA gene tree they formed a clade supported by a 92% bootstrap value using the neighbor-joining algorithm and the Kimura-2-parameter model whereas in the corresponding ML and MP analyses there was only low support for this association. Additional supermatrix analyses confirmed the distant position of *R. kunmingensis* relative to core *Rhodococcus*, particularly with the ML criterion (Supplementary File 2). The relatively isolated position of *R. kunmingensis* was noted in an extensive phylogenomic analysis of *Rhodococcus* (Sangal et al., 2016). The genome sequence of *R. kunmingensis* in the present study was incomplete but did not show any sign of contamination; the 5S, 16S, and 23S rRNA genes were located on the same contig (data not shown). Consequently, confidence can be placed in its position in the phylogenomic tree. There is only low support for the sister-group relationship between *R. kunmingensis* and *Skermania piniformis* in the GBDP tree (Figure 3) which also shows them separated by long branches. *S. piniformis* and *R. kunmingensis* contain DL-A_2_pm, arabinose, and galactose in the peptidoglycan and have DPG and PE as major polar lipids, but can be distinguished by marked differences in fatty acid, menaquinone, mycolic acid composition, and in genomic G+C content (Figure 3, Supplementary Table 1). In short, the various datasets show that *R. kunmingensis* merits recognition as a new genus within *Nocardiaceae*.

*Gordonia* appears to be paraphyletic in the GBDP tree (Figure 3) as *G. kroppenstedtii* (Kim et al., 2009) forms the sister group of *Williamsia* (Kämpfer et al., 1999) though support for this is low in both the GBDP tree and the CCT. An additional supermatrix analysis using *Tsukamurella* as outgroup shows, with strong support, that *G. kroppenstedtii* branches below the base of the core *Gordonia* and *Williamsia* clade thereby rendering *Gordonia* paraphyletic (Supplementary File 2). In the original description, *G. kroppenstedtii* appeared as the sister group of the other *Gordonia* species though bootstrap support was low and not all *Williamsia* species were represented while in a recent 16S rRNA gene analysis *G. kroppenstedtii* was recovered outside core *Gordonia* (Tsang et al., 2016). *Gordonia* and *Williamsia* have very similar phenotypic profiles (Supplementary Table 1) and there is no evidence that character states should be regarded as apomorphies of one or other of these taxa. The polar lipid profile, for instance, of *Gordonia* matches that of *Tsukamurellaceae* hence according to these features *Gordonia* may well be paraphyletic, as found in this study. Our proposal to assign *G. kroppenstedtii* to a new genus does not conflict with the phenotypic data and is taxonomically more conservative than merging *Gordonia* and *Williamsia*, a move that would run counter to their overall genetic divergence (Figure 3).

It has recently been suggested to split *Mycobacterium* into five distinct genera (Gupta et al., 2018). That study deserves credit for identifying molecular synapomorphies for each of these five clades, but since such synapomorphies were also identified for a monophyletic *Mycobacterium* as currently circumscribed, the decision to split the genus may be regarded as somehow arbitrary. In our tree (Figure 3, Supplementary File 2) *Mycobacterium* also appears to be monophyletic and not as the genomically most divergent genus within *Corynebacteriales* (Figure 3, Supplementary File 2). It remains to be seen which of the two concepts for *Mycobacterium* will be adopted by future taxonomic studies.

*Micromonospora* (Ørskov, 1923), the type genus of *Micromonosporaceae* (Krasil'nikov, 1938; Zhi et al., 2009), appears to be paraphyletic in the GBDP tree (Figure 4) as the clade composed of *Verrucosispora* and *Xiangella* appears to be the sister group to *M. nigra* (Weinstein et al., 1968; Kasai et al., 2000), while *Verrucosispora* (Rheims et al., 1998; Xi et al., 2012) appears to be paraphyletic given the position of *Xiangella phaseoli* (Wang et al., 2013a), the type species of the genus. Further*, Salinispora* (Maldonado et al., 2005) would seen to be more closely related to core *Micromonospora* than to *M. pattaloongensis* (Thawai et al., 2008). The ULT is in agreement with these relationships, albeit with lower support. Although the genome sequence of *M. chalcea* (Ørskov, 1923), the type species of the genus, was not available at the time of running the phylogenomic analysis, this organism was found in the CCT, with high support, in the *M. aurantiaca-marina-tulbaghiae* clade (Supplementary File 2). It is difficult to distinguish between the genera *Micromonospora, Salinispora, Verrucosispora*, and *Xiangella* as they have many phenotypic features in common (Supplementary Table 1), notably chemotaxonomic and morphological properties, as exemplified by their ability to form single non-motile spores on branched substrate mycelia and the typical absence of aerial hyphae (Supplementary Table 1). The original description of *Verrucosispora* was based on an ability to synthesize 10-methyl C_17:0_ and the absence of arabinose in whole-cell hydrolysates, but *M. citrea* (Kroppenstedt et al., 2005) and *M. echinaurantiaca* (Kroppenstedt et al., 2005) exhibit these features while *M. endolithica* (Hirsch et al., 2004), *M. inositola* (Kawamoto et al., 1974), and *M. viridifaciens* (Kroppenstedt et al., 2005) contain moderate proportions of 10-methyl C_17:0_ (Kroppenstedt et al., 2005) and *M. cremea* (Carro et al., 2012) and *M. mirobrigensis* (Trujillo et al., 2005) do not have arabinose in the cell wall. One of the key arguments for proposing the genus *Xiangella* was the presence of PC in the polar-lipid profile of *X. phaseoli*, however, the original polar lipid profile is not fully conclusive in this respect (Wang et al., 2013a) (Supplementary Table 1). In addition, single characters with two states are, even in the absence of homoplasies, insufficient to separate two taxa, as only one of the states can be apomorphic. It can be concluded from what is said above that there is insufficient evidence from both the phenotype and the single-gene phylogenies to separate *Verrucosispora* and *Xiangella* from *Micromonospora*, an outcome supported by 16S rRNA gene sequence evidence (Supplementary File 2). Consequently, it is proposed that *Verrucosispora* and *Xiangella* be included in *Micromonospora*.

Whereas an earlier study (Girard et al., 2013) suggested that *Micromonospora, Salinispora*, and *Verrucosispora* might represent different clades in the same genus based on an analysis of conserved protein sequences, *Micromonospora* and *Salinispora* were quite sharply separated, albeit closely related, in a genome-based phylogeny of *Micromonosporaceae* (Carro et al., 2018), although *M. pattaloongensis* was not considered in this study. The GBDP tree suggests that this species should better be removed from *Micromonospora* (Figure 4) though, the UCT and CCT were not clear on this point (Supplementary File 2) hence additional *Micromonosporaceae* genome sequences need to be examined to determine a more precise relationship between these taxa.

*Actinoplanes* (Couch, 1950) appears to be paraphyletic in the GBDP tree (Figure 4) as *Pseudosporangium ferrugineum* (Ara et al., 2008b) forms the sister group to *A. friuliensis* (Wink et al., 2014), a relationship underpinned in the UCT, ULT, and CCT (Supplementary File 2), but with low support. In general, *P. ferrugineum* has many phenotypic features in common with *Actinoplanes* (Supplementary Table 1); it can be distinguished from the latter based on menaquinone and whole-cell sugar composition but it remains unclear whether these character states yield apomorphies for both groups. Moreover, menaquinone composition can vary according to age and culture conditions (Saddler et al., 1986). Genome size and G+C content are consistent within the *Actinoplanes*-*Pseudosporangium* clade. Consequently, it is proposed that *P. ferrugineum* be transferred to *Actinoplanes*. The CCT indicated that *Couchioplanes* (Tamura et al., 1994) and *Krasilnikovia* (Ara and Kudo, 2007) form the sister group to *Pseudosporangium* but with low support only, which suggests that they may also be seen as synonyms of *Actinoplanes* though further comparative taxonomic studies are needed to address this point.

*Microlunatus* (Nakamura et al., 1995) and *Friedmanniella* (Schumann et al., 1997) belong to *Propionibacteriaceae* (Delwiche, 1957). In the original description of *Friedmanniella, F. antarctica*, the type species (Schumann et al., 1997), shared chemotaxonomic properties with its counterpart in the genus *Microlunatus, M. phosphovorus* (Nakamura et al., 1995), but was distinguished from the latter based on growth and phenotypic properties though it was not clear which character states were apomorphic. Moreover, several of the differential properties shown by *F. antarctica* were found to be variable when additional species were added to *Friedmanniella* (Iwai et al., 2010). It is evident from the GBDP tree (Figure 5) that *Microlunatus* is paraphyletic as it includes *Friedmanniella*; it is also clear that the genetic diversity shown within the clade is low. Although a genomic sequence is not available for *F. antarctica* it is evident from the CCT (Supplementary File 2) that it forms a well-supported clade with *F. flava* and *F. sagamiharensis*. Consequently, it is proposed that *Friedmanniella* be seen as a subjective synonym of *Microlunatus*.

*Propionibacterium* (Orla-Jensen, 1909; Charfreitag et al., 1988) of *Propionibacteriaceae* (Delwiche, 1957; Zhi et al., 2009) was recently split by generating three novel genera, *Acidipropionibacterium, Cutibacterium*, and *Pseudopropionibacterium*, based on the analysis of genomic data (Scholz and Kilian, 2016). In the present study, *Propionibacterium namnetense* (Aubin et al., 2016), which was described at around the same time as the revision of *Propionibacterium*, was found to form the sister group to *C. acnes* in the phylogenomic tree (Figure 5), a position in line with phenotypic data (Supplementary Table 1). Consequently, it is proposed that *P. namnetense* be classified in *Cutibacterium. P. acnes* subsp. *defendens* (McDowell et al., 2016) also needs to be transferred to *Cutibacterium*.

*Thermomonospora* (Henssen, 1957; Zhang et al., 1998), the type genus of *Thermomonosporaceae* (Stackebrandt et al., 1997; Zhang et al., 2001; Zhi et al., 2009), appears to be paraphyletic in the GBDP tree (Figure 6) as *T. curvata* (Henssen, 1957), the type species, is phylogenetically located in the *Actinomadura-Spirillospora* clade. In turn, *T. chromogena* (Krasil'nikov and Agre, 1965; McCarthy and Cross, 1984) is phylogenetically placed within *Streptosporangiaceae*. While the problematic positioning of *T. chromogena* was recently fixed by taxonomically assigning the species to the genus *Thermostaphylospora* (Wu et al., 2018), the classification of *Thermomonospora* remained unsatisfactory.

Indeed, *Actinomadura* (Lechevalier and Lechevalier, 1970) also appears to be paraphyletic in the phylogenomic tree (Figure 6) as *T. curvata* formed a well-supported clade with *A. echinospora* (Nonomura and Ohara, 1971b; Kroppenstedt et al., 1990). *A. amylolytica* (Jiao et al., 2015), *A. cellulosilytica* (Jiao et al., 2015), and *A. umbrina* (Galatenko et al., 1981) were also placed in this clade in the CCT (Supplementary File 2). An earlier study based on 16S and 23S rRNA gene sequences showed that *A. echinospora* and *T. curvata* were assigned to a clade that was clearly separate from other *Actinomadura* species (Trujillo and Goodfellow, 2012). Much like *T. curvata* the four *Actinomadura* species are thermophilic and also have chemotaxonomic and other phenotypic features in common; their genomic G+C content is also similar (Supplementary Table 1). Taking all of these data into account it is proposed that the four species be classified in an amended genus *Thermomonospora*.

Despite the changes mentioned above *Actinomadura* still appears to be paraphyletic in the GBDP tree (Figure 6) given the strong support for the inclusion of *Spirillospora albida*, the type species of the genus (Couch, 1963), among the *Actinomadura* species, including the type species, *A. madurae* (Vicent, 1894; Kroppenstedt et al., 1990). This situation is compounded by the uncertain position of some *Actinomadura* species, notably *A. alba* (Wang et al., 2007b) and *A. scrupuli* (Lee and Lee, 2010a) in the CCT (Supplementary File 2). This situation is complicated even further as in the CCT *Actinocorallia* (Iinuma et al., 1994) is found within the *Actinomadura* clade. Comparative studies based on additional genome sequences are needed before the taxonomic issues touched upon here can be addressed though it should be noted that the name *Spirillospora* has priority over *Actinomadura*.

The type genus *Nocardiopsis* (Brocq-Rousseau, 1904; Meyer, 1976) appears paraphyletic because two other *Nocardiopsaceae* genera (Rainey et al., 1996), *Allosalinactinospora* (Guo et al., 2015), and *Streptomonospora* (Cui et al., 2001; Li et al., 2003d), are phylogenetically located within *Nocardiopsis* (Figure 6). This causes core *Nocardiopsis*, including the type species *N. dassonvillei* (Brocq-Rousseau, 1904; Meyer, 1976), to be located separately from a clade containing *N. halophila, N. baichengensis, N. chromatogenes*, and *N. potens*, a second clade consisting of *N. gilva* and a third one containing *N. trehalosi*. Except for core *Nocardiopsis* at the backbone of the *Nocardiopsaceae* subtree support was low in the CCT and UCT. This situation is complicated even further as additional genera appear to be intermixed with the deviating *Nocardiopsis* species, they include *Haloactinospora* (Tang et al., 2008), *Lipingzhangella* (Zhang et al., 2016a), and *Salinactinospora* (Chang et al., 2012). The complex taxonomic relationships outlined above need to be tackled but it is not practical to do so until additional *Nocardiopsaceae* genome sequences become available.

The type genus of *Sanguibacteraceae* (Stackebrandt and Schumann, 2000; Zhi et al., 2009) is *Sanguibacter* (Fernández-Garayzábal et al., 1995; Pikuta et al., 2017). This genus appears paraphyletic in the phylogenomic tree (Figure 7) as *Jonesia* (Rocourt et al., 1987) and *Timonella* (Mishra et al., 2013) were found, with maximal support, to be the sister group to a clade composed of the majority of *Sanguibacter* species while *S. marinus* (Huang et al., 2005; Pikuta et al., 2017) and *S. soli* (Kim et al., 2008; Pikuta et al., 2017) formed a distinct clade. A similar arrangement could be observed in the CCT (Supplementary File 2). In the UCT and the ULT maximum-likelihood tree the monophyly of *Sanguibacter* was unresolved, much like in the published 16S rRNA gene trees (Kim et al., 2008). In contrast, in the ULT maximum-parsimony analysis *Sanguibacter* was found to be monophyletic, but additional supermatrix analyses underlined the topology found in the GBDP tree (Supplementary File 2). The fact that *Jonesia* and *Timonella* have lower genomic G+C contents than *Sanguibacter* (Figure 7) argues against merging these taxa into a single genus, whereas S. *marinus* and *S. soli* differ in genome size from the other *Sanguibacter* species. There is also evidence that other genera, such as *Rarobacter* (Yamamoto et al., 1988), might be more closely related to some of the groups in the *Sanguibacter-Jonesia*-*Timonella* clade so the monophyly or otherwise of this assemblage cannot be established at present. Hence it is proposed that *S. marinus* and *S. soli* be assigned to a new genus.

The position of *Demequina salsinemoris* (Matsumoto et al., 2010) in the phylogenomic tree makes *Demequina* (Yi et al., 2007; Ue et al., 2011) paraphyletic due to the inclusion of *Lysinimicrobium* (Hamada et al., 2012, 2015); this relationship was underscored in the ULT albeit with low support (Supplementary File 2). *Demequina* and *Lysinimicrobium* have similar chemotaxonomic profiles, notably the presence of an unusual isoprenoid quinone, demethylmenaquinone [DMK(H_4_)], but differ in G+C content and oxygen preference (Supplementary Table 1). However, the genomic G+C content of *D. salsinemoris* is near to those of *Lysinimicrobium* species (Fig. 7) while *D. lutea* (Ue et al., 2011) is facultatively anaerobic like *Lysinimicrobium; Demequina* has L-lysine as the diamino acid in the peptidoglycan whereas *Lysinimicrobium* mostly has L-ornithine, though *D. globuliformis* contains both L-lysine and L-ornithine (Ue et al., 2011). Moreover, it has already been pointed out that single characters with two characters states do not provide sufficient evidence for separating two taxa according to the principles of phylogenetic systematics. Given all of this information it is proposed that *Lysinimicrobium* be considered as a subjective synonym of the earlier described taxon *Demequina*.

The *Intrasporangiaceae* genera *Phycicoccus* (Lee, 2006c; Zhang et al., 2011) and *Tetrasphaera* (Maszenan et al., 2000; Ishikawa and Yokota, 2006) are non-monophyletic in the GBDP tree (Figure 8) as *P. jejuensis*, the type species of *Phycicoccus*, is not only placed far from *P. cremeus* and *P. dokdonensis* but also forms a well-supported clade with *T. duodecadis*. In turn, *T. japonica*, the type species of *Tetrasphaera* (Maszenan et al., 2000) was separated from at least three of the other *Tetrasphaera* species. These relationships were also found in the CCT in which *P. jejuensis* formed a clade with *P. endophyticus* (Liu et al., 2016). The positioning of *P. jejuensis* differs significantly between the published 16S rRNA gene trees (Azman et al., 2016), which show a monophyletic *Phycicoccus* with strong support, and the GBDP tree. Additional supermatrix analyses clearly confirmed the sister-group relationship between *P. jejuensis* and *T. duodecadis* as well as between *T. elongata* and this clade. The ULT showed the same topology but with moderate support only. The genome sequence of *P. jejuensis* used was not complete but did not show any sign of contamination; moreover, the 5S, 16S, and 23S rRNA genes were located on the same contig (data not shown). It can be concluded, therefore, that *Tetrasphaera* is intermixed not only with *Phycicoccus* due to the position of *T. duodecadis* but also with *Knoellia* (Groth et al., 2002) due to the position of *T. remsis*. Although *Knoellia, Phycicoccus*, and *Tetrasphaera* display quite similar chemotaxonomic and morphological characteristics (Supplementary Table 1) it is too early to consider merging them into a single taxon, partly because the monophyly of the entire group remains unresolved in the GBDP tree but also because it is evident from the CCT that further *Intrasporangiaceae* genera, such as *Lapillicoccus* (Lee and Lee, 2007) and *Oryzihumus* (Kageyama et al., 2005), might also be intermixed. Despite these reservations it is timely to clarify some of the relationships found between and within *Knoellia, Phycicoccus*, and *Tetrasphaera* based on phylogenetic and associated phenotypic data in order to achieve better supported taxa. Toward this end, it is proposed that *P. cremeus* and *P. dokdonensis* as well as the other *Phycicoccus* species that unambiguously formed a clade in the CCT together with these two species (Supplementary File 2) be assigned to a new genus. It is also proposed that *T. remsis* be transferred to *Knoellia* and that *T. duodecadis* and *T. elongata* be reclassified in *Phytococcus*.

It is evident from the phylogenetic analyses that the classification of *Monashia flava* (Azman et al., 2016), the type and only species of the genus, needs to be revised. In the phylogenomic tree (Figure 8), it was placed within a paraphyletic *Intrasporangium* (Figure 8), a relationship also found in the ULT, but with lower support (Supplementary File 2). In both the UCT and original 16S rRNA gene tree the position of *M. flava* was unresolved (Azman et al., 2016). Whereas, *Monashia* could well form the sister group of the three *Intrasporangium* species according to the 16S rRNA gene tree, a significant conflict with the other data sets is observed regarding the sister group of *I. oryzae*, which is either *M. flava* or *I. calvum*. Additional supermatrix analyses unambiguously confirmed *M. flava* to be most closely related to *I. oryzae* within the genus *Intrasporangium* (Supplementary File 2). The genome sequence of *Monashia flava*, while incomplete, showed no signs of contamination; additionally, the 5S, 16S, and 23S rRNA genes were located on the same contig (data not shown). Common chemotaxonomic features found between *Monashia* and *Intrasporangium* include a peptidoglycan containing LL-A_2_pm as the diagnostic diamino acid, an A3γ peptidoglycan type, major amounts of *iso*- and *anteiso-*fatty acids and MK-8(H_4_) as the predominant isoprenologue (Supplementary Table 1). In light of these phenotypic and phylogenetic data it is proposed that *M. flava* be assigned to *Intrasporangium*.

A recent study (Busse, 2016) led to an extensive revision of the classification of *Arthrobacter* (Conn and Dimmick, 1947; Koch et al., 1995) based primarily on chemotaxonomic traits though it remained unclear which ones where apomorphic character states. This strategy resulted in proposals for five new genera, namely *Glutamicibacter, Paenarthrobacter, Paeniglutamicibacter, Pseudarthrobacter*, and *Pseudoglutamicibacter*. However, it is apparent from the phylogenomic tree (Figure 8) that there are problems with this revised classification as the genera *Arthrobacter* and *Pseudoarthrobacter* do not form monophyletic groups. Further, the CCT does not yield strong support for the revised genera, including *Paenarthrobacter*, though it does not strongly suggest alternative groupings (Supplementary File 2). Additional genome sequences are needed to help chart a way forward.

### Species and subspecies

Known and confirmed heterotypic synonyms and dDDH values found to be higher or lower than expected given current species and subspecies thresholds of 70% (Wayne et al., 1987) and 79%, respectively (Meier-Kolthoff et al., 2014b) are shown in Table 1 for pairs of closely related strains. Only *Amycolatopsis keratiniphila* subsp*. keratiniphila* and *A. keratiniphila* subsp. *nogabecina* (Wink et al., 2003) displayed a value above the 79% threshold, namely 90.3%, hence it is proposed that the latter be recognized as a heterotypic synonym of the former; an amended description of *A. keratiniphila* (Al-Mussallam et al., 2003) is given. Conversely, several subspecies were shown to merit species status, they included *Bifidobacterium animalis* subsp. *lactis* (Masco et al., 2004), *B. longum* subsp. *infantis*, (Mattareli et al., 2008), *B. pseudolongum* subsp. *globosum* (Yaeshima et al., 1992), and *Nocardiopsis dassonvillei* subsp. *albirubida* (Evtushenko et al., 2000b). It is proposed that in all of these cases the originally species epithets should be reintroduced, namely *B. lactis* (Meile et al., 1997), *B. infantis* (Reuter, 1963), *B. globosum* (Biavati et al., 1982), and *N. alborubida* (Grund and Kroppenstedt, 1990). Other subspecies that require species status include *B. thermacidophilum* subsp. *porcinum* (Zhu et al., 2003), *Leucobacter musarum* subsp. *japonicus* (Clark and Hodgkin, 2015), and all three subspecies of *Clavibacter michiganensis* (Davis et al., 1984) in accordance with recent results (Li et al., 2017).

It was not possibly to distinguish between some pairs of validly named species even at the rank of subspecies since they showed a dDDH value >79% (Meier-Kolthoff et al., 2014b). These included *Cellulosimicrobium aquatile* (Sultanpuram et al., 2015) vs. *C. funkei* (Brown et al., 2006); *Dietzia cinnamea* (Yassin et al., 2006) vs. *D. maris* (Rainey et al., 1995b); and several *Bifidobacterium, Nocardiopsis*, and *Streptomyces* species. In the case of *Streptomyces* the results included synonyms well-known from the literature (Lanoot et al., 2005; Labeda et al., 2017). Similarly, *Saccharopolyspora jiangxiensis* (Zhang et al., 2009a) was shown to belong to the same species as *S. hirsuta* subsp. *kobensis* (Iwasaki et al., 1979; Lacey, 1989). Although a genome sequence is not currently available for *S. hirsuta* subsp. *hirsuta* (Lacey and Goodfellow, 1975) the two subspecies share a 16S rRNA gene sequence similarity of 98.23% (sequences DQ381814 and EU267029, both from LTP) indicating that they merit species status (Meier-Kolthoff et al., 2013b). Moreover, the two subspecies were placed apart in the CCT (Supplementary File 2) where *S. hirsuta* subsp. *hirsuta* grouped together with *S. hordei* (Goodfellow et al., 1989). In light of these findings it is proposed that *S. hirsuta* subsp. *kobensis* be elevated to species level and *S. jiangxiensis* be seen as its heterotypic synonym.

The dDDH results also showed that several species contain strains that need to be assigned to subspecies because they displayed values between 70 and 79% (Wayne et al., 1987; Meier-Kolthoff et al., 2014b). Consequently, it is proposed that *Bifidobacterium gallinarum* (Watabe et al., 1983)*, B. kashiwanohense* (Morita et al., 2011), and *B. saeculare* (Biavati et al., 1991) be classified as *B. pullorum* subsp. *saeculare* comb. nov*., B. pullorum* subsp*. gallinarum* comb. nov., and *B. catenulatum* subsp*. kashiwanohense* comb. nov. *Actinopolyspora iraqiensis* (Ruan et al., 1994) was considered to be a heterotypic synonym of *Saccharomonospora halophila* (Al-Zarban et al., 2002b) on the basis of experimental DDH results (Tang et al., 2011). However, using dDDH, it was found to be more closely related to *Saccharomonospora paurometabolica* (Li et al., 2003e), a relationship confirmed in the GBDP tree (Figure 2). Since *A. iraqiensis* has priority over *S. paurometabolica*, it is proposed that the former should be reclassified as *S. iraqiensis* comb. nov., with constituent strains recognized as S. *iraqiensis* subsp. *iraqiensis* comb. nov. and S. *iraqiensis* subsp. *paurometabolica* comb. nov. The results regarding *Oerskovia xanthineolytica* (Lechevalier, 1972) were unexpected because it is considered to be a heterotypic synonym of *C. cellulans* not of *C. aquatile* (Schumann et al., 2001) and because the GBDP tree indicated a distinct arrangement (Figure 7). In contrast to the other genomes investigated, the contigs of the genome sequence of *O. xanthineolytica* showed a somewhat heterogeneous G+C content distribution (data not shown) hence it cannot be ruled out that the sequence used was impure. Consequently, the relationships between *O. xanthineolytica* to related taxa needs to be revisited.

Finally, in the present study, all of the pairs of strains considered to represent distinct deposits of the same type strain were found to have d dDDH similarities of 99.0% or above with the exception of *Amycolatopsis methanolica* (De Boer et al., 1990) strain 239 vs. DSM 44096 (98.3%), *Bifidobacterium longum* subsp. *longum* (Mattareli et al., 2008) strain DSM 20219 vs. JCM 1217 (89.2%) and *Pseudarthrobacter chlorophenolicus* (Busse, 2016) strain A6 vs. DSM 12829 (97.3%), results which may account for the separation of each pair of these strains.

### Relationship between genomic G+C content, genome size, and phylogeny

**Figure 9:**
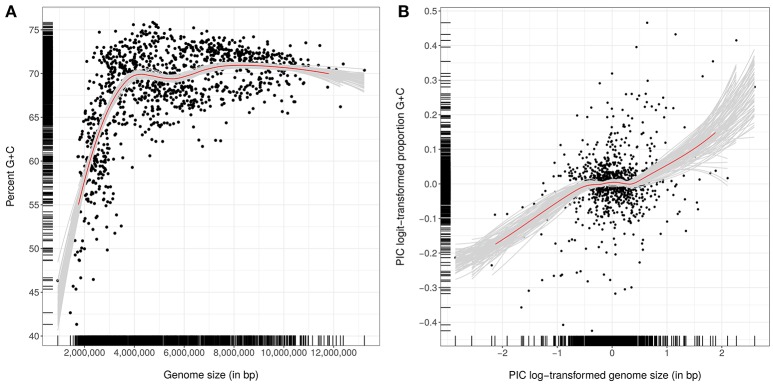
**(A)** Percent G+C content in dependence on genome size (in numbers of base pairs). **(B)** Phylogenetically independent contrasts (PICs) derived from logit-transformed proportions of G+C content in dependence on PICs derived from log-transformed genome sizes. PICs were calculated to account for the significant effect of the phylogeny on both G+C content and genome size. In both panels, Loess-based bootstrap aggregation (100 replicates) was applied; the resulting red curve denotes the average from 100 smoothers, each fitted to a subset of the original data set, to increase stability and reduce overfitting.

Genome size and G+C content appear to be phylogenetically conserved (*p* = 0.0001), a result that is in good agreement with those shown in Figures 1–8 where many of the clades encompassed strains that had similar genome sizes and G+C contents. In addition, genome size and G+C content were positively correlated (Pearson, 0.612, *p* < 10^−15^; Kendall, 0.339, *p* < 10^−15^) though the relationship was not linear (Figure 9A). In the case of the phylogenetically independent contrasts significant but weaker correlations (Pearson, 0.298, *p* < 10^−15^; Kendall, 0.043, *p* = 0.0409) were found (Figure 9B).

Experimentally derived G+C values cited in the descriptions of many species were found to be incorrect given corresponding data drawn from whole genome sequences while in other species descriptions G+C values were not available. These errors and omissions are addressed for many species using data drawn from genome sequences (see section on taxonomic revision).

## Discussion

### Conflict and agreement in phylogenetic trees from genome-scale data

Conflict with monophyly in a range of taxa was frequently seen in our genome-scale analyses though instances where a taxon was strongly supported in the GBDP tree but seriously out of kilter with corresponding 16S (or 23S) rRNA gene trees, or *vice versa*, were rarely observed (Supplementary File 2). As in previous studies, analyses of the genome-scale data resulted in more strongly resolved trees though it is known that such studies may increase incongruities between distinct analyses (Jeffroy et al., 2006; Klenk and Göker, 2010). Indeed, topological incongruities between analyses of single genes attributed to horizontal gene transfer have been used to argue against the concept of hierarchical classification (Bapteste and Boucher, 2009; Klenk and Göker, 2010). However, the addition of more genes, up to virtually all available ones, increases support in phylogenomic trees (Breider et al., 2014) thereby indicating a strong hierarchical signal. Phylogenetic inferences drawn from limited studies on few genes can hardly be called genome-scale, such studies rely on assumptions about the relative suitability of the selected genes (Lienau and DeSalle, 2009; Klenk and Göker, 2010). In contrast, whole-genome methods, such as GBDP, yield truly genome-based phylogenies though overestimating phylogenetic confidence from genome-scale data must be avoided (Taylor and Piel, 2004). Bootstrapping entire genes instead of single alignment positions can reduce incongruities between trees and thereby provide more realistic phylogenomic support values (Siddall, 2010; Simon et al., 2017) hence in this study partition bootstrapping was applied throughout in our supermatrix analyses. GBDP pseudo-bootstrapping in conjunction with the greedy-with-trimming algorithm (Meier-Kolthoff et al., 2014a) is akin to partition bootstrap.

It was particularly interesting that in the present study there were three cases where the 16S rRNA gene tree was somewhat misleading, two with respect to *Intrasporangiaceae* and one regarding the monophyly of *Mycobacteriaceae*. In all of these instances the 23S rRNA gene trees showed better congruence with the GBDP tree, albeit with low support, whereas the corresponding supermatrix analyses fully confirmed the results of the GBDP analyses. Potential causes for the probable incorrect 16S rRNA gene phylogenies include insufficient taxon sampling and misspecified models but it cannot be ruled out that in these cases the 16S rRNA gene truly conflicts with the organism tree. *Atopobium* and *Olsenella* also provided an interesting case because here the 23S rRNA gene analyses were strongly in conflict with the corresponding GBDP analyses. These differences might be due to horizontal gene transfer, particularly in light of the conflicting signal between protein-coding genes indicated by low partition bootstrap support in the supermatrix analyses (Supplementary File 2). However, irrespective of the conflicting topologies the ML and MP supermatrix trees indicate that *Atopobium* is not monophyletic.

It is encouraging that the taxonomic conclusions drawn from the GBDP tree were confirmed by corresponding supermatrix analyses in all of the cases investigated. Consequently, we believe that the approach used to assess whether conflict was evident between trees inferred from distinct data sets followed, where necessary, by additional analyses was a robust procedure. This also holds for the application of backbone constraint to augment comprehensible sampled single-gene data with information drawn from analyses of additional genes but fewer organisms (Liu et al., 2015; Hahnke et al., 2016). While the CCT allowed us to safely place type species lacking genome sequences and in one case to delineate an additional genus, it also highlighted other instances where taxonomic conclusions would have been premature.

### Incongruities between phylogenomic analyses and taxonomic classification

The origin of the non-monophyletic taxa highlighted in this study cannot be understood without understanding the taxonomic practices used to generate these taxa. The current intense debate on how to describe new taxa focusses mainly on the question of which kind of characters should be investigated and in this context primarily on the contrast between phenotypic and genomic data (Vandamme and Peeters, 2014; Sutcliffe, 2015; Thompson et al., 2015). Based on the principles of phylogenetic systematics (Hennig, 1965; Wiley and Lieberman, 2011) and observations made in the course of this study we believe that the focus should be on character interpretation rather than on character sampling. In the current dominant practice of polyphasic taxonomy (Vandamme et al., 1996) 16S rRNA gene trees are usually inferred as the initial step following the identification of the taxa of interest; subsequently, new taxa are chosen from such trees and “diagnostic” features presented. Phenotypic characters still tend to be interpreted manually, they are rarely analyzed by clustering, let alone by using phylogenetic methods (Montero-Calasanz et al., 2017).

As for the initial step, almost all of the taxonomic problems observed in this study concerned taxa for which statistical support for their monophyly was not apparent in 16S rRNA gene trees, irrespective of whether they were inferred by us or depicted in the literature. In these single-gene trees the questionable taxa appeared to be monophyletic but with low bootstrap support or paraphyletic with weak (e.g., *Amycolatopsis*) to moderate (e.g., *Corynebacterium*) support against their monophyly. Thus, calculating branch support from single genes in an appropriate manner (or from genome-scale data as described above) is a necessary, but not a sufficient prerequisite, for safely generating monophyletic taxa; indeed taxa must also be chosen from trees so as to correspond to highly supported clades (Vences et al., 2013).

**Figure 10:**
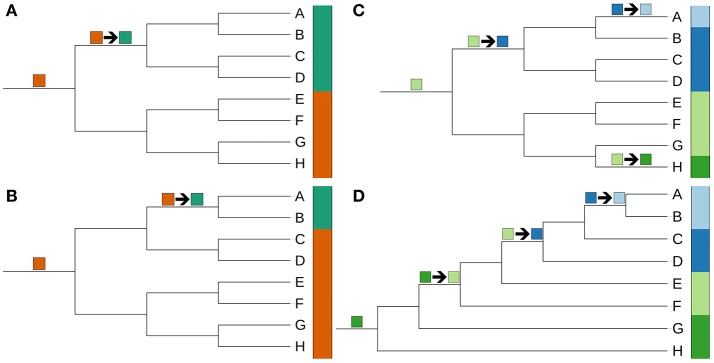
Hypothetical trees and character-state distributions illustrating the impossibility to detect “diagnostic” character states for monophyletic groups even in the case of a perfect fit of the character to the phylogeny. Most-parsimonious reconstructions of changes between character states are indicated by arrows. **(A)** Maximally symmetric tree with eight tips and a binary character without homoplasies. Each of the two character states is diagnostic for a clade. The orange-red state is actually plesiomorphic but accidentally happens to occur in a single clade only. **(B)** Same tree as before together with another binary character without homoplasies. Only one of the two character states is diagnostic for a monophyletic group. The orange-red state is again plesiomorphic, hence it comes as no surprise that it characterizes a paraphyletic group. **(C)** Same tree as before in conjunction with a multi-state character without homoplasies. Only two of the four states, light blue and dark green, are diagnostic, each for a distinct clade. **(C)** Maximally asymmetric tree with eight tips in conjunction with another multi-state character without homoplasies. Only one of the four states, light blue, is diagnostic for a clade.

The use of diagnostic features in support for taxa is a practice that clashes with the principles of phylogenetic systematics which emphasize apomorphies as evidence for the monophyly of a group (Hennig, 1965; Wiley and Lieberman, 2011). Since we were unable to detect the use of the term “apomorphy” in any of the taxonomic publications evaluated in the course of this study, we would like to draw attention to this issue by reference to Figure 10. The pitfalls of relying on diagnostic features do not originate from the extent of homoplasy, which is zero in the case of the trees and characters in Figure 10, hence the issue at hand is not one of data quality. In the case of binary characters, such as the presence of mycolic acids in *Corynebacterium* against their absence in *Turicella*, even when homoplasies are lacking only one of the two characters states can be used to support the monophyly of a group as the other state is plesiomorphic. As shown in Figure 10A, the group recognized by the plesiomorphic state might accidentally be monophyletic, but it is more likely to be paraphyletic as depicted in Figure 10B. Reptiles were originally the best known example of a paraphyletic group, as they are perfectly defined by plesiomorphic features (Wiley and Lieberman, 2011). A single feature with two character states cannot be used to justify the separation of two distinct taxa because only the apomorphic character state can argue for the monophyly of one of the two taxa. The same holds for multiple binary characters provided one of the two taxa is plesiomorphic regarding all of them. Thus, contrary to a recent study (Baek et al., 2018), we conclude that discrepancies between traditional taxonomy and phylogenomic trees do not necessarily indicate that the features on which the traditional classification was based are in true conflict with the tree and that these features *per se* need to be treated with caution.

In the case of multi-state characters, such as 16S rRNA gene signatures (Zhi et al., 2009) or peptidoglycan types [the latter have been particularly important in the classification of Gram-positive taxa, notably with respect to *Actinobacteria* (Schleifer and Kandler, 1972)], the difference between diagnostic features and apomorphies can be even more striking. Figure 10C illustrates a case of four characters states only two of which (light blue, dark green) define monophyletic groups whereas the other two states highlight paraphyletic groups. In the asymmetric tree (Figure 10D) only one of the four character states (light blue) defines a clade. The practice of only recognizing genera or higher taxa that are uniform with respect to single characters, such as peptidoglycan types and 16S rRNA gene signatures, should be discontinued as it is not sufficiently flexible to account for the various possible ways of character evolution even when homoplasies can be ruled out. The dark blue character state shown in Figure 10D, for instance, is actually a synapomorphy of strains A-D and hence plesiomorphic within that group; its change to light blue in the last common ancestor of A+B does not imply a homoplasy. Considering, the character to be diagnostic would either yield a paraphyletic taxon (C+D) or three taxa (A+B, C, and D) two of which cannot be distinguished using this character. The latter solution might amount to excessive splitting. Consequently, it would be better if taxa were allowed to have distinct character states even if the characters were previously considered to be diagnostic. An additional problem of 16S rRNA gene signatures is that they represent part of the information used to infer 16S rRNA gene trees and hence cannot provide independent evidence.

Attempts to classify large numbers of taxa using low numbers of characters (e.g., phenotypic ones) may frequently lead to the circumscription of “waste bin” taxa composed of groups that have no apparent apomorphy. This not only holds true for prokaryotic systematics but also for large non-monophyletic yeast genera, such as *Candida* (Daniel et al., 2014), *Cryptococcus* (Liu et al., 2015), and *Rhodotorula* (Wang et al., 2015), which in recent years have been split based on data derived from phylogenetic analyses. Indeed, in such instances the goal that taxonomic classification should be based on monophyletic taxa can be in conflict with the goal of providing apomorphies for all taxa, let alone diagnostic features, particularly for low numbers of previously selected (phenotypic or other) characters. As long as small numbers of characters are the limiting factor, taxonomic classification cannot fully benefit from the high resolution of phylogenetic trees based on available genome-scale data (Klenk and Göker, 2010). In cases such as *Actinomyces* we have favored the creation of taxa phylogenetically well-supported as monophyletic over insisting on recognizable apomorphies of selected characters. Researchers primarily interested in the differences in such characters could well emend taxon descriptions provided such differences were found (and, hopefully, synapomorphies would be among them). In the meantime those using taxonomic classifications can benefit from taxa that now more likely represent natural groups.

Since single characters do not yield bootstrapping or other statistical support it is not clear how to apply them in rigorous tests of the plausibility of certain phylogenetic outcomes. According to Dollo's law complex features arise only once in evolution but may be lost several times (Farris, 1977), hence a group of organisms displaying a complex feature should be monophyletic or paraphyletic in a tree, but not polyphyletic. The acid-fastness of most *Corynebacteriales*, indicating a multi-layered cell wall containing mycolic acids (Barry et al., 1998), might well-represent such a complex feature. If so, our GBDP tree (Figure 3) passed the test because *Corynebacteriales* appear to be monophyletic whereas the strains containing mycolic acids appear to be paraphyletic. Independent losses of mycolic acids within *Corynebacteriales* (Baek et al., 2018) indeed yield homoplasy but this is as expected under Dollo's law. The presence of mycolic acids, the apparent apomorphy of *Corynebacteriales*, was thereby shown to be plesiomorphic within the order and lost again in groups such as *Turicella*. A similar reasoning can be applied to rare peptidoglycan types which might characterize monophyletic or paraphyletic groups as in Figure 9D.

The results of the dDDH analyses revealed a number of new subspecies. In many cases this came as no surprise as these taxa had already regarded as later heterotypic synonyms while a dDDH subspecies boundary has only recently been introduced (Meier-Kolthoff et al., 2014b). In our view, applying a firm dDDH boundary for subspecies is a logical development analogous to cut-off values for the delineation of species. Moreover, dDDH similarities are more likely to yield monophyletic subspecies (Meier-Kolthoff et al., 2014c) than a selection of phenotypic differences based on a few characters. In other cases, heterotypic synonyms postulated in the literature or species regarded as distinct based on traditional DDH values, could not be confirmed. The most likely reason for this is that traditional DDH procedures are more error-prone than dDDH (Meier-Kolthoff et al., 2013a). Much like Average Nucleotide Identity (ANI), the choice of a similarity method and a species cut-off for dDDH was based on mimicking traditional DDH as closely as possible, albeit without its problematic reproducibility; however, according to this criterion dDDH yielded better results than ANI and can thereby be seen as the method of choice (Meier-Kolthoff et al., 2013a). For instance, the recent controversy regarding the subspecies of *Mycobacterium abscessus* (Adekambi et al., 2017; Tortoli et al., 2017) may have been avoided if dDDH had been preferred or pairwise comparison had been restricted to those involving type strains (Meier-Kolthoff et al., 2014c).

### Genomic G+C content and genome size as taxonomic markers

The significant correlation found between genome size and G+C content was not unexpected as trends to both larger genomes and higher G+C content in free-living organisms have been postulated previously (Mann and Chen, 2010). Potential mechanisms that increase genomic G+C content include positive selection (Hildebrand et al., 2010) and G+C-biased gene conversion (Lassalle et al., 2015). In contrast, symbiotic bacteria tend to have smaller genomes due to reductive evolution and to be richer in A+T content due to nutrient limitations as A+T synthesis is energetically less demanding (Rocha and Danchin, 2002; Mann and Chen, 2010). This can be easily confirmed for mutualistic actinobacteria such as *Bifidobacterium* (Supplementary File 2) and for parasitic ones like *Tropheryma* (Figure 1). In addition, we have shown that the relationship between genome size and G+C content is non-linear at least within the phylum *Actinobacteria* (Figure 9). The ability to conduct such analyses for an entire phylum underlines the advantage of comprehensive taxonomic treatments over ones restricted to specific taxa (Hahnke et al., 2016). A non-linear relationship has also been observed between genome size and G+C content of monocotyledonous plants (Šmarda et al., 2014) where it even appeared to be regularly quadratic.

As for the use of genome size as a taxonomic marker, it can be regarded as non-independent of G+C content. However, due to the non-linear relationship this dependency is much lower for higher genome sizes (Figure 9). Indeed, if one takes into account that, on average, larger genomes also display a greater variance as they represent count data (Crawley, 2007), genome sizes often appeared to be genus-specific (Figures 1–8). The correlation between G+C content and genome size was still significant after accounting for the impact of the phylogeny albeit considerably weaker (Figure 9). Both bacterial G+C content and bacterial genome size appeared to be strongly phylogenetically conserved, an observation that may have been underestimated in previous studies. It is for these reasons that genome sizes have been added to the description of the reclassified and emended species as shown below. As in the case of the *Bacteroidetes* (Hahnke et al., 2016), many species descriptions were found to be inaccurate or too imprecise now that it has been shown that within-species deviation in genomic G+C content is at most 1% (Meier-Kolthoff et al., 2014c). It is good practice to strengthen species descriptions in this way as such values not only assist in highlighting strains that do not belong to the same species but also show significant correlation to phylogenetic trees. In contrast, it is premature to redefine actinobacterial genera and higher taxa in this way; as additional type-strain genome sequences are needed before this issue can be addressed.

### Applications of the improved classification of *Actinobacteria*

The results of this comparative phylogenomic study provide a much improved framework for the classification of the phylum *Actinobacteria*, one of the largest taxonomic lineages recognized in the domain *Bacteria*. The improved classification provides a sound basis for future studies on actinobacteria, not least on those of agricultural, biotechnological, clinical, and ecological interest. In particular, the clarification of relationships within taxa that, until now, have confounded the best efforts of actinobacterial systematists (Ludwig et al., 2012) will be of especial interest to those involved in developing biotechnological applications of strains belonging to taxa classified in emended families such as the *Gordoniaceae, Micromonosporaceae, Nocardiaceae*, and *Streptomycetaceae*. Similarly, improvements in the classification of taxa assigned to emended families, notably *Actinomycetaceae* and *Corynebacteriaceae*, will provide valuable leads to those working on organisms relevant to human and veterinary medicine. Indeed, it can be concluded that comparative genomic analyses not only provide “grist to the taxonomic mill” (Klenk and Göker, 2010; Sutcliffe et al., 2012), as exemplified in this study, but also provide invaluable insights into actinobacterial biology in its entirely thereby helping to revitalize prokaryotic systematics as a fundamental scientific discipline.

### Taxonomic consequences: new taxa

#### Description of *Cryptosporangiales*, ord. nov.

Cryp.to.spo.ran.gi.a'les (N.L. neut. n. *Cryptosporangium*, type genus of the order; -*ales*, ending to denote an order; N.L. fem. pl. n. *Cryptosporangiales*, the *Cryptosporangium* order).

The description is as given for *Cryptosporangiaceae* (Zhi et al., 2009; Sen et al., 2014). The type and only genus of the order is *Cryptosporangium* (Tamura et al., 1998).

#### Description of *Sporichthyales*, ord. nov.

Spo.rich.thy.a'les (N.L. fem. n. *Sporichthya*, type genus of the order; -*ales*, ending to denote an order; N.L. fem. pl. n. *Sporichthyales*, the *Sporichthya* order).

The description is as given for *Sporichthyaceae* (Stackebrandt et al., 1997; Zhi et al., 2009). The type and sole genus of the order is *Sporichthya* (Lechevalier et al., 1968).

#### Description of *Actinopolymorphaceae*, fam. nov.

Ac.ti.no.po.ly.mor.pha'ce.ae (N.L. fem. n. *Actinopolymorpha*, type genus of the family; -*aceae*, ending to denote a family; N.L. fem. pl. n. *Actinopolymorphaceae*, the *Actinopolymorpha* family).

Aerobic, Gram-stain-positive, non-acid-alcohol-fast, non-motile organisms that form irregular cells. Aerial mycelium is absent or scant. The diamino acid of the peptidoglycan is LL-A_2_pm. The predominant respiratory quinones are either MK-9 or MK-10 and the major polar lipids DPG, PG and PI together with phosphatidylglycolipids. The genomic G+C content is around 66–73%. The family comprises *Actinopolymorpha* (Wang et al., 2001), the type genus, *Flindersiella, Tenggerimyces*, and *Thermasporomyces*.

#### Description of *Antricoccaceae*, fam. nov.

An.tri.coc.ca'ce.ae (N.L. masc. n. *Antricoccus*, type genus of the family; -*aceae*, ending to denote a family; N.L. fem. pl. n. *Antricoccaceae*, the *Antricoccus* family).

The description is as given for *Antricoccus* (Lee, 2015), the type and sole genus of the family.

#### Description of *Jatrophihabitantaceae*, fam. nov.

Ja.tro.phi.ha.bi.tan.ta'ce.ae (N.L. masc. n. *Jatrophihabitans*, type genus of the family; -*aceae*, ending to denote a family; N.L. fem. pl. n. *Jatrophihabitantaceae*, the *Jatrophihabitans* family).

The description is as given for *Jatrophihabitans* (Madhaiyan et al., 2013), the type and sole genus of the family.

#### Description of *Kribbellaceae*, fam. nov.

Krib.bel.la'ce.ae (N.L. fem. n. *Kribbella*, type genus of the family; -*aceae*, ending to denote a family; N.L. fem. pl. n. *Kribbellaceae*, the *Kribbella* family).

Aerobic, Gram-stain-positive organisms which form extensively branched hyphae that penetrate into agar media. Typically produce aerial hyphae; substrate and aerial hyphae usually show various degrees of fragmentation eventually resulting in the production of coccoid to irregular, non-motile, rod-shaped elements. Anaerobic growth has been detected. The wall peptidoglycan contains LL-A_2_pm, alanine, glutamic acid, and glycine; muramic acid residues of the peptidoglycan are N-acetylated; the main cell-wall polymer is either teichuronic or teichulosonic acid. The cellular fatty acids are mainly saturated, *iso*- and *anteiso-*branched acids, and the predominant isoprenoid quinone is MK-9(H_4_). An ubiquitous polar lipid is PC, additional components include DPG, PG, and PI. The genomic G+C content is around 65–77%. The family comprises only *Kribbella* (Park et al., 1999; Sohn et al., 2003; Everest et al., 2013), the type genus.

#### Description of *Kytococcaceae*, fam. nov.

Ky.to.coc.ca'ce.ae (N.L. masc. n. *Kytococcus*, type genus of the family; -*aceae*, ending to denote a family; N.L. fem. pl. n. *Kytococcaceae*, the *Kytococcus* family).

Aerobic, Gram-stain-positive, asporogenous organisms which form coccoid cells. The peptidoglycan contains L-lysine as the diaminoacid and is of the A4α type. The predominant isoprenologues are MK-7, MK-8, MK-9, and MK-10 and the major polar lipids DPG, PG, and PI. The predominant cellular fatty acids are *anteiso*-C_17:0_ and *iso*-C_17:1_. Does not contain mycolic acids or teichoic acids. The genomic G+C content is around 68–73%. The family only contains *Kytococcus* (Stackebrandt et al., 1995), the type genus.

#### Description of *Lawsonellaceae*, fam. nov.

Law.so.nel.la'ce.ae (N.L. fem. n. *Lawsonella*, type genus of the family; -*aceae*, ending to denote a family; N.L. fem. pl. n. *Lawsonellaceae*, the *Lawsonella* family).

The description is as given for *Lawsonella* (Bell et al., 2016), the type and sole genus of the family.

#### Description of *Ornithinimicrobiaceae*, fam. nov.

Or.ni.thi.ni.mi.cro.bi.a'ce.ae (N.L. neut. n. *Ornithinimicrobium*, type genus of the family; -*aceae*, ending to denote a family; N.L. fem. pl. n. *Ornithinimicrobiaceae*, the *Ornithinimicrobium* family).

Aerobic to microaerobic, Gram-stain-positive, non-motile organisms which form short irregular rods and/or cocci. The peptidoglycan type is A4β, and muramic acid residues of the peptidoglycan are N-acetylated. The main cellular fatty acids are *anteiso-* and *iso*-branched acids, the predominant isoprenologue is MK-8(H_4_) and the major whole-cell sugars are arabinose, rhamnose and xylose. The polar lipid profile contains DPG, PG and PI. Does not have mycolic acids. Catalase positive and oxidase negative. The genomic G+C content is around 68–74%. The family includes *Ornithinimicrobium* (Groth et al., 2001), the type genus, and *Serinicoccus*.

#### Description of *Tropherymataceae*, fam. nov.

Tro.phe.ry.ma.ta'ce.ae (N.L. neut. n. *Tropheryma*, type genus of the family; -*aceae*, ending to denote a family; N.L. fem. pl. n. *Tropherymataceae*, the *Tropheryma* family).

The description is as given for *Tropheryma* (La Scola et al., 2001) with the following additions. Grows in tissue culture media in co-culture with human fibroblasts axenically in such media enriched with amino acids. Does not grow on standard laboratory media. Forms straight regular small rods. Gram-stain-negative in culture but variably Gram-stain-positive in infected human tissues. The genomic G+C content is around 46%. The type and only genus is *Tropheryma* (La Scola et al., 2001).

#### Description of *Aldersonia*, gen. nov.

Al.der.so'ni.a (N.L. fem. n. *Aldersonia*, named after Grace Alderson, a British microbiologist who made significant contributions to rhodococcal systematics).

Aerobic, Gram-stain-positive, acid-fast, non-motile organism which forms filaments or shows elementary branching in early growth phase and produces mainly cocci in stationary phase. Displays a rod -coccus growth cycle. Circular, convex, pink colonies are formed on yeast extract-malt extract agar after incubation for 7 days at 28°C. Whole-cell hydrolysates contain DL-A_2_pm, arabinose and galactose, the predominant isoprenologue is MK-8(H_2_) and DPG, PE, PI and PIM's are evident in polar lipid profiles. The major fatty acids are C_16:0_, C_18:1_ ω9c, and C_16:1_ ω7c. The genomic G+C content is about 66%. The type species is *Aldersonia kunmingensis* comb. nov.

#### Description of *Boudabousia*, gen. nov.

Bou.da.bou'si.a (N.L. fem. n. *Boudabousia*, named after Abdellatif Boudabous Chihi, the first microbiologist in Tunisia, for his contributions to prokaryotic systematics).

Facultatively anaerobic, Gram-stain-positive, non-acid-fast, non-motile, catalase-negative, non-hemolytic organisms which form straight to slightly curved rods, some show branching. Colonies are gray, entire, convex, and pinpoint to 0.5 mm in diameter after 48 h. The major fatty acids are C_16:0_, C_18:0_, and C_18:1_ cis9. The genomic G+C content is about 54%. The type and only species is *Boudabousia marimammalium* comb. nov.

#### Description of *Bowdenia*, gen. nov.

Bow.de'ni.a (N.L. fem. n. *Bowdenia*, named after George H. Bowden, a Canadian microbiologist, for his work on oral microbiology).

Facultatively anaerobic, Gram-stain-positive, catalase-negative organisms which form short diphtheroid shaped rods; branched elements and coccoid forms may be found. Pinpoint, white or gray, opaque, shiny, entire, and convex colonies produced after 48 h under anaerobic conditions. The major isoprenologue is MK-9. The fatty-acid profile contains major amounts of *anteiso*-C_15:0_, C_16:0_, C_18:0_, and C_18:1_ w9c. The genomic G+C content is around 65%. The type species is *Bowdenia nasicola* comb. nov.

#### Description of *Buchananella*, gen. nov.

Bu.cha.na.nel'la (L. fem. dim. ending -*ella*; N.L. fem. n. *Buchananella*, named after Robert E. Buchanan, an American microbiologist who made significant contributions to prokaryotic systematics).

Facultatively anaerobic, Gram-stain-positive, weakly catalase-positive organisms which form pleomorphic rods and filaments; the latter show branching on blood agar. Microcolonies are filamentous on brain-heart-infusion agar supplemented with bovine serum. Mature colonies on bovine blood agar are white, adhere to the agar and are molar-toothed. The peptidoglycan contains L-lysine, alanine, and glutamic acid; some strains also have L-ornithine. The muramic acid moiety of the peptidoglycan is N-glycosylated. Cell-wall sugars include galactose and glucose, but not 6-deoxytalose or rhamnose. The G+C content is around 67–68%. The type species is *Buchananella hordeovulneris* comb. nov.

#### Description of *Jongsikchunia*, gen. nov.

Jong.sik.chu'ni.a (N.L. fem. n. *Jongsikchunia*, named after Jongsik Chun, a Korean microbiologist, for his extensive work on actinobacterial systematics).

Aerobic, Gram-stain-positive, non-motile organisms that form non-spore-forming rods and display a rod-coccus growth cycle. Colonies are white, opaque and polymorphic on complex media. Catalase positive and oxidase negative. Whole-cell hydrolysates contain DL-A_2_pm, arabinose and galactose. The muramic acid residues of the peptidoglycan are N-glycolated. The major fatty acids are C_16:1_ ω7c, C_16:0_, C_18:1_ ω9c, and 10-methyl C_18:0_; the predominant isoprenologue MK-9(H_2_); the polar lipid profile contains DPG, PE, PI, and PIMs. The principal mycolic acids have a chain length of 58-60 carbon atoms. The genomic G+C content is around 67%. The type species is *Jongsikchunia kroppenstedtii* comb. nov.

#### Description of *Flavimobilis*, gen. nov.

Fla.vi.mo'bi.lis (L. adj. *flavus*, yellow, pertaining to the yellow color of the colonies; L. adj. *mobilis*, mobile; N.L. masc. n. *Flavimobilis*, a yellow motile bacterium).

Aerobic or facultative anaerobic, Gram-stain-positive, motile, asporogenous organisms which form regular of irregular short rods. They grow from 15 to 42°C; arbutin and starch are degraded. The peptidoglycan contains meso-A_2_pm as the diamino acid and is of the A4α type. The cellular fatty-acid profile contains major amounts of *anteiso*-methyl branched and straight-chain saturated components; the predominant isoprenologue is MK-9(H_4_). Does not form mycolic acids. The genomic G+C content is around 72%. The type species is *Flavimobilis marinus* comb. nov.

#### Description of *Embleya*, gen. nov.

Em.ble'ya (N.L. fem. n. *Embleya*, named after the British microbiologist T. Martin Embley for his contribution to actinobacterial systematics).

Well-developed, irregularly branched substrate and aerial mycelium formed. A gray aerial spore mass is produced on oatmeal agar. Mature spore chains are spiral with more than 20 cylindrical, rugose ornamented spores per chain. Whole-cell hydrolysates contain LL-A_2_pm and traces of arabinose but lack galactose. The muramic acid residues of the peptidoglycan are N-glycolated. The major isoprenologue is MK-9(H_4_) and the predominant phospholipids PE and an unidentified glucosamine component. The genomic G+C content is about 70%. The type and only species is *Embleya scabrispora* comb. nov.

#### Description of *Gleimia*, gen. nov.

Glei'mi.a (N.L. fem. n. *Gleimia*, named after Dorothea Gleim, a German microbiologist, for her work on the DSMZ Prokaryotic Nomenclature up-to-date Service).

Facultatively anaerobic, non-motile, Gram-stain-positive, slightly curved rods or short rods or coryneform cells are formed. The peptidoglycan type is A5α. The fatty-acid profile contains major amounts of C_16:0_, C_18:1_ ω9, C_18:0_, and C_14:0_. The type species is *Gleimia europaea* comb. nov.

#### Description of *Fannyhessea*, gen. nov.

Fan.ny.hes'se.a (N.L. fem. n. *Fannyhessea*, of Hesse, named to honor Angelina Fanny Hesse for her great contribution to microbiology, the use of agar to solidify media).

Microaerophilic or anaerobic, Gram-stain-positive cocci which occur singly, in pairs or short chains. Acid is not produced from either mannose or raffinose. Indole negative. Nitrate is not reduced. Gelatin and aesculin are not hydrolysed. Predominant fatty acids are C_16:0_, C_18:0_, and C_18:1_ ω9c. The genomic G+C content is around 43%. The type species is *Fannyhessea vaginae*.

#### Description of *Lancefieldella*, gen. nov.

Lan.ce.fiel.del'la (L. fem. dim. ending -*ella*; N.L. fem. n. *Lancefieldella*, named after Rebecca Lancefield, an American microbiologist known for her significant contributions to *Streptococcus* biology).

Obligately anaerobic, Gram-stain-positive, non-motile, asporogenous, small rods which occur singly, in pairs or short chains. Central swellings may occur particularly in cells grown on solid media without Tween. Colonies on brain-heart blood agar range from pinpoint to 2 mm in diameter and are raised or low convex, entire and translucent or transparent after anaerobic incubation for 2 to 5 days at 37°C. Growth is stimulated by 0.02% (v/v) Tween 80 with 10% (v/v) rabbit serum. Acid is produced from mannose and glucose. Indole, gelatin and catalase negative. Predominant fatty acids are C_18:1_ ω9c and C_18:1_ dimethylacetyl. The genomic G+C content is around 39–45%. The type species is *Lancefieldella parvula* comb. nov.

#### Description of *Pauljensenia*, gen. nov.

Paul.jen.se'ni.a (N.L. fem. n. *Pauljensenia*, named after Paul Jensen, an American microbiologist, for his work on *Salinispora* from ecological and taxonomic perspectives).

Aerobic, Gram-stain-positive, catalase-negative, non-hemolytic organisms that form straight short to medium rods. Pinpoint colonies are formed on sheep blood agar after anaerobic incubation for 24 h. The type and only species is *Pauljensenia hongkongensis* comb. nov.

#### Description of *Schaalia*, gen. nov.

Schaa'li.a (N.L. fem. n. *Schaalia*, named after Klaus P. Schaal, a German medical microbiologist, for his extensive work on *Actinomyces* systematics).

Aerobic, Gram-stain-positive organisms which form straight to slightly curved rods some of which may show branching; some species only produce coccoid or coccobacillary cells. Microcolonies are not filamentous. Peptidoglycan contains L-lysine as the diamino acid and is either of the A5α or A5β type; the muramic acid residue of the peptidoglycan is N-acetylated. The major cell-wall sugars are mannose and rhamnose; the predominant menaquinone is MK-9(H_4_), minor amounts of MK-10(H_4_) and MK-8(H_4_) also occur. The major polar lipids are DPG and PG. Fatty-acid profiles are rich in saturated, unsaturated and *iso*- and *anteiso-*branched-chain components The genomic G+C content is 56–70%. The type species is *Schaalia odontolytica* comb. nov.

#### Description of *Pedococcus*, gen. nov.

Pe.do.coc'cus (Gr. n. pedon, the ground, earth; N.L. masc. n. *coccus*, (from Gr. n. *kokkos*) a grain or berry; N.L. masc. n. *Pedococcus*, coccus from soil).

Aerobic, Gram-stain-positive, asporogenous, catalase-positive organisms which form cocci or short rods. Circular, smooth, convex, cream, white or yellow colonies are formed. The peptidoglycan contains DL-A_2_pm as the diamino acid, demethylmenaquinone MK-8(H_4_) is the predominant isoprenologue and the main fatty acids are *iso*-C_15:0_ and *iso*-C_16:0_ acids. The genomic G+C content is about 69–74%. The type species is *Pedococcus dokdonensis* comb. nov.

#### Description of *Winkia*, gen. nov.

Win'ki.a (N.L. fem. n. *Winkia*, named after Joachim Wink, a German microbiologist who has made a significant contributions to actinobacterial systematics).

Facultatively anaerobic, Gram-stain-positive, asporogenous, catalase-positive organisms that are predominantly diphtheroid though coccobacilli may occur, rods are mainly arranged in clusters or as V or Y forms. Colonies are circular, smooth, convex, more white than cream colored with entire edges and 0.5 to 1.5 mm in diameter following incubation for 48 h. Straight-chain fatty acids are mainly palmitic and stearic acids. The genomic G+C content is around 55–58%. The type species is *Winkia neuii* comb. nov.

#### Description of *Yinghuangia*, gen. nov.

Ying.hu.an'gi.a (N.L. fem. n. *Yinghuangia*, named after Ying Huang, a Chinese microbiologist, for her work on the reclassification of *Streptomyces* based on MLSA).

Aerobic, Gram-stain-positive organisms which form an extremely branched substrate mycelium that bears an abundant aerial spore mass on yeast extract/malt extract agar. Produces imperfect spiral chains (rectinaculiapetri) or flexuous to long chains of smooth-surfaced spores. The wall peptidoglycan contains LL-A_2_pm as the diamino acid; whole-organism hydrolysates contain galactose, mannose and ribose or arabinose, glucose, rhamnose, and ribose. The predominant isoprenologues are MK-9(H_4_, H_6_, H_8_) and the major polar lipids DPG, PE, and PI; PC has also been reported. The fatty-acid profiles contain major amount of saturated, *iso*-and *anteiso*-fatty acids. The genomic G+C content is around 70–75%. The type species is *Yinghuangia aomiensis* comb. nov.

#### Description of *Actinoplanes ferrugineus*, comb. nov.

A. fer.ru.gi'ne.us (L. masc. adj. *ferrugineus*, rusty brown, referring to the orange-dark brown to clove brown color of the substrate mycelium).

Basonym: *Pseudosporangium ferrugineum* Ara et al. 2008

The description is as given for *Pseudosporangium ferrugineum* (Ara et al., 2008b) with the following modification. The G+C content of the type-strain genome is 72.5%, its approximate size 8.65 Mbp, its IMG deposit 2728369278. The type strain is 3-44-a(19) = JCM 14710 = DSM 45348.

#### Description of *Adlercreutzia caecicola*, comb. nov.

A. cae.ci'co.la (N.L. n. *caecum*, caecum; L. suff. -*cola*, inhabitant of; N.L. n. *caecicola*, caecum-dweller).

Basonym: *Parvibacter caecicola* Clavel et al. 2013

The description is as given for *Parvibacter caecicola* (Clavel et al., 2013). The type strain is NR06 = DSM 22242 = CCUG 57646.

#### Description of *Adlercreutzia caecimuris*, comb. nov.

A. cae.ci.mu'ris (N.L. n. *caecum*, caecum; L. n. *mus*/*muris*, mouse; N.L. gen. n. *caecimuris*, of the caecum of a mouse).

Basonym: *Enterorhabdus caecimuris* Clavel et al. 2010

The description is as given for *Enterorhabdus caecimuris* (Clavel et al., 2010). The type strain is B7 = CCUG 56815 = DSM 21839.

#### Description of *Adlercreutzia mucosicola*, comb. nov.

A. mu.co.si'co.la (N.L. n. *mucosa*, mucosa; L. suff. -*cola*, inhabitant of; N.L. n. *mucosicola*, inhabitant of the intestinal mucosa).

Basonym: *Enterorhabdus mucosicola* Clavel et al. 2009

The description is as given for *Enterorhabdus mucosicola* (Clavel et al., 2009). The type strain is Mt1B8 = CCUG 54980 = DSM 19490.

#### Description of *Adlercreutzia muris*, comb. nov.

A. mu'ris (L. gen. n. *muris*, of a mouse).

Basonym: *Enterorhabdus muris* Lagkouvardos et al. 2016

The description is as given for *Enterorhabdus muris* (Lagkouvardos et al., 2016). The type strain is WCA-131-CoC-2 = DSM 29508 = KCTC 15543.

#### Description of *Actinokineospora alba*, comb. nov.

A. al'ba (N.L. fem. adj. *alba*, white).

Basonym: *Alloactinosynnema album* Yuan et al. 2010

The description is as given for *Alloactinosynnema album* (Yuan et al., 2010) with the following addition. The G+C content of the type-strain genome is 69.7%, its approximate size 7.29 Mbp, its IMG deposit 2775506708. The type strain is 03-9939 = DSM 45114 = KCTC 19294 = CCM 7461.

#### Description of *Actinokineospora iranica*, comb. nov.

A. i.ra'ni.ca (N.L. fem. adj. *iranica*, of or belonging to Iran).

Basonym: *Alloactinosynnema iranicum* Nikou et al. 2014

The description is as given for *Alloactinosynnema iranicum* (Nikou et al., 2014). The type strain is Chem10 = IBRC-M 10403 = CECT 8209.

#### Description of *Aldersonia kunmingensis*, comb. nov.

A. kun.min.gen'sis (N.L. fem. adj. *kunmingensis*, pertaining to Kunming, a city in Yunnan province in south-west China).

Basonym: *Rhodococcus kunmingensis* Wang et al. 2008

The description is as given for *Rhodococcus kunmingensis* (Wang et al., 2008) with the following modification. The G+C content of the type-strain genome is 66.2%, its approximate size 5.62 Mbp, its GenBank deposit SAMN04357316. The type strain is DSM 45001 = JCM 15626 = KCTC 19149.

#### Description of *Amycolatopsis arida*, nom. nov.

A. a'ri.da (L. fem. adj. *arida*, referring to the isolation of the strain from arid soil).

Basonym: *Yuhushiella deserti* Mao et al. 2011 (The name *Amycolatopsis deserti* has already been published (Busarakam et al., 2016) hence a new epithet has been chosen to avoid homonyms).

The description is as given for *Yuhushiella deserti* (Mao et al., 2011) with the following modification and addition. The G+C content of the type-strain genome is 72.7%, its approximate size 5.96 Mbp, its GenBank deposit SAMN05421810. The type strain is RA45 = DSM 45648 = JCM 16584.

#### Description of *Bifidobacterium porcinum*, sp. nov.

B. por.ci'num (L. neut. adj. *porcinum*, of a hog).

The description is as given for *Bifidobacterium thermacidophilum* subsp. *porcinum* (Zhu et al., 2003) with the following addition. The G+C content of the type-strain genome is 60.2%, its approximate size 2.08 Mbp, its GenBank deposit SAMN02673456. The type strain is P3-14 = CGMCC 1.3009 = JCM 16945 = LMG 21689.

#### Description of *Boudabousia marimammalium*, comb. nov.

B. ma.ri.mam.ma'li.um (L. neut. n. *mare*, the sea; N.L. fem. gen. pl. n. *mammalium*, of mammals; N.L. fem. gen. pl. n. *marimammalium*, of marine mammals).

Basonym: *Actinomyces marimammalium* Hoyles et al. 2001

The description is as given for *Actinomyces marimammalium* (Hoyles et al., 2001b) with the following modification. The G+C content of the type-strain genome is 54.2%, its approximate size 1.84 Mbp, its GenBank deposit SAMN06006700. The type strain is M1749/98/1 = CCUG 41710 = CIP 106509 = DSM 15383.

#### Description of *Bowdenia nasicola*, comb. nov.

B. na.si'co.la (L. n. *nasus*, nose; L. suff. -*cola*, inhabitant of; N.L. n. *nasicola*, inhabitant of the nose, referring to the place of isolation of the type strain).

Basonym: *Actinomyces nasicola* Hall et al. 2003

The description is as given for *Actinomyces nasicola* (Hall et al., 2003c) with the following modification. The G+C content of the type-strain genome is 65.2%, its approximate size 2.64 Mbp, its GenBank deposit SAMN06092712. The type strain is R2014 = CCUG 46092 = CIP 107668.

#### Description of *Buchananella hordeovulneris*, comb. nov.

B. hor.de.o.vul'ne.ris (L. n. *hordeum*, barley; N.L. n. *Hordeum*, a grass genus; L. n. *vulnus/vulneris*, wound, injury; N.L. gen. n. *hordeovulneris*, isolated from injuries of awns of *Hordeum*).

Basonym: *Actinomyces hordeovulneris* Buchanan et al. 1984

The description is as given for *Actinomyces hordeovulneris* (Buchanan et al., 1984). The type strain is ATCC 35275 = CCUG 32937 = CIP 103149 = DSM 20732 = UCD 81-332-9.

#### Description of *Jongsikchunia kroppenstedtii*, comb. nov.

C. krop.pen.sted'ti.i (N.L. gen. n. *kroppenstedtii*, of Kroppenstedt, in honor of Reiner Michael Kroppenstedt, a German microbiologist, for his contributions to the taxonomy of *Actinobacteria*).

Basonym: *Gordonia kroppenstedtii* Kim et al. 2009

The description is as given for *Gordonia kroppenstedtii* (Kim et al., 2009) with the following modification. The G+C content of the type-strain genome is 67.1%, its approximate size 4.23 Mbp, its GenBank deposit SAMN02441055. The type strain is NP8-5 = DSM 45133 = JCM 16948 = KCTC 19360.

#### Description of *Clavibacter capsici*, sp. nov.

C. cap'si.ci (N.L. neut. gen. n. *capsici*, referring to *Capsicum*, the genus name of pepper).

The description is as given for *Clavibacter michiganensis* subsp. *capsici* (Oh et al., 2016). The type strain is PF008 = KACC 18448 = LMG 29047.

#### Description of *Clavibacter nebraskensis*, sp. nov.

C. ne.bras.ken'sis (N.L. masc. adj. *nebraskensis*, of or belonging to Nebraska).

The description is as given for *Clavibacter michiganensis* subsp. *nebraskensis* (Davis et al., 1984). The type strain is ATCC 27794 = CCUG 38894 = CIP 105362 = DSM 7483 = ICMP 3298 = JCM 9666 = LMG 3700 = LMG 5627 = LMG 7223 = NCPPB 2581 = VKM Ac-1404.

#### Description of *Clavibacter sepedonicus*, sp. nov.

C. se.pe.do'ni.cus (Gr. n. *sepedon*, rottenness, decay, putrefaction; L. neut. suff. -*icum*, suffix used with the sense of pertaining to; N.L. masc. adj. *sepedonicus*, pertaining to decay).

The description is as given for *Clavibacter michiganensis* subsp. *sepedonicus* (Davis et al., 1984) with the following modification.The G+C content of the type-strain genome is 72.4%, its approximate size 3.40 Mbp, its GenBank deposit SAMEA1705948. The type strain is ATCC 33113 = CCUG 23908 = CIP 104844 = CFBP 2049 = DSM 20744 = ICMP 2535 = JCM 9667 = LMG 2889 = NCPPB 2137 = VKM Ac-1405.

#### Description of *Cutibacterium namnetense*, comb. nov.

C. nam.ne.ten'se (N.L. neut. adj. *namnetense*, of Namnetum, the Latin name of Nantes).

Basonym: *Propionibacterium namnetense* Aubin et al. 2016

The description is as given for *Propionibacterium namnetense* (Aubin et al., 2016). The type strain is NTS 31307302 = DSM 29427 = CCUG 66358.

#### Description of *Demequina gelatinilytica*, comb. nov.

D. ge.la.ti.ni.ly'ti.ca (N.L. neut. n. *gelatinum*, gelatin; N.L. adj. *lyticus*, able to dissolve; N.L. fem. adj. *gelatinilytica*, gelatin-dissolving).

Basonym: *Lysinimicrobium gelatinilyticum* Hamada et al. 2015

The description is as given for *Lysinimicrobium gelatinilyticum* (Hamada et al., 2015). The type strain is HI12-44 = NBRC 109390 = DSM 28149.

#### Description of *Demequina iriomotensis*, comb. nov.

D. i.ri.o.mo.ten'sis (N.L. fem. adj. *iriomotensis*, pertaining to Iriomote Island, Japan, where the type strain was isolated).

Basonym: *Lysinimicrobium iriomotense* Hamada et al. 2015

The description is as given for *Lysinimicrobium iriomotense* (Hamada et al., 2015). The type strain is HI12-143 = NBRC 109399 = DSM 28146.

#### Description of *Demequina mangrovi*, comb. nov.

D. man.gro'vi (N.L. gen. n. *mangrovi*, of a mangrove).

Basonym: *Lysinimicrobium mangrovi* Hamada et al. 2012

The description is as given for *Lysinimicrobium mangrovi* (Hamada et al., 2012). The type strain is HI08-69 = NBRC 105856 = DSM 24868.

#### Description of *Demequina maris*, nom. nov.

D. ma'ris (L. gen. n. *maris*, of the sea).

Basonym: *Lysinimicrobium aestuarii* Hamada et al. 2015 (The name *Demequina aestuarii* has already been validly published (Yi et al., 2007) hence a new epithet has been chosen to avoid homonyms).

The description is as given for *Lysinimicrobium aestuarii* (Hamada et al., 2015). The type strain is HI12-104 = NBRC 109392 = DSM 28144.

#### Description of *Demequina pelophila*, comb. nov.

D. pe.lo'phi.la (Gr. n. *pelos*, mud; Gr. adj. *philos*, loving; N.L. fem. adj. *pelophila*, mud-loving).

Basonym: *Lysinimicrobium pelophilum* Hamada et al. 2015

The description is as given for *Lysinimicrobium pelophilum* (Hamada et al., 2015). The type strain is HI12-111 = NBRC 109393 = DSM 28148.

#### Description of *Demequina phytophila*, nom. nov.

D. phy.to'phi.la (N.L. n. *phyto*, plant; Gr. adj. *phila*, loving; N.L. fem. adj. *phytophila*, plant-loving).

Basonym: *Lysinimicrobium flavum* Hamada et al. 2015 (The name *Demequina flava* has already been validly published (Hamada et al., 2013) hence a new epithet has been chosen to avoid homonyms).

The description is as given for *Lysinimicrobium flavum* (Hamada et al., 2015). The type strain is HI12-45 = NBRC 109391 = DSM 28150.

#### Description of *Demequina rhizosphaerae*, comb. nov.

D. rhi.zos.phae'rae (Gr. neut. n. *rhiza*, root; L. n. *sphaera*, ball, sphere; N.L. gen. n. *rhizosphaerae*, of the rhizosphere).

Basonym: *Lysinimicrobium rhizosphaerae* Hamada et al. 2015

The description is as given for *Lysinimicrobium rhizosphaerae* (Hamada et al., 2015). The type strain is HI12-135 = NBRC 109397 = DSM 28152.

#### Description of *Demequina silvatica*, nom. nov.

D. sil.va'ti.ca (N.L. fem. adj. *silvatica*, of the forest).

Basonym: *Lysinimicrobium luteum* Hamada et al. 2015 (The name *Demequina lutea* has already been validly published (Finster et al., 2009) hence a new epithet has been chosen to avoid homonyms).

The description is as given for *Lysinimicrobium luteum* (Hamada et al., 2015). The type strain is HI12-123 = NBRC 109395 = DSM 28147.

#### Description of *Demequina soli*, comb. nov.

D. so'li (L. gen. n. *soli*, of soil).

Basonym: *Lysinimicrobium soli* Hamada et al. 2015

The description is as given for *Lysinimicrobium soli* (Hamada et al., 2015). The type strain is HI12-122 = NBRC 109394 = DSM 28151.

#### Description of *Demequina subtropica*, comb. nov.

D. sub.tro'pi.ca (L. pref. *sub*, under, below, slightly; L. neut. adj. *tropicum*, tropical; N.L. fem. adj. *subtropica*, subtropical, referring to the subtropical region).

Basonym: *Lysinimicrobium subtropicum* Hamada et al. 2015

The description is as given for *Lysinimicrobium subtropicum* (Hamada et al., 2015). The type strain is HI12-128 = NBRC 109396 = DSM 28145.

#### Description of *Embleya scabrispora*, comb. nov.

E. sca.bri'spo.ra (L. adj. *scaber*/-*bra/-brum*, scabby, rough; Gr. fem. n. *spora*, seed; N.L. fem. adj. *scabrispora*, referring to the rugose surface of the spores).

Basonym: *Streptomyces scabrisporus* Ping et al. 2004

The description is as given for *Streptomyces scabrisporus* (Ping et al., 2004). The type strain is KM-4927 = JCM 11712 = NBRC 100760 = NRRL B-24202 = DSM 41855.

#### Description of *Flavimobilis marinus*, comb. nov.

F. ma.ri'nus (L. masc. adj. *marinus*, pertaining to the sea).

Basonym: *Sanguibacter marinus* Huang et al. 2005

The description is as given for *Sanguibacter marinus* (Huang et al., 2005) with the following modification. The G+C content of the type-strain genome is 72.0%, its approximate size 2.90 Mbp, its GenBank deposit SAMN04488035. The type strain is 1-19 = CGMCC 1.3457 = JCM 12547 = DSM 19083.

#### Description of *Flavimobilis soli*, comb. nov.

F. so'li (L. gen. n. *soli*, of soil).

Basonym: *Sanguibacter soli* Kim et al. 2008

The description is as given for *Sanguibacter soli* (Kim et al., 2008) with the following modification. The G+C content of the type-strain genome is 71.9%, its approximate size 2.89 Mbp, its GenBank deposit SAMN04490250. The type strain is DCY22 = KCTC 13155 = JCM 14841.

#### Description of *Gleimia coleocanis*, comb. nov.

G. co.le.o.ca'nis (Gr. n. *koleos*, vagina; L. gen. n. *canis*, of the dog; N.L. gen. n. *coleocanis*, of the vagina of a dog).

Basonym: *Actinomyces coleocanis* Hoyles et al. 2002

The description is as given for *Actinomyces coleocanis* (Hoyles et al., 2002a) with the following addition. The G+C content of the type-strain genome is 49.6%, its approximate size 1.72 Mbp, its GenBank deposit SAMN00001853. The type strain is M343/98/2 = CCUG 41708 = CIP 106873 = DSM 15436.

#### Description of *Gleimia europaea*, comb. nov.

G. eu.ro.pae'a (L. fem. adj. *europaea*, of or belonging to Europe, European, referring to the fact that six different European laboratories contributed to the description of the species).

Basonym: *Actinomyces europaeus* Funke et al. 1997

The description is as given for *Actinomyces europaeus* (Funke et al., 1997a). The type strain is ATCC 700353 = CCUG 32789 A = CIP 105308 = LMG 18454.

#### Description of *Gleimia hominis*, comb. nov.

G. ho'mi.nis (L. n. homo/-inis, a human being, man, person; L. gen. n. *hominis*, of man, indicating that the type strain was isolated from a human).

Basonym: *Actinomyces hominis* Funke et al. 2010

The description is as given for *Actinomyces hominis* (Funke et al., 2010a). The type strain is 1094 = 7894GR = CCUG 57540 = DSM 22168.

#### Description of *Gulosibacter bifidus*, comb. nov.

G. bi'fi.dus (L. masc. adj. *bifidus*, divided into two parts).

Basonym: *Zimmermannella bifida* Lin et al. 2004

The description is as given for *Zimmermannella bifida* (Lin et al., 2004). The type strain is CCUG 50209 = JCM 12920 = NBRC 103089 = TISTR 1511 = DSM 17450.

#### Description of *Gulosibacter faecalis*, comb. nov.

G. fae.ca'lis (N.L. masc. adj. *faecalis*, faecal).

Basonym: *Zimmermannella faecalis* Lin et al. 2004

The description is as given for *Zimmermannella faecalis* (Lin et al., 2004) with the following modification. The G+C content of the type-strain genome is 68.5%, its approximate size 2.85 Mbp, its GenBank deposit SAMN02440490. The type strain is ATCC 13722 = CCUG 37873 = JCM 12866 = NBRC 15706 = TISTR 1514.

#### Description of *Haloechinothrix halophila*, comb. nov.

H. ha.lo'phi.la (Gr. n. *halos*, salt; Gr. adj. *philos*, loving; N.L. fem. adj. *halophila*, salt-loving, referring to the ability of the type strain to grow at high NaCl concentrations).

Basonym: *Amycolatopsis halophila* Tang et al. 2010

The description is as given for *Amycolatopsis halophila* (Tang et al., 2010a) with the following modification. The G+C content of the type-strain genome is 67.8%, its approximate size 5.55 Mbp, its GenBank deposit SAMN02597165. The type strain is DSM 45216 = JCM 18118 = KCTC 19403.

#### Description of *Haloechinothrix salitolerans*, comb. nov.

H. sa.li.to'le.rans (L. n. *sal*/*salis*, salt; L. part. adj. *tolerans*, tolerating, enduring; N.L. part. adj. *salitolerans*, salt tolerating).

Basonym: *Amycolatopsis salitolerans* Guan et al. 2012

The description is as given for *Amycolatopsis salitolerans* (Guan et al., 2012). The type strain is TRM F103 = CCTCC AB 208326 = JCM 15899 = DSM 45783.

#### Description of *Fannyhessea vaginae*, comb. nov.

H. va.gi'nae (L. n. *vagina*, sheath, vagina; L. gen. n. *vaginae*, of the vagina).

Basonym: *Atopobium vaginae* Rodriguez Jovita et al. 1999

The description is as given for *Atopobium vaginae* (Rodriguez Jovita et al., 1999) with the following modification. The G+C content of the type-strain genome is 42.7%, its approximate size 1.43 Mbp, its GenBank deposit SAMN00001474. The type strain is CCUG 38953 = ATCC BAA-55 = CIP 106431.

#### Description of *Intrasporangium flavum*, comb. nov.

I. fla'vum (L. neut. adj. *flavum*, yellow).

Basonym: *Monashia flava* Azman et al. 2016

The description is as given for *Monashia flava* (Azman et al., 2016). The type strain is MUSC 78 = DSM 29621 = MCCC 1K00454 = NBRC 110749.

#### Description of *Kitasatospora indigofera*, comb. nov.

K. in.di.go'fe.ra (N.L. n. *indigo*, the dye indigo; L. suff. -*fer/-fera/-ferum*, bearing; N.L. fem. adj. *indigofera*, bearing indigo, referring to production of blue to green diffusible pigments on chemically defined media).

Basonym: *Streptomyces indigoferus* Shinobu and Kawato 1960

The description is as given for *Streptomyces indigoferus* (Shinobu and Kawato, 1960). The type strain is ATCC 23924 = CBS 908.68 = BCRC (formerly CCRC) 13773 = DSM 40124 = IFO (now NBRC) 12878 = IFO (now NBRC) 3868 = JCM 4646 = NCIMB 9718 = NRRL B-3301.

#### Description of *Kitasatospora xanthocidica*, comb. nov.

K. xan.tho.ci'di.ca (Gr. adj. *xanthos*, yellow; L. v. *caedo*, to kill; N.L. fem. adj. *xanthocidica*, pertaining to yellow and to cut, probably referring to the name given the antibiotic produced which, in turn, was probably derived from its activity against *Xanthomonas oryzae*).

Basonym: *Streptomyces xanthocidicus* Asahi et al. 1966 (Approved Lists 1980)

The description is as given for *Streptomyces xanthocidicus* (Asahi et al., 1966). The type strain is CGMCC 4.1424 = ATCC 27480 = CBS 770.72 = BCRC (formerly CCRC) 11874 = DSM 40575 = IFO (now NBRC) 13469 = JCM 4243 = JCM 4862 = NRRL B-12504 = NRRL-ISP 5575 = VKM Ac-872.

#### Description of *Knoellia remsis*, comb. nov.

K. rem'sis (N.L. gen. n. *remsis*, of REMS, acronym of “Regenerative Enclosed Life Support Module Simulator”).

Basonym: *Tetrasphaera remsis* Osman et al. 2007

The description is as given for *Tetrasphaera remsis* (Osman et al., 2007) with the following modification. The G+C content of the type-strain genome is 70.7%, its approximate size 3.57 Mbp, its GenBank deposit SAMN05421826. The type strain is 3-M5-R-4 = ATCC BAA-1496 = CIP 109413 = JCM 15662.

#### Description of *Lancefieldella parvula*, comb. nov.

L. par'vu.la (L. fem. dim. adj. *parvula*, very small).

Basonym: *Atopobium parvulum* (Weinberg et al. 1937) Collins and Wallbanks 1993

The description is as given for *Atopobium parvulum* (Collins and Wallbanks, 1992) with the following modification. The G+C content of the type-strain genome is 45.7%, its approximate size 1.54 Mbp, its GenBank deposit SAMN00001907. The type strain is JCM 10300 = ATCC 33793 = DSM 20469.

#### Description of *Lancefieldella rimae*, comb. nov.

L. ri'mae (L. gen. n. *rimae*, of a fissure, pertaining to the gingival crevice).

Basonym: *Atopobium rimae* (Olsen et al. 1991) Collins and Wallbanks 1993

The description is as given for *Atopobium rimae* (Collins and Wallbanks, 1992) with the following modification. The G+C content of the type-strain genome is 49.3%, its approximate size 1.63 Mbp, its GenBank deposit SAMN00000717. The type strain is ATCC 49626 = CCUG 31168 = DSM 7090 = IFO (now NBRC) 15546 = JCM 10299 = LMG 11476.

#### Description of *Lentzea aerocolonigenes*, comb. nov.

L. ae.ro.co.lo.ni'ge.nes (Gr. n. *aer*, air; L. n. *colonia*, a colony; N.L. suff. -*genes*, producing; N.L. fem. adj. *aerocolonigenes*, producing aerial colonies).

Basonym: *Lechevalieria aerocolonigenes* (Labeda, 1986) Labeda et al. 2001

The description is as given for *Lechevalieria aerocolonigenes* (Labeda, 1986; Labeda et al., 2001) with the following modification. The G+C content of the type-strain genome is 68.9%, its approximate size 10.80 Mbp, its GenBank deposit SAMN05661031. The type strain is Shinobu 701 = ATCC 23870 = BCRC (formerly CCRC) 13661 = CIP 107109 = DSM 40034 = IFO (now NBRC) 13195 = ISP 5034 = JCM 4150 = JCM 4614 = NRRL B-3298 = NRRL ISP-5034 = VKM Ac-1081.

#### Description of *Lentzea atacamensis*, comb. nov.

L. a.ta.ca.men'sis (N.L. fem. adj. *atacamensis*, of or pertaining to the Atacama Desert of northern Chile, the source of the soil from which the type strain was isolated).

Basonym: *Lechevalieria atacamensis* Okoro et al. 2010

The description is as given for *Lechevalieria atacamensis* (Okoro et al., 2010) with the following addition. The G+C content of the type-strain genome is 68.9%, its approximate size 9.31 Mbp, its IMG deposit 2756170238. The type strain is C61 = CGMCC 4.5536 = JCM 17492 = NRRL B-24706 = DSM 45479.

#### Description of *Lentzea deserti*, comb. nov.

L. de.ser'ti (L. gen. n. *deserti*, of a desert).

Basonym: *Lechevalieria deserti* Okoro et al. 2010

The description is as given for *Lechevalieria deserti* (Okoro et al., 2010) with the following addition. The G+C content of the type-strain genome is 68.8%, its approximate size 9.53 Mbp, its IMG deposit 2756170278. The type strain is C68 = CGMCC 4.5535 = JCM 17493 = NRRL B-24707 = DSM 45480.

#### Description of *Lentzea flava*, comb. nov.

L. fla'va (L. fem. adj. *flava*, yellow, referring to the color of the substrate mycelium).

Basonym: *Lechevalieria flava* (Gauze et al. 1974) Labeda et al. 2001

The description is as given for *Lechevalieria flava* (Labeda et al., 2001) with the following addition. The G+C content of the type-strain genome is 69.0%, its approximate size 9.75 Mbp, its GenBank deposit SAMN05660974. The type strain is ATCC 29533 = BCRC (formerly CCRC) 13328 = CIP 107110 = DSM 43885 = IFO (now NBRC) 14521 = JCM 3296 = NRRL B-16131 = VKM Ac-906.

#### Description of *Lentzea fradiae*, comb. nov.

L. fra'di.ae (N.L. gen. n. *fradiae*, of Fradia, a patronymic).

Basonym: *Lechevalieria fradiae* Zhang et al. 2007

The description is as given for *Lechevalieria fradiae* (Zhang et al., 2007b) with the following modification. The G+C content of the type-strain genome is 70.5%, its approximate size 8.51 Mbp, its GenBank deposit SAMN05216553. The type strain is Z6 = CGMCC 4.3506 = JCM 14205 = DSM 45099.

#### Description of *Lentzea nigeriaca*, comb. nov.

L. ni.ge.ri'a.ca (N.L. fem. adj. *nigeriaca*, of or belonging to Nigeria).

Basonym: *Lechevalieria nigeriaca* Camas et al. 2013

The description is as given for *Lechevalieria nigeriaca* (Camas et al., 2013). The type strain is NJ2035 = DSM 45680 = KCTC 29057 = NRRL B-24881 = JCM 31207.

#### Description of *Lentzea roselyniae*, comb. nov.

L. ro.se.ly'ni.ae (N.L. fem. gen. n. *roselyniae*, of Roselyn, named after Roselyn Brown for her many practical contributions to actinomycete systematics).

Basonym: *Lechevalieria roselyniae* Okoro et al. 2010

The description is as given for *Lechevalieria roselyniae* (Okoro et al., 2010). The type strain is C81 = CGMCC 4.5537 = JCM 17494 = NRRL B-24708 = DSM 45481.

#### Description of *Lentzea xinjiangensis*, comb. nov.

L. xin.ji.an.gen'sis (N.L. fem. adj. *xinjiangensis*, referring to Xinjiang, north-western China, the source of the isolate).

Basonym: *Lechevalieria xinjiangensis* Wang et al. 2007

The description is as given for *Lechevalieria xinjiangensis* (Wang et al., 2007a) with the following modification. The G+C content of the type-strain genome is 70.7%, its approximate size 8.68 Mbp, its GenBank deposit SAMN05216188. The type strain is R24 = CGMCC 4.3525 = DSM 45081 = JCM 15473.

#### Description of *Leucobacter japonicus*, sp. nov.

L. ja.po'ni.cus (N.L. masc. adj. *japonicus*, Japanese).

The description is as given for *Leucobacter musarum* subsp. *japonicus* (Clark and Hodgkin, 2015) with the following addition. The G+C content of the type-strain genome is 66.8%, its approximate size 3.59 Mbp, its GenBank deposit SAMN02673066. The type strain is CBX130 = DSM 27158 = CIP 110719 = JCM 31936.

#### Description of *Microlunatus aerolatus*, comb. nov.

M. ae.ro.la'tus (Gr. n. *aer*, air; L. part. adj. *latus*, carried; N.L. part. adj. *aerolatus*, airborne).

Basonym: *Friedmanniella aerolata* Kim et al. 2016

The description is as given for *Friedmanniella aerolata* (Kim et al., 2016). The type strain is 7515T-26 = KACC 17306 = DSM 27139.

#### Description of *Microlunatus antarcticus*, comb. nov.

M. an.tarc'ti.cus (M.L. masc. adj. *antarcticus*, isolated from Antarctica).

Basonym: *Friedmanniella antarctica* Schumann et al. 1997

The description is as given for *Friedmanniella antarctica* (Schumann et al., 1997). The type strain is AA-1042 = NBRC 16127 = DSM 11053 = KACC 14501.

#### Description of *Microlunatus capsulatus*, comb. nov.

M. cap.su.la'tus (L. masc. adj. *capsulatus*, capsulated).

Basonym: *Friedmanniella capsulata* Maszenan et al. 1999

The description is as given for *Friedmanniella capsulata* (Maszenan et al., 1999a). The type strain is Ben 108 = ACM 5120 = JCM 13522 = KACC 14261.

#### Description of *Microlunatus flavus*, comb. nov.

M. fla'vus (L. masc. adj. *flavus*, yellow, pertaining to the yellow color of the colonies).

Basonym: *Friedmanniella flava* Zhang et al. 2013

The description is as given for *Friedmanniella flava* (Zhang et al., 2013b) with the following modification. The G+C content of the type-strain genome is 73.5%, its approximate size 4.79 Mbp, its GenBank deposit SAMN05421756. The type strain is W6 = CGMCC 4.6856 = JCM 17701 = KACC 17380.

#### Description of *Microlunatus lacustris*, comb. nov.

M. la.cus'tris (L. masc. adj. *lacustris*, belonging to the lake).

Basonym: *Friedmanniella lacustris* Lawson et al. 2000

The description is as given for *Friedmanniella lacustris* (Lawson et al., 2000). The type strain is EL-17A = DSM 11465 = ATCC BAA-165 = KACC 15021.

#### Description of *Microlunatus lucidus*, comb. nov.

M. lu'ci.dus (L. masc. adj. *lucidus*, brilliant, shining, referring to the shiny colony surface).

Basonym: *Friedmanniella lucida* Iwai et al. 2010

The description is as given for *Friedmanniella lucida* (Iwai et al., 2010). The type strain is FA2 = DSM 21742 = NBRC 104964 = KACC 15053.

#### Description of *Microlunatus luteolus*, comb.nov.

M. lu.te'o.lus (L. masc. adj. *luteolus*, referring to the yellow color of the colonies).

Basonym: *Friedmanniella luteola* Iwai et al. 2010

The description is as given for *Friedmanniella luteola* (Iwai et al., 2010) with the following modification. The G+C content of the type-strain genome is 73.4%, its approximate size 4.41 Mbp, its GenBank deposit SAMN04488544. The type strain is FA1 = DSM 21741 = NBRC 104963 = KACC 15054.

#### Description of *Microlunatus okinawensis*, comb. nov.

M. o.ki.na.wen'sis (N.L. masc. adj. *okinawensis*, pertaining to Okinawa, a prefecture in Japan, where the organism was first isolated).

Basonym: *Friedmanniella okinawensis* Iwai et al. 2010

The description is as given for *Friedmanniella okinawensis* (Iwai et al., 2010). The type strain is FB1 = DSM 21744 = NBRC 104966 = KACC 15055.

#### Description of *Microlunatus sagamiharensis*, comb. nov.

M. sa.ga.mi.ha.ren'sis (N.L. masc. adj. *sagamiharensis*, pertaining to Sagamihara, a city in Japan, where the organism was first isolated).

Basonym: *Friedmanniella sagamiharensis* Iwai et al. 2010

The description is as given for *Friedmanniella sagamiharensis* (Iwai et al., 2010) with the following modification. The G+C content of the type-strain genome is 73.4%, its approximate size 4.41 Mbp, its GenBank deposit SAMN04488544. The type strain is FB2 = DSM 21743 = NBRC 104965 = KACC 15056.

#### Description of *Microlunatus spumicolus*, comb. nov.

M. spu.mi'co.lus (L. fem. n. *spuma*, foam; L. suff. -*colus*, inhabitant of; N.L. n. *spumicolus*, inhabitant of foam).

Basonym: *Friedmanniella spumicola* Maszenan et al. 1999

The description is as given for *Friedmanniella spumicola* (Maszenan et al., 1999a). The type strain is Ben 107 = ACM 5121 = JCM 16540 = KACC 14247.

#### Description of *Micromonospora andamanensis*, comb. nov.

M. an.da.ma.nen'sis (N.L. fem. adj. *andamanensis*, referring to the Andaman Sea, Thailand, the source of the type strain).

Basonym: *Verrucosispora andamanensis* Supong et al. 2013

The description is as given for *Verrucosispora andamanensis* (Supong et al., 2013). The type strain is SP03-05 = BCC 45620 = NBRC 109075 = DSM 46721.

#### Description of *Micromonospora fiedleri*, comb. nov.

M. fie'dle.ri (N.L. fem. gen. n. *fiedleri*, after Hans-Peter Fiedler in recognition of his contributions to the search and discovery of new antibiotics from *Actinobacteria*).

Basonym: *Verrucosispora fiedleri* Goodfellow et al. 2013

The description is as given for *Verrucosispora fiedleri* (Goodfellow et al., 2013). The type strain is MG-37 = NCIMB 14794 = NRRL-B-24892 = DSM 46741 = KACC 18210.

#### Description of *Micromonospora gifhornensis*, comb. nov.

M. gif.hor.nen'sis (N.L. fem. n. *gifhornensis*, referring to the city of Gifhorn, adjacent to the peat bog from which the organisms was isolated).

Basonym: *Verrucosispora gifhornensis* Rheims et al. 1998

The description is as given for *Verrucosispora gifhornensis* (Rheims et al., 1998). The type strain is HR1-2 = DSM 44337 = NBRC 16317 = JCM 10457 = KACC 20946.

#### Description of *Micromonospora lutea*, comb. nov.

M. lu'te.a (L. fem. adj. *lutea*, orange colored).

Basonym: *Verrucosispora lutea* Liao et al. 2009

The description is as given for *Verrucosispora lutea* (Liao et al., 2009). The type strain is CCTCC AA207012 = JCM 16959 = KCTC 19195 = DSM 45424.

#### Description of *Micromonospora maris*, comb. nov.

M. ma'ris (L. gen. n. *maris*, of the sea).

Basonym: *Verrucosispora maris* Goodfellow et al. 2012

The description is as given for *Verrucosispora maris* (Goodfellow et al., 2012b). The type strain is AB-18-032 = DSM 45365 = NRRL B-24793 = JCM 31040.

#### Description of *Micromonospora phaseoli*, comb. nov.

M. pha.se.o'li (N.L. masc. n. *Phaseolus*, a genus of plants; N.L. gen. n. *phaseoli*, of *Phaseolus*, referring to the isolation of the first strains from *Phaseolus vulgaris*).

Basonym: *Xiangella phaseoli* Wang et al. 2013

The description is as given for *Xiangella phaseoli* (Wang et al., 2013a) with the following modification. Major polar lipids are PE, diPE, PI and PG. The type strain is NEAU-J5 = CGMCC 4.7038 = DSM 45730 = NBRC 110907.

#### Description of *Micromonospora qiuiae*, comb. nov.

M. qi.u.i'ae (N.L. fem. gen. n. *qiuiae*, of Qiu, in honor of Danheng Qiu, for her devotion to the investigation of *Micromonospora*-like *Actinobacteria*).

Basonym: *Verrucosispora qiuiae* Xi et al. 2012

The description is as given for *Verrucosispora qiuiae* (Xi et al., 2012). The type strain is RtIII47 = CGMCC 4.5826 = NBRC 106684 = DSM 45781 = JCM 19682.

#### Description of *Micromonospora sediminimaris*, nom. nov.

M. se.di.mi.ni.ma'ris (L. n. *sedimen*/-*inis*, sediment; L. n. *mare*/-is, the sea; N.L. gen. n. *sediminimaris*, from the marine sediment).

Basonym: V*errucosispora sediminis* Dai et al. 2010 (The name *Micromonospora sediminis* has already been validly published (Muangham et al., 2016), hence a new epithet has been chosen to avoid homonyms).

The description is as given for *Verrucosispora sediminis* (Dai et al., 2010) with the following modification. The G+C content of the type-strain genome is 71.0%, its approximate size 6.40 Mbp, its GenBank deposit SAMN05216284. The type strain is MS426 = CGMCC 4.3550 = JCM 15670 = DSM 45558.

#### Description of *Micromonospora trujilloniae*, nom. nov.

M. tru.jil.lo'ni.a.e (N.L. gen. n. *trujilloniae*, after Martha E. Trujillo in recognition of her contributions to the discovery of new *Micromonospora* strains).

Basonym: V*errucosispora wenchangensis* Xie et al. 2012 (The name *Micromonospora wenchangensis* has already been validly published (Ren et al., 2013) hence a new epithet has been chosen to avoid homonyms).

The description is as given for *Verrucosispora wenchangensis* (Xie et al., 2012). The type strain is 234402 = CCTCC AA 2011018 = DSM 45674 = JCM 31041.

#### Description of *Mobiluncus holmesii*, sp. nov.

M. hol.me'si.i (N.L. gen. n. *holmesii*, named after K. K. Holmes, a researcher of sexually transmitted diseases).

The description is as given for *Mobiluncus curtisii* subsp. *holmesii* (Spiegel and Roberts, 1984) with the following addition. The G+C content of the type-strain genome is 55.6%, its approximate size 2.08 Mbp, its GenBank deposit SAMN00253295. The type strain is BV376-6 = ATCC 35242 = CCUG 17762 = LMG 7786 = NCTC 11657.

#### Description of *Pauljensenia hongkongensis*, comb. nov.

P. hong.kon.gen'sis (N.L. fem. adj. *hongkongensis*, of or belonging to Hong Kong, where the type strain was isolated).

Basonym: *Actinomyces hongkongensis* Woo et al. 2004

The description is as given for *Actinomyces hongkongensis* (Woo et al., 2003). The type strain is HKU8 = CCUG 48484 = DSM 15629 = CIP 107949 = LMG 21939.

#### Description of *Phycicoccus duodecadis*, comb. nov.

P. du.o.de'ca.dis (L. n. *duodecas*/-*adis*, the number twelve; L. gen. n. *duodecadis*, of twelve, referring to the requirement of the organism for vitamin B12).

Basonym: *Tetrasphaera duodecadis* (Lochhead 1958) Ishikawa and Yokota 2006

The description is as given for *Tetrasphaera duodecadis* (Ishikawa and Yokota, 2006). The type strain is ATCC 13347 = NCIMB 9222 = NBRC 12959.

#### Description of *Phycicoccus elongatus*, comb. nov.

P. e.lon.ga'tus (L. masc. adj. *elongatus*, elongated, pertaining to the formation of elongated clumps).

Basonym: *Tetrasphaera elongata* Hanada et al. 2002

The description is as given for *Tetrasphaera elongata* (Hanada et al., 2002a). The type strain is Lp2 = DSM 14184 = JCM 11141.

#### Description of *Rothia halotolerans*, comb. nov.

R. ha.lo.to'le.rans (Gr. n. *halos*, salt; L. part. adj. *tolerans*, tolerating, enduring; N.L. part. adj. *halotolerans*, salt-tolerating).

Basonym: *Kocuria halotolerans* Zucchi et al. 2009

The description is as given for *Kocuria halotolerans* (Tang et al., 2009). The type strain is CCTCC AB 206069 = DSM 18442 = KCTC 19172 = JCM 31975.

#### Description of *Rothia koreensis*, comb. nov.

R. ko.re.en'sis (N.L. fem. adj. *koreensis*, pertaining to Korea).

Basonym: *Kocuria koreensis* Park et al. 2010

The description is as given for *Kocuria koreensis* (Park et al., 2010). The type strain is P31 = JCM 15915 = KCTC 19595 = DSM 23367.

#### Description of *Rothia kristinae*, comb. nov.

R. kris.ti'nae (M.L. gen. n. *kristinae*, of Kristin).

Basonym: *Kocuria kristinae* (Kloos et al., 1974) Stackebrandt et al. 1995

The description is as given for *Kocuria kristinae* (Kloos et al., 1974; Stackebrandt et al., 1995) with the following modification. The G+C content of the type-strain genome is 71.9%, its approximate size 2.36 Mbp, its GenBank deposit SAMD00046484. The type strain is ATCC 27570 = CCM 2690 = CCUG 33026 = CIP 81.69 = DSM 20032 = IEGM 390 = IFO (now NBRC) 15354 = JCM 7237 = LMG 14215 = NCTC 11038 = NRRL B-14835 = VKM B-1811.

#### Description of *Saccharomonospora iraqiensis*, comb. nov.

S. i.ra.qi.en'sis (N.L. fem. adj. *iraqiensis*, of or belonging to Iraq, the source of the soil sample from which the organism was isolated).

Basonym: *Actinopolyspora iraqiensis* Ruan et al. 1994

The description is as given for *Actinopolyspora iraqiensis* (Ruan et al., 1994) with the following addition. The G+C content of the type-strain genome is 71.5%, its approximate size 3.84 Mbp, its GenBank deposit SAMN02441411. The type strain is IQ-H1 = DSM 44640 = JCM 9891 = NBRC 103187.

#### Description of *Saccharopolyspora kobensis*, sp. nov.

S. ko.ben'sis (N.L. fem. adj. *kobensis*, pertaining to Kobe, a city in Japan where the organism was isolated).

The description is as given for *Saccharopolyspora hirsuta* subsp. *kobensis* (Lacey, 1989) with the following addition. The G+C content of the type-strain genome is 70.9%, its approximate size 7.74 Mbp, its GenBank deposit SAMN02982929. The type strain is ATCC 20501 = DSM 44795 = FERM-P 3912 = NBRC 15151 = JCM 9109.

#### Description of *Schaalia canis*, comb. nov.

S. ca'nis (L. gen. n. *canis*, of the dog).

Basonym: *Actinomyces canis* Hoyles et al. 2000

The description is as given for *Actinomyces canis* (Hoyles et al., 2000). The type strain is M2289/98/2 = CCUG 41706 = DSM 15536.

#### Description of *Schaalia cardiffensis*, comb. nov.

S. car.diff.en'sis (L. fem. adj. *cardiffensis*, pertaining to Cardiff, a city in Wales).

Basonym: *Actinomyces cardiffensis* Hall et al. 2003

The description is as given for *Actinomyces cardiffensis* (Hall et al., 2002). The type strain is R10394 = CCUG 44997 = CIP 107323.

#### Description of *Schaalia funkei*, comb. nov.

S. fun'ke.i (N.L. gen. n. *funkei*, of Funke, to honor the microbiologist Guido Funke).

Basonym: *Actinomyces funkei* Lawson et al. 2001

The description is as given for *Actinomyces funkei* (Lawson et al., 2001). The type strain is CCUG 42773 = CIP 106713.

#### Description of *Schaalia georgiae*, comb. nov.

S. ge.or'gi.ae (N.L. gen. n. *georgiae*, of Georg, named in honor of Lucile Georg, a pioneer in *Actinomyces* taxonomy).

Basonym: *Actinomyces georgiae* Johnson et al. 1990

The description is as given for *Actinomyces georgiae* (Johnson et al., 1990) with the following restriction. The G+C content of the type-strain genome is 69.9%, its approximate size 2.50 Mbp, its GenBank deposit SAMN02441198. The type strain is ATCC 49285 = CCUG 32935 = CIP 104749 = DSM 6843.

#### Description of *Schaalia hyovaginalis*, comb. nov.

S. hy.o.va.gi.na'lis (Gr. n. *hys*, pig; L. n. *vagina*, sheath, vagina; N.L. fem. adj. *hyovaginalis*, pertaining to the vagina of a pig).

Basonym: *Actinomyces hyovaginalis* Collins et al. 1993

The description is as given for *Actinomyces hyovaginalis* (Collins et al., 1993). The type strain is BM 1192/5 = ATCC 51367 = CCUG 35604 = CCUG 35715 = CIP 103923 = DSM 10695 = NCIMB 702983.

#### Description of *Schaalia meyeri*, comb. nov.

S. me'ye.ri (N.L. gen. n. *meyeri*, of Meyer, to honor Kurt F. Meyer, a German bacteriologist, who described the organism as a “new anaerobic *Streptothrix* species” in 1911).

Basonym: *Actinomyces meyeri* (ex Prévot 1938) Cato et al. 1984

The description is as given for *Actinomyces meyeri* (Cato et al., 1984) with the following modification. The G+C content of the type-strain genome is 65.6%, its approximate size 2.03 Mbp, its GenBank deposit SAMN04489715. The type strain is ATCC 35568 = CCUG 21024 = CIP 103148 = DSM 20733 = LMG 16161.

#### Description of *Schaalia naturae*, comb. nov.

S. na.tu'rae (L. gen. n. *naturae*, of nature, intended to mean from the environment).

Basonym: *Actinomyces naturae* Rao et al. 2012

The description is as given for *Actinomyces naturae* (Rao et al., 2012). The type strain is BL-79 = CCUG 56698 = NRRL B-24670 = DSM 26713.

#### Description of *Schaalia odontolytica*, comb. nov.

S. o.don.to.ly'ti.ca (Gr. n. *odous*/-*ontos*, tooth; N.L. fem. adj. *lytica*, able to dissolve; N.L. fem. adj. *odontolytica*, tooth-dissolving).

Basonym: *Actinomyces odontolyticus* Batty 1958 (Approved Lists 1980)

The description is as given for *Actinomyces odontolyticus* (Batty, 1958). The type strain is ATCC 17929 = CCUG 20536 = CIP 101124 = DSM 43760 = JCM 14871 = LMG 18080 = NCTC 9935.

#### Description of *Schaalia radingae*, comb. nov.

S. ra.din'gae (M.L. masc. n. *Radinga*, the Latin name for Reading, UK; M.L. gen. n. *radingae*, of Reading).

Basonym: *Actinomyces radingae* Wüst et al. 1995

The description is as given for *Actinomyces radingae* (Wüst et al., 1995; Vandamme et al., 1998) with the following modification. The G+C content of the type-strain genome is 58.9%, its approximate size 2.43 Mbp, its GenBank deposit SAMN04489714. The type strain is APL1 = ATCC 51856 = CCUG 32394 = CCUG 34270 = CIP 105358 = DSM 9169 = LMG 15960.

#### Description of *Schaalia suimastitidis*, comb. nov.

S. su.i.mas.ti'ti.dis (N.L. n. *sus*, pig; M.L. n. *mastitis*, infection of the milk glands; N.L. gen. n. *suimastitidis*, of porcine mastitis).

Basonym: *Actinomyces suimastitidis* Hoyles et al. 2001

The description is as given for *Actinomyces suimastitidis* (Hoyles et al., 2001a) with the following addition. The G+C content of the type-strain genome is 56.4%, its approximate size 2.29 Mbp, its GenBank deposit SAMN02440611. The type strain is CCUG 39276 = CIP 106779.

#### Description of *Schaalia turicensis*, comb. nov.

S. tu.ri.cen'sis (L. fem. adj. *turicensis*, of Turicum, the Latin name for Zürich, referring to the place where the type strain had been isolated).

Basonym: *Actinomyces turicensis* Wüst et al. 1995

The description is as given for *Actinomyces turicensis* (Wüst et al., 1995; Vandamme et al., 1998). The type strain is APL10 = ATCC 51857 = CCUG 32401 = CCUG 34269 = CIP 105357 = DSM 9168 = LMG 15961.

#### Description of *Schaalia vaccimaxillae*, comb. nov.

S. vac.ci.ma.xil'lae (L. n. *vacca*, cow; L. n. *maxilla*, jaw; N.L. gen. n. *vaccimaxillae*, of the cow's jaw).

Basonym: *Actinomyces vaccimaxillae* Hall et al. 2003

The description is as given for *Actinomyces vaccimaxillae* (Hall et al., 2003b) with the following addition. The G+C content of the type-strain genome is 57.6%, its approximate size 2.34 Mbp, its GenBank deposit SAMN02441246. The type strain is R10176 = CCUG 46091 = CIP 107423.

#### Description of *Pedococcus aerophilus*, comb. nov.

S. ae.ro'phi.lus (Gr. n. *aer*, air; Gr. adj. *philos*, loving; N.L. masc. adj. *aerophilus*, air-loving).

Basonym: *Phycicoccus aerophilus* Weon et al. 2008

The description is as given for *Phycicoccus aerophilus* (Weon et al., 2008). The type strain is 5516T-20 = KACC 20658 = DSM 18548 = JCM 16378.

#### Description of *Pedococcus badiiscoriae*, comb. nov.

S. ba.di.i.sco.ri'ae (L. adj. *badius*, brown; L. gen. n. *scoriae*, of scoria, i.e., volvanic ash; N.L. gen. n. *badiiscoriae*, of brown-colored scoria).

Basonym: *Phycicoccus badiiscoriae* Lee 2013

The description is as given for *Phycicoccus badiiscoriae* (Lee, 2013a). The type strain is Sco-B23 = KCTC 19807 = KACC 15111 = DSM 23987 = NBRC 107918.

#### Description of *Pedococcus bigeumensis*, comb. nov.

S. bi.geu.men'sis (N.L. masc. adj. *bigeumensis*, pertaining to Bigeum Island, Korea, from where the type strain was isolated).

Basonym: *Phycicoccus bigeumensis* Dastager et al. 2008

The description is as given for *Phycicoccus bigeumensis* (Dastager et al., 2008). The type strain is MSL-03 = DSM 19264 = KCTC 19266 = JCM 16023.

#### Description of *Pedococcus cremeus*, comb. nov.

S. cre.me'us (N.L. masc. adj. *cremeus*, cream-white).

Basonym: *Phycicoccus cremeus* Zhang et al. 2011

The description is as given for *Phycicoccus cremeus* (Zhang et al., 2011). The type strain is V2M29 = CGMCC 1.6963 = NBRC 104261 = DSM 28108 = JCM 17739.

#### Description of *Pedococcus dokdonensis*, comb. nov.

S. dok.do.nen'sis (N.L. masc. adj. *dokdonensis*, of Dokdo, Korea, from where the type strain was isolated).

Basonym: *Phycicoccus dokdonensis* Yoon et al. 2008

The description is as given for *Phycicoccus dokdonensis* (Yoon et al., 2008). The type strain is DS-8 = KCTC 19248 = CCUG 54521 = DSM 22329 = JCM 19120.

#### Description of *Pedococcus ginsenosidimutans*, comb. nov.

S. gin.se.no.si.di.mu'tans (N.L. n. *ginsenosidum*, ginsenoside; L. part. adj. *mutans*, transforming, converting; N.L. part. adj. *ginsenosidimutans*, ginsenoside-converting).

Basonym: *Phycicoccus ginsenosidimutans* Wang et al. 2011

The description is as given for *Phycicoccus ginsenosidimutans* (Wang et al., 2011). The type strain is BXN5-13 = DSM 21006 = KCTC 19419 = JCM 18961.

#### Description of *Pedococcus soli*, comb. nov.

S. so'li (L. gen. n. *soli*, of soil).

Basonym: *Phycicoccus soli* Singh et al. 2015

The description is as given for *Phycicoccus soli* (Singh et al., 2015). The type strain is THG-a14 = KACC 17892 = JCM 19837 = DSM 103360.

#### Description of *Thermomonospora amylolytica*, comb. nov.

T. a.my.lo.ly'ti.ca (Gr. n. *amylon*, starch; Gr. adj. *lytikos*, dissolving; N.L. fem. adj. *amylolytica*, starch dissolving).

Basonym: *Actinomadura amylolytica* Jiao et al. 2015

The description is as given for *Actinomadura amylolytica* (Jiao et al., 2015). The type strain is CCTCC AA 2012024 = DSM 45822 = JCM 30324.

#### Description of *Thermomonospora cellulosilytica*, comb. nov.

T. cel.lu.lo.si.ly'ti.ca (N.L. n. *cellulosum*, cellulose; N.L. adj. *lyticus*, able to dissolve; N.L. fem. adj. *cellulosilytica*, cellulose-dissolving).

Basonym: *Actinomadura cellulosilytica* Jiao et al. 2015

The description is as given for *Actinomadura cellulosilytica* (Jiao et al., 2015). The type strain is CCTCC AA 2012023 = DSM 45823 = JCM 30326.

#### Description of *Thermomonospora echinospora*, comb. nov.

T. e.chi.no'spo.ra (L. n. *echinus*, sea urchin; Gr. fem. n. *spora*, seed; N.L. fem. n. *echinospora*, spiny spore).

Basonym: *Actinomadura echinospora* (Nonomura and Ohara 1971) Kroppenstedt et al. 1991

The description is as given for *Actinomadura echinospora* (Nonomura and Ohara, 1971a; Kroppenstedt et al., 1990) with the following addition. The G+C content of the type-strain genome is 71.8%, its approximate size 8.64 Mbp, its GenBank deposit SAMN04489712. The type strain is ATCC 27300 = CCRC 12547 = DSM 43163 = HUT 6548 = NBRC 14042 = JCM 3148 = KCTC 9313.

#### Description of *Thermomonospora umbrina*, comb. nov.

T. um.bri'na (N.L. fem. adj. *umbrina*, wood brown).

Basonym: *Actinomadura umbrina* Galatenko et al. 1987

The description is as given for *Actinomadura umbrina* (Galatenko et al., 1981) with the following addition. The G+C content of the type-strain genome is 71.9%, its approximate size 7.40 Mbp, its IMG deposit 2778260945. The type strain is ATCC 49502 = CIP 105485 = DSM 43927 = IFO (now NBRC) 14346 = INA 2309 = JCM 6837 = NRRL B-16244 = VKM Ac-1086.

#### Description of *Winkia neuii*, comb. nov.

W. neu'i.i (N.L. gen. n. *neuii*, of Neu, named to honor Harols Neu, an authority in antimicrobial chemotherapy and infectious diseases).

Basonym: *Actinomyces neuii* Funke et al. 1994

The description is as given for *Actinomyces neuii* (Funke et al., 1994b) with the following restriction. The G+C content of the type-strain genome is 56.2%, its approximate size 2.27 Mbp, its GenBank deposit SAMN02441245. The type strain is 97/90 = ATCC 51847 = CCUG 32252 = CIP 104015 = DSM 8576.

#### Description of *Yinghuangia aomiensis*, comb. nov.

Y. a.o.mi.en'sis (N.L. fem. adj. *aomiensis*, of or pertaining to Aomi, the place from where the strain was isolated).

Basonym: *Streptomyces aomiensis* Nagai et al. 2011

The description is as given for *Streptomyces aomiensis* (Nagai et al., 2011). The type strain is M24DS4 = M24DS04 = JCM 17986 = KACC 14925 = NBRC 106164.

#### Description of *Adlercreutzia equolifaciens* subsp. *Celatus*, subsp. nov.

A. e.quo.li.fa'ci.ens subsp. ce.la'tus (L. masc. adj. *celatus*, conceal, hide, keep secret).

The description is as given for *Asaccharobacter celatus* (Minamida et al., 2008). The type strain is do03 = AHU 1763 = DSM 18785 = JCM 14811.

#### Description of *Bifidobacterium catenulatum* subsp. *Kashiwanohense*, subsp. nov.

B. ca.te.nu.la'tum subsp. ka.shi.wa.no.hen'se (N.L. neut. adj. *kashiwanohense*, of or pertaining to Kashiwanoha in Japan).

The description is as given for *Bifidobacterium kashiwanohense* (Morita et al., 2011) with the following restriction. The G+C content of the type-strain genome is 56.3%, its approximate size 2.34 Mbp, its GenBank deposit SAMD00061044. The type strain is HM2-2 = DSM 21854 = JCM 15439.

#### Description of *Bifidobacterium pullorum* subsp. *Gallinarum*, subsp. nov.

B. pul.lo'rum subsp. gal.li.na'rum (L. n. *gallina*, a hen; L. gen. pl. n. *gallinarum*, of hens).

The description is as given for *Bifidobacterium gallinarum* (Watabe et al., 1983) with the following restriction. The G+C content of the type-strain genome is 64.2%, its approximate size 2.16 Mbp, its GenBank deposit SAMN02673435. The type strain is Ch206-5 = AS 1.2283 = ATCC 33777 = BCRC (formerly CCRC) 14679 = CCUG 34990 = DSM 20670 = JCM 6291 = LMG 11586.

#### Description of *Bifidobacterium pullorum* subsp. *Saeculare*, subsp. nov.

B. pul.lo'rum subsp. sae.cu.la're (L. neut. adj. *saeculare*, of or belonging to a saeculum, commemorating the ninth centenary of the foundation of Bologna University).

The description is as given for *Bifidobacterium saeculare* (Biavati et al., 1991). The type strain is RA161 = AS 1.2278 = ATCC 49392 = CCUG 34977 = DSM 6531 = JCM 8223 = LMG 14934.

#### Description of *Cutibacterium acnes* subsp. *Defendens*, comb. nov.

C. ac'nes subsp. de.fen'dens (L. part. adj. *defendens*, defending, guarding, protecting, referring to the CRISPR/Cas system of the strains).

Basonym: *Propionibacterium acnes* subsp. *defendens* McDowell et al. 2016

The description is as given for *Propionibacterium acnes* subsp. *defendens* (McDowell et al., 2016). The type strain is ATCC 11828 = CCUG 6369 = JCM 6473.

#### Description of *Dietzia kunjamensis* subsp. *Schimae*, subsp. nov.

D. kun.ja.men'sis subsp. schi'mae (N.L. gen. n. *schimae*, of the plant genus *Schima*).

The description is as given for *Dietzia schimae* (Li et al., 2008a) with the following modification. The G+C content of the type-strain genome is 70.5%, its approximate size 3.65 Mbp, its IMG deposit 2724679793. The type strain is CCTCC AA 207015 = DSM 45139 = JCM 16003.

#### Description of *Lentzea albidocapillata* subsp. *Violacea*, subsp. nov.

L. al.bi.do.ca.pil.la'ta subsp. vi.o.la'ce.a (L. fem. adj. *violacea*, violet-colored).

The description is as given for *Saccharothrix violacea* (Lee et al., 2000). The type strain is LM 036 = DSM 44796 = JCM 10975.

#### Description of *Mycobacterium intracellulare* subsp. *Chimaera*, subsp. nov.

M. in.tra.cel.lu.la're subsp. chi.mae'ra (L. n. *chimaera*, the chimaera, the mythological being made up of parts of three different animals).

The description is as given for *Mycobacterium chimaera* (Tortoli et al., 2004) with the following addition. The G+C content of the type-strain genome is 67.7%, its approximate size 6.09 Mbp, its GenBank deposit SAMN04216918. The type strain is FI-01069 = CCUG 50989 = CIP 107892 = DSM 44623 = JCM 14737.

#### Description of *Mycobacterium intracellulare* subsp. *Yongonense*, subsp. nov.

M. in.tra.cel.lu.la're subsp. yon.go.nen'se (N.L. neut. adj. *yongonense*, of or belonging to Yongon-Dong, the location of the department performing the taxonomic research on the species).

The description is as given for *Mycobacterium yongonense* (Kim et al., 2013) with the following addition. The G+C content of the type-strain genome is 67.9%, its approximate size 5.66 Mbp, its GenBank deposit SAMN02603187. The type strain is 05-1390 = DSM 45126 = KCTC 19555.

#### Description of *Nocardia salmonicida* subsp. *Cummidelens*, subsp. nov.

N. sal.mo.ni'ci.da subsp. cum.mi.de'lens (L. n. *cummis*, gum, rubber; L. part. adj. *delens*, destroying; N.L. part. adj. *cummidelens*, rubber destroying).

The description is as given for *Nocardia cummidelens* (Maldonado et al., 2000) with the following modification. Colony color can be brown to pale orange or pale pink. The G+C content of the type-strain genome is 67.1%, its approximate size 7.50 Mbp, its GenBank deposit SAMD00040649. The type strain is R89 = CCUG 46121 = CCUG 46295 = CIP 107225 = DSM 44490 = JCM 11439 = NBRC 100378 = NCIMB 13758.

#### Description of *Saccharomonospora iraqiensis* subsp. *Paurometabolica*, subsp. nov.

S. i.ra.qi.en'sis subsp. pau.ro.me.ta.bo'li.ca (Gr. adj. *pauros*, little; Gr. adj. *metabolikos*, changeable; N.L. fem. adj. *paurometabolica*, little changeable, referring to the poor utilization of carbon sources).

The description is as given for *Saccharomonospora paurometabolica* (Li et al., 2003e). The type strain is BCRC (formerly CCRC) 16315 = CCTCC AA 001018 = DSM 44619 = JCM 13241.

#### Description of *Winkia neuii* subsp. *Anitrata*, comb. nov.

W. neu'i.i subsp. a.ni.tra'ta (Gr. pref. a, not; N.L. n. *nitratus*, nitrate; N.L. fem. adj. *anitrata*, not reducing nitrate).

Basonym: *Actinomyces neuii* subsp. anitratus Funke et al. 1994

The description is as given for *Actinomyces neuii* subsp. *anitratus* (Funke et al., 1994b). The type strain is 50/90 = ATCC 51849 = CCUG 32253 = CIP 104016 = DSM 8577 = LMG 14788.

### Taxonomic consequences: emendations

#### Emended description of *Actinobacteria* Stackebrandt et al. 1997

The original proposal of the class “Actinobacteria” (Stackebrandt et al., 1997) did not designate a type order hence this name is not validly published under Rule 27 of the *International Code of Nomenclature of Prokaryotes* (Parker et al., 2015). However, since this name is in common currency it is used here. The type order is likely to be *Actinomycetales* Buchanan 1917 162^AL^ emend. Zhi et al. 2009, 594, a move that would require the approval of the Judicial Commission. Actinobacteria show amazingly diverse morphologies that range from cocci (e.g., *Lapillicoccus* and *Micrococcus*), short rods (e.g., *Mycobacterium* and *Tropheryma*), irregular rods (e.g., *Arcanobacterium* and *Cellulomonas*), rods and cocci (e.g., *Arthrobacter* and *Brevibacterium*) to mycelia that fragment into coccoid and rod-like forms (e.g., *Nocardia* and *Promicromonospora*). Others show more pronounced morphological differentiation which ranges from extensively branched substrate hyphae that carry spores (e.g., *Catellatospora* and *Micromonospora*) or spores vesicles (e.g., *Actinoplanes* and *Dactylosporangium*) to strains that produce a stable branched substrate mycelium which bears aerial hyphae which differentiate into long chains of spores (e.g., *Nonomuraea* and *Streptomyces*) or into spore vesicles (e.g., *Planotetraspora* and *Streptosporangium*). In general, spores are non-motile though ones released from spore vesicles tend to be motile. *Actinobacteria* comprise the orders *Actinomycetales, Bifidobacteriales, Catenulisporales, Corynebacteriales, Cryptosporangiales, Frankiales, Glycomycetales, Jiangellales, Kineosporiales, Micrococcales, Micromonosporales, Propionibacteriales, Pseudonocardiales, Sporichthyales, Streptomycetales* and *Streptosporangiales*.

#### Emended description of *Corynebacteriales* Goodfellow and Jones 2015

The description is as given before (Goodfellow and Jones, 2012) with the following additions. *Corynebacteriales* are found in diverse habitats, notably in the soil ecosystem. Some strains are serious pathogens of humans (e.g., *Mycobacterium tuberculosis*), animals (e.g., *Rhodococcus equi* = *R. hoagii*) and plants (e.g., *Rhodococcus fascians*). The order comprises the families *Corynebacteriaceae, Dietziaceae, Gordoniaceae, Lawsonellaceae, Mycobacteriaceae, Nocardiaceae, Segniliparaceae* and *Tsukamurellaceae*. The type genus is *Corynebacterium*.

#### Emended description of *Micrococcales* Prévot 1940

The description is as given before (Prévot, 1940) with the following additions. The isoprenoid quinone systems are composed of menaquinones with 6–14 isoprenoid units in the side chain; one or two subunits may be saturated. The order consists of the families *Micrococcaceae, Beutenbergiaceae, Bogoriellaceae, Brevibacteriaceae, Cellulomonadaceae, Demequinaceae, Dermabacteraceae, Dermacoccaceae, Dermatophilaceae, Intrasporangiaceae, Jonesiaceae, Kytococcaceae, Microbacteriaceae, Micrococcaceae, Ornithinimicrobiaceae, Promicromonosporaceae, Rarobacteraceae* and *Ruaniaceae*. The new families assigned to this order are *Kytococcaceae, Ornithinimicrobiaceae* and *Tropherymataceae*: in contrast *Sanguibacteraceae* has been assigned to the family *Jonesiaceae*. The type genus is *Micrococcus*.

#### Emended description of *Propionibacteriales* Patrick and McDowell 2015

The description is as given before (Patrick and McDowell, 2012) with the following additions. Cellular fatty acids are composed of different combinations of straight-chain saturated and *iso*-and *anteiso*-methyl branched acids; mycolic acids are absent. The order consists of the established families *Nocardioidaceae* and *Propionibacteriaceae* as well as *Actinopolymorphaceae* fam. nov. and *Kribbellaceae* fam. nov. The type genus is *Propionibacterium*.

#### Emended description of *Streptosporangiales* Goodfellow 2015

The description is as given before (Goodfellow, 2012b) with the following additions. Some strains are thermophilic. The order contains the families *Nocardiopsaceae, Streptosporangiaceae* and *Thermomonosporaceae*. The type genus is *Streptosporangium*.

#### Emended description of *Actinomycetaceae* Buchanan 1918 emend. Stackebrandt et al. 1997 emend. Zhi et al. 2009

The description is as given before (Zhi et al., 2009) together with extensive additions made earlier (Schaal and Yassin, 2012b) and as a result of the present study. In addition to the revised type genus *Actinomyces*, the family contains the established genera *Actinobaculum, Arcanobacterium, Mobiluncus* and *Varibaculum* and seven novel genera, namely *Boudabousia, Bowdenia, Buchananella, Gleimia, Pauljensenia, Schaalia* and *Winkia*.

#### Emended description of *Atopobiaceae* Gupta et al. 2013

The description is as given before (Gupta et al., 2013) with the following additions. Obligately or facultative anaerobe, Gram-stain-positive, non-motile, catalase-negative organisms that form short rods, which often show central swellings or small cocci which may be elliptical. The main fermentation products from glucose are lactic acid with acetic and fumaric acids. Growth is enhanced by Tween 80. The G+C content is around 35–46%. The family includes the type genus *Atopobium, Olsenella* and the novel genera *Fannyhessea* and *Lancefieldella*.

#### Emended description of *Cellulomonadaceae* Stackebrandt and Prauser 1991 emend. Stackebrandt et al. 1997 emend. Stackebrandt and Schumann 2000 emend. Zhi et al. 2009

The description is as given before (Zhi et al., 2009) with a number of additions. Aerobic, Gram-stain-positive, asporogenous organisms which form slender, irregular rods; some rods are arranged at an angle to one another giving V-shaped formations. Strains typically show anaerobic growth in static cultures. Chemoorganotrophic metabolism is primarily respiration but may be fermentative. Most strains form acid from glucose both aerobically and anaerobically. Opaque, usually convex, white, yellowish or yellow colonies formed on peptone-yeast extract agar at neutral pH. Most strains are cellulolytic. The peptidoglycan contains L-lysine as the L-diamino acid; the predominant isoprenologue is MK-9(H_4_). The G+C content is around 70%. The type and only genus is *Cellulomonas*.

#### Emended description of *Corynebacteriaceae* Lehmann and Neumann 1907 emend. Stackebrandt et al. 1997 emend. Zhi et al. 2009

The description is as given before (Zhi et al., 2009) with the following additions. Gram-stain-positive, non-acid fast, non-motile, asporogenous, catalase-positive organisms which form straight to slightly curved rods with tapered ends. Club-shaped elements may be seen. Most species are facultatively anaerobic, others are aerobic. Whole-cell hydrolysates contain DL-A_2_pm, arabinose and galactose. The muramic acid residues of the peptidoglycan are N-acetylated. The peptidoglycan type is A1α with directly cross-linked DL-A_2_pm. The predominant isoprenologues are MK-8(H_2_) and MK-9(H_2_) or a mixture of these components. The major fatty acids are C_16:0_ and C_18:1_ ω9c; tuberculostearic acid may be present. Mycolic acids have 22–38 carbon atoms though some species lack these compounds. The G+C content is around 45–75%. The type and only genus is *Corynebacterium*, now that *Turicella* has been merged with *Corynebacterium*.

#### Emended description of *Demequinaceae* Ue et al. 2011

The emended description is drawn from several sources (Ue et al., 2011). Aerobic or facultatively anaerobic, Gram-stain-positive, non-acid fast, asporogenous, non-motile, catalase-positive organisms that form rods though ovoid to coccoid cells may be present in older cultures. Whole-cell hydrolysates contain arabinose, galactose and mannose. The peptidoglycan has either L-lysine or L-ornithine as the diamino acid though some species have both components; the peptidoglycan type is either A4α or A4β. The predominant isoprenologue is demethylmenaquinone DMK-9(H_2_), and fatty-acid profiles consist mainly of *iso*- and *anteiso-*branched and straight-chain components with *anteiso*-C_15:0_ as the main acid. The principal polar lipids are DPG, PG and PI. The G+C content is around 60–75%. The type and only genus is *Demequina*, now that *Lysinimicrobium* has been merged with *Demequina*.

#### Emended description of *Dermacoccaceae* Stackebrandt and Schumann 2000 emend. Zhi et al. 2009 emend. Ruckmani et al. 2011

The description of this family needs to be radically changed since the previous one (Ruckmani et al., 2011) given the addition of eight genera and the reclassification of the genus *Kytococcus* in *Kytococcaceae*. The revised family encompasses aerobic to microaerophilic, Gram-stain-positive, non-acid-fast, asporogenous, non-motile, predominately catalase-positive organisms that occur either as cocci or short rods. The peptidoglycan is of the A4α type with a L-lysine-glycine-L-serine-D-aspartic acid interpeptide bridge. The peptidoglycan is N-acetylated. Whole-cell hydrolysates contain L-lysine, arabinose, galactose, glucose, mannose, rhamnose, ribose may occur. The major fatty acids are *anteiso-*, methyl- and *iso*-methyl branched components though straight-chain saturated and monounsaturated acids may occur. The common polar lipids are DPG, PG, and PI; some genera also contain PE, PS and PIMs. Diverse menaquinones are found though MK-8(H_4_), MK-8(H_4_) and MK-8(H_6_) are the most frequently found isoprenologues. The G+C content is around 65-80%. The family includes *Dermacoccus*, the type genus*, Barrientosiimonas, Branchiibius, Calidifontibacter, Demetria, Dermacoccus, Flexivirga, Luteipulveratus, Rudaeicoccus, Tamlicoccus*, and *Yimella*.

#### Emended description of *Dermatophilaceae* Austwick 1958 emend. Stackebrandt et al. 1997 emend. Stackebrandt and Schumann 2000 emend. Zhi et al. 2009

The description of this family needs to be revised since the last emendation (Zhi et al., 2009) given the addition of five genera. It includes aerobic to facultatively anaerobic, Gram-stain-positive, acid-fast-negative, catalase-positive organisms which form cocci or branched mycelia; the mature mycelia of dermatophili undergo transverse and longitudinal divisions leading to the production of zoospores which germinate into filaments. Cocci may be motile or non-motile. The peptidoglycan contains DL-A_2_pm as the diamino acid. Whole-cell hydrolysates contain mannose and ribose, the predominant isoprenologue is either MK-8(H_2_) or MK-8(H_4_); fatty acids are mainly unsaturated and straight-chain components. The major polar lipids are DPG, PG, and PI; PE and lyso-PE may occur. The G+C content is around 60–75%. The family includes *Dermatophilus*, the type genus, *Arsenicicoccus, Austwickia, Kineosphaera, Mobilicoccus, Piscicoccus* and *Tonsilliphilus*.

#### Emended description of *Eggerthellaceae* Gupta et al. 2013

The description is as given before (Gupta et al., 2013) with changes reflecting the merging of some genera. The family includes obligatory anaerobic, Gram-stain-positive, asporogenous, asaccharolytic organisms which form cocci and/or short rods. Cells may be motile or non-motile. The peptidoglycan contains DL-A_2_pm as the diamino acid, the predominant isoprenologue is MK-6; fatty acids are mainly saturated and unsaturated and common polar lipids are DPG and PG. The G+C content is around 55–65%. The family includes *Eggerthella*, the type genus, *Adlercreutzia, Cryptobacterium, Denitrobacterium, Gordonibacter, Paraeggerthella* and *Slackia*.

#### Emended description of *Gordoniaceae* Rainey et al. 1997

The description is as given before (Stackebrandt et al., 1997) with a number of additional criteria. Aerobic, Gram-stain-positive, asporogenous, non-motile, catalase-positive organisms which form short rods and cocci; elementary branched hyphae that fragment into rod and coccoid-like elements may occur. Whole-organism hydrolysates contain DL-A_2_pm, arabinose and galactose. The peptidoglycan type is A1α. The muramic acid residues of the peptidoglycan are N-glycolated. The predominant isoprenologue is either MK-9(H_2_) or MK-9 and the common major polar lipids are DPG, PE and PI; PG and PIMs may occur. The fatty-acid profiles contain major amounts of straight-chain saturated, unsaturated and 10-methyloctadecanoic (tuberculeostearic acids). Mycolic acids have 46–70 carbon atoms. The G+C content is around 60–80%. This family includes *Gordonia*, the type genus, *Jongsikchunia* gen. nov. and *Williamsia*.

#### Emended description of *Intrasporangiaceae* Rainey et al. 1997 emend. Stackebrandt and Schumann 2000 emend. Zhi et al. 2009

The description is as given before (Zhi et al., 2009) with the following additions due to the inclusion of recently described genera and the transfer of *Arsenicicoccus* to *Dermatophilaceae* and that of *Ornithinimicrobium* and *Serinicoccus* to *Ornithinimicrobiaceae; Monashia* has been merged with *Intrasporangium*. The revised family encompasses aerobic and facultatively anaerobic, Gram-stain-positive, asporogenous organisms that typically form short rods and cocci though some strains form branched mycelia, others cocci and some a rod-coccus cycle. Generally non-motile (some strains of *Terrabacter* are motile). The peptidoglycan contains either LL- or DL-A2pm as diamino acid. The predominant isoprenologue is typically MK-8(H_4_) but major amounts of MK-8 may also be found; *Aquipuribacter* has MK-10(H_4_) as the major component. Common polar lipids are DPG and PI though PE and PG may occur. Fatty-acid profiles consist of major amounts of *iso*- and *anteiso-*branched-chain acids together with saturated and unsaturated straight-chain components. Mycolic acids are absent. The G+C content is around 65–75%. The family includes *Intrasporangium*, the type genus*, Aquipuribacter, Fodinibacter, Humibacillus, Humihabitans, Intrasporangium, Janibacter, Knoellia, Kribbia, Lapillicoccus, Marihabitans, Ornithinibacter, Ornithinicoccus, Oryzihumus, Oryzobacter, Phycicoccus, Terrabacter, Terracoccus, Tetrasphaera*, and *Pedococcus* gen. nov.

#### Emended description of *Jonesiaceae* Stackebrandt et al. 1997 emend. Zhi et al. 2009

The description is as given before (Zhi et al., 2009) together with additions that reflect the inclusion of additional genera. The family includes aerobic and facultative anaerobic, Gram-stain-positive, asporogenous, motile organisms which form irregular shaped short rods. The peptidoglycan type is A4α with L-lysine as the diamino acid. The major fatty acids are *anteiso*-C_15:0_ and C_16:0_; the predominant isoprenologue is typically MK-9 and the common major polar lipids are DPG, PG, and PI. The G+C content is around 55–75%. The family includes *Jonesia*, the type genus, *Populibacterium, Sanguibacter* and *Flavimobilis* gen. nov.

#### Emended description of *Micromonosporaceae* Krasilnikov 1938 emend. Koch et al. 1996 emend. Stackebrandt et al. 1997 emend. Zhi et al. 2009

The description is as given before (Zhi et al., 2009) together with changes that reflect the merging of some genera. The family encompasses aerobic, Gram-stain-positive, non-acid-fast, mesophilic organisms that form a stable branched and septate mycelium that rarely carries scant aerial hyphae though *Actinocatenispora* forms aerial hyphae that differentiate into chains of around 10 spores. Single spores, short spore chains or spore vesicles are formed on substrate hyphae; spores may be motile with tuffs of polar flagellae and are either smooth or ornamented. The peptidoglycan typically contains DL-A_2_pm (some taxa have L-lysine or 3-OH A_2_pm as the major diamino acid). The muramic acid residues of the peptidoglycan are N-glycolated. Predominant isoprenologues may include all types in the MK-9 and MK-10 series. PE is an ubiquitous polar lipid though DPG, PG, PI and PIMs may occur. Saturated *iso*- and *anteiso-*branched fatty acids predominate in nearly all of the genera. Mycolic acids are absent. The G+C content is around 65–75%. The family includes *Micromonospora*, the type genus*, Actinaurispora, Actinocatenispora, Actinoplanes, Allocatelliglobosispora, Amorphosporangium, Ampullariella, Asanoa, Catellatospora, Catelliglobosispora, Catenuloplanes, Couchioplanes, Dactylosporangium, Hamadaea, Krasilnikovia, Longispora, Luedemannella, Mangrovihabitans, Phytohabitans, Phytomonospora, Pilimelia, Planopolyspora, Planosporangium, Plantactinospora, Polymorphospora, Rhizocola, Rugosimonospora, Salinispora, Spirilliplanes*, and *Virgisporangium*.

#### Emended description of *Mycobacteriaceae* Chester 1897 emend. Stackebrandt et al. 1997 emend. Zhi et al. 2009

The description is as given before (Zhi et al., 2009) with a number of additions. The family contains aerobic to mesophilic, Gram-stain-positive, acid-fast, non-motile, asporogenous organisms which form slightly curved or straight rods though some strains produce branched filaments. The peptidoglycan is of the A1α type. The muramic acid residues of the peptidoglycan are N-glycolated. Whole-cell hydrolysates contain DL-A_2_pm, arabinose and galactose. The predominant isoprenologue is MK-9(H_2_), the major polar lipids are DPG, PE, PI, and PIMs and the main fatty acids straight-chain saturated, unsaturated and 10-methyloctadecanoic (tuberculostearic) acids. Mycolic acids have 60–90 carbon atoms; fatty acid esters released on pyrolysis mass spectrometry (MS) of mycolic acid esters have 22–26 carbon atoms. The G+C content is around 55–75%. The type and only genus is *Mycobacterium*.

#### Emended description of *Nocardiaceae* Castellani and Chalmers 1919 emend. Rainey et al. 1997 emend. Zhi et al. 2009

The description is as given before (Zhi et al., 2009) with changes following the inclusion of *Aldersonia, Hoyosella* and *Tomitella* and the exclusion of *Gordonia* (Stackebrandt et al., 1988) and *Williamsia* (Kämpfer et al., 1999). The family encompasses strictly aerobic and facultatively aerobic, Gram-stain-positive to Gram-stain variable, non-motile, catalase-positive organisms some of which form a extensively branched substrate mycelium that fragments into coccoid to rod-shaped elements, others are coccoid or rod-shaped or show a rod-coccus, rod-coccus-mycelium growth cycle. Strains are typically acid-alcohol or partially acid-alcohol fast at some stage of the growth cycle. Chemo-organotrophic with an oxidative metabolism. The pepetidoglycan is of the A1α type. The muramic acid residues of the peptidoglycan are N-glycolated. Predominant isoprenologues are MK-8(H_2_) or MK-9(H_2_) or MK-8(H_4_); major polar lipids are DPG PE, PI and PIMs, and fatty acids are mainly straight-chain saturated, unsaturated and 10-methyloctadecanoic (tuberculostearic) acids. Mycolic acids have between 34 and 64 carbon atoms; fatty-acid esters released on pyrolysis MS of mycolic acid esters have 12–18 carbon atoms. The G+C content is about 60–75%. The family includes *Nocardia*, the type genus, *Millisia, Rhodococcus, Skermania, Tomitella* and *Aldersonia* gen. nov.

#### Emended description of *Nocardioidaceae* Nesterenko et al. 1990 emend. Rainey et al. 1997 emend. Zhi et al. 2009

The description of the family requires substantial modifications from that given previously (Zhi et al., 2009), notably the addition of *Mumia* and the reclassification of *Actinopolymorpha* in *Actinopolymorphaceae* and that of *Kribbella, Flindersiella, Tenggerimyces*, and *Thermasporomyces* in *Kribbellaceae. Nocardioidaceae* include aerobic, Gram-stain-positive, non-acid-fast organisms which form branched substrate hyphae or irregular rods or cocci; substrate hyphae and aerial hyphae, when formed, fragment into rod-like and coccoid cells. Rod-shaped cells may be motile. The peptidoglycan contains LL-A_2_pm and glycine as amino acids and is of the A3α type; muramic acid residues of the peptidoglycan are N-acetylated; cell walls contain teichoic acids. The predominant isoprenologue is MK-8(H_4_), the common polar lipids are DPG and PG though PC, PE, PI and PIMs may occur; the fatty acids consist of saturated and monounsaturated straight-chain and *iso-, anteiso-* and 10-methyl-branched components. The G+C content is around 65-75%. The family includes *Nocardioides*, the type genus, *Marmoricola* and *Mumia*.

#### Emended description of *Promicromonosporaceae* Rainey et al. 1997 emend. Zhi et al. 2009

The description is as given before (Zhi et al., 2009) with the following additions. The family includes aerobic to facultatively anaerobic, Gram-stain positive, acid-fast-negative organisms which form a substrate mycelium that fragments into irregular elements or presents as cocci or short rods. The peptidoglycan is of the A4α type. Whole-cell hydrolysates contain L-lysine as the diamino acid and combinations of different sugars. The predominant menaquinone is MK-9(H_2_) or MK-9(H_4_) sometimes with MK-8(H_2_) or MK-8(H_4_), and major polar lipids are DPG and PG; PI and PIMs may occur. Branching fatty acids are of the *iso*- and *anteiso-*type, notably *iso*-C_15:0_ and *anteiso*-C_15:0_. Mycolic acids are absent. The G+C content is around 70-80%. The family includes *Promicromonospora*, the type genus, *Cellulosimicrobium, Isoptericola, Luteimicrobium, Krasilnikoviella, Myceligenerans, Oerskovia, Paraoerskovia, Xylanibacterium, Xylanimicrobium*, and *Xylanimonas*.

#### Emended description of *Pseudonocardiaceae* Embley et al. 1988 emend. Stackebrandt et al. 1997 emend. Zhi et al. 2009 emend. Labeda et al. 2011

The description is as given before (Labeda et al., 2011) with the following additions. Cell-wall hydrolysates contain DL-A_2_pm as diaminoacid and galactose or arabinose as whole-cell sugars. PE or DPG, PC or PG and lyso-PG are evident in polar-lipid patterns. Fatty acids are composed of straight-chain saturated and monounsaturated and *iso*-and *anteiso*-branched compounds. The family includes *Pseudonocardia*, the type genus, *Actinoalloteichus, Actinobispora, Actinokineospora, Actinomycetospora, Actinophytocola, Actinosynnema, Alloactinosynnema Allokutzneria Amycolata, Amycolatopsis, Crossiella, Faenia, Goodfellowiella, Haloechinothrix, Kibdelosporangium, Kutzneria, Labedaea, Lentzea, Longimycelium, Prauserella, Pseudoamycolata, Pseudonocardia, Saccharomonospora, Saccharopolyspora, Saccharothrix, Sciscionella, Streptoalloteichus, Thermobispora, Thermocrispum*, and *Umezawaea. Lechevalieria* and *Yuhushiella* have been merged with *Lentzea* and *Amycolatopsis*, respectively.

#### Emended description of *Streptomycetaceae* Waksman and Henrici 1943 emend. Rainey et al. 1997 emend. Kim et al. 2003 emend. Zhi et al. 2009 emend. Huang et al. 2017

The description is as given before (Huang et al., 2017) with changes following the addition of several new genera. The family encompasses aerobic, Gram-stain-positive, non-acid-alcohol-fast, non-motile organisms that form a stable, extensively branched substrate mycelium and aerial hyphae that differentiate into two to many spores. Short chains of spores may be observed on substrate hyphae. Most strains produce a wide range of pigments which are responsible for the color of substrate and aerial mycelia. Strains are generally chemoautotrophic and mesophilic but some are autotrophic and thermophilic. The peptidoglycan of the substrate mycelium contains either LL-or DL-A_2_pm as the predominant diamino acid, aerial hyphae and submerged spores have LL-A_2_pm; the peptidoglycan type is A3α. The predominant isoprenologues are typically MK-9(H_6_, H_8_), common polar lipids are DPG and PIMs though PE and PI also occur; fatty acids are rich in saturated and *iso*- and *anteiso*-compounds. The G+C content is around 65–75%. The family contains *Streptomyces*, the type genus, *Allostreptomyces, Kitasatospora* and *Streptacidiphilu*s and the novel genera *Embleya* and *Yinghuangia*.

#### Emended description of *Streptosporangiaceae* Goodfellow et al. 1990 emend. Ward-Rainey et al. 1997 emend. Zhi et al. 2009

The description is as given before (Zhi et al., 2009) with changes reflecting inclusion of additional genera, notably *Pauljensenia* and *Thermobispora*. The family includes aerobic, Gram-stain-positive, non-acid-alcohol-fast, chemoorganotrophic organisms which form a stable, branched susbstrate mycelium. When produced, aerial hyphae differentiate into either two or more spores or into spore vesicles that enclose one to many spores. Spores may be motile or non-motile. Most strains are mesophilic but some are thermophilic. The muramic acid residues of the peptidoglycan are N-acetylated. The peptidoglycan contains DL-A_2_pm and is of the A2α type. Predominant isoprenologues include all types in the MK-9 series, PE occurs as polar lipid; cells contain major amounts of *iso*-, *anteiso*-saturated, unsaturated and 10-methyl-branched components. Mycolic acids are absent. The G+C content is around 60–80%. The family contains *Streptosporangium*, the type genus, *Acrocarpospora, Herbidospora, Microbispora, Microtetraspora, Nonomuraea, Planobispora, Planomonospora, Planotetraspora, Pauljensenia, Sphaerisporangium, Thermoactinospora, Thermobispora, Thermocatellispora*, and *Thermopolyspora*.

#### Emended description of *Actinokineospora* Hasegawa 1988 emend. Labeda et al. 2010 emend. Tang et al. 2012

The description is as given before (Tang et al., 2012) with additions following the inclusion of *Alloactinosynnema*. The ubiquitous whole-cell sugar is galactose but strains can also contain arabinose, galactose, mannose or ribose. The polar-lipid profile includes DPG, PE, PG, PI, or PC. Cells can be filamentous or rod shaped. The G+C content is about 65–75%. The type species is *Actinokineospora riparia*.

#### Emended description of *Actinoplanes* Couch 1950 emend. Stackebrandt and Kroppenstedt 1988

The description is as given before (Stackebrandt and Kroppenstedt, 1987) with additions following the inclusion of *Pseudosporangium*. Whole-cell hydrolysates are rich in arabinose, galactose, glucose, mannose, ribose and xylose or arabinose, galactose and xylose; fatty-acid profiles contain *iso*-C_15:0_, C_18:1_ ω9c and C_16:0_ or *iso*-C_15:0_ and *iso*-C_16:0_; the predominant menaquinone is either MK-9(H_4_) or MK-9(H_6_). The DNA G+C content is around 69–74%. The type species is *Actinoplanes philippinensis*.

#### Emended description of *Actinomyces* Harz 1877

The description of the genus needs to be revised since the initial description (Harz, 1877) following the assignment of certain *Actinomyces* species to novel genera. Aerobic to facultatively anaerobic, Gram-stain-positive, asporogenous, non-motile organisms which tend to form short straight to slightly curved rods that occur singly or in pairs or as Y-, V-, or T-forms; filaments and branched rods may be evident; some strains present as cocci or coccoid rods. The peptidoglycan contains either L-lysine or L-ornithine as the diamino acid. Whole-cell hydrolysates contain 6-deoxytalase, fucose, galactose and glucose. MK-10 is the predominant isoprenologue and PC the predominant polar lipid. The G+C content is 57–71%. The type species is *Actinomyces bovis*.

#### Emended description of *Adlercreutzia* Maruo et al. 2008

The description is as given before (Maruo et al., 2008) with changes reflecting the addition of *Asaccharobacter, Enterorhabdus* and *Parvibacter*. Whole-cell hydrolysates contain DL-or LL-A_2_pm as the diamino acid and galactose with either ribose or glucose. The predominant isoprenologues are methylmenaquinone-6 and demethylmenaquinone-6. Major fatty acids are either C_16:0_, *iso*-C_15:0_ or C_16:0_, *iso*-C_17:0_ and C_18:1_ ω9c. The G+C content is about 60–70%. The type species is *Adlercreutzia equolifaciens*.

#### Emended description of *Amycolatopsis* Lechevalier et al. 1986 emend. Lee 2009

The description is as given before (Lee, 2009) with additions that reflect developments in the composition of the genus, notably the removal of *A. halophila* (Tang et al., 2010a) and *A. salitolerans* (Guan et al., 2012) and the inclusion of *Yuhushiella* (Mao et al., 2011). The revised genus contains aerobic to facultatively anaerobic organisms that form branching substrate hyphae that fragment into squarish and rod-shaped elements. When formed, aerial hyphae may be sterile or differentiated into chains of smooth surfaced, squarish to ellipsoidal spore-like structures. The peptidoglycan is of the A1α type, and muramic acid moieties are N-acetylated. Whole-cell hydrolysates are rich in DL-A_2_pm, arabinose and galactose and the major polar lipids DPG, PE, PG, PI and PME; the fatty acids consist of complex mixtures of saturated and branched-chain components. The G+C content is about 65-75%. The type species is *Amycolatopsis orientalis*.

#### Emended description of *Corynebacterium* Lehmann and Neumann 1896 emend. Bernard et al. 2010

The description is as given before (Bernard et al., 2010) with additions that reflect developments in the composition of the genus, notably the inclusion of the genus *Turicella* (Funke et al., 1994a). The emended description is the same as that given earlier for the family *Corynebacteriaceae*. The type species is *Corynebacterium diphtheriae*.

#### Emended description of *Demequina* Yi et al. 2007 emend. Ue et al. 2011 emend. Park et al. 2016

The description is as given before (Park et al., 2016) with additions following the inclusion of *Lysinimicrobium*. The emended description is as given earlier for the family *Demequinaceae*. The type species is *Demequina aestuarii*.

#### Emended description of *Gulosibacter* Manaia et al. 2004 emend. Park et al. 2012

The description is as given before (Park et al., 2012) with additions following the inclusion of *Zimmermannella bifida* and *Z. faecalis*. The revised genus encompasses irregular, non-motile, non-acid-fast, irregular rod-shaped cells that have a tendency to form short filaments and branched elements. Cell-wall peptidoglycan contains DAB or D-ornithine as a diamino acid. Whole-cell hydrolysates contain mainly glucose and ribose or rhamnose; predominant menaquinones are MK-8, MK-9, and MK-10, and fatty acid profiles are rich in C_16:0_, *iso*-C_16:0_, *anteiso*-C_15:0_, *iso*-C_16:0_, *anteiso*-C_17:0_ and *anteiso*-C_15:0_. The G+C content is around 60–70%. The type species is *Gulosibacter molinativorax*.

#### Emended description of *Haloechinothrix* Tang et al. 2010

The description is as given before (Tang et al., 2010b) with additions following the inclusion of *Amycolatopsis halophila* and *A. salitolerans*. Substrate mycelium fragments into squarish rod-like elements; aerial mycelium may differentiate into long chains of spore-like elements at maturity. Whole-cell hydrolysates are rich in galactose and glucose (ribose is variable) or in glucose, glucosamine and mannose. Common phospholipids are DPG, PE, OH-PE, PI, PIM, and PL or DPG, PME, PE and glucosamine-containing PL or DPG, PG, PE, and PIM. Major fatty acids are *iso*-C_16:0_ or C_16:0_; mycolic acids are absent. The G+C content is around 65–70%. The type species is *Haloechinothrix alba*.

#### Emended description of *Intrasporangium* Kalakoutskii et al. 1967 emend. Liu et al. 2012 emend. Yang et al. 2012

The description is as given before (Yang et al., 2012) with additions following the inclusion of *Monashia flava*. Produces irregular coccoid to short rods or a branching substrate mycelium that fragments into pleomorphic elements. Does not form aerial mycelium. Whole-cell hydrolysates are rich in mannose with lower amounts of galactose and rhamnose. The cellular fatty-acid profile is dominated by *iso*-C_14:0_, *iso*-C_15:0_, *anteiso*-C_15:0_ and *iso*-C_16:0_ or *iso*-C_15:0_, *anteiso*-C_15:0_, and *iso*-C_16:0_ components. The G+C content is around 65–75%. The type species is *Intrasporangium calvum*.

#### Emended description of *Kitasatospora* Omura et al. 1982 emend. Zhang et al. 1997

The description is as given before (Zhang et al., 1997) with the following additions. Whole-cell hydrolysates are rich in galactose or ribose, glucose and mannose; the predominant isoprenologues are MK-9(H_4_, H_6_, H_8_) or MK-9(H_6_, H_8_); the major polar lipids DPG, PE, PI and PIMs; fatty acids are rich in saturated, *iso*- and *anteiso*-components. The G+C content is around 65–80%. The type species is *Kitasatospora setae*.

#### Emended description of *Knoellia* Groth et al. 2002

The description is as given before (Groth et al., 2002) with additions following the inclusion of *Tetrasphaera remsis*. Cells are irregular rods and cocci that occur, singly, in pairs, tetrades, clusters and as short-rods. Fatty-acids are mainly *iso-* and *anteiso-*branched components. Mycolic acids are absent. The G+C content is around 65–75%. The type species is *Knoellia sinensis*.

#### Emended description of *Lentzea* Yassin et al. 1995 emend. Labeda et al. 2001 emend. Fang et al. 2017

The description is as given before (Fang et al., 2017) with additions following the inclusion of *Lechevalieria*. Aerial mycelium is produced but may be scant. The G+C content is around 65-80%. The type species is *Lentzea albidocapillata*.

#### Emended description of *Microlunatus* Nakamura et al. 1995

The description is as given before (Nakamura et al., 1995) with additions following the inclusion of *Friedmanniella*. Coccoid or spherical cells occur singly, in pairs and sometimes in clusters. Major fatty acids are *anteiso*-C_15:0_ and *iso*-C_15:0_; iso-C_16:0_ may occur. The G+C content is around 60–75%. The type species is *Microlunatus phosphovorus*.

#### Emended description of *Micromonospora* Orskov 1923 emend. Gao et al. 2014

The initial description (Ørskov, 1923) has to be changed dramatically (Gao et al., 2014) given the inclusion of many additional *Micromonospora* species as well as *Verrucosispora* and *Xiangella*. The genus contains aerobic to microaerophilic, Gram-stain-positive, non-acid-fast organisms that form a well developed, branched, substrate mycelium. Non-motile spores are borne singly and are either sessile or carried on short or long sporophores. Aerial hyphae are usually absent. The peptidoglycan contains DL-A_2_pm and/or hydroxy-A_2_pm and muramic acid residues of the peptidoglycan are N-glycolated. Whole-cell hydrolysates are rich in xylose with either mannose or galactose, glucose and mannose; predominant isoprenologues are MK-9(H_4_, H_6_), MK-10(H_4_, H_6_) or MK-12(H_2_) and common major polar lipids PE and PIMs; DPG, PI and PS may occur. The fatty-acid profile contains *iso*-C_16:0_ and *iso*-C_15:0_ or *iso*-C_15:0_ and C_16:0_. Mycolic acids are absent. The G+C content is around 65–75%. The type species is *Micromonospora chalcea*.

#### Emended description of *Phycicoccus* Lee 2006 emend Zhang et al. 2011

The description is as given before (Zhang et al., 2011) with the following changes. The major cell-wall diamino acid is DL-DAP acid or 3-hydroxy DL-DAP. Galactose and glucose are the main whole-cell sugars. The G+C content is around 70-75%. The type species is *Phycicoccus jejuensis*.

#### Emended description of *Rothia* Georg and Brown 1967

The initial description of the genus (Georg and Brown, 1967) has been changed given the inclusion of additional *Rothia* species as well as *Kocuria halotolerans, K. koreensis* and *K. kristinae*. The revised genus contains Gram-stain-positive, acid-fast-negative, asporogenous organisms that may be coccoid, diphtheroid or filamentous or a mixture of these forms. The peptidoglycan contains L-lysine, as the diamino acid and is of the A3α type. Menaquinone patterns vary though most strains have MK-6(H_2_), MK-7, MK-8, MK-9(H_2_) components either singly or in combination, though others have MK-7(H_2_) and in some cases MK-8(H_2_) as the predominant isoprenologue. The major fatty acids are *anteiso*- and *iso*-methyl-branched though straight-chain saturated and mono-unsaturated components may occur; mycolic acids are absent. The G+C content is around 50-75%. The type species is *Rothia dentocariosa*.

#### Emended description of *Thermomonospora* Henssen 1957 emend. Zhang et al. 1998

The last description of the genus (Zhang et al., 1998) needs to be revised following the exclusion of *Thermomonospora chromogena* and the inclusion of *Actinomadura amylolytica, A. cellulosilytica, A. echinospora* and *A. umbrina*. The revised genus encompasses aerobic, Gram-stain-positive, non-acid-fast, chemoorganotrophic organisms which form a branched substrate mycelium that carries aerial hyphae. Single or short chains of spores formed on the aerial mycelium have either a spiny or rugose ornamentation; spores may be borne on sporophores. The temperature range for growth is 25–55°C. The diamino acid of the peptidoglycan is DL-A_2_pm. Whole-cell hydrolysates may contain glucose, madurose, mannose and rhamnose; ribose, xylose, madurose, galactose and glucose; or just ribose. The predominant isoprenologues are MK-9(H_2_, H_4_, H_6_, H_8_) and the major polar lipids are DPG, PI, PG, and PIM. Fatty-acid profiles are rich in straight-chain, *iso*-and *anteiso*-components. Mycolic acids are absent. The G+C contant is around 65–75%. The type species is *Thermomonospora curvata*.

#### Emended description of *Acaricomes phytoseiuli* Pukall et al. 2006

The description is as before (Pukall et al., 2006) with the following modification. The G+C content of the type-strain genome is 62.3%, its approximate size 2.42 Mbp, its GenBank deposit SAMN02441381.

#### Emended description of *Acidimicrobium ferrooxidans* Clark and Norris 1996

The description is as before (Clark and Norris, 1996) with the following restriction. The G+C content of the type-strain genome is 68.3%, its approximate size 2.16 Mbp, its GenBank deposit SAMN02598466.

#### Emended description of *Acidipropionibacterium acidipropionici* (Orla-Jensen, 1909) Scholz and Kilian 2016

The description is as before (Scholz and Kilian, 2016) with the following addition. The G+C content of the type-strain genome is 68.8%, its approximate size 3.59 Mbp, its GenBank deposit SAMN02441222.

#### Emended description of *Acidipropionibacterium jensenii* (van Niel, 1928) Scholz and Kilian 2016

The description is as before (Scholz and Kilian, 2016) with the following addition. The G+C content of the type-strain genome is 68.7%, its approximate size 3.03 Mbp, its GenBank deposit SAMN02441538.

#### Emended description of *Acidipropionibacterium thoenii* (van Niel, 1928) Scholz and Kilian 2016

The description is as before (Scholz and Kilian, 2016) with the following addition. The G+C content of the type-strain genome is 68.0%, its approximate size 2.94 Mbp, its GenBank deposit SAMN02440839.

#### Emended description of *Acidothermus cellulolyticus* Mohagheghi et al. 1986

The description is as before (Mohagheghi et al., 1986) with the following restriction. The G+C content of the type-strain genome is 66.9%, its approximate size 2.44 Mbp, its GenBank deposit SAMN02598358.

#### Emended description of *Actinoalloteichus hymeniacidonis* Zhang et al. 2006

The description is as before (Zhang et al., 2006) with the following addition. The G+C content of the type-strain genome is 68.1%, its approximate size 6.31 Mbp, its GenBank deposit SAMN03295676.

#### Emended description of *Actinobaculum suis* (Wegienek and Reddy 1982) Lawson et al. 1997 emend. Yassin et al. 2015

The description is as before (Yassin et al., 2015) with the following modification. The G+C content of the type-strain genome is 57.8%, its approximate size 2.20 Mbp, its GenBank deposit SAMN05421878.

#### Emended description of *Actinokineospora diospyrosa* Tamura et al. 1995

The description is as before (Tamura et al., 1995) with the following modification. The G+C content of the type-strain genome is 70.3%, its approximate size 8.11 Mbp, its GenBank deposit SAMN03080616.

#### Emended description of *Actinokineospora globicatena* Tamura et al. 1995

The description is as before (Tamura et al., 1995) with the following modification. The G+C content of the type-strain genome is 70.8%, its approximate size 7.64 Mbp, its GenBank deposit SAMN03080607.

#### Emended description of *Actinokineospora inagensis* Tamura et al. 1995

The description is as before (Tamura et al., 1995) with the following modification. The G+C content of the type-strain genome is 70.2%, its approximate size 7.28 Mbp, its GenBank deposit SAMN02441427.

#### Emended description of *Actinokineospora spheciospongiae* Kämpfer et al. 2015

The description is as before (Kämpfer et al., 2015) with the following addition. The G+C content of the type-strain genome is 72.8%, its approximate size 7.53 Mbp, its GenBank deposit SAMN02382011.

#### Emended description of *Actinokineospora terrae* Tamura et al. 1995

The description is as before (Tamura et al., 1995) with the following modification. The G+C content of the type-strain genome is 71.0%, its approximate size 7.58 Mbp, its GenBank deposit SAMN04487818.

#### Emended description of *Actinomadura atramentaria* Miyadoh et al. 1987

The description is as before (Miyadoh et al., 1987) with the following modification. The G+C content of the type-strain genome is 73.7%, its approximate size 6.71 Mbp, its GenBank deposit SAMN02441315.

#### Emended description of *Actinomadura flavalba* Qin et al. 2009

The description is as before (Qin et al., 2009b) with the following modification. The G+C content of the type-strain genome is 73.3%, its approximate size 6.17 Mbp, its GenBank deposit SAMN02441028.

#### Emended description of *Actinomadura hibisca* Tomita et al. 1991

The description is as before (Tomita et al., 1990) with the following addition. The G+C content of the type-strain genome is 72.4%, its approximate size 9.02 Mbp, its GenBank deposit SAMD00046515.

#### Emended description of *Actinomadura kijaniata* Horan and Brodsky 1982

The description is as before (Horan and Brodsky, 1982) with the following addition. The G+C content of the type-strain genome is 73.2%, its approximate size 10.26 Mbp, its GenBank deposit SAMD00046512.

#### Emended description of *Actinomadura macra* (ex Celmer et al. 1979) Huang 1980

The description is as before (Huang, 1980) with the following addition. The G+C content of the type-strain genome is 70.7%, its approximate size 9.05 Mbp, its GenBank deposit SAMD00046507.

#### Emended description of *Actinomadura madurae* (Vincent 1894) Lechevalier and Lechevalier 1970

The description is as before (Lechevalier and Lechevalier, 1970) with the following addition. The G+C content of the type-strain genome is 72.1%, its approximate size 10.25 Mbp, its GenBank deposit SAMN04489713.

#### Emended description of *Actinomadura mexicana* Quintana et al. 2004

The description is as before (Quintana et al., 2003) with the following addition. The G+C content of the type-strain genome is 72.3%, its approximate size 8.79 Mbp, its IMG deposit 2724679706.

#### Emended description of *Actinomadura meyerae* Quintana et al. 2004

The description is as before (Quintana et al., 2003) with the following addition. The G+C content of the type-strain genome is 72.9%, its approximate size 8.57 Mbp, its GenBank deposit SAMN05443665.

#### Emended description of *Actinomadura oligospora* Mertz and Yao 1986

The description is as before (Mertz and Yao, 1986) with the following addition. The G+C content of the type-strain genome is 72.0%, its approximate size 9.35 Mbp, its GenBank deposit SAMN02584999.

#### Emended description of *Actinomadura pelletieri* (Laveran 1906) Lechevalier and Lechevalier 1970

The description is as before (Lechevalier and Lechevalier, 1970) with the following addition. The G+C content of the type-strain genome is 70.4%, its approximate size 7.53 Mbp, its IMG deposit 2728369498.

#### Emended description of *Actinomadura rubrobrunea* (ex Krassilnikov et al. 1968) Kroppenstedt et al. 1991

The description is as before (Kroppenstedt et al., 1990) with the following addition. The G+C content of the type-strain genome is 72.8%, its approximate size 6.72 Mbp, its GenBank deposit SAMD00046516.

#### Emended description of *Actinomadura viridilutea* (Agre and Guzeva 1975) Zhang et al. 2001

The description is as before (Zhang et al., 2001) with the following addition. The G+C content of the type-strain genome is 72.8%, its approximate size 6.72 Mbp, its IMG deposit 2728369515.

#### Emended description of *Actinomyces dentalis* Hall et al. 2005

The description is as before (Hall et al., 2005) with the following modification. The G+C content of the type-strain genome is 73.1%, its approximate size 3.53 Mbp, its GenBank deposit SAMN02440612.

#### Emended description of *Actinomyces denticolens* Dent and Williams 1984

The description is as before (Dent and Williams, 1984) with the following restriction. The G+C content of the type-strain genome is 71.2%, its approximate size 2.90 Mbp, its GenBank deposit SAMD00059584.

#### Emended description of *Actinomyces israelii* (Kruse 1896) Lachner-Sandoval 1898

The description is as before (Lachner-Sandoval, 1898) with the following addition. The G+C content of the type-strain genome is 71.4%, its approximate size 4.03 Mbp, its GenBank deposit SAMN02745698.

#### Emended description of *Actinomyces massiliensis* Renvoise et al. 2009

The description is as before (Renvoise et al., 2009) with the following addition. The G+C content of the type-strain genome is 67.8%, its approximate size 3.35 Mbp, its GenBank deposit SAMN02469892.

#### Emended description of *Actinomyces naeslundii* Thompson and Lovestedt 1951 emend. Henssge et al. 2009

The description is as before (Henssge et al., 2009) with the following modification. The G+C content of the type-strain genome is 67.9%, its approximate size 3.11 Mbp, its GenBank deposit SAMN00828754.

#### Emended description of *Actinomyces radicidentis* Collins et al. 2001

The description is as before (Collins et al., 2000a) with the following addition. The G+C content of the type-strain genome is 72.6%, its approximate size 3.05 Mbp, its GenBank deposit SAMN04435863.

#### Emended description of *Actinomyces ruminicola* An et al. 2006

The description is as before (An et al., 2006) with the following modification. The G+C content of the type-strain genome is 69.9%, its approximate size 3.09 Mbp, its GenBank deposit SAMN05216355.

#### Emended description of *Actinomyces slackii* Dent and Williams 1986

The description is as before (Dent and Williams, 1986) with the following restriction. The G+C content of the type-strain genome is 70.1%, its approximate size 3.17 Mbp, its GenBank deposit SAMN02441219.

#### Emended description of *Actinomyces timonensis* Renvoise et al. 2010

The description is as before (Renvoise et al., 2010) with the following addition. The G+C content of the type-strain genome is 71.2%, its approximate size 2.91 Mbp, its GenBank deposit SAMN02472053.

#### Emended description of *Actinomyces urogenitalis* Nikolaitchouk et al. 2000

The description is as before (Nikolaitchouk et al., 2000) with the following modification. The G+C content of the type-strain genome is 68.7%, its approximate size 2.61 Mbp, its GenBank deposit SAMN00002545.

#### Emended description of *Actinomycetospora chiangmaiensis* Jiang et al. 2008

The description is as before (Jiang et al., 2008) with the following modification. The G+C content of the type-strain genome is 74.0%, its approximate size 5.86 Mbp, its GenBank deposit SAMN02256421.

#### Emended description of *Actinoplanes cyaneus* Terekhova et al. 1987

The description is as before (Terekhova et al., 1977) with the following addition. The G+C content of the type-strain genome is 71.0%, its approximate size 10.42 Mbp, its IMG deposit 2724679919.

#### Emended description of *Actinoplanes derwentensis* Goodfellow et al. 1990

The description is as before (Goodfellow et al., 1990) with the following addition. The G+C content of the type-strain genome is 69.4%, its approximate size 10.65 Mbp, its GenBank deposit SAMN04489716.

#### Emended description of *Actinoplanes friuliensis* Aretz et al. 2001 emend. Wink et al. 2014

The description is as before (Wink et al., 2014) with the following addition. The G+C content of the type-strain genome is 70.4%, its approximate size 9.38 Mbp, its GenBank deposit SAMN02603031.

#### Emended description of *Actinoplanes globisporus* (Thiemann 1967) Stackebrandt and Kroppenstedt 1988

The description is as before (Stackebrandt and Kroppenstedt, 1987) with the following addition. The G+C content of the type-strain genome is 70.9%, its approximate size 10.99 Mbp, its GenBank deposit SAMN02256449.

#### Emended description of *Actinoplanes italicus* Beretta 1973

The description is as before (Beretta, 1973) with the following addition. The G+C content of the type-strain genome is 70.4%, its approximate size 11.15 Mbp, its IMG deposit 2728369518.

#### Emended description of *Actinoplanes missouriensis* Couch 1963

The description is as before (Couch, 1963) with the following addition. The G+C content of the type-strain genome is 70.8%, its approximate size 8.77 Mbp, its GenBank deposit SAMD00060941.

#### Emended description of *Actinoplanes philippinensis* Couch 1950

The description is as before (Couch, 1950) with the following addition. The G+C content of the type-strain genome is 71.4%, its approximate size 10.38 Mbp, its GenBank deposit SAMN05421541.

#### Emended description of *Actinoplanes rectilineatus* Lechevalier and Lechevalier 1975

The description is as before (Lechevalier and Lechevalier, 1975) with the following addition. The G+C content of the type-strain genome is 70.6%, its approximate size 10.32 Mbp, its GenBank deposit SAMN03327476.

#### Emended description of *Actinoplanes regularis* (Couch, 1963) Stackebrandt and Kroppenstedt 1988

The description is as before (Stackebrandt and Kroppenstedt, 1987) with the following addition. The G+C content of the type-strain genome is 70.3%, its approximate size 10.03 Mbp, its IMG deposit 2724679777.

#### Emended description of *Actinopolymorpha alba* Cao et al. 2009

The description is as before (Cao et al., 2009) with the following modification. The G+C content of the type-strain genome is 67.9%, its approximate size 7.98 Mbp, its GenBank deposit SAMN02440435.

#### Emended description of *Actinopolymorpha singaporensis* Wang et al. 2001

The description is as before (Wang et al., 2001) with the following modification. The G+C content of the type-strain genome is 70.6%, its approximate size 6.40 Mbp, its GenBank deposit SAMN04489717.

#### Emended description of *Actinopolyspora erythraea* Tang et al. 2011

The description is as before (Tang et al., 2011) with the following modification. The G+C content of the type-strain genome is 68.8%, its approximate size 5.34 Mbp, its GenBank deposit SAMN02911260.

#### Emended description of *Actinopolyspora halophila* Gochnauer et al. 1975

The description is as before (Gochnauer et al., 1975) with the following modification. The G+C content of the type-strain genome is 68.0%, its approximate size 5.35 Mbp, its GenBank deposit SAMN02261305.

#### Emended description of *Actinopolyspora mzabensis* Meklat et al. 2013

The description is as before (Meklat et al., 2013a) with the following addition. The G+C content of the type-strain genome is 67.7%, its approximate size 5.00 Mbp, its GenBank deposit SAMN04487820.

#### Emended description of *Actinopolyspora righensis* Meklat et al. 2014

The description is as before (Meklat et al., 2013b) with the following addition. The G+C content of the type-strain genome is 67.5%, its approximate size 4.92 Mbp, its GenBank deposit SAMN04487904.

#### Emended description of *Actinopolyspora saharensis* Meklat et al. 2013

The description is as before (Meklat et al., 2013c) with the following addition. The G+C content of the type-strain genome is 69.5%, its approximate size 4.68 Mbp, its GenBank deposit SAMN04489718.

#### Emended description of *Actinosynnema mirum* Hasegawa et al. 1978

The description is as before (Hasegawa et al., 1978) with the following addition. The G+C content of the type-strain genome is 73.7%, its approximate size 8.25 Mbp, its GenBank deposit SAMN00001904.

#### Emended description of *Actinosynnema pretiosum* Hasegawa et al. 1983

The description is as before (Hasegawa et al., 1983) with the following restriction. The G+C content of the type-strain genome is 73.4%, its approximate size 9.35 Mbp, its GenBank deposit SAMN03080609.

#### Emended description of *Aeromicrobium erythreum* Miller et al. 1991

The description is as before (Miller et al., 1991) with the following restriction. The G+C content of the type-strain genome is 72.1%, its approximate size 3.63 Mbp, its GenBank deposit SAMN03701813.

#### Emended description of *Agreia bicolorata* Evtushenko et al. 2001

The description is as before (Evtushenko et al., 2001) with the following modification. The G+C content of the type-strain genome is 65.2%, its approximate size 3.92 Mbp, its GenBank deposit SAMN03333328.

#### Emended description of *Agrococcus baldri* Zlamala et al. 2002

The description is as before (Zlamala et al., 2002) with the following restriction. The G+C content of the type-strain genome is 71.2%, its approximate size 2.90 Mbp, its GenBank deposit SAMN04487783.

#### Emended description of *Agrococcus lahaulensis* Mayilraj et al. 2006

The description is as before (Mayilraj et al., 2006b) with the following modification. The G+C content of the type-strain genome is 72.5%, its approximate size 2.68 Mbp, its GenBank deposit SAMN02440814.

#### Emended description of *Agromyces cerinus* Zgurskaya et al. 1992

The description is as before (Zgurskaya et al., 1992) with the following modification. The G+C content of the type-strain genome is 70.0%, its approximate size 4.19 Mbp, its GenBank deposit SAMN05443544.

#### Emended description of *Agromyces subbeticus* Jurado et al. 2005

The description is as before (Jurado et al., 2005) with the following modification. The G+C content of the type-strain genome is 69.1%, its approximate size 4.30 Mbp, its GenBank deposit SAMN02441229.

#### Emended description of *Allokutzneria albata* (Tomita et al. 1993) Labeda and Kroppenstedt 2008

The description is as before (Labeda and Kroppenstedt, 2008) with the following modification. The G+C content of the type-strain genome is 70.3%, its approximate size 8.57 Mbp, its GenBank deposit SAMN04489726.

#### Emended description of *Allonocardiopsis opalescens* Du et al. 2013

The description is as before (Du et al., 2013) with the following modification. The G+C content of the type-strain genome is 73.9%, its approximate size 6.97 Mbp, its IMG deposit 2728369266.

#### Emended description of *Alloscardovia criceti* (Okamoto et al. 2007) Killer et al. 2013

The description is as before (Killer et al., 2013) with the following modification. The G+C content of the type-strain genome is 50.1%, its approximate size 1.88 Mbp, its GenBank deposit SAMN02441737.

#### Emended description of *Alloscardovia omnicolens* Huys et al. 2007

The description is as before (Huys et al., 2007) with the following modification. The G+C content of the type-strain genome is 46.6%, its approximate size 1.85 Mbp, its GenBank deposit SAMN02441248.

#### Emended description of *Amycolatopsis alba* Mertz and Yao 1993

The description is as before (Mertz and Yao, 1993) with the following addition. The G+C content of the type-strain genome is 68.7%, its approximate size 9.81 Mbp, its GenBank deposit SAMN02261260.

#### Emended description of *Amycolatopsis australiensis* Tan et al. 2006

The description is as before (Tan et al., 2006) with the following addition. The G+C content of the type-strain genome is 71.9%, its approximate size 9.31 Mbp, its GenBank deposit SAMN04489730.

#### Emended description of *Amycolatopsis azurea* (Omura et al. 1983) Henssen et al. 1987

The description is as before (Henssen et al., 1987) with the following modification. The G+C content of the type-strain genome is 68.9%, its approximate size 9.22 Mbp, its GenBank deposit SAMN01939083.

#### Emended description of *Amycolatopsis balhimycina* Wink et al. 2003

The description is as before (Wink et al., 2003) with the following addition. The G+C content of the type-strain genome is 70.8%, its approximate size 10.86 Mbp, its GenBank deposit SAMN02256403.

#### Emended description of *Amycolatopsis benzoatilytica* Majumdar et al. 2006

The description is as before (Majumdar et al., 2006) with the following addition. The G+C content of the type-strain genome is 70.0%, its approximate size 8.70 Mbp, its GenBank deposit SAMN02261299.

#### Emended description of *Amycolatopsis coloradensis* Labeda 1995

The description is as before (Labeda, 1995) with the following modification. The G+C content of the type-strain genome is 68.4%, its approximate size 9.05 Mbp, its GenBank deposit SAMN04384194.

#### Emended description of *Amycolatopsis decaplanina* Wink et al. 2004

The description is as before (Wink et al., 2004) with the following addition. The G+C content of the type-strain genome is 68.6%, its approximate size 8.53 Mbp, its GenBank deposit SAMN02470008.

#### Emended description of *Amycolatopsis jejuensis* Lee 2006

The description is as before (Lee, 2006a) with the following modification. The G+C content of the type-strain genome is 69.1%, its approximate size 10.10 Mbp, its GenBank deposit SAMN02645327.

#### Emended description of *Amycolatopsis keratiniphila* Al-Musallam et al. 2003

The description is as before (Al-Mussallam et al., 2003) with the following addition. Produces light-gray aerial mycelium where present. The G+C content of the type-strain genome is 69.0%, its approximate size 9.09 Mbp, its GenBank deposit SAMN04384022.

#### Emended description of *Amycolatopsis lurida* (Lechevalier et al., 1986) Stackebrandt et al. 2004

The description is as before (Stackebrandt et al., 2004) with the following modification. The G+C content of the type-strain genome is 68.7%, its approximate size 9.05 Mbp, its GenBank deposit SAMN04489729.

#### Emended description of *Amycolatopsis marina* Bian et al. 2009

The description is as before (Bian et al., 2009) with the following modification. The G+C content of the type-strain genome is 68.2%, its approximate size 7.02 Mbp, its GenBank deposit SAMN05216266.

#### Emended description of *Amycolatopsis mediterranei* (Margalith and Beretta 1960) Lechevalier et al. 1986

The description is as before (Lechevalier et al., 1986) with the following restriction. The G+C content of the type-strain genome is 71.3%, its approximate size 10.24 Mbp, its GenBank deposit SAMN02604164.

#### Emended description of *Amycolatopsis nigrescens* Groth et al. 2007

The description is as before (Groth et al., 2007) with the following addition. The G+C content of the type-strain genome is 70.0%, its approximate size 9.11 Mbp, its GenBank deposit SAMN02261308.

#### Emended description of *Amycolatopsis niigatensis* Ding et al. 2007

The description is as before (Ding et al., 2007) with the following modification. The G+C content of the type-strain genome is 69.5%, its approximate size 9.31 Mbp, its GenBank deposit SAMN04489734.

#### Emended description of *Amycolatopsis orientalis* (Pittenger and Brigham 1956) Lechevalier et al. 1986

The description is as before (Lechevalier et al., 1986) with the following modification. The G+C content of the type-strain genome is 69.0%, its approximate size 9.06 Mbp, its GenBank deposit SAMN02142155.

#### Emended description of *Amycolatopsis pretoriensis* Labeda et al. 2003

The description is as before (Labeda et al., 2003) with the following addition. The G+C content of the type-strain genome is 71.2%, its approximate size 10.30 Mbp, its GenBank deposit SAMN05421837.

#### Emended description of *Amycolatopsis regifaucium* Tan et al. 2007

The description is as before (Tan et al., 2007) with the following addition. The G+C content of the type-strain genome is 68.5%, its approximate size 8.29 Mbp, its GenBank deposit SAMN04489731.

#### Emended description of *Amycolatopsis rifamycinica* Bala et al. 2004

The description is as before (Bala et al., 2004) with the following addition. The G+C content of the type-strain genome is 71.8%, its approximate size 9.20 Mbp, its GenBank deposit SAMN02745639.

#### Emended description of *Amycolatopsis rubida* Huang et al. 2001

The description is as before (Huang et al., 2001) with the following modification. The G+C content of the type-strain genome is 69.8%, its approximate size 9.87 Mbp, its GenBank deposit SAMN05421854.

#### Emended description of *Amycolatopsis saalfeldensis* Carlsohn et al. 2007

The description is as before (Carlsohn et al., 2007) with the following addition. The G+C content of the type-strain genome is 71.0%, its approximate size 9.86 Mbp, its GenBank deposit SAMN04489732.

#### Emended description of *Amycolatopsis sacchari* Goodfellow et al. 2001

The description is as before (Goodfellow et al., 2001) with the following addition. The G+C content of the type-strain genome is 71.3%, its approximate size 7.59 Mbp, its GenBank deposit SAMN05421835.

#### Emended description of *Amycolatopsis sulphurea* Lechevalier et al. 1986

The description is as before (Lechevalier et al., 1986) with the following modification. The G+C content of the type-strain genome is 69.4%, its approximate size 6.86 Mbp, its GenBank deposit SAMN04489728.

#### Emended description of *Amycolatopsis thermoflava* Chun et al. 1999

The description is as before (Chun et al., 1999) with the following modification. The G+C content of the type-strain genome is 71.6%, its approximate size 8.69 Mbp, its GenBank deposit SAMN02441454.

#### Emended description of *Amycolatopsis tolypomycina* Wink et al. 2003

The description is as before (Wink et al., 2003) with the following addition. The G+C content of the type-strain genome is 71.7%, its approximate size 10.36 Mbp, its GenBank deposit SAMN04489727.

#### Emended description of *Amycolatopsis vancoresmycina* Wink et al. 2003

The description is as before (Wink et al., 2003) with the following addition. The G+C content of the type-strain genome is 72.0%, its approximate size 9.84 Mbp, its GenBank deposit SAMN02645326.

#### Emended description of *Amycolatopsis xylanica* Chen et al. 2010

The description is as before (Chen et al., 2010) with the following modification. The G+C content of the type-strain genome is 68.9%, its approximate size 9.41 Mbp, its GenBank deposit SAMN05421504.

#### Emended description of *Anaerolinea thermolimosa* Yamada et al. 2006

The description is as before (Yamada et al., 2006) with the following modification. The G+C content of the type-strain genome is 54.6%, its approximate size 4.10 Mbp, its GenBank deposit SAMD00034885.

#### Emended description of *Antricoccus suffuscus* Lee 2015

The description is as before (Lee, 2015) with the following modification. The G+C content of the type-strain genome is 64.1%, its approximate size 4.68 Mbp, its IMG deposit 2728369516.

#### Emended description of *Arcanobacterium haemolyticum* (ex Mac Lean et al. 1946) Collins et al. 1983

The description is as before (Collins et al., 1982) with the following restriction. The G+C content of the type-strain genome is 53.1%, its approximate size 1.99 Mbp, its GenBank deposit SAMN02598519.

#### Emended description of *Arcanobacterium phocae* Pascual Ramos et al. 1997

The description is as given before (Ramos et al., 1997) with the following addition. The G+C content of the type-strain genome is 50.0%, its approximate size 2.00 Mbp, its GenBank deposit SAMN04489737.

#### Emended description of *Ardenticatena maritima* Kawaichi et al. 2013

The description is as before (Kawaichi et al., 2013) with the following modification. The G+C content of the type-strain genome is 59.6%, its approximate size 3.56 Mbp, its GenBank deposit SAMN03274860.

#### Emended description of *Arthrobacter alpinus* Zhang et al. 2010

The description is as before (Zhang et al., 2010a) with the following modification. The G+C content of the type-strain genome is 60.6%, its approximate size 4.62 Mbp, its GenBank deposit SAMN04489740.

#### Emended description of *Arthrobacter castelli* Heyrman et al. 2005

The description is as before (Heyrman et al., 2005) with the following modification. The G+C content of the type-strain genome is 63.6%, its approximate size 4.58 Mbp, its GenBank deposit SAMN02441477.

#### Emended description of *Arthrobacter crystallopoietes* Ensign and Rittenberg 1963

The description is as before (Ensign and Rittenberg, 1963) with the following addition. The G+C content of the type-strain genome is 64.4%, its approximate size 5.20 Mbp, its GenBank deposit SAMN04489742.

#### Emended description of *Arthrobacter cupressi* Zhang et al. 2012

The description is as before (Zhang et al., 2012) with the following modification. The G+C content of the type-strain genome is 67.0%, its approximate size 4.06 Mbp, its GenBank deposit SAMN05216555.

#### Emended description of *Arthrobacter enclensis* Dastager et al. 2015

The description is as before (Dastager et al., 2014b) with the following modification. The G+C content of the type-strain genome is 67.1%, its approximate size 4.22 Mbp, its GenBank deposit SAMN04263892.

#### Emended description of *Arthrobacter globiformis* (Conn 1928) Conn and Dimmick 1947

The description is as before (Conn and Dimmick, 1947) with the following addition. The G+C content of the type-strain genome is 66.2%, its approximate size 4.95 Mbp, its GenBank deposit SAMD00041813.

#### Emended description of *Atopobium fossor* (Bailey and Love 1986) Kageyama et al. 1999

The description is as before (Kageyama et al., 1999) with the following modification. The G+C content of the type-strain genome is 45.4%, its approximate size 1.66 Mbp, its GenBank deposit SAMN02440747.

#### Emended description of *Atopobium minutum* (Hauduroy et al. 1937) Collins and Wallbanks 1993

The description is as before (Collins and Wallbanks, 1992) with the following modification. The G+C content of the type-strain genome is 48.7%, its approximate size 1.78 Mbp, its GenBank deposit SAMN04489746.

#### Emended description of *Bellilinea caldifistulae* Yamada et al. 2007

The description is as before (Yamada et al., 2007) with the following modification. The G+C content of the type-strain genome is 52.8%, its approximate size 3.66 Mbp, its GenBank deposit SAMN03842211.

#### Emended description of *Beutenbergia cavernae* Groth et al. 1999

The description is as before (Groth et al., 1999b) with the following modification. The G+C content of the type-strain genome is 73.1%, its approximate size 4.67 Mbp, its GenBank deposit SAMN02598436.

#### Emended description of *Bifidobacterium actinocoloniiforme* Killer et al. 2011

The description is as before (Killer et al., 2011) with the following modification. The G+C content of the type-strain genome is 62.7%, its approximate size 1.83 Mbp, its GenBank deposit SAMN02442035.

#### Emended description of *Bifidobacterium adolescentis* Reuter 1963

The description is as before (Reuter, 1963) with the following addition. The G+C content of the type-strain genome is 59.2%, its approximate size 2.09 Mbp, its GenBank deposit SAMD00061080.

#### Emended description of *Bifidobacterium asteroides* Scardovi and Trovatelli 1969

The description is as before (Scardovi and Trovatelli, 1969) with the following addition. The G+C content of the type-strain genome is 60.0%, its approximate size 2.14 Mbp, its GenBank deposit SAMN02442012.

#### Emended description of *Bifidobacterium bifidum* (Tissier 1900) Orla-Jensen 1924

The description is as given before (Orla-Jensen, 1924) with the following addition. The G+C content of the type-strain genome is 62.7%, its approximate size 2.21 Mbp, its GenBank deposit SAMD00061040.

#### Emended description of *Bifidobacterium bohemicum* Killer et al. 2011

The description is as before (Killer et al., 2011) with the following modification. The G+C content of the type-strain genome is 57.5%, its approximate size 2.05 Mbp, its GenBank deposit SAMN02673427.

#### Emended description of *Bifidobacterium bombi* Killer et al. 2009

The description is as before (Killer et al., 2009) with the following modification. The G+C content of the type-strain genome is 56.1%, its approximate size 1.90 Mbp, its GenBank deposit SAMN02952076.

#### Emended description of *Bifidobacterium breve* Reuter 1963

The description is as before (Reuter, 1963) with the following addition. The G+C content of the type-strain genome is 58.9%, its approximate size 2.27 Mbp, its GenBank deposit SAMD00061041.

#### Emended description of *Bifidobacterium catenulatum* Scardovi and Crociani 1974

The description is as before (Scardovi and Crociani, 1974) with the following modification. The pH range sustaining growth is 6–8. The G+C content of the type-strain genome is 56.2%, its approximate size 2.08 Mbp, its GenBank deposit SAMD00061042.

#### Emended description of *Bifidobacterium coryneforme* (ex Scardovi and Trovatelli, 1969) Biavati et al. 1982

The description is as before (Biavati et al., 1982) with the following addition. The G+C content of the type-strain genome is 60.5%, its approximate size 1.76 Mbp, its GenBank deposit SAMN02666223.

#### Emended description of *Bifidobacterium crudilactis* Delcenserie et al. 2013

The description is as before (Delcenserie et al., 2007) with the following modification. The G+C content of the type-strain genome is 57.7%, its approximate size 2.36 Mbp, its GenBank deposit SAMN02699097.

#### Emended description of *Bifidobacterium cuniculi* Scardovi et al. 1979

The description is as before (Scardovi et al., 1979) with the following modification. The G+C content of the type-strain genome is 64.9%, its approximate size 2.53 Mbp, its GenBank deposit SAMN02673433.

#### Emended description of *Bifidobacterium dentium* Scardovi and Crociani 1974

The description is as before (Scardovi and Crociani, 1974) with the following modification. The G+C content of the type-strain genome is 58.6%, its approximate size 2.67 Mbp, its GenBank deposit SAMN05192536.

#### Emended description of *Bifidobacterium gallicum* Lauer 1990

The description is as before (Lauer, 1990) with the following modification. The G+C content of the type-strain genome is 57.5%, its approximate size 2.02 Mbp, its GenBank deposit SAMN02299421.

#### Emended description of *Bifidobacterium globosum* (ex Scardovi and Trovatelli, 1969) Biavati et al. 1982

The description is as before (Biavati et al., 1982) with the following restriction. The G+C content of the type-strain genome is 63.4%, its approximate size 1.91 Mbp, its GenBank deposit SAMN02743881.

#### Emended description of *Bifidobacterium indicum* Scardovi and Trovatelli 1969

The description is as before (Scardovi and Trovatelli, 1969) with the following addition. The G+C content of the type-strain genome is 60.5%, its approximate size 1.73 Mbp, its GenBank deposit SAMN05771123.

#### Emended description of *Bifidobacterium lemurum* Modesto et al. 2015

The description is as before (Modesto et al., 2015) with the following modification. The G+C content of the type-strain genome is 62.6%, its approximate size 2.91 Mbp, its GenBank deposit SAMD00065376.

#### Emended description of *Bifidobacterium longum* subsp. suis (Matteuzzi et al. 1971) Mattarelli et al. 2008

The description is as before (Mattareli et al., 2008) with the following modification. The G+C content of the type-strain genome is 60.0%, its approximate size 2.39 Mbp, its GenBank deposit SAMN04489749.

#### Emended description of *Bifidobacterium magnum* Scardovi and Zani 1974

The description is as before (Scardovi and Zani, 1974) with the following restriction. The G+C content of the type-strain genome is 58.7%, its approximate size 1.83 Mbp, its GenBank deposit SAMN02441586.

#### Emended description of *Bifidobacterium merycicum* Biavati and Mattarelli 1991

The description is as before (Biavati and Mattarelli, 1991) with the following modification. The G+C content of the type-strain genome is 60.3%, its approximate size 2.28 Mbp, its GenBank deposit SAMN02673440.

#### Emended description of *Bifidobacterium minimum* Biavati et al. 1982

The description is as before (Biavati et al., 1982) with the following addition. The G+C content of the type-strain genome is 62.7%, its approximate size 1.87 Mbp, its GenBank deposit SAMN02440587.

#### Emended description of *Bifidobacterium mongoliense* Watanabe et al. 2009

The description is as before (Watanabe et al., 2009) with the following modification. The G+C content of the type-strain genome is 62.8%, its approximate size 2.17 Mbp, its GenBank deposit SAMN02673442.

#### Emended description of *Bifidobacterium pseudocatenulatum* Scardovi et al. 1979

The description is as before (Scardovi et al., 1979) with the following modification. The G+C content of the type-strain genome is 56.4%, its approximate size 2.31 Mbp, its GenBank deposit SAMD00061045.

#### Emended description of *Bifidobacterium pseudolongum* Mitsuoka 1969

The description is as before (Mitsuoka, 1969) with the following addition. The G+C content of the type-strain genome is 63.1%, its approximate size 1.90 Mbp, its GenBank deposit SAMN02673445.

#### Emended description of *Bifidobacterium pullorum* Trovatelli et al. 1974

The description is as before (Trovatelli et al., 1974) with the following restriction. pH range of growth from 4.5 to 7.3. The G+C content of the type-strain genome is 64.2%, its approximate size 2.15 Mbp, its GenBank deposit SAMN02673447.

#### Emended description of *Bifidobacterium ruminantium* Biavati and Mattarelli 1991

The description is as before (Biavati and Mattarelli, 1991) with the following modification. The G+C content of the type-strain genome is 59.2%, its approximate size 2.22 Mbp, its GenBank deposit SAMN02743880.

#### Emended description of *Bifidobacterium scardovii* Hoyles et al. 2002

The description is as before (Hoyles et al., 2002b) with the following modification. The G+C content of the type-strain genome is 64.6%, its approximate size 3.16 Mbp, its GenBank deposit SAMD00061046.

#### Emended description of *Bifidobacterium stercoris* Kim et al. 2010

The description is as before (Kim et al., 2010) with the following modification. The G+C content of the type-strain genome is 59.4%, its approximate size 2.30 Mbp, its GenBank deposit SAMN02673454.

#### Emended description of *Bifidobacterium subtile* Biavati et al. 1982

The description is as before (Biavati et al., 1982) with the following addition. The G+C content of the type-strain genome is 60.9%, its approximate size 2.77 Mbp, its GenBank deposit SAMN02440843.

#### Emended description of *Bifidobacterium thermacidophilum* Dong et al. 2000 emend. Zhu et al. 2003

The description is as before (Zhu et al., 2003) with the following restriction. The G+C content of the type-strain genome is 60.4%, its approximate size 2.22 Mbp, its GenBank deposit SAMN02441522.

#### Emended description of *Bifidobacterium thermophilum* Mitsuoka 1969

The description is as before (Mitsuoka, 1969) with the following addition. The G+C content of the type-strain genome is 59.9%, its approximate size 2.10 Mbp, its GenBank deposit SAMN02673459.

#### Emended description of *Brachybacterium faecium* Collins et al. 1988

The description is as before (Collins et al., 1988a) with the following modification. The G+C content of the type-strain genome is 72.0%, its approximate size 3.61 Mbp, its GenBank deposit SAMN00002685.

#### Emended description of *Brachybacterium squillarum* Park et al. 2011

The description is as before (Park et al., 2011) with the following modification. The G+C content of the type-strain genome is 72.8%, its approximate size 3.19 Mbp, its GenBank deposit SAMN02470219.

#### Emended description of *Brevibacterium epidermidis* Collins et al. 1983

The description is as before (Collins et al., 1983) with the following modification. The G+C content of the type-strain genome is 64.3%, its approximate size 3.70 Mbp, its GenBank deposit SAMD00046483.

#### Emended description of *Brevibacterium massiliense* Roux and Raoult 2009

The description is as before (Roux and Raoult, 2009) with the following addition. The G+C content of the type-strain genome is 62.3%, its approximate size 2.35 Mbp, its GenBank deposit SAMEA3138417.

#### Emended description of *Brevibacterium mcbrellneri* McBride et al. 1994

The description is as before (McBride et al., 1993) with the following modification. The G+C content of the type-strain genome is 58.0%, its approximate size 2.56 Mbp, its GenBank deposit SAMN00139181.

#### Emended description of *Brevibacterium sandarakinum* Kämpfer et al. 2010

The description is as before (Kämpfer et al., 2010b) with the following addition. The G+C content of the type-strain genome is 63.3%, its approximate size 4.44 Mbp, its GenBank deposit SAMN04489751.

#### Emended description of *Caldilinea aerophila* Sekiguchi et al. 2003

The description is as before (Sekiguchi et al., 2003) with the following addition. The G+C content of the type-strain genome is 58.8%, its approximate size 5.14 Mbp, its GenBank deposit SAMN00707155.

#### Emended description of *Catelliglobosispora koreensis* (Lee et al., 2000) Ara et al. 2008

The description is as before (Ara et al., 2008a) with the following modification. The G+C content of the type-strain genome is 64.4%, its approximate size 7.69 Mbp, its GenBank deposit SAMN02256447.

#### Emended description of *Catenulispora acidiphila* Busti et al. 2006

The description is as before (Busti et al., 2006) with the following modification. The G+C content of the type-strain genome is 69.8%, its approximate size 10.47 Mbp, its GenBank deposit SAMN02598445.

#### Emended description of *Cellulomonas carbonis* Shi et al. 2012

The description is as before (Shi et al., 2012) with the following modification. The G+C content of the type-strain genome is 73.0%, its approximate size 3.98 Mbp, its GenBank deposit SAMN03113093.

#### Emended description of *Cellulomonas cellasea* (Kellerman et al. 1913) Bergey et al. 1923

The description is as before (Bergey et al., 1923) with the following addition. The G+C content of the type-strain genome is 74.6%, its approximate size 3.91 Mbp, its GenBank deposit SAMN03112937.

#### Emended description of *Cellulomonas fimi* (McBeth and Scales 1913) Bergey et al. 1923

The description is as before (Bergey et al., 1923) with the following addition. The G+C content of the type-strain genome is 74.7%, its approximate size 4.27 Mbp, its GenBank deposit SAMN00713615.

#### Emended description of *Cellulomonas flavigena* (Kellerman and McBeth 1912) Bergey et al. 1923

The description is as before (Bergey et al., 1923) with the following addition. The G+C content of the type-strain genome is 74.3%, its approximate size 4.12 Mbp, its GenBank deposit SAMN02598424.

#### Emended description of *Cellulomonas marina* Zhang et al. 2013

The description is as before (Zhang et al., 2013a) with the following modification. The G+C content of the type-strain genome is 75.6%, its approximate size 3.96 Mbp, its GenBank deposit SAMN05421867.

#### Emended description of *Cellulomonas massiliensis* Lagier et al. 2015

The description is as before (Lagier et al., 2012) with the following modification. The G+C content of the type-strain genome is 74.8%, its GenBank deposit SAMEA2272357.

#### Emended description of *Chloroflexus aurantiacus* Pierson and Castenholz 1974

The description is as before (Pierson and Castenholz, 1974) with the following restriction. The G+C content of the type-strain genome is 56.7%, its approximate size 5.26 Mbp, its GenBank deposit SAMN02598539.

#### Emended description of *Clavibacter michiganensis* (Smith 1910) Davis et al. 1984

The description is as before (Davis et al., 1984) with the following addition. The G+C content of the type-strain genome is 72.7%, its approximate size 3.30 Mbp, its GenBank deposit SAMN06265879.

#### Emended description of *Cnuibacter physcomitrellae* Zhou et al. 2016

The description is as before (Zhou et al., 2016) with the following modification. The G+C content of the type-strain genome is 70.8%, its approximate size 4.35 Mbp, its GenBank deposit SAMN06622498.

#### Emended description of *Collinsella intestinalis* Kageyama and Benno 2000

The description is as before (Kageyama and Benno, 2000) with the following modification. The G+C content of the type-strain genome is 62.5%, its approximate size 1.80 Mbp, its GenBank deposit SAMN00008813.

#### Emended description of *Collinsella stercoris* Kageyama and Benno 2000

The description is as before (Kageyama and Benno, 2000) with the following modification. The G+C content of the type-strain genome is 63.2%, its approximate size 2.40 Mbp, its GenBank deposit SAMN00008814.

#### Emended description of *Compostimonas suwonensis* Kim et al. 2012

The description is as before (Kim et al., 2012b) with the following modification. The G+C content of the type-strain genome is 69.3%, its approximate size 3.88 Mbp, its IMG deposit 2731639183.

#### Emended description of *Conexibacter woesei* Monciardini et al. 2003

The description is as before (Monciardini et al., 2003) with the following modification. The G+C content of the type-strain genome is 72.7%, its approximate size 6.36 Mbp, its GenBank deposit SAMN00002582.

#### Emended description of *Corynebacterium accolens* Neubauer et al. 1991

The description is as before (Neubauer et al., 1991) with the following modification. The G+C content of the type-strain genome is 59.7%, its approximate size 2.41 Mbp, its GenBank deposit SAMN00002226.

#### Emended description of *Corynebacterium afermentans* Riegel et al. 1993

The description is as before (Riegel et al., 1993) with the following modification. The G+C content of the type-strain genome is 64.9%, its approximate size 2.33 Mbp, its GenBank deposit SAMN05421802.

#### Emended description of *Corynebacterium ammoniagenes* (Cooke and Keith 1927) Collins 1987

The description is as before (Collins, 1987) with the following restriction. The G+C content of the type-strain genome is 55.6%, its approximate size 2.76 Mbp, its GenBank deposit SAMN00189098.

#### Emended description of *Corynebacterium appendicis* Yassin et al. 2002

The description is as before (Yassin et al., 2002a) with the following modification. The G+C content of the type-strain genome is 64.3%, its approximate size 2.25 Mbp, its GenBank deposit SAMN05444817.

#### Emended description of *Corynebacterium aquilae* Fernández-Garayzábal et al. 2003

The description is as given before (Fernández-Garayzábal et al., 2003) with the following addition. The G+C content of the type-strain genome is 60.9%, its approximate size 2.93 Mbp, its GenBank deposit SAMN02996496.

#### Emended description of *Corynebacterium argentoratense* Riegel et al. 1995

The description is as before (Riegel et al., 1995a) with the following modification. The G+C content of the type-strain genome is 58.9%, its approximate size 2.03 Mbp, its GenBank deposit SAMN02603032.

#### Emended description of *Corynebacterium atypicum* Hall et al. 2003

The description is as before (Hall et al., 2003a) with the following addition. The G+C content of the type-strain genome is 65.4%, its approximate size 2.36 Mbp, its GenBank deposit SAMN02911287.

#### Emended description of *Corynebacterium auriscanis* Collins et al. 2000

The description is as before (Collins et al., 1999) with the following modification. The G+C content of the type-strain genome is 58.5%, its approximate size 2.57 Mbp, its GenBank deposit SAMN03106126.

#### Emended description of *Corynebacterium callunae* (Lee and Good 1963) Yamada and Komagata 1972

The description is as before (Yamada and Komagata, 1972) with the following restriction. The G+C content of the type-strain genome is 52.4%, its approximate size 2.89 Mbp, its GenBank deposit SAMN02441249.

#### Emended description of *Corynebacterium camporealensis* Fernández-Garayzábal et al. 1998

The description is as before (Fernández-Garayzábal et al., 1998) with the following addition. The G+C content of the type-strain genome is 59.4%, its approximate size 2.45 Mbp, its GenBank deposit SAMN03365263.

#### Emended description of *Corynebacterium capitovis* Collins et al. 2001

The description is as before (Collins et al., 2001a) with the following addition. The G+C content of the type-strain genome is 64.5%, its approximate size 1.96 Mbp, its GenBank deposit SAMN02256495.

#### Emended description of *Corynebacterium casei* Brennan et al. 2001

The description is as before (Brennan et al., 2001a) with the following modification. The G+C content of the type-strain genome is 55.7%, its approximate size 3.13 Mbp, its GenBank deposit SAMN03081454.

#### Emended description of *Corynebacterium caspium* Collins et al. 2004

The description is as before (Collins et al., 2004a) with the following addition. The G+C content of the type-strain genome is 49.7%, its approximate size 1.84 Mbp, its GenBank deposit SAMN02256419.

#### Emended description of *Corynebacterium ciconiae* Fernández-Garayzábal et al. 2004

The description is as before (Fernández-Garyzábal et al., 2004) with the following addition. The G+C content of the type-strain genome is 62.1%, its approximate size 2.55 Mbp, its GenBank deposit SAMN02256507.

#### Emended description of *Corynebacterium cystitidis* Yanagawa and Honda 1978

The description is as before (Yanagawa and Honda, 1978) with the following modification. The G+C content of the type-strain genome is 57.0%, its approximate size 2.94 Mbp, its GenBank deposit SAMN05661109.

#### Emended description of *Corynebacterium deserti* Zhou et al. 2012

The description is as before (Zhou et al., 2012b) with the following modification. The G+C content of the type-strain genome is 55.3%, its approximate size 3.03 Mbp, its GenBank deposit SAMN02950576.

#### Emended description of *Corynebacterium diphtheriae* (Kruse 1886) Lehmann and Neumann 1896

The description is as before (Lehmann and Neumann, 1896) with the following addition. The G+C content of the type-strain genome is 53.5%, its approximate size 2.46 Mbp, its GenBank deposit SAMEA2517360.

#### Emended description of *Corynebacterium doosanense* Lee et al. 2009

The description is as before (Lee et al., 2009) with the following modification. The G+C content of the type-strain genome is 66.9%, its approximate size 2.65 Mbp, its GenBank deposit SAMN02256506.

#### Emended description of *Corynebacterium efficiens* Fudou et al. 2002

The description is as before (Fudou et al., 2002) with the following restriction. The G+C content of the type-strain genome is 63.0%, its approximate size 3.22 Mbp, its GenBank deposit SAMD00061103.

#### Emended description of *Corynebacterium epidermidicanis* Frischmann et al. 2012

The description is as before (Frischmann et al., 2012) with the following addition. The G+C content of the type-strain genome is 58.1%, its approximate size 2.69 Mbp, its GenBank deposit SAMN03462986.

#### Emended description of *Corynebacterium falsenii* Sjödén et al. 1998

The description is as before (Sjödén et al., 1998) with the following addition. The G+C content of the type-strain genome is 63.2%, its approximate size 2.72 Mbp, its GenBank deposit SAMN02641485.

#### Emended description of *Corynebacterium flavescens* Barksdale et al. 1979

The description is as before (Barksdale et al., 1979) with the following modification. The G+C content of the type-strain genome is 59.9%, its approximate size 2.76 Mbp, its GenBank deposit SAMN02996497.

#### Emended description of *Corynebacterium frankenforstense* Wiertz et al. 2013

The description is as before (Wiertz et al., 2013) with the following addition. The G+C content of the type-strain genome is 71.5%, its approximate size 2.60 Mbp, its GenBank deposit SAMN02991553.

#### Emended description of *Corynebacterium freiburgense* Funke et al. 2009

The description is as before (Funke et al., 2009) with the following addition. The G+C content of the type-strain genome is 49.8%, its approximate size 2.91 Mbp, its GenBank deposit SAMN02441205.

#### Emended description of *Corynebacterium glucuronolyticum* Funke et al. 1995

The description is as before (Funke et al., 1995) with the following modification. The G+C content of the type-strain genome is 59.1%, its approximate size 2.82 Mbp, its GenBank deposit SAMN02850907.

#### Emended description of *Corynebacterium glutamicum* (Kinoshita et al. 1958) Abe et al. 1967

The description is as before (Abe et al., 1967) with the following addition. The G+C content of the type-strain genome is 53.8%, its approximate size 3.28 Mbp, its GenBank deposit SAMEA3138338.

#### Emended description of *Corynebacterium halotolerans* Chen et al. 2004

The description is as before (Chen et al., 2004) with the following modification. The G+C content of the type-strain genome is 68.3%, its approximate size 3.22 Mbp, its GenBank deposit SAMN02603027.

#### Emended description of *Corynebacterium humireducens* Wu et al. 2011

The description is as before (Wu et al., 2011) with the following modification. The G+C content of the type-strain genome is 68.6%, its approximate size 2.68 Mbp, its GenBank deposit SAMN03283197.

#### Emended description of *Corynebacterium imitans* Funke et al. 1997

The description is as before (Funke et al., 1997b) with the following modification. The G+C content of the type-strain genome is 64.3%, its approximate size 2.57 Mbp, its GenBank deposit SAMN02950575.

#### Emended description of *Corynebacterium jeikeium* Jackman et al. 1988

The description is as before (Jackman et al., 1987) with the following modification. The G+C content of the type-strain genome is 61.6%, its approximate size 2.43 Mbp, its GenBank deposit SAMN00001506.

#### Emended description of *Corynebacterium kroppenstedtii* Collins et al. 1998

The description is as before (Collins et al., 1998) with the following modification. The G+C content of the type-strain genome is 57.5%, its approximate size 2.45 Mbp, its GenBank deposit SAMN02603033.

#### Emended description of *Corynebacterium kutscheri* (Migula 1900) Bergey et al. 1925

The description is as before (Bergey et al., 1925) with the following addition. The G+C content of the type-strain genome is 46.5%, its approximate size 2.35 Mbp, its GenBank deposit SAMN03365283.

#### Emended description of *Corynebacterium lactis* Wiertz et al. 2013

The description is as before (Wiertz et al., 2013) with the following addition. The G+C content of the type-strain genome is 60.5%, its approximate size 2.77 Mbp, its GenBank deposit SAMN04012704.

#### Emended description of *Corynebacterium lubricantis* Kämpfer et al. 2009

The description is as before (Kämpfer et al., 2009a) with the following addition. The G+C content of the type-strain genome is 58.6%, its approximate size 2.95 Mbp, its GenBank deposit SAMN02256424.

#### Emended description of *Corynebacterium marinum* Du et al. 2010

The description is as before (Du et al., 2010) with the following modification. The G+C content of the type-strain genome is 67.8%, its approximate size 2.73 Mbp, its GenBank deposit SAMN02800399.

#### Emended description of *Corynebacterium massiliense* Merhej et al. 2009

The description is as before (Merhej et al., 2009) with the following addition. The G+C content of the type-strain genome is 65.0%, its approximate size 2.18 Mbp, its GenBank deposit SAMN02441587.

#### Emended description of *Corynebacterium mastitidis* Fernandez-Garayzabal et al. 1997 emend. Bernard et al. 2016

The description is as before (Bernard et al., 2016) with the following restriction. The G+C content of the type-strain genome is 69.0%, its approximate size 2.37 Mbp, its GenBank deposit SAMN02441393.

#### Emended description of *Corynebacterium matruchotii* (Mendel 1919) Collins 1983

The description is as before (Collins, 1982b) with the following restriction. The G+C content of the type-strain genome is 57.1%, its approximate size 2.86 Mbp, its GenBank deposit SAMN00001942.

#### Emended description of *Corynebacterium minutissimum* (ex Sarkany et al. 1962) Collins and Jones 1983 emend. Yassin et al. 2002

The description is as before (Yassin et al., 2002b) with the following modification. The G+C content of the type-strain genome is 60.0%, its approximate size 2.66 Mbp, its GenBank deposit SAMN03140311.

#### Emended description of *Corynebacterium mooreparkense* Brennan et al. 2001

The description is as before (Brennan et al., 2001a) with the following modification. The G+C content of the type-strain genome is 67.1%, its approximate size 3.43 Mbp, its GenBank deposit SAMN02603088.

#### Emended description of *Corynebacterium mustelae* Funke et al. 2010

The description is as before (Funke et al., 2010b) with the following addition. The G+C content of the type-strain genome is 52.6%, its approximate size 3.47 Mbp, its GenBank deposit SAMN03568800.

#### Emended description of *Corynebacterium mycetoides* (ex Castellani 1942) Collins 1983

The description is as before (Collins, 1982a) with the following modification. The G+C content of the type-strain genome is 66.6%, its approximate size 2.27 Mbp, its GenBank deposit SAMN04488535.

#### Emended description of *Corynebacterium nigricans* Shukla et al. 2004

The description is as before (Shukla et al., 2003) with the following addition. The G+C content of the type-strain genome is 60.6%, its approximate size 2.82 Mbp, its GenBank deposit SAMN02603064.

#### Emended description of *Corynebacterium nuruki* Shin et al. 2011

The description is as before (Shin et al., 2011) with the following modification. The G+C content of the type-strain genome is 69.5%, its approximate size 3.11 Mbp, its GenBank deposit SAMN02470217.

#### Emended description of *Corynebacterium pseudodiphtheriticum* Lehmann and Neumann 1896

The description is as before (Lehmann and Neumann, 1896) with the following addition. The G+C content of the type-strain genome is 55.3%, its approximate size 2.26 Mbp, its GenBank deposit SAMN02743909.

#### Emended description of *Corynebacterium pseudotuberculosis* (Buchanan 1911) Eberson 1918

The description is as before (Eberson, 1918) with the following addition. The G+C content of the type-strain genome is 52.2%, its approximate size 2.34 Mbp, its GenBank deposit SAMN06701041.

#### Emended description of *Corynebacterium renale* (Migula 1900) Ernst 1906

The description is as before (Ernst, 1906) with the following addition. The G+C content of the type-strain genome is 59.1%, its approximate size 2.35 Mbp, its GenBank deposit SAMN04488536.

#### Emended description of *Corynebacterium resistens* Otsuka et al. 2005

The description is as before (Otsuka et al., 2005) with the following modification. The G+C content of the type-strain genome is 57.1%, its approximate size 2.60 Mbp, its GenBank deposit SAMN02603065.

#### Emended description of *Corynebacterium seminale* Riegel et al. 1996

The description is as before (Riegel et al., 1995b) with the following modification. The G+C content of the type-strain genome is 59.0%, its approximate size 2.85 Mbp, its GenBank deposit SAMN00001463.

#### Emended description of *Corynebacterium singulare* Riegel et al. 1997

The description is as before (Riegel et al., 1997) with the following modification. The G+C content of the type-strain genome is 60.1%, its approximate size 2.83 Mbp, its GenBank deposit SAMN03177398.

#### Emended description of *Corynebacterium sphenisci* Goyache et al. 2003

The description is as before (Goyache et al., 2003a) with the following addition. The G+C content of the type-strain genome is 74.7%, its approximate size 2.59 Mbp, its GenBank deposit SAMN02996499.

#### Emended description of *Corynebacterium spheniscorum* Goyache et al. 2003

The description is as before (Goyache et al., 2003b) with the following addition. The G+C content of the type-strain genome is 57.5%, its approximate size 2.45 Mbp, its GenBank deposit SAMN05660282.

#### Emended description of *Corynebacterium sputi* Yassin and Siering 2008

The description is as before (Yassin and Siering, 2008) with the following addition. The G+C content of the type-strain genome is 61.5%, its approximate size 2.92 Mbp, its GenBank deposit SAMN02441223.

#### Emended description of *Corynebacterium striatum* (Chester 1901) Eberson 1918

The description is as before (Eberson, 1918) with the following addition. The G+C content of the type-strain genome is 59.4%, its approximate size 2.72 Mbp, its GenBank deposit SAMN00001507.

#### Emended description of *Corynebacterium testudinoris* Collins et al. 2001

The description is as before (Collins et al., 2001b) with the following addition. The G+C content of the type-strain genome is 63.1%, its approximate size 2.72 Mbp, its GenBank deposit SAMN03480629.

#### Emended description of *Corynebacterium timonense* Merhej et al. 2009

The description is as before (Merhej et al., 2009) with the following addition. The G+C content of the type-strain genome is 66.6%, its approximate size 2.63 Mbp, its GenBank deposit SAMN04488539.

#### Emended description of *Corynebacterium ulceribovis* Yassin 2009

The description is as before (Yassin, 2009) with the following addition. The G+C content of the type-strain genome is 59.2%, its approximate size 2.30 Mbp, its GenBank deposit SAMN02256494.

#### Emended description of *Corynebacterium urealyticum* Pitcher et al. 1992

The description is as before (Pitcher et al., 1992) with the following modification. The G+C content of the type-strain genome is 64.2%, its approximate size 2.37 Mbp, its GenBank deposit SAMEA3138282.

#### Emended description of *Corynebacterium ureicelerivorans* Yassin 2007

The description is as before (Yassin, 2007) with the following addition. The G+C content of the type-strain genome is 65.0%, its approximate size 2.33 Mbp, its GenBank deposit SAMN02953970.

#### Emended description of *Corynebacterium uterequi* Hoyles et al. 2013

The description is as before (Hoyles et al., 2013) with the following addition. The G+C content of the type-strain genome is 65.5%, its approximate size 2.42 Mbp, its GenBank deposit SAMN03480647.

#### Emended description of *Corynebacterium xerosis* (Lehmann and Neumann, 1896) Lehmann and Neumann 1899

The description is as before (Lehmann and Neumann, 1899) with the following addition. The G+C content of the type-strain genome is 69.7%, its approximate size 2.69 Mbp, its GenBank deposit SAMD00046521.

#### Emended description of *Cryobacterium luteum* Liu et al. 2012

The description is as before (Liu et al., 2012) with the following modification. The G+C content of the type-strain genome is 65.1%, its approximate size 3.83 Mbp, its GenBank deposit SAMN05216281.

#### Emended description of *Cryobacterium psychrotolerans* Zhang et al. 2007

The description is as before (Zhang et al., 2007a) with the following modification. The G+C content of the type-strain genome is 68.3%, its approximate size 3.25 Mbp, its GenBank deposit SAMN05216282.

#### Emended description of *Cryptosporangium arvum* Tamura et al. 1998

The description is as before (Tamura et al., 1998) with the following modification. The G+C content of the type-strain genome is 71.7%, its approximate size 9.20 Mbp, its GenBank deposit SAMN02849401.

#### Emended description of *Cryptosporangium aurantiacum* (ex Ruan et al. 1976) Tamura and Hatano 2001

The description is as before (Tamura and Hatano, 2001) with the following addition. The G+C content of the type-strain genome is 71.2%, its approximate size 9.58 Mbp, its GenBank deposit SAMN05443668.

#### Emended description of *Cutibacterium acnes* (Gilchrist 1900) Scholz and Kilian 2016

The description is as before (Scholz and Kilian, 2016) with the following addition. The G+C content of the type-strain genome is 60.1%, its approximate size 2.48 Mbp, its GenBank deposit SAMN02440711.

#### Emended description of *Cutibacterium avidum* (Eggerth 1935) Scholz and Kilian 2016

The description is as before (Scholz and Kilian, 2016) with the following addition. The G+C content of the type-strain genome is 63.4%, its approximate size 2.53 Mbp, its GenBank deposit SAMN02299449.

#### Emended description of *Cutibacterium granulosum* (Prévot 1938) Scholz and Kilian 2016

The description is as before (Scholz and Kilian, 2016) with the following addition. The G+C content of the type-strain genome is 64.2%, its approximate size 3.52 Mbp, its GenBank deposit SAMN02798018.

#### Emended description of *Dactylosporangium aurantiacum* Thiemann et al. 1967

The description is as before (Thiemann et al., 1967) with the following addition. The G+C content of the type-strain genome is 73.2%, its approximate size 11.45 Mbp, its GenBank deposit SAMN02645310.

#### Emended description of *Dehalococcoides mccartyi* Löffler et al. 2013

The description is as before (Löffler et al., 2013) with the following restriction. The G+C content of the type-strain genome is 48.9%, its GenBank deposit SAMN02603990.

#### Emended description of *Dehalogenimonas lykanthroporepellens* Moe et al. 2009

The description is as before (Moe et al., 2009) with the following modification. The G+C content of the type-strain genome is 55.0%, its approximate size 1.69 Mbp, its GenBank deposit SAMN02598529.

#### Emended description of *Demequina oxidasica* Ue et al. 2011

The description is as before (Ue et al., 2011) with the following modification. The G+C content of the type-strain genome is 64.1%, its approximate size 2.62 Mbp, its GenBank deposit SAMD00022567.

#### Emended description of *Demequina salsinemoris* Matsumoto et al. 2010

The description is as before (Matsumoto et al., 2010) with the following restriction. The G+C content of the type-strain genome is 70.2%, its approximate size 3.21 Mbp, its GenBank deposit SAMD00022568.

#### Emended description of *Demetria terragena* Groth et al. 1997

The description is as before (Groth et al., 1997) with the following modification. The G+C content of the type-strain genome is 64.7%, its approximate size 3.57 Mbp, its GenBank deposit SAMN02440416.

#### Emended description of *Dermabacter hominis* Jones and Collins 1989

The description is as before (Jones and Collins, 1988) with the following modification. The G+C content of the type-strain genome is 63.2%, its approximate size 2.16 Mbp, its GenBank deposit SAMD00046478.

#### Emended description of *Dermatophilus congolensis* (Van Saceghem 1915) Gordon 1964

The description is as before (Gordon, 1964) with the following addition. The G+C content of the type-strain genome is 59.4%, its approximate size 2.62 Mbp, its GenBank deposit SAMN02440871.

#### Emended description of *Diaminobutyricimonas aerilata* Jang et al. 2013

The description is as before (Jang et al., 2012) with the following modification. The G+C content of the type-strain genome is 70.7%, its approximate size 3.49 Mbp, its IMG deposit 2731639227.

#### Emended description of *Dietzia alimentaria* Kim et al. 2011

The description is as before (Kim et al., 2011) with the following modification. The G+C content of the type-strain genome is 67.3%, its approximate size 3.35 Mbp, its GenBank deposit SAMN02470218.

#### Emended description of *Dietzia cinnamea* Yassin et al. 2006

The description is as before (Yassin et al., 2006) with the following modification. The G+C content of the type-strain genome is 70.8%, its approximate size 3.60 Mbp, its GenBank deposit SAMD00046472.

#### Emended description of *Dietzia kunjamensis* Mayilraj et al. 2006

The description is as before (Mayilraj et al., 2006a) with the following modification and addition. Tolerates up to 15% NaCl, grows at temperatures between 10 and 45°C and from pH 6–10. Main fatty acids are C_18:1_ ω9c, C_16:0_ and 10-methyl-C_18:0_ (tuberculostearic acid). The G+C content of the type-strain genome is 70.6%, its approximate size 3.54 Mbp, its IMG deposit 2728369259.

#### Emended description of *Dietzia maris* (Nesterenko et al. 1982) Rainey et al. 1995

The description is as before (Rainey et al., 1995b) with the following modification. The G+C content of the type-strain genome is 70.9%, its approximate size 3.51 Mbp, its GenBank deposit SAMN04543680.

#### Emended description of *Eggerthella lenta* (Eggerth 1935) Wade et al. 1999 emend. Maruo et al. 2008

The description is as before (Maruo et al., 2008) with the following modification. The G+C content of the type-strain genome is 64.2%, its approximate size 3.63 Mbp, its GenBank deposit SAMN00002594.

#### Emended description of *Falcivibrio vaginalis* Hammann et al. 1984

The description is as before (Hammann et al., 1984) with the following modification. The G+C content of the type-strain genome is 55.4%, its approximate size 2.15 Mbp, its GenBank deposit SAMN00001505.

#### Emended description of *Ferrithrix thermotolerans* Johnson et al. 2009

The description is as before (Johnson et al., 2009) with the following restriction. The G+C content of the type-strain genome is 51.1%, its approximate size 2.49 Mbp, its GenBank deposit SAMN02745225.

#### Emended description of *Gardnerella vaginalis* (Gardner and Dukes 1955) Greenwood and Pickett 1980

The description is as before (Greenwood and Pickett, 1980) with the following restriction. The G+C content of the type-strain genome is 41.3%, its approximate size 1.68 Mbp, its GenBank deposit SAMN04488545.

#### Emended description of *Georgenia soli* Kämpfer et al. 2010

The description is as before (Kämpfer et al., 2010a) with the following addition. The G+C content of the type-strain genome is 72.7%, its approximate size 4.27 Mbp, its GenBank deposit SAMN04488547.

#### Emended description of *Glaciibacter superstes* Katayama et al. 2009

The description is as before (Katayama et al., 2009) with the following modification. The G+C content of the type-strain genome is 64.4%, its approximate size 4.80 Mbp, its GenBank deposit SAMN02440580.

#### Emended description of *Glutamicibacter arilaitensis* (Irlinger et al. 2005) Busse 2016

The description is as before (Busse, 2016) with the following addition. The G+C content of the type-strain genome is 59.3%, its approximate size 3.92 Mbp, its GenBank deposit SAMEA2272213.

#### Emended description of *Glutamicibacter mysorens* (Nand and Rao 1972) Busse 2016

The description is as before (Busse, 2016) with the following addition. The G+C content of the type-strain genome is 62.0%, its approximate size 3.46 Mbp, its GenBank deposit SAMN04489744.

#### Emended description of *Glycomyces arizonensis* Labeda and Kroppenstedt 2004

The description is as before (Labeda and Kroppenstedt, 2004) with the following addition. The G+C content of the type-strain genome is 70.1%, its approximate size 5.05 Mbp, its GenBank deposit SAMN02441107.

#### Emended description of *Glycomyces sambucus* Gu et al. 2007

The description is as before (Gu et al., 2007) with the following modification. The G+C content of the type-strain genome is 71.8%, its approximate size 5.60 Mbp, its GenBank deposit SAMN05216298.

#### Emended description of *Glycomyces tenuis* Evtushenko et al. 1991

The description is as before (Evtushenko et al., 1991) with the following modification. The G+C content of the type-strain genome is 70.4%, its approximate size 5.73 Mbp, its GenBank deposit SAMN02441561.

#### Emended description of *Goodfellowiella coeruleoviolacea* (Preobrazhenskaya and Terekhova 1987) Labeda et al. 2008

The description is as before (Labeda et al., 2008) with the following modification. The G+C content of the type-strain genome is 72.4%, its approximate size 8.77 Mbp, its GenBank deposit SAMN05660962.

#### Emended description of *Gordonia amarae* (Lechevalier and Lechevalier 1974) Klatte et al. 1994

The description is as before (Klatte et al., 1994) with the following restriction. The G+C content of the type-strain genome is 67.4%, its approximate size 5.31 Mbp, its GenBank deposit SAMD00041822.

#### Emended description of *Gordonia amicalis* Kim et al. 2000

The description is as before (Kim et al., 2000) with the following modification. The G+C content of the type-strain genome is 67.4%, its approximate size 4.92 Mbp, its GenBank deposit SAMD00041764.

#### Emended description of *Gordonia araii* Kageyama et al. 2006

The description is as before (Kageyama et al., 2006) with the following addition. The G+C content of the type-strain genome is 68.0%, its approximate size 3.91 Mbp, its GenBank deposit SAMD00041783.

#### Emended description of *Gordonia bronchialis* (Tsukamura, 1971) Stackebrandt et al. 1989

The description is as before (Stackebrandt et al., 1988) with the following addition. The G+C content of the type-strain genome is 67.1%, its approximate size 5.21 Mbp, its GenBank deposit SAMN00002600.

#### Emended description of *Gordonia desulfuricans* Kim et al. 1999

The description is as before (Kim et al., 1999) with the following modification. The G+C content of the type-strain genome is 68.1%, its approximate size 5.43 Mbp, its GenBank deposit SAMD00018679.

#### Emended description of *Gordonia effusa* Kageyama et al. 2006

The description is as before (Kageyama et al., 2006) with the following addition. The G+C content of the type-strain genome is 62.5%, its approximate size 4.70 Mbp, its GenBank deposit SAMD00041782.

#### Emended description of *Gordonia hydrophobica* Bendinger et al. 1995

The description is as before (Bendinger et al., 1995) with the following modification. The G+C content of the type-strain genome is 67.5%, its approximate size 4.58 Mbp, its GenBank deposit SAMD00047212.

#### Emended description of *Gordonia lacunae* Le Roes et al. 2009

The description is as before (le Roes et al., 2008) with the following addition. The G+C content of the type-strain genome is 68.1%, its approximate size 5.76 Mbp, its GenBank deposit SAMN06928587.

#### Emended description of *Gordonia malaquae* Yassin et al. 2007

The description is as before (Yassin et al., 2007a) with the following addition. The G+C content of the type-strain genome is 66.2%, its approximate size 4.71 Mbp, its GenBank deposit SAMN04488550.

#### Emended description of *Gordonia neofelifaecis* Liu et al. 2011

The description is as before (Liu et al., 2011) with the following addition. The G+C content of the type-strain genome is 68.2%, its approximate size 4.26 Mbp, its GenBank deposit SAMN02470983.

#### Emended description of *Gordonia polyisoprenivorans* Linos et al. 1999

The description is as before (Linos et al., 1999) with the following addition. The G+C content of the type-strain genome is 66.9%, its approximate size 6.29 Mbp, its GenBank deposit SAMD00041829.

#### Emended description of *Gordonia rubripertincta* (Hefferan 1904) Stackebrandt et al. 1989

The description is as before (Stackebrandt et al., 1988) with the following restriction. The G+C content of the type-strain genome is 67.4%, its approximate size 5.20 Mbp, its GenBank deposit SAMD00041793.

#### Emended description of *Gordonia shandongensis* Luo et al. 2007

The description is as before (Luo et al., 2007) with the following addition. The G+C content of the type-strain genome is 69.3%, its approximate size 3.33 Mbp, its GenBank deposit SAMN02440656.

#### Emended description of *Gordonia terrae* (Tsukamura, 1971) Stackebrandt et al. 1989

The description is as before (Stackebrandt et al., 1988) with the following addition. The G+C content of the type-strain genome is 67.8%, its approximate size 5.67 Mbp, its GenBank deposit SAMD00041763.

#### Emended description of *Gordonia westfalica* Linos et al. 2002

The description is as before (Linos et al., 2002) with the following addition. The G+C content of the type-strain genome is 66.9%, its approximate size 6.41 Mbp, its GenBank deposit SAMN04488548.

#### Emended description of *Haloglycomyces albus* Guan et al. 2009

The description is as before (Guan et al., 2009) with the following addition. The G+C content of the type-strain genome is 60.4%, its approximate size 3.54 Mbp, its GenBank deposit SAMN02597195.

#### Emended description of *Herbiconiux ginsengi* (Qiu et al. 2007) Behrendt et al. 2011

The description is as before (Behrendt et al., 2011) with the following modification. The G+C content of the type-strain genome is 68.4%, its approximate size 4.85 Mbp, its GenBank deposit SAMN05216554.

#### Emended description of *Herbiconiux solani* Behrendt et al. 2011

The description is as before (Behrendt et al., 2011) with the following addition. The G+C content of the type-strain genome is 70.2%, its approximate size 3.85 Mbp, its GenBank deposit SAMD00046479.

#### Emended description of *Herbidospora cretacea* Kudo et al. 1993

The description is as before (Kudo et al., 1993) with the following restriction. The G+C content of the type-strain genome is 70.7%, its approximate size 8.28 Mbp, its GenBank deposit SAMD00029379.

#### Emended description of *Herbidospora sakaeratensis* Boondaeng et al. 2011

The description is as before (Boondaeng et al., 2011) with the following modification. The G+C content of the type-strain genome is 71.0%, its approximate size 8.58 Mbp, its GenBank deposit SAMD00029380.

#### Emended description of *Herpetosiphon aurantiacus* Holt and Lewin 1968

The description is as before (Holt and Lewin, 1968) with the following restriction. The G+C content of the type-strain genome is 50.9%, its approximate size 6.79 Mbp, its GenBank deposit SAMN00623048.

#### Emended description of *Herpetosiphon geysericola* (Copeland 1936) Lewin 1970

The description is as before (Lewin, 1970) with the following modification. The G+C content of the type-strain genome is 50.7%, its approximate size 6.14 Mbp, its GenBank deposit SAMN03274862.

#### Emended description of *Ilumatobacter nonamiensis* Matsumoto et al. 2013

The description is as before (Matsumoto et al., 2013) with the following modification. The G+C content of the type-strain genome is 67.0%, its approximate size 4.28 Mbp, its GenBank deposit SAMD00036665.

#### Emended description of *Intrasporangium calvum* Kalakoutskii et al. 1967 emend. Yang et al. 2012

The description is as before (Yang et al., 2012) with the following addition. The G+C content of the type-strain genome is 70.7%, its approximate size 4.02 Mbp, its GenBank deposit SAMN00713569.

#### Emended description of *Intrasporangium oryzae* (Kageyama et al. 2007) Yang et al. 2012

The description is as before (Yang et al., 2012) with the following modification. The G+C content of the type-strain genome is 71.4%, its approximate size 4.64 Mbp, its GenBank deposit SAMN02952955.

#### Emended description of *Isoptericola jiangsuensis* Wu et al. 2010

The description is as before (Wu et al., 2010) with the following modification. The G+C content of the type-strain genome is 73.8%, its approximate size 4.04 Mbp, its GenBank deposit SAMN04488560.

#### Emended description of *Janibacter hoylei* Shivaji et al. 2009

The description is as before (Shivaji et al., 2009) with the following modification. The G+C content of the type-strain genome is 71.3%, its approximate size 3.14 Mbp, its GenBank deposit SAMN02470608.

#### Emended description of *Janibacter indicus* Zhang et al. 2014

The description is as before (Zhang et al., 2014) with the following modification. The G+C content of the type-strain genome is 71.2%, its approximate size 3.42 Mbp, its GenBank deposit SAMN06296429.

#### Emended description of *Jatrophihabitans endophyticus* Madhaiyan et al. 2013 emend. Lee et al. 2018

The description is as before (Lee et al., 2018) with the following modification. The G+C content of the type-strain genome is 72.9%, its approximate size 4.48 Mbp, its GenBank deposit SAMN05443575.

#### Emended description of *Jiangella muralis* Kämpfer et al. 2011

The description is as before (Kämpfer et al., 2011) with the following addition. The G+C content of the type-strain genome is 72.3%, its approximate size 7.26 Mbp, its GenBank deposit SAMN03801508.

#### Emended description of *Jonesia denitrificans* (Prévot 1961) Rocourt et al. 1987

The description is as before (Rocourt et al., 1987) with the following addition. The G+C content of the type-strain genome is 58.4%, its approximate size 2.75 Mbp, its GenBank deposit SAMN02598439.

#### Emended description of *Kallotenue papyrolyticum* Cole et al. 2013

The description is as before (Cole et al., 2013) with the following modification. The G+C content of the type-strain genome is 65.8%, its approximate size 4.48 Mbp, its GenBank deposit SAMN02584935.

#### Emended description of *Kibdelosporangium phytohabitans* Xing et al. 2012

The description is as before (Xing et al., 2012) with the following modification. The G+C content of the type-strain genome is 68.4%, its approximate size 11.76 Mbp, its GenBank deposit SAMN03840758.

#### Emended description of *Kineococcus radiotolerans* Phillips et al. 2002

The description is as before (Phillips et al., 2002) with the following addition. The G+C content of the type-strain genome is 74.2%, its approximate size 4.96 Mbp, its GenBank deposit SAMN02598259.

#### Emended description of *Kineococcus xinjiangensis* Liu et al. 2009

The description is as before (Liu et al., 2009a) with the following modification. The G+C content of the type-strain genome is 74.6%, its approximate size 4.62 Mbp, its IMG deposit 2731639219.

#### Emended description of *Kitasatoa kauaiensis* Matsumae et al. 1968

The description is as before (Matsumae et al., 1968) with the following addition. The G+C content of the type-strain genome is 71.5%, its approximate size 7.46 Mbp, its GenBank deposit SAMN02261248.

#### Emended description of *Kitasatospora azatica* (Nakagaito et al. 1993) Zhang et al. 1997

The description is as before (Zhang et al., 1997) with the following modification. The G+C content of the type-strain genome is 71.6%, its approximate size 8.27 Mbp, its GenBank deposit SAMN02745392.

#### Emended description of *Kitasatospora cheerisanensis* Chung et al. 1999

The description is as before (Chung et al., 1999) with the following modification. The G+C content of the type-strain genome is 73.5%, its approximate size 8.04 Mbp, its GenBank deposit SAMN02796068.

#### Emended description of *Kitasatospora mediocidica* Labeda 1988

The description is as before (Labeda, 1988) with the following modification. The G+C content of the type-strain genome is 71.9%, its approximate size 8.68 Mbp, its GenBank deposit SAMN02745411.

#### Emended description of *Kitasatospora phosalacinea* Takahashi et al. 1985

The description is as before (Takahashi et al., 1984) with the following modification. The G+C content of the type-strain genome is 74.1%, its approximate size 7.62 Mbp, its GenBank deposit SAMN02645204.

#### Emended description of *Kitasatospora setae* Omura et al. 1983

The description is as before (Omura et al., 1982) with the following addition. The G+C content of the type-strain genome is 74.2%, its approximate size 8.78 Mbp, its GenBank deposit SAMD00060928.

#### Emended description of *Knoellia aerolata* Weon et al. 2007

The description is as before (Weon et al., 2007) with the following modification. The G+C content of the type-strain genome is 71.3%, its approximate size 4.09 Mbp, its GenBank deposit SAMN03144715.

#### Emended description of *Kocuria flava* Zhou et al. 2008

The description is as before (Zhou et al., 2008) with the following modification. The G+C content of the type-strain genome is 73.9%, its approximate size 3.64 Mbp, its GenBank deposit SAMN04270876.

#### Emended description of *Kocuria indica* Dastager et al. 2014

The description is as before (Dastager et al., 2014c) with the following modification. The G+C content of the type-strain genome is 68.9%, its approximate size 2.88 Mbp, its GenBank deposit SAMN06296028.

#### Emended description of *Kocuria turfanensis* Zhou et al. 2008 emend. Camacho et al. 2017

The description is as before (Camacho et al., 2017) with the following modification. The G+C content of the type-strain genome is 72.8%, its approximate size 4.16 Mbp, its GenBank deposit SAMN04455734.

#### Emended description of *Koreibacter algae* Lee and Lee 2010

The description is as before (Lee and Lee, 2010b) with the following modification. The G+C content of the type-strain genome is 70.7%, its approximate size 2.99 Mbp, its GenBank deposit SAMN04489860.

#### Emended description of *Kribbella catacumbae* (Urz et al., 2008)

The description is as before (Urz et al., 2008) with the following addition. The G+C content of the type-strain genome is 67.5%, its approximate size 9.63 Mbp, its GenBank deposit SAMN02256503.

#### Emended description of *Kutzneria buriramensis* Suriyachadkun et al. 2013

The description is as before (Suriyachadkun et al., 2013) with the following modification. The G+C content of the type-strain genome is 70.4%, its approximate size 11.97 Mbp, its GenBank deposit SAMN05421845.

#### Emended description of *Kytococcus aerolatus* Kämpfer et al. 2009

The description is as before (Kämpfer et al., 2009b) with the following addition. The G+C content of the type-strain genome is 72.3%, its approximate size 2.40 Mbp, its IMG deposit 2734482797.

#### Emended description of *Kytococcus sedentarius* (ZoBell and Upham 1944) Stackebrandt et al. 1995

The description is as before (Stackebrandt et al., 1995) with the following addition. The G+C content of the type-strain genome is 71.6%, its approximate size 2.79 Mbp, its GenBank deposit SAMN02598443.

#### Emended description of *Labedella gwakjiensis* Lee 2007

The description is as before (Lee, 2007) with the following modification. The G+C content of the type-strain genome is 69.2%, its approximate size 3.84 Mbp, its IMG deposit 2734482166.

#### Emended description of *Leifsonia rubra* Reddy et al. 2003

The description is as before (Reddy et al., 2003) with the following modification. The G+C content of the type-strain genome is 59.2%, its approximate size 2.78 Mbp, its GenBank deposit SAMN02469437.

#### Emended description of *Lentzea albida* Labeda et al. 2001

The description is as before (Labeda et al., 2001) with the following addition. The G+C content of the type-strain genome is 70.2%, its approximate size 9.44 Mbp, its GenBank deposit SAMN04488000.

#### Emended description of *Lentzea californiensis* Labeda et al. 2001

The description is as before (Labeda et al., 2001) with the following addition. The G+C content of the type-strain genome is 69.3%, its approximate size 9.00 Mbp, its GenBank deposit SAMN05660743.

#### Emended description of *Lentzea kentuckyensis* Labeda et al. 2007

The description is as before (Labeda et al., 2007) with the following addition. The G+C content of the type-strain genome is 68.8%, its approximate size 10.21 Mbp, its GenBank deposit SAMN06294978.

#### Emended description of *Lentzea waywayandensis* (Labeda and Lyons 1989) Labeda et al. 2001

The description is as before (Labeda et al., 2001) with the following modification. The G+C content of the type-strain genome is 68.9%, its approximate size 10.15 Mbp, its GenBank deposit SAMN04488564.

#### Emended description of *Leptolinea tardivitalis* Yamada et al. 2006

The description is as before (Yamada et al., 2006) with the following modification. The G+C content of the type-strain genome is 46.8%, its approximate size 3.68 Mbp, its GenBank deposit SAMN03856817.

#### Emended description of *Leucobacter chironomi* Halpern et al. 2009

The description is as before (Halpern et al., 2009) with the following addition. The G+C content of the type-strain genome is 69.9%, its approximate size 2.96 Mbp, its GenBank deposit SAMN02441232.

#### Emended description of *Leucobacter chromiiresistens* Sturm et al. 2011

The description is as before (Sturm et al., 2011) with the following addition. The G+C content of the type-strain genome is 70.3%, its approximate size 3.21 Mbp, its GenBank deposit SAMN04488565.

#### Emended description of *Leucobacter salsicius* Yun et al. 2011

The description is as before (Yun et al., 2011) with the following modification. The G+C content of the type-strain genome is 64.5%, its approximate size 3.19 Mbp, its GenBank deposit SAMN02470216.

#### Emended description of *Luteipulveratus halotolerans* Juboi et al. 2015

The description is as before (Juboi et al., 2015) with the following modification. The G+C content of the type-strain genome is 70.1%, its approximate size 4.46 Mbp, its GenBank deposit SAMN03421050.

#### Emended description of *Marmoricola scoriae* Lee and Lee 2010

The description is as before (Lee and Lee, 2010c) with the following modification. The G+C content of the type-strain genome is 73.5%, its approximate size 4.06 Mbp, its GenBank deposit SAMN04488570.

#### Emended description of *Microbacterium agarici* Young et al. 2010

The description is as before (Young et al., 2010) with the following addition. The G+C content of the type-strain genome is 64.5%, its approximate size 3.23 Mbp, its GenBank deposit SAMN04488572.

#### Emended description of *Microbacterium enclense* Mawlankar et al. 2015

The description is as before (Mawlankar et al., 2015) with the following modification. The G+C content of the type-strain genome is 70.4%, its approximate size 3.66 Mbp, its GenBank deposit SAMN04263881.

#### Emended description of *Microbacterium foliorum* Behrendt et al. 2001

The description is as before (Behrendt et al., 2001) with the following modification. The G+C content of the type-strain genome is 68.7%, its approximate size 3.56 Mbp, its GenBank deposit SAMN03256325.

#### Emended description of *Microbacterium gubbeenense* Brennan et al. 2001

The description is as before (Brennan et al., 2001b) with the following restriction. The G+C content of the type-strain genome is 68.1%, its approximate size 3.02 Mbp, its GenBank deposit SAMN02440649.

#### Emended description of *Microbacterium humi* Young et al. 2010

The description is as before (Young et al., 2010) with the following addition. The G+C content of the type-strain genome is 67.3%, its approximate size 3.36 Mbp, its GenBank deposit SAMN04489806.

#### Emended description of *Microbacterium hydrocarbonoxydans* Schippers et al. 2005

The description is as before (Schippers et al., 2005) with the following addition. The G+C content of the type-strain genome is 68.6%, its approximate size 3.59 Mbp, its GenBank deposit SAMN04489807.

#### Emended description of *Microbacterium indicum* Shivaji et al. 2007

The description is as before (Shivaji et al., 2007) with the following modification. The G+C content of the type-strain genome is 71.4%, its approximate size 2.81 Mbp, its GenBank deposit SAMN02440686.

#### Emended description of *Microbacterium luticocti* Vaz-Moreira et al. 2008

The description is as before (Vaz-Moreira et al., 2008) with the following modification. The G+C content of the type-strain genome is 70.7%, its approximate size 3.11 Mbp, its GenBank deposit SAMN02440801.

#### Emended description of *Microbacterium mangrovi* Lee et al. 2014

The description is as before (Lee et al., 2014a) with the following restriction. The G+C content of the type-strain genome is 70.0%, its approximate size 4.42 Mbp, its GenBank deposit SAMN03070121.

#### Emended description of *Microbacterium oleivorans* Schippers et al. 2005

The description is as before (Schippers et al., 2005) with the following addition. The G+C content of the type-strain genome is 69.0%, its approximate size 2.98 Mbp, its GenBank deposit SAMD00046492.

#### Emended description of *Microbacterium resistens* (Funke et al. 1998) Behrendt et al. 2001

The description is as before (Behrendt et al., 2001) with the following addition. The G+C content of the type-strain genome is 71.2%, its approximate size 3.98 Mbp, its GenBank deposit SAMD00046494.

#### Emended description of *Microbacterium trichothecenolyticum* (Yokota et al. 1993) Takeuchi and Hatano 1998

The description is as before (Takeuchi and Hatano, 1998) with the following modification. The G+C content of the type-strain genome is 70.2%, its approximate size 4.52 Mbp, its GenBank deposit SAMN03266141.

#### Emended description of *Microbispora rosea* Nonomura and Ohara 1957

The description is as before (Nonomura and Ohara, 1957) with the following addition. The G+C content of the type-strain genome is 71.2%, its approximate size 8.86 Mbp, its GenBank deposit SAMN05421833.

#### Emended description of *Micrococcus luteus* (Schroeter 1872) Cohn 1872 emend. Wieser et al. 2002

The description is as before (Wieser et al., 2002) with the following addition. The G+C content of the type-strain genome is 73.0%, its approximate size 2.50 Mbp, its GenBank deposit SAMN00000281.

#### Emended description of *Micrococcus lylae* Kloos et al. 1974 emend. Wieser et al. 2002

The description is as before (Wieser et al., 2002) with the following modification. The G+C content of the type-strain genome is 71.3%, its approximate size 2.69 Mbp, its GenBank deposit SAMD00046485.

#### Emended description of *Micrococcus terreus* Zhang et al. 2010

The description is as before (Zhang et al., 2010b) with the following modification. The G+C content of the type-strain genome is 68.9%, its approximate size 3.09 Mbp, its GenBank deposit SAMN04487966.

#### Emended description of *Microlunatus soli* Kämpfer et al. 2010

The description is as before (Kämpfer et al., 2010d) with the following addition. The G+C content of the type-strain genome is 67.4%, its approximate size 6.73 Mbp, its GenBank deposit SAMN04489812.

#### Emended description of *Micromonospora avicenniae* Li et al. 2013

The description is as before (Li et al., 2013) with the following modification. The G+C content of the type-strain genome is 71.5%, its approximate size 6.82 Mbp, its GenBank deposit SAMN05444858.

#### Emended description of *Micromonospora rosaria* (ex Wagman et al. 1972) Horan and Brodsky 1986

The description is as before (Horan and Brodsky, 1986) with the following modification. The G+C content of the type-strain genome is 73.4%, its approximate size 7.38 Mbp, its GenBank deposit SAMN04440589.

#### Emended description of *Micromonospora wenchangensis* Ren et al. 2013

The description is as before (Ren et al., 2013) with the following modification. The G+C content of the type-strain genome is 73.0%, its approximate size 7.51 Mbp, its GenBank deposit SAMN06554453.

#### Emended description of *Microtetraspora fusca* Thiemann et al. 1968

The description is as before (Thiemann et al., 1968) with the following addition. The G+C content of the type-strain genome is 70.9%, its approximate size 9.02 Mbp, its GenBank deposit SAMD00035248.

#### Emended description of *Microtetraspora glauca* Thiemann et al. 1968

The description is as before (Thiemann et al., 1968) with the following addition. The G+C content of the type-strain genome is 70.2%, its approximate size 9.27 Mbp, its GenBank deposit SAMD00035249.

#### Emended description of *Microtetraspora malaysiensis* Nakajima et al. 2004

The description is as before (Nakajima et al., 2003) with the following modification. The G+C content of the type-strain genome is 70.7%, its approximate size 8.82 Mbp, its GenBank deposit SAMD00035251.

#### Emended description of *Microtetraspora niveoalba* Nonomura and Ohara, 1971a

The description is as before (Nonomura and Ohara, 1971a) with the following addition. The G+C content of the type-strain genome is 71.7%, its approximate size 8.47 Mbp, its GenBank deposit SAMD00035250.

#### Emended description of *Mobiluncus curtisii* Spiegel and Roberts 1984 emend. Hoyles et al. 2004

The description is as before (Hoyles et al., 2004) with the following restriction. The G+C content of the type-strain genome is 55.7%, its approximate size 2.14 Mbp, its GenBank deposit SAMN00117214.

#### Emended description of *Mobiluncus mulieris* Spiegel and Roberts 1984 emend. Hoyles et al. 2004

The description is as before (Hoyles et al., 2004) with the following restriction. The G+C content of the type-strain genome is 55.1%, its approximate size 2.40 Mbp, its GenBank deposit SAMN00139192.

#### Emended description of *Motilibacter peucedani* Lee 2012

The description is as before (Lee, 2012) with the following modification. The G+C content of the type-strain genome is 73.6%, its approximate size 4.19 Mbp, its IMG deposit 2731639228.

#### Emended description of *Murinocardiopsis flavida* Kämpfer et al. 2010

The description is as before (Kämpfer et al., 2010c) with the following addition. The G+C content of the type-strain genome is 72.4%, its approximate size 7.38 Mbp, its IMG deposit 2728369519.

#### Emended description of *Mycetocola saprophilus* Tsukamoto et al. 2001

The description is as before (Tsukamoto et al., 2001) with the following modification. The G+C content of the type-strain genome is 66.1%, its approximate size 3.45 Mbp, its GenBank deposit SAMN02645226.

#### Emended description of *Mycobacterium abscessus* subsp. bolletii (Adékambi et al., 2006) Leao et al. 2011 emend. Tortoli et al. 2016

The description is as before (Tortoli et al., 2016a) with the following restriction. The G+C content of the type-strain genome is 64.1%, its approximate size 5.05 Mbp, its GenBank deposit SAMN02470911.

#### Emended description of *Mycobacterium abscessus* subsp. massiliense (Adékambi et al., 2006) Tortoli et al. 2016

The description is as before (Tortoli et al., 2016a) with the following restriction. The G+C content of the type-strain genome is 64.1%, its approximate size 4.98 Mbp, its GenBank deposit AP014547.

#### Emended description of *Mycobacterium algericum* Sahraoui et al. 2011

The description is as before (Sahraoui et al., 2011) with the following addition. The G+C content of the type-strain genome is 68.3%, its approximate size 4.62 Mbp, its GenBank deposit SAMN06064225.

#### Emended description of *Mycobacterium alsense* Tortoli et al. 2016

The description is as before (Tortoli et al., 2016b) with the following addition. The G+C content of the type-strain genome is 69.3%, its approximate size 5.69 Mbp, its GenBank deposit SAMN06064226.

#### Emended description of *Mycobacterium aromaticivorans* Hennessee et al. 2009

The description is as before (Hennessee et al., 2009) with the following addition. The G+C content of the type-strain genome is 66.4%, its approximate size 6.30 Mbp, its GenBank deposit SAMN02585141.

#### Emended description of *Mycobacterium arosiense* Bang et al. 2008

The description is as before (Bang et al., 2008) with the following addition. The G+C content of the type-strain genome is 66.8%, its approximate size 5.98 Mbp, its GenBank deposit SAMN06064229.

#### Emended description of *Mycobacterium arupense* Cloud et al. 2006

The description is as before (Cloud et al., 2006) with the following addition. The G+C content of the type-strain genome is 67.4%, its approximate size 4.44 Mbp, its GenBank deposit SAMN06064230.

#### Emended description of *Mycobacterium asiaticum* Weiszfeiler et al. 1971

The description is as before (Weiszfeiler et al., 1971) with the following addition. The G+C content of the type-strain genome is 66.3%, its approximate size 5.91 Mbp, its GenBank deposit SAMEA3138999.

#### Emended description of *Mycobacterium aurum* Tsukamura 1966

The description is as before (Tsukamura, 1966) with the following addition. The G+C content of the type-strain genome is 67.5%, its approximate size 6.02 Mbp, its GenBank deposit SAMEA3216266.

#### Emended description of *Mycobacterium austroafricanum* Tsukamura et al. 1983

The description is as before (Tsukamura et al., 1983) with the following addition. The G+C content of the type-strain genome is 67.7%, its approximate size 6.68 Mbp, its GenBank deposit SAMEA3138973.

#### Emended description of *Mycobacterium boenickei* Schinsky et al. 2004

The description is as before (Schinsky et al., 2004) with the following modification. The G+C content of the type-strain genome is 66.8%, its approximate size 6.51 Mbp, its GenBank deposit SAMEA92028418.

#### Emended description of *Mycobacterium bohemicum* Reischl et al. 1998

The description is as before (Reischl et al., 1998) with the following modification. The G+C content of the type-strain genome is 68.8%, its approximate size 5.08 Mbp, its GenBank deposit SAMEA3305052.

#### Emended description of *Mycobacterium bouchedurhonense* Ben Salah et al. 2009

The description is as before (Ben Salah et al., 2009) with the following addition. The G+C content of the type-strain genome is 68.6%, its approximate size 5.90 Mbp, its GenBank deposit SAMN06064234.

#### Emended description of *Mycobacterium branderi* Koukila-Kähkölä et al. 1995

The description is as before (Koukila-Kähkölä et al., 1995) with the following addition. The G+C content of the type-strain genome is 66.5%, its approximate size 5.90 Mbp, its GenBank deposit SAMN06064235.

#### Emended description of *Mycobacterium celatum* Butler et al. 1993

The description is as before (Butler et al., 1993) with the following addition. The G+C content of the type-strain genome is 67.0%, its approximate size 4.72 Mbp, its GenBank deposit SAMN04216916.

#### Emended description of *Mycobacterium celeriflavum* Shahraki et al. 2015

The description is as before (Shahraki et al., 2015) with the following addition. The G+C content of the type-strain genome is 66.9%, its approximate size 4.95 Mbp, its GenBank deposit SAMN06064236.

#### Emended description of *Mycobacterium chelonae* Bergey et al. 1923 emend. Kim et al. 2017

The description is as before (Kim et al., 2017) with the following addition. The G+C content of the type-strain genome is 63.9%, its approximate size 5.03 Mbp, its GenBank deposit SAMN02630629.

#### Emended description of *Mycobacterium chlorophenolicum* (Apajalahti et al. 1986) Häggblom et al. 1994

The description is as before (Hägglblom et al., 1994) with the following restriction. The G+C content of the type-strain genome is 68.4%, its approximate size 7.33 Mbp, its GenBank deposit SAMD00046518.

#### Emended description of *Mycobacterium chubuense* (ex Tsukamura 1973) Tsukamura 1981

The description is as before (Tsukamura, 1981) with the following addition. The G+C content of the type-strain genome is 69.2%, its approximate size 5.94 Mbp, its GenBank deposit SAMN03375743.

#### Emended description of *Mycobacterium colombiense* Murcia et al. 2006

The description is as before (Murcia et al., 2006) with the following addition. The G+C content of the type-strain genome is 68.1%, its approximate size 5.58 Mbp, its GenBank deposit SAMN00622208.

#### Emended description of *Mycobacterium conceptionense* Adékambi et al. 2006

The description is as before (Adékambi et al., 2006) with the following restriction. The G+C content of the type-strain genome is 66.3%, its approximate size 6.38 Mbp, its GenBank deposit SAMEA3305051.

#### Emended description of *Mycobacterium confluentis* Kirschner et al. 1992

The description is as before (Kirschner et al., 1992) with the following addition. The G+C content of the type-strain genome is 67.5%, its approximate size 5.84 Mbp, its GenBank deposit SAMN04216920.

#### Emended description of *Mycobacterium conspicuum* Springer et al. 1996

The description is as before (Springer et al., 1996) with the following addition. The G+C content of the type-strain genome is 67.4%, its approximate size 6.20 Mbp, its GenBank deposit SAMN04216921.

#### Emended description of *Mycobacterium cosmeticum* Cooksey et al. 2004

The description is as before (Cooksey et al., 2004) with the following addition. The G+C content of the type-strain genome is 68.2%, its approximate size 6.46 Mbp, its GenBank deposit SAMEA3138996.

#### Emended description of *Mycobacterium doricum* Tortoli et al. 2001

The description is as before (Tortoli et al., 2001) with the following modification. The G+C content of the type-strain genome is 67.6%, its approximate size 3.95 Mbp, its GenBank deposit SAMN04216922.

#### Emended description of *Mycobacterium engbaekii* Tortoli et al. 2013

The description is as before (Tortoli et al., 2013) with the following addition. The G+C content of the type-strain genome is 68.5%, its approximate size 4.52 Mbp, its GenBank deposit SAMN04216923.

#### Emended description of *Mycobacterium europaeum* Tortoli et al. 2011

The description is as before (Tortoli et al., 2011) with the following addition. The G+C content of the type-strain genome is 68.5%, its approximate size 5.63 Mbp, its GenBank deposit SAMN04216924.

#### Emended description of *Mycobacterium fallax* Lévy-Frébault et al. 1983

The description is as before (Lévy-Frébault et al., 1983) with the following addition. The G+C content of the type-strain genome is 70.3%, its approximate size 4.23 Mbp, its GenBank deposit SAMN04216925.

#### Emended description of *Mycobacterium florentinum* Tortoli et al. 2005

The description is as before (Tortoli et al., 2005) with the following addition. The G+C content of the type-strain genome is 66.4%, its approximate size 6.18 Mbp, its GenBank deposit SAMN04216926.

#### Emended description of *Mycobacterium fortuitum* da Costa Cruz 1938

The description is as before (da Costa Cruz, 1938) with the following addition. The G+C content of the type-strain genome is 66.4%, its approximate size 5.90 Mbp, its GenBank deposit SAMN04295031.

#### Emended description of *Mycobacterium fragae* Ramos et al. 2013

The description is as before (Ramos et al., 2013) with the following addition. The G+C content of the type-strain genome is 66.0%, its approximate size 4.73 Mbp, its GenBank deposit SAMN04216927.

#### Emended description of *Mycobacterium franklinii* Nogueira et al. 2015

The description is as before (Nogueira et al., 2015) with the following addition. The G+C content of the type-strain genome is 64.1%, its approximate size 5.43 Mbp, its GenBank deposit SAMN05186797.

#### Emended description of *Mycobacterium genavense* Böttger et al. 1993

The description is as before (Böttger et al., 1993) with the following addition. The G+C content of the type-strain genome is 66.9%, its approximate size 4.94 Mbp, its GenBank deposit SAMN02585006.

#### Emended description of *Mycobacterium gordonae* Bojalil et al. 1962

The description is as before (Bojalil et al., 1962) with the following addition. The G+C content of the type-strain genome is 66.6%, its approximate size 7.60 Mbp, its GenBank deposit SAMN04216929.

#### Emended description of *Mycobacterium haemophilum* Sompolinsky et al. 1978

The description is as before (Sompolinsky et al., 1978) with the following modification. The G+C content of the type-strain genome is 63.9%, its approximate size 4.24 Mbp, its GenBank deposit SAMN01918491.

#### Emended description of *Mycobacterium hassiacum* Schröder et al. 1997

The description is as before (Schröder et al., 1997) with the following modification. The G+C content of the type-strain genome is 69.4%, its approximate size 5.08 Mbp, its GenBank deposit SAMN02256416.

#### Emended description of *Mycobacterium heidelbergense* Haas et al. 1998

The description is as before (Haas et al., 1997) with the following addition. The G+C content of the type-strain genome is 68.0%, its approximate size 5.00 Mbp, its GenBank deposit SAMN06064240.

#### Emended description of *Mycobacterium hiberniae* Kazda et al. 1993

The description is as before (Kazda et al., 1993) with the following addition. The G+C content of the type-strain genome is 68.5%, its approximate size 4.34 Mbp, its GenBank deposit SAMN04216930.

#### Emended description of *Mycobacterium houstonense* Schinsky et al. 2004

The description is as before (Schinsky et al., 2004) with the following modification. The G+C content of the type-strain genome is 67.0%, its approximate size 6.38 Mbp, its GenBank deposit SAMEA3913388.

#### Emended description of *Mycobacterium immunogenum* Wilson et al. 2001

The description is as before (Wilson et al., 2001) with the following addition. The G+C content of the type-strain genome is 64.3%, its approximate size 5.57 Mbp, its GenBank deposit SAMN03733495.

#### Emended description of *Mycobacterium interjectum* Springer et al. 1995

The description is as before (Springer et al., 1993) with the following addition. The G+C content of the type-strain genome is 67.9%, its approximate size 5.85 Mbp, its GenBank deposit SAMEA3913390.

#### Emended description of *Mycobacterium intermedium* Meier et al. 1993

The description is as before (Meier et al., 1993) with the following addition. The G+C content of the type-strain genome is 65.8%, its approximate size 6.82 Mbp, its GenBank deposit SAMN06064242.

#### Emended description of *Mycobacterium iranicum* Shojaei et al. 2013

The description is as before (Shojaei et al., 2013) with the following addition. The G+C content of the type-strain genome is 66.1%, its approximate size 6.34 Mbp, its GenBank deposit SAMN04216933.

#### Emended description of *Mycobacterium kansasii* Hauduroy 1955

The description is as before (Hauduroy, 1955) with the following addition. The G+C content of the type-strain genome is 66.2%, its approximate size 6.58 Mbp, its GenBank deposit SAMN02603596.

#### Emended description of *Mycobacterium koreense* Kim et al. 2012

The description is as before (Kim et al., 2012a) with the following addition. The G+C content of the type-strain genome is 69.4%, its approximate size 4.08 Mbp, its GenBank deposit SAMN06651658.

#### Emended description of *Mycobacterium kubicae* Floyd et al. 2000

The description is as before (Floyd et al., 2000) with the following addition. The G+C content of the type-strain genome is 66.0%, its approximate size 5.83 Mbp, its GenBank deposit SAMN04216934.

#### Emended description of *Mycobacterium kumamotonense* Masaki et al. 2007

The description is as before (Masaki et al., 2006) with the following addition. The G+C content of the type-strain genome is 68.1%, its approximate size 4.82 Mbp, its GenBank deposit SAMN06064243.

#### Emended description of *Mycobacterium kyorinense* Okazaki et al. 2009

The description is as before (Okazaki et al., 2009) with the following addition. The G+C content of the type-strain genome is 66.9%, its approximate size 5.30 Mbp, its GenBank deposit SAMD00019087.

#### Emended description of *Mycobacterium lacus* Turenne et al. 2002

The description is as before (Turenne et al., 2002) with the following addition. The G+C content of the type-strain genome is 66.9%, its approximate size 4.91 Mbp, its GenBank deposit SAMN04216936.

#### Emended description of *Mycobacterium longobardum* Tortoli et al. 2013

The description is as before (Tortoli et al., 2013) with the following addition. The G+C content of the type-strain genome is 67.9%, its approximate size 4.81 Mbp, its GenBank deposit SAMN04216937.

#### Emended description of *Mycobacterium mageritense* Domenech et al. 1997

The description is as before (Domenech et al., 1997) with the following addition. The G+C content of the type-strain genome is 66.9%, its approximate size 7.97 Mbp, its GenBank deposit SAMEA3138978.

#### Emended description of *Mycobacterium mantenii* van Ingen et al. 2009

The description is as before (van Ingen et al., 2009d) with the following addition. The G+C content of the type-strain genome is 66.9%, its approximate size 6.12 Mbp, its GenBank deposit SAMN06064245.

#### Emended description of *Mycobacterium marseillense* Ben Salah et al. 2009

The description is as before (Ben Salah et al., 2009) with the following addition. The G+C content of the type-strain genome is 67.7%, its approximate size 5.46 Mbp, its GenBank deposit SAMN06064246.

#### Emended description of *Mycobacterium minnesotense* Hannigan et al. 2013

The description is as before (Hannigan et al., 2013) with the following addition. The G+C content of the type-strain genome is 67.1%, its approximate size 4.19 Mbp, its GenBank deposit SAMN06064248.

#### Emended description of *Mycobacterium monacense* Reischl et al. 2006

The description is as before (Reischl et al., 2006) with the following addition. The G+C content of the type-strain genome is 68.4%, its approximate size 6.00 Mbp, its GenBank deposit SAMN06064249.

#### Emended description of *Mycobacterium moriokaense* Tsukamura et al. 1986

The description is as before (Tsukamura et al., 1986) with the following modification. The G+C content of the type-strain genome is 66.0%, its approximate size 6.22 Mbp, its GenBank deposit SAMN06064251.

#### Emended description of *Mycobacterium nebraskense* Mohamed et al. 2004

The description is as before (Mohamed et al., 2004) with the following addition. The G+C content of the type-strain genome is 66.6%, its approximate size 6.73 Mbp, its GenBank deposit SAMN04216938.

#### Emended description of *Mycobacterium neworleansense* Schinsky et al. 2004

The description is as before (Schinsky et al., 2004) with the following modification. The G+C content of the type-strain genome is 66.8%, its approximate size 6.29 Mbp, its GenBank deposit SAMEA3481574.

#### Emended description of *Mycobacterium noviomagense* van Ingen et al. 2009

The description is as before (van Ingen et al., 2009b) with the following addition. The G+C content of the type-strain genome is 65.8%, its approximate size 4.74 Mbp, its GenBank deposit SAMN06064252.

#### Emended description of *Mycobacterium obuense* (ex Tsukamura and Mizuno 1971) Tsukamura and Mizuno 1981

The description is as before (Tsukamura et al., 1981) with the following addition. The G+C content of the type-strain genome is 68.0%, its approximate size 5.71 Mbp, its GenBank deposit SAMN02952053.

#### Emended description of *Mycobacterium palustre* Torkko et al. 2002

The description is as before (Torkko et al., 2002) with the following addition. The G+C content of the type-strain genome is 68.5%, its approximate size 6.04 Mbp, its GenBank deposit SAMN04216940.

#### Emended description of *Mycobacterium paraense* da Costa et al. 2015

The description is as before (Fusco da Costa et al., 2015) with the following addition. The G+C content of the type-strain genome is 69.3%, its approximate size 5.62 Mbp, its GenBank deposit SAMN04216942.

#### Emended description of *Mycobacterium paraintracellulare* Lee et al. 2016

The description is as before (Lee et al., 2016) with the following addition. The G+C content of the type-strain genome is 68.1%, its approximate size 5.50 Mbp, its GenBank deposit SAMN02603185.

#### Emended description of *Mycobacterium parascrofulaceum* Turenne et al. 2004

The description is as before (Turenne et al., 2004a) with the following addition. The G+C content of the type-strain genome is 68.4%, its approximate size 6.30 Mbp, its GenBank deposit SAMN00189881.

#### Emended description of *Mycobacterium paraseoulense* Lee et al. 2010

The description is as before (Lee et al., 2010) with the following addition. The G+C content of the type-strain genome is 67.9%, its approximate size 6.08 Mbp, its GenBank deposit SAMN06064254.

#### Emended description of *Mycobacterium parmense* Fanti et al. 2004

The description is as before (Fanti et al., 2004) with the following addition. The G+C content of the type-strain genome is 68.4%, its approximate size 5.89 Mbp, its GenBank deposit SAMN04216945.

#### Emended description of *Mycobacterium peregrinum* (ex Bojalil et al., 1962) Kusunoki and Ezaki 1992

The description is as before (Kusunoki and Ezaki, 1992) with the following addition. The G+C content of the type-strain genome is 66.3%, its approximate size 7.05 Mbp, its GenBank deposit SAMN04216946.

#### Emended description of *Mycobacterium phlei* Lehmann and Neumann 1899

The description is as before (Lehmann and Neumann, 1899) with the following addition. The G+C content of the type-strain genome is 69.4%, its approximate size 5.32 Mbp, its GenBank deposit SAMN04537322.

#### Emended description of *Mycobacterium pseudoshottsii* Rhodes et al. 2005

The description is as before (Rhodes et al., 2005) with the following addition. The G+C content of the type-strain genome is 65.7%, its approximate size 5.94 Mbp, its GenBank deposit SAMD00042842.

#### Emended description of *Mycobacterium rhodesiae* (ex Tsukamura et al. 1971) Tsukamura 1981

The description is as before (Tsukamura, 1981) with the following addition. The G+C content of the type-strain genome is 66.6%, its approximate size 6.01 Mbp, its GenBank deposit SAMN06064257.

#### Emended description of *Mycobacterium riyadhense* van Ingen et al. 2009

The description is as before (van Ingen et al., 2009a) with the following addition. The G+C content of the type-strain genome is 65.3%, its approximate size 6.27 Mbp, its GenBank deposit SAMN04216947.

#### Emended description of *Mycobacterium rufum* Hennessee et al. 2009

The description is as before (Hennessee et al., 2009) with the following addition. The G+C content of the type-strain genome is 69.2%, its approximate size 6.18 Mbp, its GenBank deposit SAMN02777056.

#### Emended description of *Mycobacterium rutilum* Hennessee et al. 2009

The description is as before (Hennessee et al., 2009) with the following addition. The G+C content of the type-strain genome is 68.4%, its approximate size 5.99 Mbp, its GenBank deposit SAMN04489835.

#### Emended description of *Mycobacterium salmoniphilum* (ex Ross 1960) Whipps et al. 2007

The description is as before (Whipps et al., 2007) with the following addition. The G+C content of the type-strain genome is 64.3%, its approximate size 4.77 Mbp, its GenBank deposit SAMN05186798.

#### Emended description of *Mycobacterium saskatchewanense* Turenne et al. 2004

The description is as before (Turenne et al., 2004b) with the following addition. The G+C content of the type-strain genome is 68.3%, its approximate size 5.93 Mbp, its GenBank deposit SAMN04216948.

#### Emended description of *Mycobacterium scrofulaceum* Prissick and Masson 1956

The description is as before (Prissick and Masson, 1956) with the following addition. The G+C content of the type-strain genome is 68.4%, its approximate size 6.17 Mbp, its GenBank deposit SAMN06064259.

#### Emended description of *Mycobacterium senuense* Mun et al. 2008

The description is as before (Mun et al., 2008) with the following addition. The G+C content of the type-strain genome is 68.7%, its approximate size 4.53 Mbp, its GenBank deposit SAMN04216949.

#### Emended description of *Mycobacterium septicum* Schinsky et al. 2000

The description is as before (Schinsky et al., 2000) with the following modification. The G+C content of the type-strain genome is 66.7%, its approximate size 6.87 Mbp, its GenBank deposit SAMEA2272699.

#### Emended description of *Mycobacterium setense* Lamy et al. 2008

The description is as before (Lamy et al., 2008) with the following addition. The G+C content of the type-strain genome is 66.4%, its approximate size 6.27 Mbp, its GenBank deposit SAMN01174788.

#### Emended description of *Mycobacterium sherrisii* van Ingen et al. 2011

The description is as before (van Ingen et al., 2011) with the following addition. The G+C content of the type-strain genome is 67.0%, its approximate size 5.56 Mbp, its GenBank deposit SAMN04216950.

#### Emended description of *Mycobacterium shimoidei* (ex Tsukamura et al. 1975) Tsukamura 1982

The description is as before (Tsukamura, 1982a) with the following addition. The G+C content of the type-strain genome is 65.8%, its approximate size 4.71 Mbp, its GenBank deposit SAMN04216951.

#### Emended description of *Mycobacterium shinjukuense* Saito et al. 2011

The description is as before (Saito et al., 2011) with the following addition. The G+C content of the type-strain genome is 67.8%, its approximate size 4.41 Mbp, its GenBank deposit SAMN06064260.

#### Emended description of *Mycobacterium simiae* Karassova et al. 1965

The description is as before (Karassova et al., 1965) with the following addition. The G+C content of the type-strain genome is 66.2%, its approximate size 5.69 Mbp, its GenBank deposit SAMEA2272207.

#### Emended description of *Mycobacterium smegmatis* (Trevisan, 1889) Lehmann and Neumann 1899

The description is as before (Lehmann and Neumann, 1899) with the following addition. The G+C content of the type-strain genome is 67.3%, its approximate size 6.98 Mbp, its GenBank deposit SAMEA2517361.

#### Emended description of *Mycobacterium szulgai* Marks et al. 1972

The description is as before (Marks and Jenkins, 1972) with the following addition. The G+C content of the type-strain genome is 65.8%, its approximate size 6.67 Mbp, its GenBank deposit SAMN04216953.

#### Emended description of *Mycobacterium thermoresistibile* Tsukamura 1966

The description is as before (Tsukamura, 1966) with the following addition. The G+C content of the type-strain genome is 69.0%, its approximate size 4.87 Mbp, its GenBank deposit SAMN02471087.

#### Emended description of *Mycobacterium triplex* Floyd et al. 1997

The description is as before (Floyd et al., 1996) with the following addition. The G+C content of the type-strain genome is 66.6%, its approximate size 6.38 Mbp, its GenBank deposit SAMEA3139001.

#### Emended description of *Mycobacterium vaccae* Bönicke and Juhasz 1964

The description is as before (Bönicke and Juhasz, 1964) with the following addition. The G+C content of the type-strain genome is 68.6%, its approximate size 6.24 Mbp, its GenBank deposit SAMN03702434.

#### Emended description of *Mycobacterium vanbaalenii* Khan et al. 2002

The description is as before (Khan et al., 2002) with the following modification. The G+C content of the type-strain genome is 67.8%, its approximate size 6.49 Mbp, its GenBank deposit SAMN02598347.

#### Emended description of *Mycobacterium vulneris* van Ingen et al. 2009

The description is as before (van Ingen et al., 2009c) with the following addition. The G+C content of the type-strain genome is 66.8%, its approximate size 6.27 Mbp, its GenBank deposit SAMN06651660.

#### Emended description of *Mycobacterium wolinskyi* Brown et al. 1999

The description is as before (Brown et al., 1999) with the following addition. The G+C content of the type-strain genome is 66.4%, its approximate size 7.52 Mbp, its GenBank deposit SAMN04216957.

#### Emended description of *Mycobacterium xenopi* Schwabacher 1959

The description is as before (Schwabacher, 1959) with the following addition. The G+C content of the type-strain genome is 65.9%, its approximate size 4.93 Mbp, its GenBank deposit SAMN04216958.

#### Emended description of *Nakamurella multipartita* (Yoshimi et al. 1996) Tao et al. 2004

The description is as before (Tao et al., 2004) with the following modification. The G+C content of the type-strain genome is 70.9%, its approximate size 6.06 Mbp, its GenBank deposit SAMN02598467.

#### Emended description of *Nesterenkonia alba* Luo et al. 2009

The description is as before (Luo et al., 2009) with the following modification. The G+C content of the type-strain genome is 63.7%, its approximate size 2.59 Mbp, its GenBank deposit SAMN02440588.

#### Emended description of *Nitriliruptor alkaliphilus* Sorokin et al. 2009

The description is as before (Sorokin et al., 2009) with the following modification. The G+C content of the type-strain genome is 72.1%, its approximate size 5.56 Mbp, its GenBank deposit SAMN02745916.

#### Emended description of *Nocardia africana* Hamid et al. 2001

The description is as before (Hamid et al., 2001) with the following addition. The G+C content of the type-strain genome is 67.9%, its approximate size 7.81 Mbp, its GenBank deposit SAMD00018765.

#### Emended description of *Nocardia amikacinitolerans* Ezeoke et al. 2013

The description is as before (Ezeoke et al., 2013) with the following restriction. The G+C content of the type-strain genome is 68.5%, its approximate size 7.66 Mbp, its GenBank deposit SAMN04244555.

#### Emended description of *Nocardia asiatica* Kageyama et al. 2004

The description is as before (Kageyama et al., 2004a) with the following restriction. The G+C content of the type-strain genome is 68.4%, its approximate size 8.46 Mbp, its GenBank deposit SAMD00041765.

#### Emended description of *Nocardia asteroides* (Eppinger 1891) Blanchard 1896

The description is as before (Blanchard, 1896) with the following addition. The G+C content of the type-strain genome is 69.9%, its approximate size 6.95 Mbp, its GenBank deposit SAMN05444423.

#### Emended description of *Nocardia brasiliensis* (Lindenberg 1909) Pinoy 1913

The description is as before (Pinoy, 1913) with the following addition. The G+C content of the type-strain genome is 68.2%, its approximate size 8.90 Mbp, its GenBank deposit SAMD00016800.

#### Emended description of *Nocardia brevicatena* (Lechevalier et al. 1961) Goodfellow and Pirouz 1982

The description is as before (Goodfellow and Pirouz, 1982) with the following addition. The G+C content of the type-strain genome is 67.0%, its approximate size 7.01 Mbp, its GenBank deposit SAMD00041812.

#### Emended description of *Nocardia carnea* (Rossi Doria 1891) Castellani and Chalmers 1913

The description is as before (Castellani and Chalmers, 1913) with the following addition. The G+C content of the type-strain genome is 67.1%, its approximate size 7.49 Mbp, its GenBank deposit SAMD00041818.

#### Emended description of *Nocardia crassostreae* Friedman et al. 1998

The description is as before (Friedman et al., 1998) with the following modification. The G+C content of the type-strain genome is 67.7%, its approximate size 8.29 Mbp, its GenBank deposit SAMD00040648.

#### Emended description of *Nocardia farcinica* Trevisan 1889

The description is as before (Trevisan, 1889) with the following addition. The G+C content of the type-strain genome is 70.7%, its approximate size 6.41 Mbp, its GenBank deposit SAMN05421776.

#### Emended description of *Nocardia fluminea* Maldonado et al. 2001

The description is as before (Maldonado et al., 2000) with the following addition. The G+C content of the type-strain genome is 67.5%, its approximate size 8.06 Mbp, its GenBank deposit SAMN04489843.

#### Emended description of *Nocardia inohanensis* Kageyama et al. 2004

The description is as before (Kageyama et al., 2004c) with the following modification. The G+C content of the type-strain genome is 67.8%, its approximate size 8.12 Mbp, its GenBank deposit SAMD00040655.

#### Emended description of *Nocardia jejuensis* Lee 2006

The description is as before (Lee, 2006b) with the following modification. The G+C content of the type-strain genome is 67.6%, its approximate size 8.65 Mbp, its GenBank deposit SAMD00018769.

#### Emended description of *Nocardia jiangxiensis* Cui et al. 2005

The description is as before (Cui et al., 2005) with the following addition. The G+C content of the type-strain genome is 66.8%, its approximate size 10.45 Mbp, its GenBank deposit SAMD00041792.

#### Emended description of *Nocardia kruczakiae* Conville et al. 2005

The description is as before (Conville et al., 2004) with the following addition. The G+C content of the type-strain genome is 68.0%, its approximate size 7.32 Mbp, its GenBank deposit SAMD00040657.

#### Emended description of *Nocardia niigatensis* Kageyama et al. 2004

The description is as before (Kageyama et al., 2004c) with the following modification. The G+C content of the type-strain genome is 68.2%, its approximate size 8.22 Mbp, its GenBank deposit SAMD00041766.

#### Emended description of *Nocardia nova* Tsukamura 1983

The description is as before (Tsukamura, 1982b) with the following addition. The G+C content of the type-strain genome is 67.9%, its approximate size 7.85 Mbp, its GenBank deposit SAMD00040661.

#### Emended description of *Nocardia otitidiscaviarum* Snijders 1924

The description is as before (Snijders, 1924) with the following addition. The G+C content of the type-strain genome is 69.0%, its approximate size 7.48 Mbp, its GenBank deposit SAMD00041819.

#### Emended description of *Nocardia pseudovaccinii* Kim et al. 2002

The description is as before (Kim et al., 2002) with the following addition. The G+C content of the type-strain genome is 65.7%, its approximate size 9.94 Mbp, its GenBank deposit SAMD00018770.

#### Emended description of *Nocardia rhamnosiphila* Everest et al. 2012

The description is as before (Everest et al., 2011) with the following addition. The G+C content of the type-strain genome is 68.5%, its approximate size 7.67 Mbp, its GenBank deposit SAMN02645234.

#### Emended description of *Nocardia shimofusensis* Kageyama et al. 2004

The description is as before (Kageyama et al., 2004b) with the following modification. The G+C content of the type-strain genome is 69.1%, its approximate size 6.33 Mbp, its GenBank deposit SAMD00040665.

#### Emended description of *Nocardia soli* Maldonado et al. 2001

The description is as before (Maldonado et al., 2000) with the following addition. The G+C content of the type-strain genome is 67.0%, its approximate size 7.55 Mbp, its GenBank deposit SAMD00018782.

#### Emended description of *Nocardia transvalensis* Pijper and Pullinger 1927

The description is as before (Pijper and Pullinger, 1927) with the following addition. The G+C content of the type-strain genome is 69.2%, its approximate size 8.38 Mbp, its GenBank deposit SAMD00041825.

#### Emended description of *Nocardia uniformis* (ex Marton and Szabó 1959) Isik et al. 1999

The description is as before (Isik et al., 1999) with the following addition. The G+C content of the type-strain genome is 66.0%, its approximate size 8.77 Mbp, its GenBank deposit SAMD00040667.

#### Emended description of *Nocardia vaccinii* Demaree and Smith 1952

The description is as before (Demaree and Smith, 1952) with the following addition. The G+C content of the type-strain genome is 66.7%, its approximate size 9.22 Mbp, its GenBank deposit SAMD00040668.

#### Emended description of *Nocardia veterana* Gürtler et al. 2001

The description is as before (Gürtler et al., 2001) with the following addition. The G+C content of the type-strain genome is 68.2%, its approximate size 6.79 Mbp, its GenBank deposit SAMD00041771.

#### Emended description of *Nocardia yamanashiensis* Kageyama et al. 2004

The description is as before (Kageyama et al., 2004c) with the following modification. The G+C content of the type-strain genome is 68.1%, its approximate size 9.10 Mbp, its GenBank deposit SAMD00018773.

#### Emended description of *Nocardioides dokdonensis* Park et al. 2008

The description is as before (Park et al., 2008) with the following modification. The G+C content of the type-strain genome is 72.3%, its approximate size 4.38 Mbp, its GenBank deposit SAMN04606834.

#### Emended description of *Nocardioides exalbidus* Li et al. 2007

The description is as before (Li et al., 2007) with the following modification. The G+C content of the type-strain genome is 71.8%, its approximate size 4.60 Mbp, its GenBank deposit SAMN04489844.

#### Emended description of *Nocardioides halotolerans* Dastager et al. 2009

The description is as before (Dastager et al., 2009) with the following modification. The G+C content of the type-strain genome is 72.0%, its approximate size 4.85 Mbp, its GenBank deposit SAMN02440651.

#### Emended description of *Nocardioides psychrotolerans* Liu et al. 2013

The description is as before (Liu et al., 2013) with the following modification. The G+C content of the type-strain genome is 70.6%, its approximate size 4.52 Mbp, its GenBank deposit SAMN05216561.

#### Emended description of *Nocardioides simplex* (Jensen 1934) O'Donnell et al. 1983

The description is as before (O'Donnell et al., 1982) with the following modification. The G+C content of the type-strain genome is 72.9%, its approximate size 5.63 Mbp, its GenBank deposit SAMN05421671.

#### Emended description of *Nocardioides szechwanensis* Liu et al. 2013

The description is as before (Liu et al., 2013) with the following modification. The G+C content of the type-strain genome is 71.0%, its approximate size 4.22 Mbp, its GenBank deposit SAMN05192576.

#### Emended description of *Nocardiopsis alba* Grund and Kroppenstedt 1990

The description is as before (Grund and Kroppenstedt, 1990) with the following addition. The G+C content of the type-strain genome is 69.8%, its approximate size 5.82 Mbp, its GenBank deposit SAMN02472146.

#### Emended description of *Nocardiopsis alkaliphila* Hozzein et al. 2004

The description is as before (Hozzein et al., 2004) with the following modification. The G+C content of the type-strain genome is 67.5%, its approximate size 5.21 Mbp, its GenBank deposit SAMN02472156.

#### Emended description of *Nocardiopsis alborubida* Grund and Kroppenstedt 1990

The description is as before (Grund and Kroppenstedt, 1990) with the following addition. The G+C content of the type-strain genome is 72.0%, its approximate size 7.18 Mbp, its GenBank deposit SAMD00046506.

#### Emended description of *Nocardiopsis halophila* Al-Tai and Ruan 1994

The description is as before (Al-Tai and Ruan, 1994) with the following additions. Urea hydrolysis is variable, The predominant menaquinone is MK-10; the major phospholipids are PC and DPG, although PME, PI and PG may occur. The G+C content of the type-strain genome is 73.7%, its approximate size 6.32 Mbp, its GenBank deposit SAMN02472157.

#### Emended description of *Nocardiopsis chromatogenes* Li et al. 2006

The description is as before (Li et al., 2006) with the following modification. The G+C content of the type-strain genome is 73.8%, its approximate size 6.91 Mbp, its GenBank deposit SAMN02472161.

#### Emended description of *Nocardiopsis dassonvillei* (Brocq-Rousseau, 1904) Meyer 1976

The description is as before (Meyer, 1976) with the following addition. The G+C content of the type-strain genome is 72.7%, its approximate size 6.54 Mbp, its GenBank deposit SAMN02598425.

#### Emended description of *Nocardiopsis flavescens* Fang et al. 2011

The description is as before (Fang et al., 2011) with the following modification. The G+C content of the type-strain genome is 74.1%, its approximate size 7.17 Mbp, its GenBank deposit SAMN05421803.

#### Emended description of *Nocardiopsis gilva* Li et al. 2006

The description is as before (Li et al., 2006) with the following modification. The G+C content of the type-strain genome is 69.7%, its approximate size 6.04 Mbp, its GenBank deposit SAMN02472152.

#### Emended description of *Nocardiopsis halotolerans* Al-Zarban et al. 2002

The description is as before (Al-Zarban et al., 2002a) with the following modification. The G+C content of the type-strain genome is 71.4%, its approximate size 6.26 Mbp, its GenBank deposit SAMN02472154.

#### Emended description of *Nocardiopsis kunsanensis* Chun et al. 2000

The description is as before (Chun et al., 2000) with the following modification. The G+C content of the type-strain genome is 69.6%, its approximate size 5.31 Mbp, its GenBank deposit SAMN02472160.

#### Emended description of *Nocardiopsis listeri* Grund and Kroppenstedt 1990

The description is as before (Grund and Kroppenstedt, 1990) with the following addition. The G+C content of the type-strain genome is 68.9%, its approximate size 5.69 Mbp, its GenBank deposit SAMD00046482.

#### Emended description of *Nocardiopsis potens* Yassin et al. 2009

The description is as before (Yassin et al., 2009) with the following addition. The G+C content of the type-strain genome is 74.8%, its approximate size 6.91 Mbp, its GenBank deposit SAMN02472155.

#### Emended description of *Nocardiopsis prasina* (Miyashita et al. 1984) Yassin et al. 1997

The description is as before (Yassin et al., 1997) with the following addition. The G+C content of the type-strain genome is 70.8%, its approximate size 6.00 Mbp, its GenBank deposit SAMN02472147.

#### Emended description of *Nocardiopsis salina* Li et al. 2004 emend. Li et al. 2006

The description is as before (Li et al., 2006) with the following modification. The G+C content of the type-strain genome is 70.4%, its approximate size 5.81 Mbp, its GenBank deposit SAMN02472151.

#### Emended description of *Nocardiopsis synnemataformans* Yassin et al. 1997

The description is as before (Yassin et al., 1997) with the following modification. The G+C content of the type-strain genome is 72.3%, its approximate size 7.36 Mbp, its GenBank deposit SAMN02472153.

#### Emended description of *Nocardiopsis trehalosi* (ex Dolak et al. 1981) Evtushenko et al. 2000

The description is as before (Evtushenko et al., 2000b) with the following addition. The G+C content of the type-strain genome is 75.2%, its approximate size 6.38 Mbp, its GenBank deposit SAMD00046510.

#### Emended description of *Nocardiopsis xinjiangensis* Li et al. 2003

The description is as before (Li et al., 2003b) with the following modification. The G+C content of the type-strain genome is 69.7%, its approximate size 5.33 Mbp, its GenBank deposit SAMN02472150.

#### Emended description of *Nonomuraea candida* Le Roes and Meyers 2009

The description is as before (le Roes and Meyers, 2008) with the following addition. The G+C content of the type-strain genome is 72.1%, its approximate size 9.63 Mbp, its GenBank deposit SAMN02645361.

#### Emended description of *Nonomuraea jiangxiensis* Li et al. 2012

The description is as before (Li et al., 2012) with the following modification. The G+C content of the type-strain genome is 70.6%, its approximate size 11.81 Mbp, its GenBank deposit SAMN05421869.

#### Emended description of *Nonomuraea kuesteri* Kämpfer et al. 2005

The description is as before (Kämpfer et al., 2005) with the following addition. The G+C content of the type-strain genome is 70.4%, its approximate size 12.10 Mbp, its GenBank deposit SAMN02645367.

#### Emended description of *Nonomuraea pusilla* (Nonomura and Ohara 1971) Zhang et al. 1998

The description is as before (Zhang et al., 1998) with the following addition. The G+C content of the type-strain genome is 72.4%, its approximate size 9.20 Mbp, its GenBank deposit SAMN05660976.

#### Emended description of *Nonomuraea solani* Wang et al. 2013

The description is as before (Wang et al., 2013b) with the following modification. The G+C content of the type-strain genome is 70.4%, its approximate size 13.24 Mbp, its GenBank deposit SAMN05444920.

#### Emended description of *Oerskovia turbata* (Erikson 1954) Prauser et al. 1970 emend. Stackebrandt et al. 2002

The description is as before (Stackebrandt et al., 2002) with the following restriction. The G+C content of the type-strain genome is 72.2%, its approximate size 4.03 Mbp, its GenBank deposit SAMN02645273.

#### Emended description of *Oerskovia xanthineolytica* Lechevalier 1972

The description is as before (Lechevalier, 1972) with the following addition. The G+C content of the type-strain genome is 74.3%, its approximate size 4.28 Mbp, its GenBank deposit SAMEA2272518.

#### Emended description of *Olsenella umbonata* Kraatz et al. 2011

The description is as before (Kraatz et al., 2011) with the following addition. The G+C content of the type-strain genome is 64.9%, its approximate size 2.35 Mbp, its GenBank deposit SAMN04489857.

#### Emended description of *Ornithinimicrobium pekingense* Liu et al. 2008

The description is as before (Liu et al., 2008) with the following modification. The G+C content of the type-strain genome is 72.9%, its approximate size 3.85 Mbp, its GenBank deposit SAMN02441240.

#### Emended description of *Paeniglutamicibacter gangotriensis* (Gupta et al. 2004) Busse 2016

The description is as before (Busse, 2016) with the following modification. The G+C content of the type-strain genome is 63.0%, its approximate size 4.32 Mbp, its GenBank deposit SAMN02469439.

#### Emended description of *Parascardovia denticolens* (Crociani et al. 1996) Jian and Dong 2002

The description is as before (Jian and Dong, 2002) with the following restriction. The G+C content of the type-strain genome is 58.3%, its approximate size 1.89 Mbp, its GenBank deposit SAMN02299441.

#### Emended description of *Patulibacter americanus* Reddy and Garcia-Pichel 2009

The description is as before Pichel (Reddy and Garcia-Pichel, 2009) with the following modification. The G+C content of the type-strain genome is 74.4%, its approximate size 4.47 Mbp, its GenBank deposit SAMN02440564.

#### Emended description of *Patulibacter minatonensis* Takahashi et al. 2006

The description is as before (Takahashi et al., 2006) with the following modification. The G+C content of the type-strain genome is 74.1%, its approximate size 5.51 Mbp, its GenBank deposit SAMN02584907.

#### Emended description of *Planomonospora sphaerica* Mertz 1994

The description is as before (Mertz, 1994) with the following addition. The G+C content of the type-strain genome is 72.7%, its approximate size 8.13 Mbp, its GenBank deposit SAMD00048613.

#### Emended description of *Prauserella alba* Li et al. 2003

The description is as before (Li et al., 2003c) with the following modification. The G+C content of the type-strain genome is 70.1%, its approximate size 5.62 Mbp, its GenBank deposit SAMN05660435.

#### Emended description of *Prauserella halophila* Li et al. 2003

The description is as before (Li et al., 2003c) with the following modification. The G+C content of the type-strain genome is 69.8%, its approximate size 5.05 Mbp, its GenBank deposit SAMN05660332.

#### Emended description of *Prauserella rugosa* (Lechevalier et al., 1986) Kim and Goodfellow 1999

The description is as before (Kim and Goodfellow, 1999) with the following restriction. The G+C content of the type-strain genome is 70.0%, its approximate size 5.28 Mbp, its GenBank deposit SAMN02946344.

#### Emended description of *Promicromonospora thailandica* Thawai and Kudo 2012

The description is as before (Thawai and Kudo, 2012) with the following modification. The G+C content of the type-strain genome is 73.1%, its approximate size 5.38 Mbp, its GenBank deposit SAMN04244578.

#### Emended description of *Promicromonospora umidemergens* Martin et al. 2010

The description is as before (Martin et al., 2010) with the following addition. The G+C content of the type-strain genome is 70.8%, its approximate size 6.60 Mbp, its GenBank deposit SAMN04324259.

#### Emended description of *Propionibacterium freudenreichii* van Niel 1928

The description is as before (van Niel, 1928) with the following addition. The G+C content of the type-strain genome is 67.3%, its approximate size 2.65 Mbp, its GenBank deposit SAMN03263638.

#### Emended description of *Propionibacterium lymphophilum* (Torrey 1916) Johnson and Cummins 1972

The description is as before (Johnson and Cummins, 1972) with the following addition. The G+C content of the type-strain genome is 56.1%, its approximate size 2.04 Mbp, its GenBank deposit SAMN02441171.

#### Emended description of *Pseudonocardia alni* (Evtushenko et al. 1989) Warwick et al. 1994

The description is as before (Warwick et al., 1994) with the following restriction. The G+C content of the type-strain genome is 74.2%, its approximate size 5.99 Mbp, its GenBank deposit SAMN04490211.

#### Emended description of *Pseudonocardia ammonioxydans* Liu et al. 2006

The description is as before (Liu et al., 2006) with the following modification. The G+C content of the type-strain genome is 73.5%, its approximate size 7.36 Mbp, its GenBank deposit SAMN05216207.

#### Emended description of *Pseudonocardia asaccharolytica* Reichert et al. 1998

The description is as before (Reichert et al., 1998) with the following addition. The G+C content of the type-strain genome is 71.8%, its approximate size 5.05 Mbp, its GenBank deposit SAMN02441500.

#### Emended description of *Pseudonocardia autotrophica* (Takamiya and Tubaki 1956) Warwick et al. 1994

The description is as before (Warwick et al., 1994) with the following modification. The G+C content of the type-strain genome is 73.0%, its approximate size 7.35 Mbp, its GenBank deposit SAMN05722966.

#### Emended description of *Pseudonocardia oroxyli* Gu et al. 2006

The description is as before (Gu et al., 2006) with the following modification. The G+C content of the type-strain genome is 73.0%, its approximate size 6.11 Mbp, its GenBank deposit SAMN05216377.

#### Emended description of *Pseudonocardia thermophila* Henssen 1957

The description is as before (Henssen, 1957) with the following addition. The G+C content of the type-strain genome is 72.9%, its approximate size 6.10 Mbp, its GenBank deposit SAMN05443637.

#### Emended description of *Pseudoscardovia suis* Killer et al. 2014

The description is as before (Killer et al., 2014) with the following modification. The G+C content of the type-strain genome is 60.5%, its approximate size 2.25 Mbp, its IMG deposit 2731639185.

#### Emended description of *Quadrisphaera granulorum* Maszenan et al. 2005

The description is as before (Maszenan et al., 2005) with the following modification. The G+C content of the type-strain genome is 72.6%, its approximate size 4.88 Mbp, its IMG deposit 2724679791.

#### Emended description of *Rathayibacter toxicus* (Riley and Ophel 1992) Sasaki et al. 1998

The description is as before (Sasaki et al., 1998) with the following modification. The G+C content of the type-strain genome is 61.5%, its approximate size 2.29 Mbp, its GenBank deposit SAMN02440627.

#### Emended description of *Renibacterium salmoninarum* Sanders and Fryer 1980

The description is as before (Sanders and Fryer, 1980) with the following modification. The G+C content of the type-strain genome is 56.3%, its approximate size 3.16 Mbp, its GenBank deposit SAMN02603684.

#### Emended description of *Rhodococcus coprophilus* Rowbotham and Cross 1979

The description is as before (Rowbotham and Cross, 1977) with the following restriction. The G+C content of the type-strain genome is 66.9%, its approximate size 4.55 Mbp, its GenBank deposit SAMD00046763.

#### Emended description of *Rhodococcus corynebacterioides* (Serrano et al. 1972) Yassin and Schaal 2005

The description is as before (Yassin and Schaal, 2005) with the following addition. The G+C content of the type-strain genome is 70.2%, its approximate size 3.90 Mbp, its GenBank deposit SAMN04357305.

#### Emended description of *Rhodococcus defluvii* Kämpfer et al. 2014

The description is as before (Kämpfer et al., 2014a) with the following addition. The G+C content of the type-strain genome is 68.7%, its approximate size 5.13 Mbp, its GenBank deposit SAMN02910065.

#### Emended description of *Rhodococcus enclensis* Dastager et al. 2014

The description is as before (Dastager et al., 2014a) with the following modification. The G+C content of the type-strain genome is 62.3%, its approximate size 7.46 Mbp, its GenBank deposit SAMN04263893.

#### Emended description of *Rhodococcus equi* (Magnusson 1923) Goodfellow and Alderson 1977

The description is as before (Goodfellow and Alderson, 1977) with the following modification. The G+C content of the type-strain genome is 68.8%, its approximate size 5.20 Mbp, its GenBank deposit SAMN02471952.

#### Emended description of *Rhodococcus erythropolis* (Gray and Thornton 1928) Goodfellow and Alderson 1979

The description is as before (Goodfellow and Alderson, 1977) with the following modification. The G+C content of the type-strain genome is 62.4%, its approximate size 6.59 Mbp, its GenBank deposit SAMD00046519.

#### Emended description of *Rhodococcus fascians* (Tilford 1936) Goodfellow 1984

The description is as before (Goodfellow, 1984) with the following restriction. The G+C content of the type-strain genome is 64.4%, its approximate size 5.77 Mbp, its GenBank deposit SAMN02570003.

#### Emended description of *Rhodococcus globerulus* Goodfellow et al. 1985

The description is as before (Goodfellow et al., 1982) with the following restriction. The G+C content of the type-strain genome is 61.7%, its approximate size 6.74 Mbp, its GenBank deposit SAMD00047211.

#### Emended description of *Rhodococcus gordoniae* Jones et al. 2004

The description is as before (Jones et al., 2004) with the following addition. The G+C content of the type-strain genome is 67.9%, its approximate size 4.82 Mbp, its GenBank deposit SAMN04357310.

#### Emended description of *Rhodococcus hoagii* (Morse 1912) Kämpfer et al. 2014

The description is as before (Kämpfer et al., 2014a) with the following addition. The G+C content of the type-strain genome is 68.8%, its approximate size 4.97 Mbp, its GenBank deposit SAMN04357304.

#### Emended description of *Rhodococcus imtechensis* Ghosh et al. 2006

The description is as before (Ghosh et al., 2006) with the following modification. The G+C content of the type-strain genome is 67.2%, its approximate size 8.23 Mbp, its GenBank deposit SAMN02470011.

#### Emended description of *Rhodococcus koreensis* Yoon et al. 2000

The description is as before (Yoon et al., 2000a) with the following modification. The G+C content of the type-strain genome is 67.4%, its approximate size 10.31 Mbp, its GenBank deposit SAMN04490239.

#### Emended description of *Rhodococcus maanshanensis* Zhang et al. 2002

The description is as before (Zhang et al., 2002) with the following modification. The G+C content of the type-strain genome is 69.2%, its approximate size 5.67 Mbp, its GenBank deposit SAMN05444583.

#### Emended description of *Rhodococcus phenolicus* Rehfuss and Urban 2006

The description is as before (Rehfuss and Urban, 2005) with the following modification. The G+C content of the type-strain genome is 68.4%, its approximate size 6.28 Mbp, its GenBank deposit SAMN04357313.

#### Emended description of *Rhodococcus pyridinivorans* Yoon et al. 2000

The description is as before (Yoon et al., 2000b) with the following modification. The G+C content of the type-strain genome is 67.8%, its approximate size 5.26 Mbp, its GenBank deposit SAMN04490240.

#### Emended description of *Rhodococcus qingshengii* Xu et al. 2007

The description is as before (Xu et al., 2007) with the following modification. Vogues-Proskauer and reduction of nitrate tests are variable. The G+C content of the type-strain genome is 62.4%, its approximate size 7.26 Mbp, its GenBank deposit SAMN04357315.

#### Emended description of *Rhodococcus rhodnii* Goodfellow and Alderson 1979

The description is as before (Goodfellow and Alderson, 1977) with the following modification. The G+C content of the type-strain genome is 69.7%, its approximate size 4.46 Mbp, its GenBank deposit SAMD00046764.

#### Emended description of *Rhodococcus rhodochrous* (Zopf, 1891) Tsukamura 1974 emend. Rainey et al. 1995

The description is as before (Rainey et al., 1995a) with the following restriction. The G+C content of the type-strain genome is 68.2%, its approximate size 5.20 Mbp, its GenBank deposit SAMD00034188.

#### Emended description of *Rhodococcus ruber* (Kruse 1896) Goodfellow and Alderson 1977

The description is as before (Goodfellow and Alderson, 1977) with the following addition. The G+C content of the type-strain genome is 70.7%, its approximate size 5.30 Mbp, its GenBank deposit SAMN04357318.

#### Emended description of *Rhodococcus triatomae* Yassin 2005

The description is as before (Yassin, 2005) with the following addition. The G+C content of the type-strain genome is 68.7%, its approximate size 4.73 Mbp, its GenBank deposit SAMN05444695.

#### Emended description of *Rhodococcus tukisamuensis* Matsuyama et al. 2003

The description is as before (Matsuyama et al., 2003) with the following modification. The G+C content of the type-strain genome is 69.8%, its approximate size 5.49 Mbp, its GenBank deposit SAMN05444580.

#### Emended description of *Rhodococcus wratislaviensis* (Goodfellow et al. 1995) Goodfellow et al. 2002

The description is as before (Goodfellow et al., 2002) with the following restriction. The G+C content of the type-strain genome is 66.8%, its approximate size 10.40 Mbp, its GenBank deposit SAMD00041784.

#### Emended description of *Rhodococcus zopfii* Stoecker et al. 1994

The description is as before (Stoecker et al., 1994) with the following modification. The G+C content of the type-strain genome is 68.2%, its approximate size 6.30 Mbp, its GenBank deposit SAMD00046765.

#### Emended description of *Roseiflexus castenholzii* Hanada et al. 2002

The description is as before (Hanada et al., 2002b) with the following modification. The G+C content of the type-strain genome is 60.7%, its approximate size 5.72 Mbp, its GenBank deposit SAMN02598306.

#### Emended description of *Rothia dentocariosa* (Onishi 1949) Georg and Brown 1967 emend. Daneshvar et al. 2004

The description is as before (Daneshvar et al., 2004) with the following addition. The G+C content of the type-strain genome is 53.7%, its approximate size 2.51 Mbp, its GenBank deposit SAMN00120582.

#### Emended description of *Rothia mucilaginosa* (Bergan and Kocur 1982) Collins et al. 2000

The description is as before (Collins et al., 2000b) with the following restriction. The G+C content of the type-strain genome is 59.5%, its approximate size 2.26 Mbp, its GenBank deposit SAMN00001919.

#### Emended description of *Rubrobacter aplysinae* Kämpfer et al. 2014

The description is as before (Kämpfer et al., 2014b) with the following addition. The G+C content of the type-strain genome is 65.1%, its approximate size 3.19 Mbp, its GenBank deposit SAMN03729479.

#### Emended description of *Rubrobacter radiotolerans* (Yoshinaka et al. 1973) Suzuki et al. 1989

The description is as before (Suzuki et al., 1988) with the following modification. The G+C content of the type-strain genome is 66.5%, its approximate size 3.40 Mbp, its GenBank deposit SAMN00767673.

#### Emended description of *Rubrobacter xylanophilus* Carreto et al. 1996

The description is as before (Carreto et al., 1996) with the following modification. The G+C content of the type-strain genome is 70.5%, its approximate size 3.23 Mbp, its GenBank deposit SAMN02598258.

#### Emended description of *Saccharomonospora azurea* Runmao 1987

The description is as before (Runmao, 1987) with the following addition. The G+C content of the type-strain genome is 70.1%, its approximate size 4.75 Mbp, its GenBank deposit SAMN02261398.

#### Emended description of *Saccharomonospora cyanea* Runmao et al. 1988

The description is as before (Runmao et al., 1988) with the following addition. The G+C content of the type-strain genome is 69.7%, its approximate size 5.41 Mbp, its GenBank deposit SAMN02256534.

#### Emended description of *Saccharomonospora glauca* Greiner-Mai et al. 1988

The description is as before (Greiner-Mai et al., 1988) with the following addition. The G+C content of the type-strain genome is 69.1%, its approximate size 4.56 Mbp, its GenBank deposit SAMN02441787.

#### Emended description of *Saccharomonospora halophila* Al-Zarban et al. 2002

The description is as before (Al-Zarban et al., 2002b) with the following addition. The G+C content of the type-strain genome is 70.9%, its approximate size 3.63 Mbp, its GenBank deposit SAMN02256397.

#### Emended description of *Saccharomonospora internatus* (Agre et al. 1974) Greiner-Mai et al. 1987

The description is as before (Greiner-Mai et al., 1987) with the following addition. The G+C content of the type-strain genome is 67.4%, its approximate size 4.31 Mbp, its GenBank deposit SAMN02982918.

#### Emended description of *Saccharomonospora viridis* (Schuurmans et al. 1956) Nonomura and Ohara 1971

The description is as before (Nonomura and Ohara, 1971a) with the following addition. The G+C content of the type-strain genome is 67.3%, its approximate size 4.31 Mbp, its GenBank deposit SAMN02598440.

#### Emended description of *Saccharomonospora xinjiangensis* Jin et al. 1998

The description is as before (Jin et al., 1998) with the following addition. The G+C content of the type-strain genome is 68.9%, its approximate size 4.77 Mbp, its GenBank deposit SAMN02261402.

#### Emended description of *Saccharopolyspora antimicrobica* Yuan et al. 2008

The description is as before (Yuan et al., 2008) with the following modification. The G+C content of the type-strain genome is 70.4%, its approximate size 8.27 Mbp, its GenBank deposit SAMN04490245.

#### Emended description of *Saccharopolyspora erythraea* (Waksman 1923) Labeda 1987

The description is as before (Labeda, 1987) with the following modification. The G+C content of the type-strain genome is 71.1%, its approximate size 8.21 Mbp, its GenBank deposit SAMEA2272514.

#### Emended description of *Saccharopolyspora flava* Lu et al. 2001

The description is as before (Lu et al., 2001) with the following modification. The G+C content of the type-strain genome is 70.8%, its approximate size 6.29 Mbp, its GenBank deposit SAMN05660874.

#### Emended description of *Saccharopolyspora rectivirgula* (Krassilnikov and Agre 1964) Korn-Wendisch et al. 1989

The description is as before (Korn-Wendisch et al., 1989) with the following modification. The G+C content of the type-strain genome is 68.9%, its approximate size 3.98 Mbp, its GenBank deposit SAMN02472042.

#### Emended description of *Saccharopolyspora spinosa* Mertz and Yao 1990

The description is as before (Mertz and Yao, 1990) with the following modification. The G+C content of the type-strain genome is 67.9%, its approximate size 9.02 Mbp, its GenBank deposit SAMN05216473.

#### Emended description of *Saccharothrix syringae* (Gause and Sveshnikova 1985) Grund and Kroppenstedt 1990

The description is as before (Grund and Kroppenstedt, 1990) with the following addition. The G+C content of the type-strain genome is 73.5%, its approximate size 10.88 Mbp, its GenBank deposit SAMN02645315.

#### Emended description of *Salinispora pacifica* Ahmed et al. 2014

The description is as before (Ahmed et al., 2013) with the following restriction. The G+C content of the type-strain genome is 69.7%, its approximate size 5.87 Mbp, its GenBank deposit SAMN02597245.

#### Emended description of *Salinispora tropica* Maldonado et al. 2005

The description is as before (Maldonado et al., 2005) with the following addition. The G+C content of the type-strain genome is 69.5%, its approximate size 5.18 Mbp, its GenBank deposit SAMN02598367.

#### Emended description of *Sanguibacter keddieii* Fernández-Garayzábal et al. 1995 emend. Pikuta et al. 2017

The description is as before (Pikuta et al., 2017) with the following modification. The G+C content of the type-strain genome is 71.9%, its approximate size 4.25 Mbp, its GenBank deposit SAMN02598426.

#### Emended description of *Scardovia inopinata* (Crociani et al. 1996) Jian and Dong 2002 emend. Downes et al. 2011

The description is as before (Downes et al., 2011) with the following modification. The G+C content of the type-strain genome is 48.6%, its approximate size 1.80 Mbp, its GenBank deposit SAMD00061049.

#### Emended description of *Sciscionella marina* Tian et al. 2009

The description is as before (Tian et al., 2009) with the following modification. The G+C content of the type-strain genome is 67.8%, its approximate size 8.54 Mbp, its GenBank deposit SAMN02256422.

#### Emended description of *Segniliparus rotundus* Butler et al. 2005

The description is as before (Butler et al., 2005) with the following modification. The G+C content of the type-strain genome is 66.8%, its approximate size 3.16 Mbp, its GenBank deposit SAMN02598516.

#### Emended description of *Segniliparus rugosus* Butler et al. 2005

The description is as before (Butler et al., 2005) with the following modification. The G+C content of the type-strain genome is 68.2%, its approximate size 3.59 Mbp, its GenBank deposit SAMN02463810.

#### Emended description of *Senegalimassilia anaerobia* Lagier et al. 2014

The description is as before (Lagier et al., 2013) with the following modification. The G+C content of the type-strain genome is 62.1%, its GenBank deposit SAMEA2272806.

#### Emended description of *Sinomonas atrocyanea* (Kuhn and Starr 1960) Zhou et al. 2009

The description is as before (Zhou et al., 2009) with the following modification. The G+C content of the type-strain genome is 71.4%, its approximate size 4.49 Mbp, its GenBank deposit SAMN04508113.

#### Emended description of *Sinomonas mesophila* Prabhu et al. 2015

The description is as before (Prabhu et al., 2015) with the following modification. The G+C content of the type-strain genome is 71.1%, its approximate size 4.01 Mbp, its GenBank deposit SAMN06212359.

#### Emended description of *Skermania piniformis* (Blackall et al. 1989) Chun et al. 1997

The description is as before (Chun et al., 1997a) with the following modification. The G+C content of the type-strain genome is 68.6%, its approximate size 4.21 Mbp, its GenBank deposit SAMD00046514.

#### Emended description of *Slackia exigua* (Poco et al. 1996) Wade et al. 1999

The description is as before (Wade et al., 1999) with the following restriction. The G+C content of the type-strain genome is 62.2%, its approximate size 2.09 Mbp, its GenBank deposit SAMN00008861.

#### Emended description of *Slackia heliotrinireducens* (Lanigan 1983) Wade et al. 1999

The description is as before (Wade et al., 1999) with the following modification. The G+C content of the type-strain genome is 60.2%, its approximate size 3.17 Mbp, its GenBank deposit SAMN02598438.

#### Emended description of *Sphaerimonospora mesophila* (Nonomura and Ohara 1971) Mingma et al. 2016

The description is as before (Mingma et al., 2016) with the following modification. The G+C content of the type-strain genome is 70.0%, its approximate size 7.34 Mbp, its GenBank deposit SAMD00046509.

#### Emended description of *Sphaerobacter thermophilus* Demharter et al. 1989 emend. Houghton et al. 2015

The description is as before (Houghton et al., 2015) with the following modification. The G+C content of the type-strain genome is 68.1%, its approximate size 3.99 Mbp, its GenBank deposit SAMN02598446.

#### Emended description of *Spirillospora albida* Couch 1963

The description is as before (Couch, 1963) with the following addition. The G+C content of the type-strain genome is 72.6%, its approximate size 6.03 Mbp, its GenBank deposit SAMN02645261.

#### Emended description of *Sporichthya polymorpha* Lechevalier et al. 1968

The description is as before (Lechevalier et al., 1968) with the following addition. The G+C content of the type-strain genome is 71.4%, its approximate size 5.50 Mbp, its GenBank deposit SAMN02261321.

#### Emended description of *Stackebrandtia nassauensis* Labeda and Kroppenstedt 2005

The description is as before (Labeda and Kroppenstedt, 2005) with the following modification. The G+C content of the type-strain genome is 68.1%, its approximate size 6.84 Mbp, its GenBank deposit SAMN02598427.

#### Emended description of *Streptacidiphilus albus* Kim et al. 2003

The description is as before (Kim et al., 2003) with the following addition. The G+C content of the type-strain genome is 71.8%, its approximate size 9.92 Mbp, its GenBank deposit SAMN02745413.

#### Emended description of *Streptacidiphilus anmyonensis* Cho et al. 2008

The description is as before (Cho et al., 2008) with the following addition. The G+C content of the type-strain genome is 72.2%, its approximate size 9.32 Mbp, its GenBank deposit SAMD00022877.

#### Emended description of *Streptacidiphilus carbonis* Kim et al. 2003

The description is as before (Kim et al., 2003) with the following addition. The G+C content of the type-strain genome is 71.5%, its approximate size 8.41 Mbp, its GenBank deposit SAMD00022873.

#### Emended description of *Streptacidiphilus melanogenes* Cho et al. 2008

The description is as before (Cho et al., 2008) with the following addition. The G+C content of the type-strain genome is 72.0%, its approximate size 8.72 Mbp, its GenBank deposit SAMD00022876.

#### Emended description of *Streptacidiphilus neutrinimicus* Kim et al. 2003

The description is as before (Kim et al., 2003) with the following addition. The G+C content of the type-strain genome is 72.2%, its approximate size 8.33 Mbp, its GenBank deposit SAMD00022875.

#### Emended description of *Streptacidiphilus oryzae* Wang et al. 2006

The description is as before (Wang et al., 2006) with the following addition. The G+C content of the type-strain genome is 73.4%, its approximate size 7.81 Mbp, its GenBank deposit SAMN02745412.

#### Emended description of *Streptacidiphilus rugosus* Cho et al. 2008

The description is as before (Cho et al., 2008) with the following addition. The G+C content of the type-strain genome is 71.8%, its approximate size 9.00 Mbp, its GenBank deposit SAMN02745414.

#### Emended description of *Streptoalloteichus hindustanus* (ex Tomita et al. 1978) Tomita et al. 1987

The description is as before (Tomita et al., 1987) with the following addition. The G+C content of the type-strain genome is 72.6%, its approximate size 7.28 Mbp, its GenBank deposit SAMN05444320.

#### Emended description of *Streptomonospora alba* Li et al. 2003

The description is as before (Li et al., 2003d) with the following modification. The G+C content of the type-strain genome is 71.9%, its approximate size 5.91 Mbp, its GenBank deposit SAMN03074668.

#### Emended description of *Streptomyces achromogenes* Okami and Umezawa 1953

The description is as before (Umezawa et al., 1953) with the following addition. The G+C content of the type-strain genome is 72.5%, its approximate size 8.11 Mbp, its GenBank deposit SAMN02645217.

#### Emended description of *Streptomyces aidingensis* Xia et al. 2013

The description is as before (Xia et al., 2013) with the following modification. The G+C content of the type-strain genome is 73.2%, its approximate size 6.47 Mbp, its GenBank deposit SAMN05421773.

#### Emended description of *Streptomyces albidoflavus* (Rossi Doria 1891) Waksman and Henrici 1948 emend. Rong et al. 2009

The description is as before (Rong et al., 2009) with the following restriction. The G+C content of the type-strain genome is 73.4%, its approximate size 7.08 Mbp, its GenBank deposit SAMN02645503.

#### Emended description of *Streptomyces alboflavus* (Waksman and Curtis 1916) Waksman and Henrici 1948

The description is as before (Waksman and Henrici, 1948) with the following addition. The G+C content of the type-strain genome is 72.2%, its approximate size 9.72 Mbp, its GenBank deposit SAMN02645294.

#### Emended description of *Streptomyces albus* (Rossi Doria 1891) Waksman and Henrici 1943 emend. Labeda et al. 2014

The description is as before (Labeda et al., 2014) with the following addition. The G+C content of the type-strain genome is 72.7%, its approximate size 7.59 Mbp, its GenBank deposit SAMD00023485.

#### Emended description of *Streptomyces alni* Liu et al. 2009

The description is as before (Liu et al., 2009b) with the following modification. The G+C content of the type-strain genome is 72.1%, its approximate size 8.27 Mbp, its GenBank deposit SAMN05216251.

#### Emended description of *Streptomyces ambofaciens* Pinnert-Sindico 1954

The description is as before (Pinnert-Sindico, 1954) with the following addition. The G+C content of the type-strain genome is 72.2%, its approximate size 8.39 Mbp, its GenBank deposit SAMN03921868.

#### Emended description of *Streptomyces amritsarensis* Sharma et al. 2014

The description is as before (Sharma et al., 2014) with the following restriction. The G+C content of the type-strain genome is 72.6%, its approximate size 7.82 Mbp, its GenBank deposit SAMN04386232.

#### Emended description of *Streptomyces antibioticus* (Waksman and Woodruff 1941) Waksman and Henrici 1948

The description is as before (Waksman and Henrici, 1948) with the following addition. The G+C content of the type-strain genome is 70.8%, its approximate size 10.12 Mbp, its GenBank deposit SAMN04193346.

#### Emended description of *Streptomyces atroolivaceus* (Preobrazhenskaya et al. 1957) Pridham et al. 1958

The description is as before (Pridham et al., 1958) with the following addition. The G+C content of the type-strain genome is 70.7%, its approximate size 8.22 Mbp, its GenBank deposit SAMN02645281.

#### Emended description of *Streptomyces aureocirculatus* (Krassilnikov and Yuan 1965) Pridham 1970

The description is as before (Pridham, 1970) with the following addition. The G+C content of the type-strain genome is 71.2%, its approximate size 9.32 Mbp, its GenBank deposit SAMN02645370.

#### Emended description of *Streptomyces azureus* Kelly et al. 1959

The description is as before (Kelly et al., 1959) with the following addition. The G+C content of the type-strain genome is 70.9%, its approximate size 8.79 Mbp, its GenBank deposit SAMD00031377.

#### Emended description of *Streptomyces baldaccii* (Farina and Locci 1966) Witt and Stackebrandt 1991

The description is as before (Witt and Stackebrandt, 1990) with the following addition. The G+C content of the type-strain genome is 72.4%, its approximate size 7.21 Mbp, its GenBank deposit SAMN02645263.

#### Emended description of *Streptomyces bikiniensis* Johnstone and Waksman 1947

The description is as before (Johstone and Waksman, 1947) with the following addition. The G+C content of the type-strain genome is 73.1%, its approximate size 7.39 Mbp, its GenBank deposit SAMN02645190.

#### Emended description of *Streptomyces bottropensis* Waksman 1961

The description is as before (Waksman, 1961) with the following addition. The G+C content of the type-strain genome is 71.2%, its approximate size 8.91 Mbp, its GenBank deposit SAMN02471210.

#### Emended description of *Streptomyces bungoensis* Eguchi et al. 1993

The description is as before (Eguchi et al., 1993) with the following modification. The G+C content of the type-strain genome is 72.7%, its approximate size 8.33 Mbp, its GenBank deposit SAMN04193354.

#### Emended description of *Streptomyces caeruleus* (Baldacci 1944) Pridham et al. 1958 emend. Lanoot et al. 2002

The description is as before (Lanoot et al., 2002) with the following modification. The G+C content of the type-strain genome is 72.5%, its approximate size 6.03 Mbp, its GenBank deposit SAMN02645219.

#### Emended description of *Streptomyces californicus* (Waksman and Curtis 1916) Waksman and Henrici 1948

The description is as before (Waksman and Henrici, 1948) with the following addition. The G+C content of the type-strain genome is 72.7%, its approximate size 7.79 Mbp, its GenBank deposit SAMN02645354.

#### Emended description of *Streptomyces canus* Heinemann et al. 1953

The description is as before (Heinemann et al., 1953) with the following addition. The G+C content of the type-strain genome is 70.2%, its approximate size 11.50 Mbp, its GenBank deposit SAMN04193350.

#### Emended description of *Streptomyces catenulae* Davisson and Finlay 1961

The description is as before (Waksman, 1961) with the following addition. The G+C content of the type-strain genome is 73.0%, its approximate size 7.05 Mbp, its GenBank deposit SAMN02645222.

#### Emended description of *Streptomyces cellostaticus* Hamada 1958

The description is as before (Hamada, 1958) with the following addition. The G+C content of the type-strain genome is 71.0%, its approximate size 9.68 Mbp, its GenBank deposit SAMN04193341.

#### Emended description of *Streptomyces celluloflavus* Nishimura et al. 1953

The description is as before (Nishimura et al., 1953) with the following addition. The G+C content of the type-strain genome is 71.7%, its approximate size 8.68 Mbp, its GenBank deposit SAMN02645237.

#### Emended description of *Streptomyces cinnabarinus* (Ryabova and Preobrazhenskaya 1957) Pridham et al. 1958

The description is as before (Pridham et al., 1958) with the following addition. The G+C content of the type-strain genome is 70.7%, its approximate size 9.35 Mbp, its GenBank deposit SAMN02645291.

#### Emended description of *Streptomyces ciscaucasicus* Sveshnikova 1986

The description is as before (Gause et al., 1983) with the following addition. The G+C content of the type-strain genome is 70.3%, its approximate size 9.79 Mbp, its GenBank deposit SAMN04193344.

#### Emended description of *Streptomyces citreofluorescens* (Korenyako et al. 1960) Pridham 1970

The description is as before (Pridham, 1970) with the following addition. The G+C content of the type-strain genome is 71.3%, its approximate size 8.40 Mbp, its GenBank deposit SAMN02645293.

#### Emended description of *Streptomyces clavuligerus* Higgens and Kastner 1971

The description is as before (Higgens and Kastner, 1971) with the following addition. The G+C content of the type-strain genome is 72.5%, its approximate size 8.53 Mbp, its GenBank deposit SAMN02471788.

#### Emended description of *Streptomyces corchorusii* Ahmad and Bhuiyan 1958 emend. Lanoot et al. 2005

The description is as before (Lanoot et al., 2005) with the following addition. The G+C content of the type-strain genome is 72.0%, its approximate size 10.27 Mbp, its GenBank deposit SAMN04193345.

#### Emended description of *Streptomyces curacoi* Cataldi 1963

The description is as before (Trejo and Bennett, 1963) with the following addition. The G+C content of the type-strain genome is 71.0%, its approximate size 8.39 Mbp, its GenBank deposit SAMN04193339.

#### Emended description of *Streptomyces cyaneofuscatus* (Kudrina 1957) Pridham et al. 1958

The description is as before (Pridham et al., 1958) with the following addition. The G+C content of the type-strain genome is 71.6%, its approximate size 7.90 Mbp, its GenBank deposit SAMN02645238.

#### Emended description of *Streptomyces durhamensis* Gordon and Lapa 1966

The description is as before (Gordon and Lapa, 1966) with the following addition. The G+C content of the type-strain genome is 71.7%, its approximate size 9.08 Mbp, its GenBank deposit SAMN02645292.

#### Emended description of *Streptomyces emeiensis* Sun et al. 2007

The description is as before (Sun et al., 2007) with the following modification. The G+C content of the type-strain genome is 72.9%, its approximate size 7.12 Mbp, its GenBank deposit SAMN05216505.

#### Emended description of *Streptomyces exfoliatus* (Waksman and Curtis 1916) Waksman and Henrici 1948

The description is as before (Waksman and Henrici, 1948) with the following addition. The G+C content of the type-strain genome is 72.0%, its approximate size 7.53 Mbp, its GenBank deposit SAMN02645344.

#### Emended description of *Streptomyces flavovariabilis* (ex Korenyako and Nikitina 1965) Sveshnikova 1986

The description is as before (Gause et al., 1983) with the following addition. The G+C content of the type-strain genome is 71.2%, its approximate size 8.76 Mbp, its GenBank deposit SAMN02645208.

#### Emended description of *Streptomyces floridae* Bartz et al. 1951

The description is as before (Bartz et al., 1951) with the following addition. The G+C content of the type-strain genome is 72.7%, its approximate size 7.90 Mbp, its GenBank deposit SAMN02645357.

#### Emended description of *Streptomyces fluorescens* (Krassilnikov 1958) Pridham 1970

The description is as before (Pridham, 1970) with the following addition. The G+C content of the type-strain genome is 71.3%, its approximate size 8.40 Mbp, its GenBank deposit SAMN02645251.

#### Emended description of *Streptomyces ghanaensis* Wallhäusser et al. 1966

The description is as before (Wallhausser et al., 1965) with the following addition. The G+C content of the type-strain genome is 72.2%, its approximate size 8.22 Mbp, its GenBank deposit SAMN02595233.

#### Emended description of *Streptomyces griseofuscus* Sakamoto et al. 1962

The description is as before (Sakamoto et al., 1962) with the following addition. The G+C content of the type-strain genome is 71.7%, its approximate size 8.95 Mbp, its GenBank deposit SAMN02645272.

#### Emended description of *Streptomyces griseolus* (Waksman 1923) Waksman and Henrici 1948

The description is as before (Waksman and Henrici, 1948) with the following addition. The G+C content of the type-strain genome is 72.0%, its approximate size 7.54 Mbp, its GenBank deposit SAMN02645254.

#### Emended description of *Streptomyces griseoruber* Yamaguchi and Saburi 1955

The description is as before (Yamaguchi and Saburi, 1955) with the following addition. The G+C content of the type-strain genome is 71.7%, its approximate size 10.11 Mbp, its GenBank deposit SAMN04193353.

#### Emended description of *Streptomyces griseorubiginosus* (Ryabova and Preobrazhenskaya 1957) Pridham et al. 1958

The description is as before (Pridham et al., 1958) with the following addition. The G+C content of the type-strain genome is 70.9%, its approximate size 9.83 Mbp, its GenBank deposit SAMN04193351.

#### Emended description of *Streptomyces griseus* (Krainsky 1914) Waksman and Henrici 1948 emend. Liu et al. 2005

The description is as before (Liu et al., 2005) with the following modification. The G+C content of the type-strain genome is 72.2%, its approximate size 8.63 Mbp, its GenBank deposit SAMN04490359.

#### Emended description of *Streptomyces halstedii* (Waksman and Curtis 1916) Waksman and Henrici 1948

The description is as before (Waksman and Henrici, 1948) with the following addition. The G+C content of the type-strain genome is 71.9%, its approximate size 7.74 Mbp, its GenBank deposit SAMN02645380.

#### Emended description of *Streptomyces indicus* Luo et al. 2011

The description is as before (Luo et al., 2011) with the following restriction. The G+C content of the type-strain genome is 71.4%, its approximate size 8.23 Mbp, its GenBank deposit SAMN05421806.

#### Emended description of *Streptomyces kebangsaanensis* Sarmin et al. 2013

The description is as before (Sarmin et al., 2013) with the following modification. The G+C content of the type-strain genome is 71.6%, its approximate size 8.22 Mbp, its GenBank deposit SAMN03254380.

#### Emended description of *Streptomyces laurentii* Trejo et al. 1979

The description is as before (Trejo et al., 1977) with the following addition. The G+C content of the type-strain genome is 72.3%, its approximate size 8.03 Mbp, its GenBank deposit SAMD00050872.

#### Emended description of *Streptomyces lavendulae* (Waksman and Curtis 1916) Waksman and Henrici 1948

The description is as before (Waksman and Henrici, 1948) with the following addition. The G+C content of the type-strain genome is 72.7%, its approximate size 8.47 Mbp, its GenBank deposit SAMN02645248.

#### Emended description of *Streptomyces lincolnensis* Mason et al. 1963

The description is as before (Mason et al., 1963) with the following addition. The G+C content of the type-strain genome is 71.0%, its approximate size 10.32 Mbp, its GenBank deposit SAMN05335414.

#### Emended description of *Streptomyces longwoodensis* Prosser and Palleroni 1981

The description is as before (Prosser and Palleroni, 1976) with the following addition. The G+C content of the type-strain genome is 73.1%, its approximate size 8.34 Mbp, its GenBank deposit SAMN04193348.

#### Emended description of *Streptomyces luteus* Luo et al. 2017

The description is as before (Luo et al., 2017) with the following modification. The G+C content of the type-strain genome is 71.6%, its approximate size 7.83 Mbp, its GenBank deposit SAMN02803977.

#### Emended description of *Streptomyces lydicus* De Boer et al. 1956

The description is as before (De Boer et al., 1956) with the following addition. The G+C content of the type-strain genome is 72.1%, its approximate size 8.18 Mbp, its GenBank deposit SAMN02645328.

#### Emended description of *Streptomyces megasporus* (ex Krassilnikov et al. 1968) Agre 1986

The description is as before (Gause et al., 1983) with the following addition. The G+C content of the type-strain genome is 72.3%, its approximate size 5.85 Mbp, its GenBank deposit SAMN02645209.

#### Emended description of *Streptomyces melanosporofaciens* Arcamone et al. 1959

The description is as before (Arcamone et al., 1959) with the following addition. The G+C content of the type-strain genome is 71.0%, its approximate size 10.77 Mbp, its GenBank deposit SAMN04490356.

#### Emended description of *Streptomyces misionensis* Cercos et al. 1962

The description is as before (Cercos et al., 1962) with the following addition. The G+C content of the type-strain genome is 72.4%, its approximate size 8.47 Mbp, its GenBank deposit SAMN04490357.

#### Emended description of *Streptomyces mobaraensis* (Nagatsu and Suzuki 1963) Witt and Stackebrandt 1991

The description is as before (Witt and Stackebrandt, 1990) with the following addition. The G+C content of the type-strain genome is 73.3%, its approximate size 7.48 Mbp, its GenBank deposit SAMN02470112.

#### Emended description of *Streptomyces monomycini* Gause and Terekhova 1986

The description is as before (Gause et al., 1983) with the following addition. The G+C content of the type-strain genome is 72.1%, its approximate size 7.80 Mbp, its GenBank deposit SAMN02645312.

#### Emended description of *Streptomyces niger* (Thirumalachar 1955) Goodfellow et al. 1986

The description is as before (Goodfellow et al., 1986b) with the following addition. The G+C content of the type-strain genome is 71.7%, its approximate size 8.49 Mbp, its GenBank deposit SAMN02645268.

#### Emended description of *Streptomyces nodosus* Trejo 1961

The description is as before (Waksman, 1961) with the following addition. The G+C content of the type-strain genome is 70.9%, its approximate size 7.71 Mbp, its GenBank deposit SAMN03020625.

#### Emended description of *Streptomyces noursei* Brown et al. 1953

The description is as before (Brown et al., 1953) with the following addition. The G+C content of the type-strain genome is 71.5%, its approximate size 9.82 Mbp, its GenBank deposit SAMN03287657.

#### Emended description of *Streptomyces ochraceiscleroticus* Pridham 1970

The description is as before (Pridham, 1970) with the following addition. The G+C content of the type-strain genome is 71.4%, its approximate size 8.41 Mbp, its GenBank deposit SAMN02645378.

#### Emended description of *Streptomyces olivaceus* (Waksman 1923) Waksman and Henrici 1948

The description is as before (Waksman and Henrici, 1948) with the following addition. The G+C content of the type-strain genome is 72.4%, its approximate size 8.58 Mbp, its GenBank deposit SAMN02645259.

#### Emended description of *Streptomyces olivochromogenes* (Waksman 1923) Waksman and Henrici 1948

The description is as before (Waksman and Henrici, 1948) with the following addition. The G+C content of the type-strain genome is 70.2%, its approximate size 11.61 Mbp, its GenBank deposit SAMN04193347.

#### Emended description of *Streptomyces paucisporeus* Xu et al. 2006

The description is as before (Xu et al., 2006) with the following modification. The G+C content of the type-strain genome is 72.3%, its approximate size 8.16 Mbp, its GenBank deposit SAMN05216499.

#### Emended description of *Streptomyces phaeopurpureus* Shinobu 1957 emend. Lanoot et al. 2004

The description is as before (Lanoot et al., 2004) with the following modification. The G+C content of the type-strain genome is 71.0%, its approximate size 9.54 Mbp, its GenBank deposit SAMN04193340.

#### Emended description of *Streptomyces pini* Madhaiyan et al. 2016

The description is as before (Madhaiyan et al., 2016) with the following modification. The G+C content of the type-strain genome is 73.0%, its approximate size 6.34 Mbp, its GenBank deposit SAMN05192584.

#### Emended description of *Streptomyces platensis* Tresner and Backus 1956

The description is as before (Tresner and Backus, 1956) with the following addition. The G+C content of the type-strain genome is 71.2%, its approximate size 8.40 Mbp, its GenBank deposit SAMN05722965.

#### Emended description of *Streptomyces pluripotens* Lee et al. 2014

The description is as before (Lee et al., 2014b) with the following restriction. The G+C content of the type-strain genome is 70.0%, its approximate size 7.48 Mbp, its GenBank deposit SAMN03070117.

#### Emended description of *Streptomyces prunicolor* (Ryabova and Preobrazhenskaya 1957) Pridham et al. 1958

The description is as before (Pridham et al., 1958) with the following addition. The G+C content of the type-strain genome is 69.7%, its approximate size 11.76 Mbp, its GenBank deposit SAMD00041815.

#### Emended description of *Streptomyces pseudovenezuelae* (Kuchaeva et al. 1961) Pridham 1970

The description is as before (Pridham, 1970) with the following addition. The G+C content of the type-strain genome is 71.0%, its approximate size 9.58 Mbp, its GenBank deposit SAMN04193342.

#### Emended description of *Streptomyces qinglanensis* Hu et al. 2012

The description is as before (Hu et al., 2012) with the following modification. The G+C content of the type-strain genome is 72.7%, its approximate size 7.17 Mbp, its GenBank deposit SAMN05421870.

#### Emended description of *Streptomyces resistomycificus* Lindenbein 1952

The description is as before (Lindenbein, 1952) with the following addition. The G+C content of the type-strain genome is 71.0%, its approximate size 9.15 Mbp, its GenBank deposit SAMN04193356.

#### Emended description of *Streptomyces rimosus* Sobin et al. 1953

The description is as before (Waksman and Lechevalier, 1953) with the following addition. The G+C content of the type-strain genome is 71.9%, its approximate size 9.50 Mbp, its GenBank deposit SAMN02471950.

#### Emended description of *Streptomyces rubidus* Xu et al. 2006

The description is as before (Xu et al., 2006) with the following modification. The G+C content of the type-strain genome is 72.9%, its approximate size 9.01 Mbp, its GenBank deposit SAMN05216267.

#### Emended description of *Streptomyces sclerotialus* Pridham 1970

The description is as before (Pridham, 1970) with the following addition. The G+C content of the type-strain genome is 71.9%, its approximate size 8.23 Mbp, its GenBank deposit SAMN02645383.

#### Emended description of *Streptomyces seoulensis* Chun et al. 1997

The description is as before (Chun et al., 1997b) with the following modification. The G+C content of the type-strain genome is 71.9%, its approximate size 5.87 Mbp, its GenBank deposit SAMN02645290.

#### Emended description of *Streptomyces somaliensis* (Brumpt 1906) Waksman and Henrici 1948

The description is as before (Waksman and Henrici, 1948) with the following addition. The G+C content of the type-strain genome is 74.1%, its approximate size 5.18 Mbp, its GenBank deposit SAMN02471954.

#### Emended description of *Streptomyces specialis* Kämpfer et al. 2008

The description is as before (Kämpfer et al., 2008) with the following addition. The G+C content of the type-strain genome is 72.7%, its approximate size 5.86 Mbp, its GenBank deposit SAMEA3696889.

#### Emended description of *Streptomyces spheroides* Wallick et al. 1956

The description is as before (Wallick et al., 1956) with the following addition. The G+C content of the type-strain genome is 70.7%, its approximate size 8.57 Mbp, its GenBank deposit SAMN02469390.

#### Emended description of *Streptomyces sporocinereus* (ex Krassilnikov 1970) Preobrazhenskaya 1986

The description is as before (Gause et al., 1983) with the following addition. The G+C content of the type-strain genome is 72.0%, its approximate size 10.15 Mbp, its GenBank deposit SAMD00040622.

#### Emended description of *Streptomyces sulphureus* (Gasperini 1894) Waksman 1953

The description is as before (Waksman and Lechevalier, 1953) with the following addition. The G+C content of the type-strain genome is 71.7%, its approximate size 7.10 Mbp, its GenBank deposit SAMN02440478.

#### Emended description of *Streptomyces varsoviensis* Kurylowicz and Woznicka 1967

The description is as before (Kurylowicz and Wóznicka, 1967) with the following addition. The G+C content of the type-strain genome is 72.4%, its approximate size 8.59 Mbp, its GenBank deposit SAMN02645386.

#### Emended description of *Streptomyces venezuelae* Ehrlich et al. 1948

The description is as before (Ehrlich et al., 1948) with the following addition. The G+C content of the type-strain genome is 72.5%, its approximate size 8.22 Mbp, its GenBank deposit SAMEA3138410.

#### Emended description of *Streptomyces vietnamensis* Zhu et al. 2007

The description is as before (Zhu et al., 2007) with the following modification. The G+C content of the type-strain genome is 72.0%, its approximate size 9.15 Mbp, its GenBank deposit SAMN02730132.

#### Emended description of *Streptomyces violaceorubidus* Terekhova 1986

The description is as before (Gause et al., 1983) with the following addition. The G+C content of the type-strain genome is 72.2%, its approximate size 7.65 Mbp, its GenBank deposit SAMN02645210.

#### Emended description of *Streptomyces virginiae* Grundy et al. 1952

The description is as before (Grundy et al., 1952) with the following addition. The G+C content of the type-strain genome is 72.4%, its approximate size 8.32 Mbp, its GenBank deposit SAMN02645365.

#### Emended description of *Streptomyces vitaminophilus* (Shomura et al. 1983) Goodfellow et al. 1986

The description is as before (Goodfellow et al., 1986a) with the following addition. The G+C content of the type-strain genome is 72.0%, its approximate size 6.43 Mbp, its GenBank deposit SAMN02256407.

#### Emended description of *Streptomyces wedmorensis* (ex Milard and Burr 1926) Preobrazhenskaya 1986

The description is as before (Gause et al., 1983) with the following addition. The G+C content of the type-strain genome is 72.1%, its approximate size 9.38 Mbp, its GenBank deposit SAMN02645189.

#### Emended description of *Streptomyces xanthophaeus* Lindenbein 1952

The description is as before (Lindenbein, 1952) with the following addition. The G+C content of the type-strain genome is 72.1%, its approximate size 6.29 Mbp, its GenBank deposit SAMN02645271.

#### Emended description of *Streptomyces yanglinensis* Xu et al. 2006

The description is as before (Xu et al., 2006) with the following modification. The G+C content of the type-strain genome is 72.6%, its approximate size 9.59 Mbp, its GenBank deposit SAMN05216223.

#### Emended description of *Streptomyces yeochonensis* Kim et al. 2004

The description is as before (Kim et al., 2004) with the following addition. The G+C content of the type-strain genome is 73.6%, its approximate size 7.82 Mbp, its GenBank deposit SAMN02745173.

#### Emended description of *Streptomyces yokosukanensis* Nakamura 1961

The description is as before (Nakamura, 1961) with the following addition. The G+C content of the type-strain genome is 71.2%, its approximate size 10.01 Mbp, its GenBank deposit SAMN04193343.

#### Emended description of *Streptosporangium amethystogenes* Nonomura and Ohara 1960

The description is as before (Nonomura and Ohara, 1960) with the following addition. The G+C content of the type-strain genome is 69.4%, its approximate size 9.44 Mbp, its GenBank deposit SAMN02645242.

#### Emended description of *Streptosporangium canum* Zhang et al. 2009

The description is as before (Zhang et al., 2009b) with the following modification. The G+C content of the type-strain genome is 70.7%, its approximate size 9.26 Mbp, its GenBank deposit SAMN05216275.

#### Emended description of *Streptosporangium roseum* Couch 1955

The description is as before (Couch, 1955) with the following addition. The G+C content of the type-strain genome is 70.9%, its approximate size 10.37 Mbp, its GenBank deposit SAMN00002585.

#### Emended description of *Tessaracoccus bendigoensis* Maszenan et al. 1999

The description is as before (Maszenan et al., 1999b) with the following modification. The G+C content of the type-strain genome is 66.7%, its approximate size 4.13 Mbp, its GenBank deposit SAMN02745244.

#### Emended description of *Tessaracoccus flavus* Kumari et al. 2016

The description is as before (Kumari et al., 2016) with the following modification. The G+C content of the type-strain genome is 68.0%, its approximate size 3.19 Mbp, its GenBank deposit SAMN05428934.

#### Emended description of *Tessaracoccus oleiagri* Cai et al. 2011

The description is as before (Cai et al., 2011) with the following restriction. The G+C content of the type-strain genome is 69.7%, its approximate size 3.16 Mbp, its GenBank deposit SAMN04488242.

#### Emended description of *Tetrasphaera jenkinsii* McKenzie et al. 2006

The description is as before (McKenzie et al., 2006) with the following addition. The G+C content of the type-strain genome is 68.2%, its approximate size 3.79 Mbp, its GenBank deposit SAMEA3146295.

#### Emended description of *Thermoactinospora rubra* Zhou et al. 2012

The description is as before (Zhou et al., 2012a) with the following modification. The G+C content of the type-strain genome is 71.7%, its approximate size 8.22 Mbp, its GenBank deposit SAMN06041775.

#### Emended description of *Thermobifida cellulosilytica* Kukolya et al. 2002

The description is as before (Kukolya et al., 2002) with the following modification. The G+C content of the type-strain genome is 72.1%, its approximate size 4.32 Mbp, its GenBank deposit SAMN03840822.

#### Emended description of *Thermobifida fusca* (McCarthy and Cross, 1984) Zhang et al. 1998

The description is as before (Zhang et al., 1998) with the following addition. The G+C content of the type-strain genome is 67.5%, its approximate size 3.64 Mbp, its GenBank deposit SAMD00046774.

#### Emended description of *Thermobifida halotolerans* Yang et al. 2008

The description is as before (Yang et al., 2008) with the following modification. The G+C content of the type-strain genome is 71.2%, its approximate size 5.43 Mbp, its GenBank deposit SAMN04009169.

#### Emended description of *Thermobispora bispora* (Henssen, 1957) Wang et al. 1996

The description is as before (Wang et al., 1996) with the following addition. The G+C content of the type-strain genome is 72.4%, its approximate size 4.19 Mbp, its GenBank deposit SAMN00002587.

#### Emended description of *Thermocrispum agreste* Korn-Wendisch et al. 1995

The description is as before (Korn-Wendisch et al., 1995) with the following addition. The G+C content of the type-strain genome is 69.9%, its approximate size 4.20 Mbp, its GenBank deposit SAMN02441562.

#### Emended description of *Thermocrispum municipale* Korn-Wendisch et al. 1995

The description is as before (Korn-Wendisch et al., 1995) with the following addition. The G+C content of the type-strain genome is 68.9%, its approximate size 4.53 Mbp, its GenBank deposit SAMN02440599.

#### Emended description of *Thermoflexus hugenholtzii* Dodsworth et al. 2014

The description is as before (Dodsworth et al., 2014) with the following modification. The G+C content of the type-strain genome is 67.3%, its approximate size 3.22 Mbp, its GenBank deposit SAMN02746019.

#### Emended description of *Thermogemmatispora carboxidivorans* King and King 2014

The description is as before (King and King, 2014) with the following modification. The G+C content of the type-strain genome is 60.9%, its approximate size 5.61 Mbp, its GenBank deposit SAMN02841144.

#### Emended description of *Thermoleophilum album* Zarilla and Perry 1986

The description is as before (Zarilla and Perry, 1984) with the following modification. The G+C content of the type-strain genome is 69.1%, its approximate size 2.21 Mbp, its GenBank deposit SAMN02745716.

#### Emended description of *Thermomonospora curvata* Henssen 1957

The description is as before (Henssen, 1957) with the following addition. The G+C content of the type-strain genome is 71.6%, its approximate size 5.64 Mbp, its GenBank deposit SAMN02598435.

#### Emended description of *Thermostaphylospora chromogena* Wu et al. 2018

The description is as before (Wu et al., 2018) with the following modification. The G+C content of the type-strain genome is 71.0%, its approximate size 5.99 Mbp, its GenBank deposit SAMN04489764.

#### Emended description of *Tomitella biformata* Katayama et al. 2010

The description is as before (Katayama et al., 2010) with the following modification. The G+C content of the type-strain genome is 68.1%, its approximate size 4.71 Mbp, its GenBank deposit SAMD00036785.

#### Emended description of *Tropheryma whipplei* La Scola et al. 2001

The description is as before (La Scola et al., 2001) with the following modification. The G+C content of the type-strain genome is 46.3%, its approximate size 0.93 Mbp, its GenBank deposit SAMN02603248.

#### Emended description of *Trueperella bernardiae* (Funke et al., 1995) Yassin et al. 2011

The description is as before (Yassin et al., 2011) with the following restriction. The G+C content of the type-strain genome is 65.4%, its approximate size 2.03 Mbp, its GenBank deposit SAMN04196844.

#### Emended description of *Tsukamurella carboxydivorans* Park et al. 2009

The description is as before (Park et al., 2009) with the following modification. The G+C content of the type-strain genome is 71.0%, its approximate size 4.95 Mbp, its GenBank deposit SAMN04457857.

#### Emended description of *Tsukamurella paurometabola* (Steinhaus 1941) Collins et al. 1988

The description is as before (Collins et al., 1988b) with the following modification. The G+C content of the type-strain genome is 68.4%, its approximate size 4.48 Mbp, its GenBank deposit SAMN00002597.

#### Emended description of *Tsukamurella spongiae* Olson et al. 2007

The description is as before (Olson et al., 2007) with the following modification. The G+C content of the type-strain genome is 71.0%, its approximate size 4.59 Mbp, its GenBank deposit SAMN04457855.

#### Emended description of *Tsukamurella sunchonensis* Seong et al. 2008

The description is as before (Seong et al., 2003) with the following modification. The G+C content of the type-strain genome is 70.1%, its approximate size 5.21 Mbp, its GenBank deposit SAMN04457858.

#### Emended description of *Umezawaea tangerina* (Kinoshita et al. 2000) Labeda and Kroppenstedt 2007

The description is as before (Labeda and Kroppenstedt, 2007) with the following modification. The G+C content of the type-strain genome is 71.6%, its approximate size 10.68 Mbp, its IMG deposit 2728369483.

#### Emended description of *Varibaculum cambriense* Hall et al. 2003

The description is as before (Hall et al., 2003d) with the following modification. The G+C content of the type-strain genome is 53.4%, its approximate size 2.02 Mbp, its GenBank deposit SAMN02440916.

#### Emended description of *Williamsia deligens* Yassin and Hupfer 2006

The description is as before (Yassin and Hupfer, 2006) with the following addition. The G+C content of the type-strain genome is 70.2%, its approximate size 4.41 Mbp, its GenBank deposit SAMN05661029.

#### Emended description of *Williamsia maris* Stach et al. 2004

The description is as before (Stach et al., 2004) with the following addition. The G+C content of the type-strain genome is 68.2%, its approximate size 4.93 Mbp, its GenBank deposit SAMN05660695.

#### Emended description of *Williamsia serinedens* Yassin et al. 2007

The description is as before (Yassin et al., 2007b) with the following addition. The G+C content of the type-strain genome is 70.4%, its approximate size 4.64 Mbp, its GenBank deposit SAMN05660736.

#### Emended description of *Williamsia sterculiae* Fang et al. 2013

The description is as before (Fang et al., 2013) with the following modification. The G+C content of the type-strain genome is 67.2%, its approximate size 4.44 Mbp, its GenBank deposit SAMN05445060.

#### Emended description of *Xylanimicrobium pachnodae* (Cazemier et al. 2004) Stackebrandt and Schumann 2004

The description is as before (Stackebrandt and Schumann, 2004) with the following modification. The G+C content of the type-strain genome is 71.9%, its approximate size 4.00 Mbp, its GenBank deposit SAMD00047215.

#### Emended description of *Yaniella halotolerans* (Li et al. 2004) Li et al. 2008

The description is as before (Li et al., 2008b) with the following modification. The G+C content of the type-strain genome is 55.6%, its approximate size 2.78 Mbp, its GenBank deposit SAMN02440577.

#### Emended description of *Zimmermannella alba* Lin et al. 2004

The description is as before (Lin et al., 2004) with the following modification. The G+C content of the type-strain genome is 64.7%, its approximate size 2.14 Mbp, its GenBank deposit SAMD00046486.

## Author contributions

TW, NK, H-PK, and MaG collected the genome sequences. JM-K and MaG phylogenetically and statistically analyzed the data. MG-L and MaG collected and interpreted the G+C content and genome-size information. MG-L, IN, LC, and MiG collected the phenotypic information. IN, LC, MiG, MaG and RP interpreted the phenotypic information. MiG, MaG, IN, LC, MG-L, RP and JM-K wrote the manuscript. All authors read and approved the final manuscript.

### Conflict of interest statement

The authors declare that the research was conducted in the absence of any commercial or financial relationships that could be construed as a potential conflict of interest.
