# Supplementary material for: Genome-Based Taxonomic Classification of the Phylum Actinobacteria
Source: Front Microbiol. 2018 Aug 22;9:2007. doi: 10.3389/fmicb.2018.02007 (PMC6113628; doi:10.3389/fmicb.2018.02007)
Supplement: Supplementary file 2 [file Data_Sheet_2.pdf]

Genome-based Taxonomic Classification of the Phylum *Actinobacteria*

Supplementary File S2

List of Figures

1 Phylogenomic GBDP tree without collapsed branches 11
2 Backbone-constrained 16S rRNA gene ML and MP tree 35
3 Unconstrained 16S rRNA gene ML and MP tree 61
4 23S rRNA gene ML and MP tree 70
5 16S rRNA gene ML and MP tree given GBDP tree sampling 79
6 Comparison of branch support 79
7 *Atopobium* ML tree 80
8 *Atopobium* MP tree 81
9 *Intrasporangium* ML tree 82
10 *Intrasporangium* MP tree 83
11 *Sanguibacter* ML tree 84
12 *Sanguibacter* MP tree 85
13 *Solirubrobacter* ML tree 86
14 *Solirubrobacter* MP tree 86
15 *Gordonia* ML tree 87
16 *Gordonia* MP tree 88
17 *Rhodococcus* ML tree 89
18 *Rhodococcus* MP tree 90
19 *Corynebacterium* ML tree 91
20 *Corynebacterium* MP tree 92

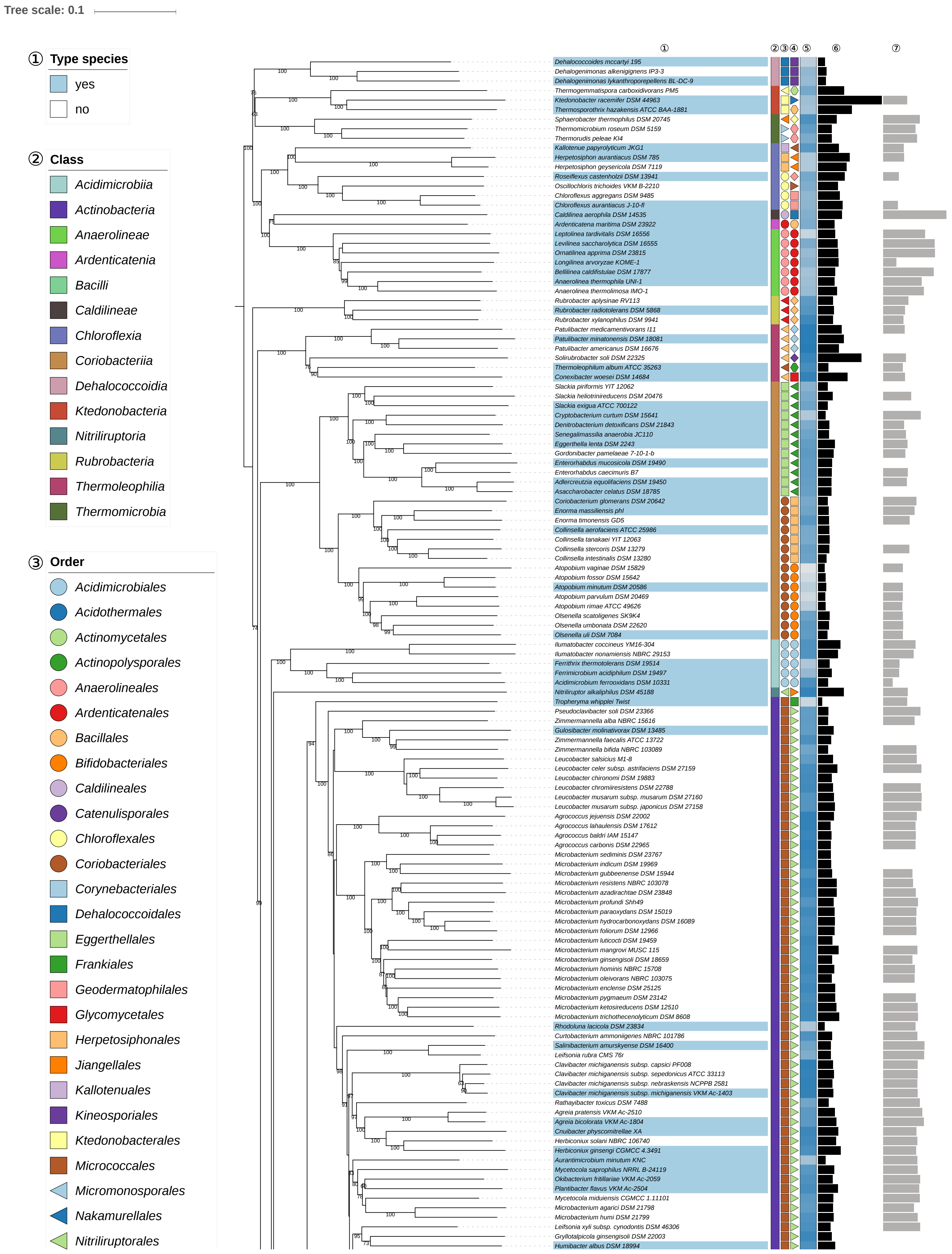

Tree scale: 0.1
① Type species
yes
no
② Class
Acidimicrobiia
Actinobacteria
Anaerolineae
Ardenticatenia
Bacilli
Caldilineae
Chloroflexia
Coriobacteriia
Dehalococcoidia
Ktedonobacteria
Nitriliruptoria
Rubrobacteria
Thermoleophilia
Thermomicrobia
③ Order
Acidimicrobiales
Acidothermales
Actinomycetales
Actinopolysporales
Anaerolineales
Ardenticatenales
Bacillales
Bifidobacteriales
Caldilineales
Catenulisporales
Chloroflexales
Coriobacteriales
Corynebacteriales
Dehalococcoidales
Eggerthellales
Frankiales
Geodermatophilales
Glycomycetales
Herpetosiphonales
Jiangellales
Kallotenuales
Kineosporiales
Ktedonobacterales
Micrococcales
Micromonosporales
Nakamurellales
Nitriliruptorales
①
②③④
⑤
⑥
⑦
Dehalococcoides mccartyi 195
Dehalogenimonas alkenigignens IP3-3
Dehalogenimonas lykanthroporepellens BL-DC-9
Thermogemmatispora carboxidivorans PM5
Ktedonobacter racemifer DSM 44963
Thermosporothrix hazakensis ATCC BAA-1881
Sphaerobacter thermophilus DSM 20745
Thermomicrobium roseum DSM 5159
Thermorudis peleae KI4
Kallotenue papyrolyticum JKG1
Herpetosiphon aurantiacus DSM 785
Herpetosiphon geysericola DSM 7119
Roseiflexus castenholzii DSM 13941
Oscillochloris trichoides VKM B-2210
Chloroflexus aggregans DSM 9485
Chloroflexus aurantiacus J-10-fl
Caldilinea aerophila DSM 14535
Ardenticatena maritima DSM 23922
Leptolinea tardivitalis DSM 16556
Levilinea saccharolytica DSM 16555
Ornatilinea apprima DSM 23815
Longilinea arvoryzae KOME-1
Bellilinea caldifistulae DSM 17877
Anaerolinea thermophila UNI-1
Anaerolinea thermolimosa IMO-1
Rubrobacter aplysinae RV113
Rubrobacter radiotolerans DSM 5868
Rubrobacter xylanophilus DSM 9941
Patulibacter medicamentivorans I11
Patulibacter minatonensis DSM 18081
Patulibacter americanus DSM 16676
Solirubrobacter soli DSM 22325
Thermoleophilum album ATCC 35263
Conexibacter woesei DSM 14684
Slackia piriformis YIT 12062
Slackia heliotrinireducens DSM 20476
Slackia exigua ATCC 700122
Cryptobacterium curtum DSM 15641
Denitrobacterium detoxificans DSM 21843
Senegalimassilia anaerobia JC110
Eggerthella lenta DSM 2243
Gordonibacter pamelaeae 7-10-1-b
Enterorhabdus mucosicola DSM 19490
Enterorhabdus caecimuris B7
Adlercreutzia equolifaciens DSM 19450
Asaccharobacter celatus DSM 18785
Coriobacterium glomerans DSM 20642
Enorma massiliensis phI
Enorma timonensis GD5
Collinsella aerofaciens ATCC 25986
Collinsella tanakaei YIT 12063
Collinsella stercoris DSM 13279
Collinsella intestinalis DSM 13280
Atopobium vaginae DSM 15829
Atopobium fossor DSM 15642
Atopobium minutum DSM 20586
Atopobium parvulum DSM 20469
Atopobium rimae ATCC 49626
Olsenella scatoligenes SK9K4
Olsenella umbonata DSM 22620
Olsenella uli DSM 7084
Ilumatobacter coccineus YM16-304
Ilumatobacter nonamiensis NBRC 29153
Ferrithrix thermotolerans DSM 19514
Ferrimicrobium acidiphilum DSM 19497
Acidimicrobium ferrooxidans DSM 10331
Nitriliruptor alkaliphilus DSM 45188
Tropheryma whipplei Twist
Pseudoclavibacter soli DSM 23366
Zimmermannella alba NBRC 15616
Gulosibacter molinativorax DSM 13485
Zimmermannella faecalis ATCC 13722
Zimmermannella bifida NBRC 103089
Leucobacter salsicius M1-8
Leucobacter celer subsp. astrifaciens DSM 27159
Leucobacter chironomi DSM 19883
Leucobacter chromiiresistens DSM 22788
Leucobacter musarum subsp. musarum DSM 27160
Leucobacter musarum subsp. japonicus DSM 27158
Agrococcus jejuensis DSM 22002
Agrococcus lahaulensis DSM 17612
Agrococcus baldri IAM 15147
Agrococcus carbonis DSM 22965
Microbacterium sediminis DSM 23767
Microbacterium indicum DSM 19969
Microbacterium gubbeenense DSM 15944
Microbacterium resistens NBRC 103078
Microbacterium azadirachtae DSM 23848
Microbacterium profundi Shh49
Microbacterium paraoxydans DSM 15019
Microbacterium hydrocarbonoxydans DSM 16089
Microbacterium foliorum DSM 12966
Microbacterium luticocti DSM 19459
Microbacterium mangrovi MUSC 115
Microbacterium ginsengisoli DSM 18659
Microbacterium hominis NBRC 15708
Microbacterium oleivorans NBRC 103075
Microbacterium enclense DSM 25125
Microbacterium pygmaeum DSM 23142
Microbacterium ketosireducens DSM 12510
Microbacterium trichothecenolyticum DSM 8608
Rhodoluna lacicola DSM 23834
Curtobacterium ammoniigenes NBRC 101786
Salinibacterium amurskyense DSM 16400
Leifsonia rubra CMS 76r
Clavibacter michiganensis subsp. capsici PF008
Clavibacter michiganensis subsp. sepedonicus ATCC 33113
Clavibacter michiganensis subsp. nebraskensis NCPPB 2581
Clavibacter michiganensis subsp. michiganensis VKM Ac-1403
Rathayibacter toxicus DSM 7488
Agreia pratensis VKM Ac-2510
Agreia bicolorata VKM Ac-1804
Cnuibacter physcomitrellae XA
Herbiconiux solani NBRC 106740
Herbiconiux ginsengi CGMCC 4.3491
Aurantimicrobium minutum KNC
Mycetocola saprophilus NRRL B-24119
Okibacterium fritillariae VKM Ac-2059
Plantibacter flavus VKM Ac-2504
Mycetocola miduiensis CGMCC 1.11101
Microbacterium agarici DSM 21798
Microbacterium humi DSM 21799
Leifsonia xyli subsp. cynodontis DSM 46306
Gryllotalpicola ginsengisoli DSM 22003
Humibacter albus DSM 18994

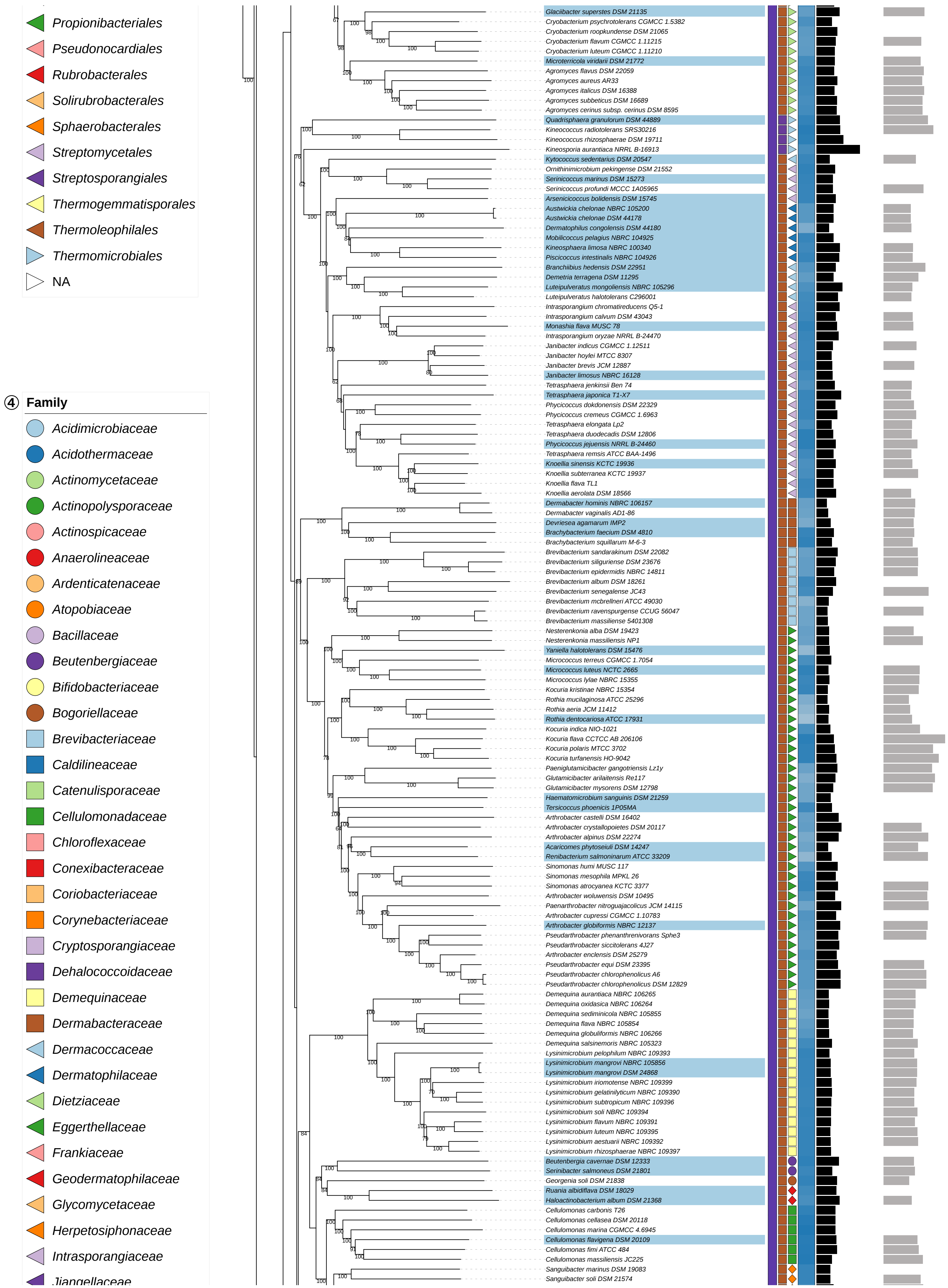

Propionibacteriales
Pseudonocardiales
Rubrobacterales
Solirubrobacterales
Sphaerobacterales
Streptomycetales
Streptosporangiales
Thermogemmatisporales
Thermoleophilales
Thermomicrobiales
NA
④ Family
Acidimicrobiaceae
Acidothermaceae
Actinomycetaceae
Actinopolysporaceae
Actinospicaceae
Anaerolineaceae
Ardenticatenaceae
Atopobiaceae
Bacillaceae
Beutenbergiaceae
Bifidobacteriaceae
Bogoriellaceae
Brevibacteriaceae
Caldilineaceae
Catenulisporaceae
Cellulomonadaceae
Chloroflexaceae
Conexibacteraceae
Coriobacteriaceae
Corynebacteriaceae
Cryptosporangiaceae
Dehalococcoidaceae
Demequinaceae
Dermabacteraceae
Dermacoccaceae
Dermatophilaceae
Dietziaceae
Eggerthellaceae
Frankiaceae
Geodermatophilaceae
Glycomycetaceae
Herpetosiphonaceae
Intrasporangiaceae
Jiangellaceae
Glaciibacter superstes DSM 21135
Cryobacterium psychrotolerans CGMCC 1.5382
Cryobacterium roopkundense DSM 21065
Cryobacterium flavum CGMCC 1.11215
Cryobacterium luteum CGMCC 1.11210
Microterricola viridarii DSM 21772
Agromyces flavus DSM 22059
Agromyces aureus AR33
Agromyces italicus DSM 16388
Agromyces subbeticus DSM 16689
Agromyces cerinus subsp. cerinus DSM 8595
Quadrisphaera granulorum DSM 44889
Kineococcus radiotolerans SRS30216
Kineococcus rhizosphaerae DSM 19711
Kineosporia aurantiaca NRRL B-16913
Kytococcus sedentarius DSM 20547
Ornithinimicrobium pekingense DSM 21552
Serinicoccus marinus DSM 15273
Serinicoccus profundi MCCC 1A05965
Arsenicicoccus bolidensis DSM 15745
Austwickia chelonae NBRC 105200
Austwickia chelonae DSM 44178
Dermatophilus congolensis DSM 44180
Mobilicoccus pelagius NBRC 104925
Kineosphaera limosa NBRC 100340
Piscicoccus intestinalis NBRC 104926
Branchiibius hedensis DSM 22951
Demetria terragena DSM 11295
Luteipulveratus mongoliensis NBRC 105296
Luteipulveratus halotolerans C296001
Intrasporangium chromatireducens Q5-1
Intrasporangium calvum DSM 43043
Monashia flava MUSC 78
Intrasporangium oryzae NRRL B-24470
Janibacter indicus CGMCC 1.12511
Janibacter hoylei MTCC 8307
Janibacter brevis JCM 12887
Janibacter limosus NBRC 16128
Tetrasphaera jenkinsii Ben 74
Tetrasphaera japonica T1-X7
Phycicoccus dokdonensis DSM 22329
Phycicoccus cremeus CGMCC 1.6963
Tetrasphaera elongata Lp2
Tetrasphaera duodecadis DSM 12806
Phycicoccus jejuensis NRRL B-24460
Tetrasphaera remsis ATCC BAA-1496
Knoellia sinensis KCTC 19936
Knoellia subterranea KCTC 19937
Knoellia flava TL1
Knoellia aerolata DSM 18566
Dermabacter hominis NBRC 106157
Dermabacter vaginalis AD1-86
Devriesea agamarum IMP2
Brachybacterium faecium DSM 4810
Brachybacterium squillarum M-6-3
Brevibacterium sandarakinum DSM 22082
Brevibacterium siliguriense DSM 23676
Brevibacterium epidermidis NBRC 14811
Brevibacterium album DSM 18261
Brevibacterium senegalense JC43
Brevibacterium mcbrellneri ATCC 49030
Brevibacterium ravenspurgense CCUG 56047
Brevibacterium massiliense 5401308
Nesterenkonia alba DSM 19423
Nesterenkonia massiliensis NP1
Yaniella halotolerans DSM 15476
Micrococcus terreus CGMCC 1.7054
Micrococcus luteus NCTC 2665
Micrococcus lylae NBRC 15355
Kocuria kristinae NBRC 15354
Rothia mucilaginosa ATCC 25296
Rothia aeria JCM 11412
Rothia dentocariosa ATCC 17931
Kocuria indica NIO-1021
Kocuria flava CCTCC AB 206106
Kocuria polaris MTCC 3702
Kocuria turfanensis HO-9042
Paeniglutamicibacter gangotriensis Lz1y
Glutamicibacter arilaitensis Re117
Glutamicibacter mysorens DSM 12798
Haematomicrobium sanguinis DSM 21259
Tersicoccus phoenicis 1P05MA
Arthrobacter castelli DSM 16402
Arthrobacter crystallopoietes DSM 20117
Arthrobacter alpinus DSM 22274
Acaricomes phytoseiuli DSM 14247
Renibacterium salmoninarum ATCC 33209
Sinomonas humi MUSC 117
Sinomonas mesophila MPKL 26
Sinomonas atrocyanea KCTC 3377
Arthrobacter woluwensis DSM 10495
Paenarthrobacter nitroguajacolicus JCM 14115
Arthrobacter cupressi CGMCC 1.10783
Arthrobacter globiformis NBRC 12137
Pseudarthrobacter phenanthrenivorans Sphe3
Pseudarthrobacter siccitolerans 4J27
Arthrobacter enclensis DSM 25279
Pseudarthrobacter equi DSM 23395
Pseudarthrobacter chlorophenolicus A6
Pseudarthrobacter chlorophenolicus DSM 12829
Demequina aurantiaca NBRC 106265
Demequina oxidasica NBRC 106264
Demequina sediminicola NBRC 105855
Demequina flava NBRC 105854
Demequina globuliformis NBRC 106266
Demequina salsinemoris NBRC 105323
Lysinimicrobium pelophilum NBRC 109393
Lysinimicrobium mangrovi NBRC 105856
Lysinimicrobium mangrovi DSM 24868
Lysinimicrobium iriomotense NBRC 109399
Lysinimicrobium gelatinilyticum NBRC 109390
Lysinimicrobium subtropicum NBRC 109396
Lysinimicrobium soli NBRC 109394
Lysinimicrobium flavum NBRC 109391
Lysinimicrobium luteum NBRC 109395
Lysinimicrobium aestuarii NBRC 109392
Lysinimicrobium rhizosphaerae NBRC 109397
Beutenbergia cavernae DSM 12333
Serinibacter salmoneus DSM 21801
Georgenia soli DSM 21838
Ruania albidiflava DSM 18029
Haloactinobacterium album DSM 21368
Cellulomonas carbonis T26
Cellulomonas cellasea DSM 20118
Cellulomonas marina CGMCC 4.6945
Cellulomonas flavigena DSM 20109
Cellulomonas fimi ATCC 484
Cellulomonas massiliensis JC225
Sanguibacter marinus DSM 19083
Sanguibacter soli DSM 21574

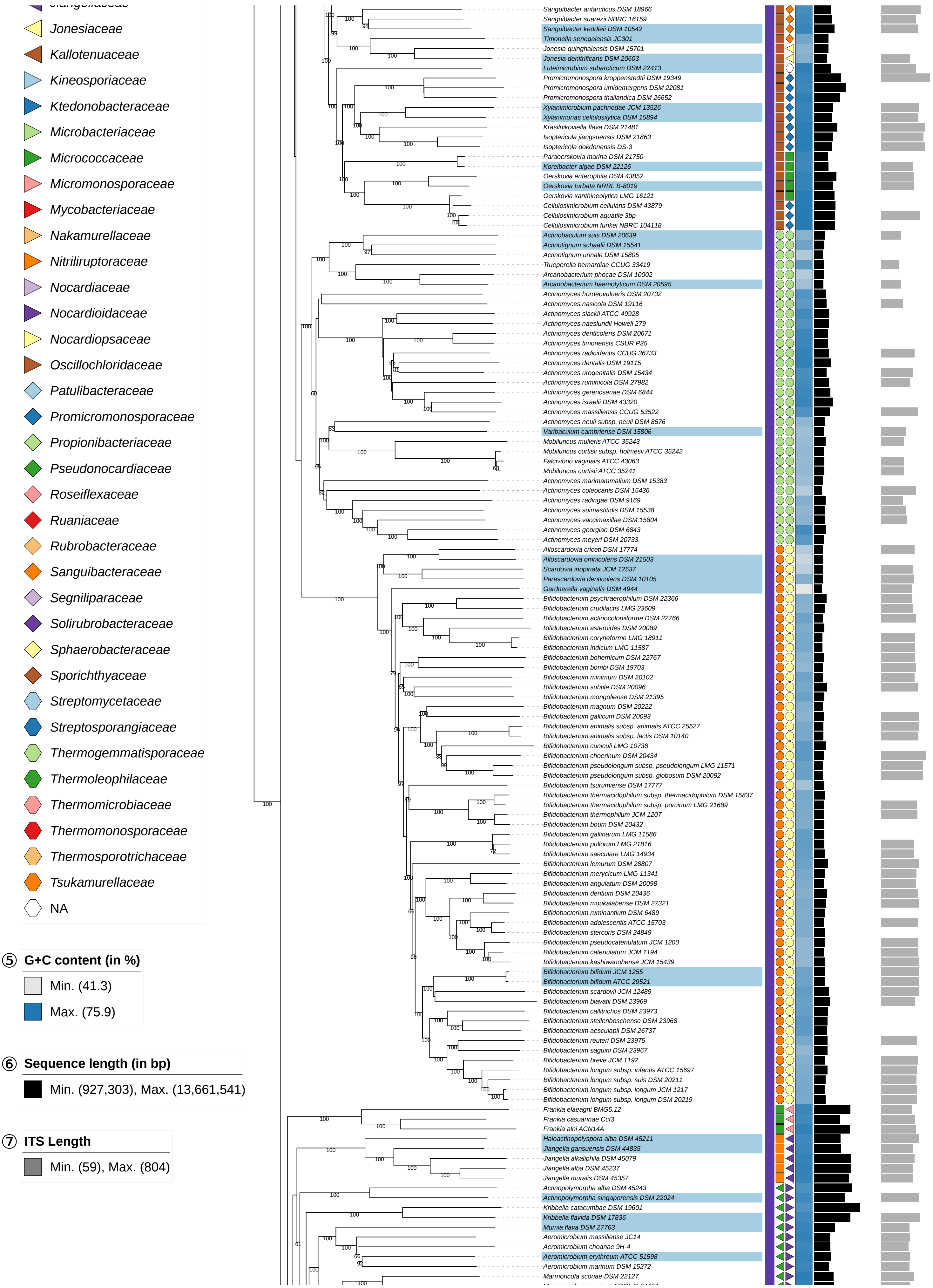
Jonesiaceae
Kallotenuaceae
Kineosporiaceae
Ktedonobacteraceae
Microbacteriaceae
Micrococcaceae
Micromonosporaceae
Mycobacteriaceae
Nakamurellaceae
Nitriliruptoraceae
Nocardiaceae
Nocardioidaceae
Nocardiopsaceae
Oscillochloridaceae
Patulibacteraceae
Promicromonosporaceae
Propionibacteriaceae
Pseudonocardiaceae
Roseiflexaceae
Ruaniaceae
Rubrobacteraceae
Sanguibacteraceae
Segniliparaceae
Solirubrobacteraceae
Sphaerobacteraceae
Sporichthyaceae
Streptomycetaceae
Streptosporangiaceae
Thermogemmatisporaceae
Thermoleophilaceae
Thermomicrobiaceae
Thermomonosporaceae
Thermosporotrichaceae
Tsukamurellaceae
NA
⑤ G+C content (in %)
Min. (41.3)
Max. (75.9)
⑥ Sequence length (in bp)
Min. (927,303), Max. (13,661,541)
⑦ ITS Length
Min. (59), Max. (804)
Sanguibacter antarcticus DSM 18966
Sanguibacter suarezii NBRC 16159
Sanguibacter keddieii DSM 10542
Timonella senegalensis JC301
Jonesia quinghaiensis DSM 15701
Jonesia denitrificans DSM 20603
Luteimicrobium subarcticum DSM 22413
Promicromonospora kroppenstedtii DSM 19349
Promicromonospora umidemergens DSM 22081
Promicromonospora thailandica DSM 26652
Xylanimicrobium pachnodae JCM 13526
Xylanimonas cellulosilytica DSM 15894
Krasilnikoviella flava DSM 21481
Isoptericola jiangsuensis DSM 21863
Isoptericola dokdonensis DS-3
Paraoerskovia marina DSM 21750
Koreibacter algae DSM 22126
Oerskovia enterophila DSM 43852
Oerskovia turbata NRRL B-8019
Oerskovia xanthineolytica LMG 16121
Cellulosimicrobium cellulans DSM 43879
Cellulosimicrobium aquatile 3bp
Cellulosimicrobium funkei NBRC 104118
Actinobaculum suis DSM 20639
Actinotignum schaalii DSM 15541
Actinotignum urinale DSM 15805
Trueperella bernardiae CCUG 33419
Arcanobacterium phocae DSM 10002
Arcanobacterium haemolyticum DSM 20595
Actinomyces hordeovulneris DSM 20732
Actinomyces nasicola DSM 19116
Actinomyces slackii ATCC 49928
Actinomyces naeslundii Howell 279
Actinomyces denticolens DSM 20671
Actinomyces timonensis CSUR P35
Actinomyces radicidentis CCUG 36733
Actinomyces dentalis DSM 19115
Actinomyces urogenitalis DSM 15434
Actinomyces ruminicola DSM 27982
Actinomyces gerencseriae DSM 6844
Actinomyces israelii DSM 43320
Actinomyces massiliensis CCUG 53522
Actinomyces neuii subsp. neuii DSM 8576
Varibaculum cambriense DSM 15806
Mobiluncus mulieris ATCC 35243
Mobiluncus curtisii subsp. holmesii ATCC 35242
Falcivibrio vaginalis ATCC 43063
Mobiluncus curtisii ATCC 35241
Actinomyces marimammalium DSM 15383
Actinomyces coleocanis DSM 15436
Actinomyces radingae DSM 9169
Actinomyces suimastitidis DSM 15538
Actinomyces vaccimaxillae DSM 15804
Actinomyces georgiae DSM 6843
Actinomyces meyeri DSM 20733
Alloscardovia criceti DSM 17774
Alloscardovia omnicolens DSM 21503
Scardovia inopinata JCM 12537
Parascardovia denticolens DSM 10105
Gardnerella vaginalis DSM 4944
Bifidobacterium psychraerophilum DSM 22366
Bifidobacterium crudilactis LMG 23609
Bifidobacterium actinocoloniiforme DSM 22766
Bifidobacterium asteroides DSM 20089
Bifidobacterium coryneforme LMG 18911
Bifidobacterium indicum LMG 11587
Bifidobacterium bohemicum DSM 22767
Bifidobacterium bombi DSM 19703
Bifidobacterium minimum DSM 20102
Bifidobacterium subtile DSM 20096
Bifidobacterium mongoliense DSM 21395
Bifidobacterium magnum DSM 20222
Bifidobacterium gallicum DSM 20093
Bifidobacterium animalis subsp. animalis ATCC 25527
Bifidobacterium animalis subsp. lactis DSM 10140
Bifidobacterium cuniculi LMG 10738
Bifidobacterium choerinum DSM 20434
Bifidobacterium pseudolongum subsp. pseudolongum LMG 11571
Bifidobacterium pseudolongum subsp. globosum DSM 20092
Bifidobacterium tsurumiense DSM 17777
Bifidobacterium thermacidophilum subsp. thermacidophilum DSM 15837
Bifidobacterium thermacidophilum subsp. porcinum LMG 21689
Bifidobacterium thermophilum JCM 1207
Bifidobacterium boum DSM 20432
Bifidobacterium gallinarum LMG 11586
Bifidobacterium pullorum LMG 21816
Bifidobacterium saeculare LMG 14934
Bifidobacterium lemurum DSM 28807
Bifidobacterium merycicum LMG 11341
Bifidobacterium angulatum DSM 20098
Bifidobacterium dentium DSM 20436
Bifidobacterium moukalabense DSM 27321
Bifidobacterium ruminantium DSM 6489
Bifidobacterium adolescentis ATCC 15703
Bifidobacterium stercoris DSM 24849
Bifidobacterium pseudocatenulatum JCM 1200
Bifidobacterium catenulatum JCM 1194
Bifidobacterium kashiwanohense JCM 15439
Bifidobacterium bifidum JCM 1255
Bifidobacterium bifidum ATCC 29521
Bifidobacterium scardovii JCM 12489
Bifidobacterium biavatii DSM 23969
Bifidobacterium callitrichos DSM 23973
Bifidobacterium stellenboschense DSM 23968
Bifidobacterium aesculapii DSM 26737
Bifidobacterium reuteri DSM 23975
Bifidobacterium saguini DSM 23967
Bifidobacterium breve JCM 1192
Bifidobacterium longum subsp. infantis ATCC 15697
Bifidobacterium longum subsp. suis DSM 20211
Bifidobacterium longum subsp. longum JCM 1217
Bifidobacterium longum subsp. longum DSM 20219
Frankia elaeagni BMG5.12
Frankia casuarinae CcI3
Frankia alni ACN14A
Haloactinopolyspora alba DSM 45211
Jiangella gansuensis DSM 44835
Jiangella alkaliphila DSM 45079
Jiangella alba DSM 45237
Jiangella muralis DSM 45357
Actinopolymorpha alba DSM 45243
Actinopolymorpha singaporensis DSM 22024
Kribbella catacumbae DSM 19601
Kribbella flavida DSM 17836
Mumia flava DSM 27763
Aeromicrobium massiliense JC14
Aeromicrobium choanae 9H-4
Aeromicrobium erythreum ATCC 51598
Aeromicrobium marinum DSM 15272
Marmoricola scoriae DSM 22127

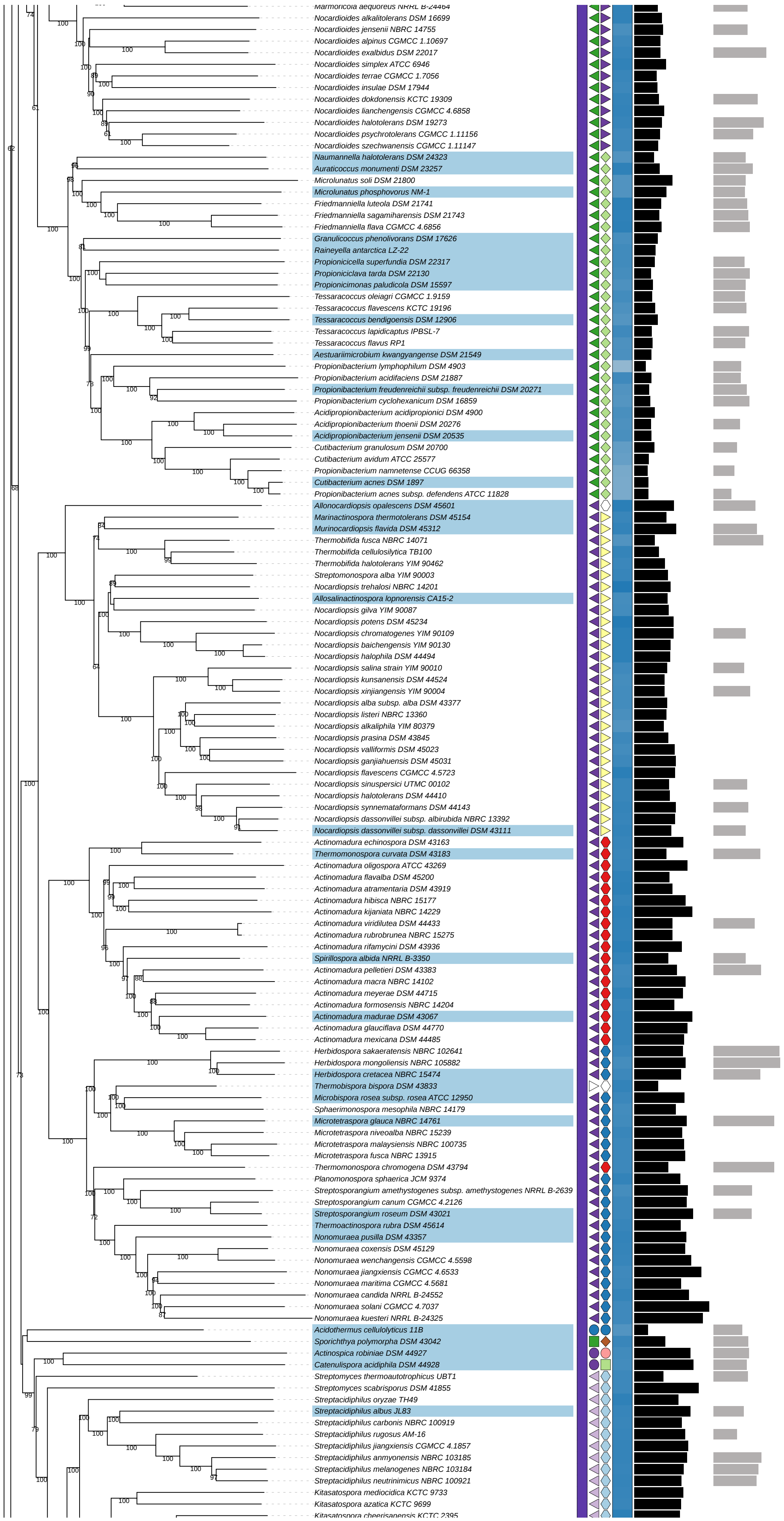
Nocardioides alkalitolerans DSM 16699
Nocardioides jensenii NBRC 14755
Nocardioides alpinus CGMCC 1.10697
Nocardioides exalbidus DSM 22017
Nocardioides simplex ATCC 6946
Nocardioides terrae CGMCC 1.7056
Nocardioides insulae DSM 17944
Nocardioides dokdonensis KCTC 19309
Nocardioides lianchengensis CGMCC 4.6858
Nocardioides halotolerans DSM 19273
Nocardioides psychrotolerans CGMCC 1.11156
Nocardioides szechwanensis CGMCC 1.11147
Naumannella halotolerans DSM 24323
Auraticoccus monumenti DSM 23257
Microlunatus soli DSM 21800
Microlunatus phosphovorus NM-1
Friedmanniella luteola DSM 21741
Friedmanniella sagamiharensis DSM 21743
Friedmanniella flava CGMCC 4.6856
Granulicoccus phenolivorans DSM 17626
Raineyella antarctica LZ-22
Propionicicella superfundia DSM 22317
Propioniciclava tarda DSM 22130
Propionicimonas paludicola DSM 15597
Tessaracoccus oleiagri CGMCC 1.9159
Tessaracoccus flavescens KCTC 19196
Tessaracoccus bendigoensis DSM 12906
Tessaracoccus lapidicaptus IPBSL-7
Tessaracoccus flavus RP1
Aestuariimicrobium kwangyangense DSM 21549
Propionibacterium lymphophilum DSM 4903
Propionibacterium acidifaciens DSM 21887
Propionibacterium freudenreichii subsp. freudenreichii DSM 20271
Propionibacterium cyclohexanicum DSM 16859
Acidipropionibacterium acidipropionici DSM 4900
Acidipropionibacterium thoenii DSM 20276
Acidipropionibacterium jensenii DSM 20535
Cutibacterium granulosum DSM 20700
Cutibacterium avidum ATCC 25577
Propionibacterium namnetense CCUG 66358
Cutibacterium acnes DSM 1897
Propionibacterium acnes subsp. defendens ATCC 11828
Allonocardiopsis opalescens DSM 45601
Marinactinospora thermotolerans DSM 45154
Murinocardiopsis flavida DSM 45312
Thermobifida fusca NBRC 14071
Thermobifida cellulosilytica TB100
Thermobifida halotolerans YIM 90462
Streptomonospora alba YIM 90003
Nocardiopsis trehalosi NBRC 14201
Allosalinactinospora lopnorensis CA15-2
Nocardiopsis gilva YIM 90087
Nocardiopsis potens DSM 45234
Nocardiopsis chromatogenes YIM 90109
Nocardiopsis baichengensis YIM 90130
Nocardiopsis halophila DSM 44494
Nocardiopsis salina strain YIM 90010
Nocardiopsis kunsanensis DSM 44524
Nocardiopsis xinjiangensis YIM 90004
Nocardiopsis alba subsp. alba DSM 43377
Nocardiopsis listeri NBRC 13360
Nocardiopsis alkaliphila YIM 80379
Nocardiopsis prasina DSM 43845
Nocardiopsis valliformis DSM 45023
Nocardiopsis ganjiahuensis DSM 45031
Nocardiopsis flavescens CGMCC 4.5723
Nocardiopsis sinuspersici UTMC 00102
Nocardiopsis halotolerans DSM 44410
Nocardiopsis synnemataformans DSM 44143
Nocardiopsis dassonvillei subsp. albirubida NBRC 13392
Nocardiopsis dassonvillei subsp. dassonvillei DSM 43111
Actinomadura echinospora DSM 43163
Thermomonospora curvata DSM 43183
Actinomadura oligospora ATCC 43269
Actinomadura flavalba DSM 45200
Actinomadura atramentaria DSM 43919
Actinomadura hibisca NBRC 15177
Actinomadura kijaniata NBRC 14229
Actinomadura viridilutea DSM 44433
Actinomadura rubrobrunea NBRC 15275
Actinomadura rifamycini DSM 43936
Spirillospora albida NRRL B-3350
Actinomadura pelletieri DSM 43383
Actinomadura macra NBRC 14102
Actinomadura meyerae DSM 44715
Actinomadura formosensis NBRC 14204
Actinomadura madurae DSM 43067
Actinomadura glauciflava DSM 44770
Actinomadura mexicana DSM 44485
Herbidospora sakaeratensis NBRC 102641
Herbidospora mongoliensis NBRC 105882
Herbidospora cretacea NBRC 15474
Thermobispora bispora DSM 43833
Microbispora rosea subsp. rosea ATCC 12950
Sphaerimonospora mesophila NBRC 14179
Microtetraspora glauca NBRC 14761
Microtetraspora niveoalba NBRC 15239
Microtetraspora malaysiensis NBRC 100735
Microtetraspora fusca NBRC 13915
Thermomonospora chromogena DSM 43794
Planomonospora sphaerica JCM 9374
Streptosporangium amethystogenes subsp. amethystogenes NRRL B-2639
Streptosporangium canum CGMCC 4.2126
Streptosporangium roseum DSM 43021
Thermoactinospora rubra DSM 45614
Nonomuraea pusilla DSM 43357
Nonomuraea coxensis DSM 45129
Nonomuraea wenchangensis CGMCC 4.5598
Nonomuraea jiangxiensis CGMCC 4.6533
Nonomuraea maritima CGMCC 4.5681
Nonomuraea candida NRRL B-24552
Nonomuraea solani CGMCC 4.7037
Nonomuraea kuesteri NRRL B-24325
Acidothermus cellulolyticus 11B
Sporichthya polymorpha DSM 43042
Actinospica robiniae DSM 44927
Catenulispora acidiphila DSM 44928
Streptomyces thermoautotrophicus UBT1
Streptomyces scabrisporus DSM 41855
Streptacidiphilus oryzae TH49
Streptacidiphilus albus JL83
Streptacidiphilus carbonis NBRC 100919
Streptacidiphilus rugosus AM-16
Streptacidiphilus jiangxiensis CGMCC 4.1857
Streptacidiphilus anmyonensis NBRC 103185
Streptacidiphilus melanogenes NBRC 103184
Streptacidiphilus neutrinimicus NBRC 100921
Kitasatospora mediocidica KCTC 9733
Kitasatospora azatica KCTC 9699

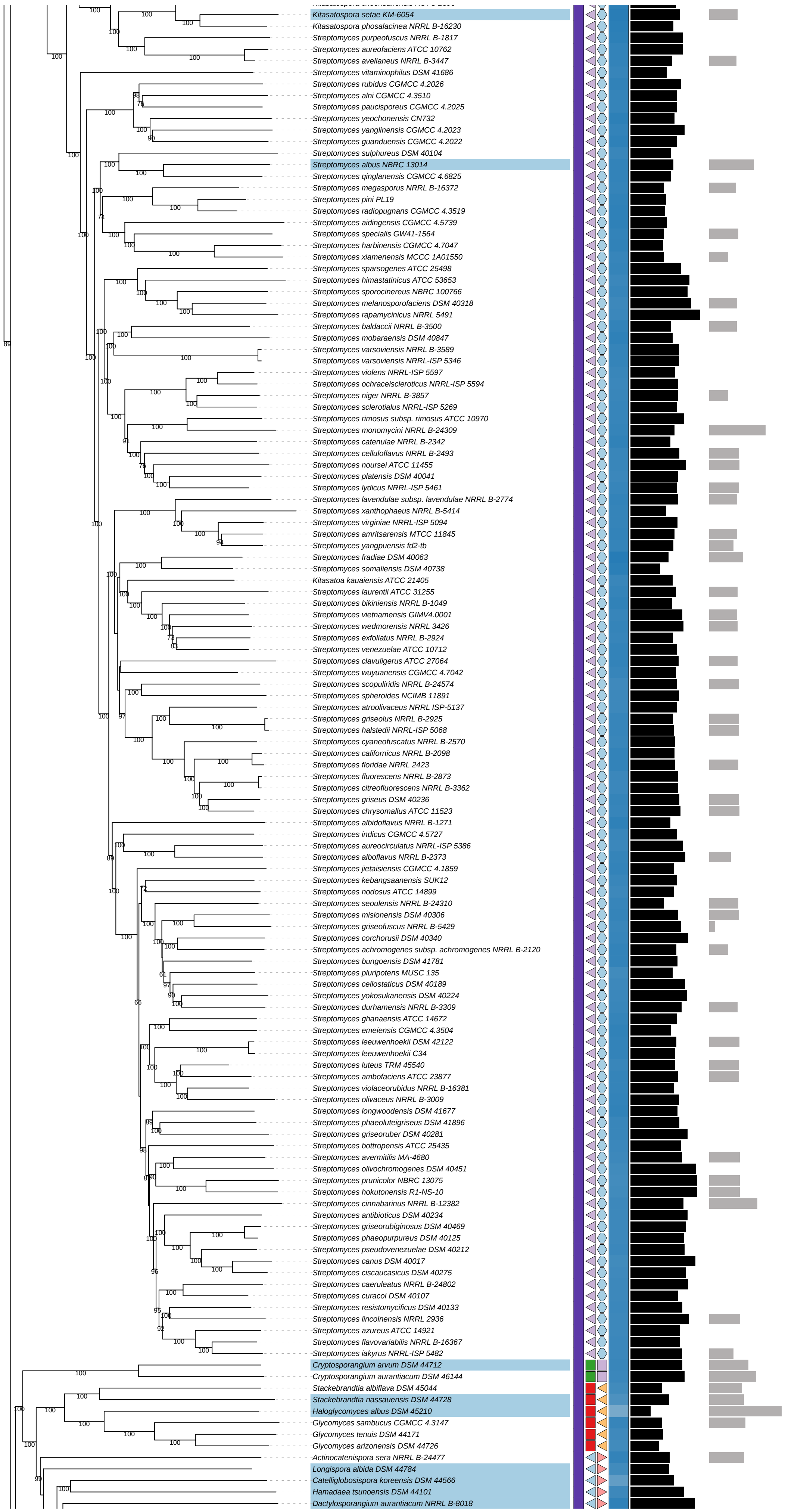
Kitasatospora setae KM-6054
Kitasatospora phosalacinea NRRL B-16230
Streptomyces purpeofuscus NRRL B-1817
Streptomyces aureofaciens ATCC 10762
Streptomyces avellaneus NRRL B-3447
Streptomyces vitaminophilus DSM 41686
Streptomyces rubidus CGMCC 4.2026
Streptomyces alni CGMCC 4.3510
Streptomyces paucisporeus CGMCC 4.2025
Streptomyces yeochonensis CN732
Streptomyces yanglinensis CGMCC 4.2023
Streptomyces guanduensis CGMCC 4.2022
Streptomyces sulphureus DSM 40104
Streptomyces albus NBRC 13014
Streptomyces qinglanensis CGMCC 4.6825
Streptomyces megasporus NRRL B-16372
Streptomyces pini PL19
Streptomyces radiopugnans CGMCC 4.3519
Streptomyces aidingensis CGMCC 4.5739
Streptomyces specialis GW41-1564
Streptomyces harbinensis CGMCC 4.7047
Streptomyces xiamenensis MCCC 1A01550
Streptomyces sparsogenes ATCC 25498
Streptomyces himastatinicus ATCC 53653
Streptomyces sporocinereus NBRC 100766
Streptomyces melanosporofaciens DSM 40318
Streptomyces rapamycinicus NRRL 5491
Streptomyces baldaccii NRRL B-3500
Streptomyces mobaraensis DSM 40847
Streptomyces varsoviensis NRRL B-3589
Streptomyces varsoviensis NRRL-ISP 5346
Streptomyces violens NRRL-ISP 5597
Streptomyces ochraceiscleroticus NRRL-ISP 5594
Streptomyces niger NRRL B-3857
Streptomyces sclerotialus NRRL-ISP 5269
Streptomyces rimosus subsp. rimosus ATCC 10970
Streptomyces monomycini NRRL B-24309
Streptomyces catenulae NRRL B-2342
Streptomyces celluloflavus NRRL B-2493
Streptomyces noursei ATCC 11455
Streptomyces platensis DSM 40041
Streptomyces lydicus NRRL-ISP 5461
Streptomyces lavendulae subsp. lavendulae NRRL B-2774
Streptomyces xanthophaeus NRRL B-5414
Streptomyces virginiae NRRL-ISP 5094
Streptomyces amritsarensis MTCC 11845
Streptomyces yangpuensis fd2-tb
Streptomyces fradiae DSM 40063
Streptomyces somaliensis DSM 40738
Kitasatoa kauaiensis ATCC 21405
Streptomyces laurentii ATCC 31255
Streptomyces bikiniensis NRRL B-1049
Streptomyces vietnamensis GIMV4.0001
Streptomyces wedmorensis NRRL 3426
Streptomyces exfoliatus NRRL B-2924
Streptomyces venezuelae ATCC 10712
Streptomyces clavuligerus ATCC 27064
Streptomyces wuyuanensis CGMCC 4.7042
Streptomyces scopuliridis NRRL B-24574
Streptomyces spheroides NCIMB 11891
Streptomyces atroolivaceus NRRL ISP-5137
Streptomyces griseolus NRRL B-2925
Streptomyces halstedii NRRL-ISP 5068
Streptomyces cyaneofuscatus NRRL B-2570
Streptomyces californicus NRRL B-2098
Streptomyces floridae NRRL 2423
Streptomyces fluorescens NRRL B-2873
Streptomyces citreofluorescens NRRL B-3362
Streptomyces griseus DSM 40236
Streptomyces chrysomallus ATCC 11523
Streptomyces albidoflavus NRRL B-1271
Streptomyces indicus CGMCC 4.5727
Streptomyces aureocirculatus NRRL-ISP 5386
Streptomyces alboflavus NRRL B-2373
Streptomyces jietaisiensis CGMCC 4.1859
Streptomyces kebangsaanensis SUK12
Streptomyces nodosus ATCC 14899
Streptomyces seoulensis NRRL B-24310
Streptomyces misionensis DSM 40306
Streptomyces griseofuscus NRRL B-5429
Streptomyces corchorusii DSM 40340
Streptomyces achromogenes subsp. achromogenes NRRL B-2120
Streptomyces bungoensis DSM 41781
Streptomyces pluripotens MUSC 135
Streptomyces cellostaticus DSM 40189
Streptomyces yokosukanensis DSM 40224
Streptomyces durhamensis NRRL B-3309
Streptomyces ghanaensis ATCC 14672
Streptomyces emeiensis CGMCC 4.3504
Streptomyces leeuwenhoekii DSM 42122
Streptomyces leeuwenhoekii C34
Streptomyces luteus TRM 45540
Streptomyces ambofaciens ATCC 23877
Streptomyces violaceorubidus NRRL B-16381
Streptomyces olivaceus NRRL B-3009
Streptomyces longwoodensis DSM 41677
Streptomyces phaeoluteigriseus DSM 41896
Streptomyces griseoruber DSM 40281
Streptomyces bottropensis ATCC 25435
Streptomyces avermitilis MA-4680
Streptomyces olivochromogenes DSM 40451
Streptomyces prunicolor NBRC 13075
Streptomyces hokutonensis R1-NS-10
Streptomyces cinnabarinus NRRL B-12382
Streptomyces antibioticus DSM 40234
Streptomyces griseorubiginosus DSM 40469
Streptomyces phaeopurpureus DSM 40125
Streptomyces pseudovenezuelae DSM 40212
Streptomyces canus DSM 40017
Streptomyces ciscaucasicus DSM 40275
Streptomyces caeruleatus NRRL B-24802
Streptomyces curacoi DSM 40107
Streptomyces resistomycificus DSM 40133
Streptomyces lincolnensis NRRL 2936
Streptomyces azureus ATCC 14921
Streptomyces flavovariabilis NRRL B-16367
Streptomyces iakyrus NRRL-ISP 5482
Cryptosporangium arvum DSM 44712
Cryptosporangium aurantiacum DSM 46144
Stackebrandtia albiflava DSM 45044
Stackebrandtia nassauensis DSM 44728
Haloglycomyces albus DSM 45210
Glycomyces sambucus CGMCC 4.3147
Glycomyces tenuis DSM 44171
Glycomyces arizonensis DSM 44726
Actinocatenispora sera NRRL B-24477
Longispora albida DSM 44784
Catelliglobosispora koreensis DSM 44566
Hamadaea tsunoensis DSM 44101
Dactylosporangium aurantiacum NRRL B-8018

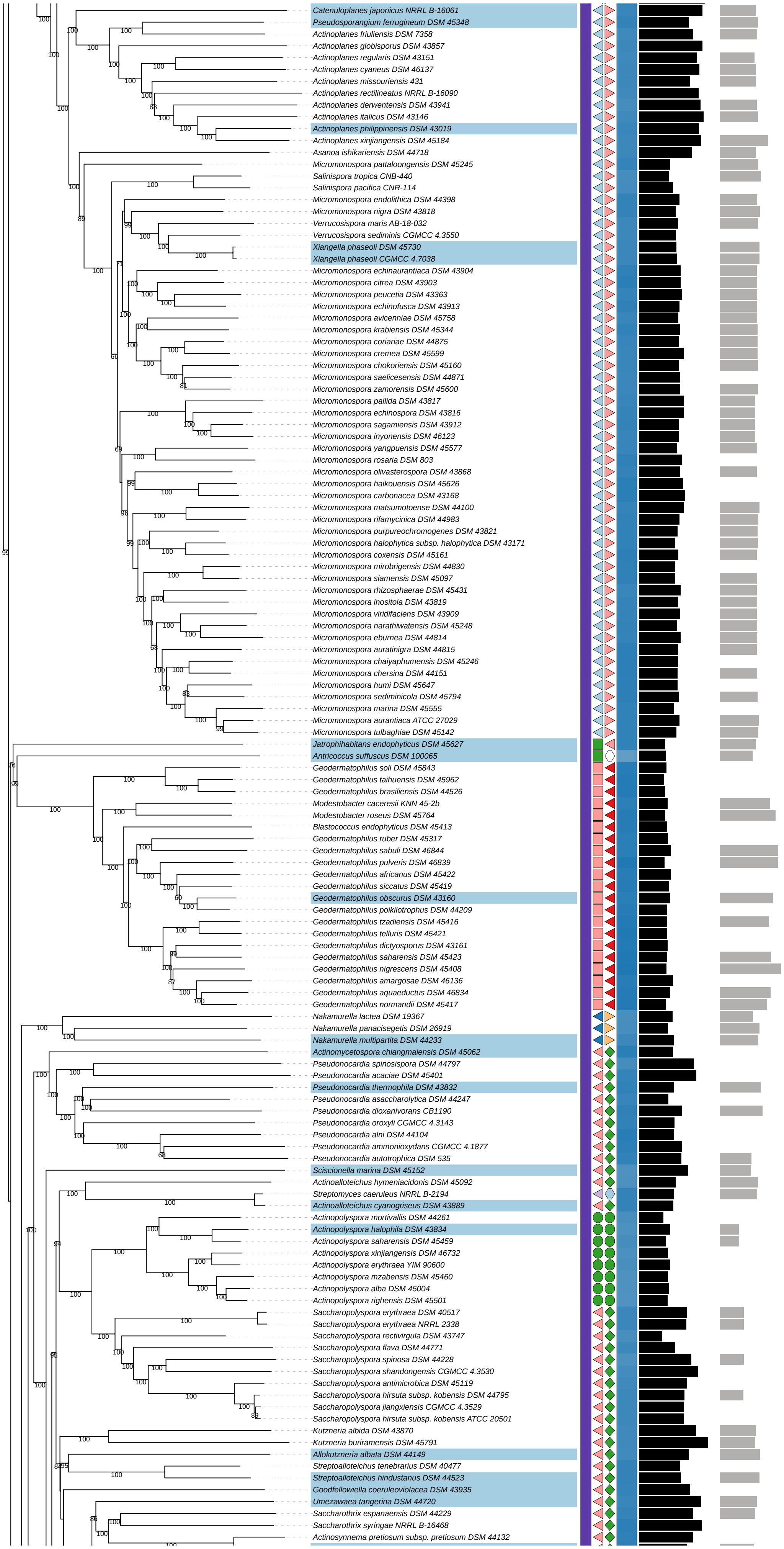
Catenuloplanes japonicus NRRL B-16061
Pseudosporangium ferrugineum DSM 45348
Actinoplanes friuliensis DSM 7358
Actinoplanes globisporus DSM 43857
Actinoplanes regularis DSM 43151
Actinoplanes cyaneus DSM 46137
Actinoplanes missouriensis 431
Actinoplanes rectilineatus NRRL B-16090
Actinoplanes derwentensis DSM 43941
Actinoplanes italicus DSM 43146
Actinoplanes philippinensis DSM 43019
Actinoplanes xinjiangensis DSM 45184
Asanoa ishikariensis DSM 44718
Micromonospora pattaloongensis DSM 45245
Salinispora tropica CNB-440
Salinispora pacifica CNR-114
Micromonospora endolithica DSM 44398
Micromonospora nigra DSM 43818
Verrucosispora maris AB-18-032
Verrucosispora sediminis CGMCC 4.3550
Xiangella phaseoli DSM 45730
Xiangella phaseoli CGMCC 4.7038
Micromonospora echinaurantiaca DSM 43904
Micromonospora citrea DSM 43903
Micromonospora peucetia DSM 43363
Micromonospora echinofusca DSM 43913
Micromonospora avicenniae DSM 45758
Micromonospora krabiensis DSM 45344
Micromonospora coriariae DSM 44875
Micromonospora cremea DSM 45599
Micromonospora chokoriensis DSM 45160
Micromonospora saelicesensis DSM 44871
Micromonospora zamorensis DSM 45600
Micromonospora pallida DSM 43817
Micromonospora echinospora DSM 43816
Micromonospora sagamiensis DSM 43912
Micromonospora inyonensis DSM 46123
Micromonospora yangpuensis DSM 45577
Micromonospora rosaria DSM 803
Micromonospora olivasterospora DSM 43868
Micromonospora haikouensis DSM 45626
Micromonospora carbonacea DSM 43168
Micromonospora matsumotoense DSM 44100
Micromonospora rifamycinica DSM 44983
Micromonospora purpureochromogenes DSM 43821
Micromonospora halophytica subsp. halophytica DSM 43171
Micromonospora coxensis DSM 45161
Micromonospora mirobrigensis DSM 44830
Micromonospora siamensis DSM 45097
Micromonospora rhizosphaerae DSM 45431
Micromonospora inositola DSM 43819
Micromonospora viridifaciens DSM 43909
Micromonospora narathiwatensis DSM 45248
Micromonospora eburnea DSM 44814
Micromonospora auratinigra DSM 44815
Micromonospora chaiyaphumensis DSM 45246
Micromonospora chersina DSM 44151
Micromonospora humi DSM 45647
Micromonospora sediminicola DSM 45794
Micromonospora marina DSM 45555
Micromonospora aurantiaca ATCC 27029
Micromonospora tulbaghiae DSM 45142
Jatrophihabitans endophyticus DSM 45627
Antricoccus suffuscus DSM 100065
Geodermatophilus soli DSM 45843
Geodermatophilus taihuensis DSM 45962
Geodermatophilus brasiliensis DSM 44526
Modestobacter caceresii KNN 45-2b
Modestobacter roseus DSM 45764
Blastococcus endophyticus DSM 45413
Geodermatophilus ruber DSM 45317
Geodermatophilus sabuli DSM 46844
Geodermatophilus pulveris DSM 46839
Geodermatophilus africanus DSM 45422
Geodermatophilus siccatus DSM 45419
Geodermatophilus obscurus DSM 43160
Geodermatophilus poikilotrophus DSM 44209
Geodermatophilus tzadiensis DSM 45416
Geodermatophilus telluris DSM 45421
Geodermatophilus dictyosporus DSM 43161
Geodermatophilus saharensis DSM 45423
Geodermatophilus nigrescens DSM 45408
Geodermatophilus amargosae DSM 46136
Geodermatophilus aquaeductus DSM 46834
Geodermatophilus normandii DSM 45417
Nakamurella lactea DSM 19367
Nakamurella panacisegetis DSM 26919
Nakamurella multipartita DSM 44233
Actinomycetospora chiangmaiensis DSM 45062
Pseudonocardia spinosispora DSM 44797
Pseudonocardia acaciae DSM 45401
Pseudonocardia thermophila DSM 43832
Pseudonocardia asaccharolytica DSM 44247
Pseudonocardia dioxanivorans CB1190
Pseudonocardia oroxyli CGMCC 4.3143
Pseudonocardia alni DSM 44104
Pseudonocardia ammonioxydans CGMCC 4.1877
Pseudonocardia autotrophica DSM 535
Sciscionella marina DSM 45152
Actinoalloteichus hymeniacidonis DSM 45092
Streptomyces caeruleus NRRL B-2194
Actinoalloteichus cyanogriseus DSM 43889
Actinopolyspora mortivallis DSM 44261
Actinopolyspora halophila DSM 43834
Actinopolyspora saharensis DSM 45459
Actinopolyspora xinjiangensis DSM 46732
Actinopolyspora erythraea YIM 90600
Actinopolyspora mzabensis DSM 45460
Actinopolyspora alba DSM 45004
Actinopolyspora righensis DSM 45501
Saccharopolyspora erythraea DSM 40517
Saccharopolyspora erythraea NRRL 2338
Saccharopolyspora rectivirgula DSM 43747
Saccharopolyspora flava DSM 44771
Saccharopolyspora spinosa DSM 44228
Saccharopolyspora shandongensis CGMCC 4.3530
Saccharopolyspora antimicrobica DSM 45119
Saccharopolyspora hirsuta subsp. kobensis DSM 44795
Saccharopolyspora jiangxiensis CGMCC 4.3529
Saccharopolyspora hirsuta subsp. kobensis ATCC 20501
Kutzneria albida DSM 43870
Kutzneria buriramensis DSM 45791
Allokutzneria albata DSM 44149
Streptoalloteichus tenebrarius DSM 40477
Streptoalloteichus hindustanus DSM 44523
Goodfellowiella coeruleoviolacea DSM 43935
Umezawaea tangerina DSM 44720
Saccharothrix espanaensis DSM 44229
Saccharothrix syringae NRRL B-16468
Actinosynnema pretiosum subsp. pretiosum DSM 44132

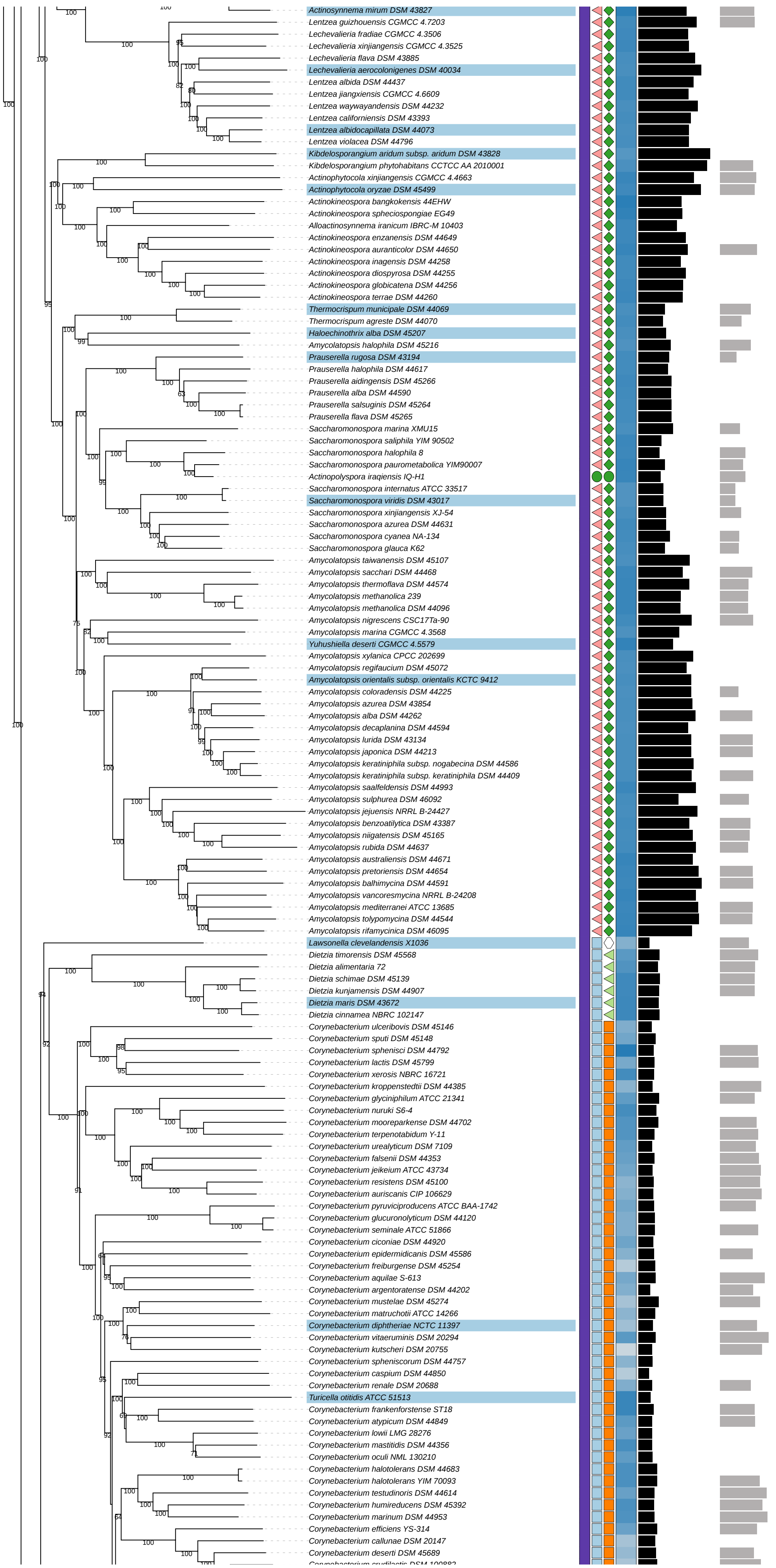
Actinosynnema mirum DSM 43827
Lentzea guizhouensis CGMCC 4.7203
Lechevalieria fradiae CGMCC 4.3506
Lechevalieria xinjiangensis CGMCC 4.3525
Lechevalieria flava DSM 43885
Lechevalieria aerocolonigenes DSM 40034
Lentzea albida DSM 44437
Lentzea jiangxiensis CGMCC 4.6609
Lentzea waywayandensis DSM 44232
Lentzea californiensis DSM 43393
Lentzea albidocapillata DSM 44073
Lentzea violacea DSM 44796
Kibdelosporangium aridum subsp. aridum DSM 43828
Kibdelosporangium phytohabitans CCTCC AA 2010001
Actinophytocola xinjiangensis CGMCC 4.4663
Actinophytocola oryzae DSM 45499
Actinokineospora bangkokensis 44EHW
Actinokineospora spheciospongiae EG49
Alloactinosynnema iranicum IBRC-M 10403
Actinokineospora enzanensis DSM 44649
Actinokineospora auranticolor DSM 44650
Actinokineospora inagensis DSM 44258
Actinokineospora diospyrosa DSM 44255
Actinokineospora globicatena DSM 44256
Actinokineospora terrae DSM 44260
Thermocrispum municipale DSM 44069
Thermocrispum agreste DSM 44070
Haloechinothrix alba DSM 45207
Amycolatopsis halophila DSM 45216
Prauserella rugosa DSM 43194
Prauserella halophila DSM 44617
Prauserella aidingensis DSM 45266
Prauserella alba DSM 44590
Prauserella salsuginis DSM 45264
Prauserella flava DSM 45265
Saccharomonospora marina XMU15
Saccharomonospora saliphila YIM 90502
Saccharomonospora halophila 8
Saccharomonospora paurometabolica YIM90007
Actinopolyspora iraqiensis IQ-H1
Saccharomonospora internatus ATCC 33517
Saccharomonospora viridis DSM 43017
Saccharomonospora xinjiangensis XJ-54
Saccharomonospora azurea DSM 44631
Saccharomonospora cyanea NA-134
Saccharomonospora glauca K62
Amycolatopsis taiwanensis DSM 45107
Amycolatopsis sacchari DSM 44468
Amycolatopsis thermoflava DSM 44574
Amycolatopsis methanolica 239
Amycolatopsis methanolica DSM 44096
Amycolatopsis nigrescens CSC17Ta-90
Amycolatopsis marina CGMCC 4.3568
Yuhushiella deserti CGMCC 4.5579
Amycolatopsis xylanica CPCC 202699
Amycolatopsis regifaucium DSM 45072
Amycolatopsis orientalis subsp. orientalis KCTC 9412
Amycolatopsis coloradensis DSM 44225
Amycolatopsis azurea DSM 43854
Amycolatopsis alba DSM 44262
Amycolatopsis decaplanina DSM 44594
Amycolatopsis lurida DSM 43134
Amycolatopsis japonica DSM 44213
Amycolatopsis keratiniphila subsp. nogabecina DSM 44586
Amycolatopsis keratiniphila subsp. keratiniphila DSM 44409
Amycolatopsis saalfeldensis DSM 44993
Amycolatopsis sulphurea DSM 46092
Amycolatopsis jejuensis NRRL B-24427
Amycolatopsis benzoatilytica DSM 43387
Amycolatopsis niigatensis DSM 45165
Amycolatopsis rubida DSM 44637
Amycolatopsis australiensis DSM 44671
Amycolatopsis pretoriensis DSM 44654
Amycolatopsis balhimycina DSM 44591
Amycolatopsis vancoresmycina NRRL B-24208
Amycolatopsis mediterranei ATCC 13685
Amycolatopsis tolypomycina DSM 44544
Amycolatopsis rifamycinica DSM 46095
Lawsonella clevelandensis X1036
Dietzia timorensis DSM 45568
Dietzia alimentaria 72
Dietzia schimae DSM 45139
Dietzia kunjamensis DSM 44907
Dietzia maris DSM 43672
Dietzia cinnamea NBRC 102147
Corynebacterium ulceribovis DSM 45146
Corynebacterium sputi DSM 45148
Corynebacterium sphenisci DSM 44792
Corynebacterium lactis DSM 45799
Corynebacterium xerosis NBRC 16721
Corynebacterium kroppenstedtii DSM 44385
Corynebacterium glyciniphilum ATCC 21341
Corynebacterium nuruki S6-4
Corynebacterium mooreparkense DSM 44702
Corynebacterium terpenotabidum Y-11
Corynebacterium urealyticum DSM 7109
Corynebacterium falsenii DSM 44353
Corynebacterium jeikeium ATCC 43734
Corynebacterium resistens DSM 45100
Corynebacterium auriscanis CIP 106629
Corynebacterium pyruviciproducens ATCC BAA-1742
Corynebacterium glucuronolyticum DSM 44120
Corynebacterium seminale ATCC 51866
Corynebacterium ciconiae DSM 44920
Corynebacterium epidermidicanis DSM 45586
Corynebacterium freiburgense DSM 45254
Corynebacterium aquilae S-613
Corynebacterium argentoratense DSM 44202
Corynebacterium mustelae DSM 45274
Corynebacterium matruchotii ATCC 14266
Corynebacterium diphtheriae NCTC 11397
Corynebacterium vitaeruminis DSM 20294
Corynebacterium kutscheri DSM 20755
Corynebacterium spheniscorum DSM 44757
Corynebacterium caspium DSM 44850
Corynebacterium renale DSM 20688
Turicella otitidis ATCC 51513
Corynebacterium frankenforstense ST18
Corynebacterium atypicum DSM 44849
Corynebacterium lowii LMG 28276
Corynebacterium mastitidis DSM 44356
Corynebacterium oculi NML 130210
Corynebacterium halotolerans DSM 44683
Corynebacterium halotolerans YIM 70093
Corynebacterium testudinoris DSM 44614
Corynebacterium humireducens DSM 45392
Corynebacterium marinum DSM 44953
Corynebacterium efficiens YS-314
Corynebacterium callunae DSM 20147
Corynebacterium deserti DSM 45689

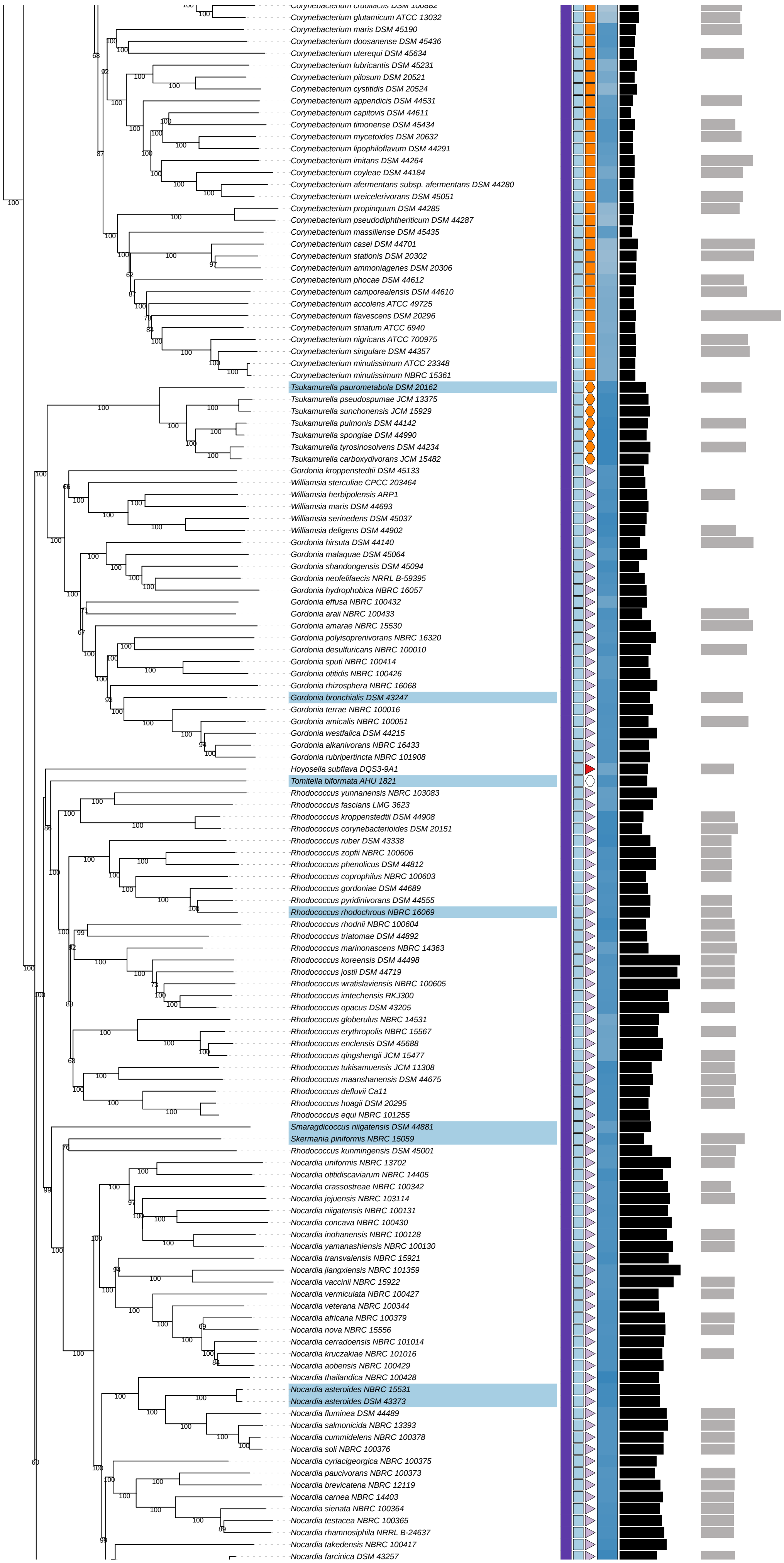

Corynebacterium glutamicum ATCC 13032
Corynebacterium maris DSM 45190
Corynebacterium doosanense DSM 45436
Corynebacterium uterequi DSM 45634
Corynebacterium lubricantis DSM 45231
Corynebacterium pilosum DSM 20521
Corynebacterium cystitidis DSM 20524
Corynebacterium appendicis DSM 44531
Corynebacterium capitovis DSM 44611
Corynebacterium timonense DSM 45434
Corynebacterium mycetoides DSM 20632
Corynebacterium lipophiloflavum DSM 44291
Corynebacterium imitans DSM 44264
Corynebacterium coyleae DSM 44184
Corynebacterium afermentans subsp. afermentans DSM 44280
Corynebacterium ureicelerivorans DSM 45051
Corynebacterium propinquum DSM 44285
Corynebacterium pseudodiphtheriticum DSM 44287
Corynebacterium massiliense DSM 45435
Corynebacterium casei DSM 44701
Corynebacterium stationis DSM 20302
Corynebacterium ammoniagenes DSM 20306
Corynebacterium phocae DSM 44612
Corynebacterium camporealensis DSM 44610
Corynebacterium accolens ATCC 49725
Corynebacterium flavescens DSM 20296
Corynebacterium striatum ATCC 6940
Corynebacterium nigricans ATCC 700975
Corynebacterium singulare DSM 44357
Corynebacterium minutissimum ATCC 23348
Corynebacterium minutissimum NBRC 15361
Tsukamurella paurometabola DSM 20162
Tsukamurella pseudospumae JCM 13375
Tsukamurella sunchonensis JCM 15929
Tsukamurella pulmonis DSM 44142
Tsukamurella spongiae DSM 44990
Tsukamurella tyrosinosolvens DSM 44234
Tsukamurella carboxydivorans JCM 15482
Gordonia kroppenstedtii DSM 45133
Williamsia sterculiae CPCC 203464
Williamsia herbipolensis ARP1
Williamsia maris DSM 44693
Williamsia serinedens DSM 45037
Williamsia deligens DSM 44902
Gordonia hirsuta DSM 44140
Gordonia malaquae DSM 45064
Gordonia shandongensis DSM 45094
Gordonia neofelifaecis NRRL B-59395
Gordonia hydrophobica NBRC 16057
Gordonia effusa NBRC 100432
Gordonia araii NBRC 100433
Gordonia amarae NBRC 15530
Gordonia polyisoprenivorans NBRC 16320
Gordonia desulfuricans NBRC 100010
Gordonia sputi NBRC 100414
Gordonia otitidis NBRC 100426
Gordonia rhizosphera NBRC 16068
Gordonia bronchialis DSM 43247
Gordonia terrae NBRC 100016
Gordonia amicalis NBRC 100051
Gordonia westfalica DSM 44215
Gordonia alkanivorans NBRC 16433
Gordonia rubripertincta NBRC 101908
Hoyosella subflava DQS3-9A1
Tomitella biformata AHU 1821
Rhodococcus yunnanensis NBRC 103083
Rhodococcus fascians LMG 3623
Rhodococcus kroppenstedtii DSM 44908
Rhodococcus corynebacterioides DSM 20151
Rhodococcus ruber DSM 43338
Rhodococcus zopfii NBRC 100606
Rhodococcus phenolicus DSM 44812
Rhodococcus coprophilus NBRC 100603
Rhodococcus gordoniae DSM 44689
Rhodococcus pyridinivorans DSM 44555
Rhodococcus rhodochrous NBRC 16069
Rhodococcus rhodnii NBRC 100604
Rhodococcus triatomae DSM 44892
Rhodococcus marinonascens NBRC 14363
Rhodococcus koreensis DSM 44498
Rhodococcus jostii DSM 44719
Rhodococcus wratislaviensis NBRC 100605
Rhodococcus imtechensis RKJ300
Rhodococcus opacus DSM 43205
Rhodococcus globerulus NBRC 14531
Rhodococcus erythropolis NBRC 15567
Rhodococcus enclensis DSM 45688
Rhodococcus qingshengii JCM 15477
Rhodococcus tukisamuensis JCM 11308
Rhodococcus maanshanensis DSM 44675
Rhodococcus defluvii Ca11
Rhodococcus hoagii DSM 20295
Rhodococcus equi NBRC 101255
Smaragdicoccus niigatensis DSM 44881
Skermania piniformis NBRC 15059
Rhodococcus kunmingensis DSM 45001
Nocardia uniformis NBRC 13702
Nocardia otitidiscaviarum NBRC 14405
Nocardia crassostreae NBRC 100342
Nocardia jejuensis NBRC 103114
Nocardia niigatensis NBRC 100131
Nocardia concava NBRC 100430
Nocardia inohanensis NBRC 100128
Nocardia yamanashiensis NBRC 100130
Nocardia transvalensis NBRC 15921
Nocardia jiangxiensis NBRC 101359
Nocardia vaccinii NBRC 15922
Nocardia vermiculata NBRC 100427
Nocardia veterana NBRC 100344
Nocardia africana NBRC 100379
Nocardia nova NBRC 15556
Nocardia cerradoensis NBRC 101014
Nocardia kruczakiae NBRC 101016
Nocardia aobensis NBRC 100429
Nocardia thailandica NBRC 100428
Nocardia asteroides NBRC 15531
Nocardia asteroides DSM 43373
Nocardia fluminea DSM 44489
Nocardia salmonicida NBRC 13393
Nocardia cummidelens NBRC 100378
Nocardia soli NBRC 100376
Nocardia cyriacigeorgica NBRC 100375
Nocardia paucivorans NBRC 100373
Nocardia brevicatena NBRC 12119
Nocardia carnea NBRC 14403
Nocardia sienata NBRC 100364
Nocardia testacea NBRC 100365
Nocardia rhamnosiphila NRRL B-24637
Nocardia takedensis NBRC 100417
Nocardia farcinica DSM 43257

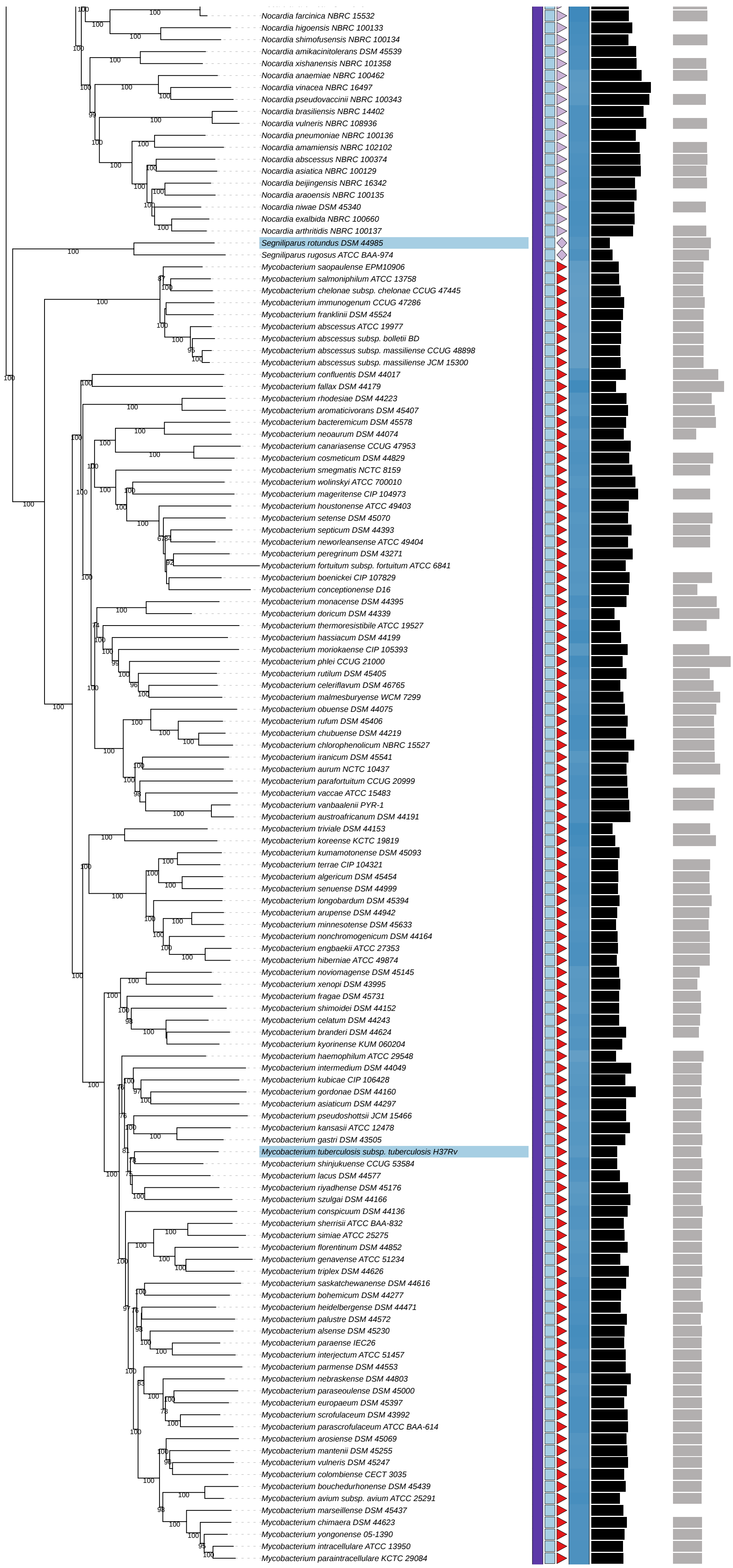

Nocardia farcinica NBRC 15532
Nocardia higoensis NBRC 100133
Nocardia shimofusensis NBRC 100134
Nocardia amikacinitolerans DSM 45539
Nocardia xishanensis NBRC 101358
Nocardia anaemiae NBRC 100462
Nocardia vinacea NBRC 16497
Nocardia pseudovaccinii NBRC 100343
Nocardia brasiliensis NBRC 14402
Nocardia vulneris NBRC 108936
Nocardia pneumoniae NBRC 100136
Nocardia amamiensis NBRC 102102
Nocardia abscessus NBRC 100374
Nocardia asiatica NBRC 100129
Nocardia beijingensis NBRC 16342
Nocardia araoensis NBRC 100135
Nocardia niwae DSM 45340
Nocardia exalbida NBRC 100660
Nocardia arthritidis NBRC 100137
Segniliparus rotundus DSM 44985
Segniliparus rugosus ATCC BAA-974
Mycobacterium saopaulense EPM10906
Mycobacterium salmoniphilum ATCC 13758
Mycobacterium chelonae subsp. chelonae CCUG 47445
Mycobacterium immunogenum CCUG 47286
Mycobacterium franklinii DSM 45524
Mycobacterium abscessus ATCC 19977
Mycobacterium abscessus subsp. bolletii BD
Mycobacterium abscessus subsp. massiliense CCUG 48898
Mycobacterium abscessus subsp. massiliense JCM 15300
Mycobacterium confluentis DSM 44017
Mycobacterium fallax DSM 44179
Mycobacterium rhodesiae DSM 44223
Mycobacterium aromaticivorans DSM 45407
Mycobacterium bacteremicum DSM 45578
Mycobacterium neoaurum DSM 44074
Mycobacterium canariasense CCUG 47953
Mycobacterium cosmeticum DSM 44829
Mycobacterium smegmatis NCTC 8159
Mycobacterium wolinskyi ATCC 700010
Mycobacterium mageritense CIP 104973
Mycobacterium houstonense ATCC 49403
Mycobacterium setense DSM 45070
Mycobacterium septicum DSM 44393
Mycobacterium neworleansense ATCC 49404
Mycobacterium peregrinum DSM 43271
Mycobacterium fortuitum subsp. fortuitum ATCC 6841
Mycobacterium boenickei CIP 107829
Mycobacterium conceptionense D16
Mycobacterium monacense DSM 44395
Mycobacterium doricum DSM 44339
Mycobacterium thermoresistibile ATCC 19527
Mycobacterium hassiacum DSM 44199
Mycobacterium moriokaense CIP 105393
Mycobacterium phlei CCUG 21000
Mycobacterium rutilum DSM 45405
Mycobacterium celeriflavum DSM 46765
Mycobacterium malmesburyense WCM 7299
Mycobacterium obuense DSM 44075
Mycobacterium rufum DSM 45406
Mycobacterium chubuense DSM 44219
Mycobacterium chlorophenolicum NBRC 15527
Mycobacterium iranicum DSM 45541
Mycobacterium aurum NCTC 10437
Mycobacterium parafortuitum CCUG 20999
Mycobacterium vaccae ATCC 15483
Mycobacterium vanbaalenii PYR-1
Mycobacterium austroafricanum DSM 44191
Mycobacterium triviale DSM 44153
Mycobacterium koreense KCTC 19819
Mycobacterium kumamotonense DSM 45093
Mycobacterium terrae CIP 104321
Mycobacterium algericum DSM 45454
Mycobacterium senuense DSM 44999
Mycobacterium longobardum DSM 45394
Mycobacterium arupense DSM 44942
Mycobacterium minnesotense DSM 45633
Mycobacterium nonchromogenicum DSM 44164
Mycobacterium engbaekii ATCC 27353
Mycobacterium hiberniae ATCC 49874
Mycobacterium noviomagense DSM 45145
Mycobacterium xenopi DSM 43995
Mycobacterium fragae DSM 45731
Mycobacterium shimoidei DSM 44152
Mycobacterium celatum DSM 44243
Mycobacterium branderi DSM 44624
Mycobacterium kyorinense KUM 060204
Mycobacterium haemophilum ATCC 29548
Mycobacterium intermedium DSM 44049
Mycobacterium kubicae CIP 106428
Mycobacterium gordonae DSM 44160
Mycobacterium asiaticum DSM 44297
Mycobacterium pseudoshottsii JCM 15466
Mycobacterium kansasii ATCC 12478
Mycobacterium gastri DSM 43505
Mycobacterium tuberculosis subsp. tuberculosis H37Rv
Mycobacterium shinjukuense CCUG 53584
Mycobacterium lacus DSM 44577
Mycobacterium riyadhense DSM 45176
Mycobacterium szulgai DSM 44166
Mycobacterium conspicuum DSM 44136
Mycobacterium sherrisii ATCC BAA-832
Mycobacterium simiae ATCC 25275
Mycobacterium florentinum DSM 44852
Mycobacterium genavense ATCC 51234
Mycobacterium triplex DSM 44626
Mycobacterium saskatchewanense DSM 44616
Mycobacterium bohemicum DSM 44277
Mycobacterium heidelbergense DSM 44471
Mycobacterium palustre DSM 44572
Mycobacterium alsense DSM 45230
Mycobacterium paraense IEC26
Mycobacterium interjectum ATCC 51457
Mycobacterium parmense DSM 44553
Mycobacterium nebraskense DSM 44803
Mycobacterium paraseoulense DSM 45000
Mycobacterium europaeum DSM 45397
Mycobacterium scrofulaceum DSM 43992
Mycobacterium parascrofulaceum ATCC BAA-614
Mycobacterium arosiense DSM 45069
Mycobacterium mantenii DSM 45255
Mycobacterium vulneris DSM 45247
Mycobacterium colombiense CECT 3035
Mycobacterium bouchedurhonense DSM 45439
Mycobacterium avium subsp. avium ATCC 25291
Mycobacterium marseillense DSM 45437
Mycobacterium chimaera DSM 44623
Mycobacterium yongonense 05-1390
Mycobacterium intracellulare ATCC 13950
Mycobacterium paraintracellulare KCTC 29084

Figure 1: Tree inferred with FastME from GBDP distances calculated from whole proteomes. The branches are scaled in terms of log-transformed intergenomic distances (GBDP formula d_5). The numbers above branches are GBDP pseudo-bootstrap support values from 100 replications. Tip colors indicate type species, colors to the right of the tips indicate, from left to right, class (2), order (3) and family (4; see the embedded legend for details). The blue gradient scale (5) indicates the exact G+C content as calculated from the genome sequences. Genome size (6) and ITS length (7) are display at the right-hand side.

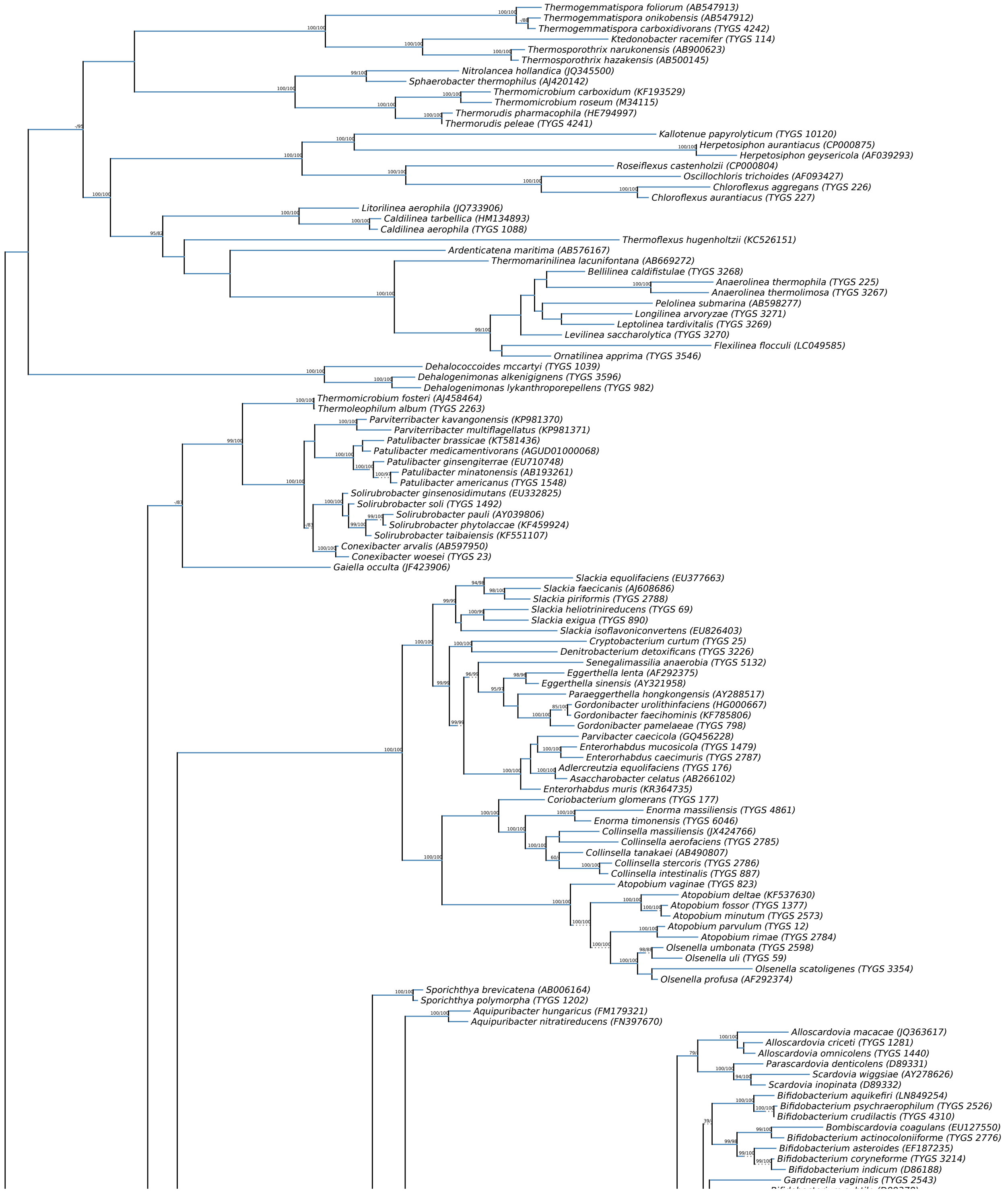

Thermogemmatispora foliorum (AB547913)
Thermogemmatispora onikobensis (AB547912)
Thermogemmatispora carboxidivorans (TYGS 4242)
Ktedonobacter racemifer (TYGS 114)
Thermosporothrix narukonensis (AB900623)
Thermosporothrix hazakensis (AB500145)
Nitrolancea hollandica (JQ345500)
Sphaerobacter thermophilus (AJ420142)
Thermomicrobium carboxidum (KF193529)
Thermomicrobium roseum (M34115)
Thermorudis pharmacophila (HE794997)
Thermorudis peleae (TYGS 4241)
Kallotenue papyrolyticum (TYGS 10120)
Herpetosiphon aurantiacus (CP000875)
Herpetosiphon geysericola (AF039293)
Roseiflexus castenholzii (CP000804)
Oscillochloris trichoides (AF093427)
Chloroflexus aggregans (TYGS 226)
Chloroflexus aurantiacus (TYGS 227)
Litorilinea aerophila (JQ733906)
Caldilinea tarbellica (HM134893)
Caldilinea aerophila (TYGS 1088)
Thermoflexus hugenholtzii (KC526151)
Ardenticatena maritima (AB576167)
Thermomarinilinea lacunifontana (AB669272)
Bellilinea caldifistulae (TYGS 3268)
Anaerolinea thermophila (TYGS 225)
Anaerolinea thermolimosa (TYGS 3267)
Pelolinea submarina (AB598277)
Longilinea arvoryzae (TYGS 3271)
Leptolinea tardivitalis (TYGS 3269)
Levilinea saccharolytica (TYGS 3270)
Flexilinea flocculi (LC049585)
Ornatilinea apprima (TYGS 3546)
Dehalococcoides mccartyi (TYGS 1039)
Dehalogenimonas alkenigignens (TYGS 3596)
Dehalogenimonas lykanthroporepellens (TYGS 982)
Thermomicrobium fosteri (AJ458464)
Thermoleophilum album (TYGS 2263)
Parviterribacter kavangonensis (KP981370)
Parviterribacter multiflagellatus (KP981371)
Patulibacter brassicae (KT581436)
Patulibacter medicamentivorans (AGUD01000068)
Patulibacter ginsengiterrae (EU710748)
Patulibacter minatonensis (AB193261)
Patulibacter americanus (TYGS 1548)
Solirubrobacter ginsenosidimutans (EU332825)
Solirubrobacter soli (TYGS 1492)
Solirubrobacter pauli (AY039806)
Solirubrobacter phytolaccae (KF459924)
Solirubrobacter taibaiensis (KF551107)
Conexibacter arvalis (AB597950)
Conexibacter woesei (TYGS 23)
Gaiella occulta (JF423906)
Slackia equolifaciens (EU377663)
Slackia faecicanis (AJ608686)
Slackia piriformis (TYGS 2788)
Slackia heliotrinireducens (TYGS 69)
Slackia exigua (TYGS 890)
Slackia isoflavoniconvertens (EU826403)
Cryptobacterium curtum (TYGS 25)
Denitrobacterium detoxificans (TYGS 3226)
Senegalimassilia anaerobia (TYGS 5132)
Eggerthella lenta (AF292375)
Eggerthella sinensis (AY321958)
Paraeggerthella hongkongensis (AY288517)
Gordonibacter urolithinfaciens (HG000667)
Gordonibacter faecihominis (KF785806)
Gordonibacter pamelaeae (TYGS 798)
Parvibacter caecicola (GQ456228)
Enterorhabdus mucosicola (TYGS 1479)
Enterorhabdus caecimuris (TYGS 2787)
Adlercreutzia equolifaciens (TYGS 176)
Asaccharobacter celatus (AB266102)
Enterorhabdus muris (KR364735)
Coriobacterium glomerans (TYGS 177)
Enorma massiliensis (TYGS 4861)
Enorma timonensis (TYGS 6046)
Collinsella massiliensis (JX424766)
Collinsella aerofaciens (TYGS 2785)
Collinsella tanakaei (AB490807)
Collinsella stercoris (TYGS 2786)
Collinsella intestinalis (TYGS 887)
Atopobium vaginae (TYGS 823)
Atopobium deltae (KF537630)
Atopobium fossor (TYGS 1377)
Atopobium minutum (TYGS 2573)
Atopobium parvulum (TYGS 12)
Atopobium rimae (TYGS 2784)
Olsenella umbonata (TYGS 2598)
Olsenella uli (TYGS 59)
Olsenella scatoligenes (TYGS 3354)
Olsenella profusa (AF292374)
Sporichthya brevicatena (AB006164)
Sporichthya polymorpha (TYGS 1202)
Aquipuribacter hungaricus (FM179321)
Aquipuribacter nitratireducens (FN397670)
Alloscardovia macacae (JQ363617)
Alloscardovia criceti (TYGS 1281)
Alloscardovia omnicolens (TYGS 1440)
Parascardovia denticolens (D89331)
Scardovia wiggsiae (AY278626)
Scardovia inopinata (D89332)
Bifidobacterium aquikefiri (LN849254)
Bifidobacterium psychraerophilum (TYGS 2526)
Bifidobacterium crudilactis (TYGS 4310)
Bombiscardovia coagulans (EU127550)
Bifidobacterium actinocoloniiforme (TYGS 2776)
Bifidobacterium asteroides (EF187235)
Bifidobacterium coryneforme (TYGS 3214)
Bifidobacterium indicum (D86188)
Gardnerella vaginalis (TYGS 2543)

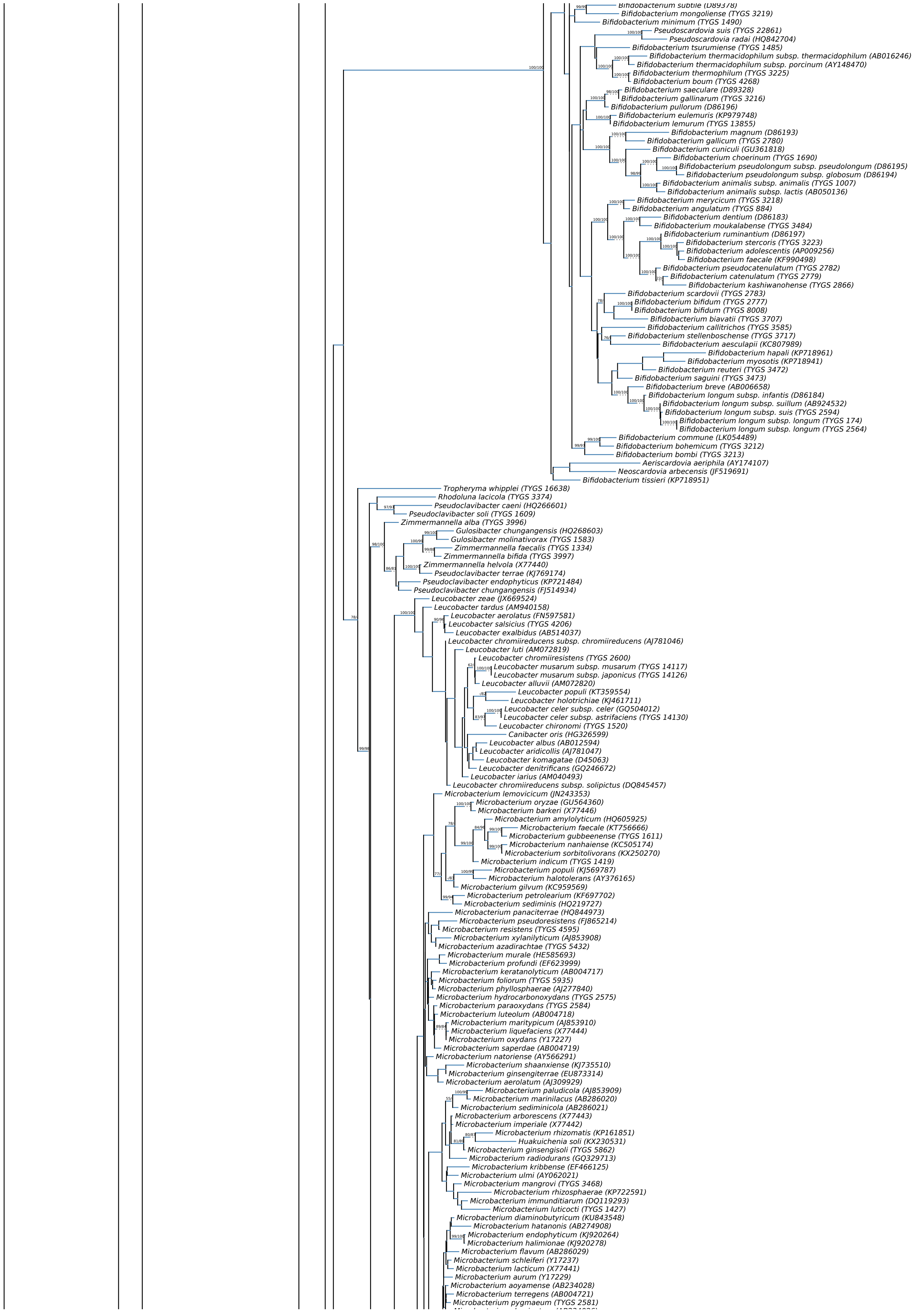

Bifidobacterium subtile (D89378)
Bifidobacterium mongoliense (TYGS 3219)
Bifidobacterium minimum (TYGS 1490)
Pseudoscardovia suis (TYGS 22861)
Pseudoscardovia radai (HQ842704)
Bifidobacterium tsurumiense (TYGS 1485)
Bifidobacterium thermacidophilum subsp. thermacidophilum (AB016246)
Bifidobacterium thermacidophilum subsp. porcinum (AY148470)
Bifidobacterium thermophilum (TYGS 3225)
Bifidobacterium boum (TYGS 4268)
Bifidobacterium saeculare (D89328)
Bifidobacterium gallinarum (TYGS 3216)
Bifidobacterium pullorum (D86196)
Bifidobacterium eulemuris (KP979748)
Bifidobacterium lemurum (TYGS 13855)
Bifidobacterium magnum (D86193)
Bifidobacterium gallicum (TYGS 2780)
Bifidobacterium cuniculi (GU361818)
Bifidobacterium choerinum (TYGS 1690)
Bifidobacterium pseudolongum subsp. pseudolongum (D86195)
Bifidobacterium pseudolongum subsp. globosum (D86194)
Bifidobacterium animalis subsp. animalis (TYGS 1007)
Bifidobacterium animalis subsp. lactis (AB050136)
Bifidobacterium merycicum (TYGS 3218)
Bifidobacterium angulatum (TYGS 884)
Bifidobacterium dentium (D86183)
Bifidobacterium moukalabense (TYGS 3484)
Bifidobacterium ruminantium (D86197)
Bifidobacterium stercoris (TYGS 3223)
Bifidobacterium adolescentis (AP009256)
Bifidobacterium faecale (KF990498)
Bifidobacterium pseudocatenulatum (TYGS 2782)
Bifidobacterium catenulatum (TYGS 2779)
Bifidobacterium kashiwanohense (TYGS 2866)
Bifidobacterium scardovii (TYGS 2783)
Bifidobacterium bifidum (TYGS 2777)
Bifidobacterium bifidum (TYGS 8008)
Bifidobacterium biavatii (TYGS 3707)
Bifidobacterium callitrichos (TYGS 3585)
Bifidobacterium stellenboschense (TYGS 3717)
Bifidobacterium aesculapii (KC807989)
Bifidobacterium hapali (KP718961)
Bifidobacterium myosotis (KP718941)
Bifidobacterium reuteri (TYGS 3472)
Bifidobacterium saguini (TYGS 3473)
Bifidobacterium breve (AB006658)
Bifidobacterium longum subsp. infantis (D86184)
Bifidobacterium longum subsp. suillum (AB924532)
Bifidobacterium longum subsp. suis (TYGS 2594)
Bifidobacterium longum subsp. longum (TYGS 174)
Bifidobacterium longum subsp. longum (TYGS 2564)
Bifidobacterium commune (LK054489)
Bifidobacterium bohemicum (TYGS 3212)
Bifidobacterium bombi (TYGS 3213)
Aeriscardovia aeriphila (AY174107)
Neoscardovia arbecensis (JF519691)
Bifidobacterium tissieri (KP718951)
Tropheryma whipplei (TYGS 16638)
Rhodoluna lacicola (TYGS 3374)
Pseudoclavibacter caeni (HQ266601)
Pseudoclavibacter soli (TYGS 1609)
Zimmermannella alba (TYGS 3996)
Gulosibacter chungangensis (HQ268603)
Gulosibacter molinativorax (TYGS 1583)
Zimmermannella faecalis (TYGS 1334)
Zimmermannella bifida (TYGS 3997)
Zimmermannella helvola (X77440)
Pseudoclavibacter terrae (KJ769174)
Pseudoclavibacter endophyticus (KP721484)
Pseudoclavibacter chungangensis (FJ514934)
Leucobacter zeae (JX669524)
Leucobacter tardus (AM940158)
Leucobacter aerolatus (FN597581)
Leucobacter salsicius (TYGS 4206)
Leucobacter exalbidus (AB514037)
Leucobacter chromiireducens subsp. chromiireducens (AJ781046)
Leucobacter luti (AM072819)
Leucobacter chromiiresistens (TYGS 2600)
Leucobacter musarum subsp. musarum (TYGS 14117)
Leucobacter musarum subsp. japonicus (TYGS 14126)
Leucobacter alluvii (AM072820)
Leucobacter populi (KT359554)
Leucobacter holotrichiae (KJ461711)
Leucobacter celer subsp. celer (GQ504012)
Leucobacter celer subsp. astrifaciens (TYGS 14130)
Leucobacter chironomi (TYGS 1520)
Canibacter oris (HG326599)
Leucobacter albus (AB012594)
Leucobacter aridicollis (AJ781047)
Leucobacter komagatae (D45063)
Leucobacter denitrificans (GQ246672)
Leucobacter iarius (AM040493)
Leucobacter chromiireducens subsp. solipictus (DQ845457)
Microbacterium lemovicicum (JN243353)
Microbacterium oryzae (GU564360)
Microbacterium barkeri (X77446)
Microbacterium amylolyticum (HQ605925)
Microbacterium faecale (KT756666)
Microbacterium gubbeenense (TYGS 1611)
Microbacterium nanhaiense (KC505174)
Microbacterium sorbitolivorans (KX250270)
Microbacterium indicum (TYGS 1419)
Microbacterium populi (KJ569787)
Microbacterium halotolerans (AY376165)
Microbacterium gilvum (KC959569)
Microbacterium petrolearium (KF697702)
Microbacterium sediminis (HQ219727)
Microbacterium panaciterrae (HQ844973)
Microbacterium pseudoresistens (FJ865214)
Microbacterium resistens (TYGS 4595)
Microbacterium xylanilyticum (AJ853908)
Microbacterium azadirachtae (TYGS 5432)
Microbacterium murale (HE585693)
Microbacterium profundi (EF623999)
Microbacterium keratanolyticum (AB004717)
Microbacterium foliorum (TYGS 5935)
Microbacterium phyllosphaerae (AJ277840)
Microbacterium hydrocarbonoxydans (TYGS 2575)
Microbacterium paraoxydans (TYGS 2584)
Microbacterium luteolum (AB004718)
Microbacterium maritypicum (AJ853910)
Microbacterium liquefaciens (X77444)
Microbacterium oxydans (Y17227)
Microbacterium saperdae (AB004719)
Microbacterium natoriense (AY566291)
Microbacterium shaanxiense (KJ735510)
Microbacterium ginsengiterrae (EU873314)
Microbacterium aerolatum (AJ309929)
Microbacterium paludicola (AJ853909)
Microbacterium marinilacus (AB286020)
Microbacterium sediminicola (AB286021)
Microbacterium arborescens (X77443)
Microbacterium imperiale (X77442)
Microbacterium rhizomatis (KP161851)
Huakuichenia soli (KX230531)
Microbacterium ginsengisoli (TYGS 5862)
Microbacterium radiodurans (GQ329713)
Microbacterium kribbense (EF466125)
Microbacterium ulmi (AY062021)
Microbacterium mangrovi (TYGS 3468)
Microbacterium rhizosphaerae (KP722591)
Microbacterium immunditiarum (DQ119293)
Microbacterium luticocti (TYGS 1427)
Microbacterium diaminobutyricum (KU843548)
Microbacterium hatanonis (AB274908)
Microbacterium endophyticum (KJ920264)
Microbacterium halimionae (KJ920278)
Microbacterium flavum (AB286029)
Microbacterium schleiferi (Y17237)
Microbacterium lacticum (X77441)
Microbacterium aurum (Y17229)
Microbacterium aoyamense (AB234028)
Microbacterium terregens (AB004721)
Microbacterium pygmaeum (TYGS 2581)

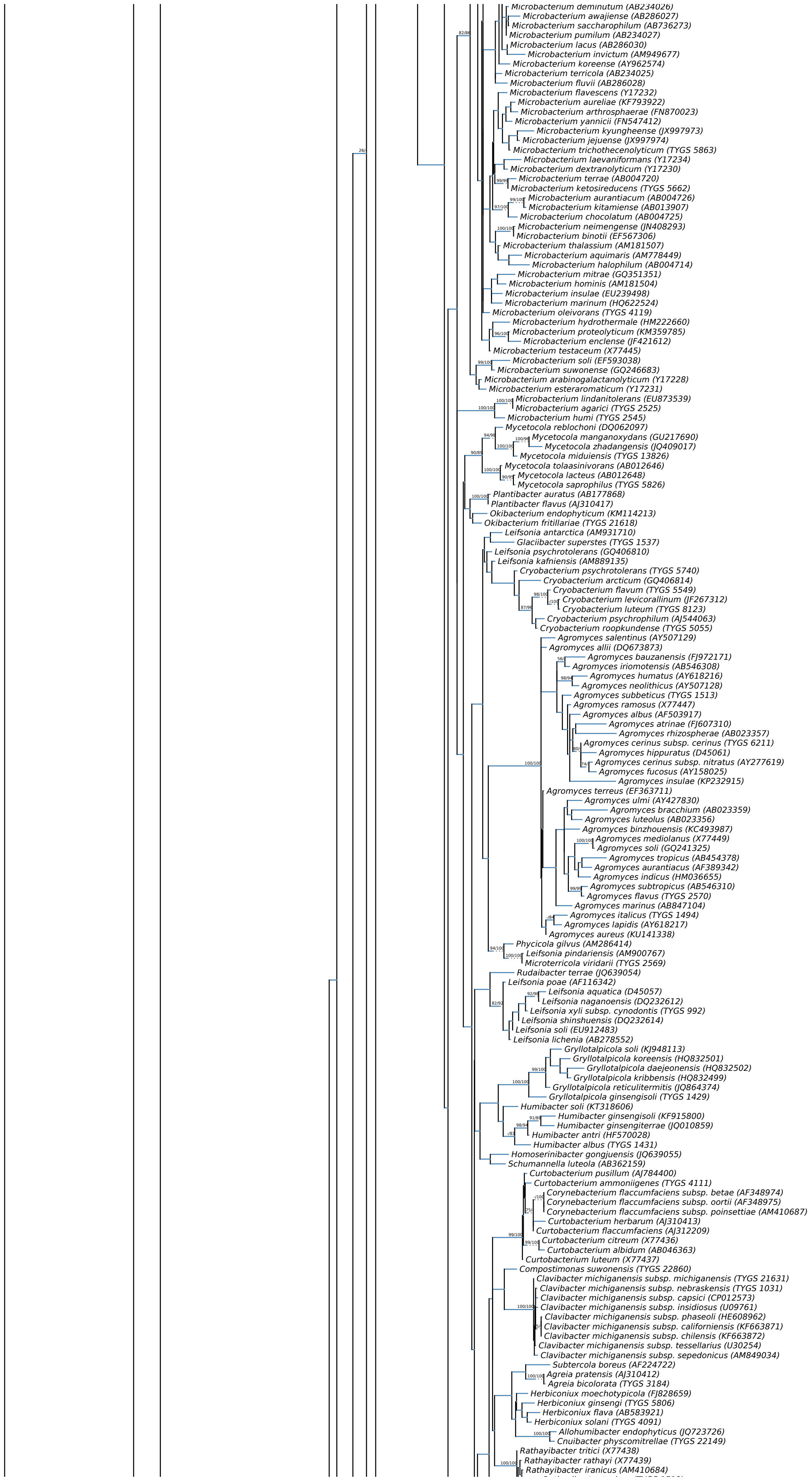
Microbacterium deminutum (AB234026)
Microbacterium awajiense (AB286027)
Microbacterium saccharophilum (AB736273)
Microbacterium pumilum (AB234027)
Microbacterium lacus (AB286030)
Microbacterium invictum (AM949677)
Microbacterium koreense (AY962574)
Microbacterium terricola (AB234025)
Microbacterium fluvii (AB286028)
Microbacterium flavescens (Y17232)
Microbacterium aureliae (KF793922)
Microbacterium arthrosphaerae (FN870023)
Microbacterium yannicii (FN547412)
Microbacterium kyungheense (JX997973)
Microbacterium jejuense (JX997974)
Microbacterium trichothecenolyticum (TYGS 5863)
Microbacterium laevaniformans (Y17234)
Microbacterium dextranolyticum (Y17230)
Microbacterium terrae (AB004720)
Microbacterium ketosireducens (TYGS 5662)
Microbacterium aurantiacum (AB004726)
Microbacterium kitamiense (AB013907)
Microbacterium chocolatum (AB004725)
Microbacterium neimengense (JN408293)
Microbacterium binotii (EF567306)
Microbacterium thalassium (AM181507)
Microbacterium aquimaris (AM778449)
Microbacterium halophilum (AB004714)
Microbacterium mitrae (GQ351351)
Microbacterium hominis (AM181504)
Microbacterium insulae (EU239498)
Microbacterium marinum (HQ622524)
Microbacterium oleivorans (TYGS 4119)
Microbacterium hydrothermale (HM222660)
Microbacterium proteolyticum (KM359785)
Microbacterium enclense (JF421612)
Microbacterium testaceum (X77445)
Microbacterium soli (EF593038)
Microbacterium suwonense (GQ246683)
Microbacterium arabinogalactanolyticum (Y17228)
Microbacterium esteraromaticum (Y17231)
Microbacterium lindanitolerans (EU873539)
Microbacterium agarici (TYGS 2525)
Microbacterium humi (TYGS 2545)
Mycetocola reblochoni (DQ062097)
Mycetocola manganoxydans (GU217690)
Mycetocola zhadangensis (JQ409017)
Mycetocola miduiensis (TYGS 13826)
Mycetocola tolaasinivorans (AB012646)
Mycetocola lacteus (AB012648)
Mycetocola saprophilus (TYGS 5826)
Plantibacter auratus (AB177868)
Plantibacter flavus (AJ310417)
Okibacterium endophyticum (KM114213)
Okibacterium fritillariae (TYGS 21618)
Leifsonia antarctica (AM931710)
Glaciibacter superstes (TYGS 1537)
Leifsonia psychrotolerans (GQ406810)
Leifsonia kafniensis (AM889135)
Cryobacterium psychrotolerans (TYGS 5740)
Cryobacterium arcticum (GQ406814)
Cryobacterium flavum (TYGS 5549)
Cryobacterium levicorallinum (JF267312)
Cryobacterium luteum (TYGS 8123)
Cryobacterium psychrophilum (AJ544063)
Cryobacterium roopkundense (TYGS 5055)
Agromyces salentinus (AY507129)
Agromyces allii (DQ673873)
Agromyces bauzanensis (FJ972171)
Agromyces iriomotensis (AB546308)
Agromyces humatus (AY618216)
Agromyces neolithicus (AY507128)
Agromyces subbeticus (TYGS 1513)
Agromyces ramosus (X77447)
Agromyces albus (AF503917)
Agromyces atrinae (FJ607310)
Agromyces rhizospherae (AB023357)
Agromyces cerinus subsp. cerinus (TYGS 6211)
Agromyces hippuratus (D45061)
Agromyces cerinus subsp. nitratus (AY277619)
Agromyces fucosus (AY158025)
Agromyces insulae (KP232915)
Agromyces terreus (EF363711)
Agromyces ulmi (AY427830)
Agromyces bracchium (AB023359)
Agromyces luteolus (AB023356)
Agromyces binzhouensis (KC493987)
Agromyces mediolanus (X77449)
Agromyces soli (GQ241325)
Agromyces tropicus (AB454378)
Agromyces aurantiacus (AF389342)
Agromyces indicus (HM036655)
Agromyces subtropicus (AB546310)
Agromyces flavus (TYGS 2570)
Agromyces marinus (AB847104)
Agromyces italicus (TYGS 1494)
Agromyces lapidis (AY618217)
Agromyces aureus (KU141338)
Phycicola gilvus (AM286414)
Leifsonia pindariensis (AM900767)
Microterricola viridarii (TYGS 2569)
Rudaibacter terrae (JQ639054)
Leifsonia poae (AF116342)
Leifsonia aquatica (D45057)
Leifsonia naganoensis (DQ232612)
Leifsonia xyli subsp. cynodontis (TYGS 992)
Leifsonia shinshuensis (DQ232614)
Leifsonia soli (EU912483)
Leifsonia lichenia (AB278552)
Gryllotalpicola soli (KJ948113)
Gryllotalpicola koreensis (HQ832501)
Gryllotalpicola daejeonensis (HQ832502)
Gryllotalpicola kribbensis (HQ832499)
Gryllotalpicola reticulitermitis (JQ864374)
Gryllotalpicola ginsengisoli (TYGS 1429)
Humibacter soli (KT318606)
Humibacter ginsengisoli (KF915800)
Humibacter ginsengiterrae (JQ010859)
Humibacter antri (HF570028)
Humibacter albus (TYGS 1431)
Homoserinibacter gongjuensis (JQ639055)
Schumannella luteola (AB362159)
Curtobacterium pusillum (AJ784400)
Curtobacterium ammoniigenes (TYGS 4111)
Corynebacterium flaccumfaciens subsp. betae (AF348974)
Corynebacterium flaccumfaciens subsp. oortii (AF348975)
Corynebacterium flaccumfaciens subsp. poinsettiae (AM410687)
Curtobacterium herbarum (AJ310413)
Curtobacterium flaccumfaciens (AJ312209)
Curtobacterium citreum (X77436)
Curtobacterium albidum (AB046363)
Curtobacterium luteum (X77437)
Compostimonas suwonensis (TYGS 22860)
Clavibacter michiganensis subsp. michiganensis (TYGS 21631)
Clavibacter michiganensis subsp. nebraskensis (TYGS 1031)
Clavibacter michiganensis subsp. capsici (CP012573)
Clavibacter michiganensis subsp. insidiosus (U09761)
Clavibacter michiganensis subsp. phaseoli (HE608962)
Clavibacter michiganensis subsp. californiensis (KF663871)
Clavibacter michiganensis subsp. chilensis (KF663872)
Clavibacter michiganensis subsp. tessellarius (U30254)
Clavibacter michiganensis subsp. sepedonicus (AM849034)
Subtercola boreus (AF224722)
Agreia pratensis (AJ310412)
Agreia bicolorata (TYGS 3184)
Herbiconiux moechotypicola (FJ828659)
Herbiconiux ginsengi (TYGS 5806)
Herbiconiux flava (AB583921)
Herbiconiux solani (TYGS 4091)
Allohumibacter endophyticus (JQ723726)
Cnuibacter physcomitrellae (TYGS 22149)
Rathayibacter tritici (X77438)
Rathayibacter rathayi (X77439)
Rathayibacter iranicus (AM410684)

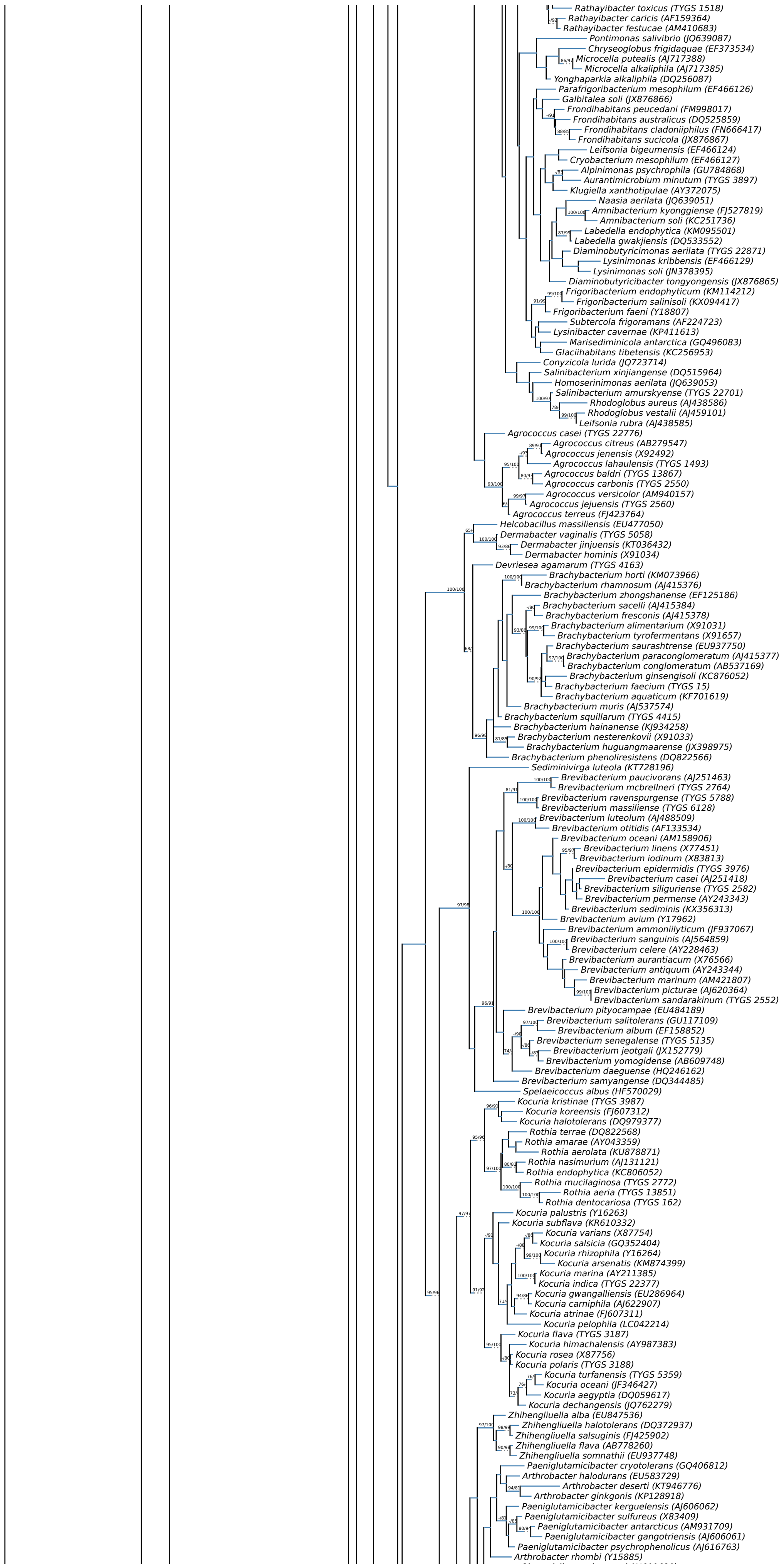
Rathayibacter toxicus (TYGS 1518)
Rathayibacter caricis (AF159364)
Rathayibacter festucae (AM410683)
Pontimonas salivibrio (JQ639087)
Chryseoglobus frigidaquae (EF373534)
Microcella putealis (AJ717388)
Microcella alkaliphila (AJ717385)
Yonghaparkia alkaliphila (DQ256087)
Parafrigoribacterium mesophilum (EF466126)
Galbitalea soli (JX876866)
Frondihabitans peucedani (FM998017)
Frondihabitans australicus (DQ525859)
Frondihabitans cladoniiphilus (FN666417)
Frondihabitans sucicola (JX876867)
Leifsonia bigeumensis (EF466124)
Cryobacterium mesophilum (EF466127)
Alpinimonas psychrophila (GU784868)
Aurantimicrobium minutum (TYGS 3897)
Klugiella xanthotipulae (AY372075)
Naasia aerilata (JQ639051)
Amnibacterium kyonggiense (FJ527819)
Amnibacterium soli (KC251736)
Labedella endophytica (KM095501)
Labedella gwakjiensis (DQ533552)
Diaminobutyricimonas aerilata (TYGS 22871)
Lysinimonas kribbensis (EF466129)
Lysinimonas soli (JN378395)
Diaminobutyricibacter tongyongensis (JX876865)
Frigoribacterium endophyticum (KM114212)
Frigoribacterium salinisoli (KX094417)
Frigoribacterium faeni (Y18807)
Subtercola frigoramans (AF224723)
Lysinibacter cavernae (KP411613)
Marisediminicola antarctica (GQ496083)
Glaciihabitans tibetensis (KC256953)
Conyzicola lurida (JQ723714)
Salinibacterium xinjiangense (DQ515964)
Homoserinimonas aerilata (JQ639053)
Salinibacterium amurskyense (TYGS 22701)
Rhodoglobus aureus (AJ438586)
Rhodoglobus vestalii (AJ459101)
Leifsonia rubra (AJ438585)
Agrococcus casei (TYGS 22776)
Agrococcus citreus (AB279547)
Agrococcus jenensis (X92492)
Agrococcus lahaulensis (TYGS 1493)
Agrococcus baldri (TYGS 13867)
Agrococcus carbonis (TYGS 2550)
Agrococcus versicolor (AM940157)
Agrococcus jejuensis (TYGS 2560)
Agrococcus terreus (FJ423764)
Helcobacillus massiliensis (EU477050)
Dermabacter vaginalis (TYGS 5058)
Dermabacter jinjuensis (KT036432)
Dermabacter hominis (X91034)
Devriesea agamarum (TYGS 4163)
Brachybacterium horti (KM073966)
Brachybacterium rhamnosum (AJ415376)
Brachybacterium zhongshanense (EF125186)
Brachybacterium sacelli (AJ415384)
Brachybacterium fresconis (AJ415378)
Brachybacterium alimentarium (X91031)
Brachybacterium tyrofermentans (X91657)
Brachybacterium saurashtrense (EU937750)
Brachybacterium paraconglomeratum (AJ415377)
Brachybacterium conglomeratum (AB537169)
Brachybacterium ginsengisoli (KC876052)
Brachybacterium faecium (TYGS 15)
Brachybacterium aquaticum (KF701619)
Brachybacterium muris (AJ537574)
Brachybacterium squillarum (TYGS 4415)
Brachybacterium hainanense (KJ934258)
Brachybacterium nesterenkovii (X91033)
Brachybacterium huguangmaarense (JX398975)
Brachybacterium phenoliresistens (DQ822566)
Sediminivirga luteola (KT728196)
Brevibacterium paucivorans (AJ251463)
Brevibacterium mcbrellneri (TYGS 2764)
Brevibacterium ravenspurgense (TYGS 5788)
Brevibacterium massiliense (TYGS 6128)
Brevibacterium luteolum (AJ488509)
Brevibacterium otitidis (AF133534)
Brevibacterium oceani (AM158906)
Brevibacterium linens (X77451)
Brevibacterium iodinum (X83813)
Brevibacterium epidermidis (TYGS 3976)
Brevibacterium casei (AJ251418)
Brevibacterium siliguriense (TYGS 2582)
Brevibacterium permense (AY243343)
Brevibacterium sediminis (KX356313)
Brevibacterium avium (Y17962)
Brevibacterium ammoniilyticum (JF937067)
Brevibacterium sanguinis (AJ564859)
Brevibacterium celere (AY228463)
Brevibacterium aurantiacum (X76566)
Brevibacterium antiquum (AY243344)
Brevibacterium marinum (AM421807)
Brevibacterium picturae (AJ620364)
Brevibacterium sandarakinum (TYGS 2552)
Brevibacterium pityocampae (EU484189)
Brevibacterium salitolerans (GU117109)
Brevibacterium album (EF158852)
Brevibacterium senegalense (TYGS 5135)
Brevibacterium jeotgali (JX152779)
Brevibacterium yomogidense (AB609748)
Brevibacterium daeguense (HQ246162)
Brevibacterium samyangense (DQ344485)
Spelaeicoccus albus (HF570029)
Kocuria kristinae (TYGS 3987)
Kocuria koreensis (FJ607312)
Kocuria halotolerans (DQ979377)
Rothia terrae (DQ822568)
Rothia amarae (AY043359)
Rothia aerolata (KU878871)
Rothia nasimurium (AJ131121)
Rothia endophytica (KC806052)
Rothia mucilaginosa (TYGS 2772)
Rothia aeria (TYGS 13851)
Rothia dentocariosa (TYGS 162)
Kocuria palustris (Y16263)
Kocuria subflava (KR610332)
Kocuria varians (X87754)
Kocuria salsicia (GQ352404)
Kocuria rhizophila (Y16264)
Kocuria arsenatis (KM874399)
Kocuria marina (AY211385)
Kocuria indica (TYGS 22377)
Kocuria gwangalliensis (EU286964)
Kocuria carniphila (AJ622907)
Kocuria atrinae (FJ607311)
Kocuria pelophila (LC042214)
Kocuria flava (TYGS 3187)
Kocuria himachalensis (AY987383)
Kocuria rosea (X87756)
Kocuria polaris (TYGS 3188)
Kocuria turfanensis (TYGS 5359)
Kocuria oceani (JF346427)
Kocuria aegyptia (DQ059617)
Kocuria dechangensis (JQ762279)
Zhihengliuella alba (EU847536)
Zhihengliuella halotolerans (DQ372937)
Zhihengliuella salsuginis (FJ425902)
Zhihengliuella flava (AB778260)
Zhihengliuella somnathii (EU937748)
Paeniglutamicibacter cryotolerans (GQ406812)
Arthrobacter halodurans (EU583729)
Arthrobacter deserti (KT946776)
Arthrobacter ginkgonis (KP128918)
Paeniglutamicibacter kerguelensis (AJ606062)
Paeniglutamicibacter sulfureus (X83409)
Paeniglutamicibacter antarcticus (AM931709)
Paeniglutamicibacter gangotriensis (AJ606061)
Paeniglutamicibacter psychrophenolicus (AJ616763)
Arthrobacter rhombi (Y15885)

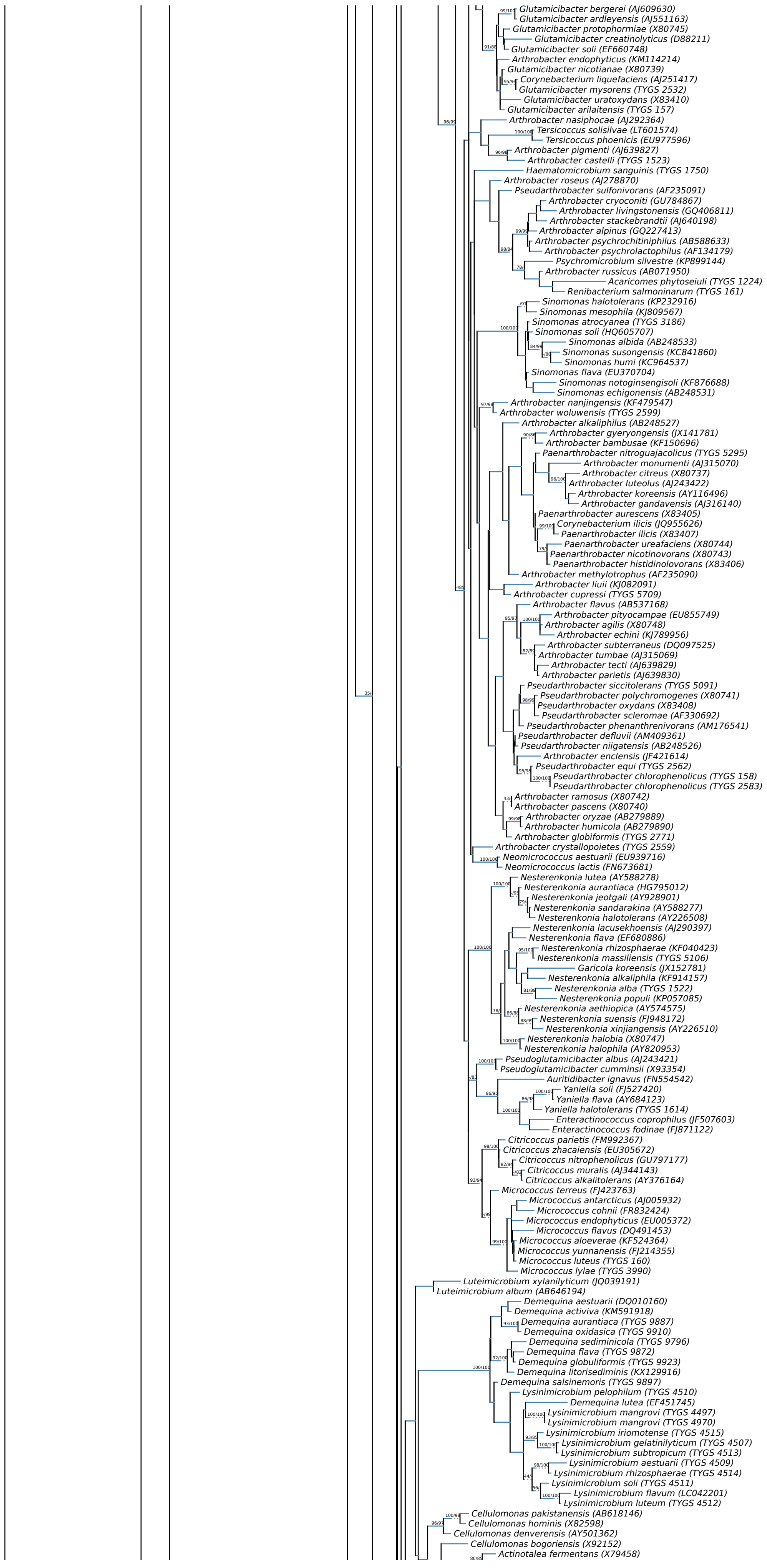

Glutamicibacter bergerei (AJ609630)
Glutamicibacter ardleyensis (AJ551163)
Glutamicibacter protophormiae (X80745)
Glutamicibacter creatinolyticus (D88211)
Glutamicibacter soli (EF660748)
Arthrobacter endophyticus (KM114214)
Glutamicibacter nicotianae (X80739)
Corynebacterium liquefaciens (AJ251417)
Glutamicibacter mysorens (TYGS 2532)
Glutamicibacter uratoxydans (X83410)
Glutamicibacter arilaitensis (TYGS 157)
Arthrobacter nasiphocae (AJ292364)
Tersicoccus solisilvae (LT601574)
Tersicoccus phoenicis (EU977596)
Arthrobacter pigmenti (AJ639827)
Arthrobacter castelli (TYGS 1523)
Haematomicrobium sanguinis (TYGS 1750)
Arthrobacter roseus (AJ278870)
Pseudarthrobacter sulfonivorans (AF235091)
Arthrobacter cryoconiti (GU784867)
Arthrobacter livingstonensis (GQ406811)
Arthrobacter stackebrandtii (AJ640198)
Arthrobacter alpinus (GQ227413)
Arthrobacter psychrochitiniphilus (AB588633)
Arthrobacter psychrolactophilus (AF134179)
Psychromicrobium silvestre (KP899144)
Arthrobacter russicus (AB071950)
Acaricomes phytoseiuli (TYGS 1224)
Renibacterium salmoninarum (TYGS 161)
Sinomonas halotolerans (KP232916)
Sinomonas mesophila (KJ809567)
Sinomonas atrocyanea (TYGS 3186)
Sinomonas soli (HQ605707)
Sinomonas albida (AB248533)
Sinomonas susongensis (KC841860)
Sinomonas humi (KC964537)
Sinomonas flava (EU370704)
Sinomonas notoginsengisoli (KF876688)
Sinomonas echigonensis (AB248531)
Arthrobacter nanjingensis (KF479547)
Arthrobacter woluwensis (TYGS 2599)
Arthrobacter alkaliphilus (AB248527)
Arthrobacter gyeryongensis (JX141781)
Arthrobacter bambusae (KF150696)
Paenarthrobacter nitroguajacolicus (TYGS 5295)
Arthrobacter monumenti (AJ315070)
Arthrobacter citreus (X80737)
Arthrobacter luteolus (AJ243422)
Arthrobacter koreensis (AY116496)
Arthrobacter gandavensis (AJ316140)
Paenarthrobacter aurescens (X83405)
Corynebacterium ilicis (JQ955626)
Paenarthrobacter ilicis (X83407)
Paenarthrobacter ureafaciens (X80744)
Paenarthrobacter nicotinovorans (X80743)
Paenarthrobacter histidinolovorans (X83406)
Arthrobacter methylotrophus (AF235090)
Arthrobacter liuii (KJ082091)
Arthrobacter cupressi (TYGS 5709)
Arthrobacter flavus (AB537168)
Arthrobacter pityocampae (EU855749)
Arthrobacter agilis (X80748)
Arthrobacter echini (KJ789956)
Arthrobacter subterraneus (DQ097525)
Arthrobacter tumbae (AJ315069)
Arthrobacter tecti (AJ639829)
Arthrobacter parietis (AJ639830)
Pseudarthrobacter siccitolerans (TYGS 5091)
Pseudarthrobacter polychromogenes (X80741)
Pseudarthrobacter oxydans (X83408)
Pseudarthrobacter scleromae (AF330692)
Pseudarthrobacter phenanthrenivorans (AM176541)
Pseudarthrobacter defluvii (AM409361)
Pseudarthrobacter niigatensis (AB248526)
Arthrobacter enclensis (JF421614)
Pseudarthrobacter equi (TYGS 2562)
Pseudarthrobacter chlorophenolicus (TYGS 158)
Pseudarthrobacter chlorophenolicus (TYGS 2583)
Arthrobacter ramosus (X80742)
Arthrobacter pascens (X80740)
Arthrobacter oryzae (AB279889)
Arthrobacter humicola (AB279890)
Arthrobacter globiformis (TYGS 2771)
Arthrobacter crystallopoietes (TYGS 2559)
Neomicrococcus aestuarii (EU939716)
Neomicrococcus lactis (FN673681)
Nesterenkonia lutea (AY588278)
Nesterenkonia aurantiaca (HG795012)
Nesterenkonia jeotgali (AY928901)
Nesterenkonia sandarakina (AY588277)
Nesterenkonia halotolerans (AY226508)
Nesterenkonia lacusekhoensis (AJ290397)
Nesterenkonia flava (EF680886)
Nesterenkonia rhizosphaerae (KF040423)
Nesterenkonia massiliensis (TYGS 5106)
Garicola koreensis (JX152781)
Nesterenkonia alkaliphila (KF914157)
Nesterenkonia alba (TYGS 1522)
Nesterenkonia populi (KP057085)
Nesterenkonia aethiopica (AY574575)
Nesterenkonia suensis (FJ948172)
Nesterenkonia xinjiangensis (AY226510)
Nesterenkonia halobia (X80747)
Nesterenkonia halophila (AY820953)
Pseudoglutamicibacter albus (AJ243421)
Pseudoglutamicibacter cumminsii (X93354)
Auritidibacter ignavus (FN554542)
Yaniella soli (FJ527420)
Yaniella flava (AY684123)
Yaniella halotolerans (TYGS 1614)
Enteractinococcus coprophilus (JF507603)
Enteractinococcus fodinae (FJ871122)
Citricoccus parietis (FM992367)
Citricoccus zhacaiensis (EU305672)
Citricoccus nitrophenolicus (GU797177)
Citricoccus muralis (AJ344143)
Citricoccus alkalitolerans (AY376164)
Micrococcus terreus (FJ423763)
Micrococcus antarcticus (AJ005932)
Micrococcus cohnii (FR832424)
Micrococcus endophyticus (EU005372)
Micrococcus flavus (DQ491453)
Micrococcus aloeverae (KF524364)
Micrococcus yunnanensis (FJ214355)
Micrococcus luteus (TYGS 160)
Micrococcus lylae (TYGS 3990)
Luteimicrobium xylanilyticum (JQ039191)
Luteimicrobium album (AB646194)
Demequina aestuarii (DQ010160)
Demequina activiva (KM591918)
Demequina aurantiaca (TYGS 9887)
Demequina oxidasica (TYGS 9910)
Demequina sediminicola (TYGS 9796)
Demequina flava (TYGS 9872)
Demequina globuliformis (TYGS 9923)
Demequina litorisediminis (KX129916)
Demequina salsinemoris (TYGS 9897)
Lysinimicrobium pelophilum (TYGS 4510)
Demequina lutea (EF451745)
Lysinimicrobium mangrovi (TYGS 4497)
Lysinimicrobium mangrovi (TYGS 4970)
Lysinimicrobium iriomotense (TYGS 4515)
Lysinimicrobium gelatinilyticum (TYGS 4507)
Lysinimicrobium subtropicum (TYGS 4513)
Lysinimicrobium aestuarii (TYGS 4509)
Lysinimicrobium rhizosphaerae (TYGS 4514)
Lysinimicrobium soli (TYGS 4511)
Lysinimicrobium flavum (LC042201)
Lysinimicrobium luteum (TYGS 4512)
Cellulomonas pakistanensis (AB618146)
Cellulomonas hominis (X82598)
Cellulomonas denverensis (AY501362)
Cellulomonas bogoriensis (X92152)
Actinotalea fermentans (X79458)

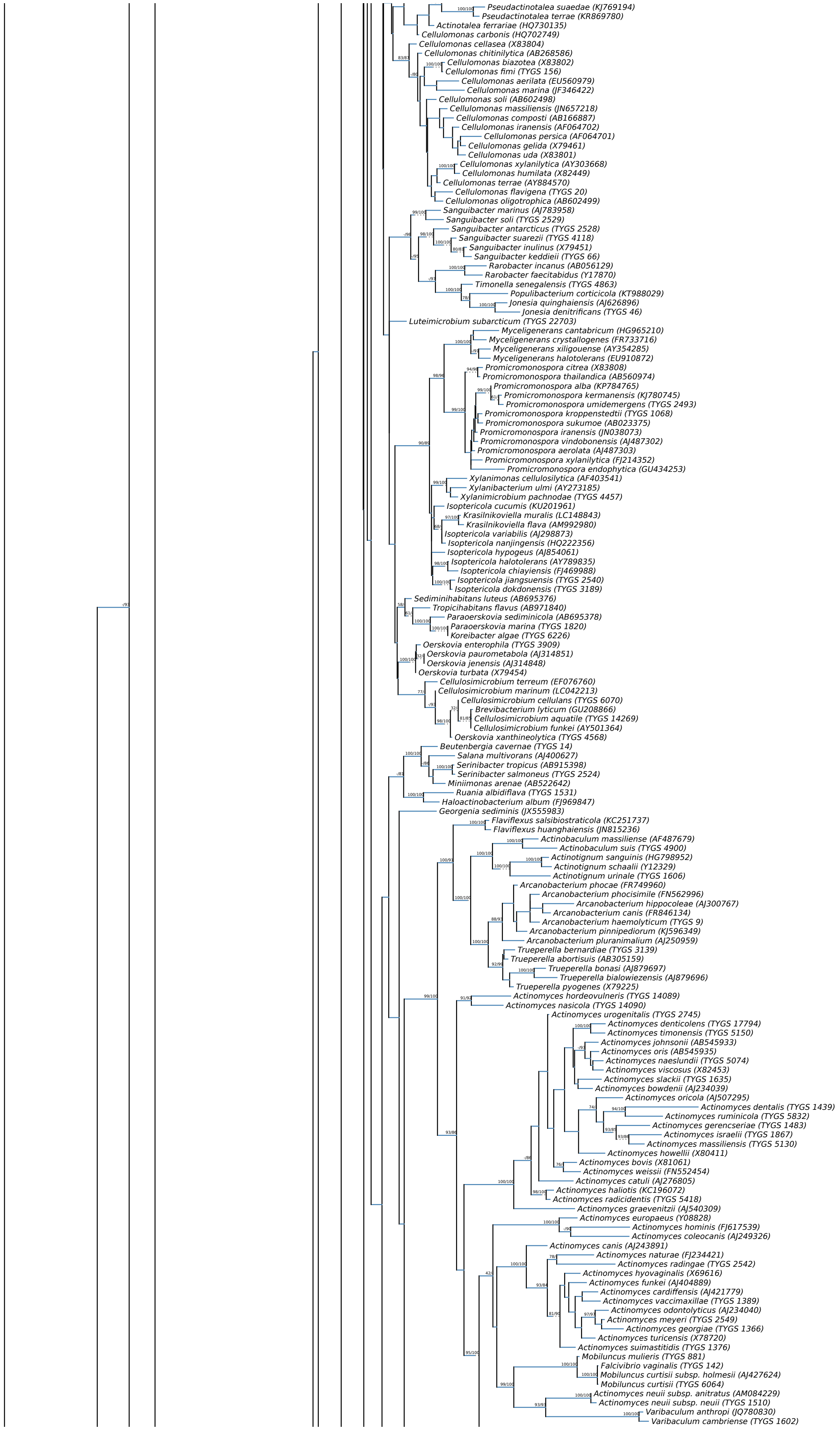
Pseudactinotalea suaedae (KJ769194)
Pseudactinotalea terrae (KR869780)
Actinotalea ferrariae (HQ730135)
Cellulomonas carbonis (HQ702749)
Cellulomonas cellasea (X83804)
Cellulomonas chitinilytica (AB268586)
Cellulomonas biazotea (X83802)
Cellulomonas fimi (TYGS 156)
Cellulomonas aerilata (EU560979)
Cellulomonas marina (JF346422)
Cellulomonas soli (AB602498)
Cellulomonas massiliensis (JN657218)
Cellulomonas composti (AB166887)
Cellulomonas iranensis (AF064702)
Cellulomonas persica (AF064701)
Cellulomonas gelida (X79461)
Cellulomonas uda (X83801)
Cellulomonas xylanilytica (AY303668)
Cellulomonas humilata (X82449)
Cellulomonas terrae (AY884570)
Cellulomonas flavigena (TYGS 20)
Cellulomonas oligotrophica (AB602499)
Sanguibacter marinus (AJ783958)
Sanguibacter soli (TYGS 2529)
Sanguibacter antarcticus (TYGS 2528)
Sanguibacter suarezii (TYGS 4118)
Sanguibacter inulinus (X79451)
Sanguibacter keddieii (TYGS 66)
Rarobacter incanus (AB056129)
Rarobacter faecitabidus (Y17870)
Timonella senegalensis (TYGS 4863)
Populibacterium corticicola (KT988029)
Jonesia quinghaiensis (AJ626896)
Jonesia denitrificans (TYGS 46)
Luteimicrobium subarcticum (TYGS 22703)
Myceligenerans cantabricum (HG965210)
Myceligenerans crystallogenes (FR733716)
Myceligenerans xiligouense (AY354285)
Myceligenerans halotolerans (EU910872)
Promicromonospora citrea (X83808)
Promicromonospora thailandica (AB560974)
Promicromonospora alba (KP784765)
Promicromonospora kermanensis (KJ780745)
Promicromonospora umidemergens (TYGS 2493)
Promicromonospora kroppenstedtii (TYGS 1068)
Promicromonospora sukumoe (AB023375)
Promicromonospora iranensis (JN038073)
Promicromonospora vindobonensis (AJ487302)
Promicromonospora aerolata (AJ487303)
Promicromonospora xylanilytica (FJ214352)
Promicromonospora endophytica (GU434253)
Xylanimonas cellulosilytica (AF403541)
Xylanibacterium ulmi (AY273185)
Xylanimicrobium pachnodae (TYGS 4457)
Isoptericola cucumis (KU201961)
Krasilnikoviella muralis (LC148843)
Krasilnikoviella flava (AM992980)
Isoptericola variabilis (AJ298873)
Isoptericola nanjingensis (HQ222356)
Isoptericola hypogeus (AJ854061)
Isoptericola halotolerans (AY789835)
Isoptericola chiayiensis (FJ469988)
Isoptericola jiangsuensis (TYGS 2540)
Isoptericola dokdonensis (TYGS 3189)
Sediminihabitans luteus (AB695376)
Tropicihabitans flavus (AB971840)
Paraoerskovia sediminicola (AB695378)
Paraoerskovia marina (TYGS 1820)
Koreibacter algae (TYGS 6226)
Oerskovia enterophila (TYGS 3909)
Oerskovia paurometabola (AJ314851)
Oerskovia jenensis (AJ314848)
Oerskovia turbata (X79454)
Cellulosimicrobium terreum (EF076760)
Cellulosimicrobium marinum (LC042213)
Cellulosimicrobium cellulans (TYGS 6070)
Brevibacterium lyticum (GU208866)
Cellulosimicrobium aquatile (TYGS 14269)
Cellulosimicrobium funkei (AY501364)
Oerskovia xanthineolytica (TYGS 4568)
Beutenbergia cavernae (TYGS 14)
Salana multivorans (AJ400627)
Serinibacter tropicus (AB915398)
Serinibacter salmoneus (TYGS 2524)
Miniimonas arenae (AB522642)
Ruania albidiflava (TYGS 1531)
Haloactinobacterium album (FJ969847)
Georgenia sediminis (JX555983)
Flaviflexus salsibiostraticola (KC251737)
Flaviflexus huanghaiensis (JN815236)
Actinobaculum massiliense (AF487679)
Actinobaculum suis (TYGS 4900)
Actinotignum sanguinis (HG798952)
Actinotignum schaalii (Y12329)
Actinotignum urinale (TYGS 1606)
Arcanobacterium phocae (FR749960)
Arcanobacterium phocisimile (FN562996)
Arcanobacterium hippocoleae (AJ300767)
Arcanobacterium canis (FR846134)
Arcanobacterium haemolyticum (TYGS 9)
Arcanobacterium pinnipediorum (KJ596349)
Arcanobacterium pluranimalium (AJ250959)
Trueperella bernardiae (TYGS 3139)
Trueperella abortisuis (AB305159)
Trueperella bonasi (AJ879697)
Trueperella bialowiezensis (AJ879696)
Trueperella pyogenes (X79225)
Actinomyces hordeovulneris (TYGS 14089)
Actinomyces nasicola (TYGS 14090)
Actinomyces urogenitalis (TYGS 2745)
Actinomyces denticolens (TYGS 17794)
Actinomyces timonensis (TYGS 5150)
Actinomyces johnsonii (AB545933)
Actinomyces oris (AB545935)
Actinomyces naeslundii (TYGS 5074)
Actinomyces viscosus (X82453)
Actinomyces slackii (TYGS 1635)
Actinomyces bowdenii (AJ234039)
Actinomyces oricola (AJ507295)
Actinomyces dentalis (TYGS 1439)
Actinomyces ruminicola (TYGS 5832)
Actinomyces gerencseriae (TYGS 1483)
Actinomyces israelii (TYGS 1867)
Actinomyces massiliensis (TYGS 5130)
Actinomyces howellii (X80411)
Actinomyces bovis (X81061)
Actinomyces weissii (FN552454)
Actinomyces catuli (AJ276805)
Actinomyces haliotis (KC196072)
Actinomyces radicidentis (TYGS 5418)
Actinomyces graevenitzii (AJ540309)
Actinomyces europaeus (Y08828)
Actinomyces hominis (FJ617539)
Actinomyces coleocanis (AJ249326)
Actinomyces canis (AJ243891)
Actinomyces naturae (FJ234421)
Actinomyces radingae (TYGS 2542)
Actinomyces hyovaginalis (X69616)
Actinomyces funkei (AJ404889)
Actinomyces cardiffensis (AJ421779)
Actinomyces vaccimaxillae (TYGS 1389)
Actinomyces odontolyticus (AJ234040)
Actinomyces meyeri (TYGS 2549)
Actinomyces georgiae (TYGS 1366)
Actinomyces turicensis (X78720)
Actinomyces suimastitidis (TYGS 1376)
Mobiluncus mulieris (TYGS 881)
Falcivibrio vaginalis (TYGS 142)
Mobiluncus curtisii subsp. holmesii (AJ427624)
Mobiluncus curtisii (TYGS 6064)
Actinomyces neuii subsp. anitratus (AM084229)
Actinomyces neuii subsp. neuii (TYGS 1510)
Varibaculum anthropi (JQ780830)
Varibaculum cambriense (TYGS 1602)

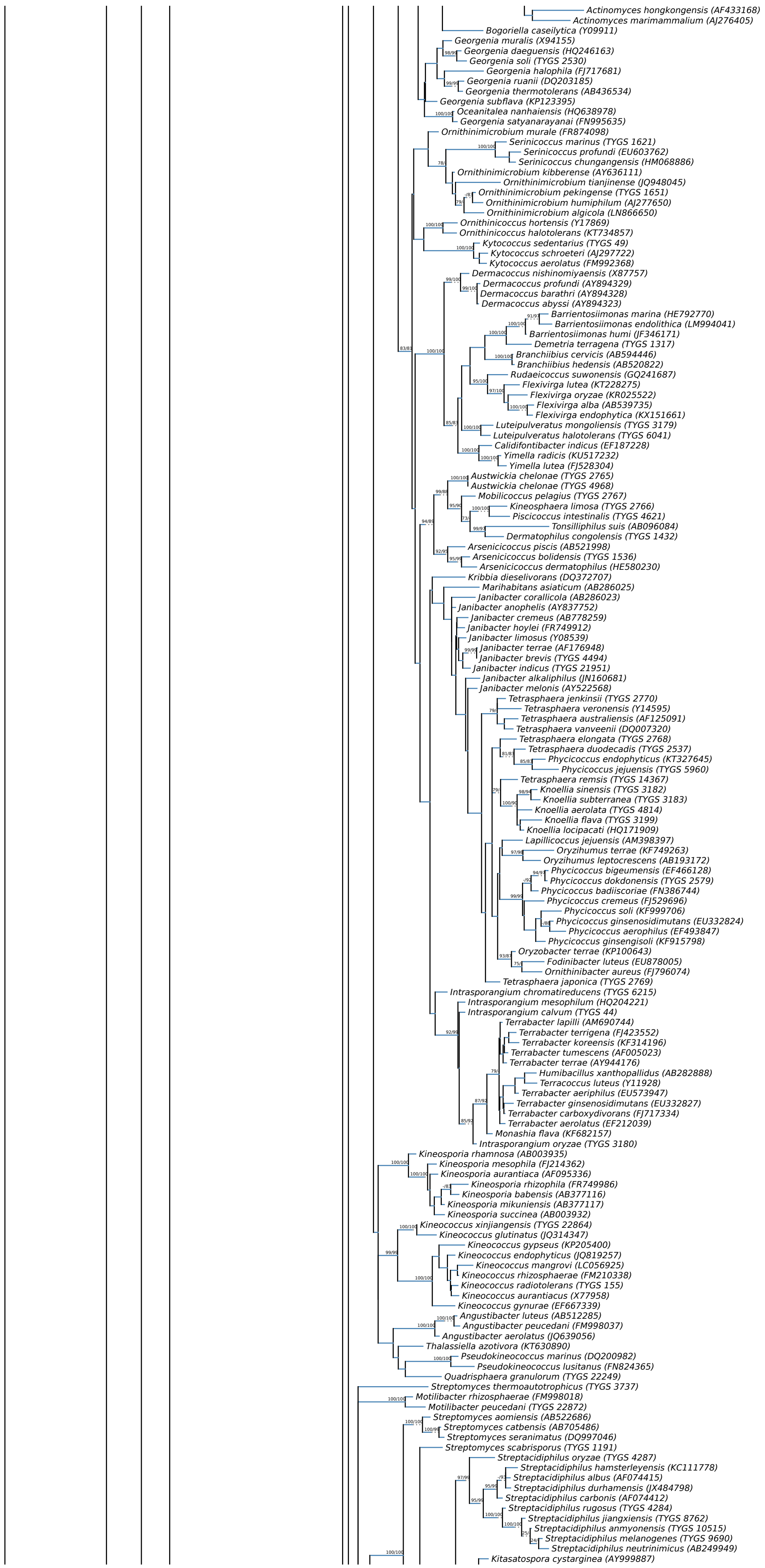
Actinomyces hongkongensis (AF433168)
Actinomyces marimammalium (AJ276405)
Bogoriella caseilytica (Y09911)
Georgenia muralis (X94155)
Georgenia daeguensis (HQ246163)
Georgenia soli (TYGS 2530)
Georgenia halophila (FJ717681)
Georgenia ruanii (DQ203185)
Georgenia thermotolerans (AB436534)
Georgenia subflava (KP123395)
Oceanitalea nanhaiensis (HQ638978)
Georgenia satyanarayanai (FN995635)
Ornithinimicrobium murale (FR874098)
Serinicoccus marinus (TYGS 1621)
Serinicoccus profundi (EU603762)
Serinicoccus chungangensis (HM068886)
Ornithinimicrobium kibberense (AY636111)
Ornithinimicrobium tianjinense (JQ948045)
Ornithinimicrobium pekingense (TYGS 1651)
Ornithinimicrobium humiphilum (AJ277650)
Ornithinimicrobium algicola (LN866650)
Ornithinicoccus hortensis (Y17869)
Ornithinicoccus halotolerans (KT734857)
Kytococcus sedentarius (TYGS 49)
Kytococcus schroeteri (AJ297722)
Kytococcus aerolatus (FM992368)
Dermacoccus nishinomiyaensis (X87757)
Dermacoccus profundi (AY894329)
Dermacoccus barathri (AY894328)
Dermacoccus abyssi (AY894323)
Barrientosiimonas marina (HE792770)
Barrientosiimonas endolithica (LM994041)
Barrientosiimonas humi (JF346171)
Demetria terragena (TYGS 1317)
Branchiibius cervicis (AB594446)
Branchiibius hedensis (AB520822)
Rudaeicoccus suwonensis (GQ241687)
Flexivirga lutea (KT228275)
Flexivirga oryzae (KR025522)
Flexivirga alba (AB539735)
Flexivirga endophytica (KX151661)
Luteipulveratus mongoliensis (TYGS 3179)
Luteipulveratus halotolerans (TYGS 6041)
Calidifontibacter indicus (EF187228)
Yimella radicis (KU517232)
Yimella lutea (FJ528304)
Austwickia chelonae (TYGS 2765)
Austwickia chelonae (TYGS 4968)
Mobilicoccus pelagius (TYGS 2767)
Kineosphaera limosa (TYGS 2766)
Piscicoccus intestinalis (TYGS 4621)
Tonsilliphilus suis (AB096084)
Dermatophilus congolensis (TYGS 1432)
Arsenicicoccus piscis (AB521998)
Arsenicicoccus bolidensis (TYGS 1536)
Arsenicicoccus dermatophilus (HE580230)
Kribbia dieselivorans (DQ372707)
Marihabitans asiaticum (AB286025)
Janibacter corallicola (AB286023)
Janibacter anophelis (AY837752)
Janibacter cremeus (AB778259)
Janibacter hoylei (FR749912)
Janibacter limosus (Y08539)
Janibacter terrae (AF176948)
Janibacter brevis (TYGS 4494)
Janibacter indicus (TYGS 21951)
Janibacter alkaliphilus (JN160681)
Janibacter melonis (AY522568)
Tetrasphaera jenkinsii (TYGS 2770)
Tetrasphaera veronensis (Y14595)
Tetrasphaera australiensis (AF125091)
Tetrasphaera vanveenii (DQ007320)
Tetrasphaera elongata (TYGS 2768)
Tetrasphaera duodecadis (TYGS 2537)
Phycicoccus endophyticus (KT327645)
Phycicoccus jejuensis (TYGS 5960)
Tetrasphaera remsis (TYGS 14367)
Knoellia sinensis (TYGS 3182)
Knoellia subterranea (TYGS 3183)
Knoellia aerolata (TYGS 4814)
Knoellia flava (TYGS 3199)
Knoellia locipacati (HQ171909)
Lapillicoccus jejuensis (AM398397)
Oryzihumus terrae (KF749263)
Oryzihumus leptocrescens (AB193172)
Phycicoccus bigeumensis (EF466128)
Phycicoccus dokdonensis (TYGS 2579)
Phycicoccus badiiscoriae (FN386744)
Phycicoccus cremeus (FJ529696)
Phycicoccus soli (KF999706)
Phycicoccus ginsenosidimutans (EU332824)
Phycicoccus aerophilus (EF493847)
Phycicoccus ginsengisoli (KF915798)
Oryzobacter terrae (KP100643)
Fodinibacter luteus (EU878005)
Ornithinibacter aureus (FJ796074)
Tetrasphaera japonica (TYGS 2769)
Intrasporangium chromatireducens (TYGS 6215)
Intrasporangium mesophilum (HQ204221)
Intrasporangium calvum (TYGS 44)
Terrabacter lapilli (AM690744)
Terrabacter terrigena (FJ423552)
Terrabacter koreensis (KF314196)
Terrabacter tumescens (AF005023)
Terrabacter terrae (AY944176)
Humibacillus xanthopallidus (AB282888)
Terracoccus luteus (Y11928)
Terrabacter aeriphilus (EU573947)
Terrabacter ginsenosidimutans (EU332827)
Terrabacter carboxydivorans (FJ717334)
Terrabacter aerolatus (EF212039)
Monashia flava (KF682157)
Intrasporangium oryzae (TYGS 3180)
Kineosporia rhamnosa (AB003935)
Kineosporia mesophila (FJ214362)
Kineosporia aurantiaca (AF095336)
Kineosporia rhizophila (FR749986)
Kineosporia babensis (AB377116)
Kineosporia mikuniensis (AB377117)
Kineosporia succinea (AB003932)
Kineococcus xinjiangensis (TYGS 22864)
Kineococcus glutinatus (JQ314347)
Kineococcus gypseus (KP205400)
Kineococcus endophyticus (JQ819257)
Kineococcus mangrovi (LC056925)
Kineococcus rhizosphaerae (FM210338)
Kineococcus radiotolerans (TYGS 155)
Kineococcus aurantiacus (X77958)
Kineococcus gynurae (EF667339)
Angustibacter luteus (AB512285)
Angustibacter peucedani (FM998037)
Angustibacter aerolatus (JQ639056)
Thalassiella azotivora (KT630890)
Pseudokineococcus marinus (DQ200982)
Pseudokineococcus lusitanus (FN824365)
Quadrisphaera granulorum (TYGS 22249)
Streptomyces thermoautotrophicus (TYGS 3737)
Motilibacter rhizosphaerae (FM998018)
Motilibacter peucedani (TYGS 22872)
Streptomyces aomiensis (AB522686)
Streptomyces catbensis (AB705486)
Streptomyces seranimatus (DQ997046)
Streptomyces scabrisporus (TYGS 1191)
Streptacidiphilus oryzae (TYGS 4287)
Streptacidiphilus hamsterleyensis (KC111778)
Streptacidiphilus albus (AF074415)
Streptacidiphilus durhamensis (JX484798)
Streptacidiphilus carbonis (AF074412)
Streptacidiphilus rugosus (TYGS 4284)
Streptacidiphilus jiangxiensis (TYGS 8762)
Streptacidiphilus anmyonensis (TYGS 10515)
Streptacidiphilus melanogenes (TYGS 9690)
Streptacidiphilus neutrinimicus (AB249949)
Kitasatospora cystarginea (AY999887)

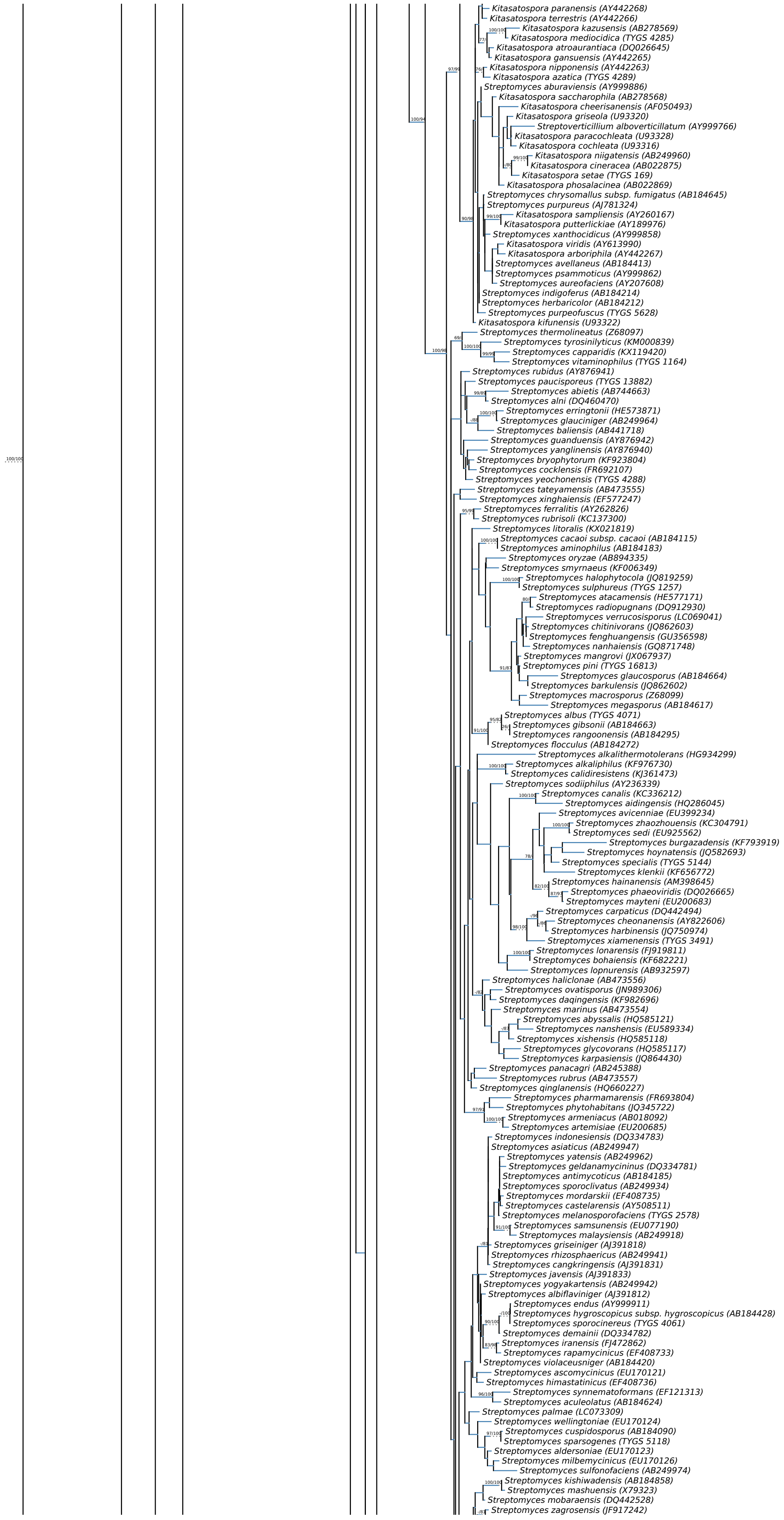
Kitasatospora paranensis (AY442268)
Kitasatospora terrestris (AY442266)
Kitasatospora kazusensis (AB278569)
Kitasatospora mediocidica (TYGS 4285)
Kitasatospora atroaurantiaca (DQ026645)
Kitasatospora gansuensis (AY442265)
Kitasatospora nipponensis (AY442263)
Kitasatospora azatica (TYGS 4289)
Streptomyces aburaviensis (AY999886)
Kitasatospora saccharophila (AB278568)
Kitasatospora cheerisanensis (AF050493)
Kitasatospora griseola (U93320)
Streptoverticillium alboverticillatum (AY999766)
Kitasatospora paracochleata (U93328)
Kitasatospora cochleata (U93316)
Kitasatospora niigatensis (AB249960)
Kitasatospora cineracea (AB022875)
Kitasatospora setae (TYGS 169)
Kitasatospora phosalacinea (AB022869)
Streptomyces chrysomallus subsp. fumigatus (AB184645)
Streptomyces purpureus (AJ781324)
Kitasatospora sampliensis (AY260167)
Kitasatospora putterlickiae (AY189976)
Streptomyces xanthocidicus (AY999858)
Kitasatospora viridis (AY613990)
Kitasatospora arboriphila (AY442267)
Streptomyces avellaneus (AB184413)
Streptomyces psammoticus (AY999862)
Streptomyces aureofaciens (AY207608)
Streptomyces indigoferus (AB184214)
Streptomyces herbaricolor (AB184212)
Streptomyces purpeofuscus (TYGS 5628)
Kitasatospora kifunensis (U93322)
Streptomyces thermolineatus (Z68097)
Streptomyces tyrosinilyticus (KM000839)
Streptomyces capparidis (KX119420)
Streptomyces vitaminophilus (TYGS 1164)
Streptomyces rubidus (AY876941)
Streptomyces paucisporeus (TYGS 13882)
Streptomyces abietis (AB744663)
Streptomyces alni (DQ460470)
Streptomyces erringtonii (HE573871)
Streptomyces glauciniger (AB249964)
Streptomyces baliensis (AB441718)
Streptomyces guanduensis (AY876942)
Streptomyces yanglinensis (AY876940)
Streptomyces bryophytorum (KF923804)
Streptomyces cocklensis (FR692107)
Streptomyces yeochonensis (TYGS 4288)
Streptomyces tateyamensis (AB473555)
Streptomyces xinghaiensis (EF577247)
Streptomyces ferralitis (AY262826)
Streptomyces rubrisoli (KC137300)
Streptomyces litoralis (KX021819)
Streptomyces cacaoi subsp. cacaoi (AB184115)
Streptomyces aminophilus (AB184183)
Streptomyces oryzae (AB894335)
Streptomyces smyrnaeus (KF006349)
Streptomyces halophytocola (JQ819259)
Streptomyces sulphureus (TYGS 1257)
Streptomyces atacamensis (HE577171)
Streptomyces radiopugnans (DQ912930)
Streptomyces verrucosisporus (LC069041)
Streptomyces chitinivorans (JQ862603)
Streptomyces fenghuangensis (GU356598)
Streptomyces nanhaiensis (GQ871748)
Streptomyces mangrovi (JX067937)
Streptomyces pini (TYGS 16813)
Streptomyces glaucosporus (AB184664)
Streptomyces barkulensis (JQ862602)
Streptomyces macrosporus (Z68099)
Streptomyces megasporus (AB184617)
Streptomyces albus (TYGS 4071)
Streptomyces gibsonii (AB184663)
Streptomyces rangoonensis (AB184295)
Streptomyces flocculus (AB184272)
Streptomyces alkalithermotolerans (HG934299)
Streptomyces alkaliphilus (KF976730)
Streptomyces calidiresistens (KJ361473)
Streptomyces sodiiphilus (AY236339)
Streptomyces canalis (KC336212)
Streptomyces aidingensis (HQ286045)
Streptomyces avicenniae (EU399234)
Streptomyces zhaozhouensis (KC304791)
Streptomyces sedi (EU925562)
Streptomyces burgazadensis (KF793919)
Streptomyces hoynatensis (JQ582693)
Streptomyces specialis (TYGS 5144)
Streptomyces klenkii (KF656772)
Streptomyces hainanensis (AM398645)
Streptomyces phaeoviridis (DQ026665)
Streptomyces mayteni (EU200683)
Streptomyces carpaticus (DQ442494)
Streptomyces cheonanensis (AY822606)
Streptomyces harbinensis (JQ750974)
Streptomyces xiamenensis (TYGS 3491)
Streptomyces lonarensis (FJ919811)
Streptomyces bohaiensis (KF682221)
Streptomyces lopnurensis (AB932597)
Streptomyces haliclonae (AB473556)
Streptomyces ovatisporus (JN989306)
Streptomyces daqingensis (KF982696)
Streptomyces marinus (AB473554)
Streptomyces abyssalis (HQ585121)
Streptomyces nanshensis (EU589334)
Streptomyces xishensis (HQ585118)
Streptomyces glycovorans (HQ585117)
Streptomyces karpasiensis (JQ864430)
Streptomyces panacagri (AB245388)
Streptomyces rubrus (AB473557)
Streptomyces qinglanensis (HQ660227)
Streptomyces pharmamarensis (FR693804)
Streptomyces phytohabitans (JQ345722)
Streptomyces armeniacus (AB018092)
Streptomyces artemisiae (EU200685)
Streptomyces indonesiensis (DQ334783)
Streptomyces asiaticus (AB249947)
Streptomyces yatensis (AB249962)
Streptomyces geldanamycininus (DQ334781)
Streptomyces antimycoticus (AB184185)
Streptomyces sporoclivatus (AB249934)
Streptomyces mordarskii (EF408735)
Streptomyces castelarensis (AY508511)
Streptomyces melanosporofaciens (TYGS 2578)
Streptomyces samsunensis (EU077190)
Streptomyces malaysiensis (AB249918)
Streptomyces griseiniger (AJ391818)
Streptomyces rhizosphaericus (AB249941)
Streptomyces cangkringensis (AJ391831)
Streptomyces javensis (AJ391833)
Streptomyces yogyakartensis (AB249942)
Streptomyces albiflaviniger (AJ391812)
Streptomyces endus (AY999911)
Streptomyces hygroscopicus subsp. hygroscopicus (AB184428)
Streptomyces sporocinereus (TYGS 4061)
Streptomyces demainii (DQ334782)
Streptomyces iranensis (FJ472862)
Streptomyces rapamycinicus (EF408733)
Streptomyces violaceusniger (AB184420)
Streptomyces ascomycinicus (EU170121)
Streptomyces himastatinicus (EF408736)
Streptomyces synnematoformans (EF121313)
Streptomyces aculeolatus (AB184624)
Streptomyces palmae (LC073309)
Streptomyces wellingtoniae (EU170124)
Streptomyces cuspidosporus (AB184090)
Streptomyces sparsogenes (TYGS 5118)
Streptomyces aldersoniae (EU170123)
Streptomyces milbemycinicus (EU170126)
Streptomyces sulfonofaciens (AB249974)
Streptomyces kishiwadensis (AB184858)
Streptomyces mashuensis (X79323)
Streptomyces mobaraensis (DQ442528)
Streptomyces zagrosensis (JF917242)

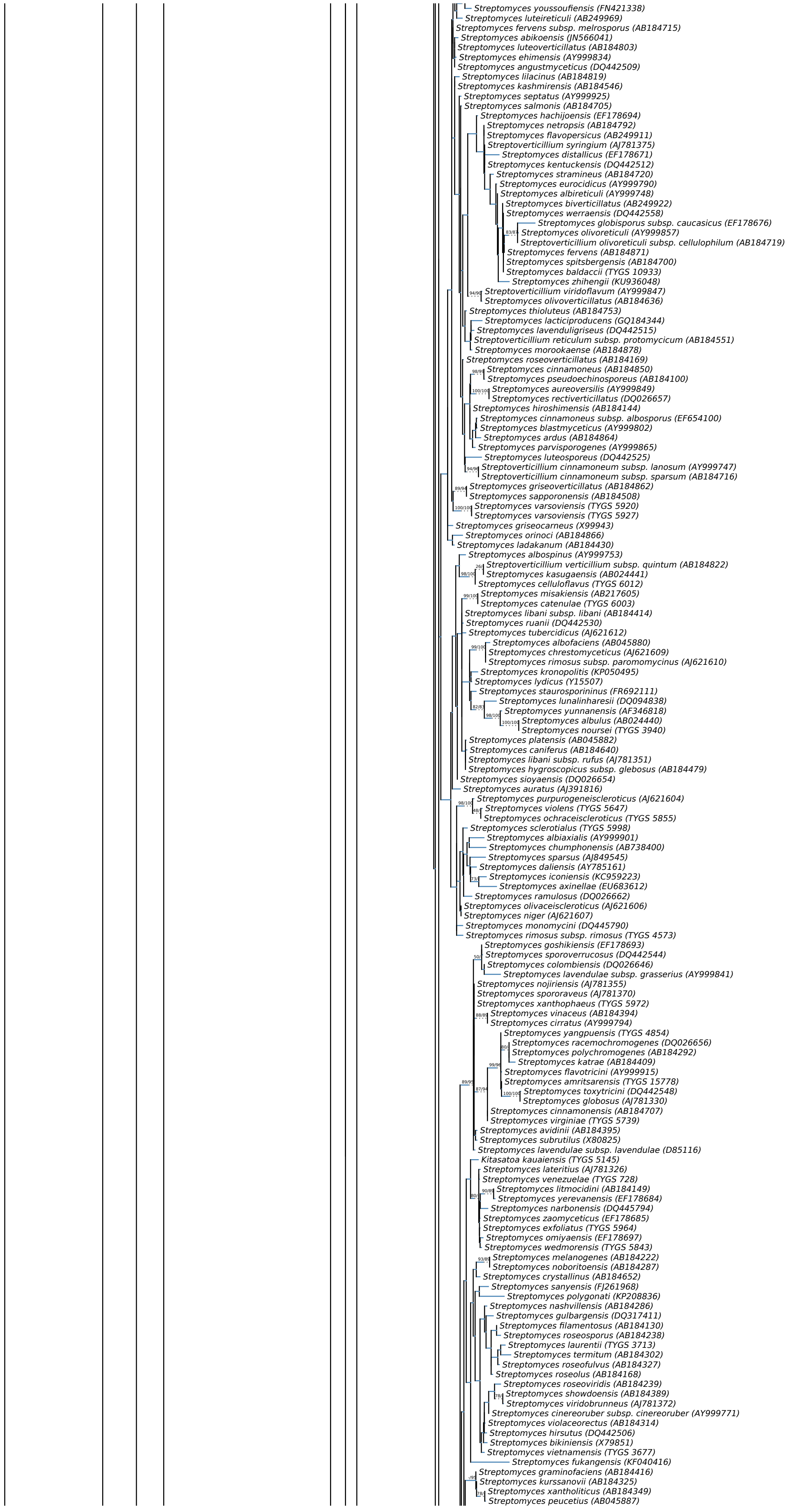
Streptomyces youssoufiensis (FN421338)
Streptomyces luteireticuli (AB249969)
Streptomyces fervens subsp. melrosporus (AB184715)
Streptomyces abikoensis (JN566041)
Streptomyces luteoverticillatus (AB184803)
Streptomyces ehimensis (AY999834)
Streptomyces angustmyceticus (DQ442509)
Streptomyces lilacinus (AB184819)
Streptomyces kashmirensis (AB184546)
Streptomyces septatus (AY999925)
Streptomyces salmonis (AB184705)
Streptomyces hachijoensis (EF178694)
Streptomyces netropsis (AB184792)
Streptomyces flavopersicus (AB249911)
Streptoverticillium syringium (AJ781375)
Streptomyces distallicus (EF178671)
Streptomyces kentuckensis (DQ442512)
Streptomyces stramineus (AB184720)
Streptomyces eurocidicus (AY999790)
Streptomyces albireticuli (AY999748)
Streptomyces biverticillatus (AB249922)
Streptomyces werraensis (DQ442558)
Streptomyces globisporus subsp. caucasicus (EF178676)
Streptomyces olivoreticuli (AY999857)
Streptoverticillium olivoreticuli subsp. cellulophilum (AB184719)
Streptomyces fervens (AB184871)
Streptomyces spitsbergensis (AB184700)
Streptomyces baldaccii (TYGS 10933)
Streptomyces zhihengii (KU936048)
Streptoverticillium viridoflavum (AY999847)
Streptomyces olivoverticillatus (AB184636)
Streptomyces thioluteus (AB184753)
Streptomyces lacticiproducens (GQ184344)
Streptomyces lavenduligriseus (DQ442515)
Streptoverticillium reticulum subsp. protomycicum (AB184551)
Streptomyces morookaense (AB184878)
Streptomyces roseoverticillatus (AB184169)
Streptomyces cinnamoneus (AB184850)
Streptomyces pseudoechinosporeus (AB184100)
Streptomyces aureoversilis (AY999849)
Streptomyces rectiverticillatus (DQ026657)
Streptomyces hiroshimensis (AB184144)
Streptomyces cinnamoneus subsp. albosporus (EF654100)
Streptomyces blastmyceticus (AY999802)
Streptomyces ardus (AB184864)
Streptomyces parvisporogenes (AY999865)
Streptomyces luteosporeus (DQ442525)
Streptoverticillium cinnamoneum subsp. lanosum (AY999747)
Streptoverticillium cinnamoneum subsp. sparsum (AB184716)
Streptomyces griseoverticillatus (AB184862)
Streptomyces sapporonensis (AB184508)
Streptomyces varsoviensis (TYGS 5920)
Streptomyces varsoviensis (TYGS 5927)
Streptomyces griseocarneus (X99943)
Streptomyces orinoci (AB184866)
Streptomyces ladakanum (AB184430)
Streptomyces albospinus (AY999753)
Streptoverticillium verticillium subsp. quintum (AB184822)
Streptomyces kasugaensis (AB024441)
Streptomyces celluloflavus (TYGS 6012)
Streptomyces misakiensis (AB217605)
Streptomyces catenulae (TYGS 6003)
Streptomyces libani subsp. libani (AB184414)
Streptomyces ruanii (DQ442530)
Streptomyces tubercidicus (AJ621612)
Streptomyces albofaciens (AB045880)
Streptomyces chrestomyceticus (AJ621609)
Streptomyces rimosus subsp. paromomycinus (AJ621610)
Streptomyces kronopolitis (KP050495)
Streptomyces lydicus (Y15507)
Streptomyces staurosporininus (FR692111)
Streptomyces lunalinharesii (DQ094838)
Streptomyces yunnanensis (AF346818)
Streptomyces albulus (AB024440)
Streptomyces noursei (TYGS 3940)
Streptomyces platensis (AB045882)
Streptomyces caniferus (AB184640)
Streptomyces libani subsp. rufus (AJ781351)
Streptomyces hygroscopicus subsp. glebosus (AB184479)
Streptomyces sioyaensis (DQ026654)
Streptomyces auratus (AJ391816)
Streptomyces purpurogeneiscleroticus (AJ621604)
Streptomyces violens (TYGS 5647)
Streptomyces ochraceiscleroticus (TYGS 5855)
Streptomyces sclerotialus (TYGS 5998)
Streptomyces albiaxialis (AY999901)
Streptomyces chumphonensis (AB738400)
Streptomyces sparsus (AJ849545)
Streptomyces daliensis (AY785161)
Streptomyces iconiensis (KC959223)
Streptomyces axinellae (EU683612)
Streptomyces ramulosus (DQ026662)
Streptomyces olivaceiscleroticus (AJ621606)
Streptomyces niger (AJ621607)
Streptomyces monomycini (DQ445790)
Streptomyces rimosus subsp. rimosus (TYGS 4573)
Streptomyces goshikiensis (EF178693)
Streptomyces sporoverrucosus (DQ442544)
Streptomyces colombiensis (DQ026646)
Streptomyces lavendulae subsp. grasserius (AY999841)
Streptomyces nojiriensis (AJ781355)
Streptomyces sporoaveus (AJ781370)
Streptomyces xanthophaeus (TYGS 5972)
Streptomyces vinaceus (AB184394)
Streptomyces cirratus (AY999794)
Streptomyces yangpuensis (TYGS 4854)
Streptomyces racemochromogenes (DQ026656)
Streptomyces polychromogenes (AB184292)
Streptomyces katrae (AB184409)
Streptomyces flavotricini (AY999915)
Streptomyces amritsarensis (TYGS 15778)
Streptomyces toxytricini (DQ442548)
Streptomyces globosus (AJ781330)
Streptomyces cinnamonensis (AB184707)
Streptomyces virginiae (TYGS 5739)
Streptomyces avidinii (AB184395)
Streptomyces subrutilus (X80825)
Streptomyces lavendulae subsp. lavendulae (D85116)
Kitasatoa kauaiensis (TYGS 5145)
Streptomyces lateritius (AJ781326)
Streptomyces venezuelae (TYGS 728)
Streptomyces litmocidini (AB184149)
Streptomyces yerevanensis (EF178684)
Streptomyces narbonensis (DQ445794)
Streptomyces zaomyceticus (EF178685)
Streptomyces exfoliatus (TYGS 5964)
Streptomyces omiyaensis (EF178697)
Streptomyces wedmorensis (TYGS 5843)
Streptomyces melanogenes (AB184222)
Streptomyces noboritoensis (AB184287)
Streptomyces crystallinus (AB184652)
Streptomyces sanyensis (FJ261968)
Streptomyces polygonati (KP208836)
Streptomyces nashvillensis (AB184286)
Streptomyces gulbargensis (DQ317411)
Streptomyces filamentosus (AB184130)
Streptomyces roseosporus (AB184238)
Streptomyces laurentii (TYGS 3713)
Streptomyces termitum (AB184302)
Streptomyces roseofulvus (AB184327)
Streptomyces roseolus (AB184168)
Streptomyces roseoviridis (AB184239)
Streptomyces showdoensis (AB184389)
Streptomyces viridobrunneus (AJ781372)
Streptomyces cinereoruber subsp. cinereoruber (AY999771)
Streptomyces violaceorectus (AB184314)
Streptomyces hirsutus (DQ442506)
Streptomyces bikiniensis (X79851)
Streptomyces vietnamensis (TYGS 3677)
Streptomyces fukangensis (KF040416)
Streptomyces graminofaciens (AB184416)
Streptomyces kurssanovii (AB184325)
Streptomyces xantholiticus (AB184349)
Streptomyces peucetius (AB045887)

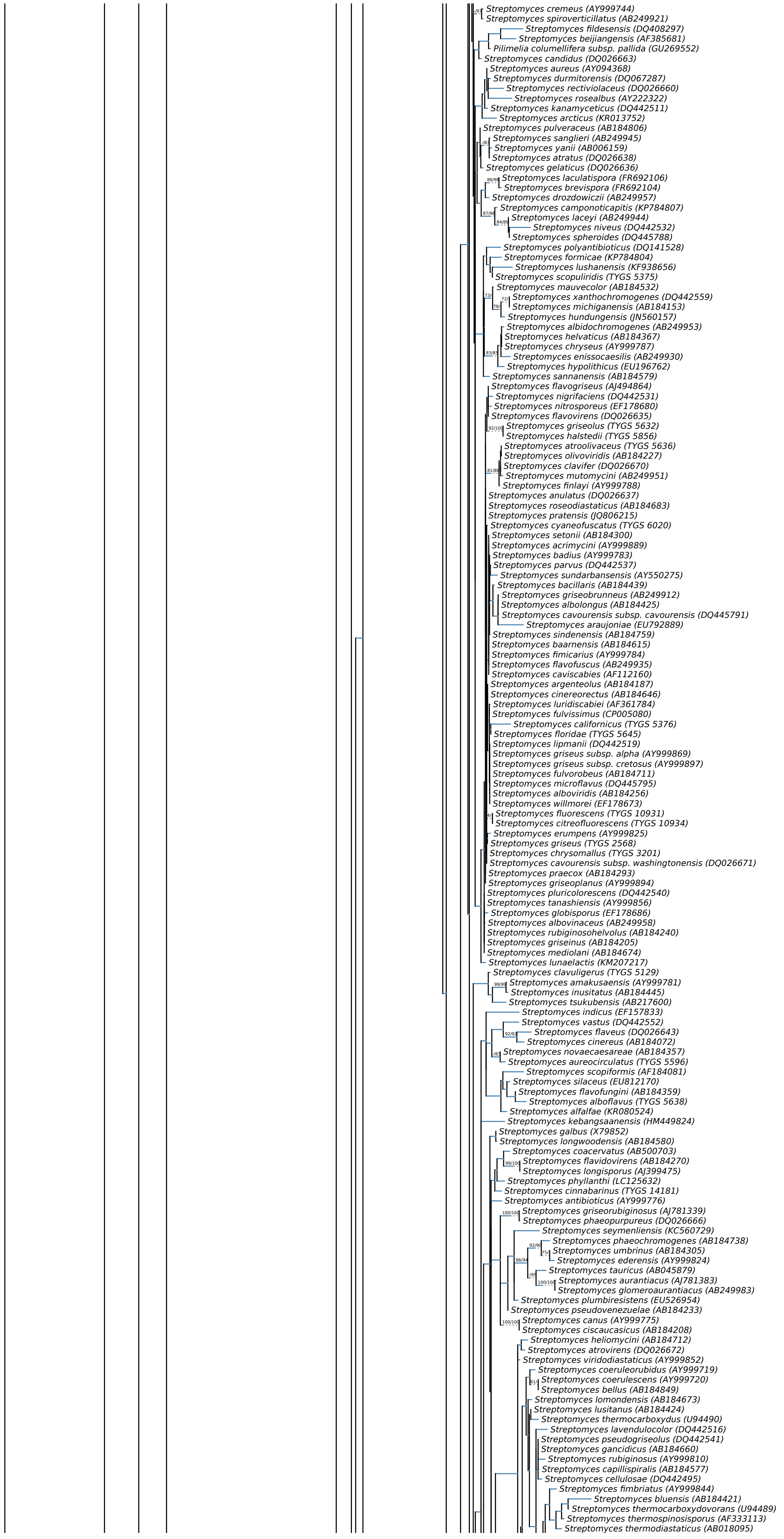

Streptomyces cremeus (AY999744)
Streptomyces spiroverticillatus (AB249921)
Streptomyces fildesensis (DQ408297)
Streptomyces beijiangensis (AF385681)
Pilimelia columellifera subsp. pallida (GU269552)
Streptomyces candidus (DQ026663)
Streptomyces aureus (AY094368)
Streptomyces durmitorensis (DQ067287)
Streptomyces rectiviolaceus (DQ026660)
Streptomyces rosealbus (AY222322)
Streptomyces kanamyceticus (DQ442511)
Streptomyces arcticus (KR013752)
Streptomyces pulveraceus (AB184806)
Streptomyces sanglieri (AB249945)
Streptomyces yanii (AB006159)
Streptomyces atratus (DQ026638)
Streptomyces gelaticus (DQ026636)
Streptomyces laculatispora (FR692106)
Streptomyces brevispora (FR692104)
Streptomyces drozdowiczii (AB249957)
Streptomyces camponoticapitis (KP784807)
Streptomyces laceyi (AB249944)
Streptomyces niveus (DQ442532)
Streptomyces spheroides (DQ445788)
Streptomyces polyantibioticus (DQ141528)
Streptomyces formicae (KP784804)
Streptomyces lushanensis (KF938656)
Streptomyces scopuliridis (TYGS 5375)
Streptomyces mauvecolor (AB184532)
Streptomyces xanthochromogenes (DQ442559)
Streptomyces michiganensis (AB184153)
Streptomyces hundungensis (JN560157)
Streptomyces albidochromogenes (AB249953)
Streptomyces helvaticus (AB184367)
Streptomyces chryseus (AY999787)
Streptomyces enissocaesilis (AB249930)
Streptomyces hypolithicus (EU196762)
Streptomyces sannanensis (AB184579)
Streptomyces flavogriseus (AJ494864)
Streptomyces nigrifaciens (DQ442531)
Streptomyces nitrosporeus (EF178680)
Streptomyces flavovirens (DQ026635)
Streptomyces griseolus (TYGS 5632)
Streptomyces halstedii (TYGS 5856)
Streptomyces atroolivaceus (TYGS 5636)
Streptomyces olivoviridis (AB184227)
Streptomyces clavifer (DQ026670)
Streptomyces mutomycini (AB249951)
Streptomyces finlayi (AY999788)
Streptomyces anulatus (DQ026637)
Streptomyces roseodiastaticus (AB184683)
Streptomyces pratensis (JQ806215)
Streptomyces cyaneofuscatus (TYGS 6020)
Streptomyces setonii (AB184300)
Streptomyces acrimycini (AY999889)
Streptomyces badius (AY999783)
Streptomyces parvus (DQ442537)
Streptomyces sundarbansensis (AY550275)
Streptomyces bacillaris (AB184439)
Streptomyces griseobrunneus (AB249912)
Streptomyces albolongus (AB184425)
Streptomyces cavourensis subsp. cavourensis (DQ445791)
Streptomyces araujoniae (EU792889)
Streptomyces sindenensis (AB184759)
Streptomyces baarnensis (AB184615)
Streptomyces fimicarius (AY999784)
Streptomyces flavofuscus (AB249935)
Streptomyces caviscabies (AF112160)
Streptomyces argenteolus (AB184187)
Streptomyces cinereorectus (AB184646)
Streptomyces luridiscabiei (AF361784)
Streptomyces fulvissimus (CP005080)
Streptomyces californicus (TYGS 5376)
Streptomyces floridae (TYGS 5645)
Streptomyces lipmanii (DQ442519)
Streptomyces griseus subsp. alpha (AY999869)
Streptomyces griseus subsp. cretosus (AY999897)
Streptomyces fulvorobeus (AB184711)
Streptomyces microflavus (DQ445795)
Streptomyces alboviridis (AB184256)
Streptomyces willmorei (EF178673)
Streptomyces fluorescens (TYGS 10931)
Streptomyces citreofluorescens (TYGS 10934)
Streptomyces erumpens (AY999825)
Streptomyces griseus (TYGS 2568)
Streptomyces chrysomallus (TYGS 3201)
Streptomyces cavourensis subsp. washingtonensis (DQ026671)
Streptomyces praecox (AB184293)
Streptomyces griseoplanus (AY999894)
Streptomyces pluricolorescens (DQ442540)
Streptomyces tanashiensis (AY999856)
Streptomyces globisporus (EF178686)
Streptomyces albovinaceus (AB249958)
Streptomyces rubiginosohelvolus (AB184240)
Streptomyces griseinus (AB184205)
Streptomyces mediolani (AB184674)
Streptomyces lunaelactis (KM207217)
Streptomyces clavuligerus (TYGS 5129)
Streptomyces amakusaensis (AY999781)
Streptomyces inusitatus (AB184445)
Streptomyces tsukubensis (AB217600)
Streptomyces indicus (EF157833)
Streptomyces vastus (DQ442552)
Streptomyces flaveus (DQ026643)
Streptomyces cinereus (AB184072)
Streptomyces novaecaesareae (AB184357)
Streptomyces aureocirculatus (TYGS 5596)
Streptomyces scopiformis (AF184081)
Streptomyces silaceus (EU812170)
Streptomyces flavofungini (AB184359)
Streptomyces alboflavus (TYGS 5638)
Streptomyces alfalfae (KR080524)
Streptomyces kebangsaanensis (HM449824)
Streptomyces galbus (X79852)
Streptomyces longwoodensis (AB184580)
Streptomyces coacervatus (AB500703)
Streptomyces flavidovirens (AB184270)
Streptomyces longisporus (AJ399475)
Streptomyces phyllanthi (LC125632)
Streptomyces cinnabarinus (TYGS 14181)
Streptomyces antibioticus (AY999776)
Streptomyces griseorubiginosus (AJ781339)
Streptomyces phaeopurpureus (DQ026666)
Streptomyces seymenliensis (KC560729)
Streptomyces phaeochromogenes (AB184738)
Streptomyces umbrinus (AB184305)
Streptomyces ederensis (AY999824)
Streptomyces tauricus (AB045879)
Streptomyces aurantiacus (AJ781383)
Streptomyces glomeroaurantiacus (AB249983)
Streptomyces plumbiresistens (EU526954)
Streptomyces pseudovenezuelae (AB184233)
Streptomyces canus (AY999775)
Streptomyces ciscaucasicus (AB184208)
Streptomyces heliomycini (AB184712)
Streptomyces atrovirens (DQ026672)
Streptomyces viridodiastaticus (AY999852)
Streptomyces coeruleorubidus (AY999719)
Streptomyces coerulescens (AY999720)
Streptomyces bellus (AB184849)
Streptomyces lomondensis (AB184673)
Streptomyces lusitanus (AB184424)
Streptomyces thermocarboxydus (U94490)
Streptomyces lavendulocolor (DQ442516)
Streptomyces pseudogriseolus (DQ442541)
Streptomyces gancidicus (AB184660)
Streptomyces rubiginosus (AY999810)
Streptomyces capillispiralis (AB184577)
Streptomyces cellulosae (DQ442495)
Streptomyces fimbriatus (AY999844)
Streptomyces bluensis (AB184421)
Streptomyces thermocarboxydovorans (U94489)
Streptomyces thermospinosisporus (AF333113)
Streptomyces thermodiastaticus (AB018095)

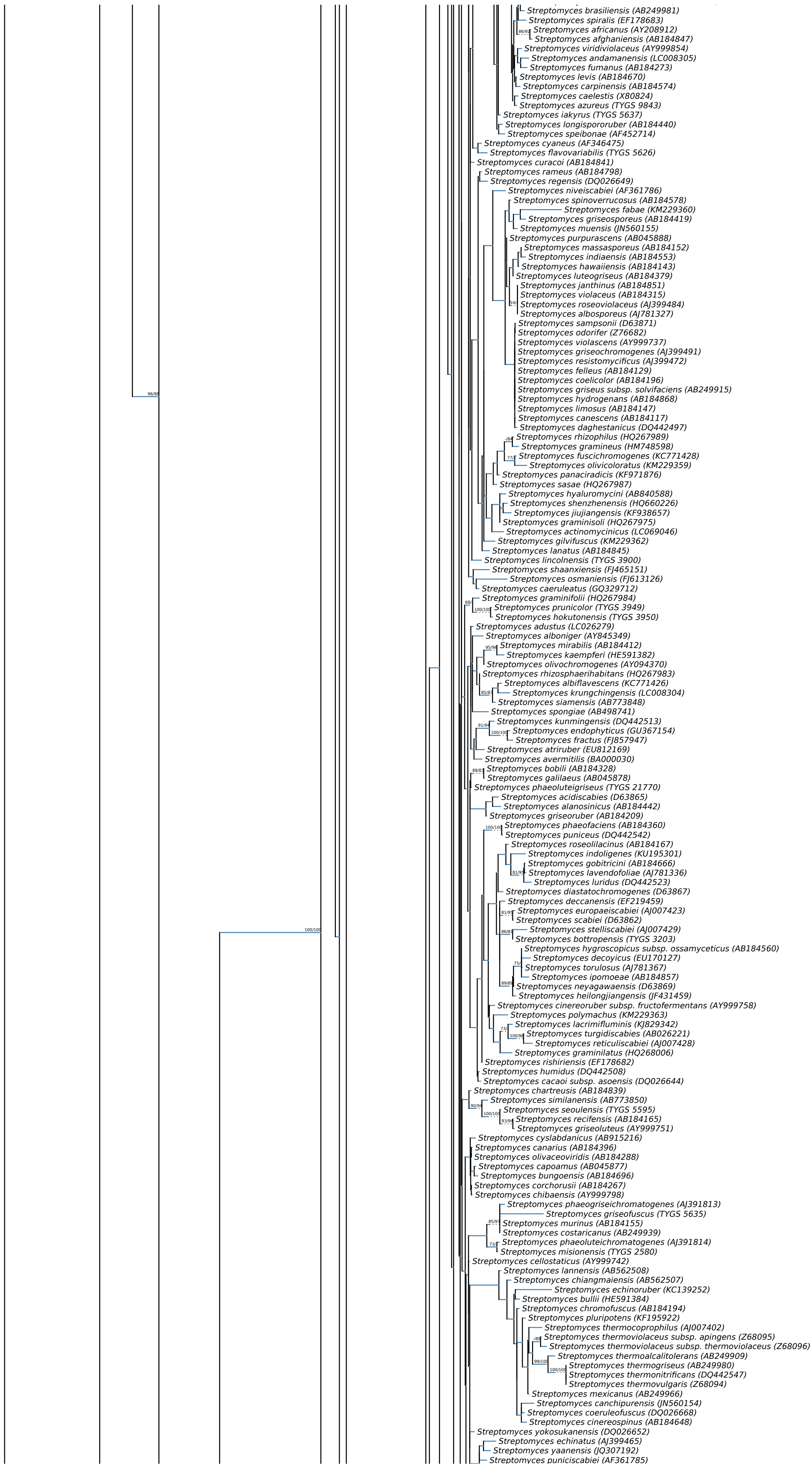

Streptomyces brasiliensis (AB249981)
Streptomyces spiralis (EF178683)
Streptomyces africanus (AY208912)
Streptomyces afghaniensis (AB184847)
Streptomyces viridiviolaceus (AY999854)
Streptomyces andamanensis (LC008305)
Streptomyces fumanus (AB184273)
Streptomyces levis (AB184670)
Streptomyces carpinensis (AB184574)
Streptomyces caelestis (X80824)
Streptomyces azureus (TYGS 9843)
Streptomyces iakyrus (TYGS 5637)
Streptomyces longispororuber (AB184440)
Streptomyces speibonae (AF452714)
Streptomyces cyaneus (AF346475)
Streptomyces flavovariabilis (TYGS 5626)
Streptomyces curacoi (AB184841)
Streptomyces rameus (AB184798)
Streptomyces regensis (DQ026649)
Streptomyces niveiscabiei (AF361786)
Streptomyces spinoverrucosus (AB184578)
Streptomyces fabae (KM229360)
Streptomyces griseosporeus (AB184419)
Streptomyces muensis (JN560155)
Streptomyces purpurascens (AB045888)
Streptomyces massasporeus (AB184152)
Streptomyces indiaensis (AB184553)
Streptomyces hawaiiensis (AB184143)
Streptomyces luteogriseus (AB184379)
Streptomyces janthinus (AB184851)
Streptomyces violaceus (AB184315)
Streptomyces roseoviolaceus (AJ399484)
Streptomyces albosporeus (AJ781327)
Streptomyces sampsonii (D63871)
Streptomyces odorifer (Z76682)
Streptomyces violascens (AY999737)
Streptomyces griseochromogenes (AJ399491)
Streptomyces resistomycificus (AJ399472)
Streptomyces felleus (AB184129)
Streptomyces coelicolor (AB184196)
Streptomyces griseus subsp. solvifaciens (AB249915)
Streptomyces hydrogenans (AB184868)
Streptomyces limosus (AB184147)
Streptomyces canescens (AB184117)
Streptomyces daghestanicus (DQ442497)
Streptomyces rhizophilus (HQ267989)
Streptomyces gramineus (HM748598)
Streptomyces fuscichromogenes (KC771428)
Streptomyces olivicoloratus (KM229359)
Streptomyces panaciradicis (KF971876)
Streptomyces sasae (HQ267987)
Streptomyces hyaluromycini (AB840588)
Streptomyces shenzhenensis (HQ660226)
Streptomyces jiujiangensis (KF938657)
Streptomyces graminisoli (HQ267975)
Streptomyces actinomycinicus (LC069046)
Streptomyces gilvifuscus (KM229362)
Streptomyces lanatus (AB184845)
Streptomyces lincolnensis (TYGS 3900)
Streptomyces shaanxiensis (FJ465151)
Streptomyces osmaniensis (FJ613126)
Streptomyces caeruleatus (GQ329712)
Streptomyces graminifolii (HQ267984)
Streptomyces prunicolor (TYGS 3949)
Streptomyces hokutonensis (TYGS 3950)
Streptomyces adustus (LC026279)
Streptomyces alboniger (AY845349)
Streptomyces mirabilis (AB184412)
Streptomyces kaempferi (HE591382)
Streptomyces olivochromogenes (AY094370)
Streptomyces rhizosphaerihabitans (HQ267983)
Streptomyces albiflavescens (KC771426)
Streptomyces krungchingensis (LC008304)
Streptomyces siamensis (AB773848)
Streptomyces spongiae (AB498741)
Streptomyces kunmingensis (DQ442513)
Streptomyces endophyticus (GU367154)
Streptomyces fractus (FJ857947)
Streptomyces atriruber (EU812169)
Streptomyces avermitilis (BA000030)
Streptomyces bobili (AB184328)
Streptomyces galilaeus (AB045878)
Streptomyces phaeoluteigriseus (TYGS 21770)
Streptomyces acidiscabies (D63865)
Streptomyces alanosinicus (AB184442)
Streptomyces griseoruber (AB184209)
Streptomyces phaeofaciens (AB184360)
Streptomyces puniceus (DQ442542)
Streptomyces roseolilacinus (AB184167)
Streptomyces indoligenes (KU195301)
Streptomyces gobitricini (AB184666)
Streptomyces lavendofoliae (AJ781336)
Streptomyces luridus (DQ442523)
Streptomyces diastatochromogenes (D63867)
Streptomyces deccanensis (EF219459)
Streptomyces europaeiscabiei (AJ007423)
Streptomyces scabiei (D63862)
Streptomyces stelliscabiei (AJ007429)
Streptomyces bottropensis (TYGS 3203)
Streptomyces hygroscopicus subsp. ossamyceticus (AB184560)
Streptomyces decoyicus (EU170127)
Streptomyces torulosus (AJ781367)
Streptomyces ipomoeae (AB184857)
Streptomyces neyagawaensis (D63869)
Streptomyces heilongjiangensis (JF431459)
Streptomyces cinereoruber subsp. fructofermentans (AY999758)
Streptomyces polymachus (KM229363)
Streptomyces lacrimifluminis (KJ829342)
Streptomyces turgidiscabies (AB026221)
Streptomyces reticuliscabiei (AJ007428)
Streptomyces graminilatus (HQ268006)
Streptomyces rishiriensis (EF178682)
Streptomyces humidus (DQ442508)
Streptomyces cacaoi subsp. asoensis (DQ026644)
Streptomyces chartreusis (AB184839)
Streptomyces similanensis (AB773850)
Streptomyces seoulensis (TYGS 5595)
Streptomyces recifensis (AB184165)
Streptomyces griseoluteus (AY999751)
Streptomyces cyslabdanicus (AB915216)
Streptomyces canarius (AB184396)
Streptomyces olivaceoviridis (AB184288)
Streptomyces capoamus (AB045877)
Streptomyces bungoensis (AB184696)
Streptomyces corchorusii (AB184267)
Streptomyces chibaensis (AY999798)
Streptomyces phaeogriseichromatogenes (AJ391813)
Streptomyces griseofuscus (TYGS 5635)
Streptomyces murinus (AB184155)
Streptomyces costaricanus (AB249939)
Streptomyces phaeoluteichromatogenes (AJ391814)
Streptomyces misionensis (TYGS 2580)
Streptomyces cellostaticus (AY999742)
Streptomyces lannensis (AB562508)
Streptomyces chiangmaiensis (AB562507)
Streptomyces echinoruber (KC139252)
Streptomyces bullii (HE591384)
Streptomyces chromofuscus (AB184194)
Streptomyces pluripotens (KF195922)
Streptomyces thermocoprophilus (AJ007402)
Streptomyces thermoviolaceus subsp. apingens (Z68095)
Streptomyces thermoviolaceus subsp. thermoviolaceus (Z68096)
Streptomyces thermoalcalitolerans (AB249909)
Streptomyces thermogriseus (AB249980)
Streptomyces thermonitrificans (DQ442547)
Streptomyces thermovulgaris (Z68094)
Streptomyces mexicanus (AB249966)
Streptomyces canchipurensis (JN560154)
Streptomyces coeruleofuscus (DQ026668)
Streptomyces cinereospinus (AB184648)
Streptomyces yokosukanensis (DQ026652)
Streptomyces echinatus (AJ399465)
Streptomyces yaanensis (JQ307192)
Streptomyces puniciscabiei (AF361785)

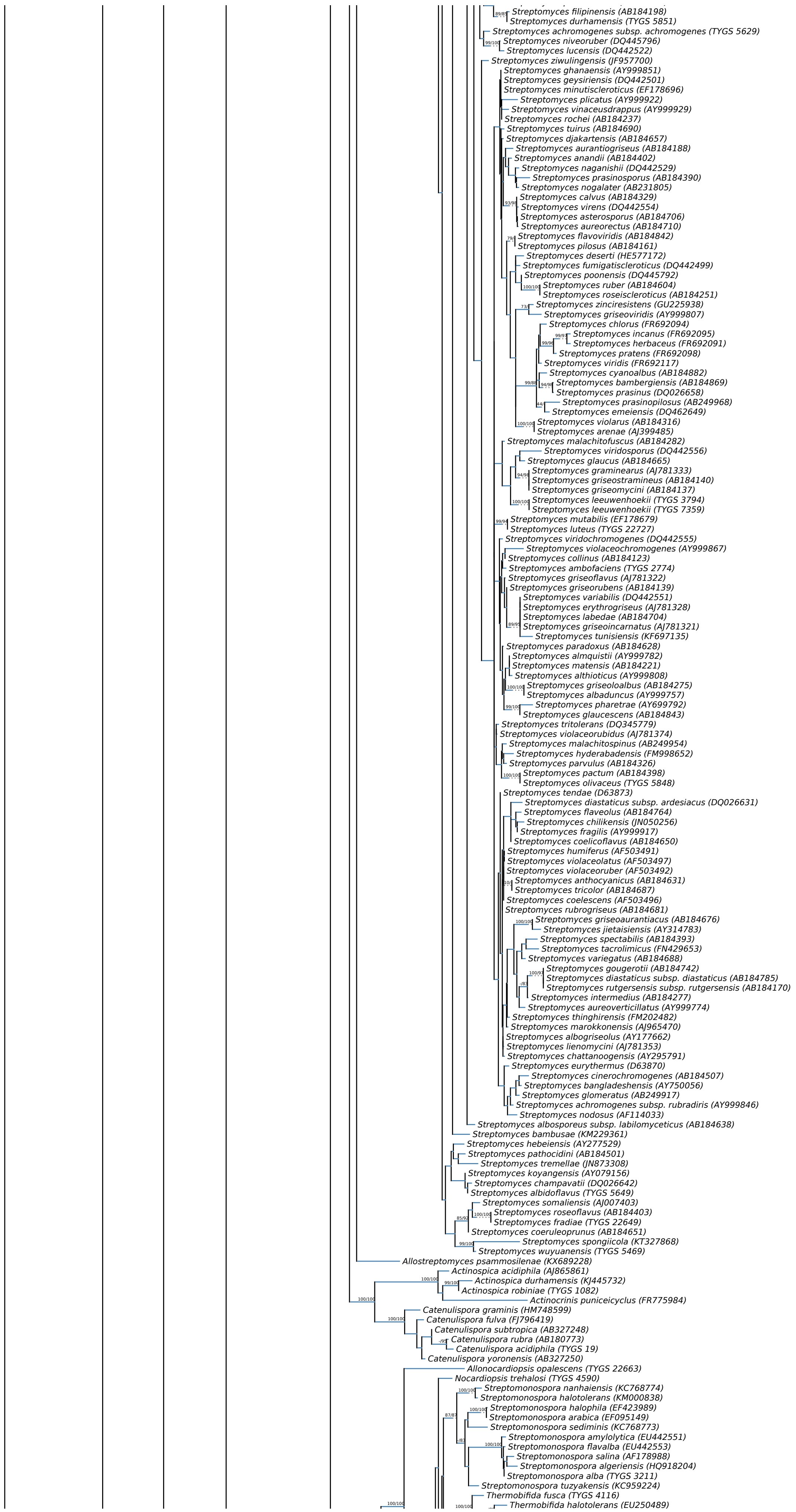

Streptomyces filipinensis (AB184198)
Streptomyces durhamensis (TYGS 5851)
Streptomyces achromogenes subsp. achromogenes (TYGS 5629)
Streptomyces niveoruber (DQ445796)
Streptomyces lucensis (DQ442522)
Streptomyces ziwulingensis (JF957700)
Streptomyces ghanaensis (AY999851)
Streptomyces geysiriensis (DQ442501)
Streptomyces minutiscleroticus (EF178696)
Streptomyces plicatus (AY999922)
Streptomyces vinaceusdrappus (AY999929)
Streptomyces rochei (AB184237)
Streptomyces tuirus (AB184690)
Streptomyces djakartensis (AB184657)
Streptomyces aurantiogriseus (AB184188)
Streptomyces anandii (AB184402)
Streptomyces naganishii (DQ442529)
Streptomyces prasinosporus (AB184390)
Streptomyces nogalater (AB231805)
Streptomyces calvus (AB184329)
Streptomyces virens (DQ442554)
Streptomyces asterosporus (AB184706)
Streptomyces aureorectus (AB184710)
Streptomyces flavoviridis (AB184842)
Streptomyces pilosus (AB184161)
Streptomyces deserti (HE577172)
Streptomyces fumigatiscleroticus (DQ442499)
Streptomyces poonensis (DQ445792)
Streptomyces ruber (AB184604)
Streptomyces roseiscleroticus (AB184251)
Streptomyces zinciresistens (GU225938)
Streptomyces griseoviridis (AY999807)
Streptomyces chlorus (FR692094)
Streptomyces incanus (FR692095)
Streptomyces herbaceus (FR692091)
Streptomyces pratens (FR692098)
Streptomyces viridis (FR692117)
Streptomyces cyanoalbus (AB184882)
Streptomyces bambergiensis (AB184869)
Streptomyces prasinus (DQ026658)
Streptomyces prasinopilosus (AB249968)
Streptomyces emeiensis (DQ462649)
Streptomyces violarus (AB184316)
Streptomyces arenae (AJ399485)
Streptomyces malachitofuscus (AB184282)
Streptomyces viridosporus (DQ442556)
Streptomyces glaucus (AB184665)
Streptomyces graminearus (AJ781333)
Streptomyces griseostramineus (AB184140)
Streptomyces griseomycini (AB184137)
Streptomyces leeuwenhoekii (TYGS 3794)
Streptomyces leeuwenhoekii (TYGS 7359)
Streptomyces mutabilis (EF178679)
Streptomyces luteus (TYGS 22727)
Streptomyces viridochromogenes (DQ442555)
Streptomyces violaceochromogenes (AY999867)
Streptomyces collinus (AB184123)
Streptomyces ambofaciens (TYGS 2774)
Streptomyces griseoflavus (AJ781322)
Streptomyces griseorubens (AB184139)
Streptomyces variabilis (DQ442551)
Streptomyces erythrogriseus (AJ781328)
Streptomyces labedae (AB184704)
Streptomyces griseoincarnatus (AJ781321)
Streptomyces tunisiensis (KF697135)
Streptomyces paradoxus (AB184628)
Streptomyces almquistii (AY999782)
Streptomyces matensis (AB184221)
Streptomyces althioticus (AY999808)
Streptomyces griseoloalbus (AB184275)
Streptomyces albaduncus (AY999757)
Streptomyces pharetrae (AY699792)
Streptomyces glaucescens (AB184843)
Streptomyces tritolerans (DQ345779)
Streptomyces violaceorubidus (AJ781374)
Streptomyces malachitospinus (AB249954)
Streptomyces hyderabadensis (FM998652)
Streptomyces parvulus (AB184326)
Streptomyces pactum (AB184398)
Streptomyces olivaceus (TYGS 5848)
Streptomyces tendae (D63873)
Streptomyces diastaticus subsp. ardesiacus (DQ026631)
Streptomyces flaveolus (AB184764)
Streptomyces chilikensis (JN050256)
Streptomyces fragilis (AY999917)
Streptomyces coelicoflavus (AB184650)
Streptomyces humiferus (AF503491)
Streptomyces violaceolatus (AF503497)
Streptomyces violaceoruber (AF503492)
Streptomyces anthocyanicus (AB184631)
Streptomyces tricolor (AB184687)
Streptomyces coelescens (AF503496)
Streptomyces rubrogriseus (AB184681)
Streptomyces griseoaurantiacus (AB184676)
Streptomyces jietaisiensis (AY314783)
Streptomyces spectabilis (AB184393)
Streptomyces tacrolimicus (FN429653)
Streptomyces variegatus (AB184688)
Streptomyces gougerotii (AB184742)
Streptomyces diastaticus subsp. diastaticus (AB184785)
Streptomyces rutgersensis subsp. rutgersensis (AB184170)
Streptomyces intermedius (AB184277)
Streptomyces aureoverticillatus (AY999774)
Streptomyces thinghirensis (FM202482)
Streptomyces marokkonensis (AJ965470)
Streptomyces albogriseolus (AY177662)
Streptomyces lienomycini (AJ781353)
Streptomyces chattanoogensis (AY295791)
Streptomyces eurythermus (D63870)
Streptomyces cinerochromogenes (AB184507)
Streptomyces bangladeshensis (AY750056)
Streptomyces glomeratus (AB249917)
Streptomyces achromogenes subsp. rubradiris (AY999846)
Streptomyces nodosus (AF114033)
Streptomyces albosporeus subsp. labilomyceticus (AB184638)
Streptomyces bambusae (KM229361)
Streptomyces hebeiensis (AY277529)
Streptomyces pathocidini (AB184501)
Streptomyces tremellae (JN873308)
Streptomyces koyangensis (AY079156)
Streptomyces champavatii (DQ026642)
Streptomyces albidoflavus (TYGS 5649)
Streptomyces somaliensis (AJ007403)
Streptomyces roseoflavus (AB184403)
Streptomyces fradiae (TYGS 22649)
Streptomyces coeruleoprunus (AB184651)
Streptomyces spongiicola (KT327868)
Streptomyces wuyuanensis (TYGS 5469)
Allostreptomyces psammosilenae (KX689228)
Actinospica acidiphila (AJ865861)
Actinospica durhamensis (KJ445732)
Actinospica robiniae (TYGS 1082)
Actinocrinis puniceicyclus (FR775984)
Catenulispora graminis (HM748599)
Catenulispora fulva (FJ796419)
Catenulispora subtropica (AB327248)
Catenulispora rubra (AB180773)
Catenulispora acidiphila (TYGS 19)
Catenulispora yoronensis (AB327250)
Allonocardiopsis opalescens (TYGS 22663)
Nocardiopsis trehalosi (TYGS 4590)
Streptomonospora nanhaiensis (KC768774)
Streptomonospora halotolerans (KM000838)
Streptomonospora halophila (EF423989)
Streptomonospora arabica (EF095149)
Streptomonospora sediminis (KC768773)
Streptomonospora amylolytica (EU442551)
Streptomonospora flavalba (EU442553)
Streptomonospora salina (AF178988)
Streptomonospora algeriensis (HQ918204)
Streptomonospora alba (TYGS 3211)
Streptomonospora tuzyakensis (KC959224)
Thermobifida fusca (TYGS 4116)
Thermobifida halotolerans (EU250489)

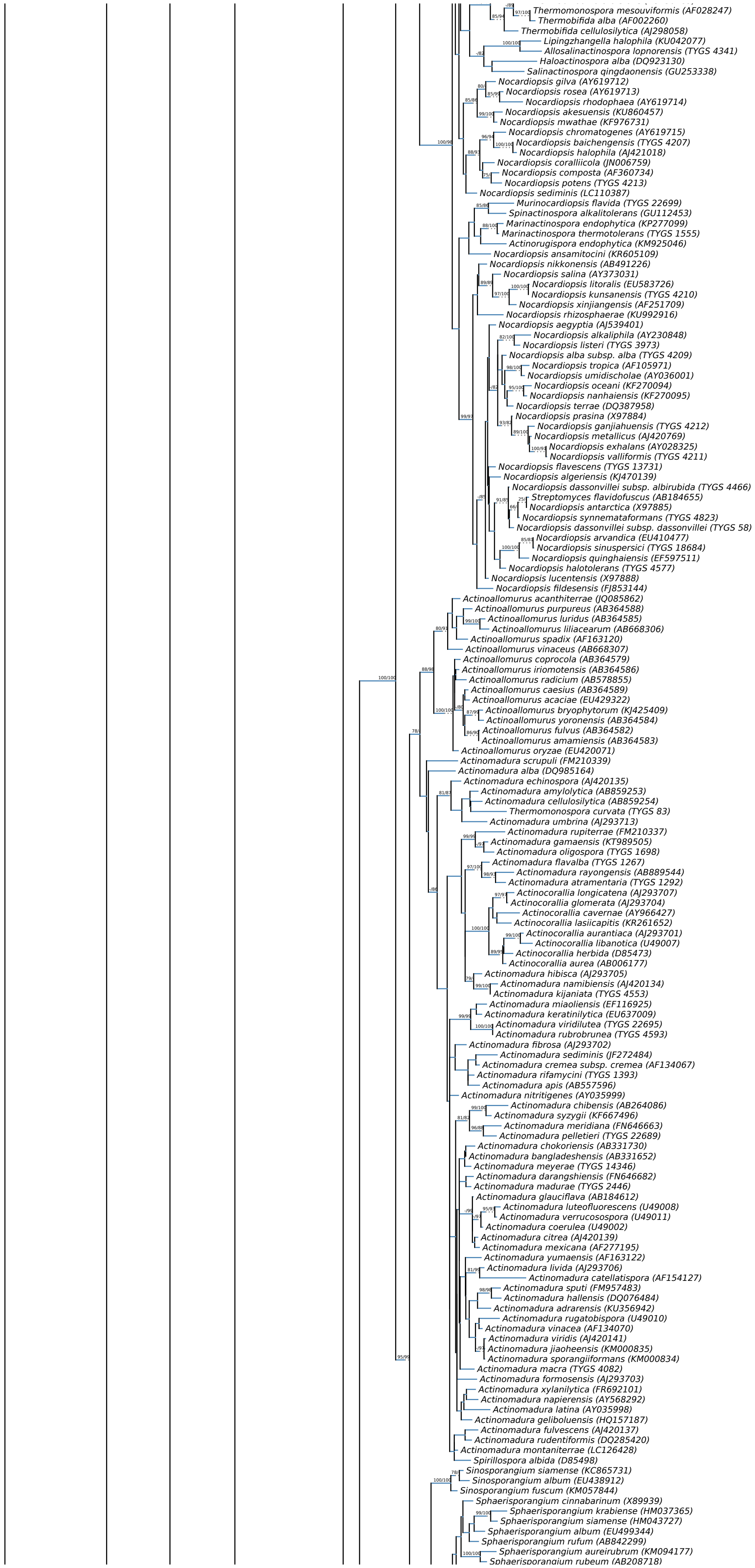
Thermomonospora mesouviformis (AF028247)
Thermobifida alba (AF002260)
Thermobifida cellulosilytica (AJ298058)
Lipingzhangella halophila (KU042077)
Allosalinactinospora lopnorensis (TYGS 4341)
Haloactinospora alba (DQ923130)
Salinactinospora qingdaonensis (GU253338)
Nocardiopsis gilva (AY619712)
Nocardiopsis rosea (AY619713)
Nocardiopsis rhodophaea (AY619714)
Nocardiopsis akesuensis (KU860457)
Nocardiopsis mwathae (KF976731)
Nocardiopsis chromatogenes (AY619715)
Nocardiopsis baichengensis (TYGS 4207)
Nocardiopsis halophila (AJ421018)
Nocardiopsis coralliicola (JN006759)
Nocardiopsis composta (AF360734)
Nocardiopsis potens (TYGS 4213)
Nocardiopsis sediminis (LC110387)
Murinocardiopsis flavida (TYGS 22699)
Spinactinospora alkalitolerans (GU112453)
Marinactinospora endophytica (KP277099)
Marinactinospora thermotolerans (TYGS 1555)
Actinorugispora endophytica (KM925046)
Nocardiopsis ansamitocini (KR605109)
Nocardiopsis nikkonensis (AB491226)
Nocardiopsis salina (AY373031)
Nocardiopsis litoralis (EU583726)
Nocardiopsis kunsanensis (TYGS 4210)
Nocardiopsis xinjiangensis (AF251709)
Nocardiopsis rhizosphaerae (KU992916)
Nocardiopsis aegyptia (AJ539401)
Nocardiopsis alkaliphila (AY230848)
Nocardiopsis listeri (TYGS 3973)
Nocardiopsis alba subsp. alba (TYGS 4209)
Nocardiopsis tropica (AF105971)
Nocardiopsis umidischolae (AY036001)
Nocardiopsis oceani (KF270094)
Nocardiopsis nanhaiensis (KF270095)
Nocardiopsis terrae (DQ387958)
Nocardiopsis prasina (X97884)
Nocardiopsis ganjiahuensis (TYGS 4212)
Nocardiopsis metallicus (AJ420769)
Nocardiopsis exhalans (AY028325)
Nocardiopsis valliformis (TYGS 4211)
Nocardiopsis flavescens (TYGS 13731)
Nocardiopsis algeriensis (KJ470139)
Nocardiopsis dassonvillei subsp. albirubida (TYGS 4466)
Streptomyces flavidofuscus (AB184655)
Nocardiopsis antarctica (X97885)
Nocardiopsis synnemataformans (TYGS 4823)
Nocardiopsis dassonvillei subsp. dassonvillei (TYGS 58)
Nocardiopsis arvandica (EU410477)
Nocardiopsis sinuspersici (TYGS 18684)
Nocardiopsis quinghaiensis (EF597511)
Nocardiopsis halotolerans (TYGS 4577)
Nocardiopsis lucentensis (X97888)
Nocardiopsis fildesensis (FJ853144)
Actinoallomurus acanthiterrae (JQ085862)
Actinoallomurus purpureus (AB364588)
Actinoallomurus luridus (AB364585)
Actinoallomurus liliacearum (AB668306)
Actinoallomurus spadix (AF163120)
Actinoallomurus vinaceus (AB668307)
Actinoallomurus coprocola (AB364579)
Actinoallomurus iriomotensis (AB364586)
Actinoallomurus radicium (AB578855)
Actinoallomurus caesius (AB364589)
Actinoallomurus acaciae (EU429322)
Actinoallomurus bryophytorum (KJ425409)
Actinoallomurus yoronensis (AB364584)
Actinoallomurus fulvus (AB364582)
Actinoallomurus amamiensis (AB364583)
Actinoallomurus oryzae (EU420071)
Actinomadura scrupuli (FM210339)
Actinomadura alba (DQ985164)
Actinomadura echinospora (AJ420135)
Actinomadura amylolytica (AB859253)
Actinomadura cellulosilytica (AB859254)
Thermomonospora curvata (TYGS 83)
Actinomadura umbrina (AJ293713)
Actinomadura rupiterrae (FM210337)
Actinomadura gamaensis (KT989505)
Actinomadura oligospora (TYGS 1698)
Actinomadura flavalba (TYGS 1267)
Actinomadura rayongensis (AB889544)
Actinomadura atramentaria (TYGS 1292)
Actinocorallia longicatena (AJ293707)
Actinocorallia glomerata (AJ293704)
Actinocorallia cavernae (AY966427)
Actinocorallia lasiicapitis (KR261652)
Actinocorallia aurantiaca (AJ293701)
Actinocorallia libanotica (U49007)
Actinocorallia herbida (D85473)
Actinocorallia aurea (AB006177)
Actinomadura hibisca (AJ293705)
Actinomadura namibiensis (AJ420134)
Actinomadura kijaniata (TYGS 4553)
Actinomadura miaoliensis (EF116925)
Actinomadura keratinilytica (EU637009)
Actinomadura viridilutea (TYGS 22695)
Actinomadura rubrobrunea (TYGS 4593)
Actinomadura fibrosa (AJ293702)
Actinomadura sediminis (JF272484)
Actinomadura cremea subsp. cremea (AF134067)
Actinomadura rifamycini (TYGS 1393)
Actinomadura apis (AB557596)
Actinomadura nitritigenes (AY035999)
Actinomadura chibensis (AB264086)
Actinomadura syzygii (KF667496)
Actinomadura meridiana (FN646663)
Actinomadura pelletieri (TYGS 22689)
Actinomadura chokoriensis (AB331730)
Actinomadura bangladeshensis (AB331652)
Actinomadura meyerae (TYGS 14346)
Actinomadura darangshiensis (FN646682)
Actinomadura madurae (TYGS 2446)
Actinomadura glauciflava (AB184612)
Actinomadura luteofluorescens (U49008)
Actinomadura verrucosospora (U49011)
Actinomadura coerulea (U49002)
Actinomadura citrea (AJ420139)
Actinomadura mexicana (AF277195)
Actinomadura yumaensis (AF163122)
Actinomadura livida (AJ293706)
Actinomadura catellatispora (AF154127)
Actinomadura sputi (FM957483)
Actinomadura hallensis (DQ076484)
Actinomadura adrarensis (KU356942)
Actinomadura rugatobispora (U49010)
Actinomadura vinacea (AF134070)
Actinomadura viridis (AJ420141)
Actinomadura jiaoheensis (KM000835)
Actinomadura sporangiiformans (KM000834)
Actinomadura macra (TYGS 4082)
Actinomadura formosensis (AJ293703)
Actinomadura xylanilytica (FR692101)
Actinomadura napierensis (AY568292)
Actinomadura latina (AY035998)
Actinomadura geliboluensis (HQ157187)
Actinomadura fulvescens (AJ420137)
Actinomadura rudentiformis (DQ285420)
Actinomadura montaniterrae (LC126428)
Spirillospora albida (D85498)
Sinosporangium siamense (KC865731)
Sinosporangium album (EU438912)
Sinosporangium fuscum (KM057844)
Sphaerisporangium cinnabarinum (X89939)
Sphaerisporangium krabiense (HM037365)
Sphaerisporangium siamense (HM043727)
Sphaerisporangium album (EU499344)
Sphaerisporangium rufum (AB842299)
Sphaerisporangium aureirubrum (KM094177)
Sphaerisporangium rubeum (AB208718)

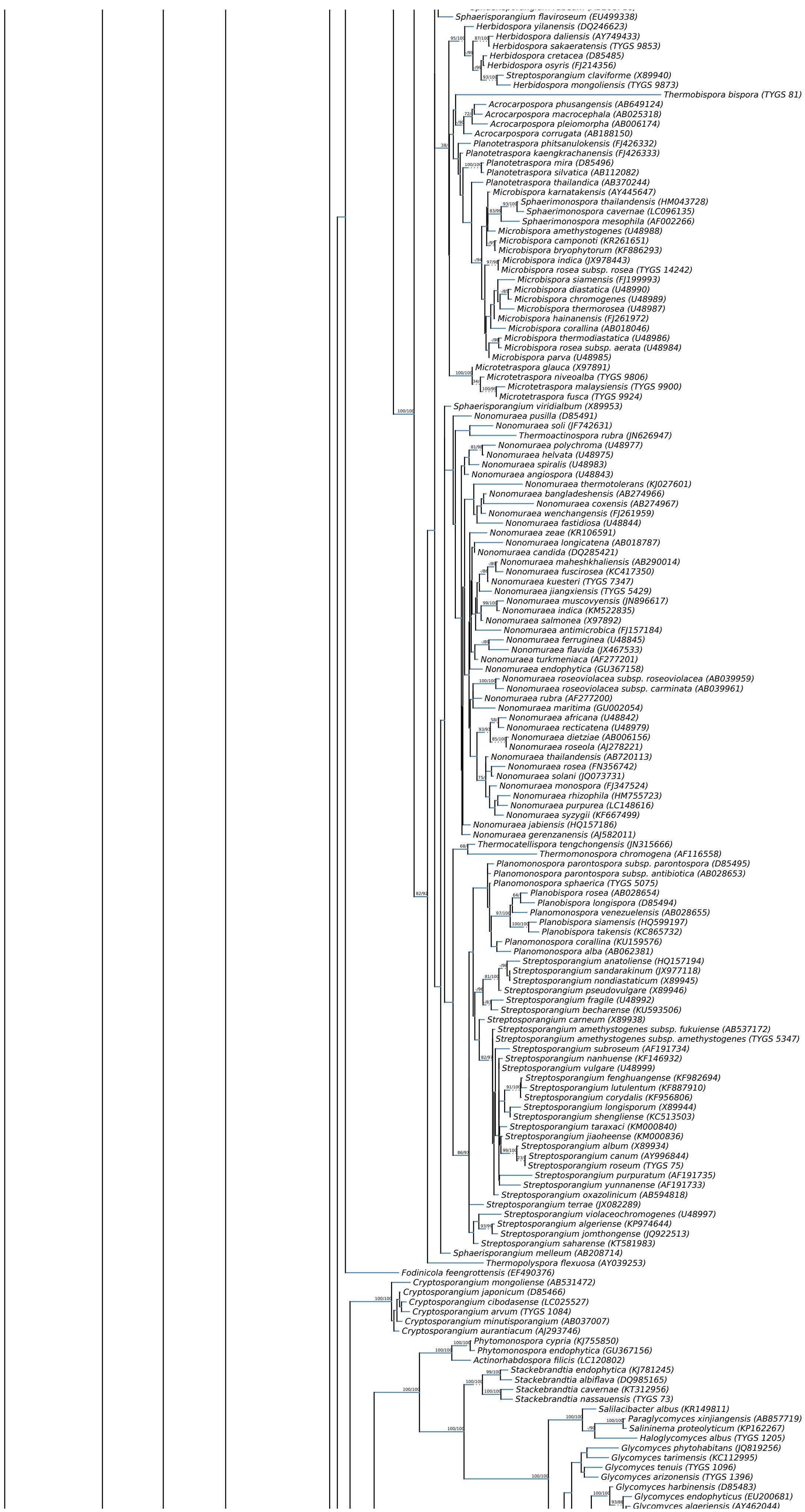
Sphaerisporangium flaviroseum (EU499338)
Herbidospora yilanensis (DQ246623)
Herbidospora daliensis (AY749433)
Herbidospora sakaeratensis (TYGS 9853)
Herbidospora cretacea (D85485)
Herbidospora osyris (FJ214356)
Streptosporangium claviforme (X89940)
Herbidospora mongoliensis (TYGS 9873)
Thermobispora bispora (TYGS 81)
Acrocarpospora phusangensis (AB649124)
Acrocarpospora macrocephala (AB025318)
Acrocarpospora pleiomorpha (AB006174)
Acrocarpospora corrugata (AB188150)
Planotetraspora phitsanulokensis (FJ426332)
Planotetraspora kaengkrachanensis (FJ426333)
Planotetraspora mira (D85496)
Planotetraspora silvatica (AB112082)
Planotetraspora thailandica (AB370244)
Microbispora karnatakensis (AY445647)
Sphaerimonospora thailandensis (HM043728)
Sphaerimonospora cavernae (LC096135)
Sphaerimonospora mesophila (AF002266)
Microbispora amethystogenes (U48988)
Microbispora camponoti (KR261651)
Microbispora bryophytorum (KF886293)
Microbispora indica (JX978443)
Microbispora rosea subsp. rosea (TYGS 14242)
Microbispora siamensis (FJ199993)
Microbispora diastatica (U48990)
Microbispora chromogenes (U48989)
Microbispora thermorosea (U48987)
Microbispora hainanensis (FJ261972)
Microbispora corallina (AB018046)
Microbispora thermodiastatica (U48986)
Microbispora rosea subsp. aerata (U48984)
Microbispora parva (U48985)
Microtetraspora glauca (X97891)
Microtetraspora niveoalba (TYGS 9806)
Microtetraspora malaysiensis (TYGS 9900)
Microtetraspora fusca (TYGS 9924)
Sphaerisporangium viridialbum (X89953)
Nonomuraea pusilla (D85491)
Nonomuraea soli (JF742631)
Thermoactinospora rubra (JN626947)
Nonomuraea polychroma (U48977)
Nonomuraea helvata (U48975)
Nonomuraea spiralis (U48983)
Nonomuraea angiospora (U48843)
Nonomuraea thermotolerans (KJ027601)
Nonomuraea bangladeshensis (AB274966)
Nonomuraea coxensis (AB274967)
Nonomuraea wenchangensis (FJ261959)
Nonomuraea fastidiosa (U48844)
Nonomuraea zeae (KR106591)
Nonomuraea longicatena (AB018787)
Nonomuraea candida (DQ285421)
Nonomuraea maheshkhaliensis (AB290014)
Nonomuraea fuscirosea (KC417350)
Nonomuraea kuesteri (TYGS 7347)
Nonomuraea jiangxiensis (TYGS 5429)
Nonomuraea muscovyensis (JN896617)
Nonomuraea indica (KM522835)
Nonomuraea salmonea (X97892)
Nonomuraea antimicrobica (FJ157184)
Nonomuraea ferruginea (U48845)
Nonomuraea flavida (JX467533)
Nonomuraea turkmeniaca (AF277201)
Nonomuraea endophytica (GU367158)
Nonomuraea roseoviolacea subsp. roseoviolacea (AB039959)
Nonomuraea roseoviolacea subsp. carminata (AB039961)
Nonomuraea rubra (AF277200)
Nonomuraea maritima (GU002054)
Nonomuraea africana (U48842)
Nonomuraea recticatena (U48979)
Nonomuraea dietziae (AB006156)
Nonomuraea roseola (AJ278221)
Nonomuraea thailandensis (AB720113)
Nonomuraea rosea (FN356742)
Nonomuraea solani (JQ073731)
Nonomuraea monospora (FJ347524)
Nonomuraea rhizophila (HM755723)
Nonomuraea purpurea (LC146616)
Nonomuraea syzygii (KF667499)
Nonomuraea jabiensis (HQ157186)
Nonomuraea gerenzanensis (AJ582011)
Thermocatellispora tengchongensis (JN315666)
Thermomonospora chromogena (AF116558)
Planomonospora parontospora subsp. parontospora (D85495)
Planomonospora parontospora subsp. antibiotica (AB028653)
Planomonospora sphaerica (TYGS 5075)
Planobispora rosea (AB028654)
Planobispora longispora (D85494)
Planomonospora venezuelensis (AB028655)
Planobispora siamensis (HQ599197)
Planobispora takensis (KC865732)
Planomonospora corallina (KU159576)
Planomonospora alba (AB062381)
Streptosporangium anatoliense (HQ157194)
Streptosporangium sandarakinum (JX977118)
Streptosporangium nondiastaticum (X89945)
Streptosporangium pseudovulgare (X89946)
Streptosporangium fragile (U48992)
Streptosporangium becharense (KU593506)
Streptosporangium carneum (X89938)
Streptosporangium amethystogenes subsp. fukuiense (AB537172)
Streptosporangium amethystogenes subsp. amethystogenes (TYGS 5347)
Streptosporangium subroseum (AF191734)
Streptosporangium nanhuense (KF146932)
Streptosporangium vulgare (U48999)
Streptosporangium fenghuangense (KF982694)
Streptosporangium lutulentum (KF887910)
Streptosporangium corydalis (KF956806)
Streptosporangium longisporum (X89944)
Streptosporangium shengliense (KC513503)
Streptosporangium taraxaci (KM000840)
Streptosporangium jiaoheense (KM000836)
Streptosporangium album (X89934)
Streptosporangium canum (AY996844)
Streptosporangium roseum (TYGS 75)
Streptosporangium purpuratum (AF191735)
Streptosporangium yunnanense (AF191733)
Streptosporangium oxazolinicum (AB594818)
Streptosporangium terrae (JX082289)
Streptosporangium violaceochromogenes (U48997)
Streptosporangium algeriense (KP974644)
Streptosporangium jomthongense (JQ922513)
Streptosporangium saharense (KT581983)
Sphaerisporangium melleum (AB208714)
Thermopolyspora flexuosa (AY039253)
Fodinicola feengrottensis (EF490376)
Cryptosporangium mongoliense (AB531472)
Cryptosporangium japonicum (D85466)
Cryptosporangium cibodasense (LC025527)
Cryptosporangium arvum (TYGS 1084)
Cryptosporangium minutisporangium (AB037007)
Cryptosporangium aurantiacum (AJ293746)
Phytomonospora cypria (KJ755850)
Phytomonospora endophytica (GU367156)
Actinorhabdospora filicis (LC120802)
Stackebrandtia endophytica (KJ781245)
Stackebrandtia albiflava (DQ985165)
Stackebrandtia cavernae (KT312956)
Stackebrandtia nassauensis (TYGS 73)
Salilacibacter albus (KR149811)
Paraglycomyces xinjiangensis (AB857719)
Salininema proteolyticum (KP162267)
Haloglycomyces albus (TYGS 1205)
Glycomyces phytohabitans (JQ819256)
Glycomyces tarimensis (KC112995)
Glycomyces tenuis (TYGS 1096)
Glycomyces arizonensis (TYGS 1396)
Glycomyces harbinensis (D85483)
Glycomyces endophyticus (EU200681)
Glycomyces algeriensis (AY462044)

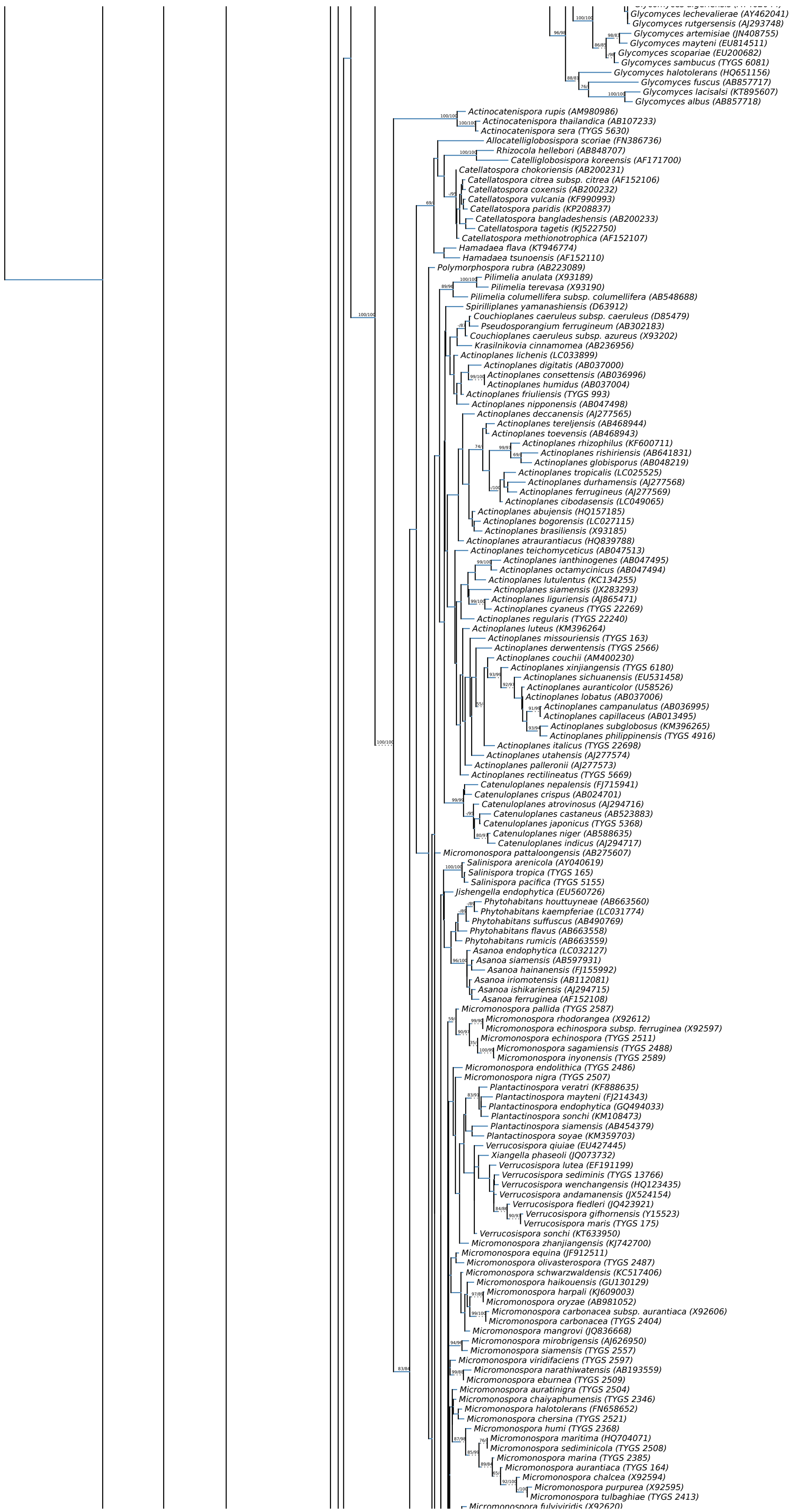
Glycomyces lechevalierae (AY462041)
Glycomyces rutgersensis (AJ293748)
Glycomyces artemisiae (JN408755)
Glycomyces mayteni (EU814511)
Glycomyces scopariae (EU200682)
Glycomyces sambucus (TYGS 6081)
Glycomyces halotolerans (HQ651156)
Glycomyces fuscus (AB857717)
Glycomyces lacisalsi (KT895607)
Glycomyces albus (AB857718)
Actinocatenispora rupis (AM980986)
Actinocatenispora thailandica (AB107233)
Actinocatenispora sera (TYGS 5630)
Allocatelliglobosispora scoriae (FN386736)
Rhizocola hellebori (AB848707)
Catelliglobosispora koreensis (AF171700)
Catellatospora chokoriensis (AB200231)
Catellatospora citrea subsp. citrea (AF152106)
Catellatospora coxensis (AB200232)
Catellatospora vulcania (KF990993)
Catellatospora paridis (KP208837)
Catellatospora bangladeshensis (AB200233)
Catellatospora tagetis (KJ522750)
Catellatospora methionotrophica (AF152107)
Hamadaea flava (KT946774)
Hamadaea tsunoensis (AF152110)
Polymorphospora rubra (AB223089)
Pilimelia anulata (X93189)
Pilimelia terevasa (X93190)
Pilimelia columellifera subsp. columellifera (AB548688)
Spirilliplanes yamanashiensis (D63912)
Couchioplanes caeruleus subsp. caeruleus (D85479)
Pseudosporangium ferrugineum (AB302183)
Couchioplanes caeruleus subsp. azureus (X93202)
Krasilnikovia cinnamomea (AB236956)
Actinoplanes lichenis (LC033899)
Actinoplanes digitatis (AB037000)
Actinoplanes consettensis (AB036996)
Actinoplanes humidus (AB037004)
Actinoplanes friuliensis (TYGS 993)
Actinoplanes nipponensis (AB047498)
Actinoplanes deccanensis (AJ277565)
Actinoplanes tereljensis (AB468944)
Actinoplanes toevensis (AB468943)
Actinoplanes rhizophilus (KF600711)
Actinoplanes rishiriensis (AB641831)
Actinoplanes globisporus (AB048219)
Actinoplanes tropicalis (LC025525)
Actinoplanes durhamensis (AJ277568)
Actinoplanes ferrugineus (AJ277569)
Actinoplanes cibodasensis (LC049065)
Actinoplanes abujensis (HQ157185)
Actinoplanes bogorensis (LC027115)
Actinoplanes brasiliensis (X93185)
Actinoplanes atraurantiacus (HQ839788)
Actinoplanes teichomyceticus (AB047513)
Actinoplanes ianthinogenes (AB047495)
Actinoplanes octamycinicus (AB047494)
Actinoplanes lutulentus (KC134255)
Actinoplanes siamensis (JX283293)
Actinoplanes liguriensis (AJ865471)
Actinoplanes cyaneus (TYGS 22269)
Actinoplanes regularis (TYGS 22240)
Actinoplanes luteus (KM396264)
Actinoplanes missouriensis (TYGS 163)
Actinoplanes derwentensis (TYGS 2566)
Actinoplanes couchii (AM400230)
Actinoplanes xinjiangensis (TYGS 6180)
Actinoplanes sichuanensis (EU531458)
Actinoplanes auranticolor (U58526)
Actinoplanes lobatus (AB037006)
Actinoplanes campanulatus (AB036995)
Actinoplanes capillaceus (AB013495)
Actinoplanes subglobosus (KM396265)
Actinoplanes philippinensis (TYGS 4916)
Actinoplanes italicus (TYGS 22698)
Actinoplanes utahensis (AJ277574)
Actinoplanes palleronii (AJ277573)
Actinoplanes rectilineatus (TYGS 5669)
Catenuloplanes nepalensis (FJ715941)
Catenuloplanes crispus (AB024701)
Catenuloplanes atrovinosus (AJ294716)
Catenuloplanes castaneus (AB523883)
Catenuloplanes japonicus (TYGS 5368)
Catenuloplanes niger (AB588635)
Catenuloplanes indicus (AJ294717)
Micromonospora pattaloongensis (AB275607)
Salinispora arenicola (AY040619)
Salinispora tropica (TYGS 165)
Salinispora pacifica (TYGS 5155)
Jishengella endophytica (EU560726)
Phytohabitans houttuyneae (AB663560)
Phytohabitans kaempferiae (LC031774)
Phytohabitans suffuscus (AB490769)
Phytohabitans flavus (AB663558)
Phytohabitans rumicis (AB663559)
Asanoa endophytica (LC032127)
Asanoa siamensis (AB597931)
Asanoa hainanensis (FJ155992)
Asanoa iriomotensis (AB112081)
Asanoa ishikariensis (AJ294715)
Asanoa ferruginea (AF152108)
Micromonospora pallida (TYGS 2587)
Micromonospora rhodorangea (X92612)
Micromonospora echinospora subsp. ferruginea (X92597)
Micromonospora echinospora (TYGS 2511)
Micromonospora sagamiensis (TYGS 2488)
Micromonospora inyonensis (TYGS 2589)
Micromonospora endolithica (TYGS 2486)
Micromonospora nigra (TYGS 2507)
Plantactinospora veratri (KF888635)
Plantactinospora mayteni (FJ214343)
Plantactinospora endophytica (GQ494033)
Plantactinospora sonchi (KM108473)
Plantactinospora siamensis (AB454379)
Plantactinospora soyae (KM359703)
Verrucosispora qiuiae (EU427445)
Xiangella phaseoli (JQ073732)
Verrucosispora lutea (EF191199)
Verrucosispora sediminis (TYGS 13766)
Verrucosispora wenchangensis (HQ123435)
Verrucosispora andamanensis (JX524154)
Verrucosispora fiedleri (JQ423921)
Verrucosispora gifhornensis (Y15523)
Verrucosispora maris (TYGS 175)
Verrucosispora sonchi (KT633950)
Micromonospora zhanjiangensis (KJ742700)
Micromonospora equina (JF912511)
Micromonospora olivasterospora (TYGS 2487)
Micromonospora schwarzwaldensis (KC517406)
Micromonospora haikouensis (GU130129)
Micromonospora harpali (KJ609003)
Micromonospora oryzae (AB981052)
Micromonospora carbonacea subsp. aurantiaca (X92606)
Micromonospora carbonacea (TYGS 2404)
Micromonospora mangrovi (JQ836668)
Micromonospora mirobrigensis (AJ626950)
Micromonospora siamensis (TYGS 2557)
Micromonospora viridifaciens (TYGS 2597)
Micromonospora narathiwatensis (AB193559)
Micromonospora eburnea (TYGS 2509)
Micromonospora auratinigra (TYGS 2504)
Micromonospora chaiyaphumensis (TYGS 2346)
Micromonospora halotolerans (FN658652)
Micromonospora chersina (TYGS 2521)
Micromonospora humi (TYGS 2368)
Micromonospora maritima (HQ704071)
Micromonospora sediminicola (TYGS 2508)
Micromonospora marina (TYGS 2385)
Micromonospora aurantiaca (TYGS 164)
Micromonospora chalcea (X92594)
Micromonospora purpurea (X92595)
Micromonospora tulbaghiae (TYGS 2413)
Micromonospora fulviviridis (X92620)

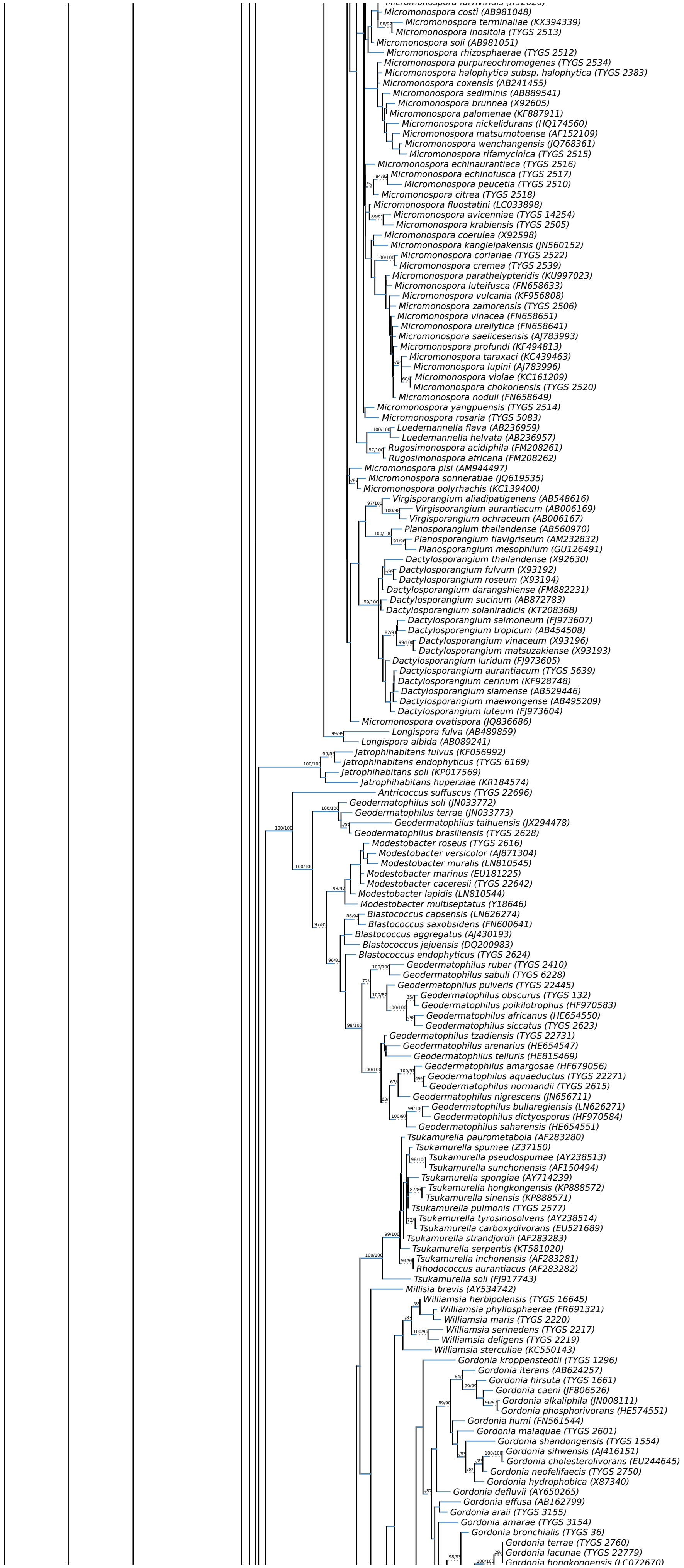
Micromonospora costi (AB981048)
Micromonospora terminaliae (KX394339)
Micromonospora inositola (TYGS 2513)
Micromonospora soli (AB981051)
Micromonospora rhizosphaerae (TYGS 2512)
Micromonospora purpureochromogenes (TYGS 2534)
Micromonospora halophytica subsp. halophytica (TYGS 2383)
Micromonospora coxensis (AB241455)
Micromonospora sediminis (AB889541)
Micromonospora brunnea (X92605)
Micromonospora palomenae (KF887911)
Micromonospora nickelidurans (HQ174560)
Micromonospora matsumotoense (AF152109)
Micromonospora wenchangensis (JQ768361)
Micromonospora rifamycinica (TYGS 2515)
Micromonospora echinaurantiaca (TYGS 2516)
Micromonospora echinofusca (TYGS 2517)
Micromonospora peucetia (TYGS 2510)
Micromonospora citrea (TYGS 2518)
Micromonospora fluostatini (LC033898)
Micromonospora avicenniae (TYGS 14254)
Micromonospora krabiensis (TYGS 2505)
Micromonospora coerulea (X92598)
Micromonospora kangleipakensis (JN560152)
Micromonospora coriariae (TYGS 2522)
Micromonospora cremea (TYGS 2539)
Micromonospora parathelypteridis (KU997023)
Micromonospora luteifusca (FN658633)
Micromonospora vulcania (KF956808)
Micromonospora zamorensis (TYGS 2506)
Micromonospora vinacea (FN658651)
Micromonospora ureilytica (FN658641)
Micromonospora saelicesensis (AJ783993)
Micromonospora profundi (KF494813)
Micromonospora taraxaci (KC439463)
Micromonospora lupini (AJ783996)
Micromonospora violae (KC161209)
Micromonospora chokoriensis (TYGS 2520)
Micromonospora noduli (FN658649)
Micromonospora yangpuensis (TYGS 2514)
Micromonospora rosaria (TYGS 5083)
Luedemannella flava (AB236959)
Luedemannella helvata (AB236957)
Rugosimonospora acidiphila (FM208261)
Rugosimonospora africana (FM208262)
Micromonospora pisi (AM944497)
Micromonospora sonneratiae (JQ619535)
Micromonospora polyrhachis (KC139400)
Virgisporangium aliadipatigenens (AB548616)
Virgisporangium aurantiacum (AB006169)
Virgisporangium ochraceum (AB006167)
Planosporangium thailandense (AB560970)
Planosporangium flavigriseum (AM232832)
Planosporangium mesophilum (GU126491)
Dactylosporangium thailandense (X92630)
Dactylosporangium fulvum (X93192)
Dactylosporangium roseum (X93194)
Dactylosporangium darangshiense (FM882231)
Dactylosporangium sucinum (AB872783)
Dactylosporangium solaniradicis (KT208368)
Dactylosporangium salmoneum (FJ973607)
Dactylosporangium tropicum (AB454508)
Dactylosporangium vinaceum (X93196)
Dactylosporangium matsuzakiense (X93193)
Dactylosporangium luridum (FJ973605)
Dactylosporangium aurantiacum (TYGS 5639)
Dactylosporangium cerinum (KF928748)
Dactylosporangium siamense (AB529446)
Dactylosporangium maewongense (AB495209)
Dactylosporangium luteum (FJ973604)
Micromonospora ovatispora (JQ836686)
Longispora fulva (AB489859)
Longispora albida (AB089241)
Jatrophihabitans fulvus (KF056992)
Jatrophihabitans endophyticus (TYGS 6169)
Jatrophihabitans soli (KP017569)
Jatrophihabitans huperziae (KR184574)
Antricoccus suffuscus (TYGS 22696)
Geodermatophilus soli (JN033772)
Geodermatophilus terrae (JN033773)
Geodermatophilus taihuensis (JX294478)
Geodermatophilus brasiliensis (TYGS 2628)
Modestobacter roseus (TYGS 2616)
Modestobacter versicolor (AJ871304)
Modestobacter muralis (LN810545)
Modestobacter marinus (EU181225)
Modestobacter caceresii (TYGS 22642)
Modestobacter lapidis (LN810544)
Modestobacter multiseptatus (Y18646)
Blastococcus capsensis (LN626274)
Blastococcus saxobsidens (FN600641)
Blastococcus aggregatus (AJ430193)
Blastococcus jejuensis (DQ200983)
Blastococcus endophyticus (TYGS 2624)
Geodermatophilus ruber (TYGS 2410)
Geodermatophilus sabuli (TYGS 6228)
Geodermatophilus pulveris (TYGS 22445)
Geodermatophilus obscurus (TYGS 132)
Geodermatophilus poikilotrophus (HF970583)
Geodermatophilus africanus (HE654550)
Geodermatophilus siccatus (TYGS 2623)
Geodermatophilus tzadiensis (TYGS 22731)
Geodermatophilus arenarius (HE654547)
Geodermatophilus telluris (HE815469)
Geodermatophilus amargosae (HF679056)
Geodermatophilus aquaeductus (TYGS 22271)
Geodermatophilus normandii (TYGS 2615)
Geodermatophilus nigrescens (JN656711)
Geodermatophilus bullaregiensis (LN626271)
Geodermatophilus dictyosporus (HF970584)
Geodermatophilus saharensis (HE654551)
Tsukamurella paurometabola (AF283280)
Tsukamurella spumae (Z37150)
Tsukamurella pseudospumae (AY238513)
Tsukamurella sunchonensis (AF150494)
Tsukamurella spongiae (AY714239)
Tsukamurella hongkongensis (KP888572)
Tsukamurella sinensis (KP888571)
Tsukamurella pulmonis (TYGS 2577)
Tsukamurella tyrosinosolvens (AY238514)
Tsukamurella carboxydivorans (EU521689)
Tsukamurella strandjordii (AF283283)
Tsukamurella serpentis (KT581020)
Tsukamurella inchonensis (AF283281)
Rhodococcus aurantiacus (AF283282)
Tsukamurella soli (FJ917743)
Millisia brevis (AY534742)
Williamsia herbipolensis (TYGS 16645)
Williamsia phyllosphaerae (FR691321)
Williamsia maris (TYGS 2220)
Williamsia serinedens (TYGS 2217)
Williamsia deligens (TYGS 2219)
Williamsia sterculiae (KC550143)
Gordonia kroppenstedtii (TYGS 1296)
Gordonia iterans (AB624257)
Gordonia hirsuta (TYGS 1661)
Gordonia caeni (JF806526)
Gordonia alkaliphila (JN008111)
Gordonia phosphorivorans (HE574551)
Gordonia humi (FN561544)
Gordonia malaquae (TYGS 2601)
Gordonia shandongensis (TYGS 1554)
Gordonia sihwensis (AJ416151)
Gordonia cholesterolivorans (EU244645)
Gordonia neofelifaecis (TYGS 2750)
Gordonia hydrophobica (X87340)
Gordonia defluvii (AY650265)
Gordonia effusa (AB162799)
Gordonia araii (TYGS 3155)
Gordonia amarae (TYGS 3154)
Gordonia bronchialis (TYGS 36)
Gordonia terrae (TYGS 2760)
Gordonia lacunae (TYGS 22779)
Gordonia hongkongensis (LC072670)

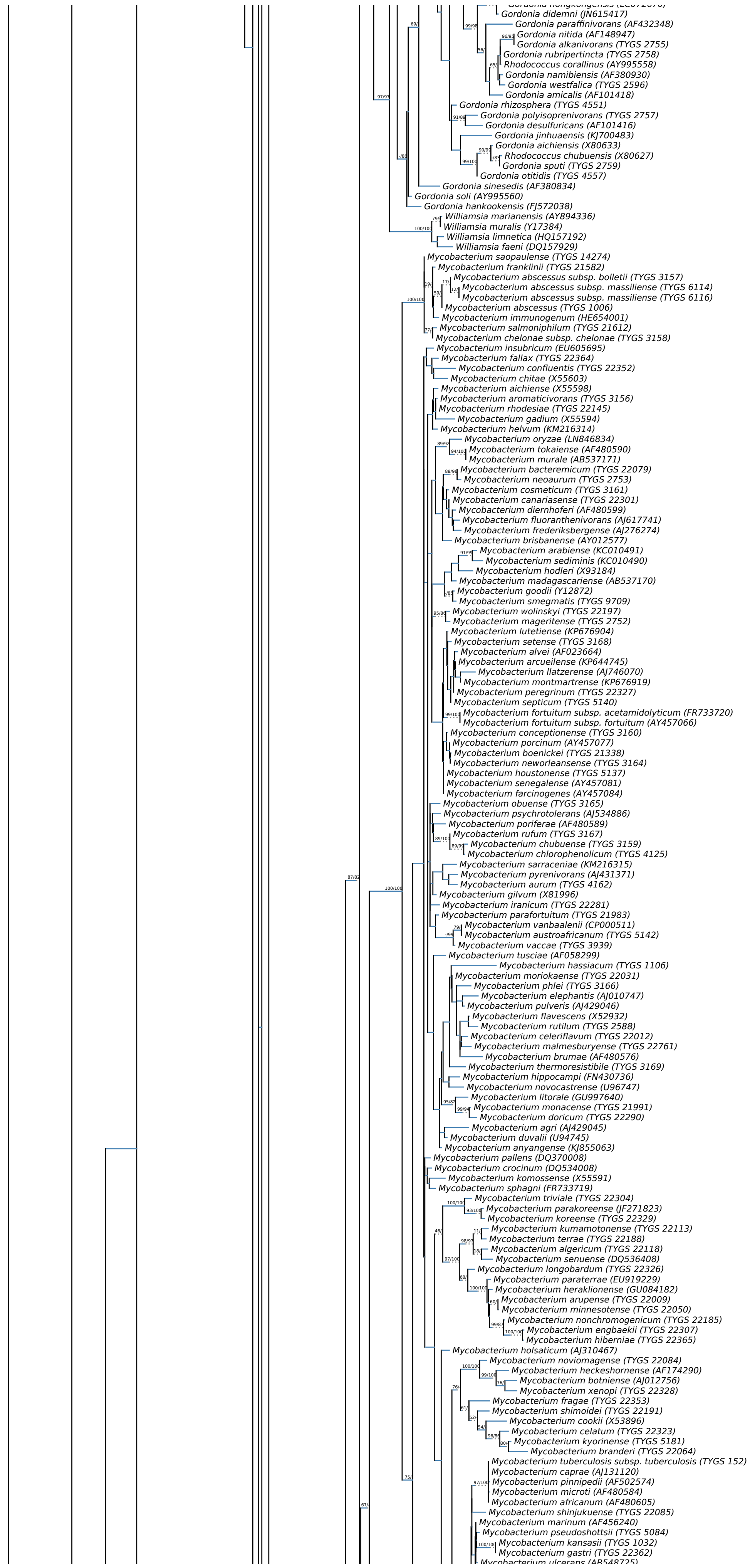

Gordonia didemni (JN615417)
Gordonia paraffinivorans (AF432348)
Gordonia nitida (AF148947)
Gordonia alkanivorans (TYGS 2755)
Gordonia rubripertincta (TYGS 2758)
Rhodococcus corallinus (AY995558)
Gordonia namibiensis (AF380930)
Gordonia westfalica (TYGS 2596)
Gordonia amicalis (AF101418)
Gordonia rhizosphera (TYGS 4551)
Gordonia polyisoprenivorans (TYGS 2757)
Gordonia desulfuricans (AF101416)
Gordonia jinhuaensis (KJ700483)
Gordonia aichiensis (X80633)
Rhodococcus chubuensis (X80627)
Gordonia sputi (TYGS 2759)
Gordonia otitidis (TYGS 4557)
Gordonia sinesedis (AF380834)
Gordonia soli (AY995560)
Gordonia hankookensis (FJ572038)
Williamsia marianensis (AY894336)
Williamsia muralis (Y17384)
Williamsia limnetica (HQ157192)
Williamsia faeni (DQ157929)
Mycobacterium saopaulense (TYGS 14274)
Mycobacterium franklinii (TYGS 21582)
Mycobacterium abscessus subsp. bolletii (TYGS 3157)
Mycobacterium abscessus subsp. massiliense (TYGS 6114)
Mycobacterium abscessus subsp. massiliense (TYGS 6116)
Mycobacterium abscessus (TYGS 1006)
Mycobacterium immunogenum (HE654001)
Mycobacterium salmoniphilum (TYGS 21612)
Mycobacterium chelonae subsp. chelonae (TYGS 3158)
Mycobacterium insubricum (EU605695)
Mycobacterium fallax (TYGS 22364)
Mycobacterium confluentis (TYGS 22352)
Mycobacterium chitae (X55603)
Mycobacterium aichiense (X55598)
Mycobacterium aromaticivorans (TYGS 3156)
Mycobacterium rhodesiae (TYGS 22145)
Mycobacterium gadium (X55594)
Mycobacterium helvum (KM216314)
Mycobacterium oryzae (LN846834)
Mycobacterium tokaiense (AF480590)
Mycobacterium murale (AB537171)
Mycobacterium bacteremicum (TYGS 22079)
Mycobacterium neoaurum (TYGS 2753)
Mycobacterium cosmeticum (TYGS 3161)
Mycobacterium canariasense (TYGS 22301)
Mycobacterium diernhoferi (AF480599)
Mycobacterium fluoranthenivorans (AJ617741)
Mycobacterium frederiksbergense (AJ276274)
Mycobacterium brisbanense (AY012577)
Mycobacterium arabiense (KC010491)
Mycobacterium sediminis (KC010490)
Mycobacterium hodleri (X93184)
Mycobacterium madagascariense (AB537170)
Mycobacterium goodii (Y12872)
Mycobacterium smegmatis (TYGS 9709)
Mycobacterium wolinskyi (TYGS 22197)
Mycobacterium mageritense (TYGS 2752)
Mycobacterium lutetiense (KP676904)
Mycobacterium setense (TYGS 3168)
Mycobacterium alvei (AF023664)
Mycobacterium arcueilense (KP644745)
Mycobacterium llatzerense (AJ746070)
Mycobacterium montmartrense (KP676919)
Mycobacterium peregrinum (TYGS 22327)
Mycobacterium septicum (TYGS 5140)
Mycobacterium fortuitum subsp. acetamidolyticum (FR733720)
Mycobacterium fortuitum subsp. fortuitum (AY457066)
Mycobacterium conceptionense (TYGS 3160)
Mycobacterium porcinum (AY457077)
Mycobacterium boenickei (TYGS 21338)
Mycobacterium neworleansense (TYGS 3164)
Mycobacterium houstonense (TYGS 5137)
Mycobacterium senegalense (AY457081)
Mycobacterium farcinogenes (AY457084)
Mycobacterium obuense (TYGS 3165)
Mycobacterium psychrotolerans (AJ534886)
Mycobacterium poriferae (AF480589)
Mycobacterium rufum (TYGS 3167)
Mycobacterium chubuense (TYGS 3159)
Mycobacterium chlorophenolicum (TYGS 4125)
Mycobacterium sarraceniae (KM216315)
Mycobacterium pyrenivorans (AJ431371)
Mycobacterium aurum (TYGS 4162)
Mycobacterium gilvum (X81996)
Mycobacterium iranicum (TYGS 22281)
Mycobacterium parafortuitum (TYGS 21983)
Mycobacterium vanbaalenii (CP000511)
Mycobacterium austroafricanum (TYGS 5142)
Mycobacterium vaccae (TYGS 3939)
Mycobacterium tusciae (AF058299)
Mycobacterium hassiacum (TYGS 1106)
Mycobacterium moriokaense (TYGS 22031)
Mycobacterium phlei (TYGS 3166)
Mycobacterium elephantis (AJ010747)
Mycobacterium pulveris (AJ429046)
Mycobacterium flavescens (X52932)
Mycobacterium rutilum (TYGS 2588)
Mycobacterium celeriflavum (TYGS 22012)
Mycobacterium malmesburyense (TYGS 22761)
Mycobacterium brumae (AF480576)
Mycobacterium thermoresistibile (TYGS 3169)
Mycobacterium hippocampi (FN430736)
Mycobacterium novocastrense (U96747)
Mycobacterium litorale (GU997640)
Mycobacterium monacense (TYGS 21991)
Mycobacterium doricum (TYGS 22290)
Mycobacterium agri (AJ429045)
Mycobacterium duvalii (U94745)
Mycobacterium anyangense (KJ855063)
Mycobacterium pallens (DQ370008)
Mycobacterium crocinum (DQ534008)
Mycobacterium komossense (X55591)
Mycobacterium sphagni (FR733719)
Mycobacterium triviale (TYGS 22304)
Mycobacterium parakoreense (JF271823)
Mycobacterium koreense (TYGS 22329)
Mycobacterium kumamotonense (TYGS 22113)
Mycobacterium terrae (TYGS 22188)
Mycobacterium algericum (TYGS 22118)
Mycobacterium senuense (DQ536408)
Mycobacterium longobardum (TYGS 22326)
Mycobacterium paraterrae (EU919229)
Mycobacterium heraklionense (GU084182)
Mycobacterium arupense (TYGS 22009)
Mycobacterium minnesotense (TYGS 22050)
Mycobacterium nonchromogenicum (TYGS 22185)
Mycobacterium engbaekii (TYGS 22307)
Mycobacterium hiberniae (TYGS 22365)
Mycobacterium holsaticum (AJ310467)
Mycobacterium noviomagense (TYGS 22084)
Mycobacterium heckeshornense (AF174290)
Mycobacterium botniense (AJ012756)
Mycobacterium xenopi (TYGS 22328)
Mycobacterium fragae (TYGS 22353)
Mycobacterium shimoidei (TYGS 22191)
Mycobacterium cookii (X53896)
Mycobacterium celatum (TYGS 22323)
Mycobacterium kyorinense (TYGS 5181)
Mycobacterium branderi (TYGS 22064)
Mycobacterium tuberculosis subsp. tuberculosis (TYGS 152)
Mycobacterium caprae (AJ131120)
Mycobacterium pinnipedii (AF502574)
Mycobacterium microti (AF480584)
Mycobacterium africanum (AF480605)
Mycobacterium shinjukuense (TYGS 22085)
Mycobacterium marinum (AF456240)
Mycobacterium pseudoshottsii (TYGS 5084)
Mycobacterium kansasii (TYGS 1032)
Mycobacterium gastri (TYGS 22362)

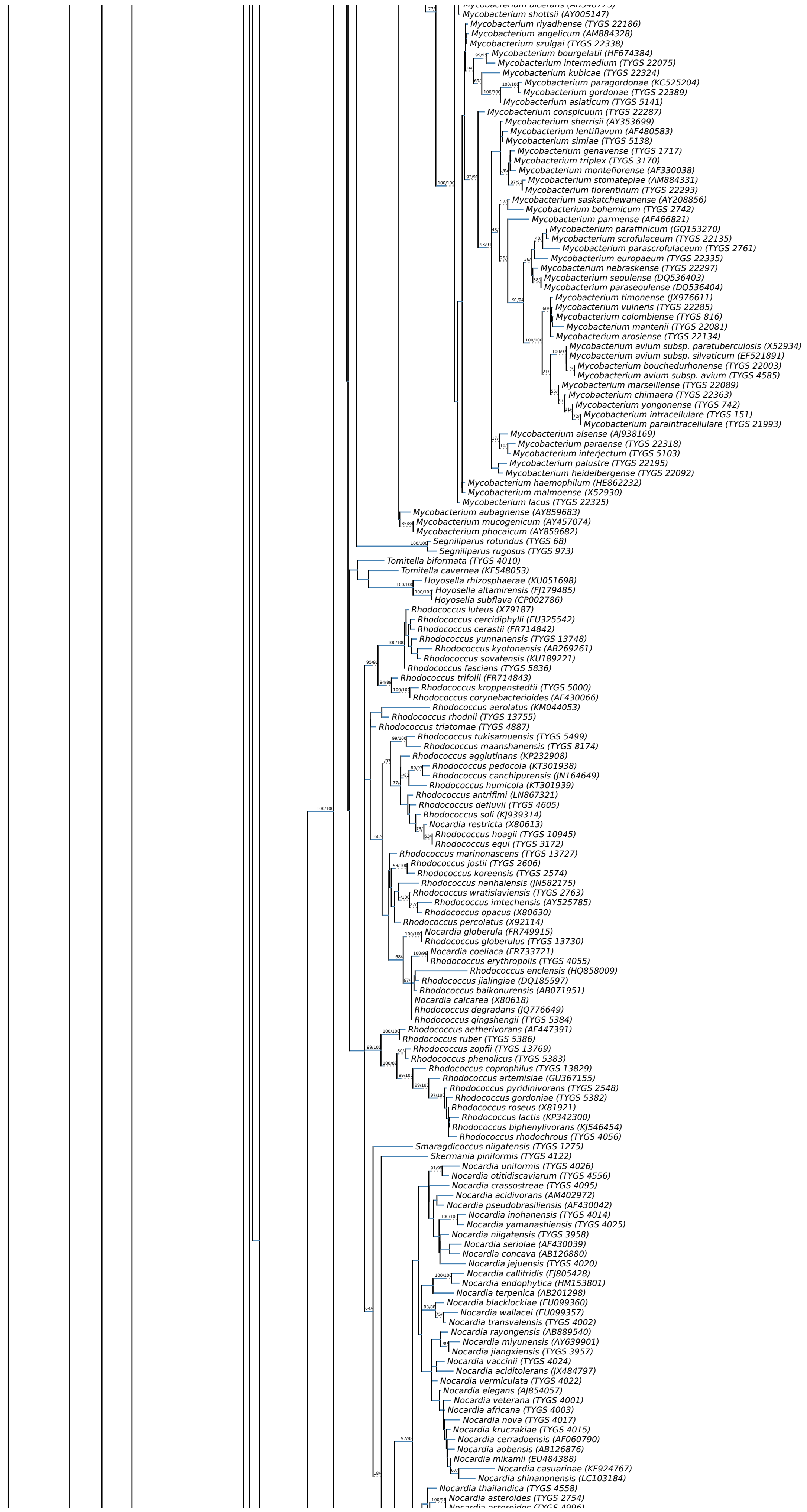

Mycobacterium shottsii (AY005147)
Mycobacterium riyadhense (TYGS 22186)
Mycobacterium angelicum (AM884328)
Mycobacterium szulgai (TYGS 22338)
Mycobacterium bourgelatii (HF674384)
Mycobacterium intermedium (TYGS 22075)
Mycobacterium kubicae (TYGS 22324)
Mycobacterium paragordonae (KC525204)
Mycobacterium gordonae (TYGS 22389)
Mycobacterium asiaticum (TYGS 5141)
Mycobacterium conspicuum (TYGS 22287)
Mycobacterium sherrisii (AY353699)
Mycobacterium lentiflavum (AF480583)
Mycobacterium simiae (TYGS 5138)
Mycobacterium genavense (TYGS 1717)
Mycobacterium triplex (TYGS 3170)
Mycobacterium montefiorense (AF330038)
Mycobacterium stomatepiae (AM884331)
Mycobacterium florentinum (TYGS 22293)
Mycobacterium saskatchewanense (AY208856)
Mycobacterium bohemicum (TYGS 2742)
Mycobacterium parmense (AF466821)
Mycobacterium paraffinicum (GQ153270)
Mycobacterium scrofulaceum (TYGS 22135)
Mycobacterium parascrofulaceum (TYGS 2761)
Mycobacterium europaeum (TYGS 22335)
Mycobacterium nebraskense (TYGS 22297)
Mycobacterium seoulense (DQ536403)
Mycobacterium paraseoulense (DQ536404)
Mycobacterium timonense (JX976611)
Mycobacterium vulneris (TYGS 22285)
Mycobacterium colombiense (TYGS 816)
Mycobacterium mantenii (TYGS 22081)
Mycobacterium arosiense (TYGS 22134)
Mycobacterium avium subsp. paratuberculosis (X52934)
Mycobacterium avium subsp. silvaticum (EF521891)
Mycobacterium bouchedurhonense (TYGS 22003)
Mycobacterium avium subsp. avium (TYGS 4585)
Mycobacterium marseillense (TYGS 22089)
Mycobacterium chimaera (TYGS 22363)
Mycobacterium yongonense (TYGS 742)
Mycobacterium intracellulare (TYGS 151)
Mycobacterium paraintracellulare (TYGS 21993)
Mycobacterium alsense (AJ938169)
Mycobacterium paraense (TYGS 22318)
Mycobacterium interjectum (TYGS 5103)
Mycobacterium palustre (TYGS 22195)
Mycobacterium heidelbergense (TYGS 22092)
Mycobacterium haemophilum (HE862232)
Mycobacterium malmoense (X52930)
Mycobacterium lacus (TYGS 22325)
Mycobacterium aubagnense (AY859683)
Mycobacterium mucogenicum (AY457074)
Mycobacterium phocaicum (AY859682)
Segniliparus rotundus (TYGS 68)
Segniliparus rugosus (TYGS 973)
Tomitella biformata (TYGS 4010)
Tomitella cavernea (KF548053)
Hoyosella rhizosphaerae (KU051698)
Hoyosella altamirensis (FJ179485)
Hoyosella subflava (CP002786)
Rhodococcus luteus (X79187)
Rhodococcus cercidiphylli (EU325542)
Rhodococcus cerastii (FR714842)
Rhodococcus yunnanensis (TYGS 13748)
Rhodococcus kyotonensis (AB269261)
Rhodococcus sovatensis (KU189221)
Rhodococcus fascians (TYGS 5836)
Rhodococcus trifolii (FR714843)
Rhodococcus kroppenstedtii (TYGS 5000)
Rhodococcus corynebacterioides (AF430066)
Rhodococcus aerolatus (KM044053)
Rhodococcus rhodnii (TYGS 13755)
Rhodococcus triatomae (TYGS 4887)
Rhodococcus tukisamuensis (TYGS 5499)
Rhodococcus maanshanensis (TYGS 8174)
Rhodococcus agglutinans (KP232908)
Rhodococcus pedocola (KT301938)
Rhodococcus canchipurensis (JN164649)
Rhodococcus humicola (KT301939)
Rhodococcus antrifimi (LN867321)
Rhodococcus defluvii (TYGS 4605)
Rhodococcus soli (KJ939314)
Nocardia restricta (X80613)
Rhodococcus hoagii (TYGS 10945)
Rhodococcus equi (TYGS 3172)
Rhodococcus marinonascens (TYGS 13727)
Rhodococcus jostii (TYGS 2606)
Rhodococcus koreensis (TYGS 2574)
Rhodococcus nanhaiensis (JN582175)
Rhodococcus wratislaviensis (TYGS 2763)
Rhodococcus imtechensis (AY525785)
Rhodococcus opacus (X80630)
Rhodococcus percolatus (X92114)
Nocardia globerula (FR749915)
Rhodococcus globerulus (TYGS 13730)
Nocardia coeliaca (FR733721)
Rhodococcus erythropolis (TYGS 4055)
Rhodococcus enclensis (HQ858009)
Rhodococcus jialingiae (DQ185597)
Rhodococcus baikonurensis (AB071951)
Nocardia calcarea (X80618)
Rhodococcus degradans (JQ776649)
Rhodococcus qingshengii (TYGS 5384)
Rhodococcus aetherivorans (AF447391)
Rhodococcus ruber (TYGS 5386)
Rhodococcus zopfii (TYGS 13769)
Rhodococcus phenolicus (TYGS 5383)
Rhodococcus coprophilus (TYGS 13829)
Rhodococcus artemisiae (GU367155)
Rhodococcus pyridinivorans (TYGS 2548)
Rhodococcus gordoniae (TYGS 5382)
Rhodococcus roseus (X81921)
Rhodococcus lactis (KP342300)
Rhodococcus biphenylivorans (KJ546454)
Rhodococcus rhodochrous (TYGS 4056)
Smaragdicoccus niigatensis (TYGS 1275)
Skermania piniformis (TYGS 4122)
Nocardia uniformis (TYGS 4026)
Nocardia otitidiscaviarum (TYGS 4556)
Nocardia crassostreae (TYGS 4095)
Nocardia acidivorans (AM402972)
Nocardia pseudobrasiliensis (AF430042)
Nocardia inohanensis (TYGS 4014)
Nocardia yamanashiensis (TYGS 4025)
Nocardia niigatensis (TYGS 3958)
Nocardia seriolae (AF430039)
Nocardia concava (AB126880)
Nocardia jejuensis (TYGS 4020)
Nocardia callitridis (FJ805428)
Nocardia endophytica (HM153801)
Nocardia terpenica (AB201298)
Nocardia blacklockiae (EU099360)
Nocardia wallacei (EU099357)
Nocardia transvalensis (TYGS 4002)
Nocardia rayongensis (AB889540)
Nocardia miyunensis (AY639901)
Nocardia jiangxiensis (TYGS 3957)
Nocardia vaccinii (TYGS 4024)
Nocardia aciditolerans (JX484797)
Nocardia vermiculata (TYGS 4022)
Nocardia elegans (AJ854057)
Nocardia veterana (TYGS 4001)
Nocardia africana (TYGS 4003)
Nocardia nova (TYGS 4017)
Nocardia kruczakiae (TYGS 4015)
Nocardia cerradoensis (AF060790)
Nocardia aobensis (AB126876)
Nocardia mikamii (EU484388)
Nocardia casuarinae (KF924767)
Nocardia shinanonensis (LC103184)
Nocardia thailandica (TYGS 4558)
Nocardia asteroides (TYGS 2754)
Nocardia asteroides (TYGS 4996)

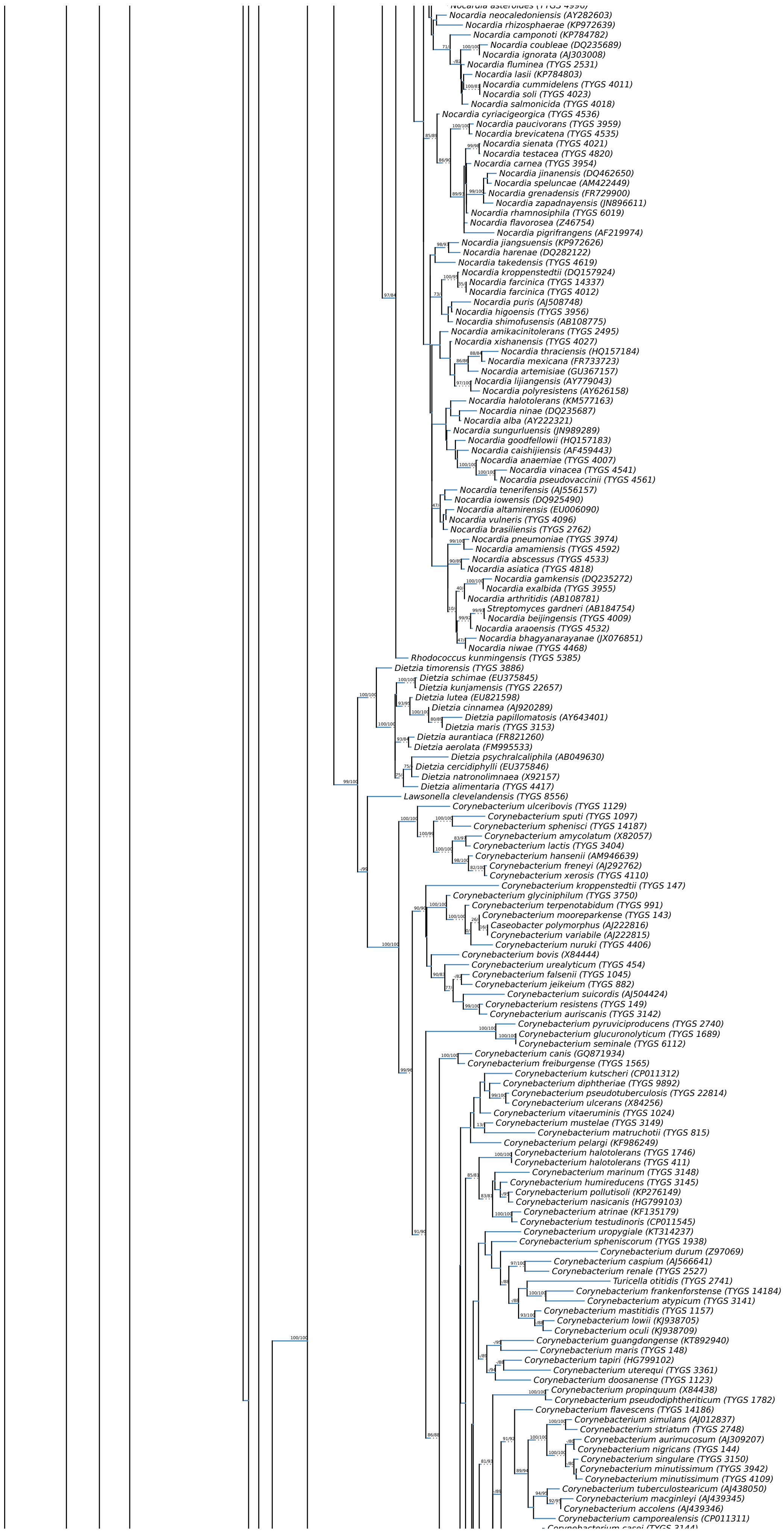
Nocardia neocaledoniensis (AY282603)
Nocardia rhizosphaerae (KP972639)
Nocardia camponoti (KP784782)
Nocardia coubleae (DQ235689)
Nocardia ignorata (AJ303008)
Nocardia fluminea (TYGS 2531)
Nocardia lasii (KP784803)
Nocardia cummidelens (TYGS 4011)
Nocardia soli (TYGS 4023)
Nocardia salmonicida (TYGS 4018)
Nocardia cyriacigeorgica (TYGS 4536)
Nocardia paucivorans (TYGS 3959)
Nocardia brevicatena (TYGS 4535)
Nocardia sienata (TYGS 4021)
Nocardia testacea (TYGS 4820)
Nocardia carnea (TYGS 3954)
Nocardia jinanensis (DQ462650)
Nocardia speluncae (AM422449)
Nocardia grenadensis (FR729900)
Nocardia zapadnayensis (JN896611)
Nocardia rhamnosiphila (TYGS 6019)
Nocardia flavorosea (Z46754)
Nocardia pigrifrangens (AF219974)
Nocardia jiangsuensis (KP972626)
Nocardia harenae (DQ282122)
Nocardia takedensis (TYGS 4619)
Nocardia kroppenstedtii (DQ157924)
Nocardia farcinica (TYGS 14337)
Nocardia farcinica (TYGS 4012)
Nocardia puris (AJ508748)
Nocardia higoensis (TYGS 3956)
Nocardia shimofusensis (AB108775)
Nocardia amikacinitolerans (TYGS 2495)
Nocardia xishanensis (TYGS 4027)
Nocardia thraciensis (HQ157184)
Nocardia mexicana (FR733723)
Nocardia artemisiae (GU367157)
Nocardia lijiangensis (AY779043)
Nocardia polyresistens (AY626158)
Nocardia halotolerans (KM577163)
Nocardia ninae (DQ235687)
Nocardia alba (AY222321)
Nocardia sungurluensis (JN989289)
Nocardia goodfellowii (HQ157183)
Nocardia caishijiensis (AF459443)
Nocardia anaemiae (TYGS 4007)
Nocardia vinacea (TYGS 4541)
Nocardia pseudovaccinii (TYGS 4561)
Nocardia tenerifensis (AJ556157)
Nocardia iowensis (DQ925490)
Nocardia altamirensis (EU006090)
Nocardia vulneris (TYGS 4096)
Nocardia brasiliensis (TYGS 2762)
Nocardia pneumoniae (TYGS 3974)
Nocardia amamiensis (TYGS 4592)
Nocardia abscessus (TYGS 4533)
Nocardia asiatica (TYGS 4818)
Nocardia gamkensis (DQ235272)
Nocardia exalbida (TYGS 3955)
Nocardia arthritidis (AB108781)
Streptomyces gardneri (AB184754)
Nocardia beijingensis (TYGS 4009)
Nocardia araoensis (TYGS 4532)
Nocardia bhagyanarayanae (JX076851)
Nocardia niwae (TYGS 4468)
Rhodococcus kunmingensis (TYGS 5385)
Dietzia timorensis (TYGS 3886)
Dietzia schimae (EU375845)
Dietzia kunjamensis (TYGS 22657)
Dietzia lutea (EU821598)
Dietzia cinnamea (AJ920289)
Dietzia papillomatosis (AY643401)
Dietzia maris (TYGS 3153)
Dietzia aurantiaca (FR821260)
Dietzia aerolata (FM995533)
Dietzia psychralcaliphila (AB049630)
Dietzia cercidiphylli (EU375846)
Dietzia natronolimnaea (X92157)
Dietzia alimentaria (TYGS 4417)
Lawsonella clevelandensis (TYGS 8556)
Corynebacterium ulceribovis (TYGS 1129)
Corynebacterium sputi (TYGS 1097)
Corynebacterium sphenisci (TYGS 14187)
Corynebacterium amycolatum (X82057)
Corynebacterium lactis (TYGS 3404)
Corynebacterium hansenii (AM946639)
Corynebacterium freneyi (AJ292762)
Corynebacterium xerosis (TYGS 4110)
Corynebacterium kroppenstedtii (TYGS 147)
Corynebacterium glyciniphilum (TYGS 3750)
Corynebacterium terpenotabidum (TYGS 991)
Corynebacterium mooreparkense (TYGS 143)
Caseobacter polymorphus (AJ222816)
Corynebacterium variabile (AJ222815)
Corynebacterium nuruki (TYGS 4406)
Corynebacterium bovis (X84444)
Corynebacterium urealyticum (TYGS 454)
Corynebacterium falsenii (TYGS 1045)
Corynebacterium jeikeium (TYGS 882)
Corynebacterium suicordis (AJ504424)
Corynebacterium resistens (TYGS 149)
Corynebacterium auriscanis (TYGS 3142)
Corynebacterium pyruviciproducens (TYGS 2740)
Corynebacterium glucuronolyticum (TYGS 1689)
Corynebacterium seminale (TYGS 6112)
Corynebacterium canis (GQ871934)
Corynebacterium freiburgense (TYGS 1565)
Corynebacterium kutscheri (CP011312)
Corynebacterium diphtheriae (TYGS 9892)
Corynebacterium pseudotuberculosis (TYGS 22814)
Corynebacterium ulcerans (X84256)
Corynebacterium vitaeruminis (TYGS 1024)
Corynebacterium mustelae (TYGS 3149)
Corynebacterium matruchotii (TYGS 815)
Corynebacterium pelargi (KF986249)
Corynebacterium halotolerans (TYGS 1746)
Corynebacterium halotolerans (TYGS 411)
Corynebacterium marinum (TYGS 3148)
Corynebacterium humireducens (TYGS 3145)
Corynebacterium pollutisoli (KP276149)
Corynebacterium nasicanis (HG799103)
Corynebacterium atrinae (KF135179)
Corynebacterium testudinoris (CP011545)
Corynebacterium uropygiale (KT314237)
Corynebacterium spheniscorum (TYGS 1938)
Corynebacterium durum (Z97069)
Corynebacterium caspium (AJ566641)
Corynebacterium renale (TYGS 2527)
Turicella otitidis (TYGS 2741)
Corynebacterium frankenforstense (TYGS 14184)
Corynebacterium atypicum (TYGS 3141)
Corynebacterium mastitidis (TYGS 1157)
Corynebacterium lowii (KJ938705)
Corynebacterium oculi (KJ938709)
Corynebacterium guangdongense (KT892940)
Corynebacterium maris (TYGS 148)
Corynebacterium tapiri (HG799102)
Corynebacterium uterequi (TYGS 3361)
Corynebacterium doosanense (TYGS 1123)
Corynebacterium propinquum (X84438)
Corynebacterium pseudodiphtheriticum (TYGS 1782)
Corynebacterium flavescens (TYGS 14186)
Corynebacterium simulans (AJ012837)
Corynebacterium striatum (TYGS 2748)
Corynebacterium aurimucosum (AJ309207)
Corynebacterium nigricans (TYGS 144)
Corynebacterium singulare (TYGS 3150)
Corynebacterium minutissimum (TYGS 3942)
Corynebacterium minutissimum (TYGS 4109)
Corynebacterium tuberculostearicum (AJ438050)
Corynebacterium macginleyi (AJ439345)
Corynebacterium accolens (AJ439346)
Corynebacterium camporealensis (CP011311)

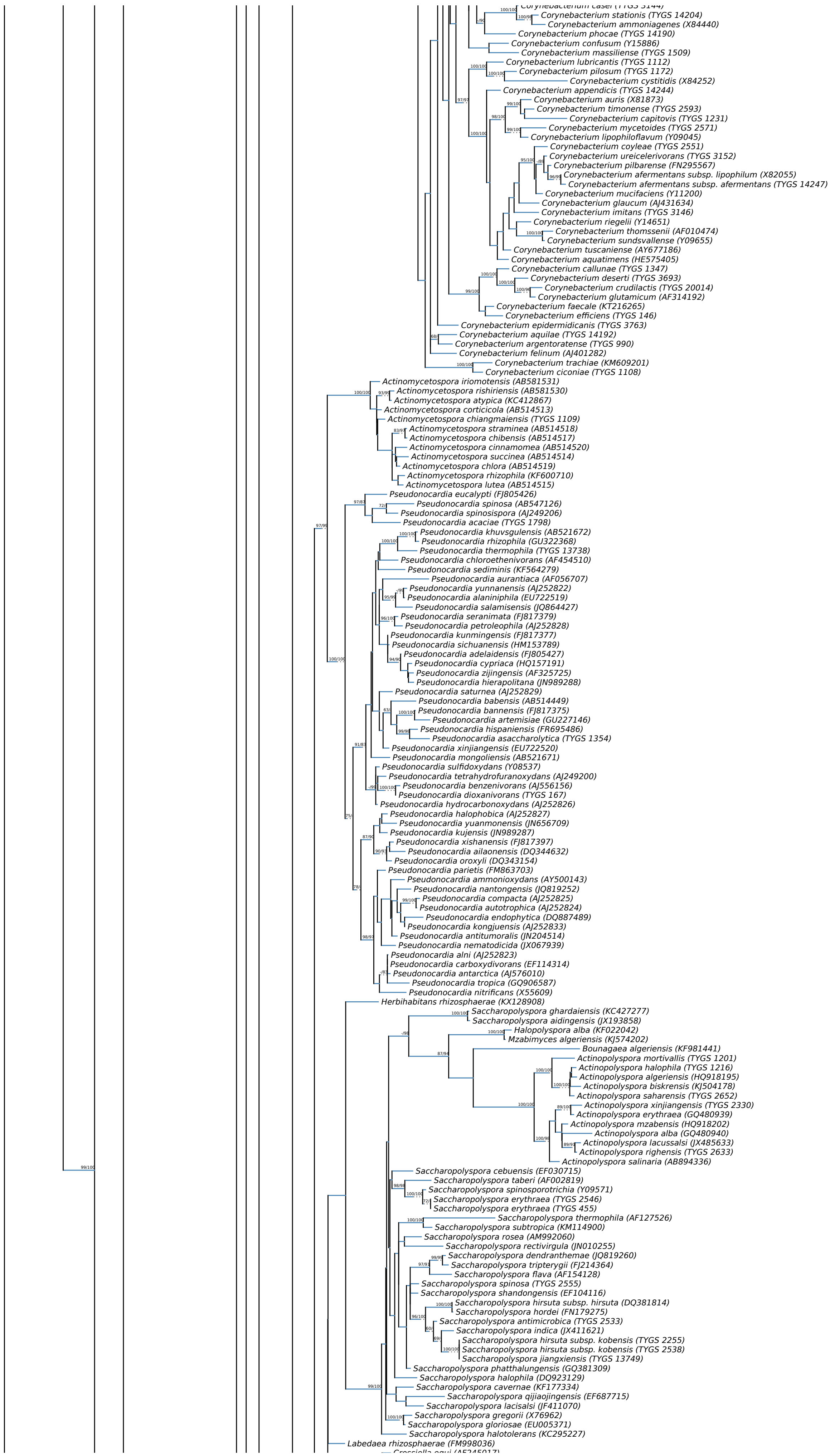

Corynebacterium stationis (TYGS 14204)
Corynebacterium ammoniagenes (X84440)
Corynebacterium phocae (TYGS 14190)
Corynebacterium confusum (Y15886)
Corynebacterium massiliense (TYGS 1509)
Corynebacterium lubricantis (TYGS 1112)
Corynebacterium pilosum (TYGS 1172)
Corynebacterium cystitidis (X84252)
Corynebacterium appendicis (TYGS 14244)
Corynebacterium auris (X81873)
Corynebacterium timonense (TYGS 2593)
Corynebacterium capitovis (TYGS 1231)
Corynebacterium mycetoides (TYGS 2571)
Corynebacterium lipophiloflavum (Y09045)
Corynebacterium coyleae (TYGS 2551)
Corynebacterium ureicelerivorans (TYGS 3152)
Corynebacterium pilbarense (FN295567)
Corynebacterium afermentans subsp. lipophilum (X82055)
Corynebacterium afermentans subsp. afermentans (TYGS 14247)
Corynebacterium mucifaciens (Y11200)
Corynebacterium glaucum (AJ431634)
Corynebacterium imitans (TYGS 3146)
Corynebacterium riegelii (Y14651)
Corynebacterium thomssenii (AF010474)
Corynebacterium sundsvallense (Y09655)
Corynebacterium tuscaniense (AY677186)
Corynebacterium aquatimens (HE575405)
Corynebacterium callunae (TYGS 1347)
Corynebacterium deserti (TYGS 3693)
Corynebacterium crudilactis (TYGS 20014)
Corynebacterium glutamicum (AF314192)
Corynebacterium faecale (KT216265)
Corynebacterium efficiens (TYGS 146)
Corynebacterium epidermidicanis (TYGS 3763)
Corynebacterium aquilae (TYGS 14192)
Corynebacterium argentoratense (TYGS 990)
Corynebacterium felinum (AJ401282)
Corynebacterium trachiae (KM609201)
Corynebacterium ciconiae (TYGS 1108)
Actinomycetospora iriomotensis (AB581531)
Actinomycetospora rishiriensis (AB581530)
Actinomycetospora atypica (KC412867)
Actinomycetospora corticicola (AB514513)
Actinomycetospora chiangmaiensis (TYGS 1109)
Actinomycetospora straminea (AB514518)
Actinomycetospora chibensis (AB514517)
Actinomycetospora cinnamomea (AB514520)
Actinomycetospora succinea (AB514514)
Actinomycetospora chlora (AB514519)
Actinomycetospora rhizophila (KF600710)
Actinomycetospora lutea (AB514515)
Pseudonocardia eucalypti (FJ805426)
Pseudonocardia spinosa (AB547126)
Pseudonocardia spinosispora (AJ249206)
Pseudonocardia acaciae (TYGS 1798)
Pseudonocardia khuvsgulensis (AB521672)
Pseudonocardia rhizophila (GU322368)
Pseudonocardia thermophila (TYGS 13738)
Pseudonocardia chloroethenivorans (AF454510)
Pseudonocardia sediminis (KF564279)
Pseudonocardia aurantiaca (AF056707)
Pseudonocardia yunnanensis (AJ252822)
Pseudonocardia alaniniphila (EU722519)
Pseudonocardia salamisensis (JQ864427)
Pseudonocardia seranimata (FJ817379)
Pseudonocardia petroleophila (AJ252828)
Pseudonocardia kunmingensis (FJ817377)
Pseudonocardia sichuanensis (HM153789)
Pseudonocardia adelaidensis (FJ805427)
Pseudonocardia cypriaca (HQ157191)
Pseudonocardia zijingensis (AF325725)
Pseudonocardia hierapolitana (JN989288)
Pseudonocardia saturnea (AJ252829)
Pseudonocardia babensis (AB514449)
Pseudonocardia bannensis (FJ817375)
Pseudonocardia artemisiae (GU227146)
Pseudonocardia hispaniensis (FR695486)
Pseudonocardia asaccharolytica (TYGS 1354)
Pseudonocardia xinjiangensis (EU722520)
Pseudonocardia mongoliensis (AB521671)
Pseudonocardia sulfidoxydans (Y08537)
Pseudonocardia tetrahydrofuranoxydans (AJ249200)
Pseudonocardia benzenivorans (AJ556156)
Pseudonocardia dioxanivorans (TYGS 167)
Pseudonocardia hydrocarbonoxydans (AJ252826)
Pseudonocardia halophobica (AJ252827)
Pseudonocardia yuanmonensis (JN656709)
Pseudonocardia kujensis (JN989287)
Pseudonocardia xishanensis (FJ817397)
Pseudonocardia ailaonensis (DQ344632)
Pseudonocardia oroxyli (DQ343154)
Pseudonocardia parietis (FM863703)
Pseudonocardia ammonioxydans (AY500143)
Pseudonocardia nantongensis (JQ819252)
Pseudonocardia compacta (AJ252825)
Pseudonocardia autotrophica (AJ252824)
Pseudonocardia endophytica (DQ887489)
Pseudonocardia kongjuensis (AJ252833)
Pseudonocardia antitumoralis (JN204514)
Pseudonocardia nematodicida (JX067939)
Pseudonocardia alni (AJ252823)
Pseudonocardia carboxydivorans (EF114314)
Pseudonocardia antarctica (AJ576010)
Pseudonocardia tropica (GQ906587)
Pseudonocardia nitrificans (X55609)
Herbihabitans rhizosphaerae (KX128908)
Saccharopolyspora ghardaiensis (KC427277)
Saccharopolyspora aidingensis (JX193858)
Halopolyspora alba (KF022042)
Mzabimyces algeriensis (KJ574202)
Bounagaea algeriensis (KF981441)
Actinopolyspora mortivallis (TYGS 1201)
Actinopolyspora halophila (TYGS 1216)
Actinopolyspora algeriensis (HQ918195)
Actinopolyspora biskrensis (KJ504178)
Actinopolyspora saharensis (TYGS 2652)
Actinopolyspora xinjiangensis (TYGS 2330)
Actinopolyspora erythraea (GQ480939)
Actinopolyspora mzabensis (HQ918202)
Actinopolyspora alba (GQ480940)
Actinopolyspora lacussalsi (JX485633)
Actinopolyspora righensis (TYGS 2633)
Actinopolyspora salinaria (AB894336)
Saccharopolyspora cebuensis (EF030715)
Saccharopolyspora taberi (AF002819)
Saccharopolyspora spinosporotrichia (Y09571)
Saccharopolyspora erythraea (TYGS 2546)
Saccharopolyspora erythraea (TYGS 455)
Saccharopolyspora thermophila (AF127526)
Saccharopolyspora subtropica (KM114900)
Saccharopolyspora rosea (AM992060)
Saccharopolyspora rectivirgula (JN010255)
Saccharopolyspora dendranthemae (JQ819260)
Saccharopolyspora tripterygii (FJ214364)
Saccharopolyspora flava (AF154128)
Saccharopolyspora spinosa (TYGS 2555)
Saccharopolyspora shandongensis (EF104116)
Saccharopolyspora hirsuta subsp. hirsuta (DQ381814)
Saccharopolyspora hordei (FN179275)
Saccharopolyspora antimicrobica (TYGS 2533)
Saccharopolyspora indica (JX411621)
Saccharopolyspora hirsuta subsp. kobensis (TYGS 2255)
Saccharopolyspora hirsuta subsp. kobensis (TYGS 2538)
Saccharopolyspora jiangxiensis (TYGS 13749)
Saccharopolyspora phatthalungensis (GQ381309)
Saccharopolyspora halophila (DQ923129)
Saccharopolyspora cavernae (KF177334)
Saccharopolyspora qijiaojingensis (EF687715)
Saccharopolyspora lacisalsi (JF411070)
Saccharopolyspora gregorii (X76962)
Saccharopolyspora gloriosae (EU005371)
Saccharopolyspora halotolerans (KC295227)
Labedaea rhizosphaerae (FM998036)

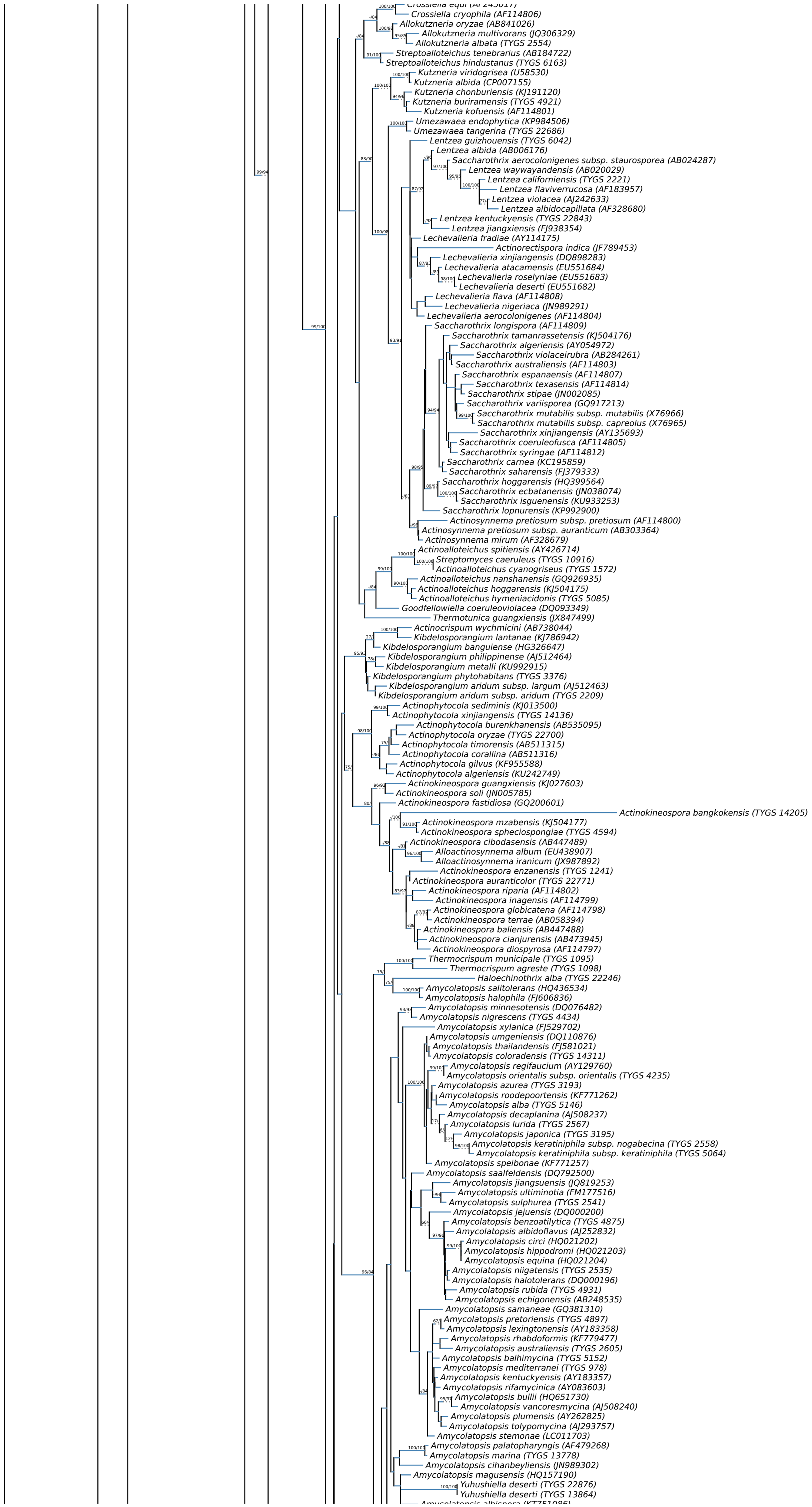
Crossiella cryophila (AF114806)
Allokutzneria oryzae (AB841026)
Allokutzneria multivorans (JQ306329)
Allokutzneria albata (TYGS 2554)
Streptoalloteichus tenebrarius (AB184722)
Streptoalloteichus hindustanus (TYGS 6163)
Kutzneria viridogrisea (U58530)
Kutzneria albida (CP007155)
Kutzneria chonburiensis (KJ191120)
Kutzneria buriramensis (TYGS 4921)
Kutzneria kofuensis (AF114801)
Umezawaea endophytica (KP984506)
Umezawaea tangerina (TYGS 22686)
Lentzea guizhouensis (TYGS 6042)
Lentzea albida (AB006176)
Saccharothrix aerocolonigenes subsp. staurosporea (AB024287)
Lentzea waywayandensis (AB020029)
Lentzea californiensis (TYGS 2221)
Lentzea flaviverrucosa (AF183957)
Lentzea violacea (AJ242633)
Lentzea albidocapillata (AF328680)
Lentzea kentuckyensis (TYGS 22843)
Lentzea jiangxiensis (FJ938354)
Lechevalieria fradiae (AY114175)
Actinorectispora indica (JF789453)
Lechevalieria xinjiangensis (DQ898283)
Lechevalieria atacamensis (EU551684)
Lechevalieria roselyniae (EU551683)
Lechevalieria deserti (EU551682)
Lechevalieria flava (AF114808)
Lechevalieria nigeriaca (JN989291)
Lechevalieria aerocolonigenes (AF114804)
Saccharothrix longispora (AF114809)
Saccharothrix tamanrassetensis (KJ504176)
Saccharothrix algeriensis (AY054972)
Saccharothrix violaceirubra (AB284261)
Saccharothrix australiensis (AF114803)
Saccharothrix espanaensis (AF114807)
Saccharothrix texasensis (AF114814)
Saccharothrix stipae (JN002085)
Saccharothrix variisporea (GQ917213)
Saccharothrix mutabilis subsp. mutabilis (X76966)
Saccharothrix mutabilis subsp. capreolus (X76965)
Saccharothrix xinjiangensis (AY135693)
Saccharothrix coeruleofusca (AF114805)
Saccharothrix syringae (AF114812)
Saccharothrix carnea (KC195859)
Saccharothrix saharensis (FJ379333)
Saccharothrix hoggarensis (HQ399564)
Saccharothrix ecbatanensis (JN038074)
Saccharothrix isguenensis (KU933253)
Saccharothrix lopnurensis (KP992900)
Actinosynnema pretiosum subsp. pretiosum (AF114800)
Actinosynnema pretiosum subsp. auranticum (AB303364)
Actinosynnema mirum (AF328679)
Actinoalloteichus spitiensis (AY426714)
Streptomyces caeruleus (TYGS 10916)
Actinoalloteichus cyanogriseus (TYGS 1572)
Actinoalloteichus nanshanensis (GQ926935)
Actinoalloteichus hoggarensis (KJ504175)
Actinoalloteichus hymeniacidonis (TYGS 5085)
Goodfellowiella coeruleoviolacea (DQ093349)
Thermotunica guangxiensis (JX847499)
Actinocrispum wychmicini (AB738044)
Kibdelosporangium lantanae (KJ786942)
Kibdelosporangium banguiense (HG326647)
Kibdelosporangium philippinense (AJ512464)
Kibdelosporangium metalli (KU992915)
Kibdelosporangium phytohabitans (TYGS 3376)
Kibdelosporangium aridum subsp. largum (AJ512463)
Kibdelosporangium aridum subsp. aridum (TYGS 2209)
Actinophytocola sediminis (KJ013500)
Actinophytocola xinjiangensis (TYGS 14136)
Actinophytocola burenkhanensis (AB535095)
Actinophytocola oryzae (TYGS 22700)
Actinophytocola timorensis (AB511315)
Actinophytocola corallina (AB511316)
Actinophytocola gilvus (KF955588)
Actinophytocola algeriensis (KU242749)
Actinokineospora guangxiensis (KJ027603)
Actinokineospora soli (JN005785)
Actinokineospora fastidiosa (GQ200601)
Actinokineospora bangkokensis (TYGS 14205)
Actinokineospora mzabensis (KJ504177)
Actinokineospora spheciospongiae (TYGS 4594)
Actinokineospora cibodasensis (AB447489)
Alloactinosynnema album (EU438907)
Alloactinosynnema iranicum (JX987892)
Actinokineospora enzanensis (TYGS 1241)
Actinokineospora auranticolor (TYGS 22771)
Actinokineospora riparia (AF114802)
Actinokineospora inagensis (AF114799)
Actinokineospora globicatena (AF114798)
Actinokineospora terrae (AB058394)
Actinokineospora baliensis (AB447488)
Actinokineospora cianjurensis (AB473945)
Actinokineospora diospyrosa (AF114797)
Thermocrispum municipale (TYGS 1095)
Thermocrispum agreste (TYGS 1098)
Haloechinothrix alba (TYGS 22246)
Amycolatopsis salitolerans (HQ436534)
Amycolatopsis halophila (FJ606836)
Amycolatopsis minnesotensis (DQ076482)
Amycolatopsis nigrescens (TYGS 4434)
Amycolatopsis xylanica (FJ529702)
Amycolatopsis umgeniensis (DQ110876)
Amycolatopsis thailandensis (FJ581021)
Amycolatopsis coloradensis (TYGS 14311)
Amycolatopsis regifaucium (AY129760)
Amycolatopsis orientalis subsp. orientalis (TYGS 4235)
Amycolatopsis azurea (TYGS 3193)
Amycolatopsis roodepoortensis (KF771262)
Amycolatopsis alba (TYGS 5146)
Amycolatopsis decaplanina (AJ508237)
Amycolatopsis lurida (TYGS 2567)
Amycolatopsis japonica (TYGS 3195)
Amycolatopsis keratiniphila subsp. nogabecina (TYGS 2558)
Amycolatopsis keratiniphila subsp. keratiniphila (TYGS 5064)
Amycolatopsis speibonae (KF771257)
Amycolatopsis saalfeldensis (DQ792500)
Amycolatopsis jiangsuensis (JQ819253)
Amycolatopsis ultiminotia (FM177516)
Amycolatopsis sulphurea (TYGS 2541)
Amycolatopsis jejuensis (DQ000200)
Amycolatopsis benzoatilytica (TYGS 4875)
Amycolatopsis albidoflavus (AJ252832)
Amycolatopsis circi (HQ021202)
Amycolatopsis hippodromi (HQ021203)
Amycolatopsis equina (HQ021204)
Amycolatopsis niigatensis (TYGS 2535)
Amycolatopsis halotolerans (DQ000196)
Amycolatopsis rubida (TYGS 4931)
Amycolatopsis echigonensis (AB248535)
Amycolatopsis samaneae (GQ381310)
Amycolatopsis pretoriensis (TYGS 4897)
Amycolatopsis lexingtonensis (AY183358)
Amycolatopsis rhabdoformis (KF779477)
Amycolatopsis australiensis (TYGS 2605)
Amycolatopsis balhimycina (TYGS 5152)
Amycolatopsis mediterranei (TYGS 978)
Amycolatopsis kentuckyensis (AY183357)
Amycolatopsis rifamycinica (AY083603)
Amycolatopsis bullii (HQ651730)
Amycolatopsis vancoresmycina (AJ508240)
Amycolatopsis plumensis (AY262825)
Amycolatopsis tolypomycina (AJ293757)
Amycolatopsis stemonae (LC011703)
Amycolatopsis palatopharyngis (AF479268)
Amycolatopsis marina (TYGS 13778)
Amycolatopsis cihanbeyliensis (JN989302)
Amycolatopsis magusensis (HQ157190)
Yuhushiella deserti (TYGS 22876)
Yuhushiella deserti (TYGS 13864)

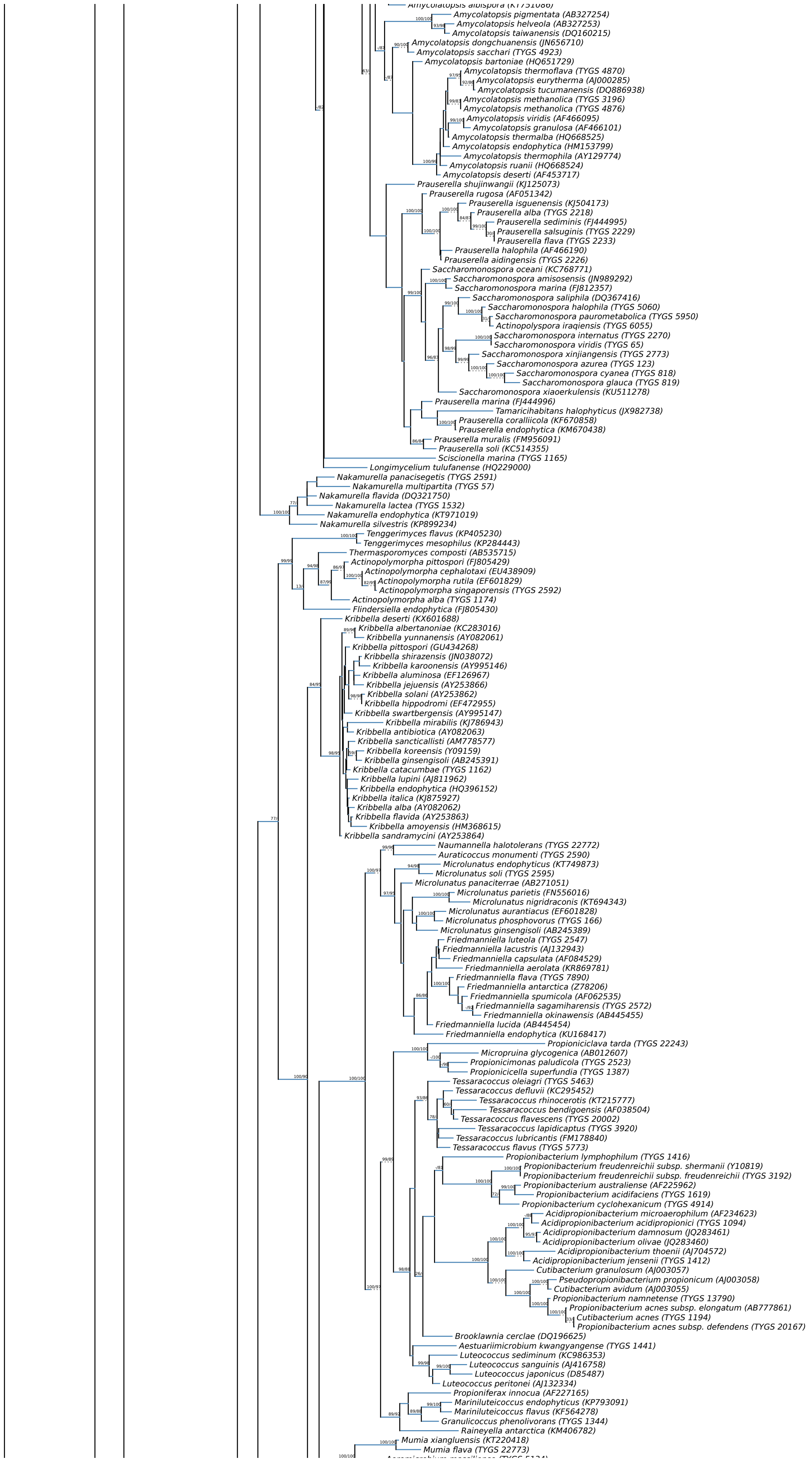
Amycolatopsis pigmentata (AB327254)
Amycolatopsis helveola (AB327253)
Amycolatopsis taiwanensis (DQ160215)
Amycolatopsis dongchuanensis (JN656710)
Amycolatopsis sacchari (TYGS 4923)
Amycolatopsis bartoniae (HQ651729)
Amycolatopsis thermoflava (TYGS 4870)
Amycolatopsis eurytherma (AJ000285)
Amycolatopsis tucumanensis (DQ886938)
Amycolatopsis methanolica (TYGS 3196)
Amycolatopsis methanolica (TYGS 4876)
Amycolatopsis viridis (AF466095)
Amycolatopsis granulosa (AF466101)
Amycolatopsis thermalba (HQ668525)
Amycolatopsis endophytica (HM153799)
Amycolatopsis thermophila (AY129774)
Amycolatopsis ruanii (HQ668524)
Amycolatopsis deserti (AF453717)
Prauserella shujinwangii (KJ125073)
Prauserella rugosa (AF051342)
Prauserella isguenensis (KJ504173)
Prauserella alba (TYGS 2218)
Prauserella sediminis (FJ444995)
Prauserella salsuginis (TYGS 2229)
Prauserella flava (TYGS 2233)
Prauserella halophila (AF466190)
Prauserella aidingensis (TYGS 2226)
Saccharomonospora oceani (KC768771)
Saccharomonospora amisosensis (JN989292)
Saccharomonospora marina (FJ812357)
Saccharomonospora saliphila (DQ367416)
Saccharomonospora halophila (TYGS 5060)
Saccharomonospora paurometabolica (TYGS 5950)
Actinopolyspora iraqiensis (TYGS 6055)
Saccharomonospora internatus (TYGS 2270)
Saccharomonospora viridis (TYGS 65)
Saccharomonospora xinjiangensis (TYGS 2773)
Saccharomonospora azurea (TYGS 123)
Saccharomonospora cyanea (TYGS 818)
Saccharomonospora glauca (TYGS 819)
Saccharomonospora xiaoerkulensis (KU511278)
Prauserella marina (FJ444996)
Tamaricihabitans halophyticus (JX982738)
Prauserella coralliicola (KF670858)
Prauserella endophytica (KM670438)
Prauserella muralis (FM956091)
Prauserella soli (KC514355)
Sciscionella marina (TYGS 1165)
Longimycelium tulufanense (HQ229000)
Nakamurella panacisegetis (TYGS 2591)
Nakamurella multipartita (TYGS 57)
Nakamurella flavida (DQ321750)
Nakamurella lactea (TYGS 1532)
Nakamurella endophytica (KT971019)
Nakamurella silvestris (KP899234)
Tenggerimyces flavus (KP405230)
Tenggerimyces mesophilus (KP284443)
Thermasporomyces composti (AB535715)
Actinopolymorpha pittospori (FJ805429)
Actinopolymorpha cephalotaxi (EU438909)
Actinopolymorpha rutila (EF601829)
Actinopolymorpha singaporensis (TYGS 2592)
Actinopolymorpha alba (TYGS 1174)
Flindersiella endophytica (FJ805430)
Kribbella deserti (KX601688)
Kribbella albertanoniae (KC283016)
Kribbella yunnanensis (AY082061)
Kribbella pittospori (GU434268)
Kribbella shirazensis (JN038072)
Kribbella karoonensis (AY995146)
Kribbella aluminosa (EF126967)
Kribbella jejuensis (AY253866)
Kribbella solani (AY253862)
Kribbella hippodromi (EF472955)
Kribbella swartbergensis (AY995147)
Kribbella mirabilis (KJ786943)
Kribbella antibiotica (AY082063)
Kribbella sancticallisti (AM778577)
Kribbella koreensis (Y09159)
Kribbella ginsengisoli (AB245391)
Kribbella catacumbae (TYGS 1162)
Kribbella lupini (AJ811962)
Kribbella endophytica (HQ396152)
Kribbella italica (KJ875927)
Kribbella alba (AY082062)
Kribbella flavida (AY253863)
Kribbella amoyensis (HM368615)
Kribbella sandramycini (AY253864)
Naumannella halotolerans (TYGS 22772)
Auraticoccus monumenti (TYGS 2590)
Microlunatus endophyticus (KT749873)
Microlunatus soli (TYGS 2595)
Microlunatus panaciterrae (AB271051)
Microlunatus parietis (FN556016)
Microlunatus nigridraconis (KT694343)
Microlunatus aurantiacus (EF601828)
Microlunatus phosphovorus (TYGS 166)
Microlunatus ginsengisoli (AB245389)
Friedmanniella luteola (TYGS 2547)
Friedmanniella lacustris (AJ132943)
Friedmanniella capsulata (AF084529)
Friedmanniella aerolata (KR869781)
Friedmanniella flava (TYGS 7890)
Friedmanniella antarctica (Z78206)
Friedmanniella spumicola (AF062535)
Friedmanniella sagamiharensis (TYGS 2572)
Friedmanniella okinawensis (AB445455)
Friedmanniella lucida (AB445454)
Friedmanniella endophytica (KU168417)
Propioniciclava tarda (TYGS 22243)
Micropruina glycogenica (AB012607)
Propionicimonas paludicola (TYGS 2523)
Propionicicella superfundia (TYGS 1387)
Tessaracoccus oleiagri (TYGS 5463)
Tessaracoccus defluvii (KC295452)
Tessaracoccus rhinocerotis (KT215777)
Tessaracoccus bendigoensis (AF038504)
Tessaracoccus flavescens (TYGS 20002)
Tessaracoccus lapidicaptus (TYGS 3920)
Tessaracoccus lubricantis (FM178840)
Tessaracoccus flavus (TYGS 5773)
Propionibacterium lymphophilum (TYGS 1416)
Propionibacterium freudenreichii subsp. shermanii (Y10819)
Propionibacterium freudenreichii subsp. freudenreichii (TYGS 3192)
Propionibacterium australiense (AF225962)
Propionibacterium acidifaciens (TYGS 1619)
Propionibacterium cyclohexanicum (TYGS 4914)
Acidipropionibacterium microaerophilum (AF234623)
Acidipropionibacterium acidipropionici (TYGS 1094)
Acidipropionibacterium damnosum (JQ283461)
Acidipropionibacterium olivae (JQ283460)
Acidipropionibacterium thoenii (AJ704572)
Acidipropionibacterium jensenii (TYGS 1412)
Cutibacterium granulosum (AJ003057)
Pseudopropionibacterium propionicum (AJ003058)
Cutibacterium avidum (AJ003055)
Propionibacterium namnetense (TYGS 13790)
Propionibacterium acnes subsp. elongatum (AB777861)
Cutibacterium acnes (TYGS 1194)
Propionibacterium acnes subsp. defendens (TYGS 20167)
Brooklawnia cerclae (DQ196625)
Aestuariimicrobium kwangyangense (TYGS 1441)
Luteococcus sediminum (KC986353)
Luteococcus sanguinis (AJ416758)
Luteococcus japonicus (D85487)
Luteococcus peritonei (AJ132334)
Propioniferax innocua (AF227165)
Mariniluteicoccus endophyticus (KP793091)
Mariniluteicoccus flavus (KF564278)
Granulicoccus phenolivorans (TYGS 1344)
Raineyella antarctica (KM406782)
Mumia xiangluensis (KT220418)
Mumia flava (TYGS 22773)

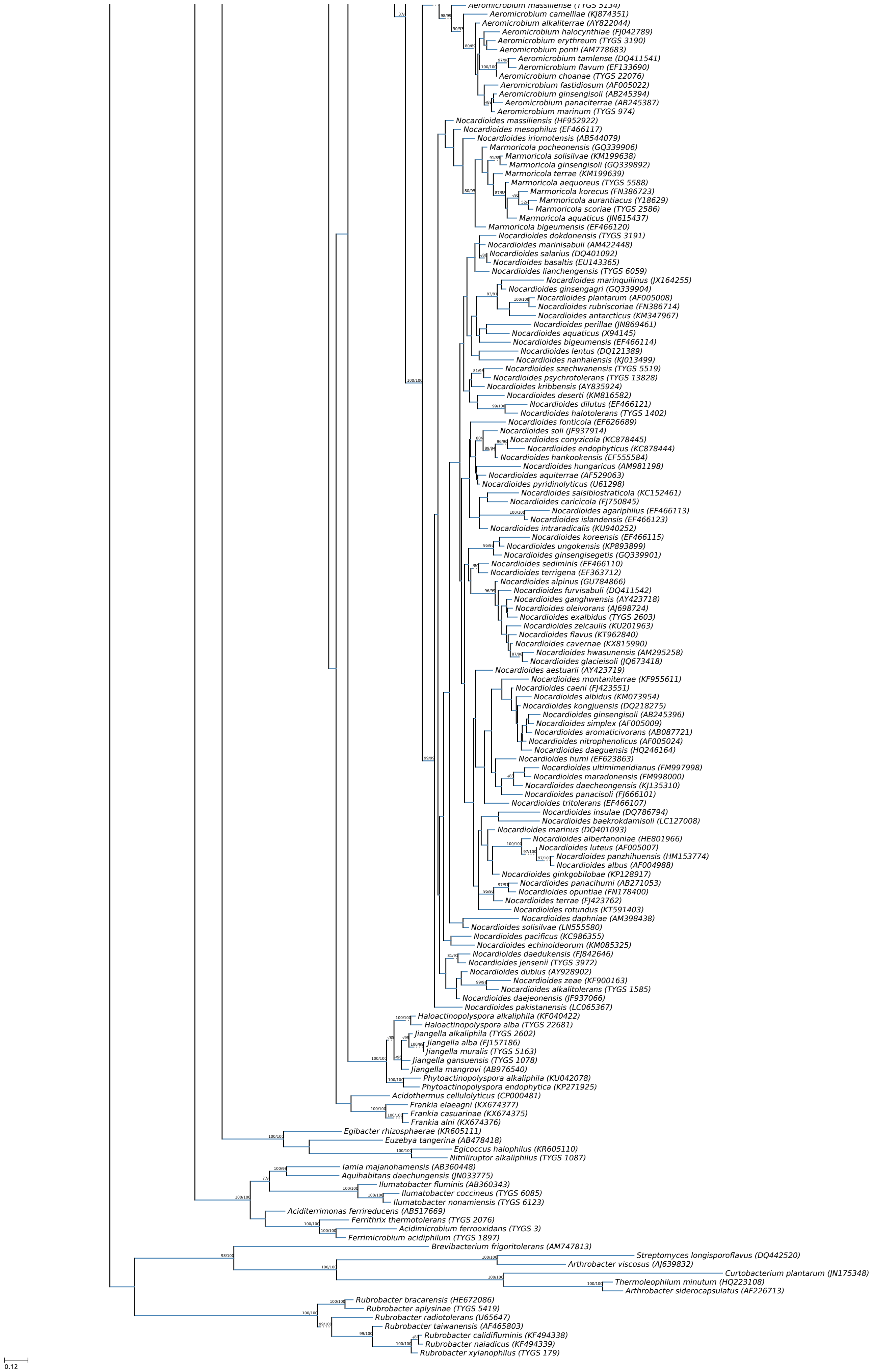

Aeromicrobium massiliense (TYGS 5134)
Aeromicrobium camelliae (KJ874351)
Aeromicrobium alkaliterrae (AY822044)
Aeromicrobium halocynthiae (FJ042789)
Aeromicrobium erythreum (TYGS 3190)
Aeromicrobium ponti (AM778683)
Aeromicrobium tamlense (DQ411541)
Aeromicrobium flavum (EF133690)
Aeromicrobium choanae (TYGS 22076)
Aeromicrobium fastidiosum (AF005022)
Aeromicrobium ginsengisoli (AB245394)
Aeromicrobium panaciterrae (AB245387)
Aeromicrobium marinum (TYGS 974)
Nocardioides massiliensis (HF952922)
Nocardioides mesophilus (EF466117)
Nocardioides iriomotensis (AB544079)
Marmoricola pocheonensis (GQ339906)
Marmoricola solisilvae (KM199638)
Marmoricola ginsengisoli (GQ339892)
Marmoricola terrae (KM199639)
Marmoricola aequoreus (TYGS 5588)
Marmoricola korecus (FN386723)
Marmoricola aurantiacus (Y18629)
Marmoricola scoriae (TYGS 2586)
Marmoricola aquaticus (JN615437)
Marmoricola bigeumensis (EF466120)
Nocardioides dokdonensis (TYGS 3191)
Nocardioides marinisabuli (AM422448)
Nocardioides salarius (DQ401092)
Nocardioides basaltis (EU143365)
Nocardioides lianchengensis (TYGS 6059)
Nocardioides marinquilinus (JX164255)
Nocardioides ginsengagri (GQ339904)
Nocardioides plantarum (AF005008)
Nocardioides rubriscoriae (FN386714)
Nocardioides antarcticus (KM347967)
Nocardioides perillae (JN869461)
Nocardioides aquaticus (X94145)
Nocardioides bigeumensis (EF466114)
Nocardioides lentus (DQ121389)
Nocardioides nanhaiensis (KJ013499)
Nocardioides szechwanensis (TYGS 5519)
Nocardioides psychrotolerans (TYGS 13828)
Nocardioides kribbensis (AY835924)
Nocardioides deserti (KM816582)
Nocardioides dilutus (EF466121)
Nocardioides halotolerans (TYGS 1402)
Nocardioides fonticola (EF626689)
Nocardioides soli (JF937914)
Nocardioides conyzicola (KC878445)
Nocardioides endophyticus (KC878444)
Nocardioides hankookensis (EF555584)
Nocardioides hungaricus (AM981198)
Nocardioides aquiterrae (AF529063)
Nocardioides pyridinolyticus (U61298)
Nocardioides salsibiostraticola (KC152461)
Nocardioides caricicola (FJ750845)
Nocardioides agariphilus (EF466113)
Nocardioides islandensis (EF466123)
Nocardioides intraradicalis (KU940252)
Nocardioides koreensis (EF466115)
Nocardioides ungokensis (KP893899)
Nocardioides ginsengisegetis (GQ339901)
Nocardioides sediminis (EF466110)
Nocardioides terrigena (EF363712)
Nocardioides alpinus (GU784866)
Nocardioides furvisabuli (DQ411542)
Nocardioides ganghwensis (AY423718)
Nocardioides oleivorans (AJ698724)
Nocardioides exalbidus (TYGS 2603)
Nocardioides zeicaulis (KU201963)
Nocardioides flavus (KT962840)
Nocardioides cavernae (KX815990)
Nocardioides hwasunensis (AM295258)
Nocardioides glacieisoli (JQ673418)
Nocardioides aestuarii (AY423719)
Nocardioides montaniterrae (KF955611)
Nocardioides caeni (FJ423551)
Nocardioides albidus (KM073954)
Nocardioides kongjuensis (DQ218275)
Nocardioides ginsengisoli (AB245396)
Nocardioides simplex (AF005009)
Nocardioides aromaticivorans (AB087721)
Nocardioides nitrophenolicus (AF005024)
Nocardioides daeguensis (HQ246164)
Nocardioides humi (EF623863)
Nocardioides ultimimeridianus (FM997998)
Nocardioides maradonensis (FM998000)
Nocardioides daecheongensis (KJ135310)
Nocardioides panacisoli (FJ666101)
Nocardioides tritolerans (EF466107)
Nocardioides insulae (DQ786794)
Nocardioides baekrokdamisoli (LC127008)
Nocardioides marinus (DQ401093)
Nocardioides albertanoniae (HE801966)
Nocardioides luteus (AF005007)
Nocardioides panzhihuensis (HM153774)
Nocardioides albus (AF004988)
Nocardioides ginkgobilobae (KP128917)
Nocardioides panacihumi (AB271053)
Nocardioides opuntiae (FN178400)
Nocardioides terrae (FJ423762)
Nocardioides rotundus (KT591403)
Nocardioides daphniae (AM398438)
Nocardioides solisilvae (LN555580)
Nocardioides pacificus (KC986355)
Nocardioides echinoideorum (KM085325)
Nocardioides daedukensis (FJ842646)
Nocardioides jensenii (TYGS 3972)
Nocardioides dubius (AY928902)
Nocardioides zeae (KF900163)
Nocardioides alkalitolerans (TYGS 1585)
Nocardioides daejeonensis (JF937066)
Nocardioides pakistanensis (LC065367)
Haloactinopolyspora alkaliphila (KF040422)
Haloactinopolyspora alba (TYGS 22681)
Jiangella alkaliphila (TYGS 2602)
Jiangella alba (FJ157186)
Jiangella muralis (TYGS 5163)
Jiangella gansuensis (TYGS 1078)
Jiangella mangrovi (AB976540)
Phytoactinopolyspora alkaliphila (KU042078)
Phytoactinopolyspora endophytica (KP271925)
Acidothermus cellulolyticus (CP000481)
Frankia elaeagni (KX674377)
Frankia casuarinae (KX674375)
Frankia alni (KX674376)
Egibacter rhizosphaerae (KR605111)
Euzebya tangerina (AB478418)
Egicoccus halophilus (KR605110)
Nitriliruptor alkaliphilus (TYGS 1087)
Iamia majanohamensis (AB360448)
Aquihabitans daechungensis (JN033775)
Ilumatobacter fluminis (AB360343)
Ilumatobacter coccineus (TYGS 6085)
Ilumatobacter nonamiensis (TYGS 6123)
Aciditerrimonas ferrireducens (AB517669)
Ferrithrix thermotolerans (TYGS 2076)
Acidimicrobium ferrooxidans (TYGS 3)
Ferrimicrobium acidiphilum (TYGS 1897)
Brevibacterium frigoritolerans (AM747813)
Streptomyces longisporoflavus (DQ442520)
Arthrobacter viscosus (AJ639832)
Curtobacterium plantarum (JN175348)
Thermoleophilum minutum (HQ223108)
Arthrobacter siderocapsulatus (AF226713)
Rubrobacter bracarensis (HE672086)
Rubrobacter aplysinae (TYGS 5419)
Rubrobacter radiotolerans (U65647)
Rubrobacter taiwanensis (AF465803)
Rubrobacter calidifluminis (KF494338)
Rubrobacter naiadicus (KF494339)
Rubrobacter xylanophilus (TYGS 179)
0.12

Figure 2: Backbone-constrained comprehensive 16S rRNA gene ML and MP tree (CCT). Dotted parts of branches are filled in to allow proper placement of bootstrap values and are not part of the actual branch length.

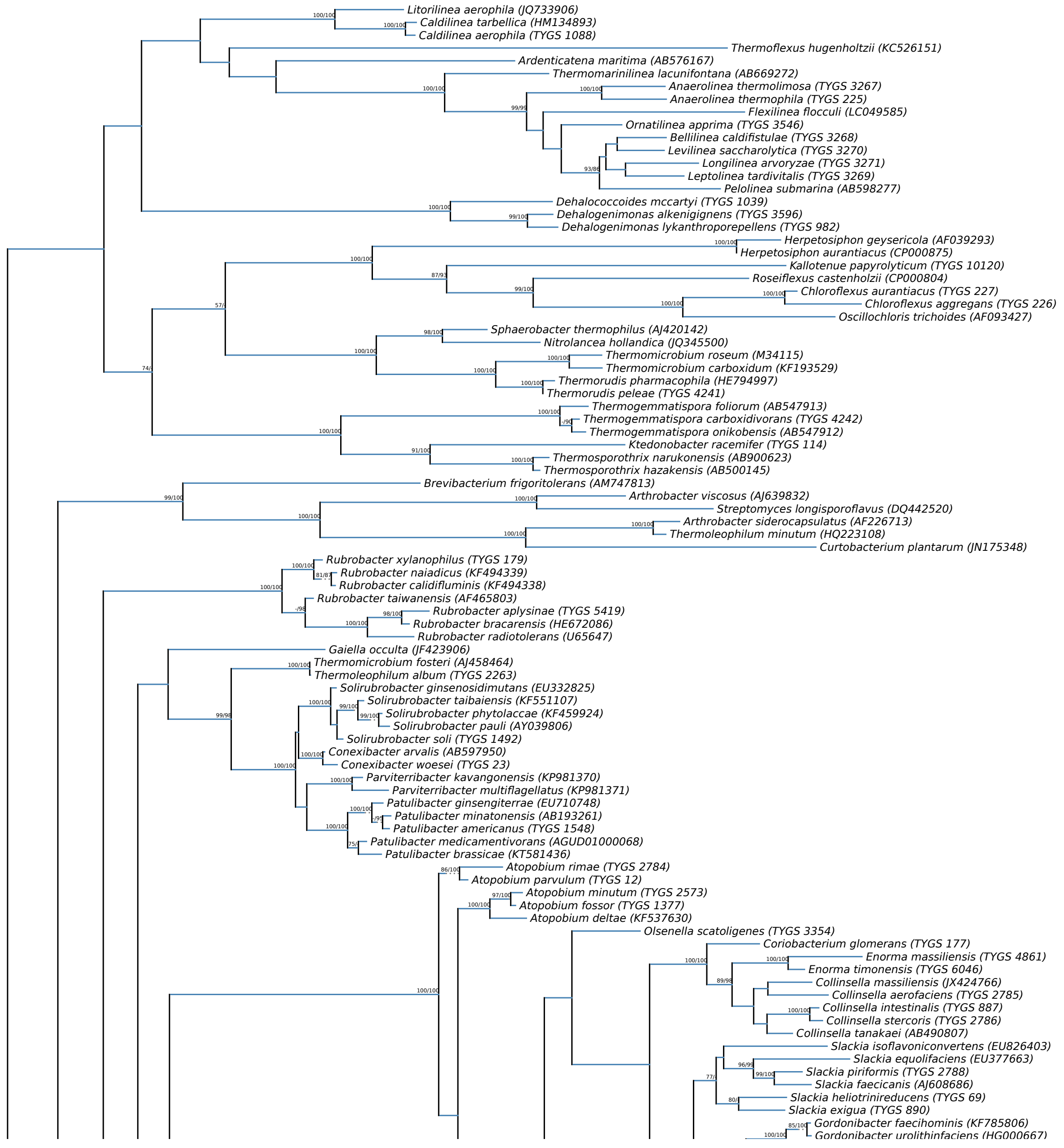
Litorilinea aerophila (JQ733906)
Caldilinea tarbellica (HM134893)
Caldilinea aerophila (TYGS 1088)
Thermoflexus hugenholtzii (KC526151)
Ardenticatena maritima (AB576167)
Thermomarinilinea lacunifontana (AB669272)
Anaerolinea thermolimosa (TYGS 3267)
Anaerolinea thermophila (TYGS 225)
Flexilinea flocculi (LC049585)
Ornatilinea apprima (TYGS 3546)
Bellilinea caldifistulae (TYGS 3268)
Levilinea saccharolytica (TYGS 3270)
Longilinea arvoryzae (TYGS 3271)
Leptolinea tardivitalis (TYGS 3269)
Pelolinea submarina (AB598277)
Dehalococcoides mccartyi (TYGS 1039)
Dehalogenimonas alkenigignens (TYGS 3596)
Dehalogenimonas lykanthroporepellens (TYGS 982)
Herpetosiphon geysericola (AF039293)
Herpetosiphon aurantiacus (CP000875)
Kallotenue papyrolyticum (TYGS 10120)
Roseiflexus castenholzii (CP000804)
Chloroflexus aurantiacus (TYGS 227)
Chloroflexus aggregans (TYGS 226)
Oscillochloris trichoides (AF093427)
Sphaerobacter thermophilus (AJ420142)
Nitrolancea hollandica (JQ345500)
Thermomicrobium roseum (M34115)
Thermomicrobium carboxidum (KF193529)
Thermorudis pharmacophila (HE794997)
Thermorudis peleae (TYGS 4241)
Thermogemmatispora foliorum (AB547913)
Thermogemmatispora carboxidivorans (TYGS 4242)
Thermogemmatispora onikobensis (AB547912)
Ktedonobacter racemifer (TYGS 114)
Thermosporothrix narukonensis (AB900623)
Thermosporothrix hazakensis (AB500145)
Brevibacterium frigoritolerans (AM747813)
Arthrobacter viscosus (AJ639832)
Streptomyces longisporoflavus (DQ442520)
Arthrobacter siderocapsulatus (AF226713)
Thermoleophilum minutum (HQ223108)
Curtobacterium plantarum (JN175348)
Rubrobacter xylanophilus (TYGS 179)
Rubrobacter naiadicus (KF494339)
Rubrobacter calidifluminis (KF494338)
Rubrobacter taiwanensis (AF465803)
Rubrobacter aplysinae (TYGS 5419)
Rubrobacter bracarensis (HE672086)
Rubrobacter radiotolerans (U65647)
Gaiella occulta (JF423906)
Thermomicrobium fosteri (AJ458464)
Thermoleophilum album (TYGS 2263)
Solirubrobacter ginsenosidimutans (EU332825)
Solirubrobacter taibaiensis (KF551107)
Solirubrobacter phytolaccae (KF459924)
Solirubrobacter pauli (AY039806)
Solirubrobacter soli (TYGS 1492)
Conexibacter arvalis (AB597950)
Conexibacter woesei (TYGS 23)
Parviterribacter kavangonensis (KP981370)
Parviterribacter multiflagellatus (KP981371)
Patulibacter ginsengiterrae (EU710748)
Patulibacter minatonensis (AB193261)
Patulibacter americanus (TYGS 1548)
Patulibacter medicamentivorans (AGUD01000068)
Patulibacter brassicae (KT581436)
Atopobium rimae (TYGS 2784)
Atopobium parvulum (TYGS 12)
Atopobium minutum (TYGS 2573)
Atopobium fossor (TYGS 1377)
Atopobium deltae (KF537630)
Olsenella scatoligenes (TYGS 3354)
Coriobacterium glomerans (TYGS 177)
Enorma massiliensis (TYGS 4861)
Enorma timonensis (TYGS 6046)
Collinsella massiliensis (JX424766)
Collinsella aerofaciens (TYGS 2785)
Collinsella intestinalis (TYGS 887)
Collinsella stercoris (TYGS 2786)
Collinsella tanakaei (AB490807)
Slackia isoflavoniconvertens (EU826403)
Slackia equolifaciens (EU377663)
Slackia piriformis (TYGS 2788)
Slackia faecicanis (AJ608686)
Slackia heliotrinireducens (TYGS 69)
Slackia exigua (TYGS 890)
Gordonibacter faecihominis (KF785806)
Gordonibacter urolithinfaciens (HG000667)

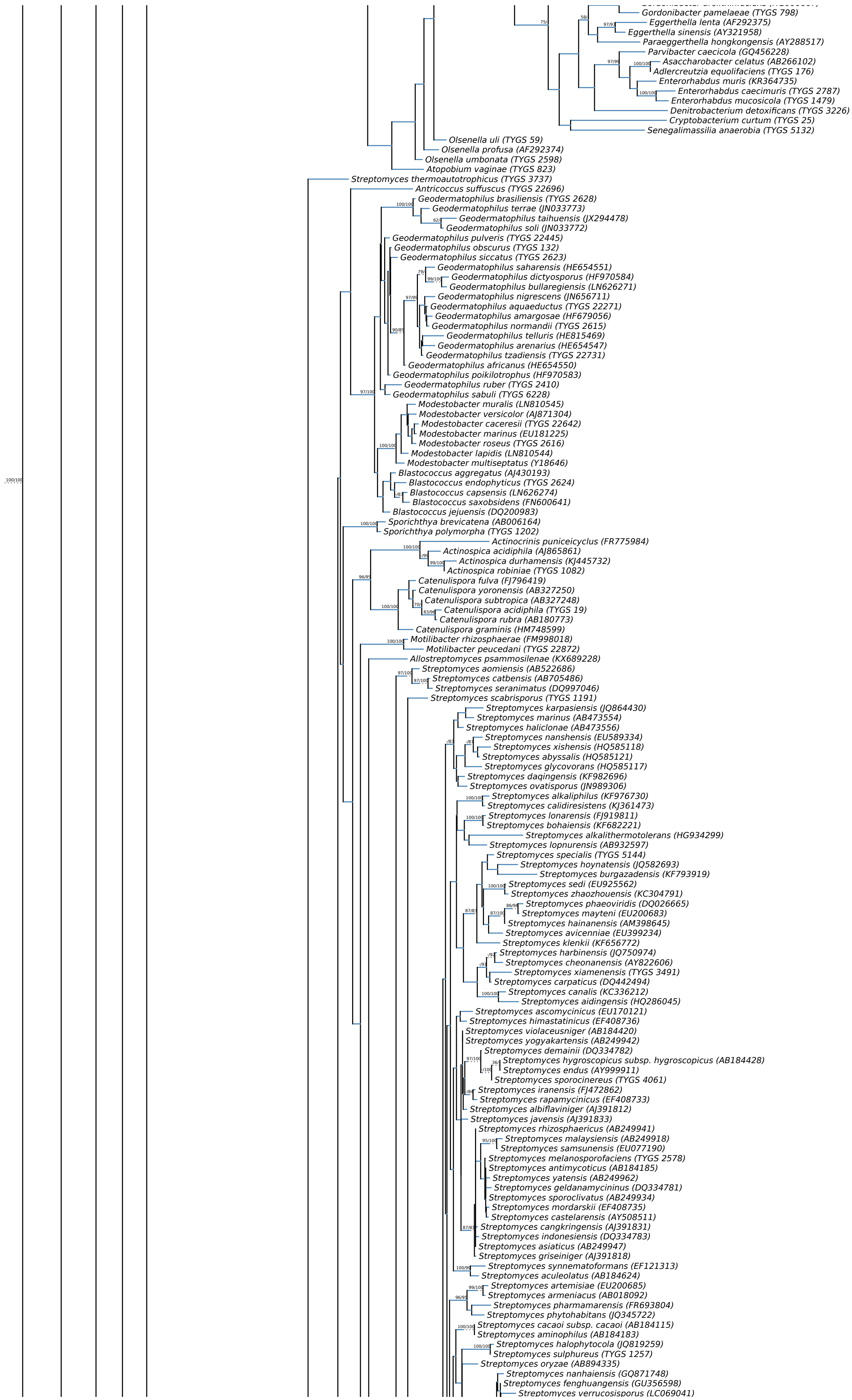

Gordonibacter pamelaeae (TYGS 798)
Eggerthella lenta (AF292375)
Eggerthella sinensis (AY321958)
Paraeggerthella hongkongensis (AY288517)
Parvibacter caecicola (GQ456228)
Asaccharobacter celatus (AB266102)
Adlercreutzia equolifaciens (TYGS 176)
Enterorhabdus muris (KR364735)
Enterorhabdus caecimuris (TYGS 2787)
Enterorhabdus mucosicola (TYGS 1479)
Denitrobacterium detoxificans (TYGS 3226)
Cryptobacterium curtum (TYGS 25)
Senegalimassilia anaerobia (TYGS 5132)
Olsenella uli (TYGS 59)
Olsenella profusa (AF292374)
Olsenella umbonata (TYGS 2598)
Atopobium vaginae (TYGS 823)
Streptomyces thermoautotrophicus (TYGS 3737)
Antricoccus suffuscus (TYGS 22696)
Geodermatophilus brasiliensis (TYGS 2628)
Geodermatophilus terrae (JN033773)
Geodermatophilus taihuensis (JX294478)
Geodermatophilus soli (JN033772)
Geodermatophilus pulveris (TYGS 22445)
Geodermatophilus obscurus (TYGS 132)
Geodermatophilus siccatus (TYGS 2623)
Geodermatophilus saharensis (HE654551)
Geodermatophilus dictyosporus (HF970584)
Geodermatophilus bullaregiensis (LN626271)
Geodermatophilus nigrescens (JN656711)
Geodermatophilus aquaeductus (TYGS 22271)
Geodermatophilus amargosae (HF679056)
Geodermatophilus normandii (TYGS 2615)
Geodermatophilus telluris (HE815469)
Geodermatophilus arenarius (HE654547)
Geodermatophilus tzadiensis (TYGS 22731)
Geodermatophilus africanus (HE654550)
Geodermatophilus poikilotrophus (HF970583)
Geodermatophilus ruber (TYGS 2410)
Geodermatophilus sabuli (TYGS 6228)
Modestobacter muralis (LN810545)
Modestobacter versicolor (AJ871304)
Modestobacter caceresii (TYGS 22642)
Modestobacter marinus (EU181225)
Modestobacter roseus (TYGS 2616)
Modestobacter lapidis (LN810544)
Modestobacter multiseptatus (Y18646)
Blastococcus aggregatus (AJ430193)
Blastococcus endophyticus (TYGS 2624)
Blastococcus capsensis (LN626274)
Blastococcus saxobsidens (FN600641)
Blastococcus jejuensis (DQ200983)
Sporichthya brevicatena (AB006164)
Sporichthya polymorpha (TYGS 1202)
Actinocrinis puniceicyclus (FR775984)
Actinospica acidiphila (AJ865861)
Actinospica durhamensis (KJ445732)
Actinospica robiniae (TYGS 1082)
Catenulispora fulva (FJ796419)
Catenulispora yoronensis (AB327250)
Catenulispora subtropica (AB327248)
Catenulispora acidiphila (TYGS 19)
Catenulispora rubra (AB180773)
Catenulispora graminis (HM748599)
Motilibacter rhizosphaerae (FM998018)
Motilibacter peucedani (TYGS 22872)
Allostreptomyces psammosilenae (KX689228)
Streptomyces aomiensis (AB522686)
Streptomyces catbensis (AB705486)
Streptomyces seranimatus (DQ997046)
Streptomyces scabrisporus (TYGS 1191)
Streptomyces karpasiensis (JQ864430)
Streptomyces marinus (AB473554)
Streptomyces haliclonae (AB473556)
Streptomyces nanshensis (EU589334)
Streptomyces xishensis (HQ585118)
Streptomyces abyssalis (HQ585121)
Streptomyces glycovorans (HQ585117)
Streptomyces daqingensis (KF982696)
Streptomyces ovatisporus (JN989306)
Streptomyces alkaliphilus (KF976730)
Streptomyces calidiresistens (KJ361473)
Streptomyces lonarensis (FJ919811)
Streptomyces bohaiensis (KF682221)
Streptomyces alkalithermotolerans (HG934299)
Streptomyces lopnurensis (AB932597)
Streptomyces specialis (TYGS 5144)
Streptomyces hoynatensis (JQ582693)
Streptomyces burgazadensis (KF793919)
Streptomyces sedi (EU925562)
Streptomyces zhaozhouensis (KC304791)
Streptomyces phaeoviridis (DQ026665)
Streptomyces mayteni (EU200683)
Streptomyces hainanensis (AM398645)
Streptomyces avicenniae (EU399234)
Streptomyces klenkii (KF656772)
Streptomyces harbinensis (JQ750974)
Streptomyces cheonanensis (AY822606)
Streptomyces xiamenensis (TYGS 3491)
Streptomyces carpaticus (DQ442494)
Streptomyces canalis (KC336212)
Streptomyces aidingensis (HQ286045)
Streptomyces ascomycinicus (EU170121)
Streptomyces himastatinicus (EF408736)
Streptomyces violaceusniger (AB184420)
Streptomyces yogyakartensis (AB249942)
Streptomyces demainii (DQ334782)
Streptomyces hygroscopicus subsp. hygroscopicus (AB184428)
Streptomyces endus (AY999911)
Streptomyces sporocinereus (TYGS 4061)
Streptomyces iranensis (FJ472862)
Streptomyces rapamycinicus (EF408733)
Streptomyces albiflaviniger (AJ391812)
Streptomyces javensis (AJ391833)
Streptomyces rhizosphaericus (AB249941)
Streptomyces malaysiensis (AB249918)
Streptomyces samsunensis (EU077190)
Streptomyces melanosporofaciens (TYGS 2578)
Streptomyces antimycoticus (AB184185)
Streptomyces yatensis (AB249962)
Streptomyces geldanamycininus (DQ334781)
Streptomyces sporoclivatus (AB249934)
Streptomyces mordarskii (EF408735)
Streptomyces castelarensis (AY508511)
Streptomyces cangkringensis (AJ391831)
Streptomyces indonesiensis (DQ334783)
Streptomyces asiaticus (AB249947)
Streptomyces griseiniger (AJ391818)
Streptomyces synnematoformans (EF121313)
Streptomyces aculeolatus (AB184624)
Streptomyces artemisiae (EU200685)
Streptomyces armeniacus (AB018092)
Streptomyces pharmamarensis (FR693804)
Streptomyces phytohabitans (JQ345722)
Streptomyces cacaoi subsp. cacaoi (AB184115)
Streptomyces aminophilus (AB184183)
Streptomyces halophytocola (JQ819259)
Streptomyces sulphureus (TYGS 1257)
Streptomyces oryzae (AB894335)
Streptomyces nanhaiensis (GQ871748)
Streptomyces fenghuangensis (GU356598)
Streptomyces verrucosisporus (LC069041)

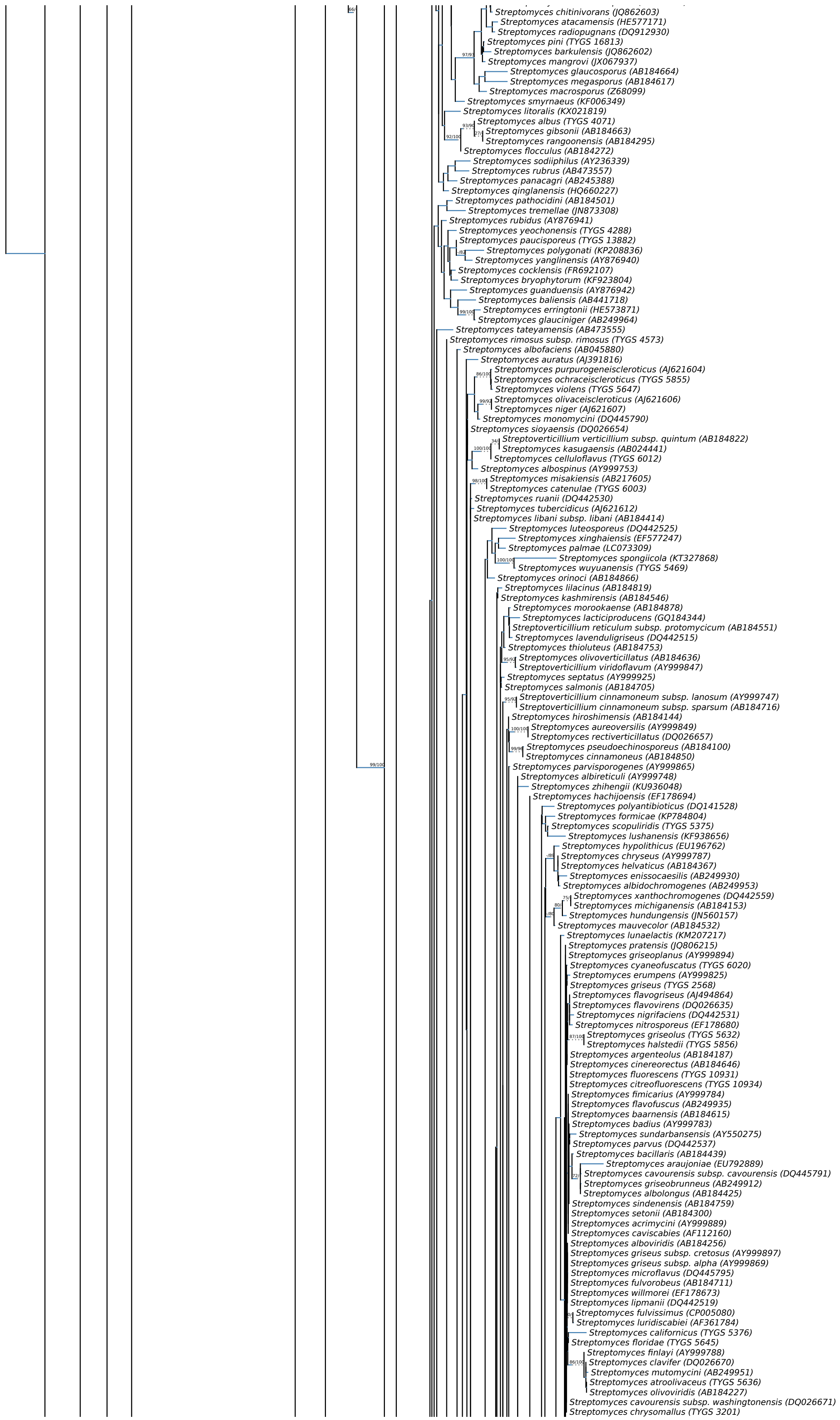
Streptomyces chitinivorans (JQ862603)
Streptomyces atacamensis (HE577171)
Streptomyces radiopugnans (DQ912930)
Streptomyces pini (TYGS 16813)
Streptomyces barkulensis (JQ862602)
Streptomyces mangrovi (JX067937)
Streptomyces glaucosporus (AB184664)
Streptomyces megasporus (AB184617)
Streptomyces macrosporus (Z68099)
Streptomyces smyrnaeus (KF006349)
Streptomyces litoralis (KX021819)
Streptomyces albus (TYGS 4071)
Streptomyces gibsonii (AB184663)
Streptomyces rangoonensis (AB184295)
Streptomyces flocculus (AB184272)
Streptomyces sodiiphilus (AY236339)
Streptomyces rubrus (AB473557)
Streptomyces panacagri (AB245388)
Streptomyces qinglanensis (HQ660227)
Streptomyces pathocidini (AB184501)
Streptomyces tremellae (JN873308)
Streptomyces rubidus (AY876941)
Streptomyces yeochonensis (TYGS 4288)
Streptomyces paucisporeus (TYGS 13882)
Streptomyces polygonati (KP208836)
Streptomyces yanglinensis (AY876940)
Streptomyces cocklensis (FR692107)
Streptomyces bryophytorum (KF923804)
Streptomyces guanduensis (AY876942)
Streptomyces baliensis (AB441718)
Streptomyces erringtonii (HE573871)
Streptomyces glauciniger (AB249964)
Streptomyces tateyamensis (AB473555)
Streptomyces rimosus subsp. rimosus (TYGS 4573)
Streptomyces albofaciens (AB045880)
Streptomyces auratus (AJ391816)
Streptomyces purpurogeneiscleroticus (AJ621604)
Streptomyces ochraceiscleroticus (TYGS 5855)
Streptomyces violens (TYGS 5647)
Streptomyces olivaceiscleroticus (AJ621606)
Streptomyces niger (AJ621607)
Streptomyces monomycini (DQ445790)
Streptomyces sioyaensis (DQ026654)
Streptoverticillium verticillium subsp. quintum (AB184822)
Streptomyces kasugaensis (AB024441)
Streptomyces celluloflavus (TYGS 6012)
Streptomyces albospinus (AY999753)
Streptomyces misakiensis (AB217605)
Streptomyces catenulae (TYGS 6003)
Streptomyces ruanii (DQ442530)
Streptomyces tubercidicus (AJ621612)
Streptomyces libani subsp. libani (AB184414)
Streptomyces luteosporeus (DQ442525)
Streptomyces xinghaiensis (EF577247)
Streptomyces palmae (LC073309)
Streptomyces spongiicola (KT327868)
Streptomyces wuyuanensis (TYGS 5469)
Streptomyces orinoci (AB184866)
Streptomyces lilacinus (AB184819)
Streptomyces kashmirensis (AB184546)
Streptomyces morookaense (AB184878)
Streptomyces lacticiproducens (GQ184344)
Streptoverticillium reticulum subsp. protomycicum (AB184551)
Streptomyces lavenduligriseus (DQ442515)
Streptomyces thioluteus (AB184753)
Streptomyces olivoverticillatus (AB184636)
Streptoverticillium viridoflavum (AY999847)
Streptomyces septatus (AY999925)
Streptomyces salmonis (AB184705)
Streptoverticillium cinnamoneum subsp. lanosum (AY999747)
Streptoverticillium cinnamoneum subsp. sparsum (AB184716)
Streptomyces hiroshimensis (AB184144)
Streptomyces aureoversilis (AY999849)
Streptomyces rectiverticillatus (DQ026657)
Streptomyces pseudoechinosporeus (AB184100)
Streptomyces cinnamoneus (AB184850)
Streptomyces parvisporogenes (AY999865)
Streptomyces albireticuli (AY999748)
Streptomyces zhihengii (KU936048)
Streptomyces hachijoensis (EF178694)
Streptomyces polyantibioticus (DQ141528)
Streptomyces formicae (KP784804)
Streptomyces scopuliridis (TYGS 5375)
Streptomyces lushanensis (KF938656)
Streptomyces hypolithicus (EU196762)
Streptomyces chryseus (AY999787)
Streptomyces helvaticus (AB184367)
Streptomyces enissocaesilis (AB249930)
Streptomyces albidochromogenes (AB249953)
Streptomyces xanthochromogenes (DQ442559)
Streptomyces michiganensis (AB184153)
Streptomyces hundungensis (JN560157)
Streptomyces mauvecolor (AB184532)
Streptomyces lunaelactis (KM207217)
Streptomyces pratensis (JQ806215)
Streptomyces griseoplanus (AY999894)
Streptomyces cyaneofuscatus (TYGS 6020)
Streptomyces erumpens (AY999825)
Streptomyces griseus (TYGS 2568)
Streptomyces flavogriseus (AJ494864)
Streptomyces flavovirens (DQ026635)
Streptomyces nigrifaciens (DQ442531)
Streptomyces nitrosporeus (EF178680)
Streptomyces griseolus (TYGS 5632)
Streptomyces halstedii (TYGS 5856)
Streptomyces argenteolus (AB184187)
Streptomyces cinereorectus (AB184646)
Streptomyces fluorescens (TYGS 10931)
Streptomyces citreofluorescens (TYGS 10934)
Streptomyces fimicarius (AY999784)
Streptomyces flavofuscus (AB249935)
Streptomyces baarnensis (AB184615)
Streptomyces badius (AY999783)
Streptomyces sundarbansensis (AY550275)
Streptomyces parvus (DQ442537)
Streptomyces bacillaris (AB184439)
Streptomyces araujoniae (EU792889)
Streptomyces cavourensis subsp. cavourensis (DQ445791)
Streptomyces griseobrunneus (AB249912)
Streptomyces albolongus (AB184425)
Streptomyces sindenensis (AB184759)
Streptomyces setonii (AB184300)
Streptomyces acrimycini (AY999889)
Streptomyces caviscabies (AF112160)
Streptomyces alboviridis (AB184256)
Streptomyces griseus subsp. cretosus (AY999897)
Streptomyces griseus subsp. alpha (AY999869)
Streptomyces microflavus (DQ445795)
Streptomyces fulvorobeus (AB184711)
Streptomyces willmorei (EF178673)
Streptomyces lipmanii (DQ442519)
Streptomyces fulvissimus (CP005080)
Streptomyces luridiscabiei (AF361784)
Streptomyces californicus (TYGS 5376)
Streptomyces floridae (TYGS 5645)
Streptomyces finlayi (AY999788)
Streptomyces clavifer (DQ026670)
Streptomyces mutomycini (AB249951)
Streptomyces atroolivaceus (TYGS 5636)
Streptomyces olivoviridis (AB184227)
Streptomyces cavourensis subsp. washingtonensis (DQ026671)
Streptomyces chrysomallus (TYGS 3201)

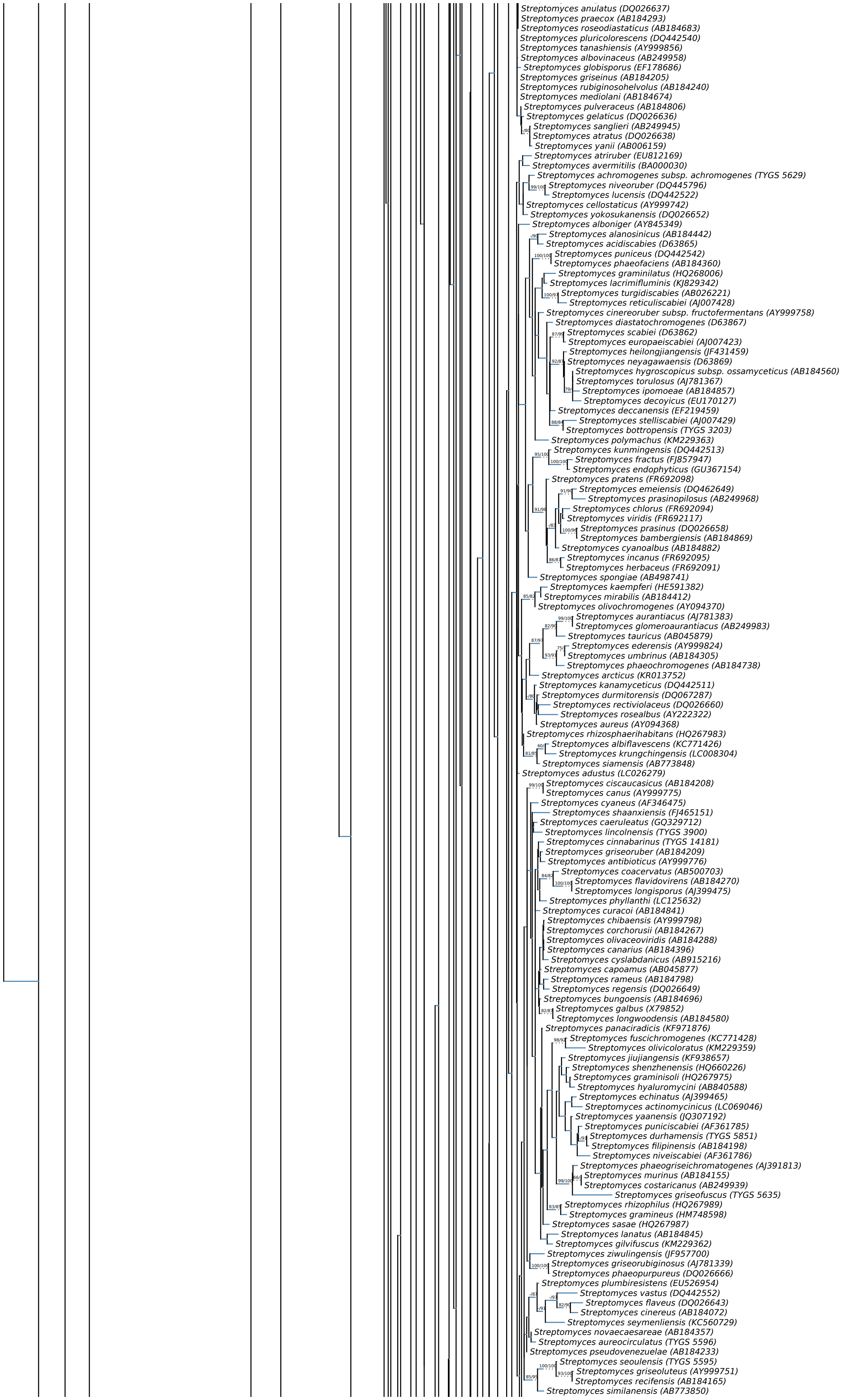
Streptomyces anulatus (DQ026637)
Streptomyces praecox (AB184293)
Streptomyces roseodiastaticus (AB184683)
Streptomyces pluricolorescens (DQ442540)
Streptomyces tanashiensis (AY999856)
Streptomyces albovinaceus (AB249958)
Streptomyces globisporus (EF178686)
Streptomyces griseinus (AB184205)
Streptomyces rubiginosohelvolus (AB184240)
Streptomyces mediolani (AB184674)
Streptomyces pulveraceus (AB184806)
Streptomyces gelaticus (DQ026636)
Streptomyces sanglieri (AB249945)
Streptomyces atratus (DQ026638)
Streptomyces yanii (AB006159)
Streptomyces atriruber (EU812169)
Streptomyces avermitilis (BA000030)
Streptomyces achromogenes subsp. achromogenes (TYGS 5629)
Streptomyces niveoruber (DQ445796)
Streptomyces lucensis (DQ442522)
Streptomyces cellostaticus (AY999742)
Streptomyces yokosukanensis (DQ026652)
Streptomyces alboniger (AY845349)
Streptomyces alanosinicus (AB184442)
Streptomyces acidiscabies (D63865)
Streptomyces puniceus (DQ442542)
Streptomyces phaeofaciens (AB184360)
Streptomyces graminilatus (HQ268006)
Streptomyces lacrimifluminis (KJ829342)
Streptomyces turgidiscabies (AB026221)
Streptomyces reticuliscabiei (AJ007428)
Streptomyces cinereoruber subsp. fructofermentans (AY999758)
Streptomyces diastatochromogenes (D63867)
Streptomyces scabiei (D63862)
Streptomyces europaeiscabiei (AJ007423)
Streptomyces heilongjiangensis (JF431459)
Streptomyces neyagawaensis (D63869)
Streptomyces hygroscopicus subsp. ossamyceticus (AB184560)
Streptomyces torulosus (AJ781367)
Streptomyces ipomoeae (AB184857)
Streptomyces decoyicus (EU170127)
Streptomyces deccanensis (EF219459)
Streptomyces stelliscabiei (AJ007429)
Streptomyces bottropensis (TYGS 3203)
Streptomyces polymachus (KM229363)
Streptomyces kunmingensis (DQ442513)
Streptomyces fractus (FJ857947)
Streptomyces endophyticus (GU367154)
Streptomyces pratens (FR692098)
Streptomyces emeiensis (DQ462649)
Streptomyces prasinopilosus (AB249968)
Streptomyces chlorus (FR692094)
Streptomyces viridis (FR692117)
Streptomyces prasinus (DQ026658)
Streptomyces bambergiensis (AB184869)
Streptomyces cyanoalbus (AB184882)
Streptomyces incanus (FR692095)
Streptomyces herbaceus (FR692091)
Streptomyces spongiae (AB498741)
Streptomyces kaempferi (HE591382)
Streptomyces mirabilis (AB184412)
Streptomyces olivochromogenes (AY094370)
Streptomyces aurantiacus (AJ781383)
Streptomyces glomeroaurantiacus (AB249983)
Streptomyces tauricus (AB045879)
Streptomyces ederensis (AY999824)
Streptomyces umbrinus (AB184305)
Streptomyces phaeochromogenes (AB184738)
Streptomyces arcticus (KR013752)
Streptomyces kanamyceticus (DQ442511)
Streptomyces durmitorensis (DQ067287)
Streptomyces rectiviolaceus (DQ026660)
Streptomyces rosealbus (AY222322)
Streptomyces aureus (AY094368)
Streptomyces rhizosphaerihabitans (HQ267983)
Streptomyces albiflavescens (KC771426)
Streptomyces krungchingensis (LC008304)
Streptomyces siamensis (AB773848)
Streptomyces adustus (LC026279)
Streptomyces ciscaucasicus (AB184208)
Streptomyces canus (AY999775)
Streptomyces cyaneus (AF346475)
Streptomyces shaanxiensis (FJ465151)
Streptomyces caeruleatus (GQ329712)
Streptomyces lincolnensis (TYGS 3900)
Streptomyces cinnabarinus (TYGS 14181)
Streptomyces griseoruber (AB184209)
Streptomyces antibioticus (AY999776)
Streptomyces coacervatus (AB500703)
Streptomyces flavidovirens (AB184270)
Streptomyces longisporus (AJ399475)
Streptomyces phyllanthi (LC125632)
Streptomyces curacoi (AB184841)
Streptomyces chibaensis (AY999798)
Streptomyces corchorusii (AB184267)
Streptomyces olivaceoviridis (AB184288)
Streptomyces canarius (AB184396)
Streptomyces cyslabdanicus (AB915216)
Streptomyces capoamus (AB045877)
Streptomyces rameus (AB184798)
Streptomyces regensis (DQ026649)
Streptomyces bungoensis (AB184696)
Streptomyces galbus (X79852)
Streptomyces longwoodensis (AB184580)
Streptomyces panaciradicis (KF971876)
Streptomyces fuscichromogenes (KC771428)
Streptomyces olivicoloratus (KM229359)
Streptomyces jiujiangensis (KF938657)
Streptomyces shenzhenensis (HQ660226)
Streptomyces graminisoli (HQ267975)
Streptomyces hyaluromycini (AB840588)
Streptomyces echinatus (AJ399465)
Streptomyces actinomycinicus (LC069046)
Streptomyces yaanensis (JQ307192)
Streptomyces puniciscabiei (AF361785)
Streptomyces durhamensis (TYGS 5851)
Streptomyces filipinensis (AB184198)
Streptomyces niveiscabiei (AF361786)
Streptomyces phaeogriseichromatogenes (AJ391813)
Streptomyces murinus (AB184155)
Streptomyces costaricanus (AB249939)
Streptomyces griseofuscus (TYGS 5635)
Streptomyces rhizophilus (HQ267989)
Streptomyces gramineus (HM748598)
Streptomyces sasae (HQ267987)
Streptomyces lanatus (AB184845)
Streptomyces gilvifuscus (KM229362)
Streptomyces ziwulingensis (JF957700)
Streptomyces griseorubiginosus (AJ781339)
Streptomyces phaeopurpureus (DQ026666)
Streptomyces plumbiresistens (EU526954)
Streptomyces vastus (DQ442552)
Streptomyces flaveus (DQ026643)
Streptomyces cinereus (AB184072)
Streptomyces seymenliensis (KC560729)
Streptomyces novaecaesareae (AB184357)
Streptomyces aureocirculatus (TYGS 5596)
Streptomyces pseudovenezuelae (AB184233)
Streptomyces seoulensis (TYGS 5595)
Streptomyces griseoluteus (AY999751)
Streptomyces recifensis (AB184165)
Streptomyces similanensis (AB773850)

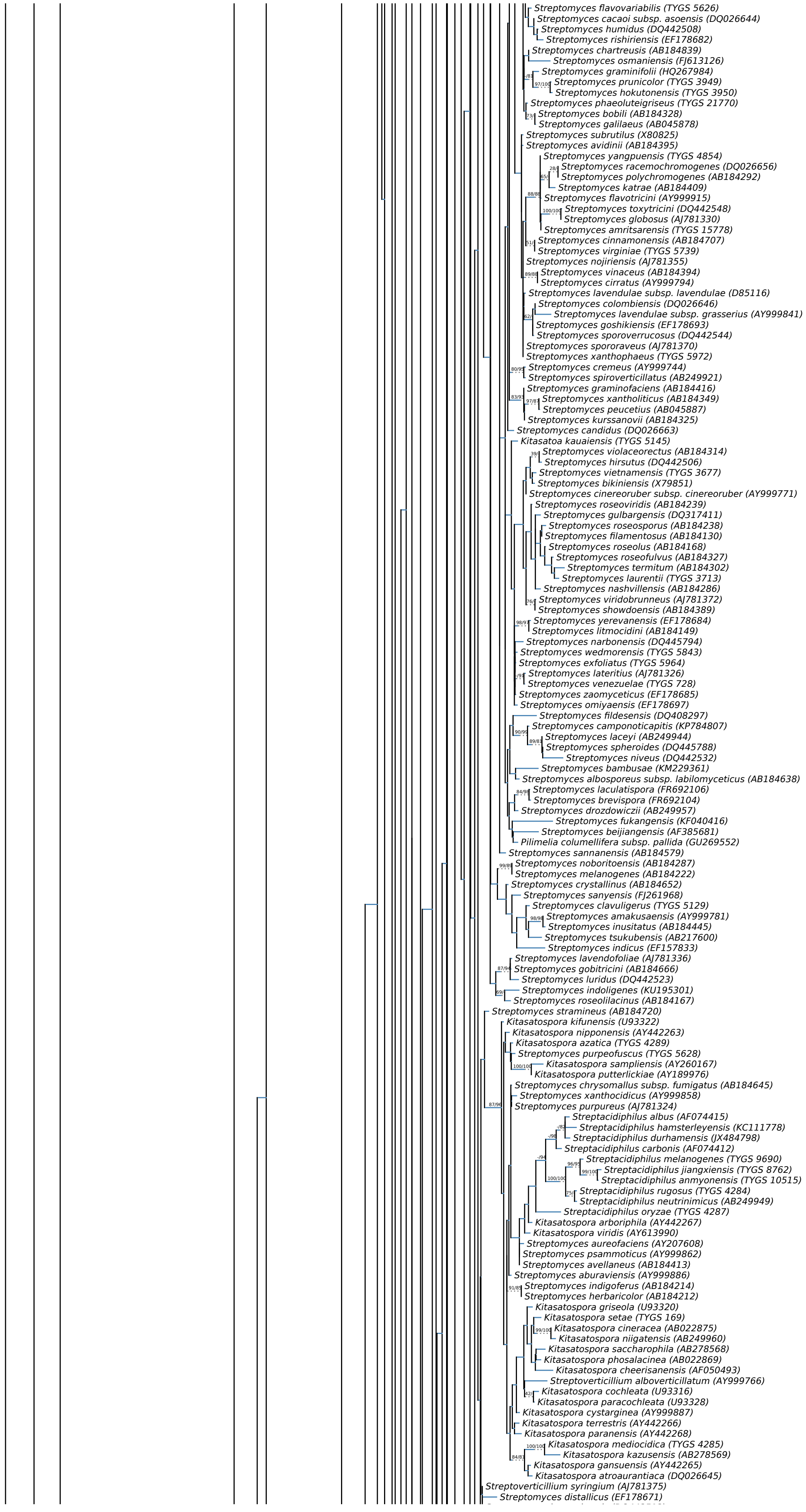
Streptomyces flavovariabilis (TYGS 5626)
Streptomyces cacaoi subsp. asoensis (DQ026644)
Streptomyces humidus (DQ442508)
Streptomyces rishiriensis (EF178682)
Streptomyces chartreusis (AB184839)
Streptomyces osmaniensis (FJ613126)
Streptomyces graminifolii (HQ267984)
Streptomyces prunicolor (TYGS 3949)
Streptomyces hokutonensis (TYGS 3950)
Streptomyces phaeoluteigriseus (TYGS 21770)
Streptomyces bobili (AB184328)
Streptomyces galilaeus (AB045878)
Streptomyces subrutilus (X80825)
Streptomyces avidinii (AB184395)
Streptomyces yangpuensis (TYGS 4854)
Streptomyces racemochromogenes (DQ026656)
Streptomyces polychromogenes (AB184292)
Streptomyces katrae (AB184409)
Streptomyces flavotricini (AY999915)
Streptomyces toxytricini (DQ442548)
Streptomyces globosus (AJ781330)
Streptomyces amritsarensis (TYGS 15778)
Streptomyces cinnamonensis (AB184707)
Streptomyces virginiae (TYGS 5739)
Streptomyces nojiriensis (AJ781355)
Streptomyces vinaceus (AB184394)
Streptomyces cirratus (AY999794)
Streptomyces lavendulae subsp. lavendulae (D85116)
Streptomyces colombiensis (DQ026646)
Streptomyces lavendulae subsp. grasserius (AY999841)
Streptomyces goshikiensis (EF178693)
Streptomyces sporoverrucosus (DQ442544)
Streptomyces spororaveus (AJ781370)
Streptomyces xanthophaeus (TYGS 5972)
Streptomyces cremeus (AY999744)
Streptomyces spiroverticillatus (AB249921)
Streptomyces graminofaciens (AB184416)
Streptomyces xantholiticus (AB184349)
Streptomyces peucetius (AB045887)
Streptomyces kurssanovii (AB184325)
Streptomyces candidus (DQ026663)
Kitasatoa kauaiensis (TYGS 5145)
Streptomyces violaceorectus (AB184314)
Streptomyces hirsutus (DQ442506)
Streptomyces vietnamensis (TYGS 3677)
Streptomyces bikiniensis (X79851)
Streptomyces cinereoruber subsp. cinereoruber (AY999771)
Streptomyces roseoviridis (AB184239)
Streptomyces gulbargensis (DQ317411)
Streptomyces roseosporus (AB184238)
Streptomyces filamentosus (AB184130)
Streptomyces roseolus (AB184168)
Streptomyces roseofulvus (AB184327)
Streptomyces termitum (AB184302)
Streptomyces laurentii (TYGS 3713)
Streptomyces nashvillensis (AB184286)
Streptomyces viridobrunneus (AJ781372)
Streptomyces showdoensis (AB184389)
Streptomyces yerevanensis (EF178684)
Streptomyces litmocidini (AB184149)
Streptomyces narbonensis (DQ445794)
Streptomyces wedmorensis (TYGS 5843)
Streptomyces exfoliatus (TYGS 5964)
Streptomyces lateritius (AJ781326)
Streptomyces venezuelae (TYGS 728)
Streptomyces zaomyceticus (EF178685)
Streptomyces omiyaensis (EF178697)
Streptomyces fildesensis (DQ408297)
Streptomyces camponoticapitis (KP784807)
Streptomyces laceyi (AB249944)
Streptomyces spheroides (DQ445788)
Streptomyces niveus (DQ442532)
Streptomyces bambusae (KM229361)
Streptomyces albosporeus subsp. labilomyceticus (AB184638)
Streptomyces laculatispora (FR692106)
Streptomyces brevispora (FR692104)
Streptomyces drozdowiczii (AB249957)
Streptomyces fukangensis (KF040416)
Streptomyces beijiangensis (AF385681)
Pilimelia columellifera subsp. pallida (GU269552)
Streptomyces sannanensis (AB184579)
Streptomyces noboritoensis (AB184287)
Streptomyces melanogenes (AB184222)
Streptomyces crystallinus (AB184652)
Streptomyces sanyensis (FJ261968)
Streptomyces clavuligerus (TYGS 5129)
Streptomyces amakusaensis (AY999781)
Streptomyces inusitatus (AB184445)
Streptomyces tsukubensis (AB217600)
Streptomyces indicus (EF157833)
Streptomyces lavendofoliae (AJ781336)
Streptomyces gobitricini (AB184666)
Streptomyces luridus (DQ442523)
Streptomyces indoligenes (KU195301)
Streptomyces roseolilacinus (AB184167)
Streptomyces stramineus (AB184720)
Kitasatospora kifunensis (U93322)
Kitasatospora nipponensis (AY442263)
Kitasatospora azatica (TYGS 4289)
Streptomyces purpeofuscus (TYGS 5628)
Kitasatospora sampliensis (AY260167)
Kitasatospora putterlickiae (AY189976)
Streptomyces chrysomallus subsp. fumigatus (AB184645)
Streptomyces xanthocidicus (AY999858)
Streptomyces purpureus (AJ781324)
Streptacidiphilus albus (AF074415)
Streptacidiphilus hamsterleyensis (KC111778)
Streptacidiphilus durhamensis (JX484798)
Streptacidiphilus carbonis (AF074412)
Streptacidiphilus melanogenes (TYGS 9690)
Streptacidiphilus jiangxiensis (TYGS 8762)
Streptacidiphilus anmyonensis (TYGS 10515)
Streptacidiphilus rugosus (TYGS 4284)
Streptacidiphilus neutrinimicus (AB249949)
Streptacidiphilus oryzae (TYGS 4287)
Kitasatospora arboriphila (AY442267)
Kitasatospora viridis (AY613990)
Streptomyces aureofaciens (AY207608)
Streptomyces psammoticus (AY999862)
Streptomyces avellaneus (AB184413)
Streptomyces aburaviensis (AY999886)
Streptomyces indigoferus (AB184214)
Streptomyces herbaricolor (AB184212)
Kitasatospora griseola (U93320)
Kitasatospora setae (TYGS 169)
Kitasatospora cineracea (AB022875)
Kitasatospora niigatensis (AB249960)
Kitasatospora saccharophila (AB278568)
Kitasatospora phosalacinea (AB022869)
Kitasatospora cheerisanensis (AF050493)
Streptoverticillium alboverticillatum (AY999766)
Kitasatospora cochleata (U93316)
Kitasatospora paracochleata (U93328)
Kitasatospora cystarginea (AY999887)
Kitasatospora terrestris (AY442266)
Kitasatospora paranensis (AY442268)
Kitasatospora mediocidica (TYGS 4285)
Kitasatospora kazusensis (AB278569)
Kitasatospora gansuensis (AY442265)
Kitasatospora atroaurantiaca (DQ026645)
Streptoverticillium syringium (AJ781375)
Streptomyces distallicus (EF178671)

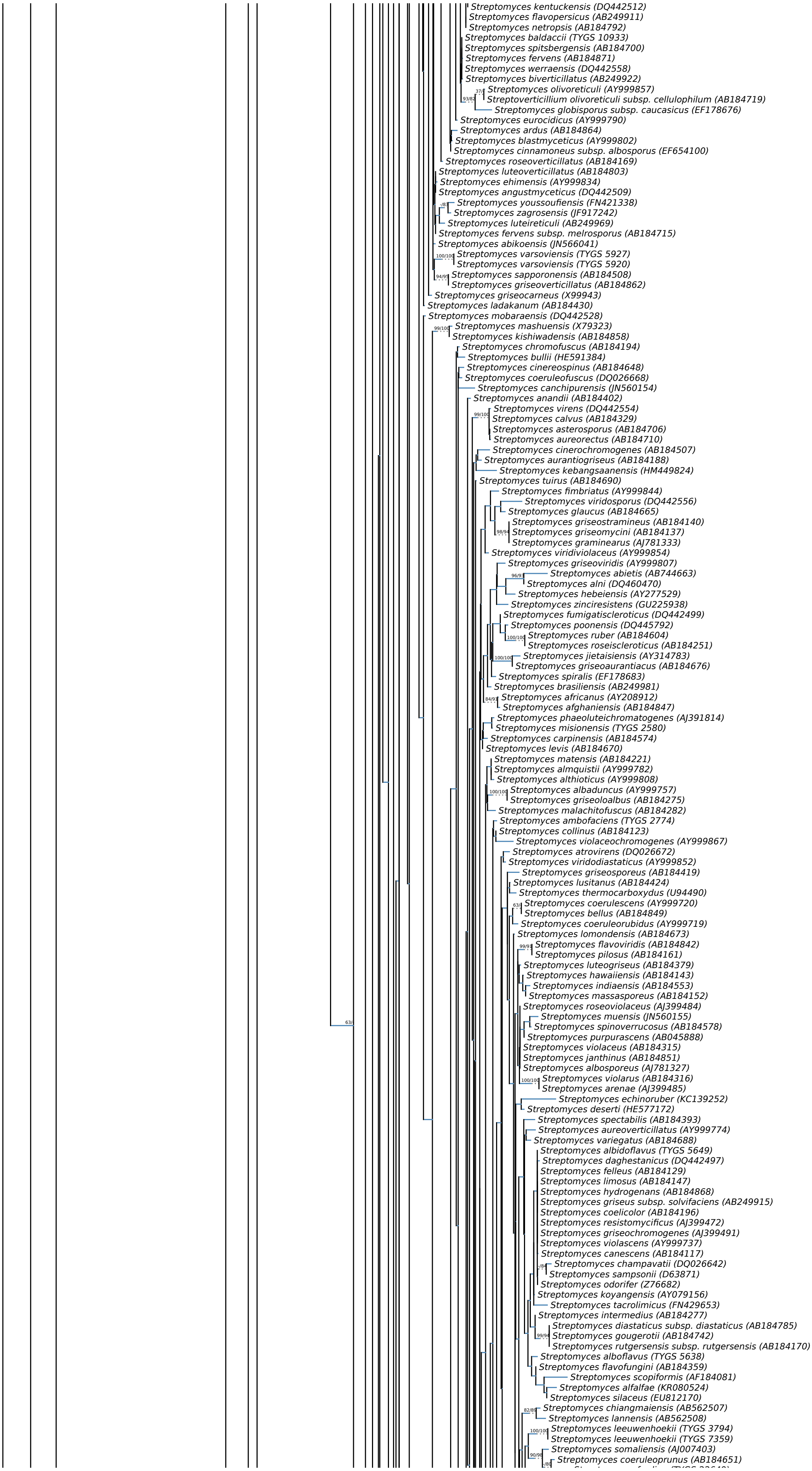
Streptomyces kentuckensis (DQ442512)
Streptomyces flavopersicus (AB249911)
Streptomyces netropsis (AB184792)
Streptomyces baldaccii (TYGS 10933)
Streptomyces spitsbergensis (AB184700)
Streptomyces fervens (AB184871)
Streptomyces werraensis (DQ442558)
Streptomyces biverticillatus (AB249922)
Streptomyces olivoreticuli (AY999857)
Streptoverticillium olivoreticuli subsp. cellulophilum (AB184719)
Streptomyces globisporus subsp. caucasicus (EF178676)
Streptomyces eurocidicus (AY999790)
Streptomyces ardus (AB184864)
Streptomyces blastmyceticus (AY999802)
Streptomyces cinnamoneus subsp. albosporus (EF654100)
Streptomyces roseoverticillatus (AB184169)
Streptomyces luteoverticillatus (AB184803)
Streptomyces ehimensis (AY999834)
Streptomyces angustmyceticus (DQ442509)
Streptomyces youssoufiensis (FN421338)
Streptomyces zagrosensis (JF917242)
Streptomyces luteireticuli (AB249969)
Streptomyces fervens subsp. melrosporus (AB184715)
Streptomyces abikoensis (JN566041)
Streptomyces varsoviensis (TYGS 5927)
Streptomyces varsoviensis (TYGS 5920)
Streptomyces sapporonensis (AB184508)
Streptomyces griseoverticillatus (AB184862)
Streptomyces griseocarneus (X99943)
Streptomyces ladakanum (AB184430)
Streptomyces mobaraensis (DQ442528)
Streptomyces mashuensis (X79323)
Streptomyces kishiwadensis (AB184858)
Streptomyces chromofuscus (AB184194)
Streptomyces bullii (HE591384)
Streptomyces cinereospinus (AB184648)
Streptomyces coeruleofuscus (DQ026668)
Streptomyces canchipurensis (JN560154)
Streptomyces anandii (AB184402)
Streptomyces virens (DQ442554)
Streptomyces calvus (AB184329)
Streptomyces asterosporus (AB184706)
Streptomyces aureorectus (AB184710)
Streptomyces cinerochromogenes (AB184507)
Streptomyces aurantiogriseus (AB184188)
Streptomyces kebangsaanensis (HM449824)
Streptomyces tuirus (AB184690)
Streptomyces fimbriatus (AY999844)
Streptomyces viridosporus (DQ442556)
Streptomyces glaucus (AB184665)
Streptomyces griseostramineus (AB184140)
Streptomyces griseomycini (AB184137)
Streptomyces graminearus (AJ781333)
Streptomyces viridiviolaceus (AY999854)
Streptomyces griseoviridis (AY999807)
Streptomyces abietis (AB744663)
Streptomyces alni (DQ460470)
Streptomyces hebeiensis (AY277529)
Streptomyces zinciresistens (GU225938)
Streptomyces fumigatiscleroticus (DQ442499)
Streptomyces poonensis (DQ445792)
Streptomyces ruber (AB184604)
Streptomyces roseiscleroticus (AB184251)
Streptomyces jietaisiensis (AY314783)
Streptomyces griseoaurantiacus (AB184676)
Streptomyces spiralis (EF178683)
Streptomyces brasiliensis (AB249981)
Streptomyces africanus (AY208912)
Streptomyces afghaniensis (AB184847)
Streptomyces phaeoluteichromatogenes (AJ391814)
Streptomyces misionensis (TYGS 2580)
Streptomyces carpinensis (AB184574)
Streptomyces levis (AB184670)
Streptomyces matensis (AB184221)
Streptomyces almquistii (AY999782)
Streptomyces althioticus (AY999808)
Streptomyces albaduncus (AY999757)
Streptomyces griseoloalbus (AB184275)
Streptomyces malachitofuscus (AB184282)
Streptomyces ambofaciens (TYGS 2774)
Streptomyces collinus (AB184123)
Streptomyces violaceochromogenes (AY999867)
Streptomyces atrovirens (DQ026672)
Streptomyces viridodiastaticus (AY999852)
Streptomyces griseosporeus (AB184419)
Streptomyces lusitanus (AB184424)
Streptomyces thermocarboxydus (U94490)
Streptomyces coerulescens (AY999720)
Streptomyces bellus (AB184849)
Streptomyces coeruleorubidus (AY999719)
Streptomyces lomondensis (AB184673)
Streptomyces flavoviridis (AB184842)
Streptomyces pilosus (AB184161)
Streptomyces luteogriseus (AB184379)
Streptomyces hawaiiensis (AB184143)
Streptomyces indiaensis (AB184553)
Streptomyces massasporeus (AB184152)
Streptomyces roseoviolaceus (AJ399484)
Streptomyces muensis (JN560155)
Streptomyces spinoverrucosus (AB184578)
Streptomyces purpurascens (AB045888)
Streptomyces violaceus (AB184315)
Streptomyces janthinus (AB184851)
Streptomyces albosporeus (AJ781327)
Streptomyces violarus (AB184316)
Streptomyces arenae (AJ399485)
Streptomyces echinoruber (KC139252)
Streptomyces deserti (HE577172)
Streptomyces spectabilis (AB184393)
Streptomyces aureoverticillatus (AY999774)
Streptomyces variegatus (AB184688)
Streptomyces albidoflavus (TYGS 5649)
Streptomyces daghestanicus (DQ442497)
Streptomyces felleus (AB184129)
Streptomyces limosus (AB184147)
Streptomyces hydrogenans (AB184868)
Streptomyces griseus subsp. solvifaciens (AB249915)
Streptomyces coelicolor (AB184196)
Streptomyces resistomycificus (AJ399472)
Streptomyces griseochromogenes (AJ399491)
Streptomyces violascens (AY999737)
Streptomyces canescens (AB184117)
Streptomyces champavatii (DQ026642)
Streptomyces sampsonii (D63871)
Streptomyces odorifer (Z76682)
Streptomyces koyangensis (AY079156)
Streptomyces tacrolimicus (FN429653)
Streptomyces intermedius (AB184277)
Streptomyces diastaticus subsp. diastaticus (AB184785)
Streptomyces gougerotii (AB184742)
Streptomyces rutgersensis subsp. rutgersensis (AB184170)
Streptomyces alboflavus (TYGS 5638)
Streptomyces flavofungini (AB184359)
Streptomyces scopiformis (AF184081)
Streptomyces alfalfae (KR080524)
Streptomyces silaceus (EU812170)
Streptomyces chiangmaiensis (AB562507)
Streptomyces lannensis (AB562508)
Streptomyces leeuwenhoekii (TYGS 3794)
Streptomyces leeuwenhoekii (TYGS 7359)
Streptomyces somaliensis (AJ007403)
Streptomyces coeruleoprunus (AB184651)

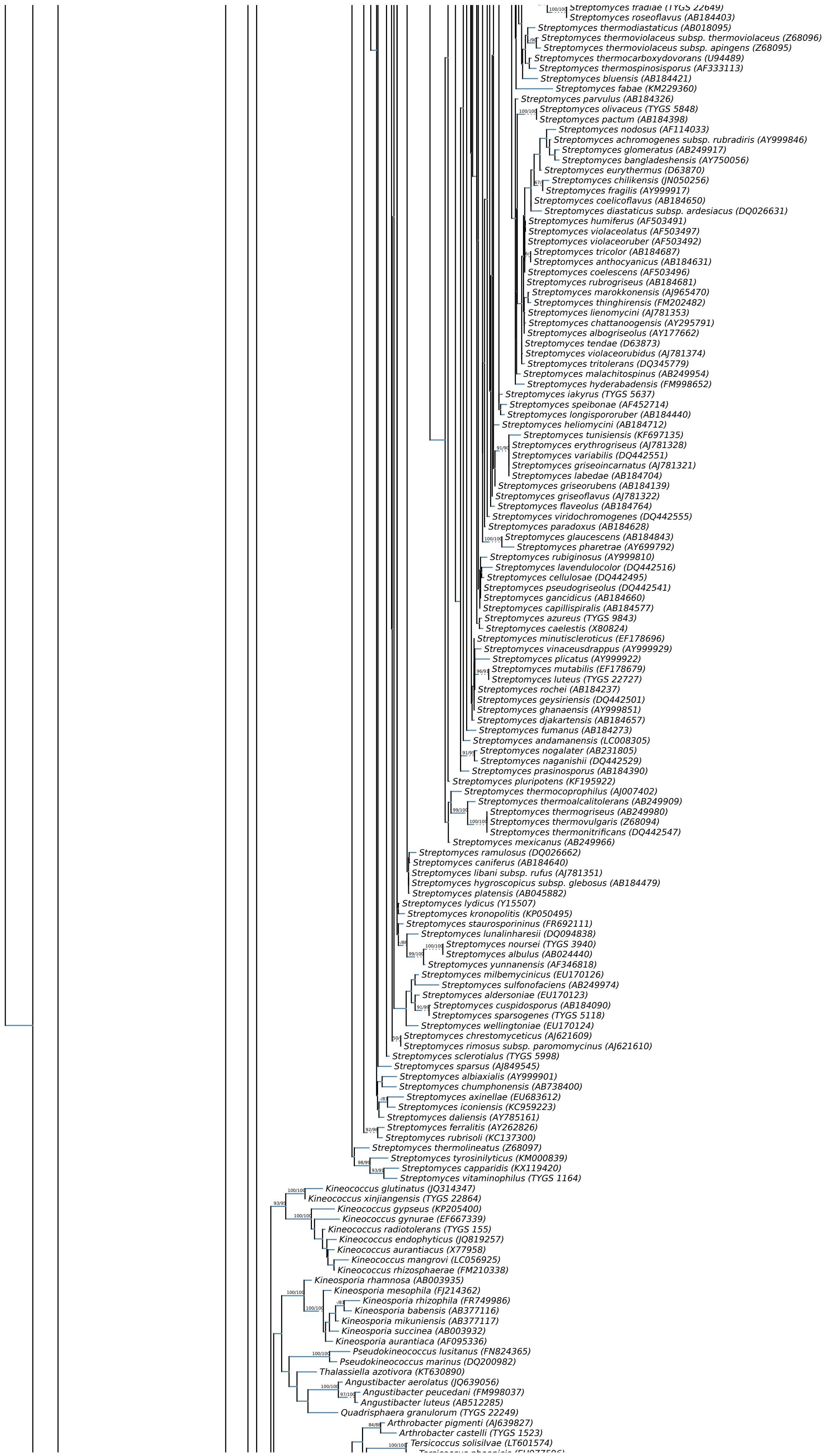

Streptomyces fradiae (TYGS 22649)
Streptomyces roseoflavus (AB184403)
Streptomyces thermodiastaticus (AB018095)
Streptomyces thermoviolaceus subsp. thermoviolaceus (Z68096)
Streptomyces thermoviolaceus subsp. apingens (Z68095)
Streptomyces thermocarboxydovorans (U94489)
Streptomyces thermospinosisporus (AF333113)
Streptomyces bluensis (AB184421)
Streptomyces fabae (KM229360)
Streptomyces parvulus (AB184326)
Streptomyces olivaceus (TYGS 5848)
Streptomyces pactum (AB184398)
Streptomyces nodosus (AF114033)
Streptomyces achromogenes subsp. rubradiris (AY999846)
Streptomyces glomeratus (AB249917)
Streptomyces bangladeshensis (AY750056)
Streptomyces eurythermus (D63870)
Streptomyces chilikensis (JN050256)
Streptomyces fragilis (AY999917)
Streptomyces coelicoflavus (AB184650)
Streptomyces diastaticus subsp. ardesiacus (DQ026631)
Streptomyces humiferus (AF503491)
Streptomyces violaceolatus (AF503497)
Streptomyces violaceoruber (AF503492)
Streptomyces tricolor (AB184687)
Streptomyces anthocyanicus (AB184631)
Streptomyces coelescens (AF503496)
Streptomyces rubrogriseus (AB184681)
Streptomyces marokkonensis (AJ965470)
Streptomyces thinghirensis (FM202482)
Streptomyces lienomycini (AJ781353)
Streptomyces chattanoogensis (AY295791)
Streptomyces albogriseolus (AY177662)
Streptomyces tendae (D63873)
Streptomyces violaceorubidus (AJ781374)
Streptomyces tritolerans (DQ345779)
Streptomyces malachitospinus (AB249954)
Streptomyces hyderabadensis (FM998652)
Streptomyces iakyrus (TYGS 5637)
Streptomyces speibonae (AF452714)
Streptomyces longispororuber (AB184440)
Streptomyces heliomycini (AB184712)
Streptomyces tunisiensis (KF697135)
Streptomyces erythrogriseus (AJ781328)
Streptomyces variabilis (DQ442551)
Streptomyces griseoincarnatus (AJ781321)
Streptomyces labedae (AB184704)
Streptomyces griseorubens (AB184139)
Streptomyces griseoflavus (AJ781322)
Streptomyces flaveolus (AB184764)
Streptomyces viridochromogenes (DQ442555)
Streptomyces paradoxus (AB184628)
Streptomyces glaucescens (AB184843)
Streptomyces pharetrae (AY699792)
Streptomyces rubiginosus (AY999810)
Streptomyces lavendulocolor (DQ442516)
Streptomyces cellulosae (DQ442495)
Streptomyces pseudogriseolus (DQ442541)
Streptomyces gancidicus (AB184660)
Streptomyces capillispiralis (AB184577)
Streptomyces azureus (TYGS 9843)
Streptomyces caelestis (X80824)
Streptomyces minutiscleroticus (EF178696)
Streptomyces vinaceusdrappus (AY999929)
Streptomyces plicatus (AY999922)
Streptomyces mutabilis (EF178679)
Streptomyces luteus (TYGS 22727)
Streptomyces rochei (AB184237)
Streptomyces geysiriensis (DQ442501)
Streptomyces ghanaensis (AY999851)
Streptomyces djakartensis (AB184657)
Streptomyces fumanus (AB184273)
Streptomyces andamanensis (LC008305)
Streptomyces nogalater (AB231805)
Streptomyces naganishii (DQ442529)
Streptomyces prasinosporus (AB184390)
Streptomyces pluripotens (KF195922)
Streptomyces thermocoprophilus (AJ007402)
Streptomyces thermoalcalitolerans (AB249909)
Streptomyces thermogriseus (AB249980)
Streptomyces thermovulgaris (Z68094)
Streptomyces thermonitrificans (DQ442547)
Streptomyces mexicanus (AB249966)
Streptomyces ramulosus (DQ026662)
Streptomyces caniferus (AB184640)
Streptomyces libani subsp. rufus (AJ781351)
Streptomyces hygroscopicus subsp. glebosus (AB184479)
Streptomyces platensis (AB045882)
Streptomyces lydicus (Y15507)
Streptomyces kronopolitis (KP050495)
Streptomyces staurosporininus (FR692111)
Streptomyces lunalinharesii (DQ094838)
Streptomyces noursei (TYGS 3940)
Streptomyces albulus (AB024440)
Streptomyces yunnanensis (AF346818)
Streptomyces milbemycinicus (EU170126)
Streptomyces sulfonofaciens (AB249974)
Streptomyces aldersoniae (EU170123)
Streptomyces cuspidosporus (AB184090)
Streptomyces sparsogenes (TYGS 5118)
Streptomyces wellingtoniae (EU170124)
Streptomyces chrestomyceticus (AJ621609)
Streptomyces rimosus subsp. paromomycinus (AJ621610)
Streptomyces sclerotialus (TYGS 5998)
Streptomyces sparsus (AJ849545)
Streptomyces albiaxialis (AY999901)
Streptomyces chumphonensis (AB738400)
Streptomyces axinellae (EU683612)
Streptomyces iconiensis (KC959223)
Streptomyces daliensis (AY785161)
Streptomyces ferralitis (AY262826)
Streptomyces rubrisoli (KC137300)
Streptomyces thermolineatus (Z68097)
Streptomyces tyrosinilyticus (KM000839)
Streptomyces capparidis (KX119420)
Streptomyces vitaminophilus (TYGS 1164)
Kineococcus glutinatus (JQ314347)
Kineococcus xinjiangensis (TYGS 22864)
Kineococcus gypseus (KP205400)
Kineococcus gynurae (EF667339)
Kineococcus radiotolerans (TYGS 155)
Kineococcus endophyticus (JQ819257)
Kineococcus aurantiacus (X77958)
Kineococcus mangrovi (LC056925)
Kineococcus rhizosphaerae (FM210338)
Kineosporia rhamnosa (AB003935)
Kineosporia mesophila (FJ214362)
Kineosporia rhizophila (FR749986)
Kineosporia babensis (AB377116)
Kineosporia mikuniensis (AB377117)
Kineosporia succinea (AB003932)
Kineosporia aurantiaca (AF095336)
Pseudokineococcus lusitanus (FN824365)
Pseudokineococcus marinus (DQ200982)
Thalassiella azotivora (KT630890)
Angustibacter aerolatus (JQ639056)
Angustibacter peucedani (FM998037)
Angustibacter luteus (AB512285)
Quadrisphaera granulorum (TYGS 22249)
Arthrobacter pigmenti (AJ639827)
Arthrobacter castelli (TYGS 1523)
Tersicoccus solisilvae (LT601574)

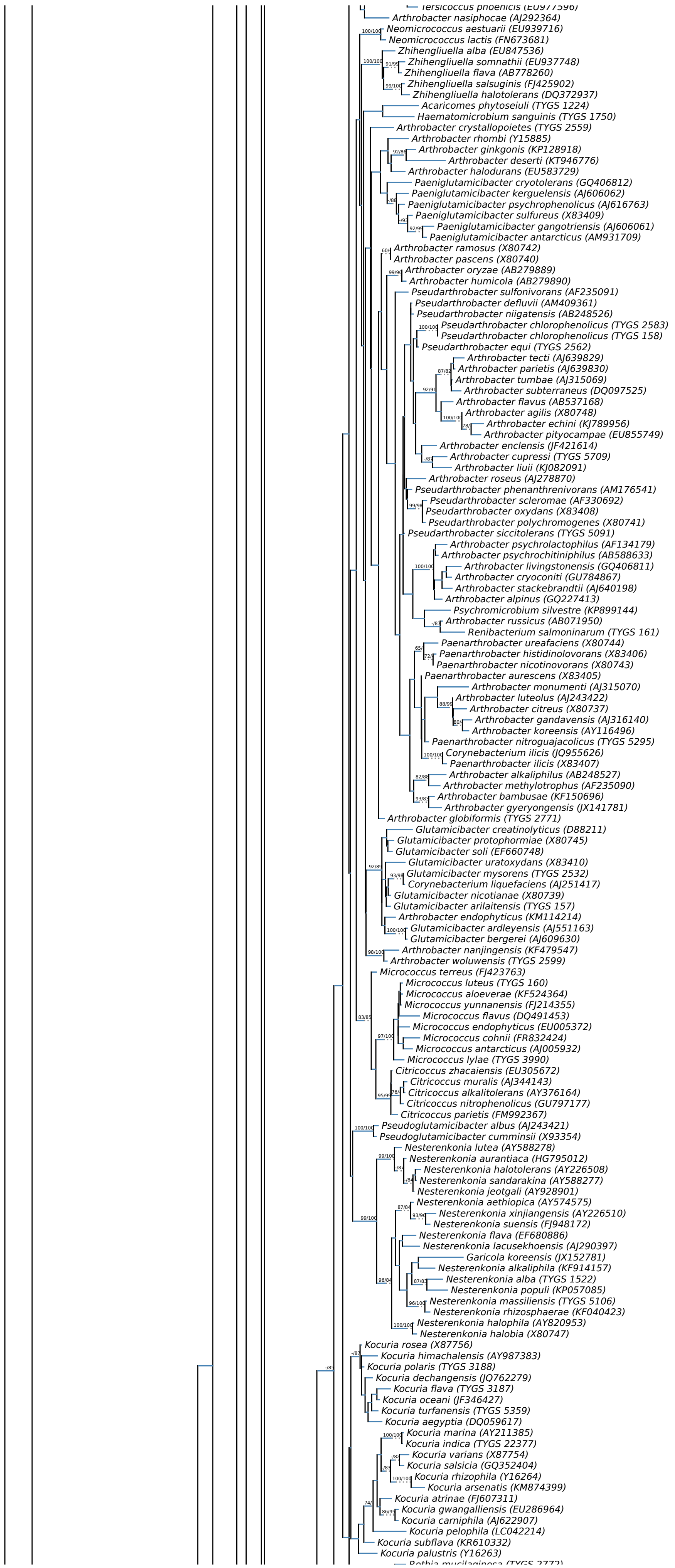
Arthrobacter nasiphocae (AJ292364)
Neomicrococcus aestuarii (EU939716)
Neomicrococcus lactis (FN673681)
Zhihengliuella alba (EU847536)
Zhihengliuella somnathii (EU937748)
Zhihengliuella flava (AB778260)
Zhihengliuella salsuginis (FJ425902)
Zhihengliuella halotolerans (DQ372937)
Acaricomes phytoseiuli (TYGS 1224)
Haematomicrobium sanguinis (TYGS 1750)
Arthrobacter crystallopoietes (TYGS 2559)
Arthrobacter rhombi (Y15885)
Arthrobacter ginkgonis (KP128918)
Arthrobacter deserti (KT946776)
Arthrobacter halodurans (EU583729)
Paeniglutamicibacter cryotolerans (GQ406812)
Paeniglutamicibacter kerguelensis (AJ606062)
Paeniglutamicibacter psychrophenolicus (AJ616763)
Paeniglutamicibacter sulfureus (X83409)
Paeniglutamicibacter gangotriensis (AJ606061)
Paeniglutamicibacter antarcticus (AM931709)
Arthrobacter ramosus (X80742)
Arthrobacter pascens (X80740)
Arthrobacter oryzae (AB279889)
Arthrobacter humicola (AB279890)
Pseudarthrobacter sulfonivorans (AF235091)
Pseudarthrobacter defluvii (AM409361)
Pseudarthrobacter niigatensis (AB248526)
Pseudarthrobacter chlorophenolicus (TYGS 2583)
Pseudarthrobacter chlorophenolicus (TYGS 158)
Pseudarthrobacter equi (TYGS 2562)
Arthrobacter tecti (AJ639829)
Arthrobacter parietis (AJ639830)
Arthrobacter tumbae (AJ315069)
Arthrobacter subterraneus (DQ097525)
Arthrobacter flavus (AB537168)
Arthrobacter agilis (X80748)
Arthrobacter echini (KJ789956)
Arthrobacter pityocampae (EU855749)
Arthrobacter enclensis (JF421614)
Arthrobacter cupressi (TYGS 5709)
Arthrobacter liuii (KJ082091)
Arthrobacter roseus (AJ278870)
Pseudarthrobacter phenanthrenivorans (AM176541)
Pseudarthrobacter scleromae (AF330692)
Pseudarthrobacter oxydans (X83408)
Pseudarthrobacter polychromogenes (X80741)
Pseudarthrobacter siccitolerans (TYGS 5091)
Arthrobacter psychrolactophilus (AF134179)
Arthrobacter psychrochitiniphilus (AB588633)
Arthrobacter livingstonensis (GQ406811)
Arthrobacter cryoconiti (GU784867)
Arthrobacter stackebrandtii (AJ640198)
Arthrobacter alpinus (GQ227413)
Psychromicrobium silvestre (KP899144)
Arthrobacter russicus (AB071950)
Renibacterium salmoninarum (TYGS 161)
Paenarthrobacter ureafaciens (X80744)
Paenarthrobacter histidinolovorans (X83406)
Paenarthrobacter nicotinovorans (X80743)
Paenarthrobacter aurescens (X83405)
Arthrobacter monumenti (AJ315070)
Arthrobacter luteolus (AJ243422)
Arthrobacter citreus (X80737)
Arthrobacter gandavensis (AJ316140)
Arthrobacter koreensis (AY116496)
Paenarthrobacter nitroguajacolicus (TYGS 5295)
Corynebacterium ilicis (JQ955626)
Paenarthrobacter ilicis (X83407)
Arthrobacter alkaliphilus (AB248527)
Arthrobacter methylotrophus (AF235090)
Arthrobacter bambusae (KF150696)
Arthrobacter gyeryongensis (JX141781)
Arthrobacter globiformis (TYGS 2771)
Glutamicibacter creatinolyticus (D88211)
Glutamicibacter protophormiae (X80745)
Glutamicibacter soli (EF660748)
Glutamicibacter uratoxydans (X83410)
Glutamicibacter mysorens (TYGS 2532)
Corynebacterium liquefaciens (AJ251417)
Glutamicibacter nicotianae (X80739)
Glutamicibacter arilaitensis (TYGS 157)
Arthrobacter endophyticus (KM114214)
Glutamicibacter ardleyensis (AJ551163)
Glutamicibacter bergerei (AJ609630)
Arthrobacter nanjingensis (KF479547)
Arthrobacter woluwensis (TYGS 2599)
Micrococcus terreus (FJ423763)
Micrococcus luteus (TYGS 160)
Micrococcus aloeverae (KF524364)
Micrococcus yunnanensis (FJ214355)
Micrococcus flavus (DQ491453)
Micrococcus endophyticus (EU005372)
Micrococcus cohnii (FR832424)
Micrococcus antarcticus (AJ005932)
Micrococcus lylae (TYGS 3990)
Citricoccus zhacaiensis (EU305672)
Citricoccus muralis (AJ344143)
Citricoccus alkalitolerans (AY376164)
Citricoccus nitrophenolicus (GU797177)
Citricoccus parietis (FM992367)
Pseudoglutamicibacter albus (AJ243421)
Pseudoglutamicibacter cumminsii (X93354)
Nesterenkonia lutea (AY588278)
Nesterenkonia aurantiaca (HG795012)
Nesterenkonia halotolerans (AY226508)
Nesterenkonia sandarakina (AY588277)
Nesterenkonia jeotgali (AY928901)
Nesterenkonia aethiopica (AY574575)
Nesterenkonia xinjiangensis (AY226510)
Nesterenkonia suensis (FJ948172)
Nesterenkonia flava (EF680886)
Nesterenkonia lacusekhoensis (AJ290397)
Garicola koreensis (JX152781)
Nesterenkonia alkaliphila (KF914157)
Nesterenkonia alba (TYGS 1522)
Nesterenkonia populi (KP057085)
Nesterenkonia massiliensis (TYGS 5106)
Nesterenkonia rhizosphaerae (KF040423)
Nesterenkonia halophila (AY820953)
Nesterenkonia halobia (X80747)
Kocuria rosea (X87756)
Kocuria himachalensis (AY987383)
Kocuria polaris (TYGS 3188)
Kocuria dechangensis (JQ762279)
Kocuria flava (TYGS 3187)
Kocuria oceani (JF346427)
Kocuria turfanensis (TYGS 5359)
Kocuria aegyptia (DQ059617)
Kocuria marina (AY211385)
Kocuria indica (TYGS 22377)
Kocuria varians (X87754)
Kocuria salsicia (GQ352404)
Kocuria rhizophila (Y16264)
Kocuria arsenatis (KM874399)
Kocuria atrinae (FJ607311)
Kocuria gwangalliensis (EU286964)
Kocuria carniphila (AJ622907)
Kocuria pelophila (LC042214)
Kocuria subflava (KR610332)
Kocuria palustris (Y16263)

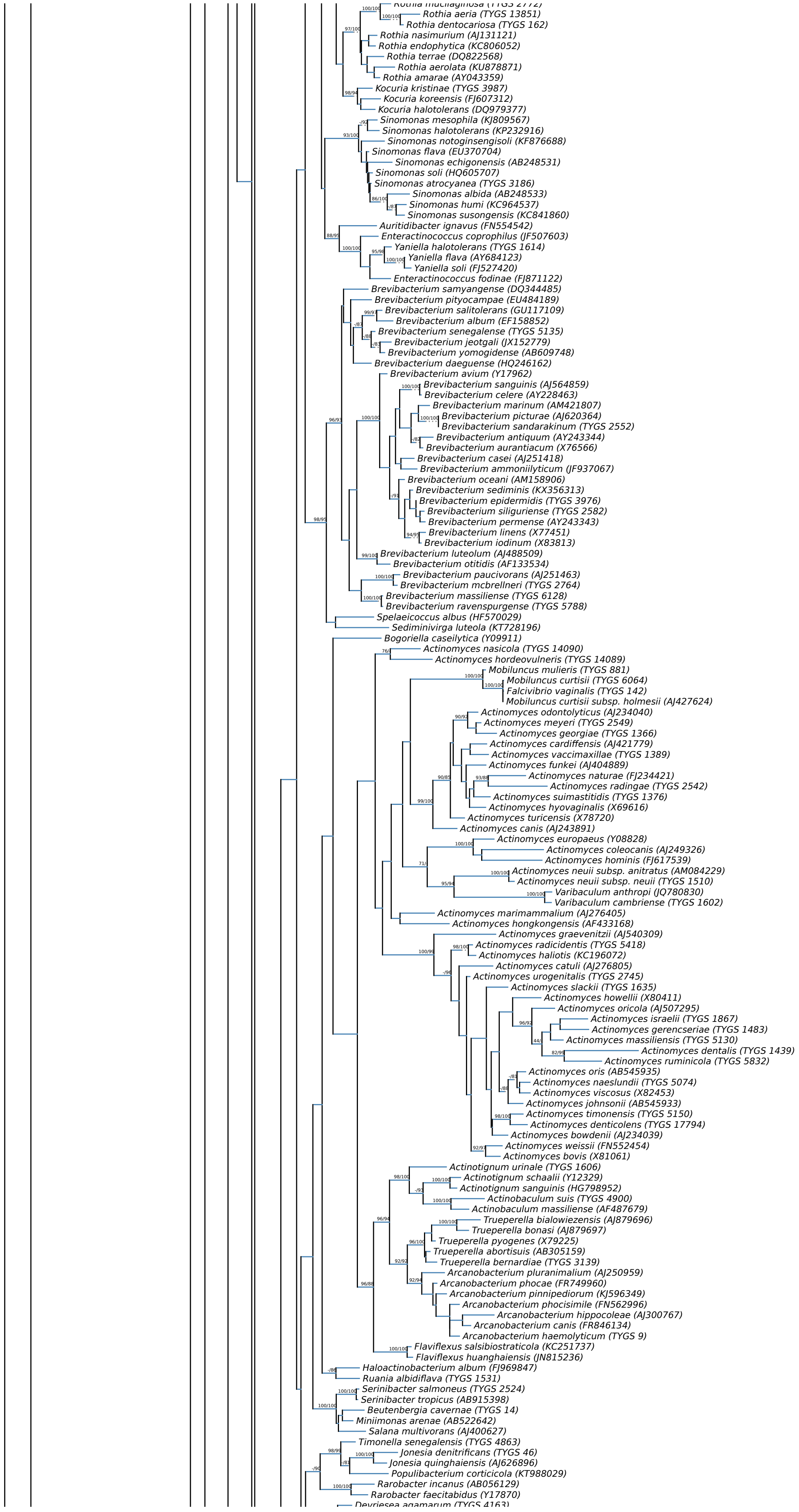
Rothia mucilaginosa (TYGS 2772)
Rothia aeria (TYGS 13851)
Rothia dentocariosa (TYGS 162)
Rothia nasimurium (AJ131121)
Rothia endophytica (KC806052)
Rothia terrae (DQ822568)
Rothia aerolata (KU878871)
Rothia amarae (AY043359)
Kocuria kristinae (TYGS 3987)
Kocuria koreensis (FJ607312)
Kocuria halotolerans (DQ979377)
Sinomonas mesophila (KJ809567)
Sinomonas halotolerans (KP232916)
Sinomonas notoginsengisoli (KF876688)
Sinomonas flava (EU370704)
Sinomonas echigonensis (AB248531)
Sinomonas soli (HQ605707)
Sinomonas atrocyanea (TYGS 3186)
Sinomonas albida (AB248533)
Sinomonas humi (KC964537)
Sinomonas susongensis (KC841860)
Auritidibacter ignavus (FN554542)
Enteractinococcus coprophilus (JF507603)
Yaniella halotolerans (TYGS 1614)
Yaniella flava (AY684123)
Yaniella soli (FJ527420)
Enteractinococcus fodinae (FJ871122)
Brevibacterium samyangense (DQ344485)
Brevibacterium pityocampae (EU484189)
Brevibacterium salitolerans (GU117109)
Brevibacterium album (EF158852)
Brevibacterium senegalense (TYGS 5135)
Brevibacterium jeotgali (JX152779)
Brevibacterium yomogidense (AB609748)
Brevibacterium daeguense (HQ246162)
Brevibacterium avium (Y17962)
Brevibacterium sanguinis (AJ564859)
Brevibacterium celere (AY228463)
Brevibacterium marinum (AM421807)
Brevibacterium picturae (AJ620364)
Brevibacterium sandarakinum (TYGS 2552)
Brevibacterium antiquum (AY243344)
Brevibacterium aurantiacum (X76566)
Brevibacterium casei (AJ251418)
Brevibacterium ammoniilyticum (JF937067)
Brevibacterium oceani (AM158906)
Brevibacterium sediminis (KX356313)
Brevibacterium epidermidis (TYGS 3976)
Brevibacterium siliguriense (TYGS 2582)
Brevibacterium permense (AY243343)
Brevibacterium linens (X77451)
Brevibacterium iodinum (X83813)
Brevibacterium luteolum (AJ488509)
Brevibacterium otitidis (AF133534)
Brevibacterium paucivorans (AJ251463)
Brevibacterium mcbrellneri (TYGS 2764)
Brevibacterium massiliense (TYGS 6128)
Brevibacterium ravenspurgense (TYGS 5788)
Spelaeicoccus albus (HF570029)
Sediminivirga luteola (KT728196)
Bogoriella caseilytica (Y09911)
Actinomyces nasicola (TYGS 14090)
Actinomyces hordeovulneris (TYGS 14089)
Mobiluncus mulieris (TYGS 881)
Mobiluncus curtisii (TYGS 6064)
Falcivibrio vaginalis (TYGS 142)
Mobiluncus curtisii subsp. holmesii (AJ427624)
Actinomyces odontolyticus (AJ234040)
Actinomyces meyeri (TYGS 2549)
Actinomyces georgiae (TYGS 1366)
Actinomyces cardiffensis (AJ421779)
Actinomyces vaccimaxillae (TYGS 1389)
Actinomyces funkei (AJ404889)
Actinomyces naturae (FJ234421)
Actinomyces radingae (TYGS 2542)
Actinomyces suimastitidis (TYGS 1376)
Actinomyces hyovaginalis (X69616)
Actinomyces turicensis (X78720)
Actinomyces canis (AJ243891)
Actinomyces europaeus (Y08828)
Actinomyces coleocanis (AJ249326)
Actinomyces hominis (FJ617539)
Actinomyces neuii subsp. anitratus (AM084229)
Actinomyces neuii subsp. neuii (TYGS 1510)
Varibaculum anthropi (JQ780830)
Varibaculum cambriense (TYGS 1602)
Actinomyces marimammalium (AJ276405)
Actinomyces hongkongensis (AF433168)
Actinomyces graevenitzii (AJ540309)
Actinomyces radicidentis (TYGS 5418)
Actinomyces haliotis (KC196072)
Actinomyces catuli (AJ276805)
Actinomyces urogenitalis (TYGS 2745)
Actinomyces slackii (TYGS 1635)
Actinomyces howellii (X80411)
Actinomyces oricola (AJ507295)
Actinomyces israelii (TYGS 1867)
Actinomyces gerencseriae (TYGS 1483)
Actinomyces massiliensis (TYGS 5130)
Actinomyces dentalis (TYGS 1439)
Actinomyces ruminicola (TYGS 5832)
Actinomyces oris (AB545935)
Actinomyces naeslundii (TYGS 5074)
Actinomyces viscosus (X82453)
Actinomyces johnsonii (AB545933)
Actinomyces timonensis (TYGS 5150)
Actinomyces denticolens (TYGS 17794)
Actinomyces bowdenii (AJ234039)
Actinomyces weissii (FN552454)
Actinomyces bovis (X81061)
Actinotignum urinale (TYGS 1606)
Actinotignum schaalii (Y12329)
Actinotignum sanguinis (HG798952)
Actinobaculum suis (TYGS 4900)
Actinobaculum massiliense (AF487679)
Trueperella bialowiezensis (AJ879696)
Trueperella bonasi (AJ879697)
Trueperella pyogenes (X79225)
Trueperella abortisuis (AB305159)
Trueperella bernardiae (TYGS 3139)
Arcanobacterium pluranimalium (AJ250959)
Arcanobacterium phocae (FR749960)
Arcanobacterium pinnipediorum (KJ596349)
Arcanobacterium phocisimile (FN562996)
Arcanobacterium hippocoleae (AJ300767)
Arcanobacterium canis (FR846134)
Arcanobacterium haemolyticum (TYGS 9)
Flaviflexus salsibiostraticola (KC251737)
Flaviflexus huanghaiensis (JN815236)
Haloactinobacterium album (FJ969847)
Ruania albidiflava (TYGS 1531)
Serinibacter salmoneus (TYGS 2524)
Serinibacter tropicus (AB915398)
Beutenbergia cavernae (TYGS 14)
Miniimonas arenae (AB522642)
Salana multivorans (AJ400627)
Timonella senegalensis (TYGS 4863)
Jonesia denitrificans (TYGS 46)
Jonesia quinghaiensis (AJ626896)
Populibacterium corticicola (KT988029)
Rarobacter incanus (AB056129)
Rarobacter faecitabidus (Y17870)
Devriesea agamarum (TYGS 4163)

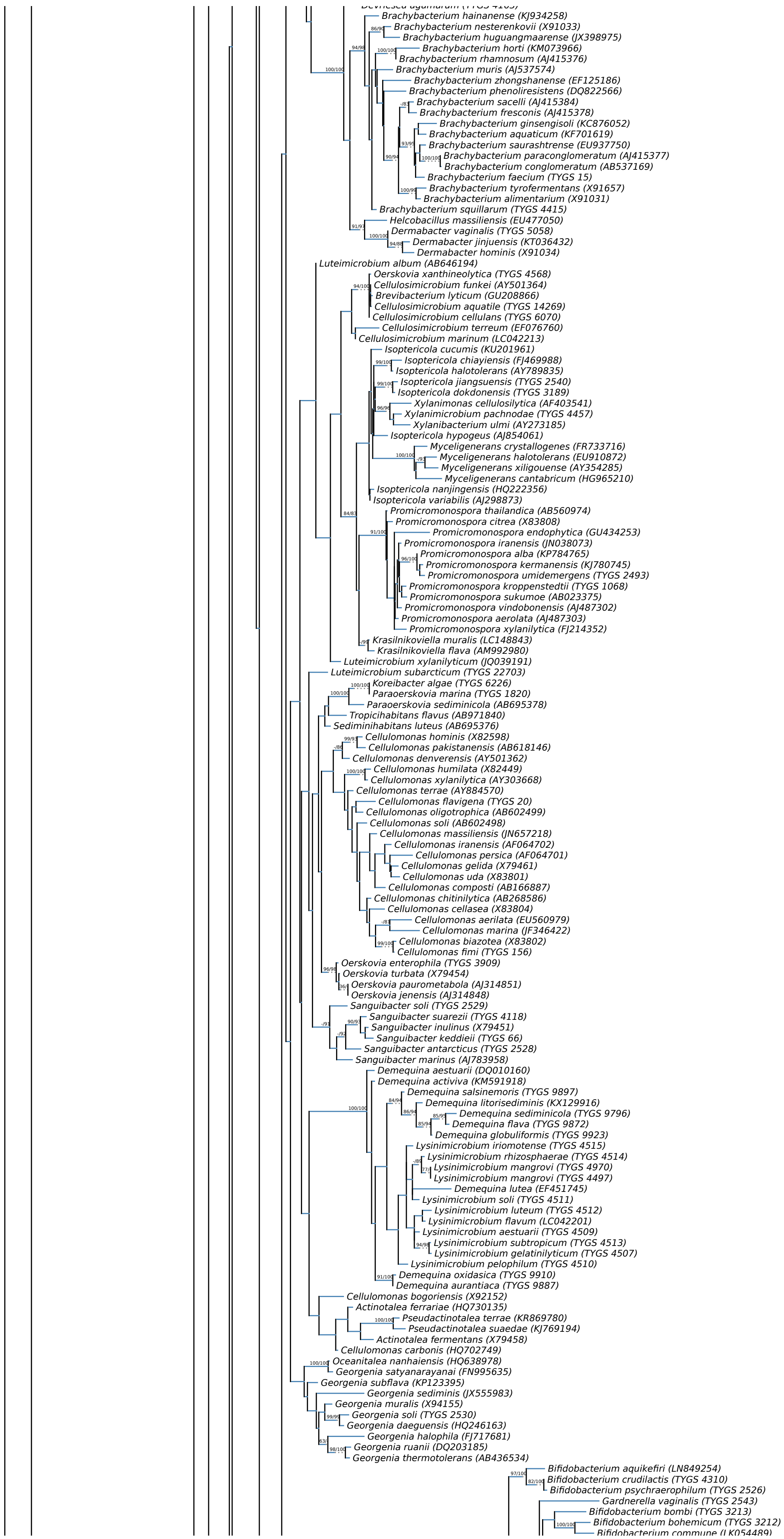
Brachybacterium hainanense (KJ934258)
Brachybacterium nesterenkovii (X91033)
Brachybacterium huguangmaarense (JX398975)
Brachybacterium horti (KM073966)
Brachybacterium rhamnosum (AJ415376)
Brachybacterium muris (AJ537574)
Brachybacterium zhongshanense (EF125186)
Brachybacterium phenoliresistens (DQ822566)
Brachybacterium sacelli (AJ415384)
Brachybacterium fresconis (AJ415378)
Brachybacterium ginsengisoli (KC876052)
Brachybacterium aquaticum (KF701619)
Brachybacterium saurashtrense (EU937750)
Brachybacterium paraconglomeratum (AJ415377)
Brachybacterium conglomeratum (AB537169)
Brachybacterium faecium (TYGS 15)
Brachybacterium tyrofermentans (X91657)
Brachybacterium alimentarium (X91031)
Brachybacterium squillarum (TYGS 4415)
Helcobacillus massiliensis (EU477050)
Dermabacter vaginalis (TYGS 5058)
Dermabacter jinjuensis (KT036432)
Dermabacter hominis (X91034)
Luteimicrobium album (AB646194)
Oerskovia xanthineolytica (TYGS 4568)
Cellulosimicrobium funkei (AY501364)
Brevibacterium lyticum (GU208866)
Cellulosimicrobium aquatile (TYGS 14269)
Cellulosimicrobium cellulans (TYGS 6070)
Cellulosimicrobium terreum (EF076760)
Cellulosimicrobium marinum (LC042213)
Isoptericola cucumis (KU201961)
Isoptericola chiayiensis (FJ469988)
Isoptericola halotolerans (AY789835)
Isoptericola jiangsuensis (TYGS 2540)
Isoptericola dokdonensis (TYGS 3189)
Xylanimonas cellulosilytica (AF403541)
Xylanimicrobium pachnodae (TYGS 4457)
Xylanibacterium ulmi (AY273185)
Isoptericola hypogeus (AJ854061)
Myceligenerans crystallogenes (FR733716)
Myceligenerans halotolerans (EU910872)
Myceligenerans xiligouense (AY354285)
Myceligenerans cantabricum (HG965210)
Isoptericola nanjingensis (HQ222356)
Isoptericola variabilis (AJ298873)
Promicromonospora thailandica (AB560974)
Promicromonospora citrea (X83808)
Promicromonospora endophytica (GU434253)
Promicromonospora iranensis (JN038073)
Promicromonospora alba (KP784765)
Promicromonospora kermanensis (KJ780745)
Promicromonospora umidemergens (TYGS 2493)
Promicromonospora kroppenstedtii (TYGS 1068)
Promicromonospora sukumoe (AB023375)
Promicromonospora vindobonensis (AJ487302)
Promicromonospora aerolata (AJ487303)
Promicromonospora xylanilytica (FJ214352)
Krasilnikoviella muralis (LC148843)
Krasilnikoviella flava (AM992980)
Luteimicrobium xylanilyticum (JQ039191)
Luteimicrobium subarcticum (TYGS 22703)
Koreibacter algae (TYGS 6226)
Paraoerskovia marina (TYGS 1820)
Paraoerskovia sediminicola (AB695378)
Tropicihabitans flavus (AB971840)
Sediminihabitans luteus (AB695376)
Cellulomonas hominis (X82598)
Cellulomonas pakistanensis (AB618146)
Cellulomonas denverensis (AY501362)
Cellulomonas humilata (X82449)
Cellulomonas xylanilytica (AY303668)
Cellulomonas terrae (AY884570)
Cellulomonas flavigena (TYGS 20)
Cellulomonas oligotrophica (AB602499)
Cellulomonas soli (AB602498)
Cellulomonas massiliensis (JN657218)
Cellulomonas iranensis (AF064702)
Cellulomonas persica (AF064701)
Cellulomonas gelida (X79461)
Cellulomonas uda (X83801)
Cellulomonas composti (AB166887)
Cellulomonas chitinilytica (AB268586)
Cellulomonas cellasea (X83804)
Cellulomonas aerilata (EU560979)
Cellulomonas marina (JF346422)
Cellulomonas biazotea (X83802)
Cellulomonas fimi (TYGS 156)
Oerskovia enterophila (TYGS 3909)
Oerskovia turbata (X79454)
Oerskovia paurometabola (AJ314851)
Oerskovia jenensis (AJ314848)
Sanguibacter soli (TYGS 2529)
Sanguibacter suarezii (TYGS 4118)
Sanguibacter inulinus (X79451)
Sanguibacter keddieii (TYGS 66)
Sanguibacter antarcticus (TYGS 2528)
Sanguibacter marinus (AJ783958)
Demequina aestuarii (DQ010160)
Demequina activiva (KM591918)
Demequina salsinemoris (TYGS 9897)
Demequina litorisediminis (KX129916)
Demequina sediminicola (TYGS 9796)
Demequina flava (TYGS 9872)
Demequina globuliformis (TYGS 9923)
Lysinimicrobium iriomotense (TYGS 4515)
Lysinimicrobium rhizosphaerae (TYGS 4514)
Lysinimicrobium mangrovi (TYGS 4970)
Lysinimicrobium mangrovi (TYGS 4497)
Demequina lutea (EF451745)
Lysinimicrobium soli (TYGS 4511)
Lysinimicrobium luteum (TYGS 4512)
Lysinimicrobium flavum (LC042201)
Lysinimicrobium aestuarii (TYGS 4509)
Lysinimicrobium subtropicum (TYGS 4513)
Lysinimicrobium gelatinilyticum (TYGS 4507)
Lysinimicrobium pelophilum (TYGS 4510)
Demequina oxidasica (TYGS 9910)
Demequina aurantiaca (TYGS 9887)
Cellulomonas bogoriensis (X92152)
Actinotalea ferrariae (HQ730135)
Pseudactinotalea terrae (KR869780)
Pseudactinotalea suaedae (KJ769194)
Actinotalea fermentans (X79458)
Cellulomonas carbonis (HQ702749)
Oceanitalea nanhaiensis (HQ638978)
Georgenia satyanarayanai (FN995635)
Georgenia subflava (KP123395)
Georgenia sediminis (JX555983)
Georgenia muralis (X94155)
Georgenia soli (TYGS 2530)
Georgenia daeguensis (HQ246163)
Georgenia halophila (FJ717681)
Georgenia ruanii (DQ203185)
Georgenia thermotolerans (AB436534)
Bifidobacterium aquikefiri (LN849254)
Bifidobacterium crudilactis (TYGS 4310)
Bifidobacterium psychraerophilum (TYGS 2526)
Gardnerella vaginalis (TYGS 2543)
Bifidobacterium bombi (TYGS 3213)
Bifidobacterium bohemicum (TYGS 3212)
Bifidobacterium commune (LK054489)

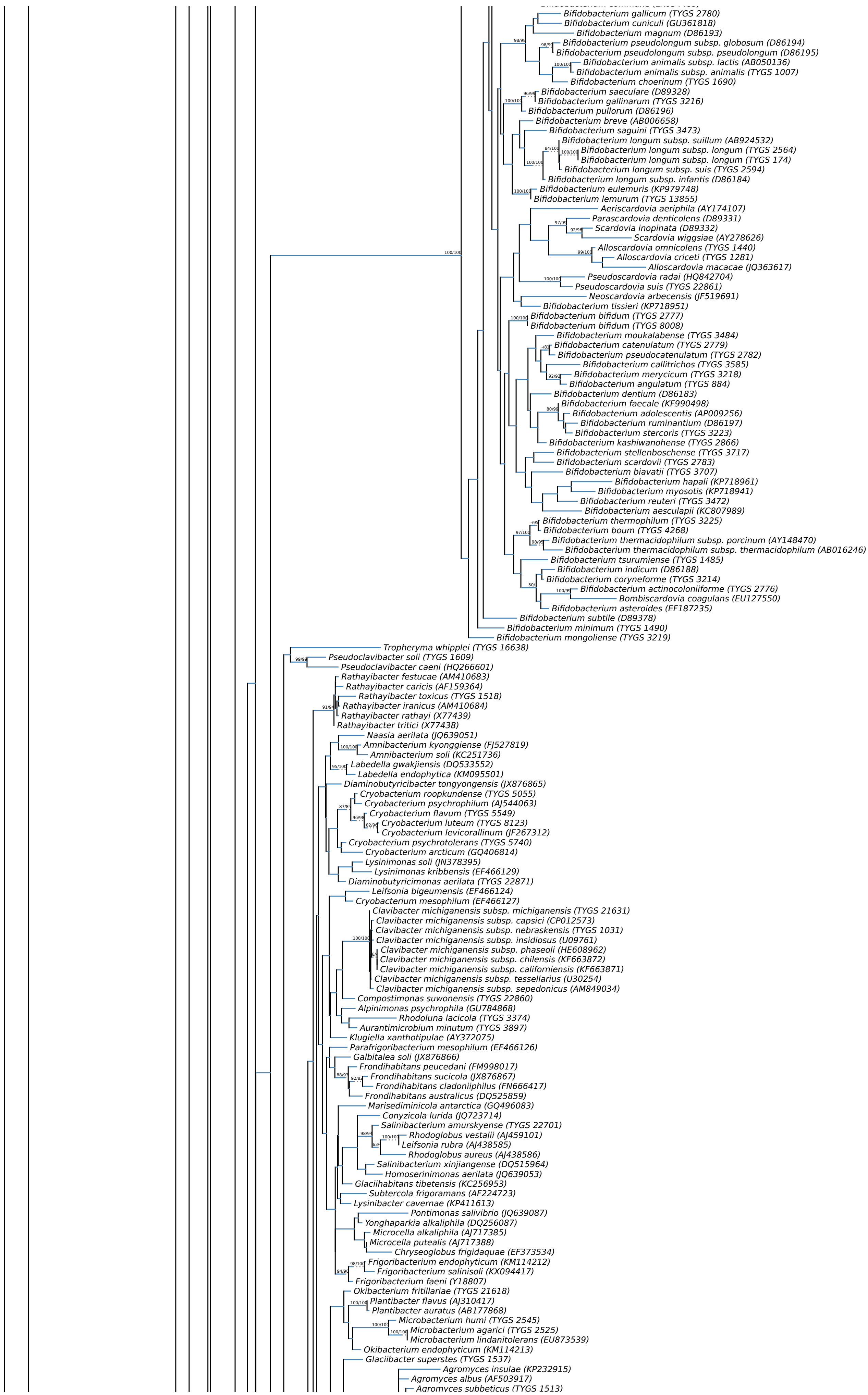
Bifidobacterium gallicum (TYGS 2780)
Bifidobacterium cuniculi (GU361818)
Bifidobacterium magnum (D86193)
Bifidobacterium pseudolongum subsp. globosum (D86194)
Bifidobacterium pseudolongum subsp. pseudolongum (D86195)
Bifidobacterium animalis subsp. lactis (AB050136)
Bifidobacterium animalis subsp. animalis (TYGS 1007)
Bifidobacterium choerinum (TYGS 1690)
Bifidobacterium saeculare (D89328)
Bifidobacterium gallinarum (TYGS 3216)
Bifidobacterium pullorum (D86196)
Bifidobacterium breve (AB006658)
Bifidobacterium saguini (TYGS 3473)
Bifidobacterium longum subsp. suillum (AB924532)
Bifidobacterium longum subsp. longum (TYGS 2564)
Bifidobacterium longum subsp. longum (TYGS 174)
Bifidobacterium longum subsp. suis (TYGS 2594)
Bifidobacterium longum subsp. infantis (D86184)
Bifidobacterium eulemuris (KP979748)
Bifidobacterium lemurum (TYGS 13855)
Aeriscardovia aeriphila (AY174107)
Parascardovia denticolens (D89331)
Scardovia inopinata (D89332)
Scardovia wiggsiae (AY278626)
Alloscardovia omnicolens (TYGS 1440)
Alloscardovia criceti (TYGS 1281)
Alloscardovia macacae (JQ363617)
Pseudoscardovia radai (HQ842704)
Pseudoscardovia suis (TYGS 22861)
Neoscardovia arbecensis (JF519691)
Bifidobacterium tissieri (KP718951)
Bifidobacterium bifidum (TYGS 2777)
Bifidobacterium bifidum (TYGS 8008)
Bifidobacterium moukalabense (TYGS 3484)
Bifidobacterium catenulatum (TYGS 2779)
Bifidobacterium pseudocatenulatum (TYGS 2782)
Bifidobacterium callitrichos (TYGS 3585)
Bifidobacterium merycicum (TYGS 3218)
Bifidobacterium angulatum (TYGS 884)
Bifidobacterium dentium (D86183)
Bifidobacterium faecale (KF990498)
Bifidobacterium adolescentis (AP009256)
Bifidobacterium ruminantium (D86197)
Bifidobacterium stercoris (TYGS 3223)
Bifidobacterium kashiwanohense (TYGS 2866)
Bifidobacterium stellenboschense (TYGS 3717)
Bifidobacterium scardovii (TYGS 2783)
Bifidobacterium biavatii (TYGS 3707)
Bifidobacterium hapali (KP718961)
Bifidobacterium myosotis (KP718941)
Bifidobacterium reuteri (TYGS 3472)
Bifidobacterium aesculapii (KC807989)
Bifidobacterium thermophilum (TYGS 3225)
Bifidobacterium boum (TYGS 4268)
Bifidobacterium thermacidophilum subsp. porcinum (AY148470)
Bifidobacterium thermacidophilum subsp. thermacidophilum (AB016246)
Bifidobacterium tsurumiense (TYGS 1485)
Bifidobacterium indicum (D86188)
Bifidobacterium coryneforme (TYGS 3214)
Bifidobacterium actinocoloniiforme (TYGS 2776)
Bombiscardovia coagulans (EU127550)
Bifidobacterium asteroides (EF187235)
Bifidobacterium subtile (D89378)
Bifidobacterium minimum (TYGS 1490)
Bifidobacterium mongoliense (TYGS 3219)
Tropheryma whipplei (TYGS 16638)
Pseudoclavibacter soli (TYGS 1609)
Pseudoclavibacter caeni (HQ266601)
Rathayibacter festucae (AM410683)
Rathayibacter caricis (AF159364)
Rathayibacter toxicus (TYGS 1518)
Rathayibacter iranicus (AM410684)
Rathayibacter rathayi (X77439)
Rathayibacter tritici (X77438)
Naasia aerilata (JQ639051)
Amnibacterium kyonggiense (FJ527819)
Amnibacterium soli (KC251736)
Labedella gwakjiensis (DQ533552)
Labedella endophytica (KM095501)
Diaminobutyricibacter tongyongensis (JX876865)
Cryobacterium roopkundense (TYGS 5055)
Cryobacterium psychrophilum (AJ544063)
Cryobacterium flavum (TYGS 5549)
Cryobacterium luteum (TYGS 8123)
Cryobacterium levicorallinum (JF267312)
Cryobacterium psychrotolerans (TYGS 5740)
Cryobacterium arcticum (GQ406814)
Lysinimonas soli (JN378395)
Lysinimonas kribbensis (EF466129)
Diaminobutyricimonas aerilata (TYGS 22871)
Leifsonia bigeumensis (EF466124)
Cryobacterium mesophilum (EF466127)
Clavibacter michiganensis subsp. michiganensis (TYGS 21631)
Clavibacter michiganensis subsp. capsici (CP012573)
Clavibacter michiganensis subsp. nebraskensis (TYGS 1031)
Clavibacter michiganensis subsp. insidiosus (U09761)
Clavibacter michiganensis subsp. phaseoli (HE608962)
Clavibacter michiganensis subsp. chilensis (KF663872)
Clavibacter michiganensis subsp. californiensis (KF663871)
Clavibacter michiganensis subsp. tessellarius (U30254)
Clavibacter michiganensis subsp. sepedonicus (AM849034)
Compostimonas suwonensis (TYGS 22860)
Alpinimonas psychrophila (GU784868)
Rhodoluna lacicola (TYGS 3374)
Aurantimicrobium minutum (TYGS 3897)
Klugiella xanthotipulae (AY372075)
Parafrigoribacterium mesophilum (EF466126)
Galbitalea soli (JX876866)
Frondihabitans peucedani (FM998017)
Frondihabitans sucicola (JX876867)
Frondihabitans cladoniiphilus (FN666417)
Frondihabitans australicus (DQ525859)
Marisediminicola antarctica (GQ496083)
Conyzicola lurida (JQ723714)
Salinibacterium amurskyense (TYGS 22701)
Rhodoglobus vestalii (AJ459101)
Leifsonia rubra (AJ438585)
Rhodoglobus aureus (AJ438586)
Salinibacterium xinjiangense (DQ515964)
Homoserinimonas aerilata (JQ639053)
Glaciihabitans tibetensis (KC256953)
Subtercola frigoramans (AF224723)
Lysinibacter cavernae (KP411613)
Pontimonas salivibrio (JQ639087)
Yonghaparkia alkaliphila (DQ256087)
Microcella alkaliphila (AJ717385)
Microcella putealis (AJ717388)
Chryseoglobus frigidaquae (EF373534)
Frigoribacterium endophyticum (KM114212)
Frigoribacterium salinisoli (KX094417)
Frigoribacterium faeni (Y18807)
Okibacterium fritillariae (TYGS 21618)
Plantibacter flavus (AJ310417)
Plantibacter auratus (AB177868)
Microbacterium humi (TYGS 2545)
Microbacterium agarici (TYGS 2525)
Microbacterium lindanitolerans (EU873539)
Okibacterium endophyticum (KM114213)
Glaciibacter superstes (TYGS 1537)
Agromyces insulae (KP232915)
Agromyces albus (AF503917)
Agromyces subbeticus (TYGS 1513)

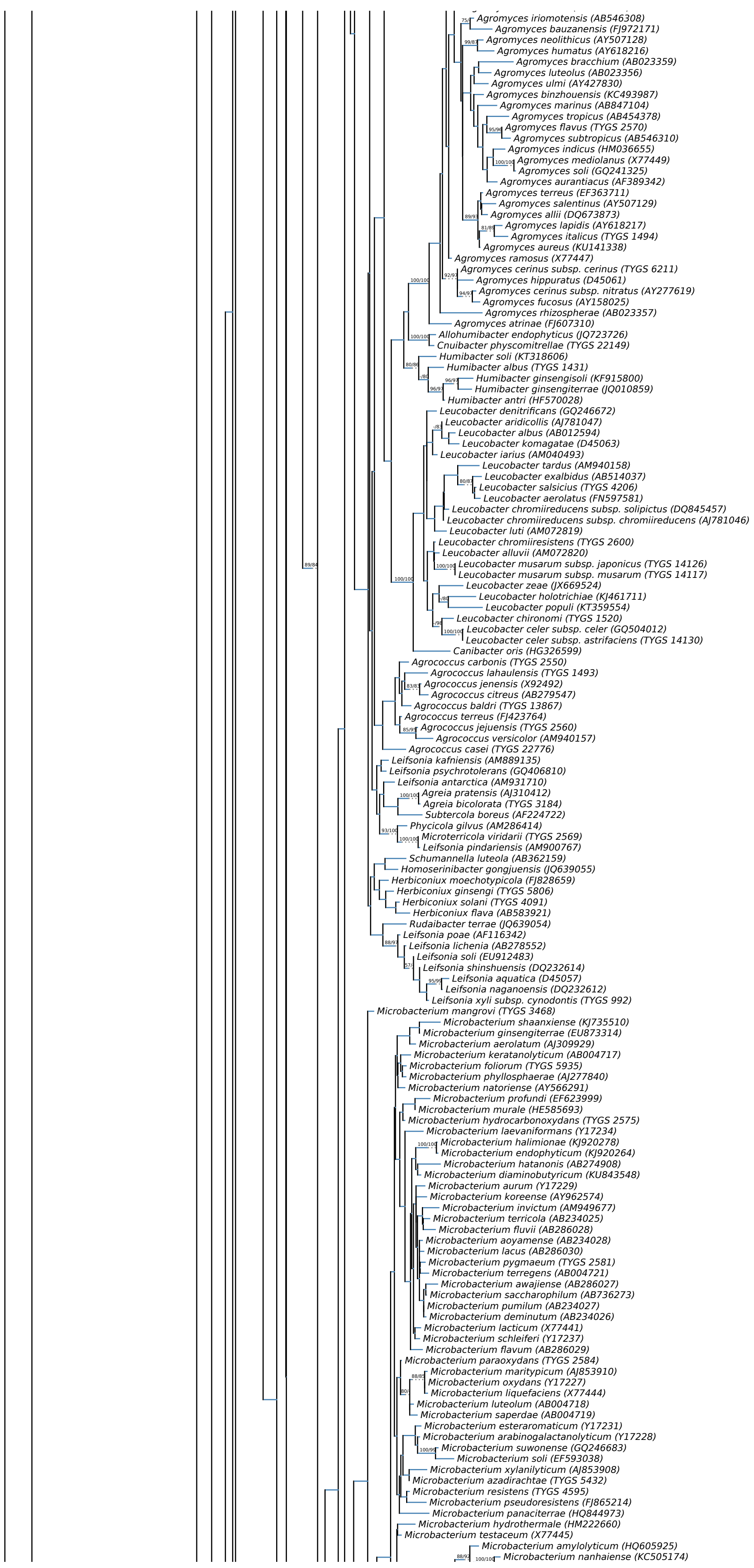

Agromyces iriomotensis (AB546308)
Agromyces bauzanensis (FJ972171)
Agromyces neolithicus (AY507128)
Agromyces humatus (AY618216)
Agromyces bracchium (AB023359)
Agromyces luteolus (AB023356)
Agromyces ulmi (AY427830)
Agromyces binzhouensis (KC493987)
Agromyces marinus (AB847104)
Agromyces tropicus (AB454378)
Agromyces flavus (TYGS 2570)
Agromyces subtropicus (AB546310)
Agromyces indicus (HM036655)
Agromyces mediolanus (X77449)
Agromyces soli (GQ241325)
Agromyces aurantiacus (AF389342)
Agromyces terreus (EF363711)
Agromyces salentinus (AY507129)
Agromyces allii (DQ673873)
Agromyces lapidis (AY618217)
Agromyces italicus (TYGS 1494)
Agromyces aureus (KU141338)
Agromyces ramosus (X77447)
Agromyces cerinus subsp. cerinus (TYGS 6211)
Agromyces hippuratus (D45061)
Agromyces cerinus subsp. nitratus (AY277619)
Agromyces fucosus (AY158025)
Agromyces rhizospherae (AB023357)
Agromyces atrinae (FJ607310)
Allohumibacter endophyticus (JQ723726)
Cnuibacter physcomitrellae (TYGS 22149)
Humibacter soli (KT318606)
Humibacter albus (TYGS 1431)
Humibacter ginsengisoli (KF915800)
Humibacter ginsengiterrae (JQ010859)
Humibacter antri (HF570028)
Leucobacter denitrificans (GQ246672)
Leucobacter aridicollis (AJ781047)
Leucobacter albus (AB012594)
Leucobacter komagatae (D45063)
Leucobacter iarius (AM040493)
Leucobacter tardus (AM940158)
Leucobacter exalbidus (AB514037)
Leucobacter salsicius (TYGS 4206)
Leucobacter aerolatus (FN597581)
Leucobacter chromiireducens subsp. solipictus (DQ845457)
Leucobacter chromiireducens subsp. chromiireducens (AJ781046)
Leucobacter luti (AM072819)
Leucobacter chromiiresistens (TYGS 2600)
Leucobacter alluvii (AM072820)
Leucobacter musarum subsp. japonicus (TYGS 14126)
Leucobacter musarum subsp. musarum (TYGS 14117)
Leucobacter zeae (JX669524)
Leucobacter holotrichiae (KJ461711)
Leucobacter populi (KT359554)
Leucobacter chironomi (TYGS 1520)
Leucobacter celer subsp. celer (GQ504012)
Leucobacter celer subsp. astrifaciens (TYGS 14130)
Canibacter oris (HG326599)
Agrococcus carbonis (TYGS 2550)
Agrococcus lahaulensis (TYGS 1493)
Agrococcus jenensis (X92492)
Agrococcus citreus (AB279547)
Agrococcus baldri (TYGS 13867)
Agrococcus terreus (FJ423764)
Agrococcus jejuensis (TYGS 2560)
Agrococcus versicolor (AM940157)
Agrococcus casei (TYGS 22776)
Leifsonia kafniensis (AM889135)
Leifsonia psychrotolerans (GQ406810)
Leifsonia antarctica (AM931710)
Agreia pratensis (AJ310412)
Agreia bicolorata (TYGS 3184)
Subtercola boreus (AF224722)
Phycicola gilvus (AM286414)
Microterricola viridarii (TYGS 2569)
Leifsonia pindariensis (AM900767)
Schumannella luteola (AB362159)
Homoserinibacter gongjuensis (JQ639055)
Herbiconiux moechotypicola (FJ828659)
Herbiconiux ginsengi (TYGS 5806)
Herbiconiux solani (TYGS 4091)
Herbiconiux flava (AB583921)
Rudaibacter terrae (JQ639054)
Leifsonia poae (AF116342)
Leifsonia lichenia (AB278552)
Leifsonia soli (EU912483)
Leifsonia shinshuensis (DQ232614)
Leifsonia aquatica (D45057)
Leifsonia naganoensis (DQ232612)
Leifsonia xyli subsp. cynodontis (TYGS 992)
Microbacterium mangrovi (TYGS 3468)
Microbacterium shaanxiense (KJ735510)
Microbacterium ginsengiterrae (EU873314)
Microbacterium aerolatum (AJ309929)
Microbacterium keratanolyticum (AB004717)
Microbacterium foliorum (TYGS 5935)
Microbacterium phyllosphaerae (AJ277840)
Microbacterium natoriense (AY566291)
Microbacterium profundi (EF623999)
Microbacterium murale (HE585693)
Microbacterium hydrocarbonoxydans (TYGS 2575)
Microbacterium laevaniformans (Y17234)
Microbacterium halimionae (KJ920278)
Microbacterium endophyticum (KJ920264)
Microbacterium hatanonis (AB274908)
Microbacterium diaminobutyricum (KU843548)
Microbacterium aurum (Y17229)
Microbacterium koreense (AY962574)
Microbacterium invictum (AM949677)
Microbacterium terricola (AB234025)
Microbacterium fluvii (AB286028)
Microbacterium aoyamense (AB234028)
Microbacterium lacus (AB286030)
Microbacterium pygmaeum (TYGS 2581)
Microbacterium terregens (AB004721)
Microbacterium awajiense (AB286027)
Microbacterium saccharophilum (AB736273)
Microbacterium pumilum (AB234027)
Microbacterium deminutum (AB234026)
Microbacterium lacticum (X77441)
Microbacterium schleiferi (Y17237)
Microbacterium flavum (AB286029)
Microbacterium paraoxydans (TYGS 2584)
Microbacterium maritypicum (AJ853910)
Microbacterium oxydans (Y17227)
Microbacterium liquefaciens (X77444)
Microbacterium luteolum (AB004718)
Microbacterium saperdae (AB004719)
Microbacterium esteraromaticum (Y17231)
Microbacterium arabinogalactanolyticum (Y17228)
Microbacterium suwonense (GQ246683)
Microbacterium soli (EF593038)
Microbacterium xylanilyticum (AJ853908)
Microbacterium azadirachtae (TYGS 5432)
Microbacterium resistens (TYGS 4595)
Microbacterium pseudoresistens (FJ865214)
Microbacterium panaciterrae (HQ844973)
Microbacterium hydrothermale (HM222660)
Microbacterium testaceum (X77445)
Microbacterium amylolyticum (HQ605925)
Microbacterium nanhaiense (KC505174)

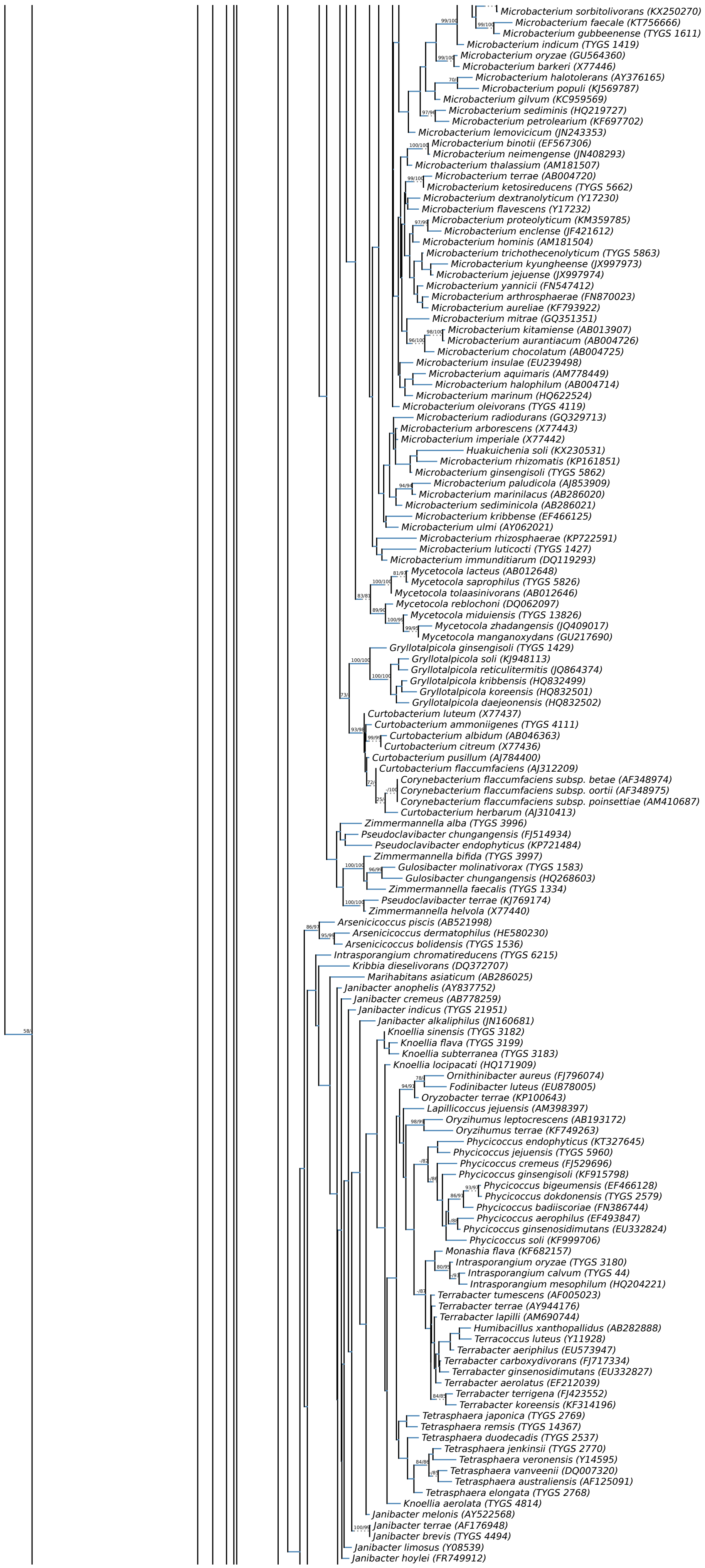

Microbacterium sorbitolivorans (KX250270)
Microbacterium faecale (KT756666)
Microbacterium gubbeenense (TYGS 1611)
Microbacterium indicum (TYGS 1419)
Microbacterium oryzae (GU564360)
Microbacterium barkeri (X77446)
Microbacterium halotolerans (AY376165)
Microbacterium populi (KJ569787)
Microbacterium gilvum (KC959569)
Microbacterium sediminis (HQ219727)
Microbacterium petrolearium (KF697702)
Microbacterium lemovicicum (JN243353)
Microbacterium binotii (EF567306)
Microbacterium neimengense (JN408293)
Microbacterium thalassium (AM181507)
Microbacterium terrae (AB004720)
Microbacterium ketosireducens (TYGS 5662)
Microbacterium dextranolyticum (Y17230)
Microbacterium flavescens (Y17232)
Microbacterium proteolyticum (KM359785)
Microbacterium enclense (JF421612)
Microbacterium hominis (AM181504)
Microbacterium trichothecenolyticum (TYGS 5863)
Microbacterium kyungheense (JX997973)
Microbacterium jejuense (JX997974)
Microbacterium yannicii (FN547412)
Microbacterium arthrosphaerae (FN870023)
Microbacterium aureliae (KF793922)
Microbacterium mitrae (GQ351351)
Microbacterium kitamiense (AB013907)
Microbacterium aurantiacum (AB004726)
Microbacterium chocolatum (AB004725)
Microbacterium insulae (EU239498)
Microbacterium aquimaris (AM778449)
Microbacterium halophilum (AB004714)
Microbacterium marinum (HQ622524)
Microbacterium oleivorans (TYGS 4119)
Microbacterium radiodurans (GQ329713)
Microbacterium arborescens (X77443)
Microbacterium imperiale (X77442)
Huakuichenia soli (KX230531)
Microbacterium rhizomatis (KP161851)
Microbacterium ginsengisoli (TYGS 5862)
Microbacterium paludicola (AJ853909)
Microbacterium marinilacus (AB286020)
Microbacterium sediminicola (AB286021)
Microbacterium kribbense (EF466125)
Microbacterium ulmi (AY062021)
Microbacterium rhizosphaerae (KP722591)
Microbacterium luticocti (TYGS 1427)
Microbacterium immunditiarum (DQ119293)
Mycetocola lacteus (AB012648)
Mycetocola saprophilus (TYGS 5826)
Mycetocola tolaasinivorans (AB012646)
Mycetocola reblochoni (DQ062097)
Mycetocola miduiensis (TYGS 13826)
Mycetocola zhadangensis (JQ409017)
Mycetocola manganoxydans (GU217690)
Gryllotalpicola ginsengisoli (TYGS 1429)
Gryllotalpicola soli (KJ948113)
Gryllotalpicola reticulitermitis (JQ864374)
Gryllotalpicola kribbensis (HQ832499)
Gryllotalpicola koreensis (HQ832501)
Gryllotalpicola daejeonensis (HQ832502)
Curtobacterium luteum (X77437)
Curtobacterium ammoniigenes (TYGS 4111)
Curtobacterium albidum (AB046363)
Curtobacterium citreum (X77436)
Curtobacterium pusillum (AJ784400)
Curtobacterium flaccumfaciens (AJ312209)
Corynebacterium flaccumfaciens subsp. betae (AF348974)
Corynebacterium flaccumfaciens subsp. oortii (AF348975)
Corynebacterium flaccumfaciens subsp. poinsettiae (AM410687)
Curtobacterium herbarum (AJ310413)
Zimmermannella alba (TYGS 3996)
Pseudoclavibacter chungangensis (FJ514934)
Pseudoclavibacter endophyticus (KP721484)
Zimmermannella bifida (TYGS 3997)
Gulosibacter molinativorax (TYGS 1583)
Gulosibacter chungangensis (HQ268603)
Zimmermannella faecalis (TYGS 1334)
Pseudoclavibacter terrae (KJ769174)
Zimmermannella helvola (X77440)
Arsenicicoccus piscis (AB521998)
Arsenicicoccus dermatophilus (HE580230)
Arsenicicoccus bolidensis (TYGS 1536)
Intrasporangium chromatireducens (TYGS 6215)
Kribbia dieselivorans (DQ372707)
Marihabitans asiaticum (AB286025)
Janibacter anophelis (AY837752)
Janibacter cremeus (AB778259)
Janibacter indicus (TYGS 21951)
Janibacter alkaliphilus (JN160681)
Knoellia sinensis (TYGS 3182)
Knoellia flava (TYGS 3199)
Knoellia subterranea (TYGS 3183)
Knoellia locipacati (HQ171909)
Ornithinibacter aureus (FJ796074)
Fodinibacter luteus (EU878005)
Oryzobacter terrae (KP100643)
Lapillicoccus jejuensis (AM398397)
Oryzihumus leptocrescens (AB193172)
Oryzihumus terrae (KF749263)
Phycicoccus endophyticus (KT327645)
Phycicoccus jejuensis (TYGS 5960)
Phycicoccus cremeus (FJ529696)
Phycicoccus ginsengisoli (KF915798)
Phycicoccus bigeumensis (EF466128)
Phycicoccus dokdonensis (TYGS 2579)
Phycicoccus badiiscoriae (FN386744)
Phycicoccus aerophilus (EF493847)
Phycicoccus ginsenosidimutans (EU332824)
Phycicoccus soli (KF999706)
Monashia flava (KF682157)
Intrasporangium oryzae (TYGS 3180)
Intrasporangium calvum (TYGS 44)
Intrasporangium mesophilum (HQ204221)
Terrabacter tumescens (AF005023)
Terrabacter terrae (AY944176)
Terrabacter lapilli (AM690744)
Humibacillus xanthopallidus (AB282888)
Terracoccus luteus (Y11928)
Terrabacter aeriphilus (EU573947)
Terrabacter carboxydivorans (FJ717334)
Terrabacter ginsenosidimutans (EU332827)
Terrabacter aerolatus (EF212039)
Terrabacter terrigena (FJ423552)
Terrabacter koreensis (KF314196)
Tetrasphaera japonica (TYGS 2769)
Tetrasphaera remsis (TYGS 14367)
Tetrasphaera duodecadis (TYGS 2537)
Tetrasphaera jenkinsii (TYGS 2770)
Tetrasphaera veronensis (Y14595)
Tetrasphaera vanveenii (DQ007320)
Tetrasphaera australiensis (AF125091)
Tetrasphaera elongata (TYGS 2768)
Knoellia aerolata (TYGS 4814)
Janibacter melonis (AY522568)
Janibacter terrae (AF176948)
Janibacter brevis (TYGS 4494)
Janibacter limosus (Y08539)
Janibacter hoylei (FR749912)

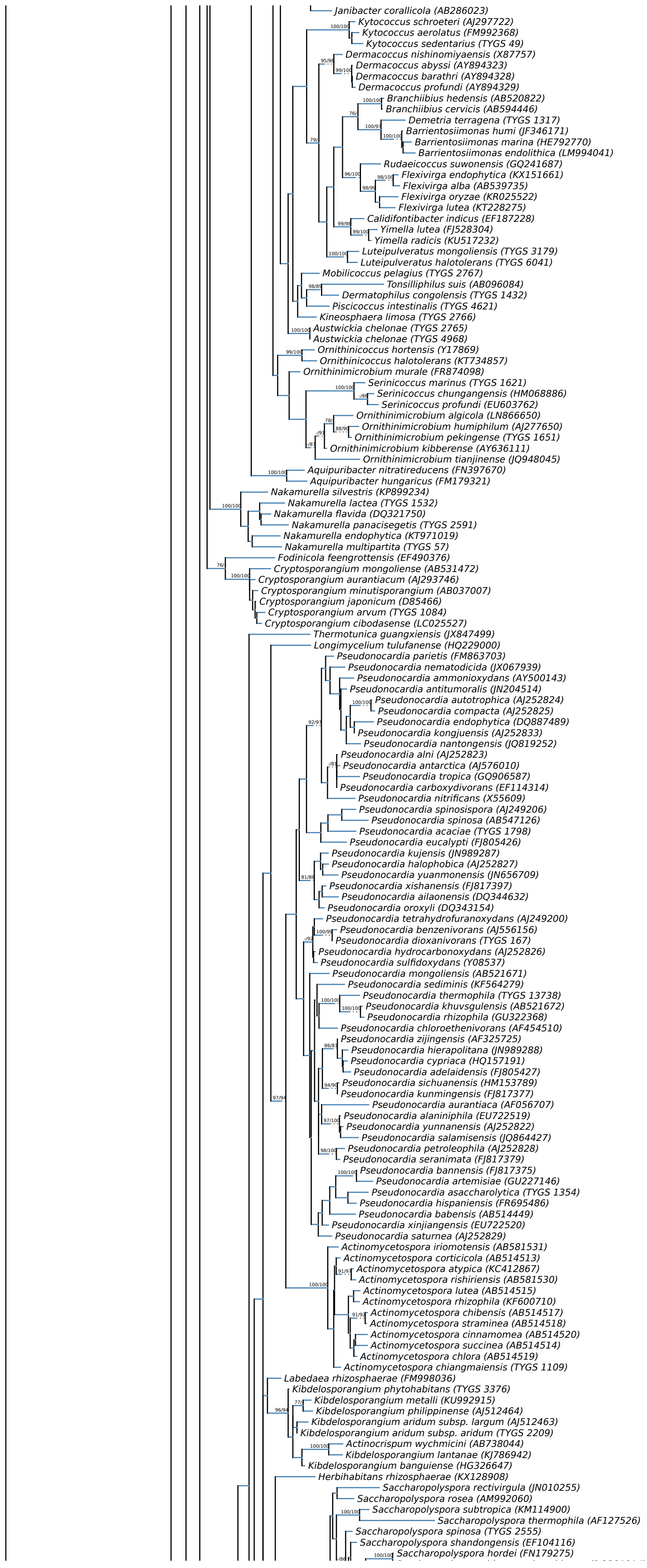

Janibacter corallicola (AB286023)
Kytococcus schroeteri (AJ297722)
Kytococcus aerolatus (FM992368)
Kytococcus sedentarius (TYGS 49)
Dermacoccus nishinomiyaensis (X87757)
Dermacoccus abyssi (AY894323)
Dermacoccus barathri (AY894328)
Dermacoccus profundi (AY894329)
Branchiibius hedensis (AB520822)
Branchiibius cervicis (AB594446)
Demetria terragena (TYGS 1317)
Barrientosiimonas humi (JF346171)
Barrientosiimonas marina (HE792770)
Barrientosiimonas endolithica (LM994041)
Rudaeicoccus suwonensis (GQ241687)
Flexivirga endophytica (KX151661)
Flexivirga alba (AB539735)
Flexivirga oryzae (KR025522)
Flexivirga lutea (KT228275)
Calidifontibacter indicus (EF187228)
Yimella lutea (FJ528304)
Yimella radicis (KU517232)
Luteipulveratus mongoliensis (TYGS 3179)
Luteipulveratus halotolerans (TYGS 6041)
Mobilicoccus pelagius (TYGS 2767)
Tonsilliphilus suis (AB096084)
Dermatophilus congolensis (TYGS 1432)
Piscicoccus intestinalis (TYGS 4621)
Kineosphaera limosa (TYGS 2766)
Austwickia chelonae (TYGS 2765)
Austwickia chelonae (TYGS 4968)
Ornithinicoccus hortensis (Y17869)
Ornithinicoccus halotolerans (KT734857)
Ornithinimicrobium murale (FR874098)
Serinicoccus marinus (TYGS 1621)
Serinicoccus chungangensis (HM068886)
Serinicoccus profundi (EU603762)
Ornithinimicrobium algicola (LN866650)
Ornithinimicrobium humiphilum (AJ277650)
Ornithinimicrobium pekingense (TYGS 1651)
Ornithinimicrobium kibberense (AY636111)
Ornithinimicrobium tianjinense (JQ948045)
Aquipuribacter nitratireducens (FN397670)
Aquipuribacter hungaricus (FM179321)
Nakamurella silvestris (KP899234)
Nakamurella lactea (TYGS 1532)
Nakamurella flavida (DQ321750)
Nakamurella panacisegetis (TYGS 2591)
Nakamurella endophytica (KT971019)
Nakamurella multipartita (TYGS 57)
Fodinicola feengrottensis (EF490376)
Cryptosporangium mongoliense (AB531472)
Cryptosporangium aurantiacum (AJ293746)
Cryptosporangium minutisporangium (AB037007)
Cryptosporangium japonicum (D85466)
Cryptosporangium arvum (TYGS 1084)
Cryptosporangium cibodasense (LC025527)
Thermotunica guangxiensis (JX847499)
Longimycelium tulufanense (HQ229000)
Pseudonocardia parietis (FM863703)
Pseudonocardia nematodicida (JX067939)
Pseudonocardia ammonioxydans (AY500143)
Pseudonocardia antitumoralis (JN204514)
Pseudonocardia autotrophica (AJ252824)
Pseudonocardia compacta (AJ252825)
Pseudonocardia endophytica (DQ887489)
Pseudonocardia kongjuensis (AJ252833)
Pseudonocardia nantongensis (JQ819252)
Pseudonocardia alni (AJ252823)
Pseudonocardia antarctica (AJ576010)
Pseudonocardia tropica (GQ906587)
Pseudonocardia carboxydivorans (EF114314)
Pseudonocardia nitrificans (X55609)
Pseudonocardia spinosispora (AJ249206)
Pseudonocardia spinosa (AB547126)
Pseudonocardia acaciae (TYGS 1798)
Pseudonocardia eucalypti (FJ805426)
Pseudonocardia kujensis (JN989287)
Pseudonocardia halophobica (AJ252827)
Pseudonocardia yuanmonensis (JN656709)
Pseudonocardia xishanensis (FJ817397)
Pseudonocardia ailaonensis (DQ344632)
Pseudonocardia oroxyli (DQ343154)
Pseudonocardia tetrahydrofuranoxydans (AJ249200)
Pseudonocardia benzenivorans (AJ556156)
Pseudonocardia dioxanivorans (TYGS 167)
Pseudonocardia hydrocarbonoxydans (AJ252826)
Pseudonocardia sulfidoxydans (Y08537)
Pseudonocardia mongoliensis (AB521671)
Pseudonocardia sediminis (KF564279)
Pseudonocardia thermophila (TYGS 13738)
Pseudonocardia khuvsgulensis (AB521672)
Pseudonocardia rhizophila (GU322368)
Pseudonocardia chloroethenivorans (AF454510)
Pseudonocardia zijingensis (AF325725)
Pseudonocardia hierapolitana (JN989288)
Pseudonocardia cypriaca (HQ157191)
Pseudonocardia adelaidensis (FJ805427)
Pseudonocardia sichuanensis (HM153789)
Pseudonocardia kunmingensis (FJ817377)
Pseudonocardia aurantiaca (AF056707)
Pseudonocardia alaniniphila (EU722519)
Pseudonocardia yunnanensis (AJ252822)
Pseudonocardia salamisensis (JQ864427)
Pseudonocardia petroleophila (AJ252828)
Pseudonocardia seranimata (FJ817379)
Pseudonocardia bannensis (FJ817375)
Pseudonocardia artemisiae (GU227146)
Pseudonocardia asaccharolytica (TYGS 1354)
Pseudonocardia hispaniensis (FR695486)
Pseudonocardia babensis (AB514449)
Pseudonocardia xinjiangensis (EU722520)
Pseudonocardia saturnea (AJ252829)
Actinomycetospora iriomotensis (AB581531)
Actinomycetospora corticicola (AB514513)
Actinomycetospora atypica (KC412867)
Actinomycetospora rishiriensis (AB581530)
Actinomycetospora lutea (AB514515)
Actinomycetospora rhizophila (KF600710)
Actinomycetospora chibensis (AB514517)
Actinomycetospora straminea (AB514518)
Actinomycetospora cinnamomea (AB514520)
Actinomycetospora succinea (AB514514)
Actinomycetospora chlora (AB514519)
Actinomycetospora chiangmaiensis (TYGS 1109)
Labedaea rhizosphaerae (FM998036)
Kibdelosporangium phytohabitans (TYGS 3376)
Kibdelosporangium metalli (KU992915)
Kibdelosporangium philippinense (AJ512464)
Kibdelosporangium aridum subsp. largum (AJ512463)
Kibdelosporangium aridum subsp. aridum (TYGS 2209)
Actinocrispum wychmicini (AB738044)
Kibdelosporangium lantanae (KJ786942)
Kibdelosporangium banguiense (HG326647)
Herbihabitans rhizosphaerae (KX128908)
Saccharopolyspora rectivirgula (JN010255)
Saccharopolyspora rosea (AM992060)
Saccharopolyspora subtropica (KM114900)
Saccharopolyspora thermophila (AF127526)
Saccharopolyspora spinosa (TYGS 2555)
Saccharopolyspora shandongensis (EF104116)
Saccharopolyspora hordei (FN179275)

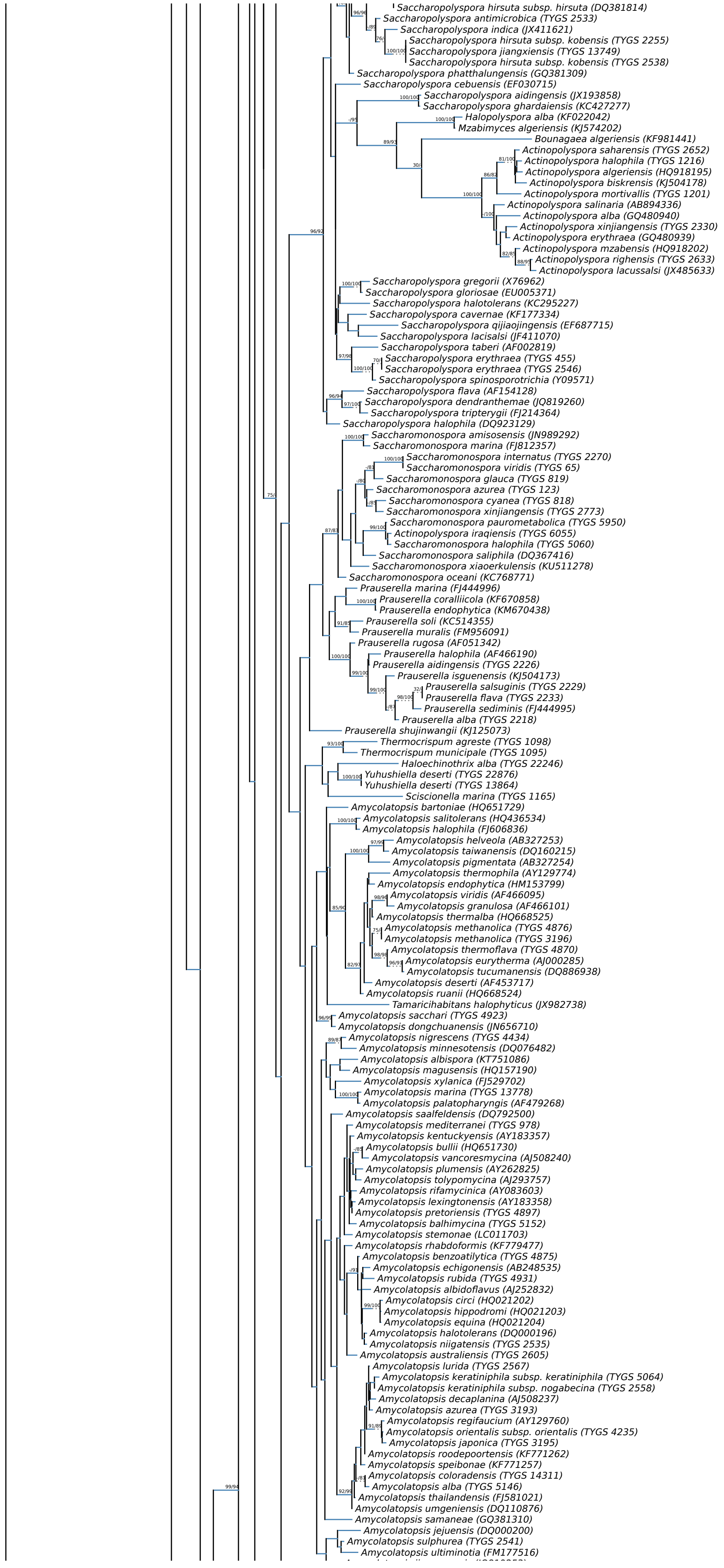
Saccharopolyspora hirsuta subsp. hirsuta (DQ381814)
Saccharopolyspora antimicrobica (TYGS 2533)
Saccharopolyspora indica (JX411621)
Saccharopolyspora hirsuta subsp. kobensis (TYGS 2255)
Saccharopolyspora jiangxiensis (TYGS 13749)
Saccharopolyspora hirsuta subsp. kobensis (TYGS 2538)
Saccharopolyspora phatthalungensis (GQ381309)
Saccharopolyspora cebuensis (EF030715)
Saccharopolyspora aidingensis (JX193858)
Saccharopolyspora ghardaiensis (KC427277)
Halopolyspora alba (KF022042)
Mzabimyces algeriensis (KJ574202)
Bounagaea algeriensis (KF981441)
Actinopolyspora saharensis (TYGS 2652)
Actinopolyspora halophila (TYGS 1216)
Actinopolyspora algeriensis (HQ918195)
Actinopolyspora biskrensis (KJ504178)
Actinopolyspora mortivallis (TYGS 1201)
Actinopolyspora salinaria (AB894336)
Actinopolyspora alba (GQ480940)
Actinopolyspora xinjiangensis (TYGS 2330)
Actinopolyspora erythraea (GQ480939)
Actinopolyspora mzabensis (HQ918202)
Actinopolyspora righensis (TYGS 2633)
Actinopolyspora lacussalsi (JX485633)
Saccharopolyspora gregorii (X76962)
Saccharopolyspora gloriosae (EU005371)
Saccharopolyspora halotolerans (KC295227)
Saccharopolyspora cavernae (KF177334)
Saccharopolyspora qijiaojingensis (EF687715)
Saccharopolyspora lacisalsi (JF411070)
Saccharopolyspora taberi (AF002819)
Saccharopolyspora erythraea (TYGS 455)
Saccharopolyspora erythraea (TYGS 2546)
Saccharopolyspora spinosporotrichia (Y09571)
Saccharopolyspora flava (AF154128)
Saccharopolyspora dendranthemae (JQ819260)
Saccharopolyspora tripterygii (FJ214364)
Saccharopolyspora halophila (DQ923129)
Saccharomonospora amisosensis (JN989292)
Saccharomonospora marina (FJ812357)
Saccharomonospora internatus (TYGS 2270)
Saccharomonospora viridis (TYGS 65)
Saccharomonospora glauca (TYGS 819)
Saccharomonospora azurea (TYGS 123)
Saccharomonospora cyanea (TYGS 818)
Saccharomonospora xinjiangensis (TYGS 2773)
Saccharomonospora paurometabolica (TYGS 5950)
Actinopolyspora iraqiensis (TYGS 6055)
Saccharomonospora halophila (TYGS 5060)
Saccharomonospora saliphila (DQ367416)
Saccharomonospora xiaoerkulensis (KU511278)
Saccharomonospora oceani (KC768771)
Prauserella marina (FJ444996)
Prauserella coralliicola (KF670858)
Prauserella endophytica (KM670438)
Prauserella soli (KC514355)
Prauserella muralis (FM956091)
Prauserella rugosa (AF051342)
Prauserella halophila (AF466190)
Prauserella aidingensis (TYGS 2226)
Prauserella isguenensis (KJ504173)
Prauserella salsuginis (TYGS 2229)
Prauserella flava (TYGS 2233)
Prauserella sediminis (FJ444995)
Prauserella alba (TYGS 2218)
Prauserella shujinwangii (KJ125073)
Thermocrispum agreste (TYGS 1098)
Thermocrispum municipale (TYGS 1095)
Haloechinothrix alba (TYGS 22246)
Yuhushiella deserti (TYGS 22876)
Yuhushiella deserti (TYGS 13864)
Sciscionella marina (TYGS 1165)
Amycolatopsis bartoniae (HQ651729)
Amycolatopsis salitolerans (HQ436534)
Amycolatopsis halophila (FJ606836)
Amycolatopsis helveola (AB327253)
Amycolatopsis taiwanensis (DQ160215)
Amycolatopsis pigmentata (AB327254)
Amycolatopsis thermophila (AY129774)
Amycolatopsis endophytica (HM153799)
Amycolatopsis viridis (AF466095)
Amycolatopsis granulosa (AF466101)
Amycolatopsis thermalba (HQ668525)
Amycolatopsis methanolica (TYGS 4876)
Amycolatopsis methanolica (TYGS 3196)
Amycolatopsis thermoflava (TYGS 4870)
Amycolatopsis eurytherma (AJ000285)
Amycolatopsis tucumanensis (DQ886938)
Amycolatopsis deserti (AF453717)
Amycolatopsis ruanii (HQ668524)
Tamaricihabitans halophyticus (JX982738)
Amycolatopsis sacchari (TYGS 4923)
Amycolatopsis dongchuanensis (JN656710)
Amycolatopsis nigrescens (TYGS 4434)
Amycolatopsis minnesotensis (DQ076482)
Amycolatopsis albispora (KT751086)
Amycolatopsis magusensis (HQ157190)
Amycolatopsis xylanica (FJ529702)
Amycolatopsis marina (TYGS 13778)
Amycolatopsis palatopharyngis (AF479268)
Amycolatopsis saalfeldensis (DQ792500)
Amycolatopsis mediterranei (TYGS 978)
Amycolatopsis kentuckyensis (AY183357)
Amycolatopsis bullii (HQ651730)
Amycolatopsis vancoresmycina (AJ508240)
Amycolatopsis plumensis (AY262825)
Amycolatopsis tolypomycina (AJ293757)
Amycolatopsis rifamycinica (AY083603)
Amycolatopsis lexingtonensis (AY183358)
Amycolatopsis pretoriensis (TYGS 4897)
Amycolatopsis balhimycina (TYGS 5152)
Amycolatopsis stemonae (LC011703)
Amycolatopsis rhabdoformis (KF779477)
Amycolatopsis benzoatilytica (TYGS 4875)
Amycolatopsis echigonensis (AB248535)
Amycolatopsis rubida (TYGS 4931)
Amycolatopsis albidoflavus (AJ252832)
Amycolatopsis circi (HQ021202)
Amycolatopsis hippodromi (HQ021203)
Amycolatopsis equina (HQ021204)
Amycolatopsis halotolerans (DQ000196)
Amycolatopsis niigatensis (TYGS 2535)
Amycolatopsis australiensis (TYGS 2605)
Amycolatopsis lurida (TYGS 2567)
Amycolatopsis keratiniphila subsp. keratiniphila (TYGS 5064)
Amycolatopsis keratiniphila subsp. nogabecina (TYGS 2558)
Amycolatopsis decaplanina (AJ508237)
Amycolatopsis azurea (TYGS 3193)
Amycolatopsis regifaucium (AY129760)
Amycolatopsis orientalis subsp. orientalis (TYGS 4235)
Amycolatopsis japonica (TYGS 3195)
Amycolatopsis roodepoortensis (KF771262)
Amycolatopsis speibonae (KF771257)
Amycolatopsis coloradensis (TYGS 14311)
Amycolatopsis alba (TYGS 5146)
Amycolatopsis thailandensis (FJ581021)
Amycolatopsis umgeniensis (DQ110876)
Amycolatopsis samaneae (GQ381310)
Amycolatopsis jejuensis (DQ000200)
Amycolatopsis sulphurea (TYGS 2541)
Amycolatopsis ultiminotia (FM177516)

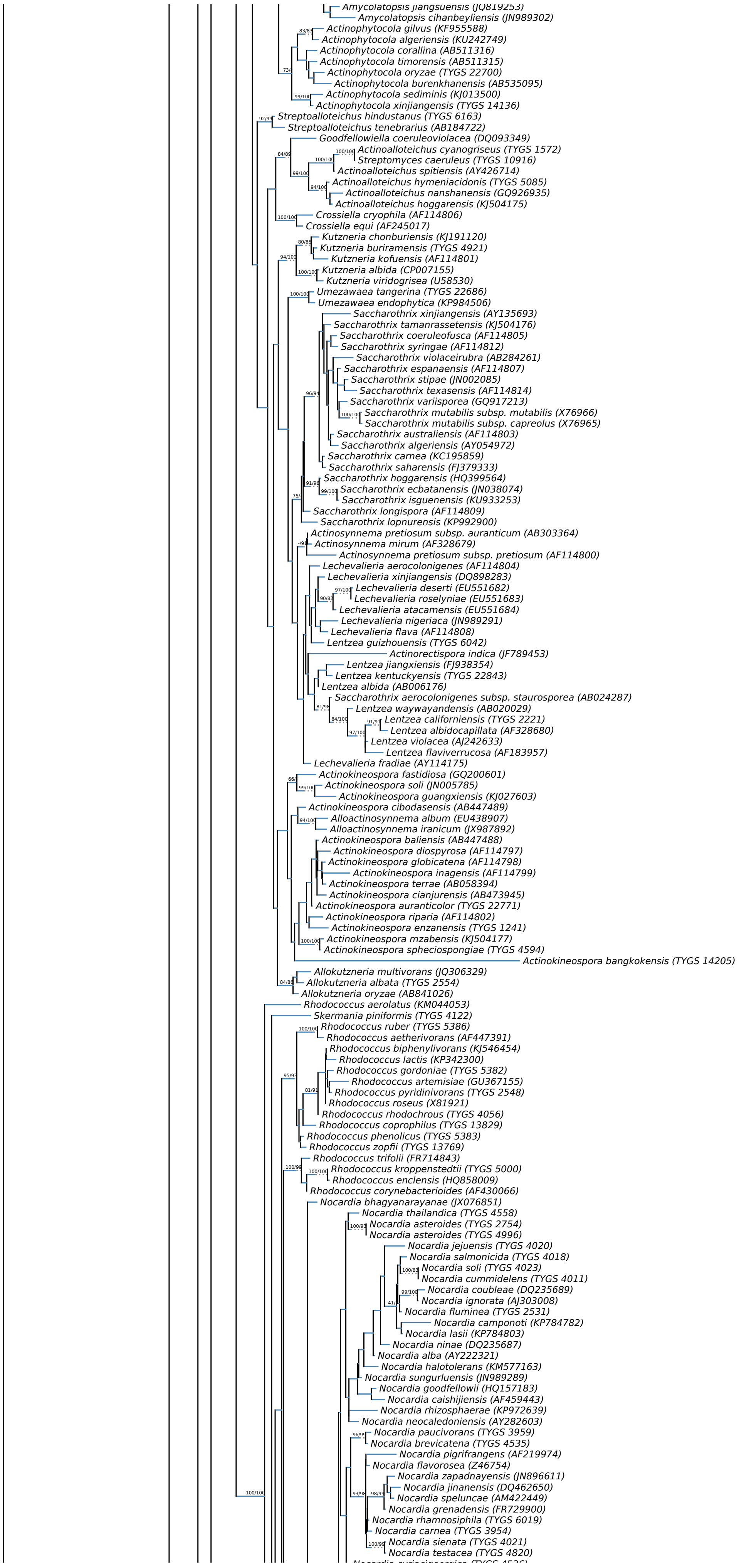
Amycolatopsis jiangsuensis (JQ819253)
Amycolatopsis cihanbeyliensis (JN989302)
Actinophytocola gilvus (KF955588)
Actinophytocola algeriensis (KU242749)
Actinophytocola corallina (AB511316)
Actinophytocola timorensis (AB511315)
Actinophytocola oryzae (TYGS 22700)
Actinophytocola burenkhanensis (AB535095)
Actinophytocola sediminis (KJ013500)
Actinophytocola xinjiangensis (TYGS 14136)
Streptoalloteichus hindustanus (TYGS 6163)
Streptoalloteichus tenebrarius (AB184722)
Goodfellowiella coeruleoviolacea (DQ093349)
Actinoalloteichus cyanogriseus (TYGS 1572)
Streptomyces caeruleus (TYGS 10916)
Actinoalloteichus spitiensis (AY426714)
Actinoalloteichus hymeniacidonis (TYGS 5085)
Actinoalloteichus nanshanensis (GQ926935)
Actinoalloteichus hoggarensis (KJ504175)
Crossiella cryophila (AF114806)
Crossiella equi (AF245017)
Kutzneria chonburiensis (KJ191120)
Kutzneria buriramensis (TYGS 4921)
Kutzneria kofuensis (AF114801)
Kutzneria albida (CP007155)
Kutzneria viridogrisea (U58530)
Umezawaea tangerina (TYGS 22686)
Umezawaea endophytica (KP984506)
Saccharothrix xinjiangensis (AY135693)
Saccharothrix tamanrassetensis (KJ504176)
Saccharothrix coeruleofusca (AF114805)
Saccharothrix syringae (AF114812)
Saccharothrix violaceirubra (AB284261)
Saccharothrix espanaensis (AF114807)
Saccharothrix stipae (JN002085)
Saccharothrix texasensis (AF114814)
Saccharothrix variisporea (GQ917213)
Saccharothrix mutabilis subsp. mutabilis (X76966)
Saccharothrix mutabilis subsp. capreolus (X76965)
Saccharothrix australiensis (AF114803)
Saccharothrix algeriensis (AY054972)
Saccharothrix carnea (KC195859)
Saccharothrix saharensis (FJ379333)
Saccharothrix hoggarensis (HQ399564)
Saccharothrix ecbatanensis (JN038074)
Saccharothrix isguenensis (KU933253)
Saccharothrix longispora (AF114809)
Saccharothrix lopnurensis (KP992900)
Actinosynnema pretiosum subsp. auranticum (AB303364)
Actinosynnema mirum (AF328679)
Actinosynnema pretiosum subsp. pretiosum (AF114800)
Lechevalieria aerocolonigenes (AF114804)
Lechevalieria xinjiangensis (DQ898283)
Lechevalieria deserti (EU551682)
Lechevalieria roselyniae (EU551683)
Lechevalieria atacamensis (EU551684)
Lechevalieria nigeriaca (JN989291)
Lechevalieria flava (AF114808)
Lentzea guizhouensis (TYGS 6042)
Actinorectispora indica (JF789453)
Lentzea jiangxiensis (FJ938354)
Lentzea kentuckyensis (TYGS 22843)
Lentzea albida (AB006176)
Saccharothrix aerocolonigenes subsp. staurosporea (AB024287)
Lentzea waywayandensis (AB020029)
Lentzea californiensis (TYGS 2221)
Lentzea albidocapillata (AF328680)
Lentzea violacea (AJ242633)
Lentzea flaviverrucosa (AF183957)
Lechevalieria fradiae (AY114175)
Actinokineospora fastidiosa (GQ200601)
Actinokineospora soli (JN005785)
Actinokineospora guangxiensis (KJ027603)
Actinokineospora cibodasensis (AB447489)
Alloactinosynnema album (EU438907)
Alloactinosynnema iranicum (JX987892)
Actinokineospora baliensis (AB447488)
Actinokineospora diospyrosa (AF114797)
Actinokineospora globicatena (AF114798)
Actinokineospora inagensis (AF114799)
Actinokineospora terrae (AB058394)
Actinokineospora cianjurensis (AB473945)
Actinokineospora auranticolor (TYGS 22771)
Actinokineospora riparia (AF114802)
Actinokineospora enzanensis (TYGS 1241)
Actinokineospora mzabensis (KJ504177)
Actinokineospora spheciospongiae (TYGS 4594)
Actinokineospora bangkokensis (TYGS 14205)
Allokutzneria multivorans (JQ306329)
Allokutzneria albata (TYGS 2554)
Allokutzneria oryzae (AB841026)
Rhodococcus aerolatus (KM044053)
Skermania piniformis (TYGS 4122)
Rhodococcus ruber (TYGS 5386)
Rhodococcus aetherivorans (AF447391)
Rhodococcus biphenylivorans (KJ546454)
Rhodococcus lactis (KP342300)
Rhodococcus gordoniae (TYGS 5382)
Rhodococcus artemisiae (GU367155)
Rhodococcus pyridinivorans (TYGS 2548)
Rhodococcus roseus (X81921)
Rhodococcus rhodochrous (TYGS 4056)
Rhodococcus coprophilus (TYGS 13829)
Rhodococcus phenolicus (TYGS 5383)
Rhodococcus zopfii (TYGS 13769)
Rhodococcus trifolii (FR714843)
Rhodococcus kroppenstedtii (TYGS 5000)
Rhodococcus enclensis (HQ858009)
Rhodococcus corynebacterioides (AF430066)
Nocardia bhagyanarayanae (JX076851)
Nocardia thailandica (TYGS 4558)
Nocardia asteroides (TYGS 2754)
Nocardia asteroides (TYGS 4996)
Nocardia jejuensis (TYGS 4020)
Nocardia salmonicida (TYGS 4018)
Nocardia soli (TYGS 4023)
Nocardia cummidelens (TYGS 4011)
Nocardia coubleae (DQ235689)
Nocardia ignorata (AJ303008)
Nocardia fluminea (TYGS 2531)
Nocardia camponoti (KP784782)
Nocardia lasii (KP784803)
Nocardia ninae (DQ235687)
Nocardia alba (AY222321)
Nocardia halotolerans (KM577163)
Nocardia sungurluensis (JN989289)
Nocardia goodfellowii (HQ157183)
Nocardia caishijiensis (AF459443)
Nocardia rhizosphaerae (KP972639)
Nocardia neocaledoniensis (AY282603)
Nocardia paucivorans (TYGS 3959)
Nocardia brevicatena (TYGS 4535)
Nocardia pigrifrangens (AF219974)
Nocardia flavorosea (Z46754)
Nocardia zapadnayensis (JN896611)
Nocardia jinanensis (DQ462650)
Nocardia speluncae (AM422449)
Nocardia grenadensis (FR729900)
Nocardia rhamnosiphila (TYGS 6019)
Nocardia carnea (TYGS 3954)
Nocardia sienata (TYGS 4021)
Nocardia testacea (TYGS 4820)

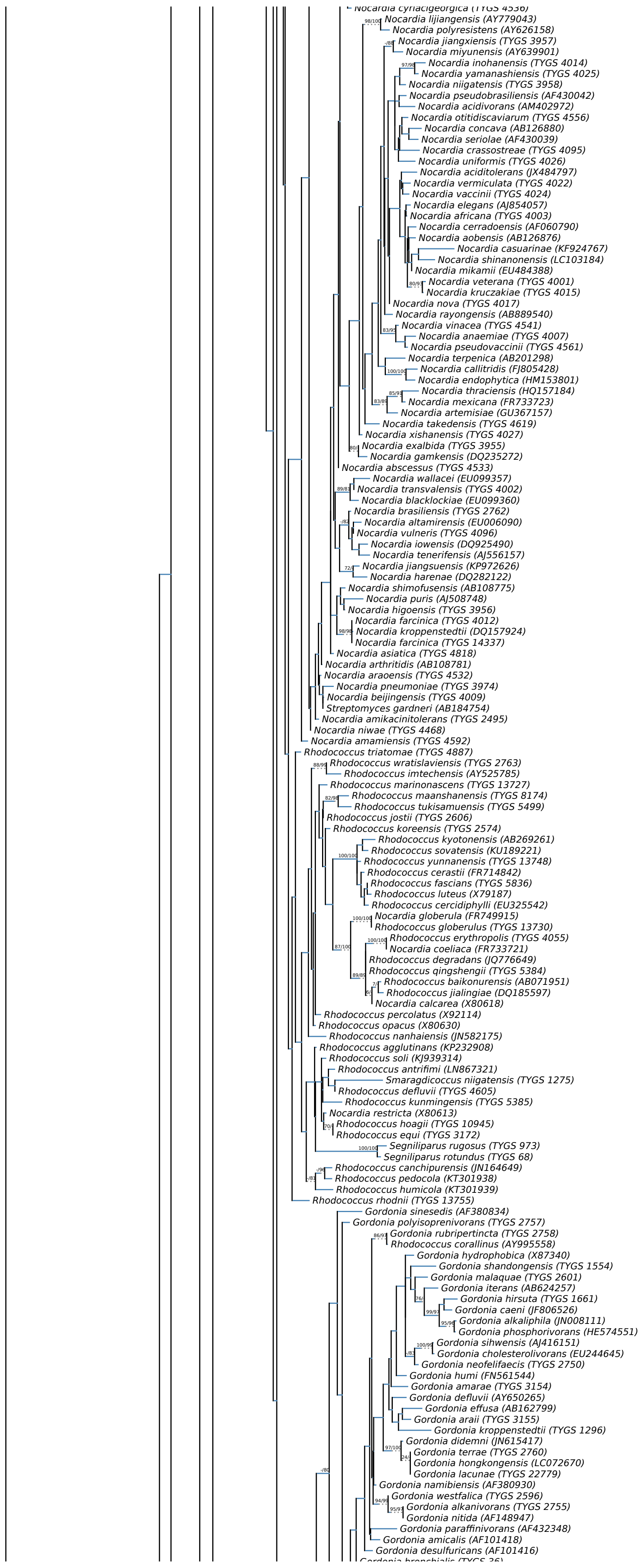
Nocardia cyriacigeorgica (TYGS 4536)
Nocardia lijiangensis (AY779043)
Nocardia polyresistens (AY626158)
Nocardia jiangxiensis (TYGS 3957)
Nocardia miyunensis (AY639901)
Nocardia inohanensis (TYGS 4014)
Nocardia yamanashiensis (TYGS 4025)
Nocardia niigatensis (TYGS 3958)
Nocardia pseudobrasiliensis (AF430042)
Nocardia acidivorans (AM402972)
Nocardia otitidiscaviarum (TYGS 4556)
Nocardia concava (AB126880)
Nocardia seriolae (AF430039)
Nocardia crassostreae (TYGS 4095)
Nocardia uniformis (TYGS 4026)
Nocardia aciditolerans (JX484797)
Nocardia vermiculata (TYGS 4022)
Nocardia vaccinii (TYGS 4024)
Nocardia elegans (AJ854057)
Nocardia africana (TYGS 4003)
Nocardia cerradoensis (AF060790)
Nocardia aobensis (AB126876)
Nocardia casuarinae (KF924767)
Nocardia shinanonensis (LC103184)
Nocardia mikamii (EU484388)
Nocardia veterana (TYGS 4001)
Nocardia kruczakiae (TYGS 4015)
Nocardia nova (TYGS 4017)
Nocardia rayongensis (AB889540)
Nocardia vinacea (TYGS 4541)
Nocardia anaemiae (TYGS 4007)
Nocardia pseudovaccinii (TYGS 4561)
Nocardia terpenica (AB201298)
Nocardia callitridis (FJ805428)
Nocardia endophytica (HM153801)
Nocardia thraciensis (HQ157184)
Nocardia mexicana (FR733723)
Nocardia artemisiae (GU367157)
Nocardia takedensis (TYGS 4619)
Nocardia xishanensis (TYGS 4027)
Nocardia exalbida (TYGS 3955)
Nocardia gamkensis (DQ235272)
Nocardia abscessus (TYGS 4533)
Nocardia wallacei (EU099357)
Nocardia transvalensis (TYGS 4002)
Nocardia blacklockiae (EU099360)
Nocardia brasiliensis (TYGS 2762)
Nocardia altamirensis (EU006090)
Nocardia vulneris (TYGS 4096)
Nocardia iowensis (DQ925490)
Nocardia tenerifensis (AJ556157)
Nocardia jiangsuensis (KP972626)
Nocardia harenae (DQ282122)
Nocardia shimofusensis (AB108775)
Nocardia puris (AJ508748)
Nocardia higoensis (TYGS 3956)
Nocardia farcinica (TYGS 4012)
Nocardia kroppenstedtii (DQ157924)
Nocardia farcinica (TYGS 14337)
Nocardia asiatica (TYGS 4818)
Nocardia arthritidis (AB108781)
Nocardia araoensis (TYGS 4532)
Nocardia pneumoniae (TYGS 3974)
Nocardia beijingensis (TYGS 4009)
Streptomyces gardneri (AB184754)
Nocardia amikacinitolerans (TYGS 2495)
Nocardia niwae (TYGS 4468)
Nocardia amamiensis (TYGS 4592)
Rhodococcus triatomae (TYGS 4887)
Rhodococcus wratislaviensis (TYGS 2763)
Rhodococcus imtechensis (AY525785)
Rhodococcus marinonascens (TYGS 13727)
Rhodococcus maanshanensis (TYGS 8174)
Rhodococcus tukisamuensis (TYGS 5499)
Rhodococcus jostii (TYGS 2606)
Rhodococcus koreensis (TYGS 2574)
Rhodococcus kyotonensis (AB269261)
Rhodococcus sovatensis (KU189221)
Rhodococcus yunnanensis (TYGS 13748)
Rhodococcus cerastii (FR714842)
Rhodococcus fascians (TYGS 5836)
Rhodococcus luteus (X79187)
Rhodococcus cercidiphylli (EU325542)
Nocardia globerula (FR749915)
Rhodococcus globerulus (TYGS 13730)
Rhodococcus erythropolis (TYGS 4055)
Nocardia coeliaca (FR733721)
Rhodococcus degradans (JQ776649)
Rhodococcus qingshengii (TYGS 5384)
Rhodococcus baikonurensis (AB071951)
Rhodococcus jialingiae (DQ185597)
Nocardia calcarea (X80618)
Rhodococcus percolatus (X92114)
Rhodococcus opacus (X80630)
Rhodococcus nanhaiensis (JN582175)
Rhodococcus agglutinans (KP232908)
Rhodococcus soli (KJ939314)
Rhodococcus antrifimi (LN867321)
Smaragdicoccus niigatensis (TYGS 1275)
Rhodococcus defluvii (TYGS 4605)
Rhodococcus kunmingensis (TYGS 5385)
Nocardia restricta (X80613)
Rhodococcus hoagii (TYGS 10945)
Rhodococcus equi (TYGS 3172)
Segniliparus rugosus (TYGS 973)
Segniliparus rotundus (TYGS 68)
Rhodococcus canchipurensis (JN164649)
Rhodococcus pedocola (KT301938)
Rhodococcus humicola (KT301939)
Rhodococcus rhodnii (TYGS 13755)
Gordonia sinesedis (AF380834)
Gordonia polyisoprenivorans (TYGS 2757)
Gordonia rubripertincta (TYGS 2758)
Rhodococcus corallinus (AY995558)
Gordonia hydrophobica (X87340)
Gordonia shandongensis (TYGS 1554)
Gordonia malaquae (TYGS 2601)
Gordonia iterans (AB624257)
Gordonia hirsuta (TYGS 1661)
Gordonia caeni (JF806526)
Gordonia alkaliphila (JN008111)
Gordonia phosphorivorans (HE574551)
Gordonia sihwensis (AJ416151)
Gordonia cholesterolivorans (EU244645)
Gordonia neofelifaecis (TYGS 2750)
Gordonia humi (FN561544)
Gordonia amarae (TYGS 3154)
Gordonia defluvii (AY650265)
Gordonia effusa (AB162799)
Gordonia araii (TYGS 3155)
Gordonia kroppenstedtii (TYGS 1296)
Gordonia didemni (JN615417)
Gordonia terrae (TYGS 2760)
Gordonia hongkongensis (LC072670)
Gordonia lacunae (TYGS 22779)
Gordonia namibiensis (AF380930)
Gordonia westfalica (TYGS 2596)
Gordonia alkanivorans (TYGS 2755)
Gordonia nitida (AF148947)
Gordonia paraffinivorans (AF432348)
Gordonia amicalis (AF101418)
Gordonia desulfuricans (AF101416)
Gordonia bronchialis (TYGS 36)

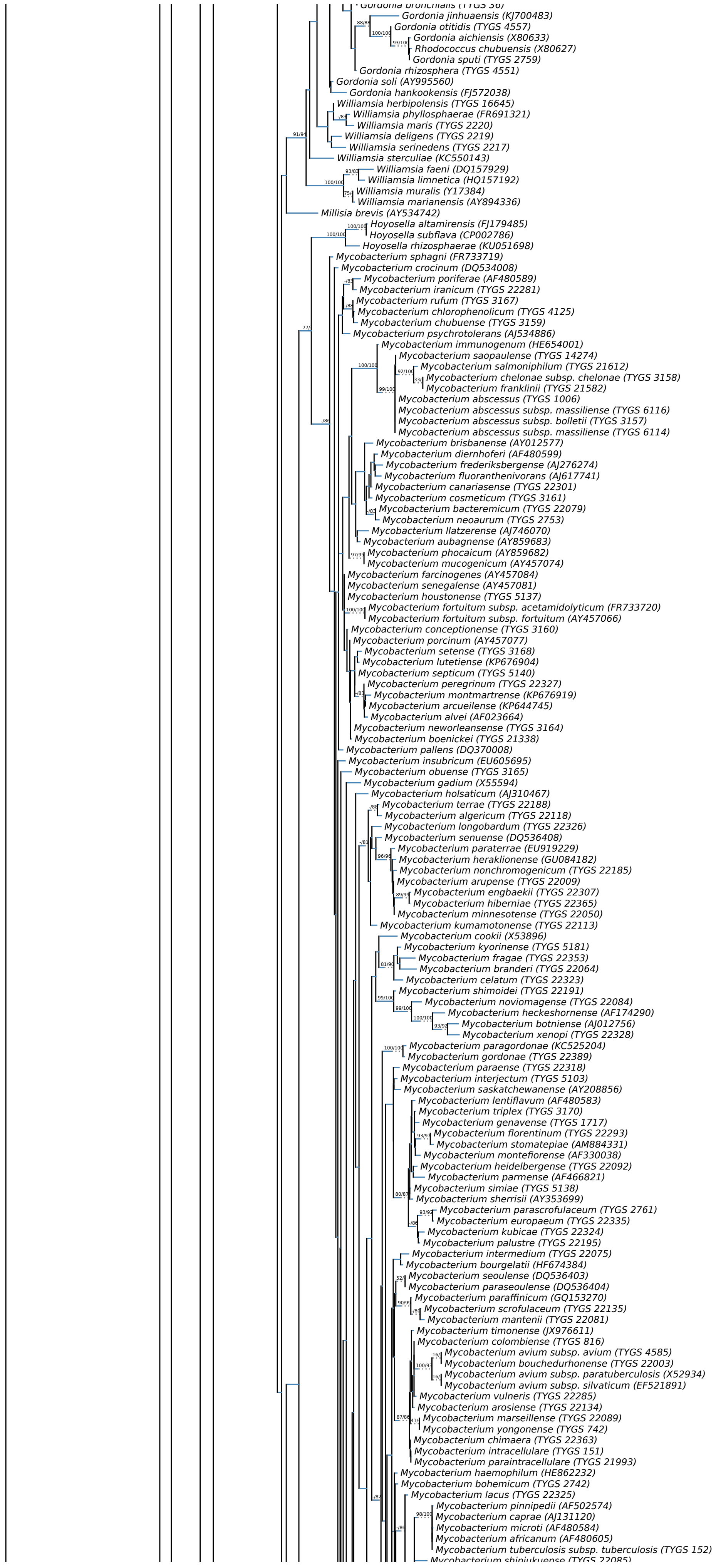
Gordonia jinhuaensis (KJ700483)
Gordonia otitidis (TYGS 4557)
Gordonia aichiensis (X80633)
Rhodococcus chubuensis (X80627)
Gordonia sputi (TYGS 2759)
Gordonia rhizosphera (TYGS 4551)
Gordonia soli (AY995560)
Gordonia hankookensis (FJ572038)
Williamsia herbipolensis (TYGS 16645)
Williamsia phyllosphaerae (FR691321)
Williamsia maris (TYGS 2220)
Williamsia deligens (TYGS 2219)
Williamsia serinedens (TYGS 2217)
Williamsia sterculiae (KC550143)
Williamsia faeni (DQ157929)
Williamsia limnetica (HQ157192)
Williamsia muralis (Y17384)
Williamsia marianensis (AY894336)
Millisia brevis (AY534742)
Hoyosella altamirensis (FJ179485)
Hoyosella subflava (CP002786)
Hoyosella rhizosphaerae (KU051698)
Mycobacterium sphagni (FR733719)
Mycobacterium crocinum (DQ534008)
Mycobacterium poriferae (AF480589)
Mycobacterium iranicum (TYGS 22281)
Mycobacterium rufum (TYGS 3167)
Mycobacterium chlorophenolicum (TYGS 4125)
Mycobacterium chubuense (TYGS 3159)
Mycobacterium psychrotolerans (AJ534886)
Mycobacterium immunogenum (HE654001)
Mycobacterium saopaulense (TYGS 14274)
Mycobacterium salmoniphilum (TYGS 21612)
Mycobacterium chelonae subsp. chelonae (TYGS 3158)
Mycobacterium franklinii (TYGS 21582)
Mycobacterium abscessus (TYGS 1006)
Mycobacterium abscessus subsp. massiliense (TYGS 6116)
Mycobacterium abscessus subsp. bolletii (TYGS 3157)
Mycobacterium abscessus subsp. massiliense (TYGS 6114)
Mycobacterium brisbanense (AY012577)
Mycobacterium diernhoferi (AF480599)
Mycobacterium frederiksbergense (AJ276274)
Mycobacterium fluoranthenivorans (AJ617741)
Mycobacterium canariasense (TYGS 22301)
Mycobacterium cosmeticum (TYGS 3161)
Mycobacterium bacteremicum (TYGS 22079)
Mycobacterium neoaurum (TYGS 2753)
Mycobacterium llatzerense (AJ746070)
Mycobacterium aubagnense (AY859683)
Mycobacterium phocaicum (AY859682)
Mycobacterium mucogenicum (AY457074)
Mycobacterium farcinogenes (AY457084)
Mycobacterium senegalense (AY457081)
Mycobacterium houstonense (TYGS 5137)
Mycobacterium fortuitum subsp. acetamidolyticum (FR733720)
Mycobacterium fortuitum subsp. fortuitum (AY457066)
Mycobacterium conceptionense (TYGS 3160)
Mycobacterium porcinum (AY457077)
Mycobacterium setense (TYGS 3168)
Mycobacterium lutetiense (KP676904)
Mycobacterium septicum (TYGS 5140)
Mycobacterium peregrinum (TYGS 22327)
Mycobacterium montmartrense (KP676919)
Mycobacterium arcueilense (KP644745)
Mycobacterium alvei (AF023664)
Mycobacterium neworleansense (TYGS 3164)
Mycobacterium boenickei (TYGS 21338)
Mycobacterium pallens (DQ370008)
Mycobacterium insubricum (EU605695)
Mycobacterium obuense (TYGS 3165)
Mycobacterium gadium (X55594)
Mycobacterium holsaticum (AJ310467)
Mycobacterium terrae (TYGS 22188)
Mycobacterium algericum (TYGS 22118)
Mycobacterium longobardum (TYGS 22326)
Mycobacterium senuense (DQ536408)
Mycobacterium paraterrae (EU919229)
Mycobacterium heraklionense (GU084182)
Mycobacterium nonchromogenicum (TYGS 22185)
Mycobacterium arupense (TYGS 22009)
Mycobacterium engbaekii (TYGS 22307)
Mycobacterium hiberniae (TYGS 22365)
Mycobacterium minnesotense (TYGS 22050)
Mycobacterium kumamotonense (TYGS 22113)
Mycobacterium cookii (X53896)
Mycobacterium kyorinense (TYGS 5181)
Mycobacterium fragae (TYGS 22353)
Mycobacterium branderi (TYGS 22064)
Mycobacterium celatum (TYGS 22323)
Mycobacterium shimoidei (TYGS 22191)
Mycobacterium noviomagense (TYGS 22084)
Mycobacterium heckeshornense (AF174290)
Mycobacterium botniense (AJ012756)
Mycobacterium xenopi (TYGS 22328)
Mycobacterium paragordonae (KC525204)
Mycobacterium gordonae (TYGS 22389)
Mycobacterium paraense (TYGS 22318)
Mycobacterium interjectum (TYGS 5103)
Mycobacterium saskatchewanense (AY208856)
Mycobacterium lentiflavum (AF480583)
Mycobacterium triplex (TYGS 3170)
Mycobacterium genavense (TYGS 1717)
Mycobacterium florentinum (TYGS 22293)
Mycobacterium stomatepiae (AM884331)
Mycobacterium montefiorense (AF330038)
Mycobacterium heidelbergense (TYGS 22092)
Mycobacterium parmense (AF466821)
Mycobacterium simiae (TYGS 5138)
Mycobacterium sherrisii (AY353699)
Mycobacterium parascrofulaceum (TYGS 2761)
Mycobacterium europaeum (TYGS 22335)
Mycobacterium kubicae (TYGS 22324)
Mycobacterium palustre (TYGS 22195)
Mycobacterium intermedium (TYGS 22075)
Mycobacterium bourgelatii (HF674384)
Mycobacterium seoulense (DQ536403)
Mycobacterium paraseoulense (DQ536404)
Mycobacterium paraffinicum (GQ153270)
Mycobacterium scrofulaceum (TYGS 22135)
Mycobacterium mantenii (TYGS 22081)
Mycobacterium timonense (JX976611)
Mycobacterium colombiense (TYGS 816)
Mycobacterium avium subsp. avium (TYGS 4585)
Mycobacterium bouchedurhonense (TYGS 22003)
Mycobacterium avium subsp. paratuberculosis (X52934)
Mycobacterium avium subsp. silvaticum (EF521891)
Mycobacterium vulneris (TYGS 22285)
Mycobacterium arosiense (TYGS 22134)
Mycobacterium marseillense (TYGS 22089)
Mycobacterium yongonense (TYGS 742)
Mycobacterium chimaera (TYGS 22363)
Mycobacterium intracellulare (TYGS 151)
Mycobacterium paraintracellulare (TYGS 21993)
Mycobacterium haemophilum (HE862232)
Mycobacterium bohemicum (TYGS 2742)
Mycobacterium lacus (TYGS 22325)
Mycobacterium pinnipedii (AF502574)
Mycobacterium caprae (AJ131120)
Mycobacterium microti (AF480584)
Mycobacterium africanum (AF480605)
Mycobacterium tuberculosis subsp. tuberculosis (TYGS 152)

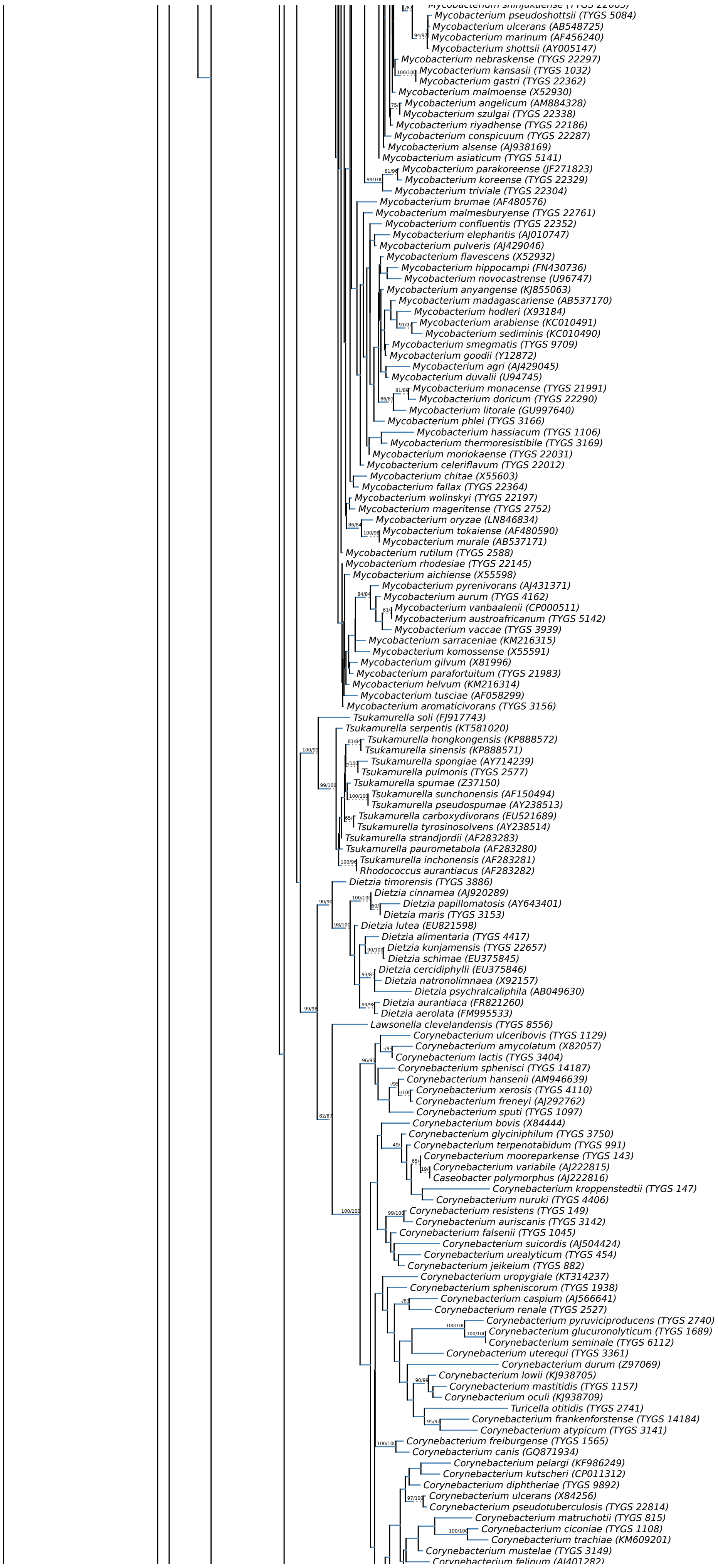
Mycobacterium pseudoshottsii (TYGS 5084)
Mycobacterium ulcerans (AB548725)
Mycobacterium marinum (AF456240)
Mycobacterium shottsii (AY005147)
Mycobacterium nebraskense (TYGS 22297)
Mycobacterium kansasii (TYGS 1032)
Mycobacterium gastri (TYGS 22362)
Mycobacterium malmoense (X52930)
Mycobacterium angelicum (AM884328)
Mycobacterium szulgai (TYGS 22338)
Mycobacterium riyadhense (TYGS 22186)
Mycobacterium conspicuum (TYGS 22287)
Mycobacterium alsense (AJ938169)
Mycobacterium asiaticum (TYGS 5141)
Mycobacterium parakoreense (JF271823)
Mycobacterium koreense (TYGS 22329)
Mycobacterium triviale (TYGS 22304)
Mycobacterium brumae (AF480576)
Mycobacterium malmesburyense (TYGS 22761)
Mycobacterium confluentis (TYGS 22352)
Mycobacterium elephantis (AJ010747)
Mycobacterium pulveris (AJ429046)
Mycobacterium flavescens (X52932)
Mycobacterium hippocampi (FN430736)
Mycobacterium novocastrense (U96747)
Mycobacterium anyangense (KJ855063)
Mycobacterium madagascariense (AB537170)
Mycobacterium hodleri (X93184)
Mycobacterium arabiense (KC010491)
Mycobacterium sediminis (KC010490)
Mycobacterium smegmatis (TYGS 9709)
Mycobacterium goodii (Y12872)
Mycobacterium agri (AJ429045)
Mycobacterium duvalii (U94745)
Mycobacterium monacense (TYGS 21991)
Mycobacterium doricum (TYGS 22290)
Mycobacterium litorale (GU997640)
Mycobacterium phlei (TYGS 3166)
Mycobacterium hassiacum (TYGS 1106)
Mycobacterium thermoresistibile (TYGS 3169)
Mycobacterium moriokaense (TYGS 22031)
Mycobacterium celeriflavum (TYGS 22012)
Mycobacterium chitae (X55603)
Mycobacterium fallax (TYGS 22364)
Mycobacterium wolinskyi (TYGS 22197)
Mycobacterium mageritense (TYGS 2752)
Mycobacterium oryzae (LN846834)
Mycobacterium tokaiense (AF480590)
Mycobacterium murale (AB537171)
Mycobacterium rutilum (TYGS 2588)
Mycobacterium rhodesiae (TYGS 22145)
Mycobacterium aichiense (X55598)
Mycobacterium pyrenivorans (AJ431371)
Mycobacterium aurum (TYGS 4162)
Mycobacterium vanbaalenii (CP000511)
Mycobacterium austroafricanum (TYGS 5142)
Mycobacterium vaccae (TYGS 3939)
Mycobacterium sarraceniae (KM216315)
Mycobacterium komossense (X55591)
Mycobacterium gilvum (X81996)
Mycobacterium parafortuitum (TYGS 21983)
Mycobacterium helvum (KM216314)
Mycobacterium tusciae (AF058299)
Mycobacterium aromaticivorans (TYGS 3156)
Tsukamurella soli (FJ917743)
Tsukamurella serpentis (KT581020)
Tsukamurella hongkongensis (KP888572)
Tsukamurella sinensis (KP888571)
Tsukamurella spongiae (AY714239)
Tsukamurella pulmonis (TYGS 2577)
Tsukamurella spumae (Z37150)
Tsukamurella sunchonensis (AF150494)
Tsukamurella pseudospumae (AY238513)
Tsukamurella carboxydivorans (EU521689)
Tsukamurella tyrosinosolvens (AY238514)
Tsukamurella strandjordii (AF283283)
Tsukamurella paurometabola (AF283280)
Tsukamurella inchonensis (AF283281)
Rhodococcus aurantiacus (AF283282)
Dietzia timorensis (TYGS 3886)
Dietzia cinnamea (AJ920289)
Dietzia papillomatosis (AY643401)
Dietzia maris (TYGS 3153)
Dietzia lutea (EU821598)
Dietzia alimentaria (TYGS 4417)
Dietzia kunjamensis (TYGS 22657)
Dietzia schimae (EU375845)
Dietzia cercidiphylli (EU375846)
Dietzia natronolimnaea (X92157)
Dietzia psychralcaliphila (AB049630)
Dietzia aurantiaca (FR821260)
Dietzia aerolata (FM995533)
Lawsonella clevelandensis (TYGS 8556)
Corynebacterium ulceribovis (TYGS 1129)
Corynebacterium amycolatum (X82057)
Corynebacterium lactis (TYGS 3404)
Corynebacterium sphenisci (TYGS 14187)
Corynebacterium hansenii (AM946639)
Corynebacterium xerosis (TYGS 4110)
Corynebacterium freneyi (AJ292762)
Corynebacterium sputi (TYGS 1097)
Corynebacterium bovis (X84444)
Corynebacterium glyciniphilum (TYGS 3750)
Corynebacterium terpenotabidum (TYGS 991)
Corynebacterium mooreparkense (TYGS 143)
Corynebacterium variabile (AJ222815)
Caseobacter polymorphus (AJ222816)
Corynebacterium kroppenstedtii (TYGS 147)
Corynebacterium nuruki (TYGS 4406)
Corynebacterium resistens (TYGS 149)
Corynebacterium auriscanis (TYGS 3142)
Corynebacterium falsenii (TYGS 1045)
Corynebacterium suicordis (AJ504424)
Corynebacterium urealyticum (TYGS 454)
Corynebacterium jeikeium (TYGS 882)
Corynebacterium uropygiale (KT314237)
Corynebacterium spheniscorum (TYGS 1938)
Corynebacterium caspium (AJ566641)
Corynebacterium renale (TYGS 2527)
Corynebacterium pyruviciproducens (TYGS 2740)
Corynebacterium glucuronolyticum (TYGS 1689)
Corynebacterium seminale (TYGS 6112)
Corynebacterium uterequi (TYGS 3361)
Corynebacterium durum (Z97069)
Corynebacterium lowii (KJ938705)
Corynebacterium mastitidis (TYGS 1157)
Corynebacterium oculi (KJ938709)
Turicella otitidis (TYGS 2741)
Corynebacterium frankenforstense (TYGS 14184)
Corynebacterium atypicum (TYGS 3141)
Corynebacterium freiburgense (TYGS 1565)
Corynebacterium canis (GQ871934)
Corynebacterium pelargi (KF986249)
Corynebacterium kutscheri (CP011312)
Corynebacterium diphtheriae (TYGS 9892)
Corynebacterium ulcerans (X84256)
Corynebacterium pseudotuberculosis (TYGS 22814)
Corynebacterium matruchotii (TYGS 815)
Corynebacterium ciconiae (TYGS 1108)
Corynebacterium trachiae (KM609201)
Corynebacterium mustelae (TYGS 3149)
Corynebacterium felinum (AJ401282)

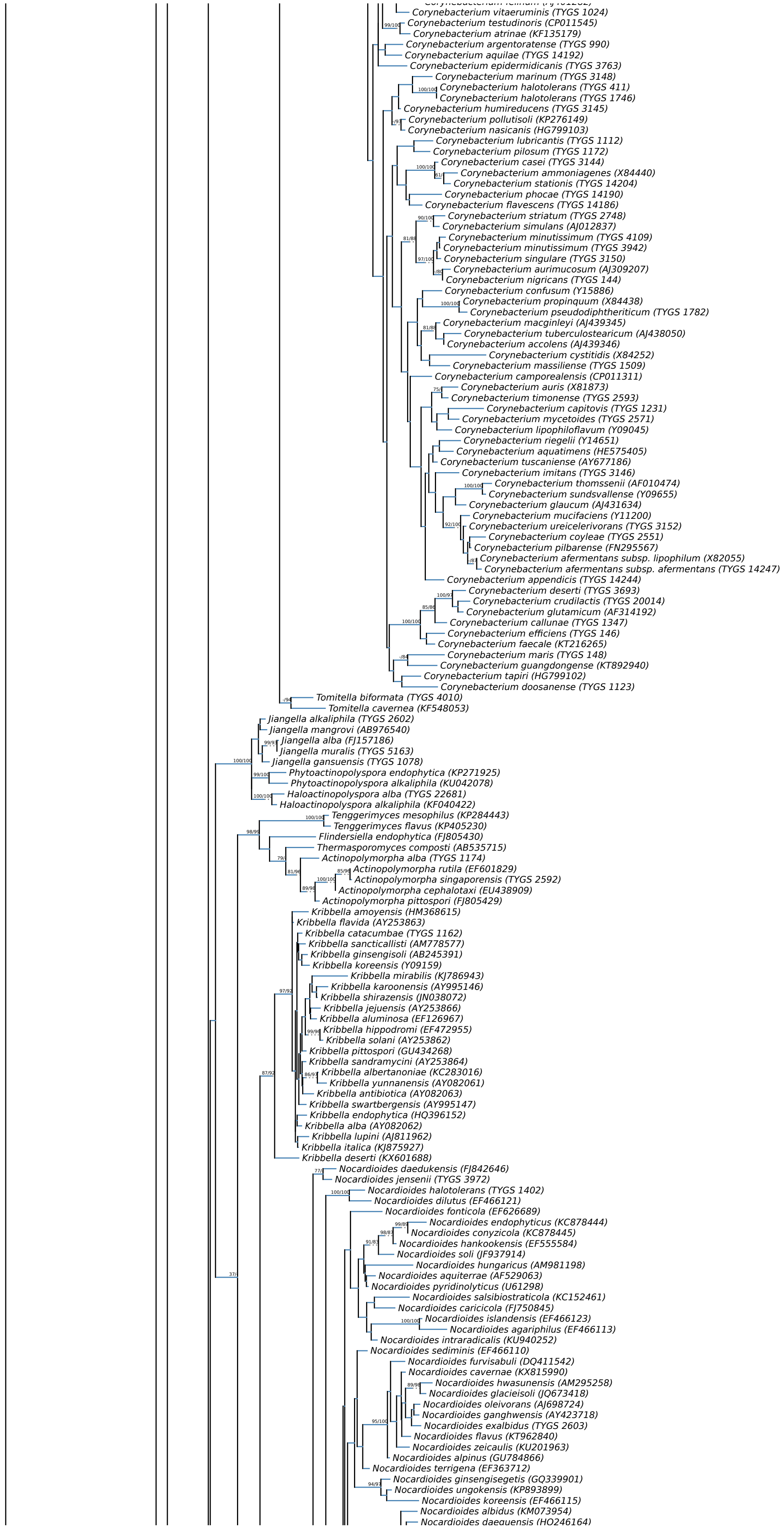

Corynebacterium vitaeruminis (TYGS 1024)
Corynebacterium testudinoris (CP011545)
Corynebacterium atrinae (KF135179)
Corynebacterium argentoratense (TYGS 990)
Corynebacterium aquilae (TYGS 14192)
Corynebacterium epidermidicanis (TYGS 3763)
Corynebacterium marinum (TYGS 3148)
Corynebacterium halotolerans (TYGS 411)
Corynebacterium halotolerans (TYGS 1746)
Corynebacterium humireducens (TYGS 3145)
Corynebacterium pollutisoli (KP276149)
Corynebacterium nasicanis (HG799103)
Corynebacterium lubricantis (TYGS 1112)
Corynebacterium pilosum (TYGS 1172)
Corynebacterium casei (TYGS 3144)
Corynebacterium ammoniagenes (X84440)
Corynebacterium stationis (TYGS 14204)
Corynebacterium phocae (TYGS 14190)
Corynebacterium flavescens (TYGS 14186)
Corynebacterium striatum (TYGS 2748)
Corynebacterium simulans (AJ012837)
Corynebacterium minutissimum (TYGS 4109)
Corynebacterium minutissimum (TYGS 3942)
Corynebacterium singulare (TYGS 3150)
Corynebacterium aurimucosum (AJ309207)
Corynebacterium nigricans (TYGS 144)
Corynebacterium confusum (Y15886)
Corynebacterium propinquum (X84438)
Corynebacterium pseudodiphtheriticum (TYGS 1782)
Corynebacterium macginleyi (AJ439345)
Corynebacterium tuberculostearicum (AJ438050)
Corynebacterium accolens (AJ439346)
Corynebacterium cystitidis (X84252)
Corynebacterium massiliense (TYGS 1509)
Corynebacterium camporealensis (CP011311)
Corynebacterium auris (X81873)
Corynebacterium timonense (TYGS 2593)
Corynebacterium capitovis (TYGS 1231)
Corynebacterium mycetoides (TYGS 2571)
Corynebacterium lipophiloflavum (Y09045)
Corynebacterium riegelii (Y14651)
Corynebacterium aquatimens (HE575405)
Corynebacterium tuscaniense (AY677186)
Corynebacterium imitans (TYGS 3146)
Corynebacterium thomssenii (AF010474)
Corynebacterium sundsvallense (Y09655)
Corynebacterium glaucum (AJ431634)
Corynebacterium mucifaciens (Y11200)
Corynebacterium ureicelerivorans (TYGS 3152)
Corynebacterium coyleae (TYGS 2551)
Corynebacterium pilbarense (FN295567)
Corynebacterium afermentans subsp. lipophilum (X82055)
Corynebacterium afermentans subsp. afermentans (TYGS 14247)
Corynebacterium appendicis (TYGS 14244)
Corynebacterium deserti (TYGS 3693)
Corynebacterium crudilactis (TYGS 20014)
Corynebacterium glutamicum (AF314192)
Corynebacterium callunae (TYGS 1347)
Corynebacterium efficiens (TYGS 146)
Corynebacterium faecale (KT216265)
Corynebacterium maris (TYGS 148)
Corynebacterium guangdongense (KT892940)
Corynebacterium tapiri (HG799102)
Corynebacterium doosanense (TYGS 1123)
Tomitella biformata (TYGS 4010)
Tomitella cavernea (KF548053)
Jiangella alkaliphila (TYGS 2602)
Jiangella mangrovi (AB976540)
Jiangella alba (FJ157186)
Jiangella muralis (TYGS 5163)
Jiangella gansuensis (TYGS 1078)
Phytoactinopolyspora endophytica (KP271925)
Phytoactinopolyspora alkaliphila (KU042078)
Haloactinopolyspora alba (TYGS 22681)
Haloactinopolyspora alkaliphila (KF040422)
Tenggerimyces mesophilus (KP284443)
Tenggerimyces flavus (KP405230)
Flindersiella endophytica (FJ805430)
Thermasporomyces composti (AB535715)
Actinopolymorpha alba (TYGS 1174)
Actinopolymorpha rutila (EF601829)
Actinopolymorpha singaporensis (TYGS 2592)
Actinopolymorpha cephalotaxi (EU438909)
Actinopolymorpha pittospori (FJ805429)
Kribbella amoyensis (HM368615)
Kribbella flavida (AY253863)
Kribbella catacumbae (TYGS 1162)
Kribbella sancticallisti (AM778577)
Kribbella ginsengisoli (AB245391)
Kribbella koreensis (Y09159)
Kribbella mirabilis (KJ786943)
Kribbella karoonensis (AY995146)
Kribbella shirazensis (JN038072)
Kribbella jejuensis (AY253866)
Kribbella aluminosa (EF126967)
Kribbella hippodromi (EF472955)
Kribbella solani (AY253862)
Kribbella pittospori (GU434268)
Kribbella sandramycini (AY253864)
Kribbella albertanoniae (KC283016)
Kribbella yunnanensis (AY082061)
Kribbella antibiotica (AY082063)
Kribbella swartbergensis (AY995147)
Kribbella endophytica (HQ396152)
Kribbella alba (AY082062)
Kribbella lupini (AJ811962)
Kribbella italica (KJ875927)
Kribbella deserti (KX601688)
Nocardioides daedukensis (FJ842646)
Nocardioides jensenii (TYGS 3972)
Nocardioides halotolerans (TYGS 1402)
Nocardioides dilutus (EF466121)
Nocardioides fonticola (EF626689)
Nocardioides endophyticus (KC878444)
Nocardioides conyzicola (KC878445)
Nocardioides hankookensis (EF555584)
Nocardioides soli (JF937914)
Nocardioides hungaricus (AM981198)
Nocardioides aquiterrae (AF529063)
Nocardioides pyridinolyticus (U61298)
Nocardioides salsibiostraticola (KC152461)
Nocardioides caricicola (FJ750845)
Nocardioides islandensis (EF466123)
Nocardioides agariphilus (EF466113)
Nocardioides intraradicalis (KU940252)
Nocardioides sediminis (EF466110)
Nocardioides furvisabuli (DQ411542)
Nocardioides cavernae (KX815990)
Nocardioides hwasunensis (AM295258)
Nocardioides glacieisoli (JQ673418)
Nocardioides oleivorans (AJ698724)
Nocardioides ganghwensis (AY423718)
Nocardioides exalbidus (TYGS 2603)
Nocardioides flavus (KT962840)
Nocardioides zeicaulis (KU201963)
Nocardioides alpinus (GU784866)
Nocardioides terrigena (EF363712)
Nocardioides ginsengisegetis (GQ339901)
Nocardioides ungokensis (KP893899)
Nocardioides koreensis (EF466115)
Nocardioides albidus (KM073954)
Nocardioides daeguensis (HQ246164)

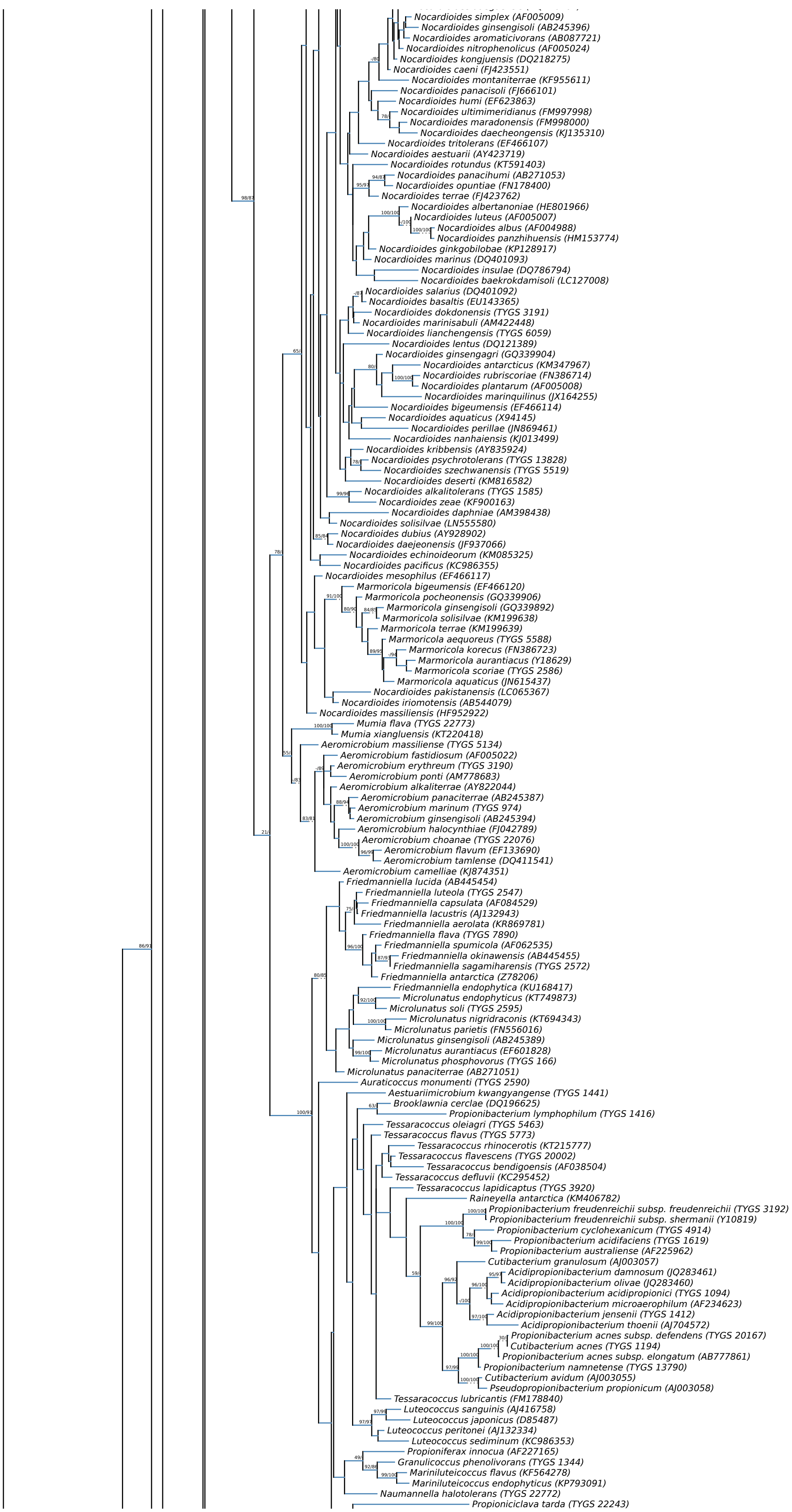

Nocardioides simplex (AF005009)
Nocardioides ginsengisoli (AB245396)
Nocardioides aromaticivorans (AB087721)
Nocardioides nitrophenolicus (AF005024)
Nocardioides kongjuensis (DQ218275)
Nocardioides caeni (FJ423551)
Nocardioides montaniterrae (KF955611)
Nocardioides panacisoli (FJ666101)
Nocardioides humi (EF623863)
Nocardioides ultimimeridianus (FM997998)
Nocardioides maradonensis (FM998000)
Nocardioides daecheongensis (KJ135310)
Nocardioides tritolerans (EF466107)
Nocardioides aestuarii (AY423719)
Nocardioides rotundus (KT591403)
Nocardioides panacihumi (AB271053)
Nocardioides opuntiae (FN178400)
Nocardioides terrae (FJ423762)
Nocardioides albertanoniae (HE801966)
Nocardioides luteus (AF005007)
Nocardioides albus (AF004988)
Nocardioides panzhihuensis (HM153774)
Nocardioides ginkgobilobae (KP128917)
Nocardioides marinus (DQ401093)
Nocardioides insulae (DQ786794)
Nocardioides baekrokdamisoli (LC127008)
Nocardioides salarius (DQ401092)
Nocardioides basaltis (EU143365)
Nocardioides dokdonensis (TYGS 3191)
Nocardioides marinisabuli (AM422448)
Nocardioides lianchengensis (TYGS 6059)
Nocardioides lentus (DQ121389)
Nocardioides ginsengagri (GQ339904)
Nocardioides antarcticus (KM347967)
Nocardioides rubriscoriae (FN386714)
Nocardioides plantarum (AF005008)
Nocardioides marinquilinus (JX164255)
Nocardioides bigeumensis (EF466114)
Nocardioides aquaticus (X94145)
Nocardioides perillae (JN869461)
Nocardioides nanhaiensis (KJ013499)
Nocardioides kribbensis (AY835924)
Nocardioides psychrotolerans (TYGS 13828)
Nocardioides szechwanensis (TYGS 5519)
Nocardioides deserti (KM816582)
Nocardioides alkalitolerans (TYGS 1585)
Nocardioides zeae (KF900163)
Nocardioides daphniae (AM398438)
Nocardioides solisilvae (LN555580)
Nocardioides dubius (AY928902)
Nocardioides daejeonensis (JF937066)
Nocardioides echinoideorum (KM085325)
Nocardioides pacificus (KC986355)
Nocardioides mesophilus (EF466117)
Marmoricola bigeumensis (EF466120)
Marmoricola pocheonensis (GQ339906)
Marmoricola ginsengisoli (GQ339892)
Marmoricola solisilvae (KM199638)
Marmoricola terrae (KM199639)
Marmoricola aequoreus (TYGS 5588)
Marmoricola korecus (FN386723)
Marmoricola aurantiacus (Y18629)
Marmoricola scoriae (TYGS 2586)
Marmoricola aquaticus (JN615437)
Nocardioides pakistanensis (LC065367)
Nocardioides iriomotensis (AB544079)
Nocardioides massiliensis (HF952922)
Mumia flava (TYGS 22773)
Mumia xiangluensis (KT220418)
Aeromicrobium massiliense (TYGS 5134)
Aeromicrobium fastidiosum (AF005022)
Aeromicrobium erythreum (TYGS 3190)
Aeromicrobium ponti (AM778683)
Aeromicrobium alkaliterrae (AY822044)
Aeromicrobium panaciterrae (AB245387)
Aeromicrobium marinum (TYGS 974)
Aeromicrobium ginsengisoli (AB245394)
Aeromicrobium halocynthiae (FJ042789)
Aeromicrobium choanae (TYGS 22076)
Aeromicrobium flavum (EF133690)
Aeromicrobium tamlense (DQ411541)
Aeromicrobium camelliae (KJ874351)
Friedmanniella lucida (AB445454)
Friedmanniella luteola (TYGS 2547)
Friedmanniella capsulata (AF084529)
Friedmanniella lacustris (AJ132943)
Friedmanniella aerolata (KR869781)
Friedmanniella flava (TYGS 7890)
Friedmanniella spumicola (AF062535)
Friedmanniella okinawensis (AB445455)
Friedmanniella sagamiharensis (TYGS 2572)
Friedmanniella antarctica (Z78206)
Friedmanniella endophytica (KU168417)
Microlunatus endophyticus (KT749873)
Microlunatus soli (TYGS 2595)
Microlunatus nigridraconis (KT694343)
Microlunatus parietis (FN556016)
Microlunatus ginsengisoli (AB245389)
Microlunatus aurantiacus (EF601828)
Microlunatus phosphovorus (TYGS 166)
Microlunatus panaciterrae (AB271051)
Auraticoccus monumenti (TYGS 2590)
Aestuariimicrobium kwangyangense (TYGS 1441)
Brooklawnia cerclae (DQ196625)
Propionibacterium lymphophilum (TYGS 1416)
Tessaracoccus oleiagri (TYGS 5463)
Tessaracoccus flavus (TYGS 5773)
Tessaracoccus rhinocerotis (KT215777)
Tessaracoccus flavescens (TYGS 20002)
Tessaracoccus bendigoensis (AF038504)
Tessaracoccus defluvii (KC295452)
Tessaracoccus lapidicaptus (TYGS 3920)
Raineyella antarctica (KM406782)
Propionibacterium freudenreichii subsp. freudenreichii (TYGS 3192)
Propionibacterium freudenreichii subsp. shermanii (Y10819)
Propionibacterium cyclohexanicum (TYGS 4914)
Propionibacterium acidifaciens (TYGS 1619)
Propionibacterium australiense (AF225962)
Cutibacterium granulosum (AJ003057)
Acidipropionibacterium damnosum (JQ283461)
Acidipropionibacterium olivae (JQ283460)
Acidipropionibacterium acidipropionici (TYGS 1094)
Acidipropionibacterium microaerophilum (AF234623)
Acidipropionibacterium jensenii (TYGS 1412)
Acidipropionibacterium thoenii (AJ704572)
Propionibacterium acnes subsp. defendens (TYGS 20167)
Cutibacterium acnes (TYGS 1194)
Propionibacterium acnes subsp. elongatum (AB777861)
Propionibacterium namnetense (TYGS 13790)
Cutibacterium avidum (AJ003055)
Pseudopropionibacterium propionicum (AJ003058)
Tessaracoccus lubricantis (FM178840)
Luteococcus sanguinis (AJ416758)
Luteococcus japonicus (D85487)
Luteococcus peritonei (AJ132334)
Luteococcus sediminum (KC986353)
Propioniferax innocua (AF227165)
Granulicoccus phenolivorans (TYGS 1344)
Mariniluteicoccus flavus (KF564278)
Mariniluteicoccus endophyticus (KP793091)
Naumannella halotolerans (TYGS 22772)
Propioniciclava tarda (TYGS 22243)

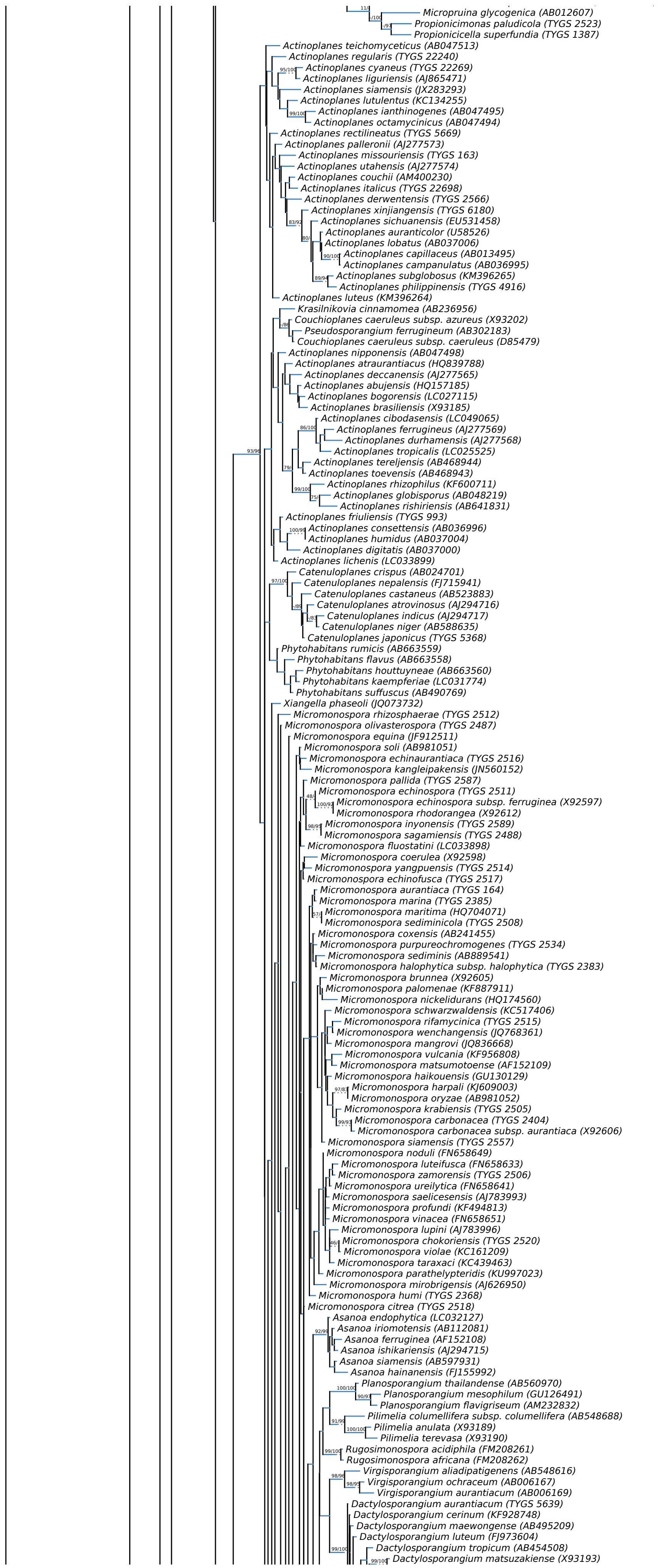
Micropruina glycogenica (AB012607)
Propionicimonas paludicola (TYGS 2523)
Propionicicella superfundia (TYGS 1387)
Actinoplanes teichomyceticus (AB047513)
Actinoplanes regularis (TYGS 22240)
Actinoplanes cyaneus (TYGS 22269)
Actinoplanes liguriensis (AJ865471)
Actinoplanes siamensis (JX283293)
Actinoplanes lutulentus (KC134255)
Actinoplanes ianthinogenes (AB047495)
Actinoplanes octamycinicus (AB047494)
Actinoplanes rectilineatus (TYGS 5669)
Actinoplanes palleronii (AJ277573)
Actinoplanes missouriensis (TYGS 163)
Actinoplanes utahensis (AJ277574)
Actinoplanes couchii (AM400230)
Actinoplanes italicus (TYGS 22698)
Actinoplanes derwentensis (TYGS 2566)
Actinoplanes xinjiangensis (TYGS 6180)
Actinoplanes sichuanensis (EU531458)
Actinoplanes auranticolor (U58526)
Actinoplanes lobatus (AB037006)
Actinoplanes capillaceus (AB013495)
Actinoplanes campanulatus (AB036995)
Actinoplanes subglobosus (KM396265)
Actinoplanes philippinensis (TYGS 4916)
Actinoplanes luteus (KM396264)
Krasilnikovia cinnamomea (AB236956)
Couchioplanes caeruleus subsp. azureus (X93202)
Pseudosporangium ferrugineum (AB302183)
Couchioplanes caeruleus subsp. caeruleus (D85479)
Actinoplanes nipponensis (AB047498)
Actinoplanes atraurantiacus (HQ839788)
Actinoplanes deccanensis (AJ277565)
Actinoplanes abujensis (HQ157185)
Actinoplanes bogorensis (LC027115)
Actinoplanes brasiliensis (X93185)
Actinoplanes cibodasensis (LC049065)
Actinoplanes ferrugineus (AJ277569)
Actinoplanes durhamensis (AJ277568)
Actinoplanes tropicalis (LC025525)
Actinoplanes tereljensis (AB468944)
Actinoplanes toevensis (AB468943)
Actinoplanes rhizophilus (KF600711)
Actinoplanes globisporus (AB048219)
Actinoplanes rishiriensis (AB641831)
Actinoplanes friuliensis (TYGS 993)
Actinoplanes consettensis (AB036996)
Actinoplanes humidus (AB037004)
Actinoplanes digitatis (AB037000)
Actinoplanes lichenis (LC033899)
Catenuloplanes crispus (AB024701)
Catenuloplanes nepalensis (FJ715941)
Catenuloplanes castaneus (AB523883)
Catenuloplanes atrovinosus (AJ294716)
Catenuloplanes indicus (AJ294717)
Catenuloplanes niger (AB588635)
Catenuloplanes japonicus (TYGS 5368)
Phytohabitans rumicis (AB663559)
Phytohabitans flavus (AB663558)
Phytohabitans houttuyneae (AB663560)
Phytohabitans kaempferiae (LC031774)
Phytohabitans suffuscus (AB490769)
Xiangella phaseoli (JQ073732)
Micromonospora rhizosphaerae (TYGS 2512)
Micromonospora olivasterospora (TYGS 2487)
Micromonospora equina (JF912511)
Micromonospora soli (AB981051)
Micromonospora echinaurantiaca (TYGS 2516)
Micromonospora kangleipakensis (JN560152)
Micromonospora pallida (TYGS 2587)
Micromonospora echinospora (TYGS 2511)
Micromonospora echinospora subsp. ferruginea (X92597)
Micromonospora rhodorangea (X92612)
Micromonospora inyonensis (TYGS 2589)
Micromonospora sagamiensis (TYGS 2488)
Micromonospora fluostatini (LC033898)
Micromonospora coerulea (X92598)
Micromonospora yangpuensis (TYGS 2514)
Micromonospora echinofusca (TYGS 2517)
Micromonospora aurantiaca (TYGS 164)
Micromonospora marina (TYGS 2385)
Micromonospora maritima (HQ704071)
Micromonospora sediminicola (TYGS 2508)
Micromonospora coxensis (AB241455)
Micromonospora purpureochromogenes (TYGS 2534)
Micromonospora sediminis (AB889541)
Micromonospora halophytica subsp. halophytica (TYGS 2383)
Micromonospora brunnea (X92605)
Micromonospora palomenae (KF887911)
Micromonospora nickelidurans (HQ174560)
Micromonospora schwarzwaldensis (KC517406)
Micromonospora rifamycinica (TYGS 2515)
Micromonospora wenchangensis (JQ768361)
Micromonospora mangrovi (JQ836668)
Micromonospora vulcania (KF956808)
Micromonospora matsumotoense (AF152109)
Micromonospora haikouensis (GU130129)
Micromonospora harpali (KJ609003)
Micromonospora oryzae (AB981052)
Micromonospora krabiensis (TYGS 2505)
Micromonospora carbonacea (TYGS 2404)
Micromonospora carbonacea subsp. aurantiaca (X92606)
Micromonospora siamensis (TYGS 2557)
Micromonospora noduli (FN658649)
Micromonospora luteifusca (FN658633)
Micromonospora zamorensis (TYGS 2506)
Micromonospora ureilytica (FN658641)
Micromonospora saelicesensis (AJ783993)
Micromonospora profundi (KF494813)
Micromonospora vinacea (FN658651)
Micromonospora lupini (AJ783996)
Micromonospora chokoriensis (TYGS 2520)
Micromonospora violae (KC161209)
Micromonospora taraxaci (KC439463)
Micromonospora parathelypteridis (KU997023)
Micromonospora mirobrigensis (AJ626950)
Micromonospora humi (TYGS 2368)
Micromonospora citrea (TYGS 2518)
Asanoa endophytica (LC032127)
Asanoa iriomotensis (AB112081)
Asanoa ferruginea (AF152108)
Asanoa ishikariensis (AJ294715)
Asanoa siamensis (AB597931)
Asanoa hainanensis (FJ155992)
Planosporangium thailandense (AB560970)
Planosporangium mesophilum (GU126491)
Planosporangium flavigriseum (AM232832)
Pilimelia columellifera subsp. columellifera (AB548688)
Pilimelia anulata (X93189)
Pilimelia terevasa (X93190)
Rugosimonospora acidiphila (FM208261)
Rugosimonospora africana (FM208262)
Virgisporangium aliadipatigenens (AB548616)
Virgisporangium ochraceum (AB006167)
Virgisporangium aurantiacum (AB006169)
Dactylosporangium aurantiacum (TYGS 5639)
Dactylosporangium cerinum (KF928748)
Dactylosporangium maewongense (AB495209)
Dactylosporangium luteum (FJ973604)
Dactylosporangium tropicum (AB454508)
Dactylosporangium matsuzakiense (X93193)

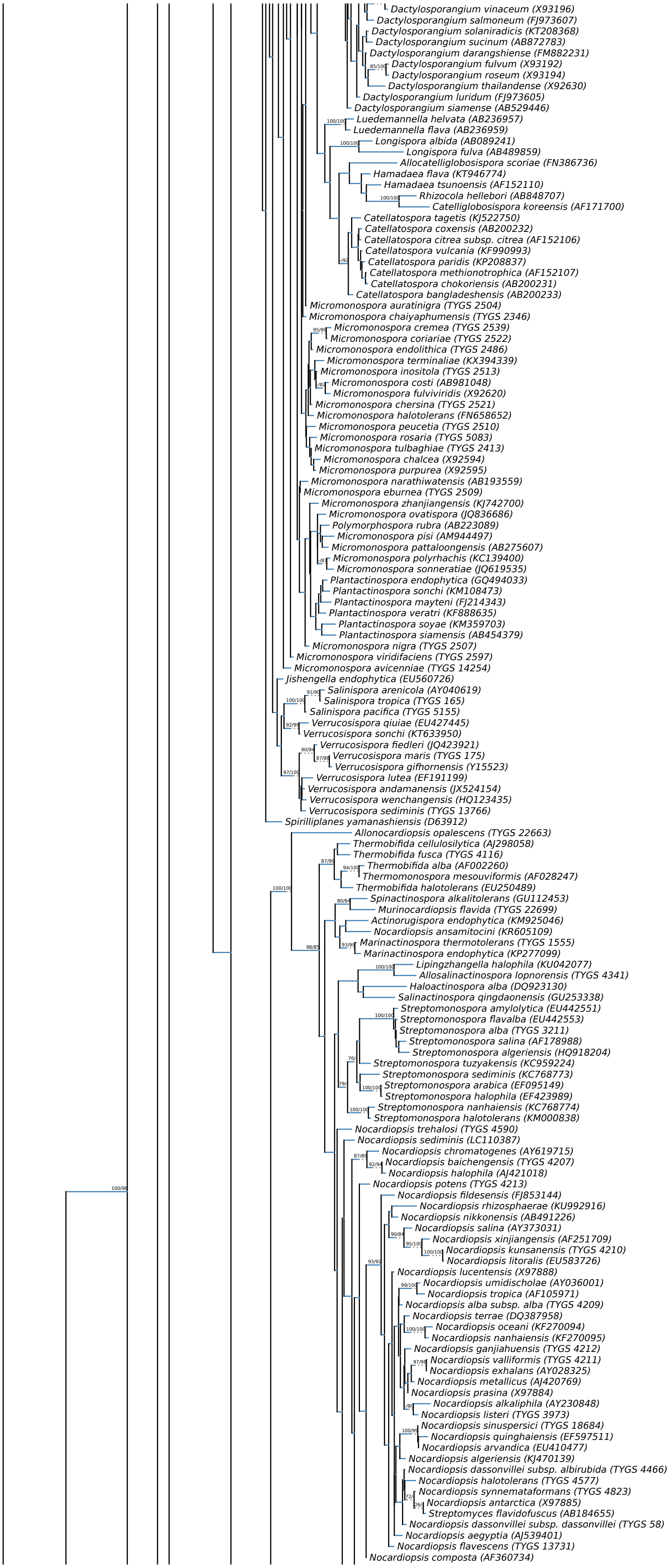

Dactylosporangium vinaceum (X93196)
Dactylosporangium salmoneum (FJ973607)
Dactylosporangium solaniradicis (KT208368)
Dactylosporangium sucinum (AB872783)
Dactylosporangium darangshiense (FM882231)
Dactylosporangium fulvum (X93192)
Dactylosporangium roseum (X93194)
Dactylosporangium thailandense (X92630)
Dactylosporangium luridum (FJ973605)
Dactylosporangium siamense (AB529446)
Luedemannella helvata (AB236957)
Luedemannella flava (AB236959)
Longispora albida (AB089241)
Longispora fulva (AB489859)
Allocatelliglobosispora scoriae (FN386736)
Hamadaea flava (KT946774)
Hamadaea tsunoensis (AF152110)
Rhizocola hellebori (AB848707)
Catelliglobosispora koreensis (AF171700)
Catellatospora tagetis (KJ522750)
Catellatospora coxensis (AB200232)
Catellatospora citrea subsp. citrea (AF152106)
Catellatospora vulcania (KF990993)
Catellatospora paridis (KP208837)
Catellatospora methionotrophica (AF152107)
Catellatospora chokoriensis (AB200231)
Catellatospora bangladeshensis (AB200233)
Micromonospora auratinigra (TYGS 2504)
Micromonospora chaiyaphumensis (TYGS 2346)
Micromonospora cremea (TYGS 2539)
Micromonospora coriariae (TYGS 2522)
Micromonospora endolithica (TYGS 2486)
Micromonospora terminaliae (KX394339)
Micromonospora inositola (TYGS 2513)
Micromonospora costi (AB981048)
Micromonospora fulviviridis (X92620)
Micromonospora chersina (TYGS 2521)
Micromonospora halotolerans (FN658652)
Micromonospora peucetia (TYGS 2510)
Micromonospora rosaria (TYGS 5083)
Micromonospora tulbaghiae (TYGS 2413)
Micromonospora chalcea (X92594)
Micromonospora purpurea (X92595)
Micromonospora narathiwatensis (AB193559)
Micromonospora eburnea (TYGS 2509)
Micromonospora zhanjiangensis (KJ742700)
Micromonospora ovatispora (JQ836686)
Polymorphospora rubra (AB223089)
Micromonospora pisi (AM944497)
Micromonospora pattaloongensis (AB275607)
Micromonospora polyrhachis (KC139400)
Micromonospora sonneratiae (JQ619535)
Plantactinospora endophytica (GQ494033)
Plantactinospora sonchi (KM108473)
Plantactinospora mayteni (FJ214343)
Plantactinospora veratri (KF888635)
Plantactinospora soyae (KM359703)
Plantactinospora siamensis (AB454379)
Micromonospora nigra (TYGS 2507)
Micromonospora viridifaciens (TYGS 2597)
Micromonospora avicenniae (TYGS 14254)
Jishengella endophytica (EU560726)
Salinispora arenicola (AY040619)
Salinispora tropica (TYGS 165)
Salinispora pacifica (TYGS 5155)
Verrucosispora qiuiae (EU427445)
Verrucosispora sonchi (KT633950)
Verrucosispora fiedleri (JQ423921)
Verrucosispora maris (TYGS 175)
Verrucosispora gifhornensis (Y15523)
Verrucosispora lutea (EF191199)
Verrucosispora andamanensis (JX524154)
Verrucosispora wenchangensis (HQ123435)
Verrucosispora sediminis (TYGS 13766)
Spirilliplanes yamanashiensis (D63912)
Allonocardiopsis opalescens (TYGS 22663)
Thermobifida cellulosilytica (AJ298058)
Thermobifida fusca (TYGS 4116)
Thermobifida alba (AF002260)
Thermomonospora mesouviformis (AF028247)
Thermobifida halotolerans (EU250489)
Spinactinospora alkalitolerans (GU112453)
Murinocardiopsis flavida (TYGS 22699)
Actinorugispora endophytica (KM925046)
Nocardiopsis ansamitocini (KR605109)
Marinactinospora thermotolerans (TYGS 1555)
Marinactinospora endophytica (KP277099)
Lipingzhangella halophila (KU042077)
Allosalinactinospora lopnorensis (TYGS 4341)
Haloactinospora alba (DQ923130)
Salinactinospora qingdaonensis (GU253338)
Streptomonospora amylolytica (EU442551)
Streptomonospora flavalba (EU442553)
Streptomonospora alba (TYGS 3211)
Streptomonospora salina (AF178988)
Streptomonospora algeriensis (HQ918204)
Streptomonospora tuzyakensis (KC959224)
Streptomonospora sediminis (KC768773)
Streptomonospora arabica (EF095149)
Streptomonospora halophila (EF423989)
Streptomonospora nanhaiensis (KC768774)
Streptomonospora halotolerans (KM000838)
Nocardiopsis trehalosi (TYGS 4590)
Nocardiopsis sediminis (LC110387)
Nocardiopsis chromatogenes (AY619715)
Nocardiopsis baichengensis (TYGS 4207)
Nocardiopsis halophila (AJ421018)
Nocardiopsis potens (TYGS 4213)
Nocardiopsis fildesensis (FJ853144)
Nocardiopsis rhizosphaerae (KU992916)
Nocardiopsis nikkonensis (AB491226)
Nocardiopsis salina (AY373031)
Nocardiopsis xinjiangensis (AF251709)
Nocardiopsis kunsanensis (TYGS 4210)
Nocardiopsis litoralis (EU583726)
Nocardiopsis lucentensis (X97888)
Nocardiopsis umidischolae (AY036001)
Nocardiopsis tropica (AF105971)
Nocardiopsis alba subsp. alba (TYGS 4209)
Nocardiopsis terrae (DQ387958)
Nocardiopsis oceani (KF270094)
Nocardiopsis nanhaiensis (KF270095)
Nocardiopsis ganjiahuensis (TYGS 4212)
Nocardiopsis valliformis (TYGS 4211)
Nocardiopsis exhalans (AY028325)
Nocardiopsis metallicus (AJ420769)
Nocardiopsis prasina (X97884)
Nocardiopsis alkaliphila (AY230848)
Nocardiopsis listeri (TYGS 3973)
Nocardiopsis sinuspersici (TYGS 18684)
Nocardiopsis quinghaiensis (EF597511)
Nocardiopsis arvandica (EU410477)
Nocardiopsis algeriensis (KJ470139)
Nocardiopsis dassonvillei subsp. albirubida (TYGS 4466)
Nocardiopsis halotolerans (TYGS 4577)
Nocardiopsis synnemataformans (TYGS 4823)
Nocardiopsis antarctica (X97885)
Streptomyces flavidofuscus (AB184655)
Nocardiopsis dassonvillei subsp. dassonvillei (TYGS 58)
Nocardiopsis aegyptia (AJ539401)
Nocardiopsis flavescens (TYGS 13731)
Nocardiopsis composta (AF360734)

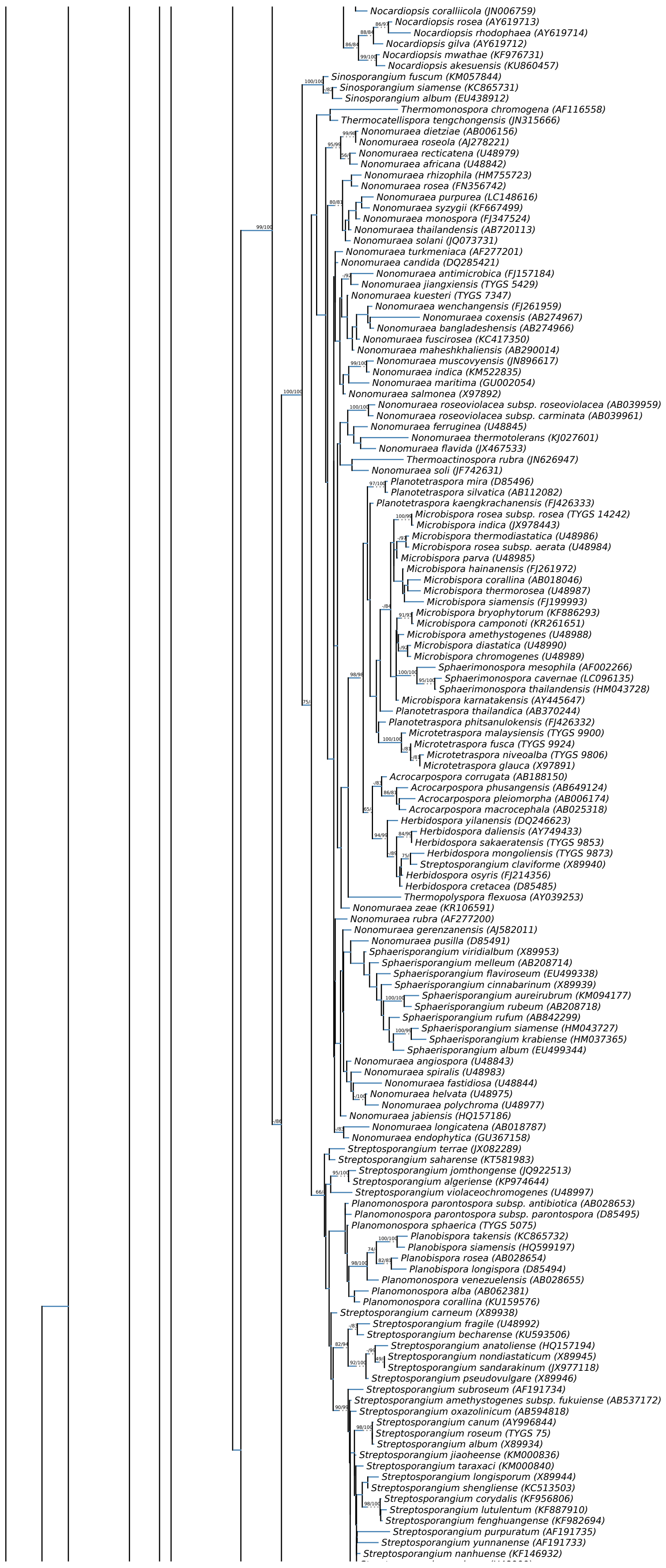
Nocardiopsis coralliicola (JN006759)
Nocardiopsis rosea (AY619713)
Nocardiopsis rhodophaea (AY619714)
Nocardiopsis gilva (AY619712)
Nocardiopsis mwathae (KF976731)
Nocardiopsis akesuensis (KU860457)
Sinosporangium fuscum (KM057844)
Sinosporangium siamense (KC865731)
Sinosporangium album (EU438912)
Thermomonospora chromogena (AF116558)
Thermocatellispora tengchongensis (JN315666)
Nonomuraea dietziae (AB006156)
Nonomuraea roseola (AJ278221)
Nonomuraea recticatena (U48979)
Nonomuraea africana (U48842)
Nonomuraea rhizophila (HM755723)
Nonomuraea rosea (FN356742)
Nonomuraea purpurea (LC148616)
Nonomuraea syzygii (KF667499)
Nonomuraea monospora (FJ347524)
Nonomuraea thailandensis (AB720113)
Nonomuraea solani (JQ073731)
Nonomuraea turkmeniaca (AF277201)
Nonomuraea candida (DQ285421)
Nonomuraea antimicrobica (FJ157184)
Nonomuraea jiangxiensis (TYGS 5429)
Nonomuraea kuesteri (TYGS 7347)
Nonomuraea wenchangensis (FJ261959)
Nonomuraea coxensis (AB274967)
Nonomuraea bangladeshensis (AB274966)
Nonomuraea fuscirosea (KC417350)
Nonomuraea maheshkhaliensis (AB290014)
Nonomuraea muscovyensis (JN896617)
Nonomuraea indica (KM522835)
Nonomuraea maritima (GU002054)
Nonomuraea salmonea (X97892)
Nonomuraea roseoviolacea subsp. roseoviolacea (AB039959)
Nonomuraea roseoviolacea subsp. carminata (AB039961)
Nonomuraea ferruginea (U48845)
Nonomuraea thermotolerans (KJ027601)
Nonomuraea flavida (JX467533)
Thermoactinospora rubra (JN626947)
Nonomuraea soli (JF742631)
Planotetraspora mira (D85496)
Planotetraspora silvatica (AB112082)
Planotetraspora kaengkrachanensis (FJ426333)
Microbispora rosea subsp. rosea (TYGS 14242)
Microbispora indica (JX978443)
Microbispora thermodiastatica (U48986)
Microbispora rosea subsp. aerata (U48984)
Microbispora parva (U48985)
Microbispora hainanensis (FJ261972)
Microbispora corallina (AB018046)
Microbispora thermorosea (U48987)
Microbispora siamensis (FJ199993)
Microbispora bryophytorum (KF886293)
Microbispora camponoti (KR261651)
Microbispora amethystogenes (U48988)
Microbispora diastatica (U48990)
Microbispora chromogenes (U48989)
Sphaerimonospora mesophila (AF002266)
Sphaerimonospora cavernae (LC096135)
Sphaerimonospora thailandensis (HM043728)
Microbispora karnatakensis (AY445647)
Planotetraspora thailandica (AB370244)
Planotetraspora phitsanulokensis (FJ426332)
Microtetraspora malaysiensis (TYGS 9900)
Microtetraspora fusca (TYGS 9924)
Microtetraspora niveoalba (TYGS 9806)
Microtetraspora glauca (X97891)
Acrocarpospora corrugata (AB188150)
Acrocarpospora phusangensis (AB649124)
Acrocarpospora pleiomorpha (AB006174)
Acrocarpospora macrocephala (AB025318)
Herbidospora yilanensis (DQ246623)
Herbidospora daliensis (AY749433)
Herbidospora sakaeratensis (TYGS 9853)
Herbidospora mongoliensis (TYGS 9873)
Streptosporangium claviforme (X89940)
Herbidospora osyris (FJ214356)
Herbidospora cretacea (D85485)
Thermopolyspora flexuosa (AY039253)
Nonomuraea zeae (KR106591)
Nonomuraea rubra (AF277200)
Nonomuraea gerenzanensis (AJ582011)
Nonomuraea pusilla (D85491)
Sphaerisporangium viridialbum (X89953)
Sphaerisporangium melleum (AB208714)
Sphaerisporangium flaviroseum (EU499338)
Sphaerisporangium cinnabarinum (X89939)
Sphaerisporangium aureirubrum (KM094177)
Sphaerisporangium rubeum (AB208718)
Sphaerisporangium rufum (AB842299)
Sphaerisporangium siamense (HM043727)
Sphaerisporangium krabiense (HM037365)
Sphaerisporangium album (EU499344)
Nonomuraea angiospora (U48843)
Nonomuraea spiralis (U48983)
Nonomuraea fastidiosa (U48844)
Nonomuraea helvata (U48975)
Nonomuraea polychroma (U48977)
Nonomuraea jabiensis (HQ157186)
Nonomuraea longicatena (AB018787)
Nonomuraea endophytica (GU367158)
Streptosporangium terrae (JX082289)
Streptosporangium saharense (KT581983)
Streptosporangium jomthongense (JQ922513)
Streptosporangium algeriense (KP974644)
Streptosporangium violaceochromogenes (U48997)
Planomonospora parontospora subsp. antibiotica (AB028653)
Planomonospora parontospora subsp. parontospora (D85495)
Planomonospora sphaerica (TYGS 5075)
Planobispora takensis (KC865732)
Planobispora siamensis (HQ599197)
Planobispora rosea (AB028654)
Planobispora longispora (D85494)
Planomonospora venezuelensis (AB028655)
Planomonospora alba (AB062381)
Planomonospora corallina (KU159576)
Streptosporangium carneum (X89938)
Streptosporangium fragile (U48992)
Streptosporangium becharense (KU593506)
Streptosporangium anatoliense (HQ157194)
Streptosporangium nondiastaticum (X89945)
Streptosporangium sandarakinum (JX977118)
Streptosporangium pseudovulgare (X89946)
Streptosporangium subroseum (AF191734)
Streptosporangium amethystogenes subsp. fukuiense (AB537172)
Streptosporangium oxazolinicum (AB594818)
Streptosporangium canum (AY996844)
Streptosporangium roseum (TYGS 75)
Streptosporangium album (X89934)
Streptosporangium jiaoheense (KM000836)
Streptosporangium taraxaci (KM000840)
Streptosporangium longisporum (X89944)
Streptosporangium shengliense (KC513503)
Streptosporangium corydalis (KF956806)
Streptosporangium lutulentum (KF887910)
Streptosporangium fenghuangense (KF982694)
Streptosporangium purpuratum (AF191735)
Streptosporangium yunnanense (AF191733)
Streptosporangium nanhuense (KF146932)

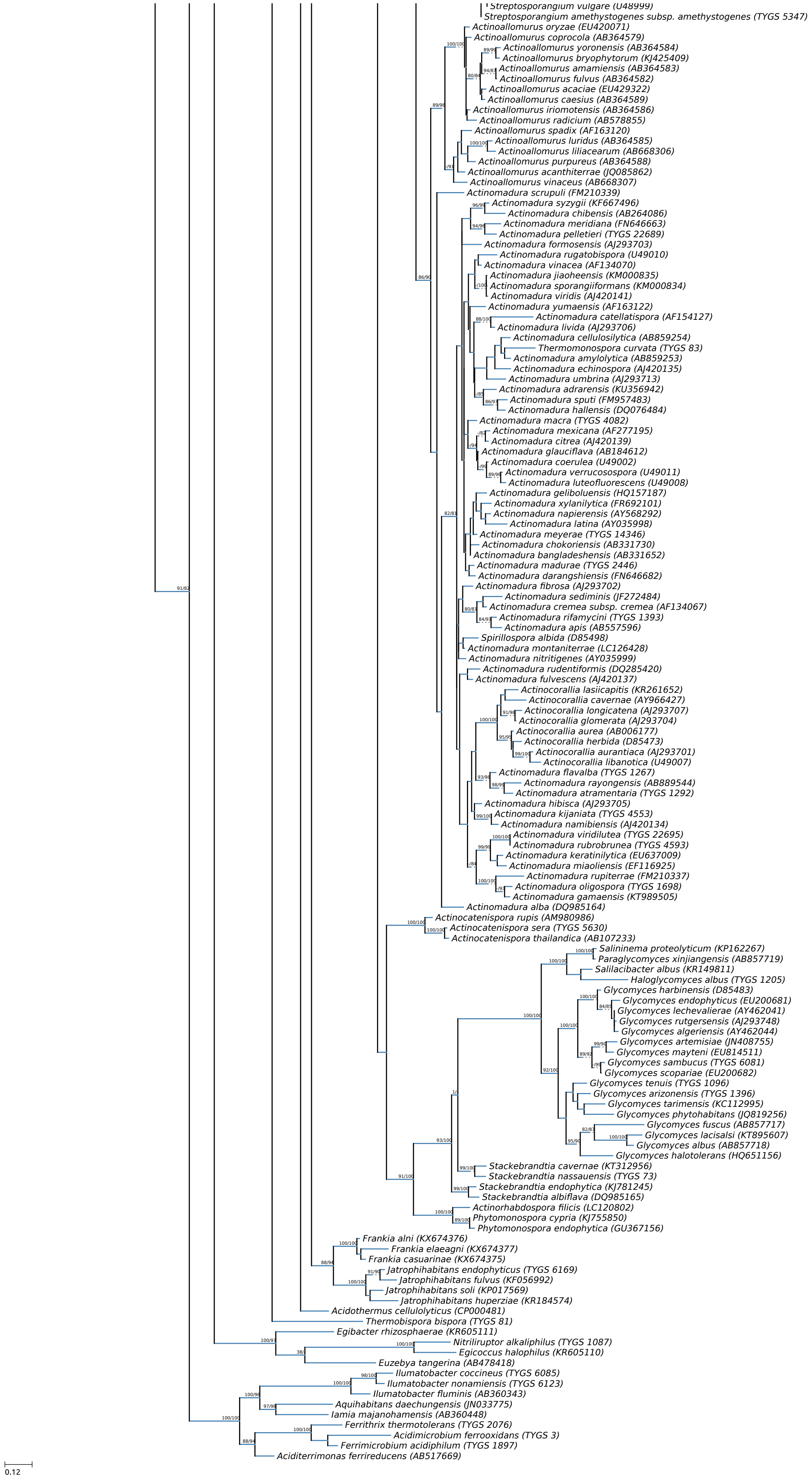
Streptosporangium vulgare (U48999)
Streptosporangium amethystogenes subsp. amethystogenes (TYGS 5347)
Actinoallomurus oryzae (EU420071)
Actinoallomurus coprocola (AB364579)
Actinoallomurus yoronensis (AB364584)
Actinoallomurus bryophytorum (KJ425409)
Actinoallomurus amamiensis (AB364583)
Actinoallomurus fulvus (AB364582)
Actinoallomurus acaciae (EU429322)
Actinoallomurus caesius (AB364589)
Actinoallomurus iriomotensis (AB364586)
Actinoallomurus radicium (AB578855)
Actinoallomurus spadix (AF163120)
Actinoallomurus luridus (AB364585)
Actinoallomurus liliacearum (AB668306)
Actinoallomurus purpureus (AB364588)
Actinoallomurus acanthiterrae (JQ085862)
Actinoallomurus vinaceus (AB668307)
Actinomadura scrupuli (FM210339)
Actinomadura syzygii (KF667496)
Actinomadura chibensis (AB264086)
Actinomadura meridiana (FN646663)
Actinomadura pelletieri (TYGS 22689)
Actinomadura formosensis (AJ293703)
Actinomadura rugatobispora (U49010)
Actinomadura vinacea (AF134070)
Actinomadura jiaoheensis (KM000835)
Actinomadura sporangiiformans (KM000834)
Actinomadura viridis (AJ420141)
Actinomadura yumaensis (AF163122)
Actinomadura catellatispora (AF154127)
Actinomadura livida (AJ293706)
Actinomadura cellulosilytica (AB859254)
Thermomonospora curvata (TYGS 83)
Actinomadura amylolytica (AB859253)
Actinomadura echinospora (AJ420135)
Actinomadura umbrina (AJ293713)
Actinomadura adrarensis (KU356942)
Actinomadura sputi (FM957483)
Actinomadura hallensis (DQ076484)
Actinomadura macra (TYGS 4082)
Actinomadura mexicana (AF277195)
Actinomadura citrea (AJ420139)
Actinomadura glauciflava (AB184612)
Actinomadura coerulea (U49002)
Actinomadura verrucosospora (U49011)
Actinomadura luteofluorescens (U49008)
Actinomadura geliboluensis (HQ157187)
Actinomadura xylanilytica (FR692101)
Actinomadura napierensis (AY568292)
Actinomadura latina (AY035998)
Actinomadura meyerae (TYGS 14346)
Actinomadura chokoriensis (AB331730)
Actinomadura bangladeshensis (AB331652)
Actinomadura madurae (TYGS 2446)
Actinomadura darangshiensis (FN646682)
Actinomadura fibrosa (AJ293702)
Actinomadura sediminis (JF272484)
Actinomadura cremea subsp. cremea (AF134067)
Actinomadura rifamycini (TYGS 1393)
Actinomadura apis (AB557596)
Spirillospora albida (D85498)
Actinomadura montaniterrae (LC126428)
Actinomadura nitritigenes (AY035999)
Actinomadura rudentiformis (DQ285420)
Actinomadura fulvescens (AJ420137)
Actinocorallia lasiicapitis (KR261652)
Actinocorallia cavernae (AY966427)
Actinocorallia longicatena (AJ293707)
Actinocorallia glomerata (AJ293704)
Actinocorallia aurea (AB006177)
Actinocorallia herbida (D85473)
Actinocorallia aurantiaca (AJ293701)
Actinocorallia libanotica (U49007)
Actinomadura flavalba (TYGS 1267)
Actinomadura rayongensis (AB889544)
Actinomadura atramentaria (TYGS 1292)
Actinomadura hibisca (AJ293705)
Actinomadura kijaniata (TYGS 4553)
Actinomadura namibiensis (AJ420134)
Actinomadura viridilutea (TYGS 22695)
Actinomadura rubrobrunea (TYGS 4593)
Actinomadura keratinilytica (EU637009)
Actinomadura miaoliensis (EF116925)
Actinomadura rupiterrae (FM210337)
Actinomadura oligospora (TYGS 1698)
Actinomadura gamaensis (KT989505)
Actinomadura alba (DQ985164)
Actinocatenispora rupis (AM980986)
Actinocatenispora sera (TYGS 5630)
Actinocatenispora thailandica (AB107233)
Salininema proteolyticum (KP162267)
Paraglycomyces xinjiangensis (AB857719)
Salilacibacter albus (KR149811)
Haloglycomyces albus (TYGS 1205)
Glycomyces harbinensis (D85483)
Glycomyces endophyticus (EU200681)
Glycomyces lechevalierae (AY462041)
Glycomyces rutgersensis (AJ293748)
Glycomyces algeriensis (AY462044)
Glycomyces artemisiae (JN408755)
Glycomyces mayteni (EU814511)
Glycomyces sambucus (TYGS 6081)
Glycomyces scopariae (EU200682)
Glycomyces tenuis (TYGS 1096)
Glycomyces arizonensis (TYGS 1396)
Glycomyces tarimensis (KC112995)
Glycomyces phytohabitans (JQ819256)
Glycomyces fuscus (AB857717)
Glycomyces lacisalsi (KT895607)
Glycomyces albus (AB857718)
Glycomyces halotolerans (HQ651156)
Stackebrandtia cavernae (KT312956)
Stackebrandtia nassauensis (TYGS 73)
Stackebrandtia endophytica (KJ781245)
Stackebrandtia albiflava (DQ985165)
Actinorhabdospora filicis (LC120802)
Phytomonospora cypria (KJ755850)
Phytomonospora endophytica (GU367156)
Frankia alni (KX674376)
Frankia elaeagni (KX674377)
Frankia casuarinae (KX674375)
Jatrophihabitans endophyticus (TYGS 6169)
Jatrophihabitans fulvus (KF056992)
Jatrophihabitans soli (KP017569)
Jatrophihabitans huperziae (KR184574)
Acidothermus cellulolyticus (CP000481)
Thermobispora bispora (TYGS 81)
Egibacter rhizosphaerae (KR605111)
Nitriliruptor alkaliphilus (TYGS 1087)
Egicoccus halophilus (KR605110)
Euzebya tangerina (AB478418)
Ilumatobacter coccineus (TYGS 6085)
Ilumatobacter nonamiensis (TYGS 6123)
Ilumatobacter fluminis (AB360343)
Aquihabitans daechungensis (JN033775)
Iamia majanohamensis (AB360448)
Ferrithrix thermotolerans (TYGS 2076)
Acidimicrobium ferrooxidans (TYGS 3)
Ferrimicrobium acidiphilum (TYGS 1897)
Aciditerrimonas ferrireducens (AB517669)
0.12

Figure 3: Unconstrained comprehensive 16S rRNA gene ML and MP tree (UCT). Dotted parts of branches are filled in to allow proper placement of bootstrap values and are not part of the actual branch length.

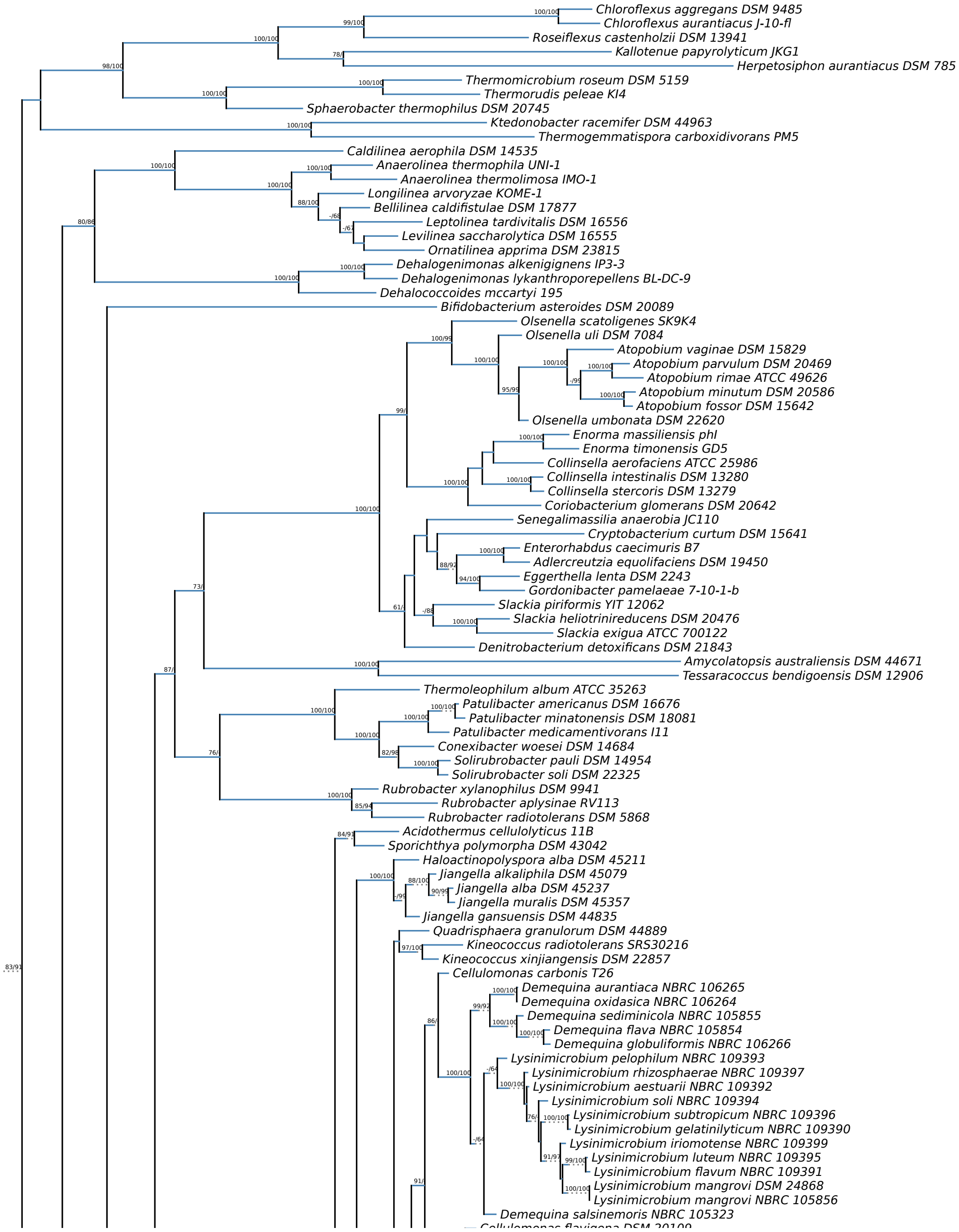
Chloroflexus aggregans DSM 9485
Chloroflexus aurantiacus J-10-fl
Roseiflexus castenholzii DSM 13941
Kallotenue papyrolyticum JKG1
Herpetosiphon aurantiacus DSM 785
Thermomicrobium roseum DSM 5159
Thermorudis peleae KI4
Sphaerobacter thermophilus DSM 20745
Ktedonobacter racemifer DSM 44963
Thermogemmatispora carboxidivorans PM5
Caldilinea aerophila DSM 14535
Anaerolinea thermophila UNI-1
Anaerolinea thermolimosa IMO-1
Longilinea arvoryzae KOME-1
Bellilinea caldifistulae DSM 17877
Leptolinea tardivitalis DSM 16556
Levilinea saccharolytica DSM 16555
Ornatilinea apprima DSM 23815
Dehalogenimonas alkenigignens IP3-3
Dehalogenimonas lykanthroporepellens BL-DC-9
Dehalococcoides mccartyi 195
Bifidobacterium asteroides DSM 20089
Olsenella scatoligenes SK9K4
Olsenella uli DSM 7084
Atopobium vaginae DSM 15829
Atopobium parvulum DSM 20469
Atopobium rimae ATCC 49626
Atopobium minutum DSM 20586
Atopobium fossor DSM 15642
Olsenella umbonata DSM 22620
Enorma massiliensis phI
Enorma timonensis GD5
Collinsella aerofaciens ATCC 25986
Collinsella intestinalis DSM 13280
Collinsella stercoris DSM 13279
Coriobacterium glomerans DSM 20642
Senegalimassilia anaerobia JC110
Cryptobacterium curtum DSM 15641
Enterorhabdus caecimuris B7
Adlercreutzia equolifaciens DSM 19450
Eggerthella lenta DSM 2243
Gordonibacter pamelaeae 7-10-1-b
Slackia piriformis YIT 12062
Slackia heliotrinireducens DSM 20476
Slackia exigua ATCC 700122
Denitrobacterium detoxificans DSM 21843
Amycolatopsis australiensis DSM 44671
Tessaracoccus bendigoensis DSM 12906
Thermoleophilum album ATCC 35263
Patulibacter americanus DSM 16676
Patulibacter minatonensis DSM 18081
Patulibacter medicamentivorans I11
Conexibacter woesei DSM 14684
Solirubrobacter pauli DSM 14954
Solirubrobacter soli DSM 22325
Rubrobacter xylanophilus DSM 9941
Rubrobacter aplysinae RV113
Rubrobacter radiotolerans DSM 5868
Acidothermus cellulolyticus 11B
Sporichthya polymorpha DSM 43042
Haloactinopolyspora alba DSM 45211
Jiangella alkaliphila DSM 45079
Jiangella alba DSM 45237
Jiangella muralis DSM 45357
Jiangella gansuensis DSM 44835
Quadrisphaera granulorum DSM 44889
Kineococcus radiotolerans SRS30216
Kineococcus xinjiangensis DSM 22857
Cellulomonas carbonis T26
Demequina aurantiaca NBRC 106265
Demequina oxidasica NBRC 106264
Demequina sediminicola NBRC 105855
Demequina flava NBRC 105854
Demequina globuliformis NBRC 106266
Lysinimicrobium pelophilum NBRC 109393
Lysinimicrobium rhizosphaerae NBRC 109397
Lysinimicrobium aestuarii NBRC 109392
Lysinimicrobium soli NBRC 109394
Lysinimicrobium subtropicum NBRC 109396
Lysinimicrobium gelatinilyticum NBRC 109390
Lysinimicrobium iriomotense NBRC 109399
Lysinimicrobium luteum NBRC 109395
Lysinimicrobium flavum NBRC 109391
Lysinimicrobium mangrovi DSM 24868
Lysinimicrobium mangrovi NBRC 105856
Demequina salsinemoris NBRC 105323

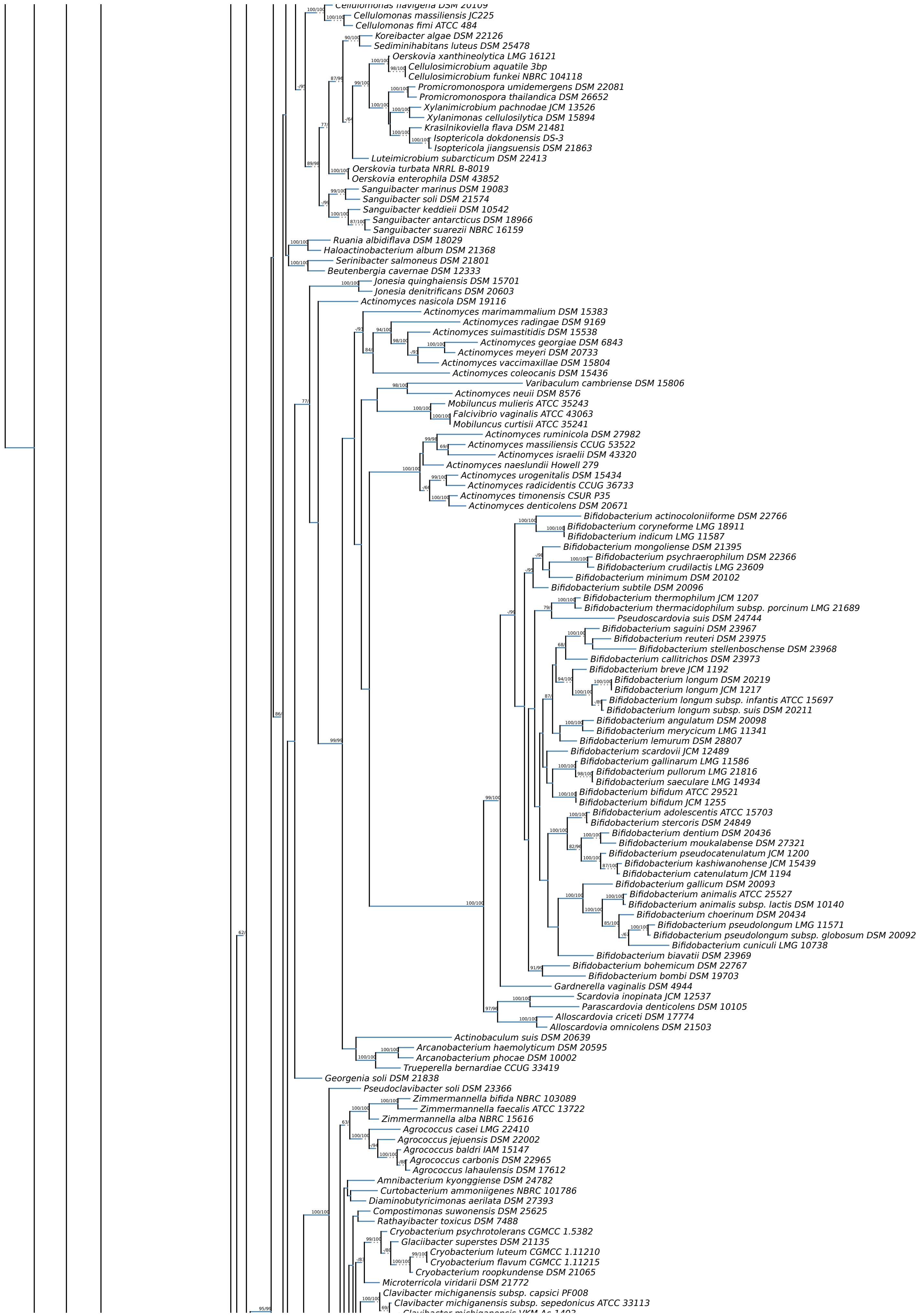
Cellulomonas flavigena DSM 20109
Cellulomonas massiliensis JC225
Cellulomonas fimi ATCC 484
Koreibacter algae DSM 22126
Sediminihabitans luteus DSM 25478
Oerskovia xanthineolytica LMG 16121
Cellulosimicrobium aquatile 3bp
Cellulosimicrobium funkei NBRC 104118
Promicromonospora umidemergens DSM 22081
Promicromonospora thailandica DSM 26652
Xylanimicrobium pachnodae JCM 13526
Xylanimonas cellulosilytica DSM 15894
Krasilnikoviella flava DSM 21481
Isoptericola dokdonensis DS-3
Isoptericola jiangsuensis DSM 21863
Luteimicrobium subarcticum DSM 22413
Oerskovia turbata NRRL B-8019
Oerskovia enterophila DSM 43852
Sanguibacter marinus DSM 19083
Sanguibacter soli DSM 21574
Sanguibacter keddieii DSM 10542
Sanguibacter antarcticus DSM 18966
Sanguibacter suarezii NBRC 16159
Ruania albidiflava DSM 18029
Haloactinobacterium album DSM 21368
Serinibacter salmoneus DSM 21801
Beutenbergia cavernae DSM 12333
Jonesia quinghaiensis DSM 15701
Jonesia denitrificans DSM 20603
Actinomyces nasicola DSM 19116
Actinomyces marimammalium DSM 15383
Actinomyces radingae DSM 9169
Actinomyces suimastitidis DSM 15538
Actinomyces georgiae DSM 6843
Actinomyces meyeri DSM 20733
Actinomyces vaccimaxillae DSM 15804
Actinomyces coleocanis DSM 15436
Varibaculum cambriense DSM 15806
Actinomyces neuii DSM 8576
Mobiluncus mulieris ATCC 35243
Falcivibrio vaginalis ATCC 43063
Mobiluncus curtisii ATCC 35241
Actinomyces ruminicola DSM 27982
Actinomyces massiliensis CCUG 53522
Actinomyces israelii DSM 43320
Actinomyces naeslundii Howell 279
Actinomyces urogenitalis DSM 15434
Actinomyces radicidentis CCUG 36733
Actinomyces timonensis CSUR P35
Actinomyces denticolens DSM 20671
Bifidobacterium actinocoloniiforme DSM 22766
Bifidobacterium coryneforme LMG 18911
Bifidobacterium indicum LMG 11587
Bifidobacterium mongoliense DSM 21395
Bifidobacterium psychraerophilum DSM 22366
Bifidobacterium crudilactis LMG 23609
Bifidobacterium minimum DSM 20102
Bifidobacterium subtile DSM 20096
Bifidobacterium thermophilum JCM 1207
Bifidobacterium thermacidophilum subsp. porcinum LMG 21689
Pseudoscardovia suis DSM 24744
Bifidobacterium saguini DSM 23967
Bifidobacterium reuteri DSM 23975
Bifidobacterium stellenboschense DSM 23968
Bifidobacterium callitrichos DSM 23973
Bifidobacterium breve JCM 1192
Bifidobacterium longum DSM 20219
Bifidobacterium longum JCM 1217
Bifidobacterium longum subsp. infantis ATCC 15697
Bifidobacterium longum subsp. suis DSM 20211
Bifidobacterium angulatum DSM 20098
Bifidobacterium merycicum LMG 11341
Bifidobacterium lemurum DSM 28807
Bifidobacterium scardovii JCM 12489
Bifidobacterium gallinarum LMG 11586
Bifidobacterium pullorum LMG 21816
Bifidobacterium saeculare LMG 14934
Bifidobacterium bifidum ATCC 29521
Bifidobacterium bifidum JCM 1255
Bifidobacterium adolescentis ATCC 15703
Bifidobacterium stercoris DSM 24849
Bifidobacterium dentium DSM 20436
Bifidobacterium moukalabense DSM 27321
Bifidobacterium pseudocatenulatum JCM 1200
Bifidobacterium kashiwanohense JCM 15439
Bifidobacterium catenulatum JCM 1194
Bifidobacterium gallicum DSM 20093
Bifidobacterium animalis ATCC 25527
Bifidobacterium animalis subsp. lactis DSM 10140
Bifidobacterium choerinum DSM 20434
Bifidobacterium pseudolongum LMG 11571
Bifidobacterium pseudolongum subsp. globosum DSM 20092
Bifidobacterium cuniculi LMG 10738
Bifidobacterium biavatii DSM 23969
Bifidobacterium bohemicum DSM 22767
Bifidobacterium bombi DSM 19703
Gardnerella vaginalis DSM 4944
Scardovia inopinata JCM 12537
Parascardovia denticolens DSM 10105
Alloscardovia criceti DSM 17774
Alloscardovia omnicolens DSM 21503
Actinobaculum suis DSM 20639
Arcanobacterium haemolyticum DSM 20595
Arcanobacterium phocae DSM 10002
Trueperella bernardiae CCUG 33419
Georgenia soli DSM 21838
Pseudoclavibacter soli DSM 23366
Zimmermannella bifida NBRC 103089
Zimmermannella faecalis ATCC 13722
Zimmermannella alba NBRC 15616
Agrococcus casei LMG 22410
Agrococcus jejuensis DSM 22002
Agrococcus baldri IAM 15147
Agrococcus carbonis DSM 22965
Agrococcus lahaulensis DSM 17612
Amnibacterium kyonggiense DSM 24782
Curtobacterium ammoniigenes NBRC 101786
Diaminobutyricimonas aerilata DSM 27393
Compostimonas suwonensis DSM 25625
Rathayibacter toxicus DSM 7488
Cryobacterium psychrotolerans CGMCC 1.5382
Glaciibacter superstes DSM 21135
Cryobacterium luteum CGMCC 1.11210
Cryobacterium flavum CGMCC 1.11215
Cryobacterium roopkundense DSM 21065
Microterricola viridarii DSM 21772
Clavibacter michiganensis subsp. capsici PF008
Clavibacter michiganensis subsp. sepedonicus ATCC 33113

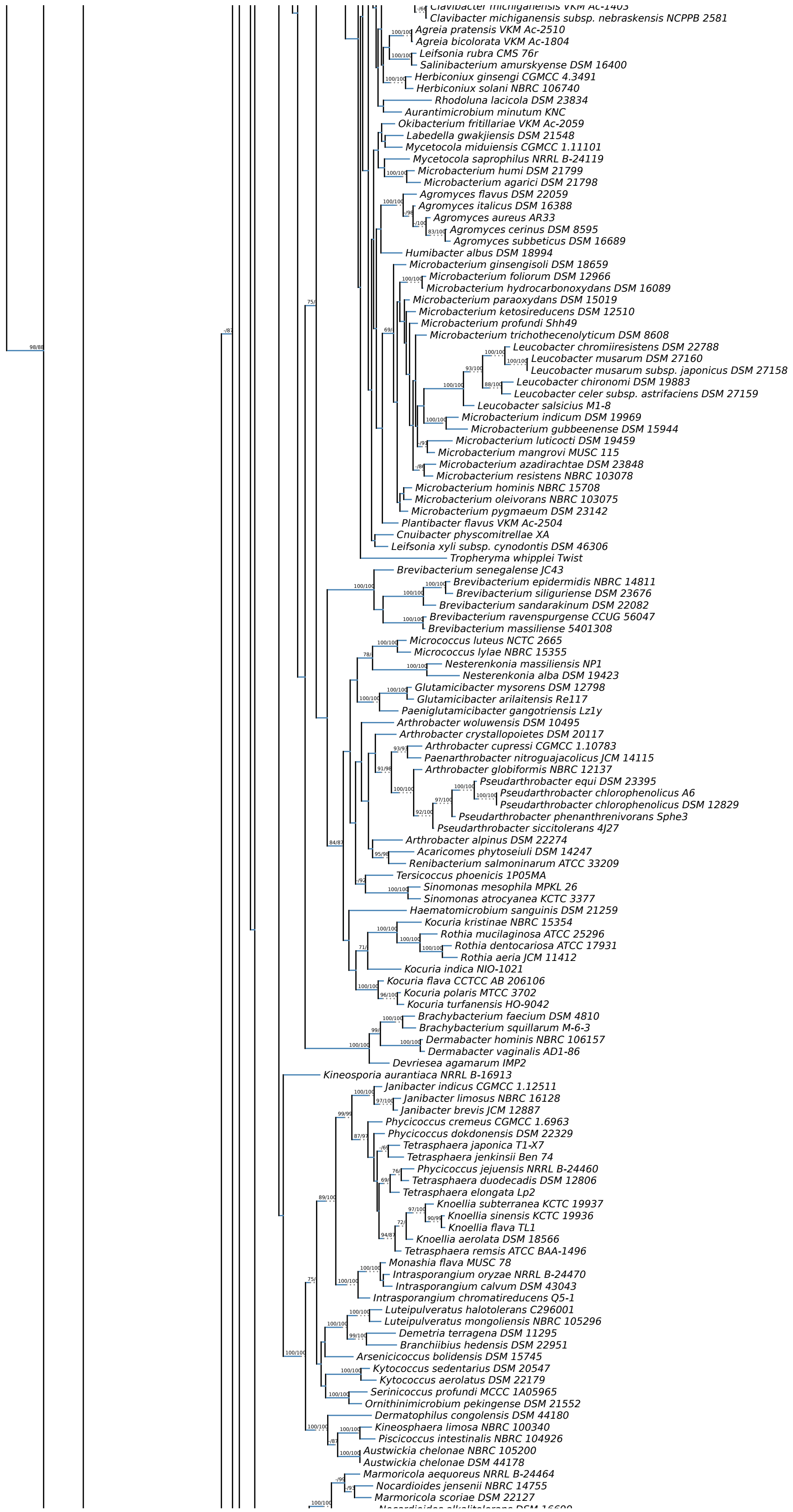

Clavibacter michiganensis VKM Ac-1403
Clavibacter michiganensis subsp. nebraskensis NCPPB 2581
Agreia pratensis VKM Ac-2510
Agreia bicolorata VKM Ac-1804
Leifsonia rubra CMS 76r
Salinibacterium amurskyense DSM 16400
Herbiconiux ginsengi CGMCC 4.3491
Herbiconiux solani NBRC 106740
Rhodoluna lacicola DSM 23834
Aurantimicrobium minutum KNC
Okibacterium fritillariae VKM Ac-2059
Labedella gwakjiensis DSM 21548
Mycetocola miduiensis CGMCC 1.11101
Mycetocola saprophilus NRRL B-24119
Microbacterium humi DSM 21799
Microbacterium agarici DSM 21798
Agromyces flavus DSM 22059
Agromyces italicus DSM 16388
Agromyces aureus AR33
Agromyces cerinus DSM 8595
Agromyces subbeticus DSM 16689
Humibacter albus DSM 18994
Microbacterium ginsengisoli DSM 18659
Microbacterium foliorum DSM 12966
Microbacterium hydrocarbonoxydans DSM 16089
Microbacterium paraoxydans DSM 15019
Microbacterium ketosireducens DSM 12510
Microbacterium profundi Shh49
Microbacterium trichothecenolyticum DSM 8608
Leucobacter chromiiresistens DSM 22788
Leucobacter musarum DSM 27160
Leucobacter musarum subsp. japonicus DSM 27158
Leucobacter chironomi DSM 19883
Leucobacter celer subsp. astrifaciens DSM 27159
Leucobacter salsicius M1-8
Microbacterium indicum DSM 19969
Microbacterium gubbeenense DSM 15944
Microbacterium luticocti DSM 19459
Microbacterium mangrovi MUSC 115
Microbacterium azadirachtae DSM 23848
Microbacterium resistens NBRC 103078
Microbacterium hominis NBRC 15708
Microbacterium oleivorans NBRC 103075
Microbacterium pygmaeum DSM 23142
Plantibacter flavus VKM Ac-2504
Cnuibacter physcomitrellae XA
Leifsonia xyli subsp. cynodontis DSM 46306
Tropheryma whipplei Twist
Brevibacterium senegalense JC43
Brevibacterium epidermidis NBRC 14811
Brevibacterium siliguriense DSM 23676
Brevibacterium sandarakinum DSM 22082
Brevibacterium ravenspurgense CCUG 56047
Brevibacterium massiliense 5401308
Micrococcus luteus NCTC 2665
Micrococcus lylae NBRC 15355
Nesterenkonia massiliensis NP1
Nesterenkonia alba DSM 19423
Glutamicibacter mysorens DSM 12798
Glutamicibacter arilaitensis Re117
Paeniglutamicibacter gangotriensis Lz1y
Arthrobacter woluwensis DSM 10495
Arthrobacter crystallopoietes DSM 20117
Arthrobacter cupressi CGMCC 1.10783
Paenarthrobacter nitroguajacolicus JCM 14115
Arthrobacter globiformis NBRC 12137
Pseudarthrobacter equi DSM 23395
Pseudarthrobacter chlorophenolicus A6
Pseudarthrobacter chlorophenolicus DSM 12829
Pseudarthrobacter phenanthrenivorans Sphe3
Pseudarthrobacter siccitolerans 4J27
Arthrobacter alpinus DSM 22274
Acaricomes phytoseiuli DSM 14247
Renibacterium salmoninarum ATCC 33209
Tersicoccus phoenicis 1P05MA
Sinomonas mesophila MPKL 26
Sinomonas atrocyanea KCTC 3377
Haematomicrobium sanguinis DSM 21259
Kocuria kristinae NBRC 15354
Rothia mucilaginosa ATCC 25296
Rothia dentocariosa ATCC 17931
Rothia aeria JCM 11412
Kocuria indica NIO-1021
Kocuria flava CCTCC AB 206106
Kocuria polaris MTCC 3702
Kocuria turfanensis HO-9042
Brachybacterium faecium DSM 4810
Brachybacterium squillarum M-6-3
Dermabacter hominis NBRC 106157
Dermabacter vaginalis AD1-86
Devriesea agamarum IMP2
Kineosporia aurantiaca NRRL B-16913
Janibacter indicus CGMCC 1.12511
Janibacter limosus NBRC 16128
Janibacter brevis JCM 12887
Phycicoccus cremeus CGMCC 1.6963
Phycicoccus dokdonensis DSM 22329
Tetrasphaera japonica T1-X7
Tetrasphaera jenkinsii Ben 74
Phycicoccus jejuensis NRRL B-24460
Tetrasphaera duodecadis DSM 12806
Tetrasphaera elongata Lp2
Knoellia subterranea KCTC 19937
Knoellia sinensis KCTC 19936
Knoellia flava TL1
Knoellia aerolata DSM 18566
Tetrasphaera remsis ATCC BAA-1496
Monashia flava MUSC 78
Intrasporangium oryzae NRRL B-24470
Intrasporangium calvum DSM 43043
Intrasporangium chromatireducens Q5-1
Luteipulveratus halotolerans C296001
Luteipulveratus mongoliensis NBRC 105296
Demetria terragena DSM 11295
Branchiibius hedensis DSM 22951
Arsenicicoccus bolidensis DSM 15745
Kytococcus sedentarius DSM 20547
Kytococcus aerolatus DSM 22179
Serinicoccus profundi MCCC 1A05965
Ornithinimicrobium pekingense DSM 21552
Dermatophilus congolensis DSM 44180
Kineosphaera limosa NBRC 100340
Piscicoccus intestinalis NBRC 104926
Austwickia chelonae NBRC 105200
Austwickia chelonae DSM 44178
Marmoricola aequoreus NRRL B-24464
Nocardioides jensenii NBRC 14755
Marmoricola scoriae DSM 22127

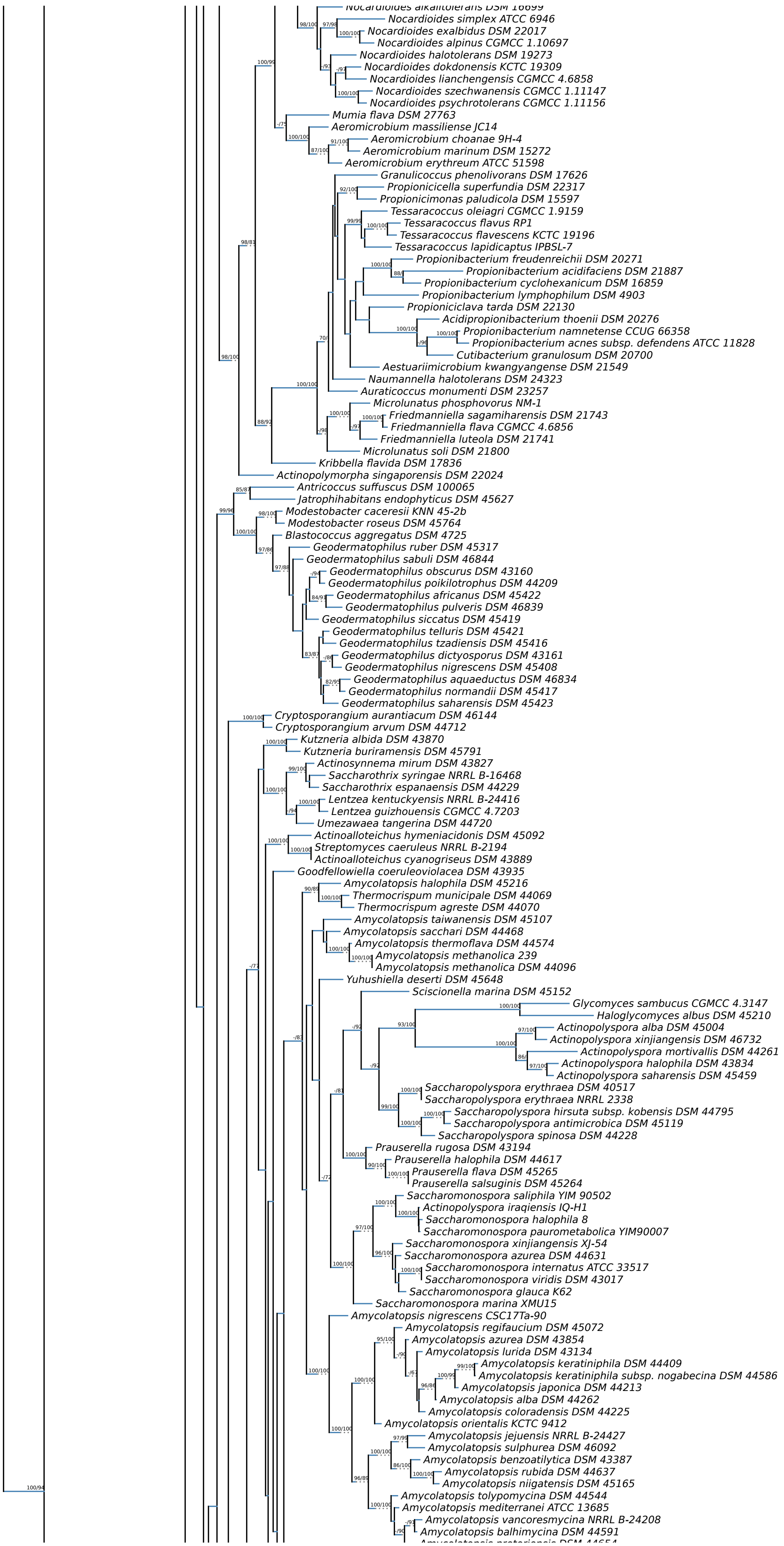

Nocardioides alkalitolerans DSM 16699
Nocardioides simplex ATCC 6946
Nocardioides exalbidus DSM 22017
Nocardioides alpinus CGMCC 1.10697
Nocardioides halotolerans DSM 19273
Nocardioides dokdonensis KCTC 19309
Nocardioides lianchengensis CGMCC 4.6858
Nocardioides szechwanensis CGMCC 1.11147
Nocardioides psychrotolerans CGMCC 1.11156
Mumia flava DSM 27763
Aeromicrobium massiliense JC14
Aeromicrobium choanae 9H-4
Aeromicrobium marinum DSM 15272
Aeromicrobium erythreum ATCC 51598
Granulicoccus phenolivorans DSM 17626
Propionicicella superfundia DSM 22317
Propionicimonas paludicola DSM 15597
Tessaracoccus oleiagri CGMCC 1.9159
Tessaracoccus flavus RP1
Tessaracoccus flavescens KCTC 19196
Tessaracoccus lapidicaptus IPBSL-7
Propionibacterium freudenreichii DSM 20271
Propionibacterium acidifaciens DSM 21887
Propionibacterium cyclohexanicum DSM 16859
Propionibacterium lymphophilum DSM 4903
Propioniciclava tarda DSM 22130
Acidipropionibacterium thoenii DSM 20276
Propionibacterium namnetense CCUG 66358
Propionibacterium acnes subsp. defendens ATCC 11828
Cutibacterium granulosum DSM 20700
Aestuariimicrobium kwangyangense DSM 21549
Naumannella halotolerans DSM 24323
Auraticoccus monumenti DSM 23257
Microlunatus phosphovorus NM-1
Friedmanniella sagamiharensis DSM 21743
Friedmanniella flava CGMCC 4.6856
Friedmanniella luteola DSM 21741
Microlunatus soli DSM 21800
Kribbella flavida DSM 17836
Actinopolymorpha singaporensis DSM 22024
Antricoccus suffuscus DSM 100065
Jatrophihabitans endophyticus DSM 45627
Modestobacter caceresii KNN 45-2b
Modestobacter roseus DSM 45764
Blastococcus aggregatus DSM 4725
Geodermatophilus ruber DSM 45317
Geodermatophilus sabuli DSM 46844
Geodermatophilus obscurus DSM 43160
Geodermatophilus poikilotrophus DSM 44209
Geodermatophilus africanus DSM 45422
Geodermatophilus pulveris DSM 46839
Geodermatophilus siccatus DSM 45419
Geodermatophilus telluris DSM 45421
Geodermatophilus tzadiensis DSM 45416
Geodermatophilus dictyosporus DSM 43161
Geodermatophilus nigrescens DSM 45408
Geodermatophilus aquaeductus DSM 46834
Geodermatophilus normandii DSM 45417
Geodermatophilus saharensis DSM 45423
Cryptosporangium aurantiacum DSM 46144
Cryptosporangium arvum DSM 44712
Kutzneria albida DSM 43870
Kutzneria buriramensis DSM 45791
Actinosynnema mirum DSM 43827
Saccharothrix syringae NRRL B-16468
Saccharothrix espanaensis DSM 44229
Lentzea kentuckyensis NRRL B-24416
Lentzea guizhouensis CGMCC 4.7203
Umezawaea tangerina DSM 44720
Actinoalloteichus hymeniacidonis DSM 45092
Streptomyces caeruleus NRRL B-2194
Actinoalloteichus cyanogriseus DSM 43889
Goodfellowiella coeruleoviolacea DSM 43935
Amycolatopsis halophila DSM 45216
Thermocrispum municipale DSM 44069
Thermocrispum agreste DSM 44070
Amycolatopsis taiwanensis DSM 45107
Amycolatopsis sacchari DSM 44468
Amycolatopsis thermoflava DSM 44574
Amycolatopsis methanolica 239
Amycolatopsis methanolica DSM 44096
Yuhushiella deserti DSM 45648
Sciscionella marina DSM 45152
Glycomyces sambucus CGMCC 4.3147
Haloglycomyces albus DSM 45210
Actinopolyspora alba DSM 45004
Actinopolyspora xinjiangensis DSM 46732
Actinopolyspora mortivallis DSM 44261
Actinopolyspora halophila DSM 43834
Actinopolyspora saharensis DSM 45459
Saccharopolyspora erythraea DSM 40517
Saccharopolyspora erythraea NRRL 2338
Saccharopolyspora hirsuta subsp. kobensis DSM 44795
Saccharopolyspora antimicrobica DSM 45119
Saccharopolyspora spinosa DSM 44228
Prauserella rugosa DSM 43194
Prauserella halophila DSM 44617
Prauserella flava DSM 45265
Prauserella salsuginis DSM 45264
Saccharomonospora saliphila YIM 90502
Actinopolyspora iraqiensis IQ-H1
Saccharomonospora halophila 8
Saccharomonospora paurometabolica YIM90007
Saccharomonospora xinjiangensis XJ-54
Saccharomonospora azurea DSM 44631
Saccharomonospora internatus ATCC 33517
Saccharomonospora viridis DSM 43017
Saccharomonospora glauca K62
Saccharomonospora marina XMU15
Amycolatopsis nigrescens CSC17Ta-90
Amycolatopsis regifaucium DSM 45072
Amycolatopsis azurea DSM 43854
Amycolatopsis lurida DSM 43134
Amycolatopsis keratiniphila DSM 44409
Amycolatopsis keratiniphila subsp. nogabecina DSM 44586
Amycolatopsis japonica DSM 44213
Amycolatopsis alba DSM 44262
Amycolatopsis coloradensis DSM 44225
Amycolatopsis orientalis KCTC 9412
Amycolatopsis jejuensis NRRL B-24427
Amycolatopsis sulphurea DSM 46092
Amycolatopsis benzoatilytica DSM 43387
Amycolatopsis rubida DSM 44637
Amycolatopsis niigatensis DSM 45165
Amycolatopsis tolypomycina DSM 44544
Amycolatopsis mediterranei ATCC 13685
Amycolatopsis vancoresmycina NRRL B-24208
Amycolatopsis balhimycina DSM 44591

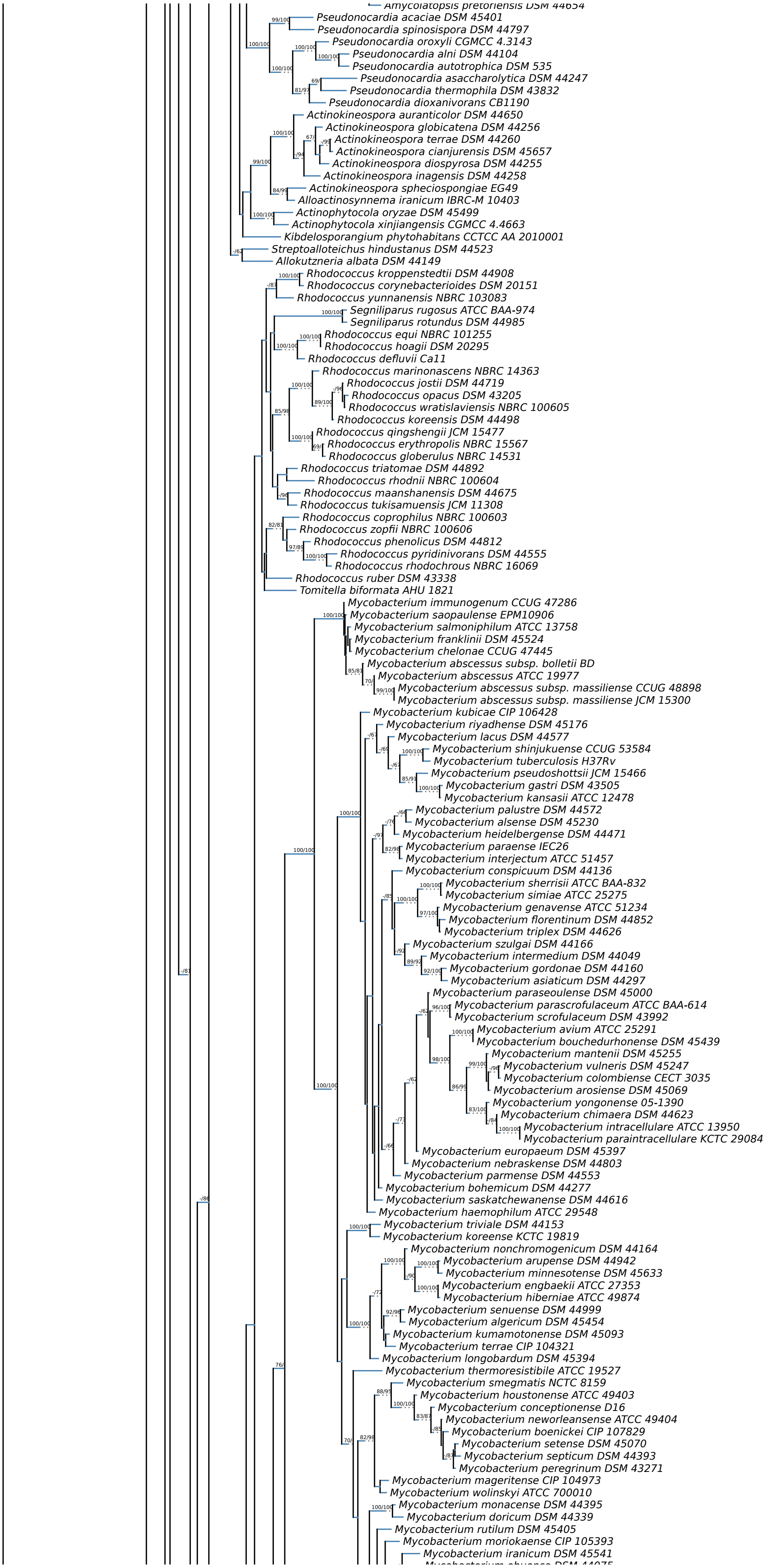
Pseudonocardia acaciae DSM 45401
Pseudonocardia spinosispora DSM 44797
Pseudonocardia oroxyli CGMCC 4.3143
Pseudonocardia alni DSM 44104
Pseudonocardia autotrophica DSM 535
Pseudonocardia asaccharolytica DSM 44247
Pseudonocardia thermophila DSM 43832
Pseudonocardia dioxanivorans CB1190
Actinokineospora auranticolor DSM 44650
Actinokineospora globicatena DSM 44256
Actinokineospora terrae DSM 44260
Actinokineospora cianjurensis DSM 45657
Actinokineospora diospyrosa DSM 44255
Actinokineospora inagensis DSM 44258
Actinokineospora spheciospongiae EG49
Alloactinosynnema iranicum IBRC-M 10403
Actinophytocola oryzae DSM 45499
Actinophytocola xinjiangensis CGMCC 4.4663
Kibdelosporangium phytohabitans CCTCC AA 2010001
Streptoalloteichus hindustanus DSM 44523
Allokutzneria albata DSM 44149
Rhodococcus kroppenstedtii DSM 44908
Rhodococcus corynebacterioides DSM 20151
Rhodococcus yunnanensis NBRC 103083
Segniliparus rugosus ATCC BAA-974
Segniliparus rotundus DSM 44985
Rhodococcus equi NBRC 101255
Rhodococcus hoagii DSM 20295
Rhodococcus defluvii Ca11
Rhodococcus marinonascens NBRC 14363
Rhodococcus jostii DSM 44719
Rhodococcus opacus DSM 43205
Rhodococcus wratislaviensis NBRC 100605
Rhodococcus koreensis DSM 44498
Rhodococcus qingshengii JCM 15477
Rhodococcus erythropolis NBRC 15567
Rhodococcus globerulus NBRC 14531
Rhodococcus triatomae DSM 44892
Rhodococcus rhodnii NBRC 100604
Rhodococcus maanshanensis DSM 44675
Rhodococcus tukisamuensis JCM 11308
Rhodococcus coprophilus NBRC 100603
Rhodococcus zopfii NBRC 100606
Rhodococcus phenolicus DSM 44812
Rhodococcus pyridinivorans DSM 44555
Rhodococcus rhodochrous NBRC 16069
Rhodococcus ruber DSM 43338
Tomitella biformata AHU 1821
Mycobacterium immunogenum CCUG 47286
Mycobacterium saopaulense EPM10906
Mycobacterium salmoniphilum ATCC 13758
Mycobacterium franklinii DSM 45524
Mycobacterium chelonae CCUG 47445
Mycobacterium abscessus subsp. bolletii BD
Mycobacterium abscessus ATCC 19977
Mycobacterium abscessus subsp. massiliense CCUG 48898
Mycobacterium abscessus subsp. massiliense JCM 15300
Mycobacterium kubicae CIP 106428
Mycobacterium riyadhense DSM 45176
Mycobacterium lacus DSM 44577
Mycobacterium shinjukuense CCUG 53584
Mycobacterium tuberculosis H37Rv
Mycobacterium pseudoshottsii JCM 15466
Mycobacterium gastri DSM 43505
Mycobacterium kansasii ATCC 12478
Mycobacterium palustre DSM 44572
Mycobacterium alsense DSM 45230
Mycobacterium heidelbergense DSM 44471
Mycobacterium paraense IEC26
Mycobacterium interjectum ATCC 51457
Mycobacterium conspicuum DSM 44136
Mycobacterium sherrisii ATCC BAA-832
Mycobacterium simiae ATCC 25275
Mycobacterium genavense ATCC 51234
Mycobacterium florentinum DSM 44852
Mycobacterium triplex DSM 44626
Mycobacterium szulgai DSM 44166
Mycobacterium intermedium DSM 44049
Mycobacterium gordonae DSM 44160
Mycobacterium asiaticum DSM 44297
Mycobacterium paraseoulense DSM 45000
Mycobacterium parascrofulaceum ATCC BAA-614
Mycobacterium scrofulaceum DSM 43992
Mycobacterium avium ATCC 25291
Mycobacterium bouchedurhonense DSM 45439
Mycobacterium mantenii DSM 45255
Mycobacterium vulneris DSM 45247
Mycobacterium colombiense CECT 3035
Mycobacterium arosiense DSM 45069
Mycobacterium yongonense 05-1390
Mycobacterium chimaera DSM 44623
Mycobacterium intracellulare ATCC 13950
Mycobacterium paraintracellulare KCTC 29084
Mycobacterium europaeum DSM 45397
Mycobacterium nebraskense DSM 44803
Mycobacterium parmense DSM 44553
Mycobacterium bohemicum DSM 44277
Mycobacterium saskatchewanense DSM 44616
Mycobacterium haemophilum ATCC 29548
Mycobacterium triviale DSM 44153
Mycobacterium koreense KCTC 19819
Mycobacterium nonchromogenicum DSM 44164
Mycobacterium arupense DSM 44942
Mycobacterium minnesotense DSM 45633
Mycobacterium engbaekii ATCC 27353
Mycobacterium hiberniae ATCC 49874
Mycobacterium senuense DSM 44999
Mycobacterium algericum DSM 45454
Mycobacterium kumamotonense DSM 45093
Mycobacterium terrae CIP 104321
Mycobacterium longobardum DSM 45394
Mycobacterium thermoresistibile ATCC 19527
Mycobacterium smegmatis NCTC 8159
Mycobacterium houstonense ATCC 49403
Mycobacterium conceptionense D16
Mycobacterium neworleansense ATCC 49404
Mycobacterium boenickei CIP 107829
Mycobacterium setense DSM 45070
Mycobacterium septicum DSM 44393
Mycobacterium peregrinum DSM 43271
Mycobacterium mageritense CIP 104973
Mycobacterium wolinskyi ATCC 700010
Mycobacterium monacense DSM 44395
Mycobacterium doricum DSM 44339
Mycobacterium rutilum DSM 45405
Mycobacterium moriokaense CIP 105393
Mycobacterium iranicum DSM 45541

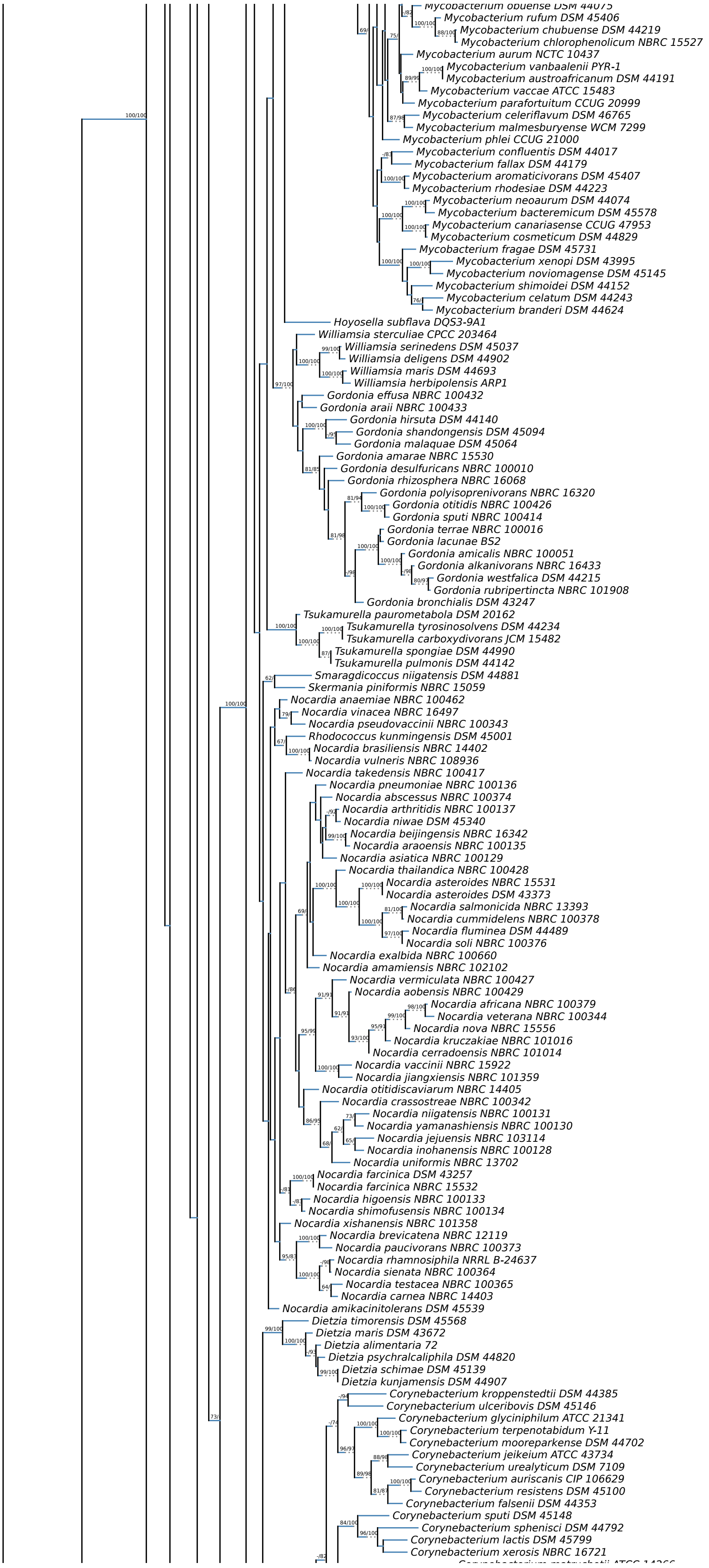
Mycobacterium obuense DSM 44075
Mycobacterium rufum DSM 45406
Mycobacterium chubuense DSM 44219
Mycobacterium chlorophenolicum NBRC 15527
Mycobacterium aurum NCTC 10437
Mycobacterium vanbaalenii PYR-1
Mycobacterium austroafricanum DSM 44191
Mycobacterium vaccae ATCC 15483
Mycobacterium parafortuitum CCUG 20999
Mycobacterium celeriflavum DSM 46765
Mycobacterium malmesburyense WCM 7299
Mycobacterium phlei CCUG 21000
Mycobacterium confluentis DSM 44017
Mycobacterium fallax DSM 44179
Mycobacterium aromaticivorans DSM 45407
Mycobacterium rhodesiae DSM 44223
Mycobacterium neoaurum DSM 44074
Mycobacterium bacteremicum DSM 45578
Mycobacterium canariasense CCUG 47953
Mycobacterium cosmeticum DSM 44829
Mycobacterium fragae DSM 45731
Mycobacterium xenopi DSM 43995
Mycobacterium noviomagense DSM 45145
Mycobacterium shimoidei DSM 44152
Mycobacterium celatum DSM 44243
Mycobacterium branderi DSM 44624
Hoyosella subflava DQS3-9A1
Williamsia sterculiae CPCC 203464
Williamsia serinedens DSM 45037
Williamsia deligens DSM 44902
Williamsia maris DSM 44693
Williamsia herbipolensis ARP1
Gordonia effusa NBRC 100432
Gordonia araii NBRC 100433
Gordonia hirsuta DSM 44140
Gordonia shandongensis DSM 45094
Gordonia malaquae DSM 45064
Gordonia amarae NBRC 15530
Gordonia desulfuricans NBRC 100010
Gordonia rhizosphera NBRC 16068
Gordonia polyisoprenivorans NBRC 16320
Gordonia otitidis NBRC 100426
Gordonia sputi NBRC 100414
Gordonia terrae NBRC 100016
Gordonia lacunae BS2
Gordonia amicalis NBRC 100051
Gordonia alkanivorans NBRC 16433
Gordonia westfalica DSM 44215
Gordonia rubripertincta NBRC 101908
Gordonia bronchialis DSM 43247
Tsukamurella paurometabola DSM 20162
Tsukamurella tyrosinosolvens DSM 44234
Tsukamurella carboxydivorans JCM 15482
Tsukamurella spongiae DSM 44990
Tsukamurella pulmonis DSM 44142
Smaragdicoccus niigatensis DSM 44881
Skermania piniformis NBRC 15059
Nocardia anaemiae NBRC 100462
Nocardia vinacea NBRC 16497
Nocardia pseudovaccinii NBRC 100343
Rhodococcus kunmingensis DSM 45001
Nocardia brasiliensis NBRC 14402
Nocardia vulneris NBRC 108936
Nocardia takedensis NBRC 100417
Nocardia pneumoniae NBRC 100136
Nocardia abscessus NBRC 100374
Nocardia arthritidis NBRC 100137
Nocardia niwae DSM 45340
Nocardia beijingensis NBRC 16342
Nocardia araoensis NBRC 100135
Nocardia asiatica NBRC 100129
Nocardia thailandica NBRC 100428
Nocardia asteroides NBRC 15531
Nocardia asteroides DSM 43373
Nocardia salmonicida NBRC 13393
Nocardia cummidelens NBRC 100378
Nocardia fluminea DSM 44489
Nocardia soli NBRC 100376
Nocardia exalbida NBRC 100660
Nocardia amamiensis NBRC 102102
Nocardia vermiculata NBRC 100427
Nocardia aobensis NBRC 100429
Nocardia africana NBRC 100379
Nocardia veterana NBRC 100344
Nocardia nova NBRC 15556
Nocardia kruczakiae NBRC 101016
Nocardia cerradoensis NBRC 101014
Nocardia vaccinii NBRC 15922
Nocardia jiangxiensis NBRC 101359
Nocardia otitidiscaviarum NBRC 14405
Nocardia crassostreae NBRC 100342
Nocardia niigatensis NBRC 100131
Nocardia yamanashiensis NBRC 100130
Nocardia jejuensis NBRC 103114
Nocardia inohanensis NBRC 100128
Nocardia uniformis NBRC 13702
Nocardia farcinica DSM 43257
Nocardia farcinica NBRC 15532
Nocardia higoensis NBRC 100133
Nocardia shimofusensis NBRC 100134
Nocardia xishanensis NBRC 101358
Nocardia brevicatena NBRC 12119
Nocardia paucivorans NBRC 100373
Nocardia rhamnosiphila NRRL B-24637
Nocardia sienata NBRC 100364
Nocardia testacea NBRC 100365
Nocardia carnea NBRC 14403
Nocardia amikacinitolerans DSM 45539
Dietzia timorensis DSM 45568
Dietzia maris DSM 43672
Dietzia alimentaria 72
Dietzia psychralcaliphila DSM 44820
Dietzia schimae DSM 45139
Dietzia kunjamensis DSM 44907
Corynebacterium kroppenstedtii DSM 44385
Corynebacterium ulceribovis DSM 45146
Corynebacterium glyciniphilum ATCC 21341
Corynebacterium terpenotabidum Y-11
Corynebacterium mooreparkense DSM 44702
Corynebacterium jeikeium ATCC 43734
Corynebacterium urealyticum DSM 7109
Corynebacterium auriscanis CIP 106629
Corynebacterium resistens DSM 45100
Corynebacterium falsenii DSM 44353
Corynebacterium sputi DSM 45148
Corynebacterium sphenisci DSM 44792
Corynebacterium lactis DSM 45799
Corynebacterium xerosis NBRC 16721

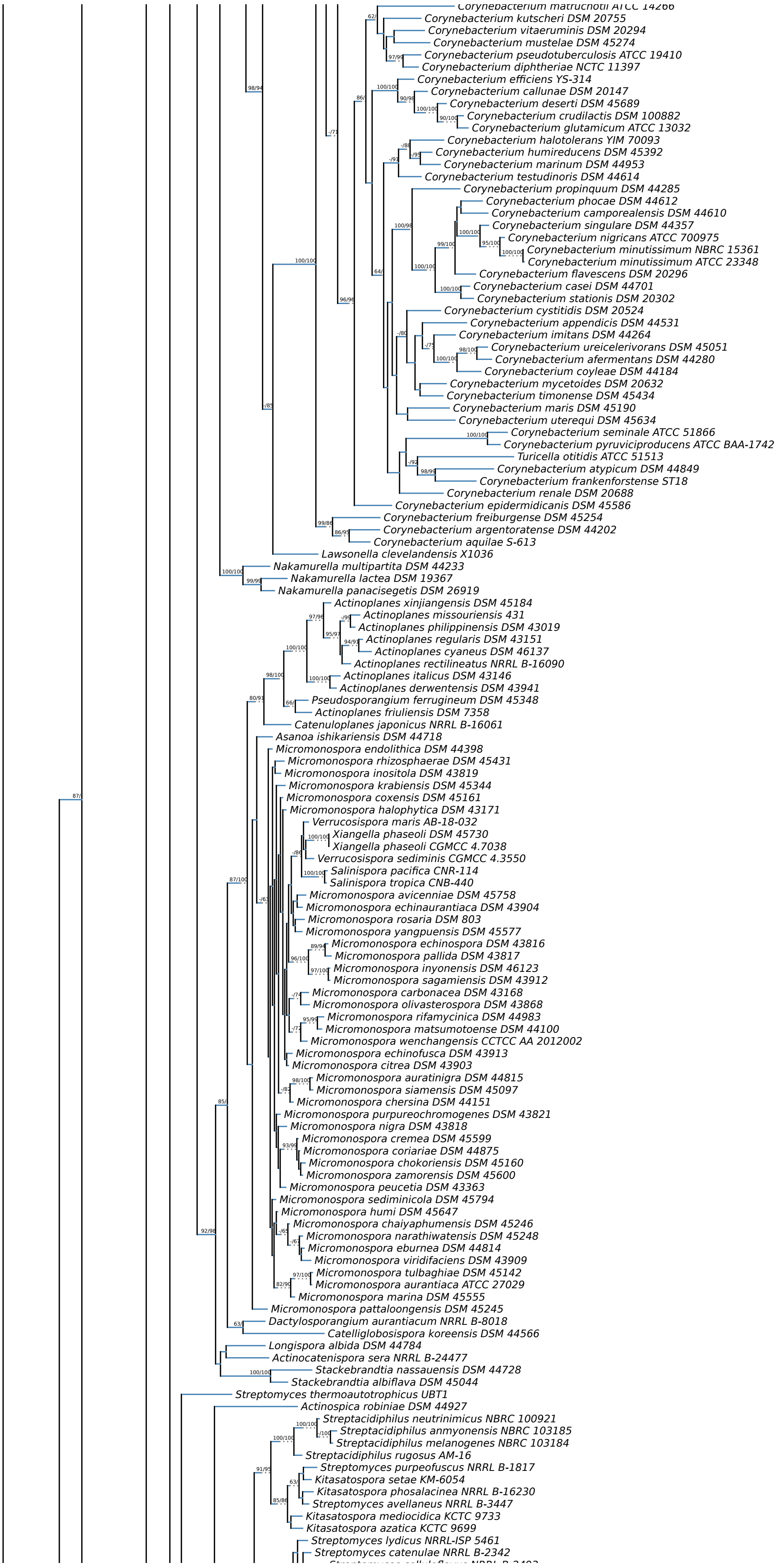

Corynebacterium matruchotii ATCC 14266
Corynebacterium kutscheri DSM 20755
Corynebacterium vitaeruminis DSM 20294
Corynebacterium mustelae DSM 45274
Corynebacterium pseudotuberculosis ATCC 19410
Corynebacterium diphtheriae NCTC 11397
Corynebacterium efficiens YS-314
Corynebacterium callunae DSM 20147
Corynebacterium deserti DSM 45689
Corynebacterium crudilactis DSM 100882
Corynebacterium glutamicum ATCC 13032
Corynebacterium halotolerans YIM 70093
Corynebacterium humireducens DSM 45392
Corynebacterium marinum DSM 44953
Corynebacterium testudinoris DSM 44614
Corynebacterium propinquum DSM 44285
Corynebacterium phocae DSM 44612
Corynebacterium camporealensis DSM 44610
Corynebacterium singulare DSM 44357
Corynebacterium nigricans ATCC 700975
Corynebacterium minutissimum NBRC 15361
Corynebacterium minutissimum ATCC 23348
Corynebacterium flavescens DSM 20296
Corynebacterium casei DSM 44701
Corynebacterium stationis DSM 20302
Corynebacterium cystitidis DSM 20524
Corynebacterium appendicis DSM 44531
Corynebacterium imitans DSM 44264
Corynebacterium ureicelerivorans DSM 45051
Corynebacterium afermentans DSM 44280
Corynebacterium coyleae DSM 44184
Corynebacterium mycetoides DSM 20632
Corynebacterium timonense DSM 45434
Corynebacterium maris DSM 45190
Corynebacterium uterequi DSM 45634
Corynebacterium seminale ATCC 51866
Corynebacterium pyruviciproducens ATCC BAA-1742
Turicella otitidis ATCC 51513
Corynebacterium atypicum DSM 44849
Corynebacterium frankenforstense ST18
Corynebacterium renale DSM 20688
Corynebacterium epidermidicanis DSM 45586
Corynebacterium freiburgense DSM 45254
Corynebacterium argentoratense DSM 44202
Corynebacterium aquilae S-613
Lawsonella clevelandensis X1036
Nakamurella multipartita DSM 44233
Nakamurella lactea DSM 19367
Nakamurella panacisegetis DSM 26919
Actinoplanes xinjiangensis DSM 45184
Actinoplanes missouriensis 431
Actinoplanes philippinensis DSM 43019
Actinoplanes regularis DSM 43151
Actinoplanes cyaneus DSM 46137
Actinoplanes rectilineatus NRRL B-16090
Actinoplanes italicus DSM 43146
Actinoplanes derwentensis DSM 43941
Pseudosporangium ferrugineum DSM 45348
Actinoplanes friuliensis DSM 7358
Catenuloplanes japonicus NRRL B-16061
Asanoa ishikariensis DSM 44718
Micromonospora endolithica DSM 44398
Micromonospora rhizosphaerae DSM 45431
Micromonospora inositola DSM 43819
Micromonospora krabiensis DSM 45344
Micromonospora coxensis DSM 45161
Micromonospora halophytica DSM 43171
Verrucosispora maris AB-18-032
Xiangella phaseoli DSM 45730
Xiangella phaseoli CGMCC 4.7038
Verrucosispora sediminis CGMCC 4.3550
Salinispora pacifica CNR-114
Salinispora tropica CNB-440
Micromonospora avicenniae DSM 45758
Micromonospora echinaurantiaca DSM 43904
Micromonospora rosaria DSM 803
Micromonospora yangpuensis DSM 45577
Micromonospora echinospora DSM 43816
Micromonospora pallida DSM 43817
Micromonospora inyonensis DSM 46123
Micromonospora sagamiensis DSM 43912
Micromonospora carbonacea DSM 43168
Micromonospora olivasterospora DSM 43868
Micromonospora rifamycinica DSM 44983
Micromonospora matsumotoense DSM 44100
Micromonospora wenchangensis CCTCC AA 2012002
Micromonospora echinofusca DSM 43913
Micromonospora citrea DSM 43903
Micromonospora auratinigra DSM 44815
Micromonospora siamensis DSM 45097
Micromonospora chersina DSM 44151
Micromonospora purpureochromogenes DSM 43821
Micromonospora nigra DSM 43818
Micromonospora cremea DSM 45599
Micromonospora coriariae DSM 44875
Micromonospora chokoriensis DSM 45160
Micromonospora zamorensis DSM 45600
Micromonospora peucetia DSM 43363
Micromonospora sediminicola DSM 45794
Micromonospora humi DSM 45647
Micromonospora chaiyaphumensis DSM 45246
Micromonospora narathiwatensis DSM 45248
Micromonospora eburnea DSM 44814
Micromonospora viridifaciens DSM 43909
Micromonospora tulbaghiae DSM 45142
Micromonospora aurantiaca ATCC 27029
Micromonospora marina DSM 45555
Micromonospora pattaloongensis DSM 45245
Dactylosporangium aurantiacum NRRL B-8018
Catelliglobosispora koreensis DSM 44566
Longispora albida DSM 44784
Actinocatenispora sera NRRL B-24477
Stackebrandtia nassauensis DSM 44728
Stackebrandtia albiflava DSM 45044
Streptomyces thermoautotrophicus UBT1
Actinospica robiniae DSM 44927
Streptacidiphilus neutrinimicus NBRC 100921
Streptacidiphilus anmyonensis NBRC 103185
Streptacidiphilus melanogenes NBRC 103184
Streptacidiphilus rugosus AM-16
Streptomyces purpeofuscus NRRL B-1817
Kitasatospora setae KM-6054
Kitasatospora phosalacinea NRRL B-16230
Streptomyces avellaneus NRRL B-3447
Kitasatospora mediocidica KCTC 9733
Kitasatospora azatica KCTC 9699
Streptomyces lydicus NRRL-ISP 5461
Streptomyces catenulae NRRL B-2342

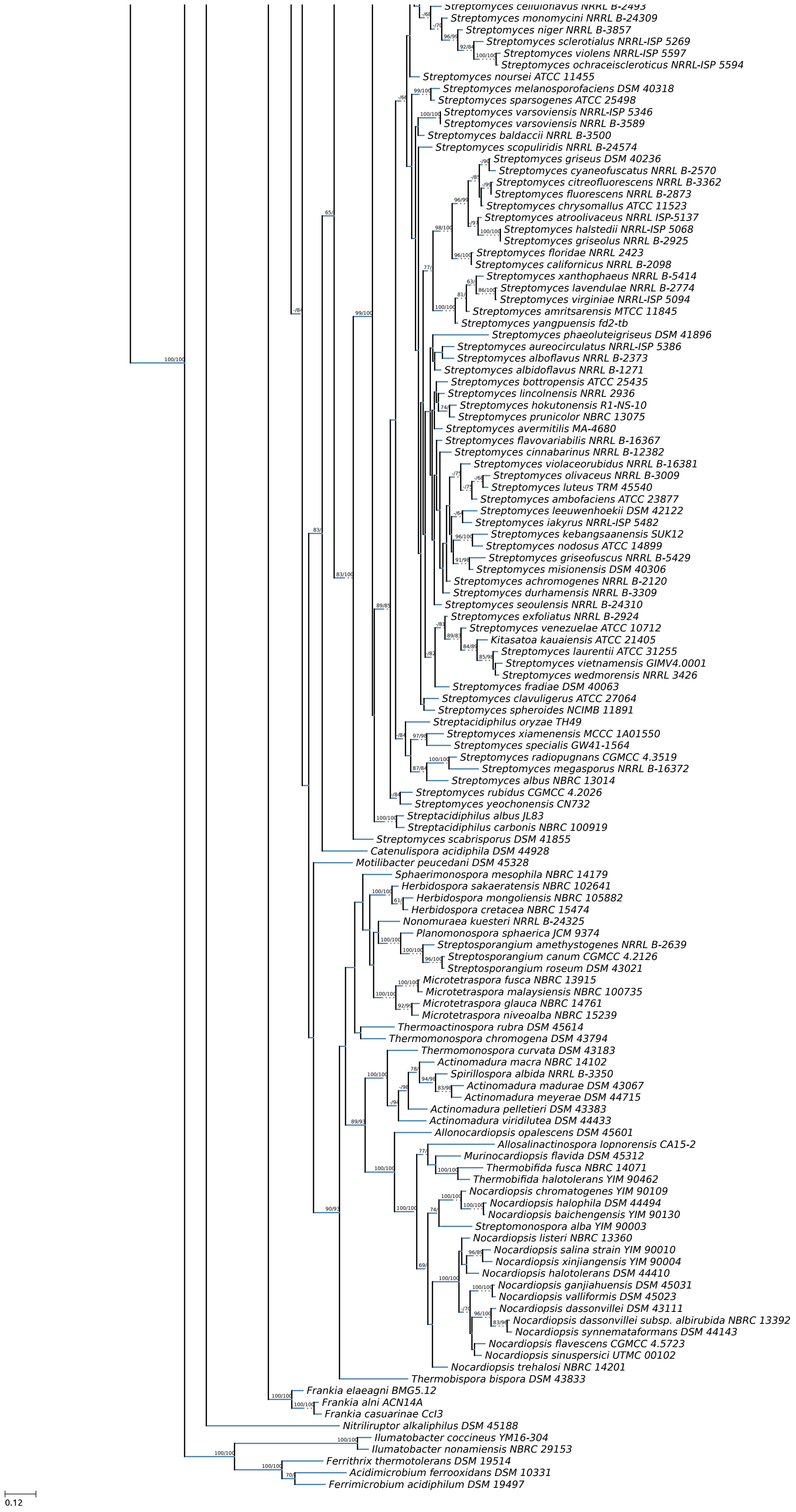

Streptomyces monomycini NRRL B-24309
Streptomyces niger NRRL B-3857
Streptomyces sclerotialus NRRL-ISP 5269
Streptomyces violens NRRL-ISP 5597
Streptomyces ochraceiscleroticus NRRL-ISP 5594
Streptomyces noursei ATCC 11455
Streptomyces melanosporofaciens DSM 40318
Streptomyces sparsogenes ATCC 25498
Streptomyces varsoviensis NRRL-ISP 5346
Streptomyces varsoviensis NRRL B-3589
Streptomyces baldaccii NRRL B-3500
Streptomyces scopuliridis NRRL B-24574
Streptomyces griseus DSM 40236
Streptomyces cyaneofuscatus NRRL B-2570
Streptomyces citreofluorescens NRRL B-3362
Streptomyces fluorescens NRRL B-2873
Streptomyces chrysomallus ATCC 11523
Streptomyces atroolivaceus NRRL ISP-5137
Streptomyces halstedii NRRL-ISP 5068
Streptomyces griseolus NRRL B-2925
Streptomyces floridae NRRL 2423
Streptomyces californicus NRRL B-2098
Streptomyces xanthophaeus NRRL B-5414
Streptomyces lavendulae NRRL B-2774
Streptomyces virginiae NRRL-ISP 5094
Streptomyces amritsarensis MTCC 11845
Streptomyces yangpuensis fd2-tb
Streptomyces phaeoluteigriseus DSM 41896
Streptomyces aureocirculatus NRRL-ISP 5386
Streptomyces alboflavus NRRL B-2373
Streptomyces albidoflavus NRRL B-1271
Streptomyces bottropensis ATCC 25435
Streptomyces lincolnensis NRRL 2936
Streptomyces hokutonensis R1-NS-10
Streptomyces prunicolor NBRC 13075
Streptomyces avermitilis MA-4680
Streptomyces flavovariabilis NRRL B-16367
Streptomyces cinnabarinus NRRL B-12382
Streptomyces violaceorubidus NRRL B-16381
Streptomyces olivaceus NRRL B-3009
Streptomyces luteus TRM 45540
Streptomyces ambofaciens ATCC 23877
Streptomyces leeuwenhoekii DSM 42122
Streptomyces iakyrus NRRL-ISP 5482
Streptomyces kebangsaanensis SUK12
Streptomyces nodosus ATCC 14899
Streptomyces griseofuscus NRRL B-5429
Streptomyces misionensis DSM 40306
Streptomyces achromogenes NRRL B-2120
Streptomyces durhamensis NRRL B-3309
Streptomyces seoulensis NRRL B-24310
Streptomyces exfoliatus NRRL B-2924
Streptomyces venezuelae ATCC 10712
Kitasatoa kauaiensis ATCC 21405
Streptomyces laurentii ATCC 31255
Streptomyces vietnamensis GIMV4.0001
Streptomyces wedmorensis NRRL 3426
Streptomyces fradiae DSM 40063
Streptomyces clavuligerus ATCC 27064
Streptomyces spheroides NCIMB 11891
Streptacidiphilus oryzae TH49
Streptomyces xiamenensis MCCC 1A01550
Streptomyces specialis GW41-1564
Streptomyces radiopugnans CGMCC 4.3519
Streptomyces megasporus NRRL B-16372
Streptomyces albus NBRC 13014
Streptomyces rubidus CGMCC 4.2026
Streptomyces yeochonensis CN732
Streptacidiphilus albus JL83
Streptacidiphilus carbonis NBRC 100919
Streptomyces scabrisporus DSM 41855
Catenulispora acidiphila DSM 44928
Motilibacter peucedani DSM 45328
Sphaerimonospora mesophila NBRC 14179
Herbidospora sakaeratensis NBRC 102641
Herbidospora mongoliensis NBRC 105882
Herbidospora cretacea NBRC 15474
Nonomuraea kuesteri NRRL B-24325
Planomonospora sphaerica JCM 9374
Streptosporangium amethystogenes NRRL B-2639
Streptosporangium canum CGMCC 4.2126
Streptosporangium roseum DSM 43021
Microtetraspora fusca NBRC 13915
Microtetraspora malaysiensis NBRC 100735
Microtetraspora glauca NBRC 14761
Microtetraspora niveoalba NBRC 15239
Thermoactinospora rubra DSM 45614
Thermomonospora chromogena DSM 43794
Thermomonospora curvata DSM 43183
Actinomadura macra NBRC 14102
Spirillospora albida NRRL B-3350
Actinomadura madurae DSM 43067
Actinomadura meyerae DSM 44715
Actinomadura pelletieri DSM 43383
Actinomadura viridilutea DSM 44433
Allonocardiopsis opalescens DSM 45601
Allosalinactinospora lopnorensis CA15-2
Murinocardiopsis flavida DSM 45312
Thermobifida fusca NBRC 14071
Thermobifida halotolerans YIM 90462
Nocardiopsis chromatogenes YIM 90109
Nocardiopsis halophila DSM 44494
Nocardiopsis baichengensis YIM 90130
Streptomonospora alba YIM 90003
Nocardiopsis listeri NBRC 13360
Nocardiopsis salina strain YIM 90010
Nocardiopsis xinjiangensis YIM 90004
Nocardiopsis halotolerans DSM 44410
Nocardiopsis ganjiahuensis DSM 45031
Nocardiopsis valliformis DSM 45023
Nocardiopsis dassonvillei DSM 43111
Nocardiopsis dassonvillei subsp. albirubida NBRC 13392
Nocardiopsis synnemataformans DSM 44143
Nocardiopsis flavescens CGMCC 4.5723
Nocardiopsis sinuspersici UTMC 00102
Nocardiopsis trehalosi NBRC 14201
Thermobispora bispora DSM 43833
Frankia elaeagni BMG5.12
Frankia alni ACN14A
Frankia casuarinae CcI3
Nitriliruptor alkaliphilus DSM 45188
Ilumatobacter coccineus YM16-304
Ilumatobacter nonamiensis NBRC 29153
Ferrithrix thermotolerans DSM 19514
Acidimicrobium ferrooxidans DSM 10331
Ferrimicrobium acidiphilum DSM 19497
0.12

Figure 4: 23S rRNA gene ML and MP tree. Dotted parts of branches are filled in to allow proper placement of bootstrap values and are not part of the actual branch length.

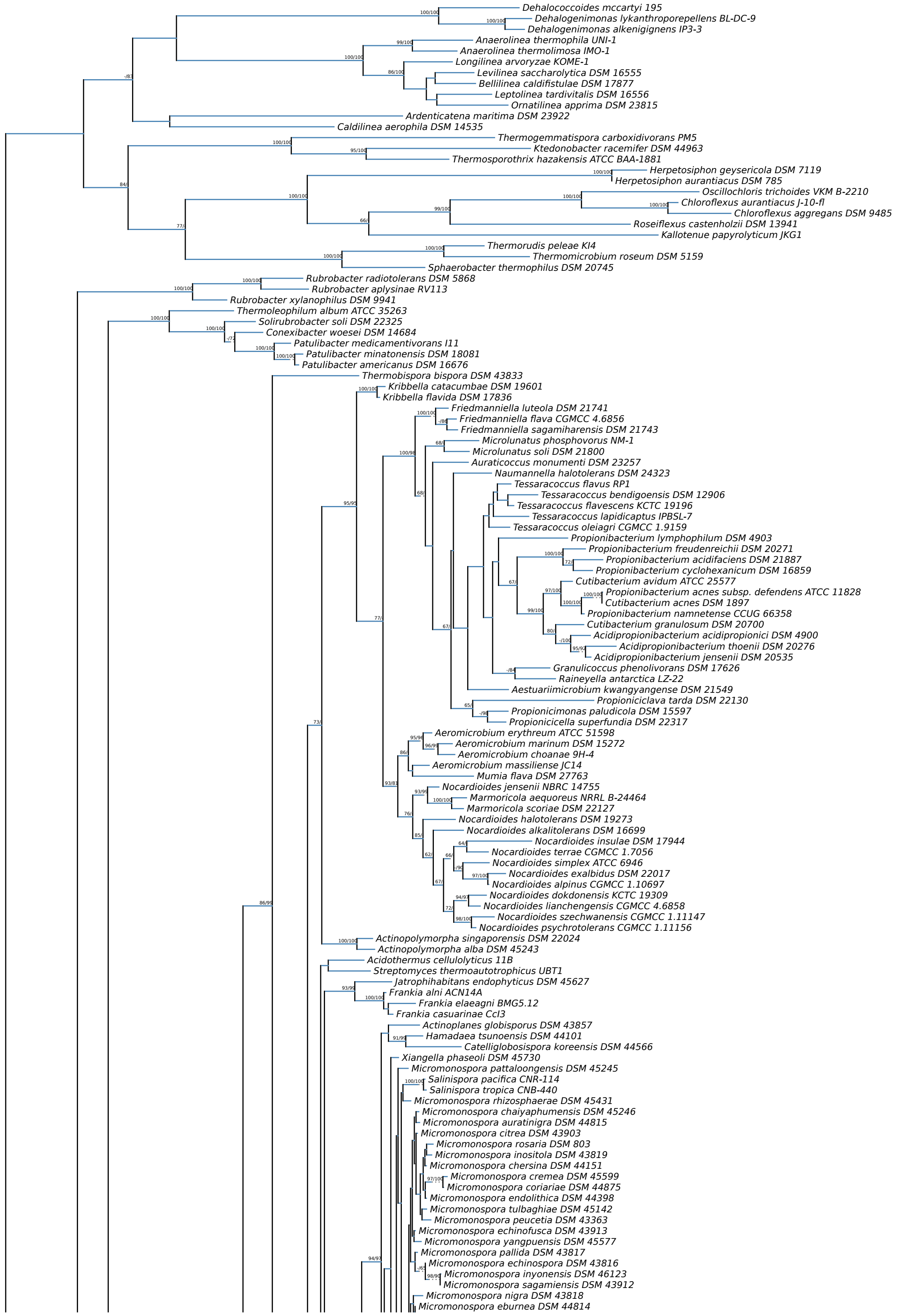
Dehalococcoides mccartyi 195
Dehalogenimonas lykanthroporepellens BL-DC-9
Dehalogenimonas alkenigignens IP3-3
Anaerolinea thermophila UNI-1
Anaerolinea thermolimosa IMO-1
Longilinea arvoryzae KOME-1
Levilinea saccharolytica DSM 16555
Bellilinea caldifistulae DSM 17877
Leptolinea tardivitalis DSM 16556
Ornatilinea apprima DSM 23815
Ardenticatena maritima DSM 23922
Caldilinea aerophila DSM 14535
Thermogemmatispora carboxidivorans PM5
Ktedonobacter racemifer DSM 44963
Thermosporothrix hazakensis ATCC BAA-1881
Herpetosiphon geysericola DSM 7119
Herpetosiphon aurantiacus DSM 785
Oscillochloris trichoides VKM B-2210
Chloroflexus aurantiacus J-10-fl
Chloroflexus aggregans DSM 9485
Roseiflexus castenholzii DSM 13941
Kallotenue papyrolyticum JKG1
Thermorudis peleae KI4
Thermomicrobium roseum DSM 5159
Sphaerobacter thermophilus DSM 20745
Rubrobacter radiotolerans DSM 5868
Rubrobacter aplysinae RV113
Rubrobacter xylanophilus DSM 9941
Thermoleophilum album ATCC 35263
Solirubrobacter soli DSM 22325
Conexibacter woesei DSM 14684
Patulibacter medicamentivorans I11
Patulibacter minatonensis DSM 18081
Patulibacter americanus DSM 16676
Thermobispora bispora DSM 43833
Kribbella catacumbae DSM 19601
Kribbella flavida DSM 17836
Friedmanniella luteola DSM 21741
Friedmanniella flava CGMCC 4.6856
Friedmanniella sagamiharensis DSM 21743
Microlunatus phosphovorus NM-1
Microlunatus soli DSM 21800
Auraticoccus monumenti DSM 23257
Naumannella halotolerans DSM 24323
Tessaracoccus flavus RP1
Tessaracoccus bendigoensis DSM 12906
Tessaracoccus flavescens KCTC 19196
Tessaracoccus lapidicaptus IPBSL-7
Tessaracoccus oleiagri CGMCC 1.9159
Propionibacterium lymphophilum DSM 4903
Propionibacterium freudenreichii DSM 20271
Propionibacterium acidifaciens DSM 21887
Propionibacterium cyclohexanicum DSM 16859
Cutibacterium avidum ATCC 25577
Propionibacterium acnes subsp. defendens ATCC 11828
Cutibacterium acnes DSM 1897
Propionibacterium namnetense CCUG 66358
Cutibacterium granulosum DSM 20700
Acidipropionibacterium acidipropionici DSM 4900
Acidipropionibacterium thoenii DSM 20276
Acidipropionibacterium jensenii DSM 20535
Granulicoccus phenolivorans DSM 17626
Raineyella antarctica LZ-22
Aestuariimicrobium kwangyangense DSM 21549
Propioniciclava tarda DSM 22130
Propionicimonas paludicola DSM 15597
Propionicicella superfundia DSM 22317
Aeromicrobium erythreum ATCC 51598
Aeromicrobium marinum DSM 15272
Aeromicrobium choanae 9H-4
Aeromicrobium massiliense JC14
Mumia flava DSM 27763
Nocardioides jensenii NBRC 14755
Marmoricola aequoreus NRRL B-24464
Marmoricola scoriae DSM 22127
Nocardioides halotolerans DSM 19273
Nocardioides alkalitolerans DSM 16699
Nocardioides insulae DSM 17944
Nocardioides terrae CGMCC 1.7056
Nocardioides simplex ATCC 6946
Nocardioides exalbidus DSM 22017
Nocardioides alpinus CGMCC 1.10697
Nocardioides dokdonensis KCTC 19309
Nocardioides lianchengensis CGMCC 4.6858
Nocardioides szechwanensis CGMCC 1.11147
Nocardioides psychrotolerans CGMCC 1.11156
Actinopolymorpha singaporensis DSM 22024
Actinopolymorpha alba DSM 45243
Acidothermus cellulolyticus 11B
Streptomyces thermoautotrophicus UBT1
Jatrophihabitans endophyticus DSM 45627
Frankia alni ACN14A
Frankia elaeagni BMG5.12
Frankia casuarinae Ccl3
Actinoplanes globisporus DSM 43857
Hamadaea tsunoensis DSM 44101
Catelliglobosispora koreensis DSM 44566
Xiangella phaseoli DSM 45730
Micromonospora pattaloongensis DSM 45245
Salinispora pacifica CNR-114
Salinispora tropica CNB-440
Micromonospora rhizosphaerae DSM 45431
Micromonospora chaiyaphumensis DSM 45246
Micromonospora auratinigra DSM 44815
Micromonospora citrea DSM 43903
Micromonospora rosaria DSM 803
Micromonospora inositola DSM 43819
Micromonospora chersina DSM 44151
Micromonospora cremea DSM 45599
Micromonospora coriariae DSM 44875
Micromonospora endolithica DSM 44398
Micromonospora tulbaghiae DSM 45142
Micromonospora peucetia DSM 43363
Micromonospora echinofusca DSM 43913
Micromonospora yangpuensis DSM 45577
Micromonospora pallida DSM 43817
Micromonospora echinospora DSM 43816
Micromonospora inyonensis DSM 46123
Micromonospora sagamiensis DSM 43912
Micromonospora nigra DSM 43818
Micromonospora eburnea DSM 44814

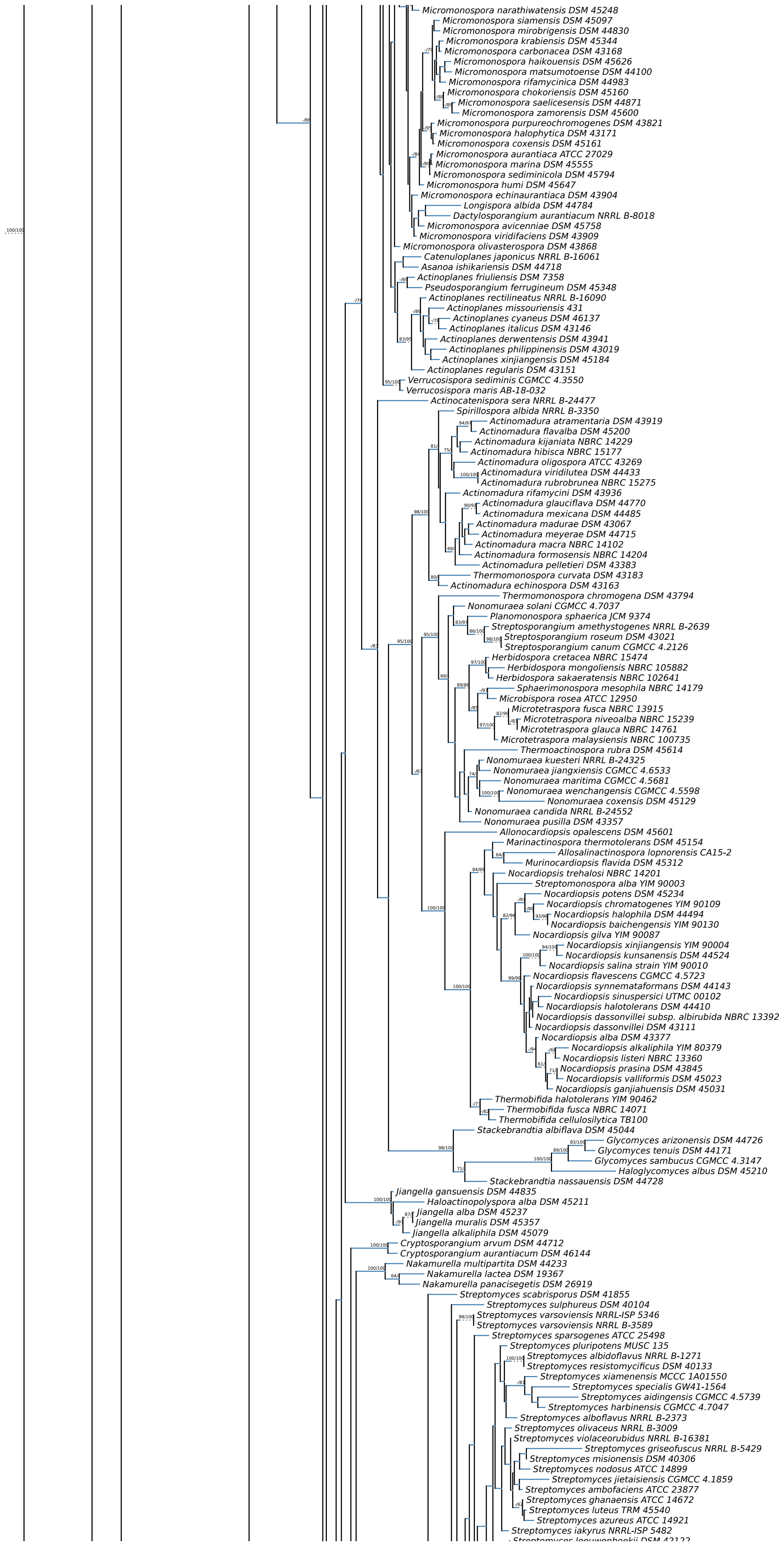
Micromonospora narathiwatensis DSM 45248
Micromonospora siamensis DSM 45097
Micromonospora mirobrigensis DSM 44830
Micromonospora krabiensis DSM 45344
Micromonospora carbonacea DSM 43168
Micromonospora haikouensis DSM 45626
Micromonospora matsumotoense DSM 44100
Micromonospora rifamycinica DSM 44983
Micromonospora chokoriensis DSM 45160
Micromonospora saelicesensis DSM 44871
Micromonospora zamorensis DSM 45600
Micromonospora purpureochromogenes DSM 43821
Micromonospora halophytica DSM 43171
Micromonospora coxensis DSM 45161
Micromonospora aurantiaca ATCC 27029
Micromonospora marina DSM 45555
Micromonospora sediminicola DSM 45794
Micromonospora humi DSM 45647
Micromonospora echinaurantiaca DSM 43904
Longispora albida DSM 44784
Dactylosporangium aurantiacum NRRL B-8018
Micromonospora avicenniae DSM 45758
Micromonospora viridifaciens DSM 43909
Micromonospora olivasterospora DSM 43868
Catenuloplanes japonicus NRRL B-16061
Asanoa ishikariensis DSM 44718
Actinoplanes friuliensis DSM 7358
Pseudosporangium ferrugineum DSM 45348
Actinoplanes rectilineatus NRRL B-16090
Actinoplanes missouriensis 431
Actinoplanes cyaneus DSM 46137
Actinoplanes italicus DSM 43146
Actinoplanes derwentensis DSM 43941
Actinoplanes philippinensis DSM 43019
Actinoplanes xinjiangensis DSM 45184
Actinoplanes regularis DSM 43151
Verrucosispora sediminis CGMCC 4.3550
Verrucosispora maris AB-18-032
Actinocatenispora sera NRRL B-24477
Spirillospora albida NRRL B-3350
Actinomadura atramentaria DSM 43919
Actinomadura flavalba DSM 45200
Actinomadura kijaniata NBRC 14229
Actinomadura hibisca NBRC 15177
Actinomadura oligospora ATCC 43269
Actinomadura viridilutea DSM 44433
Actinomadura rubrobrunea NBRC 15275
Actinomadura rifamycini DSM 43936
Actinomadura glauciflava DSM 44770
Actinomadura mexicana DSM 44485
Actinomadura madurae DSM 43067
Actinomadura meyerae DSM 44715
Actinomadura macra NBRC 14102
Actinomadura formosensis NBRC 14204
Actinomadura pelletieri DSM 43383
Thermomonospora curvata DSM 43183
Actinomadura echinospora DSM 43163
Thermomonospora chromogena DSM 43794
Nonomuraea solani CGMCC 4.7037
Planomonospora sphaerica JCM 9374
Streptosporangium amethystogenes NRRL B-2639
Streptosporangium roseum DSM 43021
Streptosporangium canum CGMCC 4.2126
Herbidospora cretacea NBRC 15474
Herbidospora mongoliensis NBRC 105882
Herbidospora sakaeratensis NBRC 102641
Sphaerimonospora mesophila NBRC 14179
Microbispora rosea ATCC 12950
Microtetraspora fusca NBRC 13915
Microtetraspora niveoalba NBRC 15239
Microtetraspora glauca NBRC 14761
Microtetraspora malaysiensis NBRC 100735
Thermoactinospora rubra DSM 45614
Nonomuraea kuesteri NRRL B-24325
Nonomuraea jiangxiensis CGMCC 4.6533
Nonomuraea maritima CGMCC 4.5681
Nonomuraea wenchangensis CGMCC 4.5598
Nonomuraea coxensis DSM 45129
Nonomuraea candida NRRL B-24552
Nonomuraea pusilla DSM 43357
Allonocardiopsis opalescens DSM 45601
Marinactinospora thermotolerans DSM 45154
Allosalinactinospora lopnorensis CA15-2
Murinocardiopsis flavida DSM 45312
Nocardiopsis trehalosi NBRC 14201
Streptomonospora alba YIM 90003
Nocardiopsis potens DSM 45234
Nocardiopsis chromatogenes YIM 90109
Nocardiopsis halophila DSM 44494
Nocardiopsis baichengensis YIM 90130
Nocardiopsis gilva YIM 90087
Nocardiopsis xinjiangensis YIM 90004
Nocardiopsis kunsanensis DSM 44524
Nocardiopsis salina strain YIM 90010
Nocardiopsis flavescens CGMCC 4.5723
Nocardiopsis synnemataformans DSM 44143
Nocardiopsis sinuspersici UTMC 00102
Nocardiopsis halotolerans DSM 44410
Nocardiopsis dassonvillei subsp. albirubida NBRC 13392
Nocardiopsis dassonvillei DSM 43111
Nocardiopsis alba DSM 43377
Nocardiopsis alkaliphila YIM 80379
Nocardiopsis listeri NBRC 13360
Nocardiopsis prasina DSM 43845
Nocardiopsis valliformis DSM 45023
Nocardiopsis ganjiahuensis DSM 45031
Thermobifida halotolerans YIM 90462
Thermobifida fusca NBRC 14071
Thermobifida cellulosilytica TB100
Stackebrandtia albiflava DSM 45044
Glycomyces arizonensis DSM 44726
Glycomyces tenuis DSM 44171
Glycomyces sambucus CGMCC 4.3147
Haloglycomyces albus DSM 45210
Stackebrandtia nassauensis DSM 44728
Jiangella gansuensis DSM 44835
Haloactinopolyspora alba DSM 45211
Jiangella alba DSM 45237
Jiangella muralis DSM 45357
Jiangella alkaliphila DSM 45079
Cryptosporangium arvum DSM 44712
Cryptosporangium aurantiacum DSM 46144
Nakamurella multipartita DSM 44233
Nakamurella lactea DSM 19367
Nakamurella panacisegetis DSM 26919
Streptomyces scabrisporus DSM 41855
Streptomyces sulphureus DSM 40104
Streptomyces varsoviensis NRRL-ISP 5346
Streptomyces varsoviensis NRRL B-3589
Streptomyces sparsogenes ATCC 25498
Streptomyces pluripotens MUSC 135
Streptomyces albidoflavus NRRL B-1271
Streptomyces resistomycificus DSM 40133
Streptomyces xiamenensis MCCC 1A01550
Streptomyces specialis GW41-1564
Streptomyces aidingensis CGMCC 4.5739
Streptomyces harbinensis CGMCC 4.7047
Streptomyces alboflavus NRRL B-2373
Streptomyces olivaceus NRRL B-3009
Streptomyces violaceorubidus NRRL B-16381
Streptomyces griseofuscus NRRL B-5429
Streptomyces misionensis DSM 40306
Streptomyces nodosus ATCC 14899
Streptomyces jietaisiensis CGMCC 4.1859
Streptomyces ambofaciens ATCC 23877
Streptomyces ghanaensis ATCC 14672
Streptomyces luteus TRM 45540
Streptomyces azureus ATCC 14921
Streptomyces iakyrus NRRL-ISP 5482

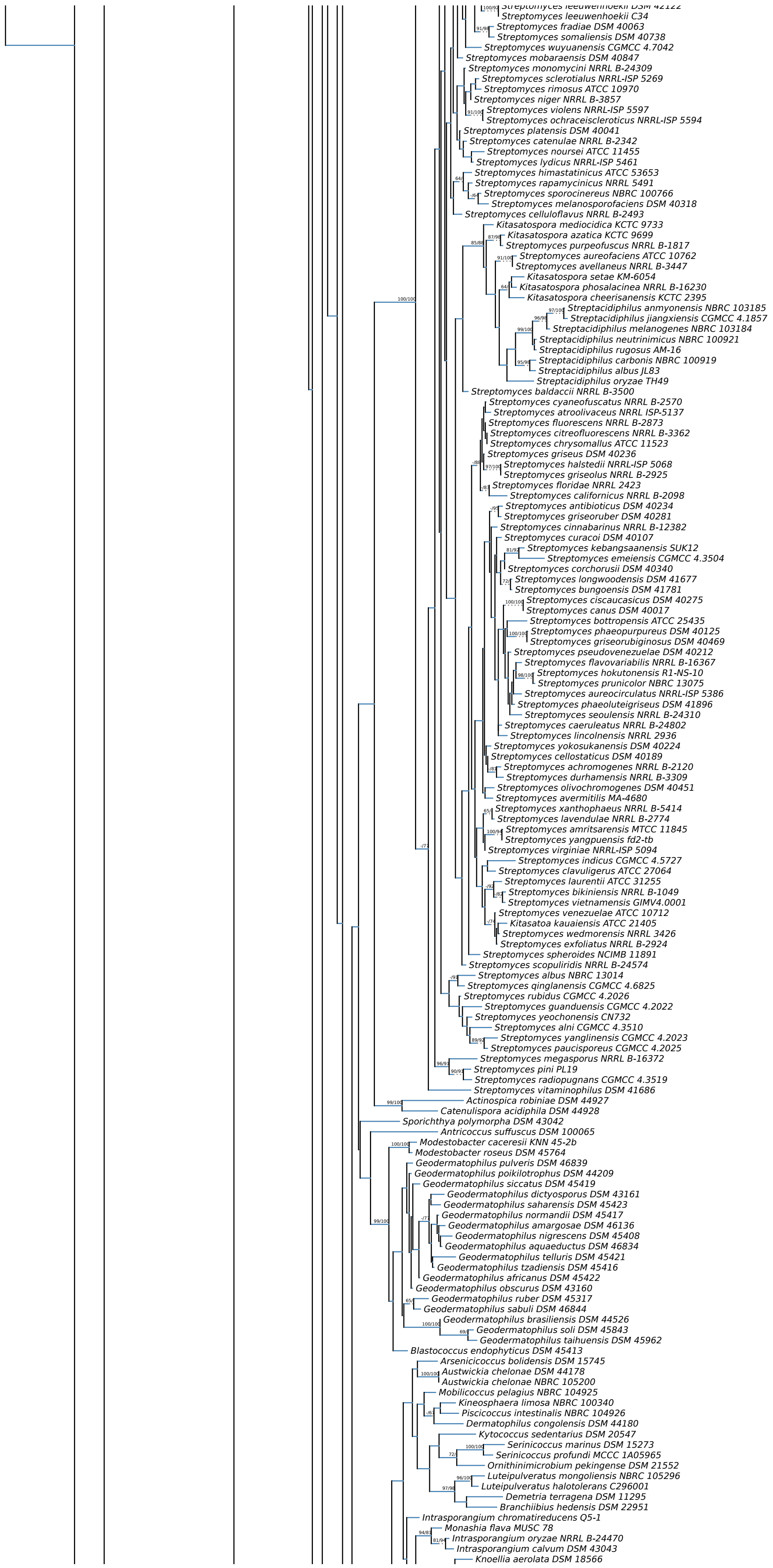
Streptomyces leeuwenhoekii DSM 42122
Streptomyces leeuwenhoekii C34
Streptomyces fradiae DSM 40063
Streptomyces somaliensis DSM 40738
Streptomyces wuyuanensis CGMCC 4.7042
Streptomyces mobaraensis DSM 40847
Streptomyces monomycini NRRL B-24309
Streptomyces sclerotialus NRRL-ISP 5269
Streptomyces rimosus ATCC 10970
Streptomyces niger NRRL B-3857
Streptomyces violens NRRL-ISP 5597
Streptomyces ochraceiscleroticus NRRL-ISP 5594
Streptomyces platensis DSM 40041
Streptomyces catenulae NRRL B-2342
Streptomyces noursei ATCC 11455
Streptomyces lydicus NRRL-ISP 5461
Streptomyces himastatinicus ATCC 53653
Streptomyces rapamycinicus NRRL 5491
Streptomyces sporocinereus NBRC 100766
Streptomyces melanosporofaciens DSM 40318
Streptomyces celluloflavus NRRL B-2493
Kitasatospora mediocidica KCTC 9733
Kitasatospora azatica KCTC 9699
Streptomyces purpeofuscus NRRL B-1817
Streptomyces aureofaciens ATCC 10762
Streptomyces avellaneus NRRL B-3447
Kitasatospora setae KM-6054
Kitasatospora phosalacinea NRRL B-16230
Kitasatospora cheerisanensis KCTC 2395
Streptacidiphilus anmyonensis NBRC 103185
Streptacidiphilus jiangxiensis CGMCC 4.1857
Streptacidiphilus melanogenes NBRC 103184
Streptacidiphilus neutrinimicus NBRC 100921
Streptacidiphilus rugosus AM-16
Streptacidiphilus carbonis NBRC 100919
Streptacidiphilus albus JL83
Streptacidiphilus oryzae TH49
Streptomyces baldaccii NRRL B-3500
Streptomyces cyaneofuscatus NRRL B-2570
Streptomyces atroolivaceus NRRL ISP-5137
Streptomyces fluorescens NRRL B-2873
Streptomyces citreofluorescens NRRL B-3362
Streptomyces chrysomallus ATCC 11523
Streptomyces griseus DSM 40236
Streptomyces halstedii NRRL-ISP 5068
Streptomyces griseolus NRRL B-2925
Streptomyces floridae NRRL 2423
Streptomyces californicus NRRL B-2098
Streptomyces antibioticus DSM 40234
Streptomyces griseoruber DSM 40281
Streptomyces cinnabarinus NRRL B-12382
Streptomyces curacoi DSM 40107
Streptomyces kebangsaanensis SUK12
Streptomyces emeiensis CGMCC 4.3504
Streptomyces corchorusii DSM 40340
Streptomyces longwoodensis DSM 41677
Streptomyces bungoensis DSM 41781
Streptomyces ciscaucasicus DSM 40275
Streptomyces canus DSM 40017
Streptomyces bottropensis ATCC 25435
Streptomyces phaeopurpureus DSM 40125
Streptomyces griseorubiginosus DSM 40469
Streptomyces pseudovenezuelae DSM 40212
Streptomyces flavovariabilis NRRL B-16367
Streptomyces hokutonensis R1-NS-10
Streptomyces prunicolor NBRC 13075
Streptomyces aureocirculatus NRRL-ISP 5386
Streptomyces phaeoluteigriseus DSM 41896
Streptomyces seoulensis NRRL B-24310
Streptomyces caeruleatus NRRL B-24802
Streptomyces lincolnensis NRRL 2936
Streptomyces yokosukanensis DSM 40224
Streptomyces cellostaticus DSM 40189
Streptomyces achromogenes NRRL B-2120
Streptomyces durhamensis NRRL B-3309
Streptomyces olivochromogenes DSM 40451
Streptomyces avermitilis MA-4680
Streptomyces xanthophaeus NRRL B-5414
Streptomyces lavendulae NRRL B-2774
Streptomyces amritsarensis MTCC 11845
Streptomyces yangpuensis fd2-tb
Streptomyces virginiae NRRL-ISP 5094
Streptomyces indicus CGMCC 4.5727
Streptomyces clavuligerus ATCC 27064
Streptomyces laurentii ATCC 31255
Streptomyces bikiniensis NRRL B-1049
Streptomyces vietnamensis GIMV4.0001
Streptomyces venezuelae ATCC 10712
Kitasatoa kauaiensis ATCC 21405
Streptomyces wedmorensis NRRL 3426
Streptomyces exfoliatus NRRL B-2924
Streptomyces spheroides NCIMB 11891
Streptomyces scopuliridis NRRL B-24574
Streptomyces albus NBRC 13014
Streptomyces qinglanensis CGMCC 4.6825
Streptomyces rubidus CGMCC 4.2026
Streptomyces guanduensis CGMCC 4.2022
Streptomyces yeochonensis CN732
Streptomyces alni CGMCC 4.3510
Streptomyces yanglinensis CGMCC 4.2023
Streptomyces paucisporeus CGMCC 4.2025
Streptomyces megasporus NRRL B-16372
Streptomyces pini PL19
Streptomyces radiopugnans CGMCC 4.3519
Streptomyces vitaminophilus DSM 41686
Actinospica robiniae DSM 44927
Catenulispora acidiphila DSM 44928
Sporichthya polymorpha DSM 43042
Antricoccus suffuscus DSM 100065
Modestobacter caceresii KNN 45-2b
Modestobacter roseus DSM 45764
Geodermatophilus pulveris DSM 46839
Geodermatophilus poikilotrophus DSM 44209
Geodermatophilus siccatus DSM 45419
Geodermatophilus dictyosporus DSM 43161
Geodermatophilus saharensis DSM 45423
Geodermatophilus normandii DSM 45417
Geodermatophilus amargosae DSM 46136
Geodermatophilus nigrescens DSM 45408
Geodermatophilus aquaeductus DSM 46834
Geodermatophilus telluris DSM 45421
Geodermatophilus tzadiensis DSM 45416
Geodermatophilus africanus DSM 45422
Geodermatophilus obscurus DSM 43160
Geodermatophilus ruber DSM 45317
Geodermatophilus sabuli DSM 46844
Geodermatophilus brasiliensis DSM 44526
Geodermatophilus soli DSM 45843
Geodermatophilus taihuensis DSM 45962
Blastococcus endophyticus DSM 45413
Arsenicicoccus bolidensis DSM 15745
Austwickia chelonae DSM 44178
Austwickia chelonae NBRC 105200
Mobilicoccus pelagius NBRC 104925
Kineosphaera limosa NBRC 100340
Piscicoccus intestinalis NBRC 104926
Dermatophilus congolensis DSM 44180
Kytococcus sedentarius DSM 20547
Serinicoccus marinus DSM 15273
Serinicoccus profundi MCCC 1A05965
Ornithinimicrobium pekingense DSM 21552
Luteipulveratus mongoliensis NBRC 105296
Luteipulveratus halotolerans C296001
Demetria terragena DSM 11295
Branchiibius hedensis DSM 22951
Intrasporangium chromatireducens Q5-1
Monashia flava MUSC 78
Intrasporangium oryzae NRRL B-24470
Intrasporangium calvum DSM 43043
Knoellia aerolata DSM 18566

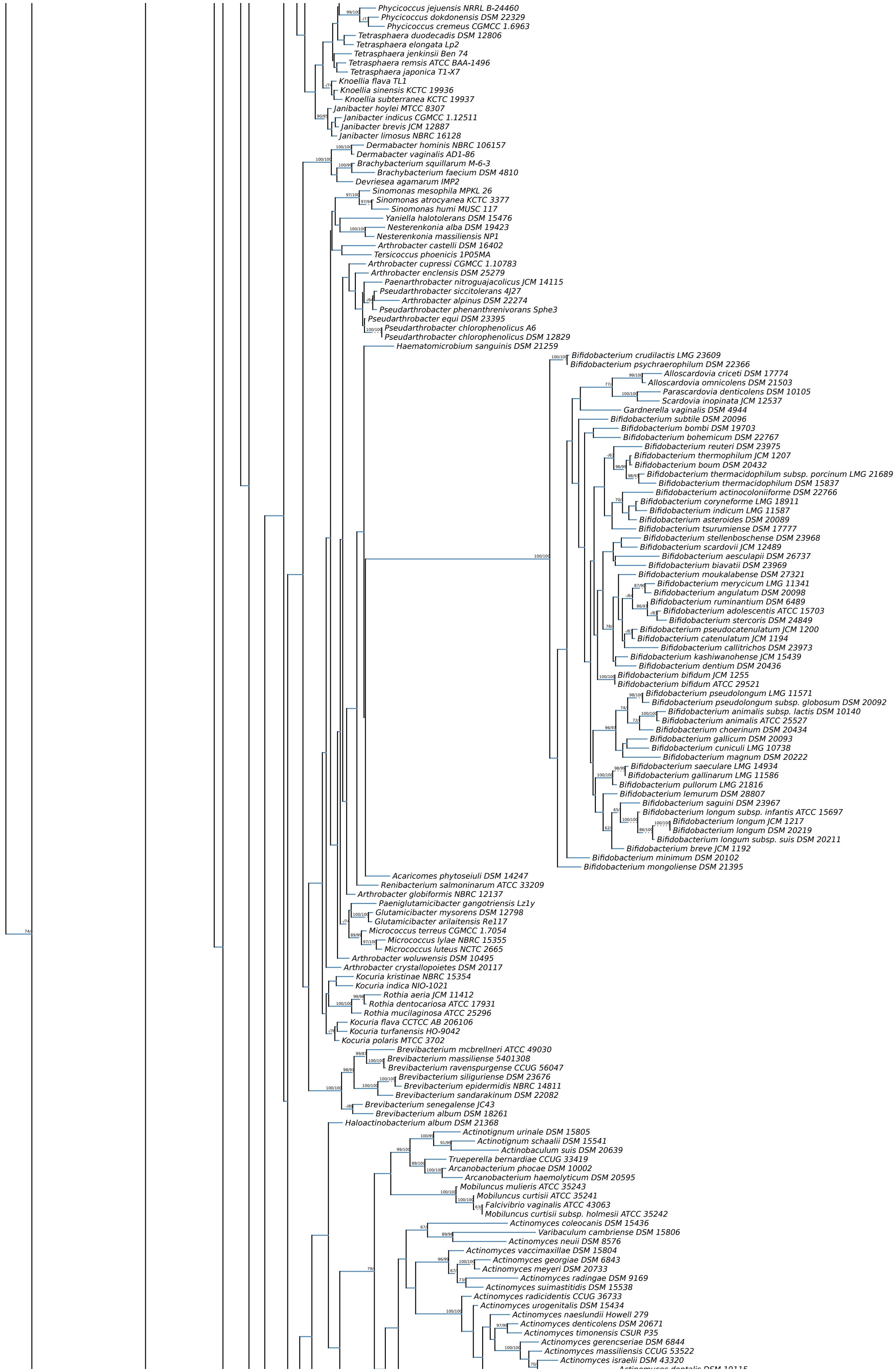

Phycicoccus jejuensis NRRL B-24460
Phycicoccus dokdonensis DSM 22329
Phycicoccus cremeus CGMCC 1.6963
Tetrasphaera duodecadis DSM 12806
Tetrasphaera elongata Lp2
Tetrasphaera jenkinsii Ben 74
Tetrasphaera remsis ATCC BAA-1496
Tetrasphaera japonica T1-X7
Knoellia flava TL1
Knoellia sinensis KCTC 19936
Knoellia subterranea KCTC 19937
Janibacter hoylei MTCC 8307
Janibacter indicus CGMCC 1.12511
Janibacter brevis JCM 12887
Janibacter limosus NBRC 16128
Dermabacter hominis NBRC 106157
Dermabacter vaginalis AD1-86
Brachybacterium squillarum M-6-3
Brachybacterium faecium DSM 4810
Devriesea agamarum IMP2
Sinomonas mesophila MPKL 26
Sinomonas atrocyanea KCTC 3377
Sinomonas humi MUSC 117
Yaniella halotolerans DSM 15476
Nesterenkonia alba DSM 19423
Nesterenkonia massiliensis NP1
Arthrobacter castelli DSM 16402
Tersicoccus phoenicis 1P05MA
Arthrobacter cupressi CGMCC 1.10783
Arthrobacter enclensis DSM 25279
Paenarthrobacter nitroguajacolicus JCM 14115
Pseudarthrobacter siccitolerans 4J27
Arthrobacter alpinus DSM 22274
Pseudarthrobacter phenanthrenivorans Sphe3
Pseudarthrobacter equi DSM 23395
Pseudarthrobacter chlorophenolicus A6
Pseudarthrobacter chlorophenolicus DSM 12829
Haematomicrobium sanguinis DSM 21259
Bifidobacterium crudilactis LMG 23609
Bifidobacterium psychraerophilum DSM 22366
Alloscardovia criceti DSM 17774
Alloscardovia omnicolens DSM 21503
Parascardovia denticolens DSM 10105
Scardovia inopinata JCM 12537
Gardnerella vaginalis DSM 4944
Bifidobacterium subtile DSM 20096
Bifidobacterium bombi DSM 19703
Bifidobacterium bohemicum DSM 22767
Bifidobacterium reuteri DSM 23975
Bifidobacterium thermophilum JCM 1207
Bifidobacterium boum DSM 20432
Bifidobacterium thermacidophilum subsp. porcinum LMG 21689
Bifidobacterium thermacidophilum DSM 15837
Bifidobacterium actinocoloniiforme DSM 22766
Bifidobacterium coryneforme LMG 18911
Bifidobacterium indicum LMG 11587
Bifidobacterium asteroides DSM 20089
Bifidobacterium tsurumiense DSM 17777
Bifidobacterium stellenboschense DSM 23968
Bifidobacterium scardovii JCM 12489
Bifidobacterium aesculapii DSM 26737
Bifidobacterium biavatii DSM 23969
Bifidobacterium moukalabense DSM 27321
Bifidobacterium merycicum LMG 11341
Bifidobacterium angulatum DSM 20098
Bifidobacterium ruminantium DSM 6489
Bifidobacterium adolescentis ATCC 15703
Bifidobacterium stercoris DSM 24849
Bifidobacterium pseudocatenulatum JCM 1200
Bifidobacterium catenulatum JCM 1194
Bifidobacterium callitrichos DSM 23973
Bifidobacterium kashiwanohense JCM 15439
Bifidobacterium dentium DSM 20436
Bifidobacterium bifidum JCM 1255
Bifidobacterium bifidum ATCC 29521
Bifidobacterium pseudolongum LMG 11571
Bifidobacterium pseudolongum subsp. globosum DSM 20092
Bifidobacterium animalis subsp. lactis DSM 10140
Bifidobacterium animalis ATCC 25527
Bifidobacterium choerinum DSM 20434
Bifidobacterium gallicum DSM 20093
Bifidobacterium cuniculi LMG 10738
Bifidobacterium magnum DSM 20222
Bifidobacterium saeculare LMG 14934
Bifidobacterium gallinarum LMG 11586
Bifidobacterium pullorum LMG 21816
Bifidobacterium lemurum DSM 28807
Bifidobacterium saguini DSM 23967
Bifidobacterium longum subsp. infantis ATCC 15697
Bifidobacterium longum JCM 1217
Bifidobacterium longum DSM 20219
Bifidobacterium longum subsp. suis DSM 20211
Bifidobacterium breve JCM 1192
Bifidobacterium minimum DSM 20102
Bifidobacterium mongoliense DSM 21395
Acaricomes phytoseiuli DSM 14247
Renibacterium salmoninarum ATCC 33209
Arthrobacter globiformis NBRC 12137
Paeniglutamicibacter gangotriensis Lz1y
Glutamicibacter mysorens DSM 12798
Glutamicibacter arilaitensis Re117
Micrococcus terreus CGMCC 1.7054
Micrococcus lylae NBRC 15355
Micrococcus luteus NCTC 2665
Arthrobacter woluwensis DSM 10495
Arthrobacter crystallopoietes DSM 20117
Kocuria kristinae NBRC 15354
Kocuria indica NIO-1021
Rothia aeria JCM 11412
Rothia dentocariosa ATCC 17931
Rothia mucilaginosa ATCC 25296
Kocuria flava CCTCC AB 206106
Kocuria turfanensis HO-9042
Kocuria polaris MTCC 3702
Brevibacterium mcbrellneri ATCC 49030
Brevibacterium massiliense 5401308
Brevibacterium ravenspurgense CCUG 56047
Brevibacterium siliguriense DSM 23676
Brevibacterium epidermidis NBRC 14811
Brevibacterium sandarakinum DSM 22082
Brevibacterium senegalense JC43
Brevibacterium album DSM 18261
Haloactinobacterium album DSM 21368
Actinotignum urinale DSM 15805
Actinotignum schaalii DSM 15541
Actinobaculum suis DSM 20639
Trueperella bernardiae CCUG 33419
Arcanobacterium phocae DSM 10002
Arcanobacterium haemolyticum DSM 20595
Mobiluncus mulieris ATCC 35243
Mobiluncus curtisii ATCC 35241
Falcivibrio vaginalis ATCC 43063
Mobiluncus curtisii subsp. holmesii ATCC 35242
Actinomyces coleocanis DSM 15436
Varibaculum cambriense DSM 15806
Actinomyces neuii DSM 8576
Actinomyces vaccimaxillae DSM 15804
Actinomyces georgiae DSM 6843
Actinomyces meyeri DSM 20733
Actinomyces radingae DSM 9169
Actinomyces suimastitidis DSM 15538
Actinomyces radicidentis CCUG 36733
Actinomyces urogenitalis DSM 15434
Actinomyces naeslundii Howell 279
Actinomyces denticolens DSM 20671
Actinomyces timonensis CSUR P35
Actinomyces gerencseriae DSM 6844
Actinomyces massiliensis CCUG 53522
Actinomyces israelii DSM 43320

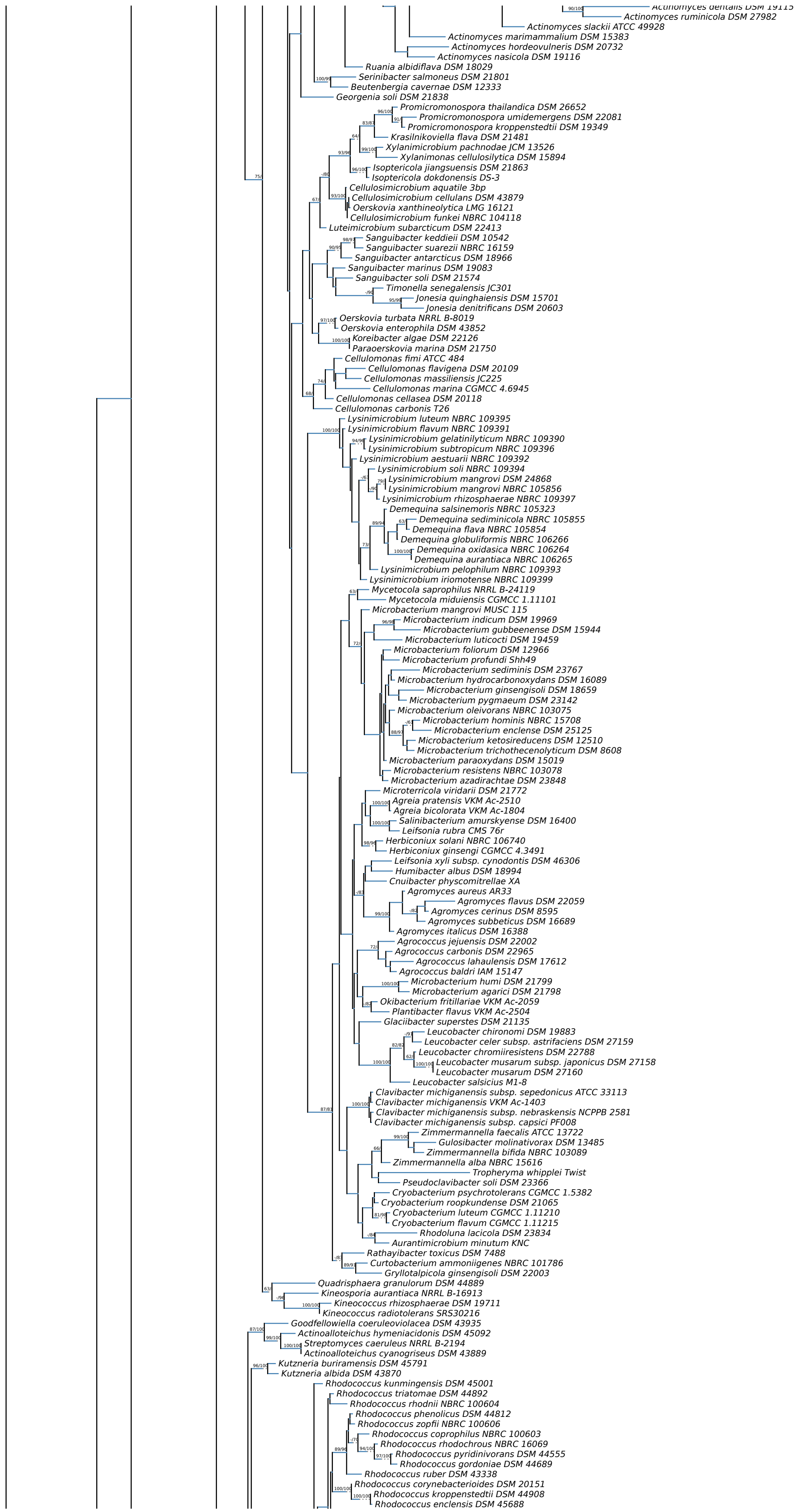
Actinomyces dentalis DSM 19115
Actinomyces ruminicola DSM 27982
Actinomyces slackii ATCC 49928
Actinomyces marimammalium DSM 15383
Actinomyces hordeovulneris DSM 20732
Actinomyces nasicola DSM 19116
Ruania albidiflava DSM 18029
Serinibacter salmoneus DSM 21801
Beutenbergia cavernae DSM 12333
Georgenia soli DSM 21838
Promicromonospora thailandica DSM 26652
Promicromonospora umidemergens DSM 22081
Promicromonospora kroppenstedtii DSM 19349
Krasilnikoviella flava DSM 21481
Xylanimicrobium pachnodae JCM 13526
Xylanimonas cellulosilytica DSM 15894
Isoptericola jiangsuensis DSM 21863
Isoptericola dokdonensis DS-3
Cellulosimicrobium aquatile 3bp
Cellulosimicrobium cellulans DSM 43879
Oerskovia xanthineolytica LMG 16121
Cellulosimicrobium funkei NBRC 104118
Luteimicrobium subarcticum DSM 22413
Sanguibacter keddieii DSM 10542
Sanguibacter suarezii NBRC 16159
Sanguibacter antarcticus DSM 18966
Sanguibacter marinus DSM 19083
Sanguibacter soli DSM 21574
Timonella senegalensis JC301
Jonesia quinghaiensis DSM 15701
Jonesia denitrificans DSM 20603
Oerskovia turbata NRRL B-8019
Oerskovia enterophila DSM 43852
Koreibacter algae DSM 22126
Paraoerskovia marina DSM 21750
Cellulomonas fimi ATCC 484
Cellulomonas flavigena DSM 20109
Cellulomonas massiliensis JC225
Cellulomonas marina CGMCC 4.6945
Cellulomonas cellasea DSM 20118
Cellulomonas carbonis T26
Lysinimicrobium luteum NBRC 109395
Lysinimicrobium flavum NBRC 109391
Lysinimicrobium gelatinilyticum NBRC 109390
Lysinimicrobium subtropicum NBRC 109396
Lysinimicrobium aestuarii NBRC 109392
Lysinimicrobium soli NBRC 109394
Lysinimicrobium mangrovi DSM 24868
Lysinimicrobium mangrovi NBRC 105856
Lysinimicrobium rhizosphaerae NBRC 109397
Demequina salsinemoris NBRC 105323
Demequina sediminicola NBRC 105855
Demequina flava NBRC 105854
Demequina globuliformis NBRC 106266
Demequina oxidasica NBRC 106264
Demequina aurantiaca NBRC 106265
Lysinimicrobium pelophilum NBRC 109393
Lysinimicrobium iriomotense NBRC 109399
Mycetocola saprophilus NRRL B-24119
Mycetocola miduiensis CGMCC 1.11101
Microbacterium mangrovi MUSC 115
Microbacterium indicum DSM 19969
Microbacterium gubbeenense DSM 15944
Microbacterium luticocti DSM 19459
Microbacterium foliorum DSM 12966
Microbacterium profundi Shh49
Microbacterium sediminis DSM 23767
Microbacterium hydrocarbonoxydans DSM 16089
Microbacterium ginsengisoli DSM 18659
Microbacterium pygmaeum DSM 23142
Microbacterium oleivorans NBRC 103075
Microbacterium hominis NBRC 15708
Microbacterium enclense DSM 25125
Microbacterium ketosireducens DSM 12510
Microbacterium trichothecenolyticum DSM 8608
Microbacterium paraoxydans DSM 15019
Microbacterium resistens NBRC 103078
Microbacterium azadirachtae DSM 23848
Microterricola viridarii DSM 21772
Agreia pratensis VKM Ac-2510
Agreia bicolorata VKM Ac-1804
Salinibacterium amurskyense DSM 16400
Leifsonia rubra CMS 76r
Herbiconiux solani NBRC 106740
Herbiconiux ginsengi CGMCC 4.3491
Leifsonia xyli subsp. cynodontis DSM 46306
Humibacter albus DSM 18994
Cnuibacter physcomitrellae XA
Agromyces aureus AR33
Agromyces flavus DSM 22059
Agromyces cerinus DSM 8595
Agromyces subbeticus DSM 16689
Agromyces italicus DSM 16388
Agrococcus jejuensis DSM 22002
Agrococcus carbonis DSM 22965
Agrococcus lahaulensis DSM 17612
Agrococcus baldri IAM 15147
Microbacterium humi DSM 21799
Microbacterium agarici DSM 21798
Okibacterium fritillariae VKM Ac-2059
Plantibacter flavus VKM Ac-2504
Glaciibacter superstes DSM 21135
Leucobacter chironomi DSM 19883
Leucobacter celer subsp. astrifaciens DSM 27159
Leucobacter chromiiresistens DSM 22788
Leucobacter musarum subsp. japonicus DSM 27158
Leucobacter musarum DSM 27160
Leucobacter salsicius M1-8
Clavibacter michiganensis subsp. sepedonicus ATCC 33113
Clavibacter michiganensis VKM Ac-1403
Clavibacter michiganensis subsp. nebraskensis NCPPB 2581
Clavibacter michiganensis subsp. capsici PF008
Zimmermannella faecalis ATCC 13722
Gulosibacter molinativorax DSM 13485
Zimmermannella bifida NBRC 103089
Zimmermannella alba NBRC 15616
Tropheryma whipplei Twist
Pseudoclavibacter soli DSM 23366
Cryobacterium psychrotolerans CGMCC 1.5382
Cryobacterium roopkundense DSM 21065
Cryobacterium luteum CGMCC 1.11210
Cryobacterium flavum CGMCC 1.11215
Rhodoluna lacicola DSM 23834
Aurantimicrobium minutum KNC
Rathayibacter toxicus DSM 7488
Curtobacterium ammoniigenes NBRC 101786
Gryllotalpicola ginsengisoli DSM 22003
Quadrisphaera granulorum DSM 44889
Kineosporia aurantiaca NRRL B-16913
Kineococcus rhizosphaerae DSM 19711
Kineococcus radiotolerans SRS30216
Goodfellowiella coeruleoviolacea DSM 43935
Actinoalloteichus hymeniacidonis DSM 45092
Streptomyces caeruleus NRRL B-2194
Actinoalloteichus cyanogriseus DSM 43889
Kutzneria buriramensis DSM 45791
Kutzneria albida DSM 43870
Rhodococcus kunmingensis DSM 45001
Rhodococcus triatomae DSM 44892
Rhodococcus rhodnii NBRC 100604
Rhodococcus phenolicus DSM 44812
Rhodococcus zopfii NBRC 100606
Rhodococcus coprophilus NBRC 100603
Rhodococcus rhodochrous NBRC 16069
Rhodococcus pyridinivorans DSM 44555
Rhodococcus gordoniae DSM 44689
Rhodococcus ruber DSM 43338
Rhodococcus corynebacterioides DSM 20151
Rhodococcus kroppenstedtii DSM 44908
Rhodococcus enclensis DSM 45688

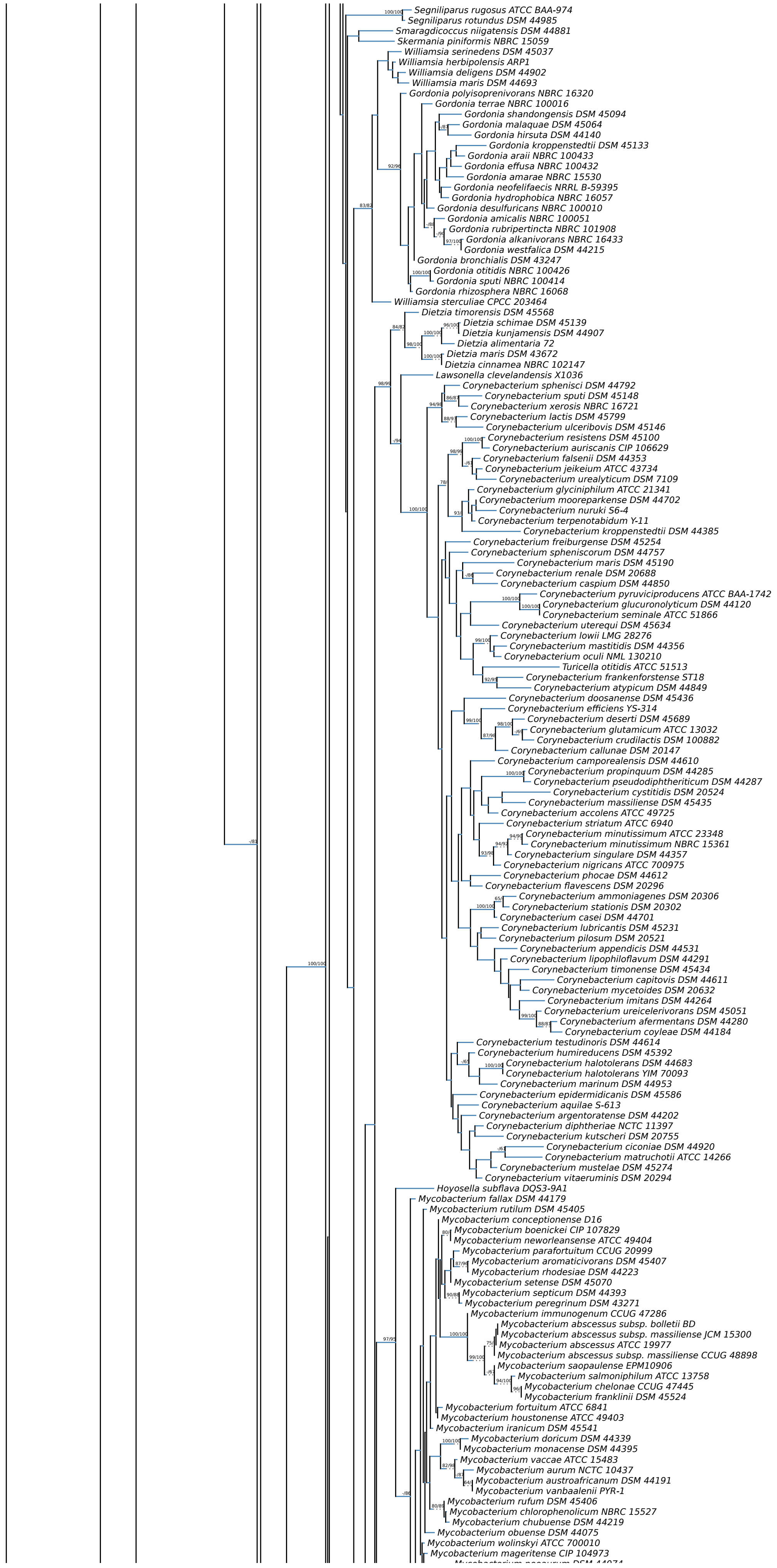

Segniliparus rugosus ATCC BAA-974
Segniliparus rotundus DSM 44985
Smaragdicoccus niigatensis DSM 44881
Skermania piniformis NBRC 15059
Williamsia serinedens DSM 45037
Williamsia herbipolensis ARP1
Williamsia deligens DSM 44902
Williamsia maris DSM 44693
Gordonia polyisoprenivorans NBRC 16320
Gordonia terrae NBRC 100016
Gordonia shandongensis DSM 45094
Gordonia malaquae DSM 45064
Gordonia hirsuta DSM 44140
Gordonia kroppenstedtii DSM 45133
Gordonia araii NBRC 100433
Gordonia effusa NBRC 100432
Gordonia amarae NBRC 15530
Gordonia neofelifaecis NRRL B-59395
Gordonia hydrophobica NBRC 16057
Gordonia desulfuricans NBRC 100010
Gordonia amicalis NBRC 100051
Gordonia rubripertincta NBRC 101908
Gordonia alkanivorans NBRC 16433
Gordonia westfalica DSM 44215
Gordonia bronchialis DSM 43247
Gordonia otitidis NBRC 100426
Gordonia sputi NBRC 100414
Gordonia rhizosphera NBRC 16068
Williamsia sterculiae CPCC 203464
Dietzia timorensis DSM 45568
Dietzia schimae DSM 45139
Dietzia kunjamensis DSM 44907
Dietzia alimentaria 72
Dietzia maris DSM 43672
Dietzia cinnamea NBRC 102147
Lawsonella clevelandensis X1036
Corynebacterium sphenisci DSM 44792
Corynebacterium sputi DSM 45148
Corynebacterium xerosis NBRC 16721
Corynebacterium lactis DSM 45799
Corynebacterium ulceribovis DSM 45146
Corynebacterium resistens DSM 45100
Corynebacterium auriscanis CIP 106629
Corynebacterium falsenii DSM 44353
Corynebacterium jeikeium ATCC 43734
Corynebacterium urealyticum DSM 7109
Corynebacterium glyciniphilum ATCC 21341
Corynebacterium mooreparkense DSM 44702
Corynebacterium nuruki S6-4
Corynebacterium terpenotabidum Y-11
Corynebacterium kroppenstedtii DSM 44385
Corynebacterium freiburgense DSM 45254
Corynebacterium spheniscorum DSM 44757
Corynebacterium maris DSM 45190
Corynebacterium renale DSM 20688
Corynebacterium caspium DSM 44850
Corynebacterium pyruviciproducens ATCC BAA-1742
Corynebacterium glucuronolyticum DSM 44120
Corynebacterium seminale ATCC 51866
Corynebacterium uterequi DSM 45634
Corynebacterium lowii LMG 28276
Corynebacterium mastitidis DSM 44356
Corynebacterium oculi NML 130210
Turicella otitidis ATCC 51513
Corynebacterium frankenforstense ST18
Corynebacterium atypicum DSM 44849
Corynebacterium doosanense DSM 45436
Corynebacterium efficiens YS-314
Corynebacterium deserti DSM 45689
Corynebacterium glutamicum ATCC 13032
Corynebacterium crudilactis DSM 100882
Corynebacterium callunae DSM 20147
Corynebacterium camporealensis DSM 44610
Corynebacterium propinquum DSM 44285
Corynebacterium pseudodiphtheriticum DSM 44287
Corynebacterium cystitidis DSM 20524
Corynebacterium massiliense DSM 45435
Corynebacterium accolens ATCC 49725
Corynebacterium striatum ATCC 6940
Corynebacterium minutissimum ATCC 23348
Corynebacterium minutissimum NBRC 15361
Corynebacterium singulare DSM 44357
Corynebacterium nigricans ATCC 700975
Corynebacterium phocae DSM 44612
Corynebacterium flavescens DSM 20296
Corynebacterium ammoniagenes DSM 20306
Corynebacterium stationis DSM 20302
Corynebacterium casei DSM 44701
Corynebacterium lubricantis DSM 45231
Corynebacterium pilosum DSM 20521
Corynebacterium appendicis DSM 44531
Corynebacterium lipophiloflavum DSM 44291
Corynebacterium timonense DSM 45434
Corynebacterium capitovis DSM 44611
Corynebacterium mycetoides DSM 20632
Corynebacterium imitans DSM 44264
Corynebacterium ureicelerivorans DSM 45051
Corynebacterium afermentans DSM 44280
Corynebacterium coyleae DSM 44184
Corynebacterium testudinoris DSM 44614
Corynebacterium humireducens DSM 45392
Corynebacterium halotolerans DSM 44683
Corynebacterium halotolerans YIM 70093
Corynebacterium marinum DSM 44953
Corynebacterium epidermidicanis DSM 45586
Corynebacterium aquilae S-613
Corynebacterium argentoratense DSM 44202
Corynebacterium diphtheriae NCTC 11397
Corynebacterium kutscheri DSM 20755
Corynebacterium ciconiae DSM 44920
Corynebacterium matruchotii ATCC 14266
Corynebacterium mustelae DSM 45274
Corynebacterium vitaeruminis DSM 20294
Hoyosella subflava DQS3-9A1
Mycobacterium fallax DSM 44179
Mycobacterium rutilum DSM 45405
Mycobacterium conceptionense D16
Mycobacterium boenickei CIP 107829
Mycobacterium neworleansense ATCC 49404
Mycobacterium parafortuitum CCUG 20999
Mycobacterium aromaticivorans DSM 45407
Mycobacterium rhodesiae DSM 44223
Mycobacterium setense DSM 45070
Mycobacterium septicum DSM 44393
Mycobacterium peregrinum DSM 43271
Mycobacterium immunogenum CCUG 47286
Mycobacterium abscessus subsp. bolletii BD
Mycobacterium abscessus subsp. massiliense JCM 15300
Mycobacterium abscessus ATCC 19977
Mycobacterium abscessus subsp. massiliense CCUG 48898
Mycobacterium saopaulense EPM10906
Mycobacterium salmoniphilum ATCC 13758
Mycobacterium chelonae CCUG 47445
Mycobacterium franklinii DSM 45524
Mycobacterium fortuitum ATCC 6841
Mycobacterium houstonense ATCC 49403
Mycobacterium iranicum DSM 45541
Mycobacterium doricum DSM 44339
Mycobacterium monacense DSM 44395
Mycobacterium vaccae ATCC 15483
Mycobacterium aurum NCTC 10437
Mycobacterium austroafricanum DSM 44191
Mycobacterium vanbaalenii PYR-1
Mycobacterium rufum DSM 45406
Mycobacterium chlorophenolicum NBRC 15527
Mycobacterium chubuense DSM 44219
Mycobacterium obuense DSM 44075
Mycobacterium wolinskyi ATCC 700010
Mycobacterium mageritense CIP 104973

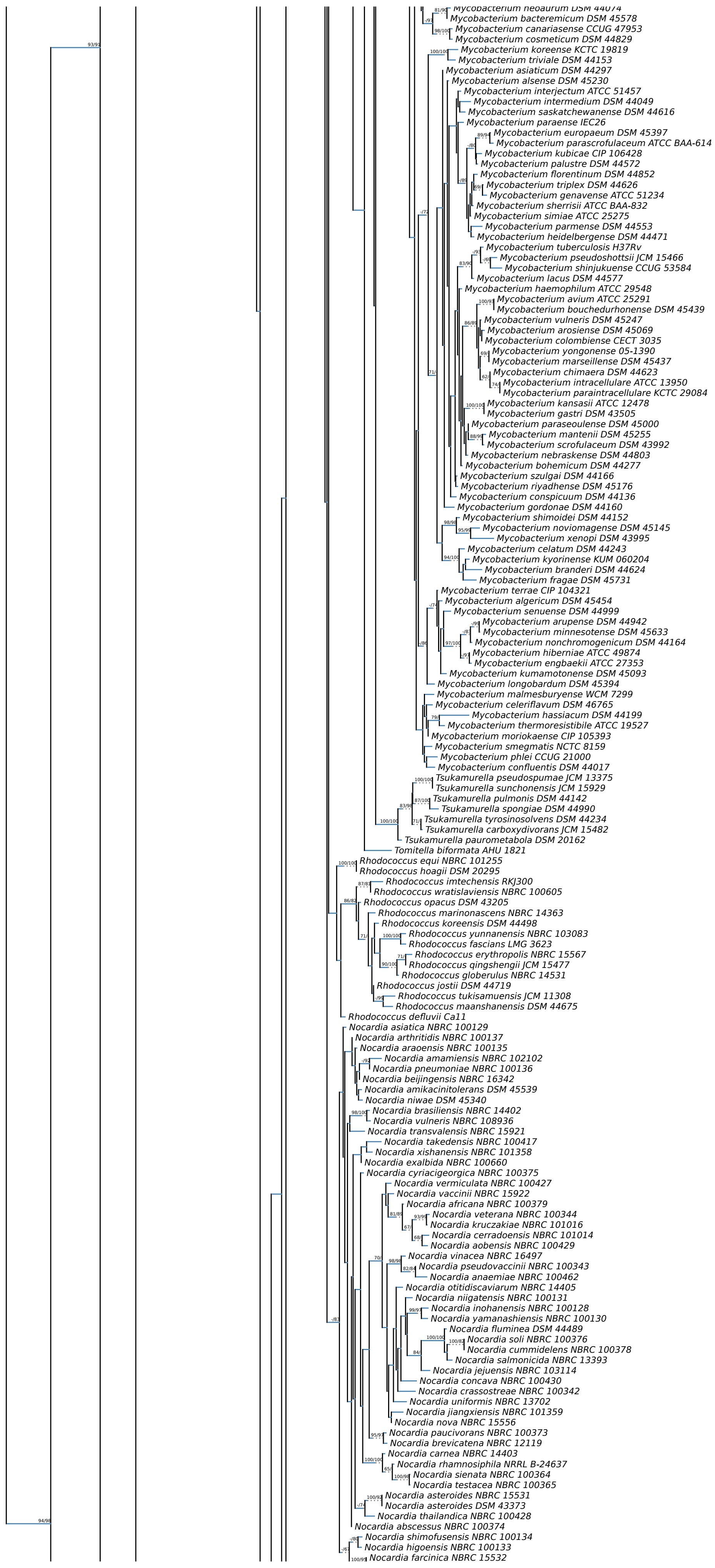
Mycobacterium neoaurum DSM 44074
Mycobacterium bacteremicum DSM 45578
Mycobacterium canariasense CCUG 47953
Mycobacterium cosmeticum DSM 44829
Mycobacterium koreense KCTC 19819
Mycobacterium triviale DSM 44153
Mycobacterium asiaticum DSM 44297
Mycobacterium alsense DSM 45230
Mycobacterium interjectum ATCC 51457
Mycobacterium intermedium DSM 44049
Mycobacterium saskatchewanense DSM 44616
Mycobacterium paraense IEC26
Mycobacterium europaeum DSM 45397
Mycobacterium parascrofulaceum ATCC BAA-614
Mycobacterium kubicae CIP 106428
Mycobacterium palustre DSM 44572
Mycobacterium florentinum DSM 44852
Mycobacterium triplex DSM 44626
Mycobacterium genavense ATCC 51234
Mycobacterium sherrisii ATCC BAA-832
Mycobacterium simiae ATCC 25275
Mycobacterium parmense DSM 44553
Mycobacterium heidelbergense DSM 44471
Mycobacterium tuberculosis H37Rv
Mycobacterium pseudoshottsii JCM 15466
Mycobacterium shinjukuense CCUG 53584
Mycobacterium lacus DSM 44577
Mycobacterium haemophilum ATCC 29548
Mycobacterium avium ATCC 25291
Mycobacterium bouchedurhonense DSM 45439
Mycobacterium vulneris DSM 45247
Mycobacterium arosiense DSM 45069
Mycobacterium colombiense CECT 3035
Mycobacterium yongonense 05-1390
Mycobacterium marseillense DSM 45437
Mycobacterium chimaera DSM 44623
Mycobacterium intracellulare ATCC 13950
Mycobacterium paraintracellulare KCTC 29084
Mycobacterium kansasii ATCC 12478
Mycobacterium gastri DSM 43505
Mycobacterium paraseoulense DSM 45000
Mycobacterium mantenii DSM 45255
Mycobacterium scrofulaceum DSM 43992
Mycobacterium nebraskense DSM 44803
Mycobacterium bohemicum DSM 44277
Mycobacterium szulgai DSM 44166
Mycobacterium riyadhense DSM 45176
Mycobacterium conspicuum DSM 44136
Mycobacterium gordonae DSM 44160
Mycobacterium shimoidei DSM 44152
Mycobacterium noviomagense DSM 45145
Mycobacterium xenopi DSM 43995
Mycobacterium celatum DSM 44243
Mycobacterium kyorinense KUM 060204
Mycobacterium branderi DSM 44624
Mycobacterium fragae DSM 45731
Mycobacterium terrae CIP 104321
Mycobacterium algericum DSM 45454
Mycobacterium senuense DSM 44999
Mycobacterium arupense DSM 44942
Mycobacterium minnesotense DSM 45633
Mycobacterium nonchromogenicum DSM 44164
Mycobacterium hiberniae ATCC 49874
Mycobacterium engbaekii ATCC 27353
Mycobacterium kumamotonense DSM 45093
Mycobacterium longobardum DSM 45394
Mycobacterium malmesburyense WCM 7299
Mycobacterium celeriflavum DSM 46765
Mycobacterium hassiacum DSM 44199
Mycobacterium thermoresistibile ATCC 19527
Mycobacterium moriokaense CIP 105393
Mycobacterium smegmatis NCTC 8159
Mycobacterium phlei CCUG 21000
Mycobacterium confluentis DSM 44017
Tsukamurella pseudospumae JCM 13375
Tsukamurella sunchonensis JCM 15929
Tsukamurella pulmonis DSM 44142
Tsukamurella spongiae DSM 44990
Tsukamurella tyrosinosolvens DSM 44234
Tsukamurella carboxydivorans JCM 15482
Tsukamurella paurometabola DSM 20162
Tomitella biformata AHU 1821
Rhodococcus equi NBRC 101255
Rhodococcus hoagii DSM 20295
Rhodococcus imtechensis RKJ300
Rhodococcus wratislaviensis NBRC 100605
Rhodococcus opacus DSM 43205
Rhodococcus marinonascens NBRC 14363
Rhodococcus koreensis DSM 44498
Rhodococcus yunnanensis NBRC 103083
Rhodococcus fascians LMG 3623
Rhodococcus erythropolis NBRC 15567
Rhodococcus qingshengii JCM 15477
Rhodococcus globerulus NBRC 14531
Rhodococcus jostii DSM 44719
Rhodococcus tukisamuensis JCM 11308
Rhodococcus maanshanensis DSM 44675
Rhodococcus defluvii Ca11
Nocardia asiatica NBRC 100129
Nocardia arthritidis NBRC 100137
Nocardia araoensis NBRC 100135
Nocardia amamiensis NBRC 102102
Nocardia pneumoniae NBRC 100136
Nocardia beijingensis NBRC 16342
Nocardia amikacinitolerans DSM 45539
Nocardia niwae DSM 45340
Nocardia brasiliensis NBRC 14402
Nocardia vulneris NBRC 108936
Nocardia transvalensis NBRC 15921
Nocardia takedensis NBRC 100417
Nocardia xishanensis NBRC 101358
Nocardia exalbida NBRC 100660
Nocardia cyriacigeorgica NBRC 100375
Nocardia vermiculata NBRC 100427
Nocardia vaccinii NBRC 15922
Nocardia africana NBRC 100379
Nocardia veterana NBRC 100344
Nocardia kruczakiae NBRC 101016
Nocardia cerradoensis NBRC 101014
Nocardia aobensis NBRC 100429
Nocardia vinacea NBRC 16497
Nocardia pseudovaccinii NBRC 100343
Nocardia anaemiae NBRC 100462
Nocardia otitidiscaviarum NBRC 14405
Nocardia niigatensis NBRC 100131
Nocardia inohanensis NBRC 100128
Nocardia yamanashiensis NBRC 100130
Nocardia fluminea DSM 44489
Nocardia soli NBRC 100376
Nocardia cummidelens NBRC 100378
Nocardia salmonicida NBRC 13393
Nocardia jejuensis NBRC 103114
Nocardia concava NBRC 100430
Nocardia crassostreae NBRC 100342
Nocardia uniformis NBRC 13702
Nocardia jiangxiensis NBRC 101359
Nocardia nova NBRC 15556
Nocardia paucivorans NBRC 100373
Nocardia brevicatena NBRC 12119
Nocardia carnea NBRC 14403
Nocardia rhamnosiphila NRRL B-24637
Nocardia sienata NBRC 100364
Nocardia testacea NBRC 100365
Nocardia asteroides NBRC 15531
Nocardia asteroides DSM 43373
Nocardia thailandica NBRC 100428
Nocardia abscessus NBRC 100374
Nocardia shimofusensis NBRC 100134
Nocardia higoensis NBRC 100133
Nocardia farcinica NBRC 15532

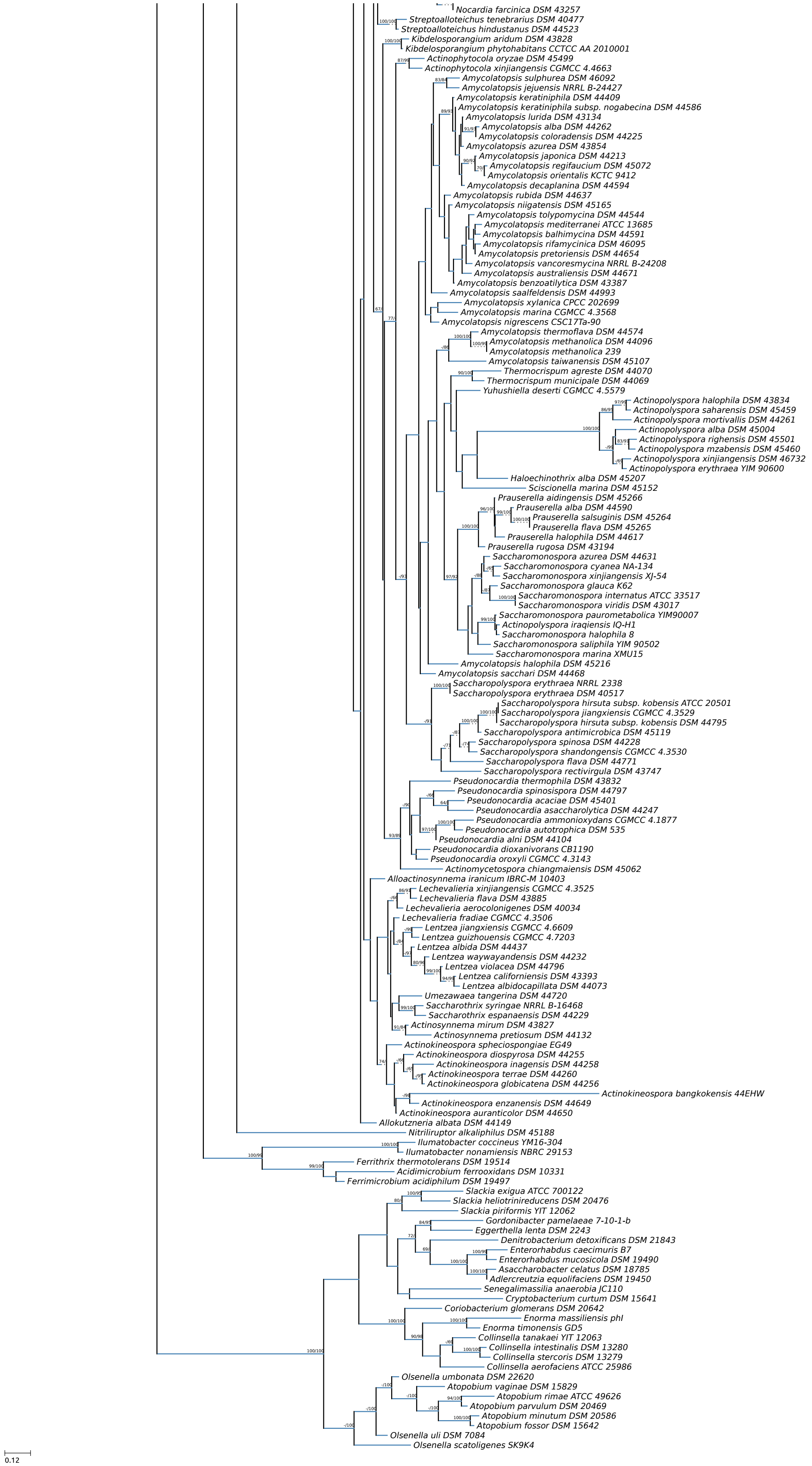
Nocardia farcinica DSM 43257
Streptoalloteichus tenebrarius DSM 40477
Streptoalloteichus hindustanus DSM 44523
Kibdelosporangium aridum DSM 43828
Kibdelosporangium phytohabitans CCTCC AA 2010001
Actinophytocola oryzae DSM 45499
Actinophytocola xinjiangensis CGMCC 4.4663
Amycolatopsis sulphurea DSM 46092
Amycolatopsis jejuensis NRRL B-24427
Amycolatopsis keratiniphila DSM 44409
Amycolatopsis keratiniphila subsp. nogabecina DSM 44586
Amycolatopsis lurida DSM 43134
Amycolatopsis alba DSM 44262
Amycolatopsis coloradensis DSM 44225
Amycolatopsis azurea DSM 43854
Amycolatopsis japonica DSM 44213
Amycolatopsis regifaucium DSM 45072
Amycolatopsis orientalis KCTC 9412
Amycolatopsis decaplanina DSM 44594
Amycolatopsis rubida DSM 44637
Amycolatopsis niigatensis DSM 45165
Amycolatopsis tolypomycina DSM 44544
Amycolatopsis mediterranei ATCC 13685
Amycolatopsis balhimycina DSM 44591
Amycolatopsis rifamycinica DSM 46095
Amycolatopsis pretoriensis DSM 44654
Amycolatopsis vancoresmycina NRRL B-24208
Amycolatopsis australiensis DSM 44671
Amycolatopsis benzoatilytica DSM 43387
Amycolatopsis saalfeldensis DSM 44993
Amycolatopsis xylanica CPCC 202699
Amycolatopsis marina CGMCC 4.3568
Amycolatopsis nigrescens CSC17Ta-90
Amycolatopsis thermoflava DSM 44574
Amycolatopsis methanolica DSM 44096
Amycolatopsis methanolica 239
Amycolatopsis taiwanensis DSM 45107
Thermocrispum agreste DSM 44070
Thermocrispum municipale DSM 44069
Yuhushiella deserti CGMCC 4.5579
Actinopolyspora halophila DSM 43834
Actinopolyspora saharensis DSM 45459
Actinopolyspora mortivallis DSM 44261
Actinopolyspora alba DSM 45004
Actinopolyspora righensis DSM 45501
Actinopolyspora mzabensis DSM 45460
Actinopolyspora xinjiangensis DSM 46732
Actinopolyspora erythraea YIM 90600
Haloechinothrix alba DSM 45207
Sciscionella marina DSM 45152
Prauserella aidingensis DSM 45266
Prauserella alba DSM 44590
Prauserella salsuginis DSM 45264
Prauserella flava DSM 45265
Prauserella halophila DSM 44617
Prauserella rugosa DSM 43194
Saccharomonospora azurea DSM 44631
Saccharomonospora cyanea NA-134
Saccharomonospora xinjiangensis XJ-54
Saccharomonospora glauca K62
Saccharomonospora internatus ATCC 33517
Saccharomonospora viridis DSM 43017
Saccharomonospora paurometabolica YIM90007
Actinopolyspora iraqiensis IQ-H1
Saccharomonospora halophila 8
Saccharomonospora saliphila YIM 90502
Saccharomonospora marina XMU15
Amycolatopsis halophila DSM 45216
Amycolatopsis sacchari DSM 44468
Saccharopolyspora erythraea NRRL 2338
Saccharopolyspora erythraea DSM 40517
Saccharopolyspora hirsuta subsp. kobensis ATCC 20501
Saccharopolyspora jiangxiensis CGMCC 4.3529
Saccharopolyspora hirsuta subsp. kobensis DSM 44795
Saccharopolyspora antimicrobica DSM 45119
Saccharopolyspora spinosa DSM 44228
Saccharopolyspora shandongensis CGMCC 4.3530
Saccharopolyspora flava DSM 44771
Saccharopolyspora rectivirgula DSM 43747
Pseudonocardia thermophila DSM 43832
Pseudonocardia spinosispora DSM 44797
Pseudonocardia acaciae DSM 45401
Pseudonocardia asaccharolytica DSM 44247
Pseudonocardia ammonioxydans CGMCC 4.1877
Pseudonocardia autotrophica DSM 535
Pseudonocardia alni DSM 44104
Pseudonocardia dioxanivorans CB1190
Pseudonocardia oroxyli CGMCC 4.3143
Actinomycetospora chiangmaiensis DSM 45062
Alloactinosynnema iranicum IBRC-M 10403
Lechevalieria xinjiangensis CGMCC 4.3525
Lechevalieria flava DSM 43885
Lechevalieria aerocolonigenes DSM 40034
Lechevalieria fradiae CGMCC 4.3506
Lentzea jiangxiensis CGMCC 4.6609
Lentzea guizhouensis CGMCC 4.7203
Lentzea albida DSM 44437
Lentzea waywayandensis DSM 44232
Lentzea violacea DSM 44796
Lentzea californiensis DSM 43393
Lentzea albidocapillata DSM 44073
Umezawaea tangerina DSM 44720
Saccharothrix syringae NRRL B-16468
Saccharothrix espanaensis DSM 44229
Actinosynnema mirum DSM 43827
Actinosynnema pretiosum DSM 44132
Actinokineospora spheciospongiae EG49
Actinokineospora diospyrosa DSM 44255
Actinokineospora inagensis DSM 44258
Actinokineospora terrae DSM 44260
Actinokineospora globicatena DSM 44256
Actinokineospora bangkokensis 44EHW
Actinokineospora enzanensis DSM 44649
Actinokineospora auranticolor DSM 44650
Allokutzneria albata DSM 44149
Nitriliruptor alkaliphilus DSM 45188
Ilumatobacter coccineus YM16-304
Ilumatobacter nonamiensis NBRC 29153
Ferrithrix thermotolerans DSM 19514
Acidimicrobium ferrooxidans DSM 10331
Ferrimicrobium acidiphilum DSM 19497
Slackia exigua ATCC 700122
Slackia heliotrinireducens DSM 20476
Slackia piriformis YIT 12062
Gordonibacter pamelaeae 7-10-1-b
Eggerthella lenta DSM 2243
Denitrobacterium detoxificans DSM 21843
Enterorhabdus caecimuris B7
Enterorhabdus mucosicola DSM 19490
Asaccharobacter celatus DSM 18785
Adlercreutzia equolifaciens DSM 19450
Senegalimassilia anaerobia JC110
Cryptobacterium curtum DSM 15641
Coriobacterium glomerans DSM 20642
Enorma massiliensis phI
Enorma timonensis GD5
Collinsella tanakaei YIT 12063
Collinsella intestinalis DSM 13280
Collinsella stercoris DSM 13279
Collinsella aerofaciens ATCC 25986
Olsenella umbonata DSM 22620
Atopobium vaginae DSM 15829
Atopobium rimae ATCC 49626
Atopobium parvulum DSM 20469
Atopobium minutum DSM 20586
Atopobium fossor DSM 15642
Olsenella uli DSM 7084
Olsenella scatoligenes SK9K4
0.12

Figure 5: 16S rRNA gene ML and MP tree using the sampling from the GBDP tree. Dotted parts of branches are filled in to allow proper placement of bootstrap values and are not part of the actual branch length.

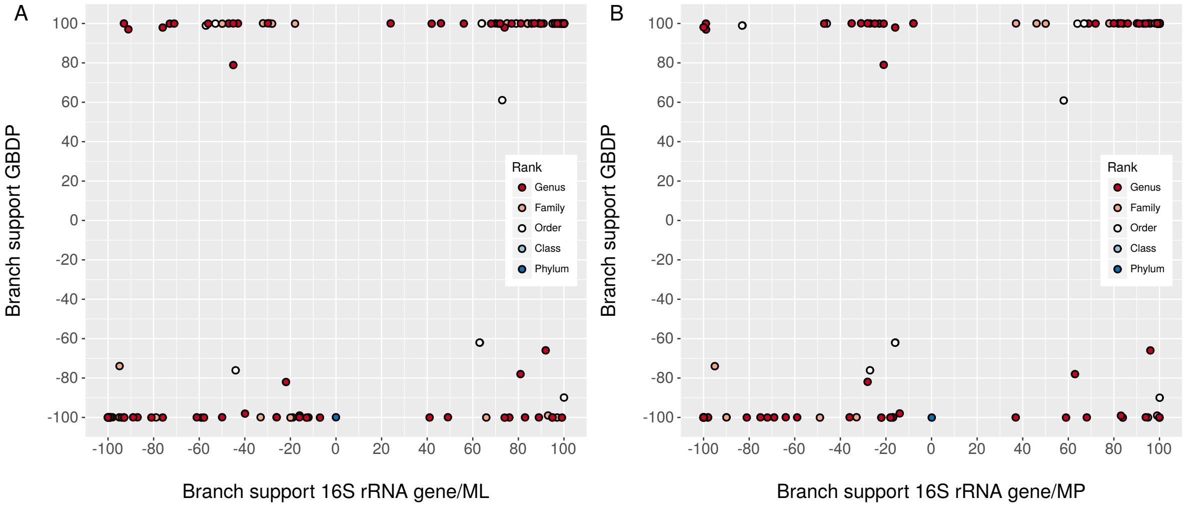

Figure 6: Comparison of branch support. Shown are the negative or positive branch support values for each taxon inferred from whole genomes with GBDP in comparison to those inferred from 16S rRNA gene sequences with ML (A) and MP (B). The colors indicate the taxonomic rank. The upper right corner contains the taxa significantly supported by all methods, the lower left corner those significantly opposed by all methods. The other two corners, which would indicate a significant conflict between the methods, are empty. Jitter was used to avoid overplotting.

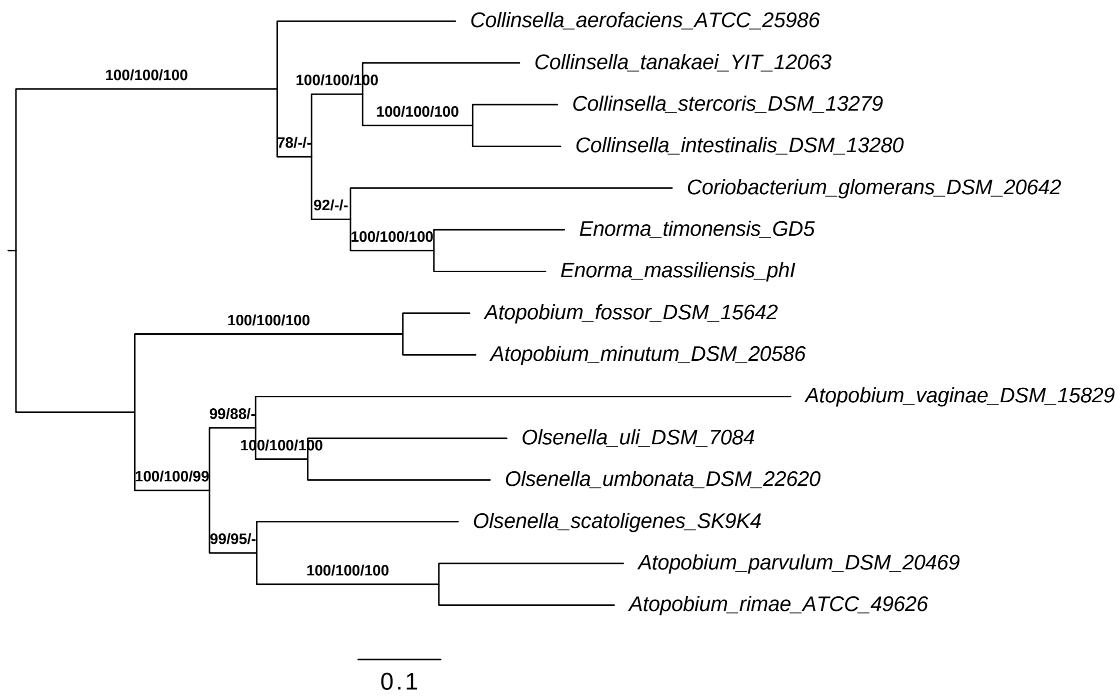

Figure 7: Phylogenetic tree inferred with RAxML from the *Atopobium* supermatrix including single-copy core genes. Branches are scaled in terms of expected number of changes per site. Numbers above branches are partition bootstrap support values from (i) RAxML analysis of the single-copy core genes; (ii) TNT analysis of the single-copy core genes; (iii) TNT analysis of the supermatrix that included single-copy genes that occurred in at least four of the genomes.

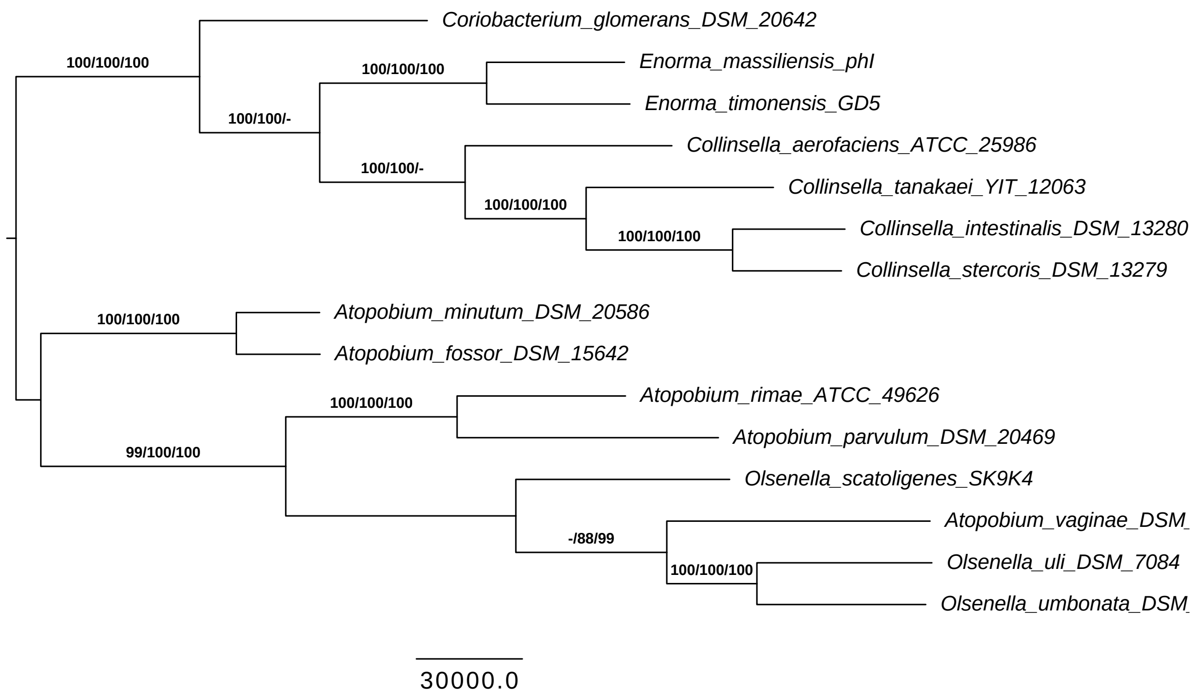

Figure 8: Phylogenetic tree inferred with TNT from the *Atopobium* supermatrix including single-copy genes that occurred in at least four of the genomes. Branches are scaled in terms of the minimum number of changes (ACCTRAN optimization). Numbers above branches are partition bootstrap support values from (i) TNT analysis of the supermatrix that included single-copy genes that occurred in at least four of the genomes; (ii) TNT analysis of the single-copy core genes; (iii) RAxML analysis of the single-copy core genes.

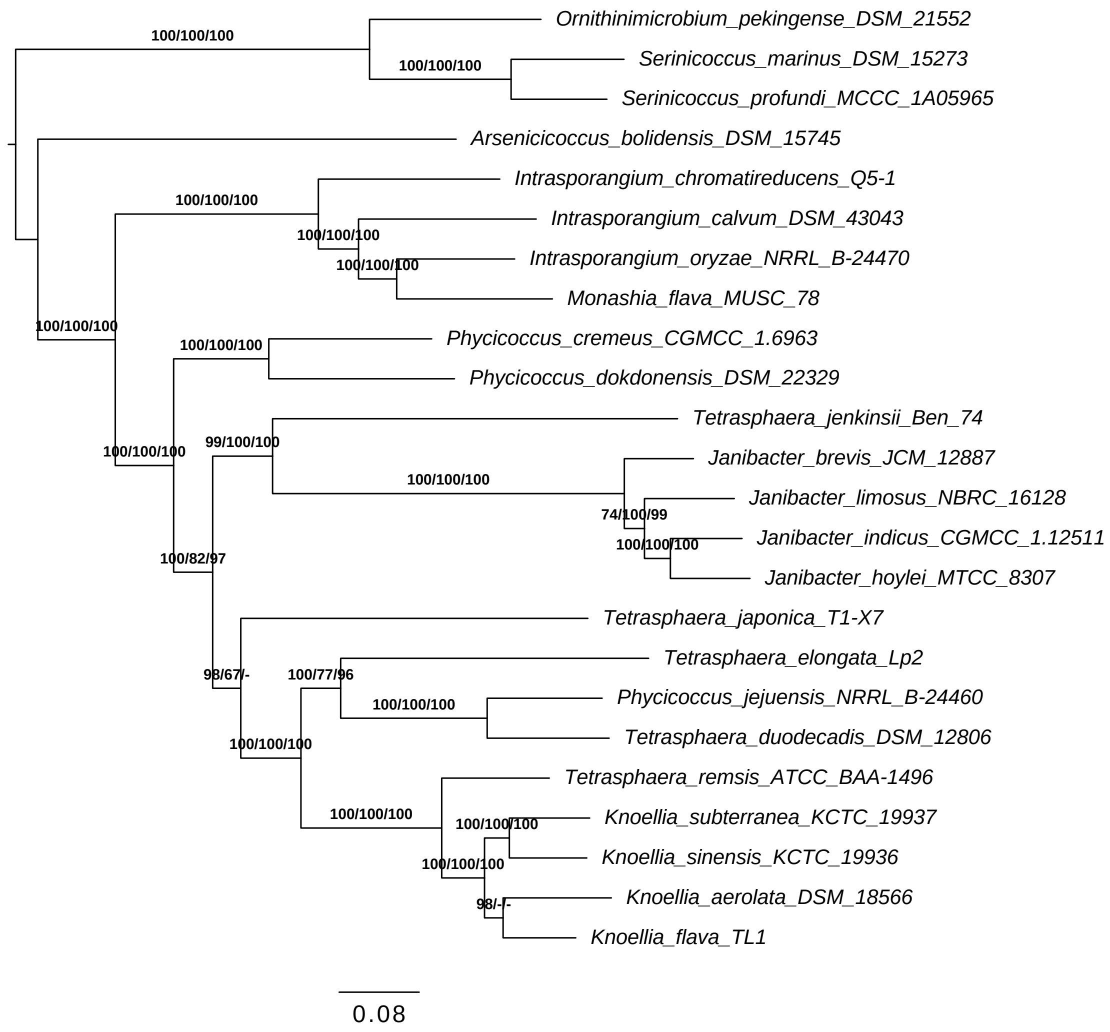

Figure 9: Phylogenetic tree inferred with RAxML from the *Intrasporangium* supermatrix including single-copy core genes. Branches are scaled in terms of expected number of changes per site. Numbers above branches are partition bootstrap support values from (i) RAxML analysis of the single-copy core genes; (ii) TNT analysis of the single-copy core genes; (iii) TNT analysis of the supermatrix that included single-copy genes that occurred in at least four of the genomes.

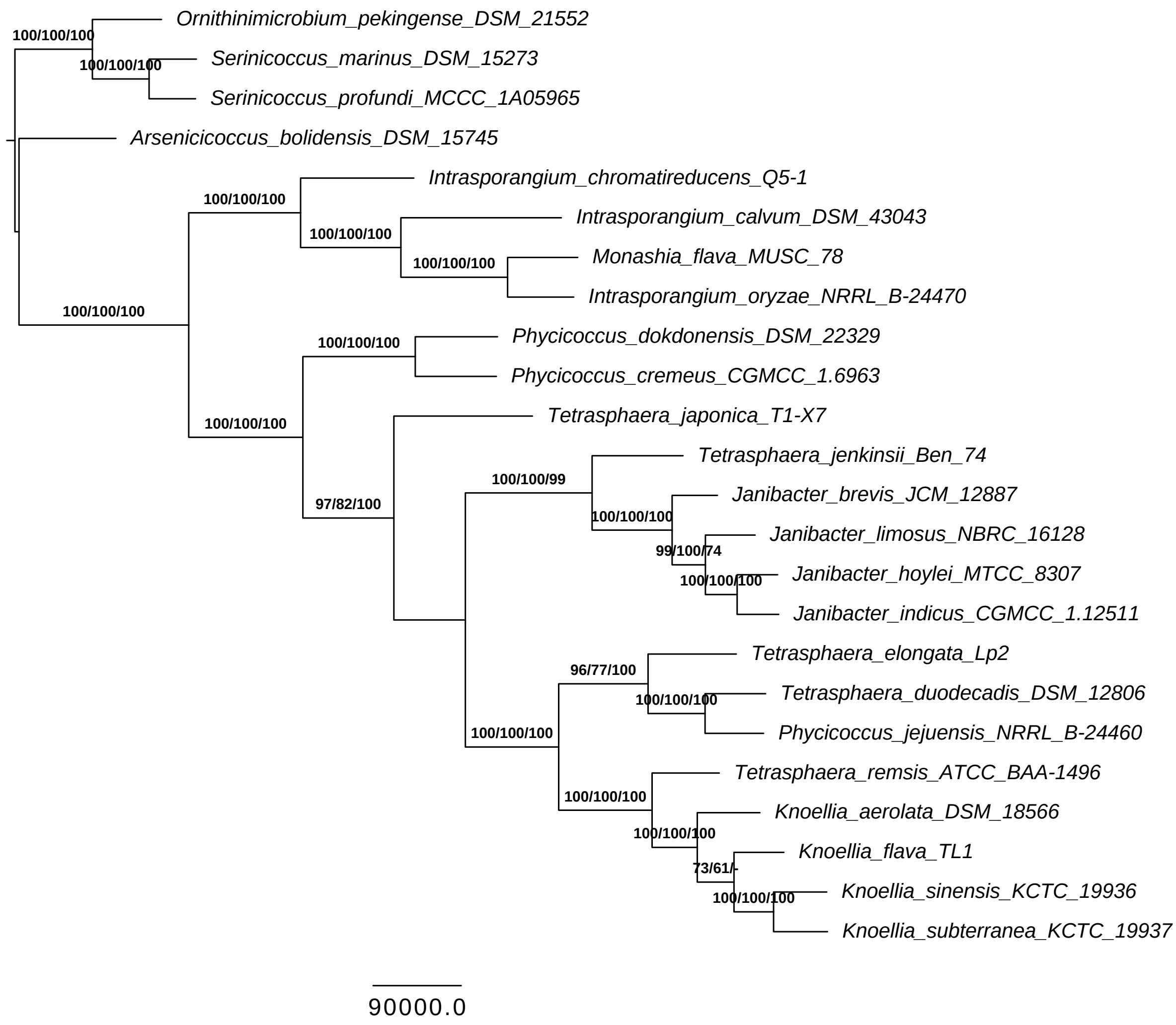

Figure 10: Phylogenetic tree inferred with TNT from the *Intrasporangium* supermatrix including single-copy genes that occurred in at least four of the genomes. Branches are scaled in terms of the minimum number of changes (ACCTRAN optimization). Numbers above branches are partition bootstrap support values from (i) TNT analysis of the supermatrix that included single-copy genes that occurred in at least four of the genomes; (ii) TNT analysis of the single-copy core genes; (iii) RAxML analysis of the single-copy core genes.

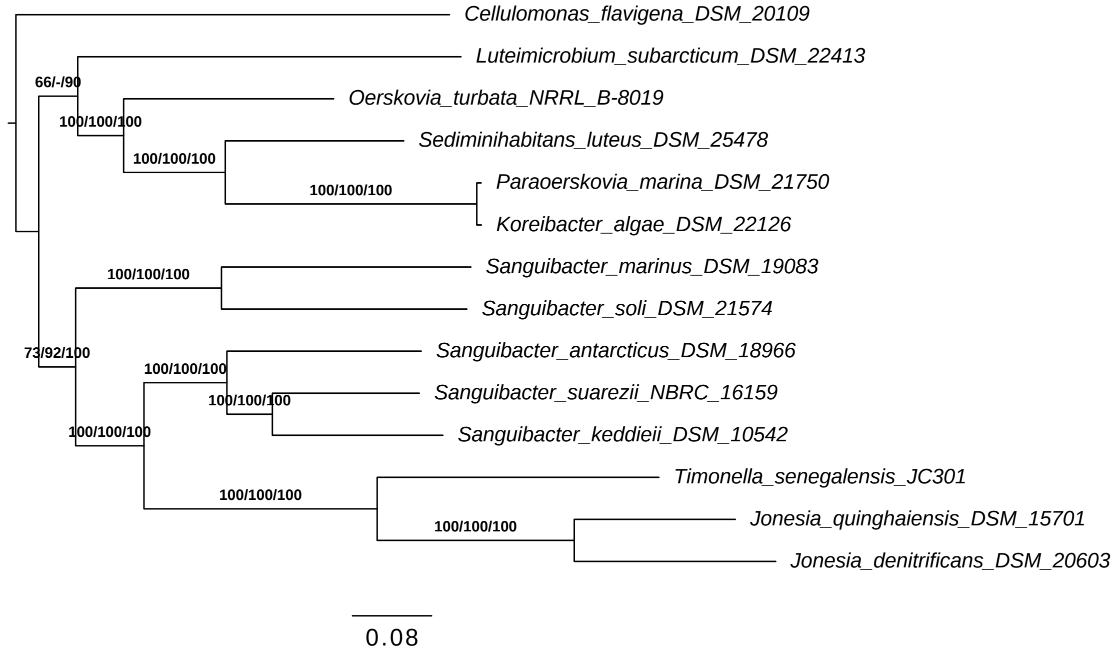

Figure 11: Phylogenetic tree inferred with RAxML from the *Sanguibacter* supermatrix including single-copy core genes. Branches are scaled in terms of expected number of changes per site. Numbers above branches are partition bootstrap support values from (i) RAxML analysis of the single-copy core genes; (ii) TNT analysis of the single-copy core genes; (iii) TNT analysis of the supermatrix that included single-copy genes that occurred in at least four of the genomes.

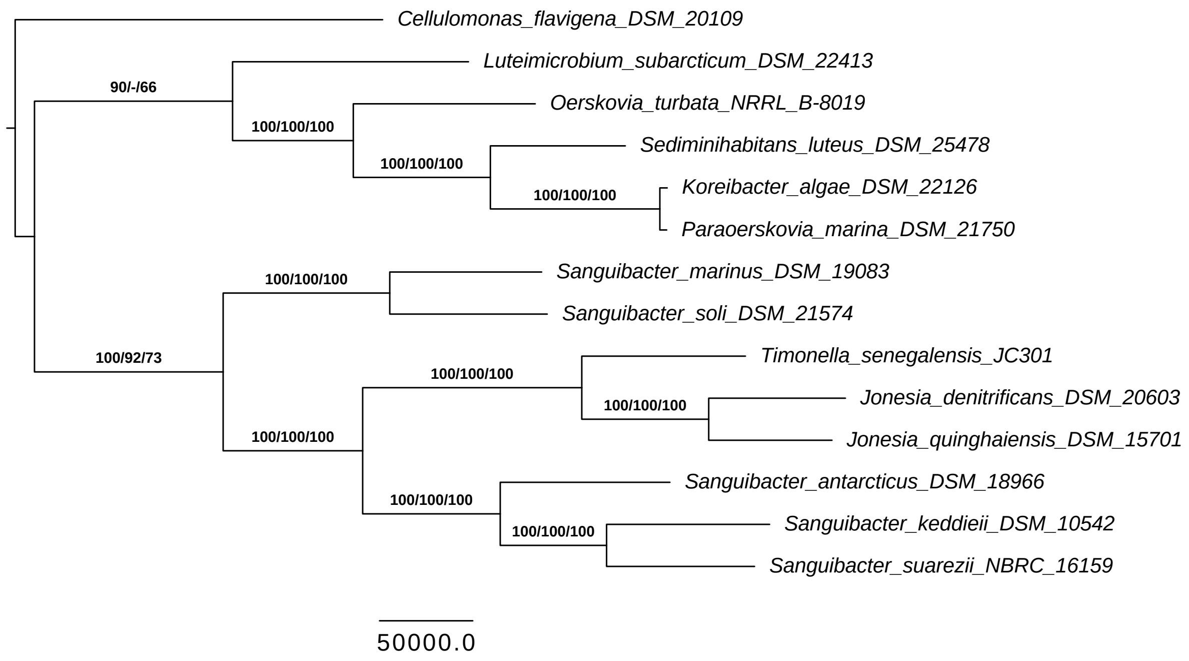

Figure 12: Phylogenetic tree inferred with TNT from the *Sanguibacter* supermatrix including single-copy genes that occurred in at least four of the genomes. Branches are scaled in terms of the minimum number of changes (ACCTRAN optimization). Numbers above branches are partition bootstrap support values from (i) TNT analysis of the supermatrix that included single-copy genes that occurred in at least four of the genomes; (ii) TNT analysis of the single-copy core genes; (iii) RAxML analysis of the single-copy core genes.

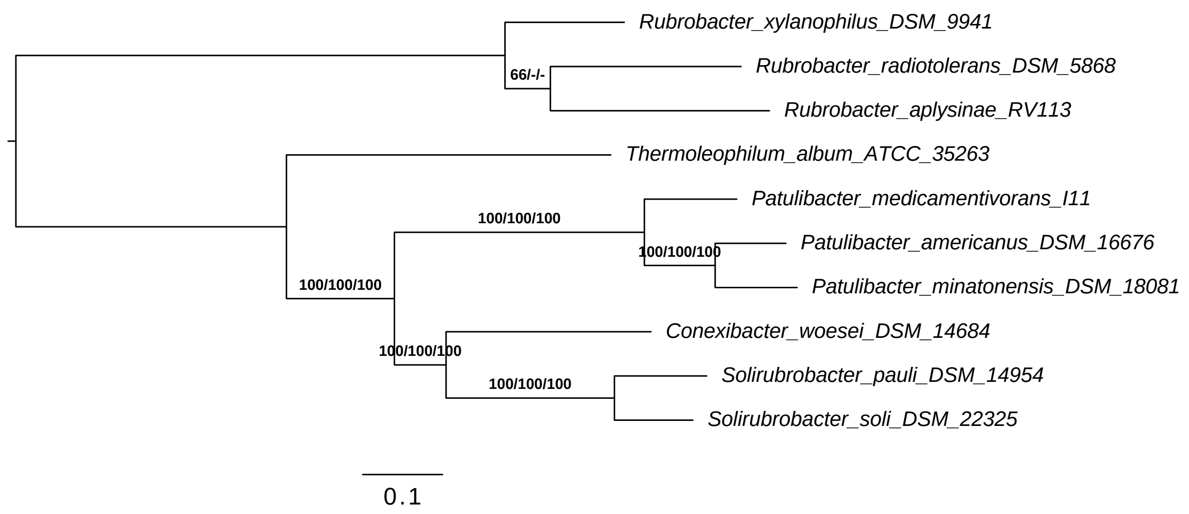

Figure 13: Phylogenetic tree inferred with RAxML from the *Solirubrobacter* supermatrix including single-copy core genes. Branches are scaled in terms of expected number of changes per site. Numbers above branches are partition bootstrap support values from (i) RAxML analysis of the single-copy core genes; (ii) TNT analysis of the single-copy core genes; (iii) TNT analysis of the supermatrix that included single-copy genes that occurred in at least four of the genomes.

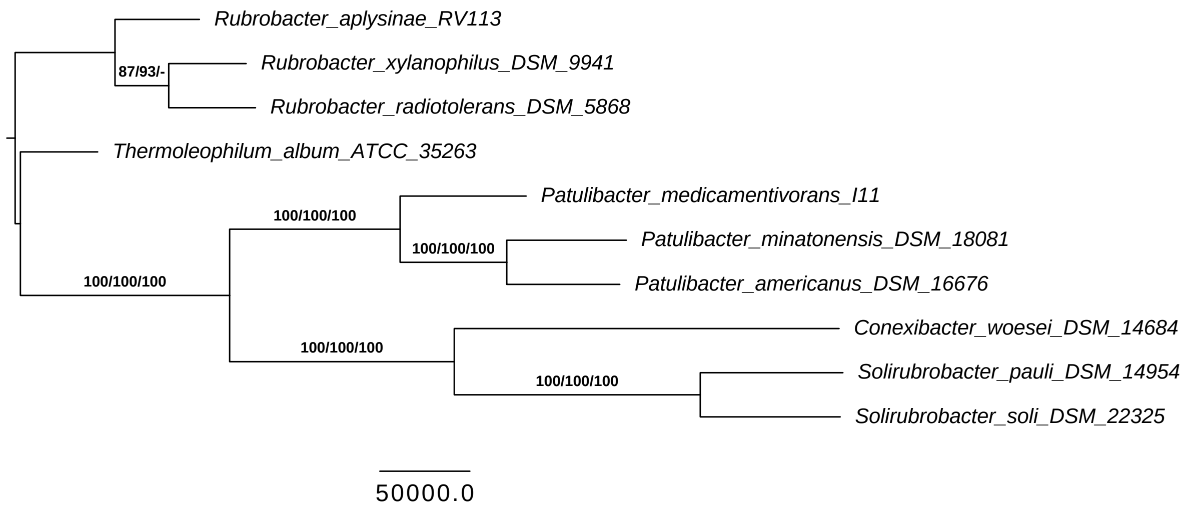

Figure 14: Phylogenetic tree inferred with TNT from the *Solirubrobacter* supermatrix including single-copy genes that occurred in at least four of the genomes. Branches are scaled in terms of the minimum number of changes (ACCTRAN optimization). Numbers above branches are partition bootstrap support values from (i) TNT analysis of the supermatrix that included single-copy genes that occurred in at least four of the genomes; (ii) TNT analysis of the single-copy core genes; (iii) RAxML analysis of the single-copy core genes.

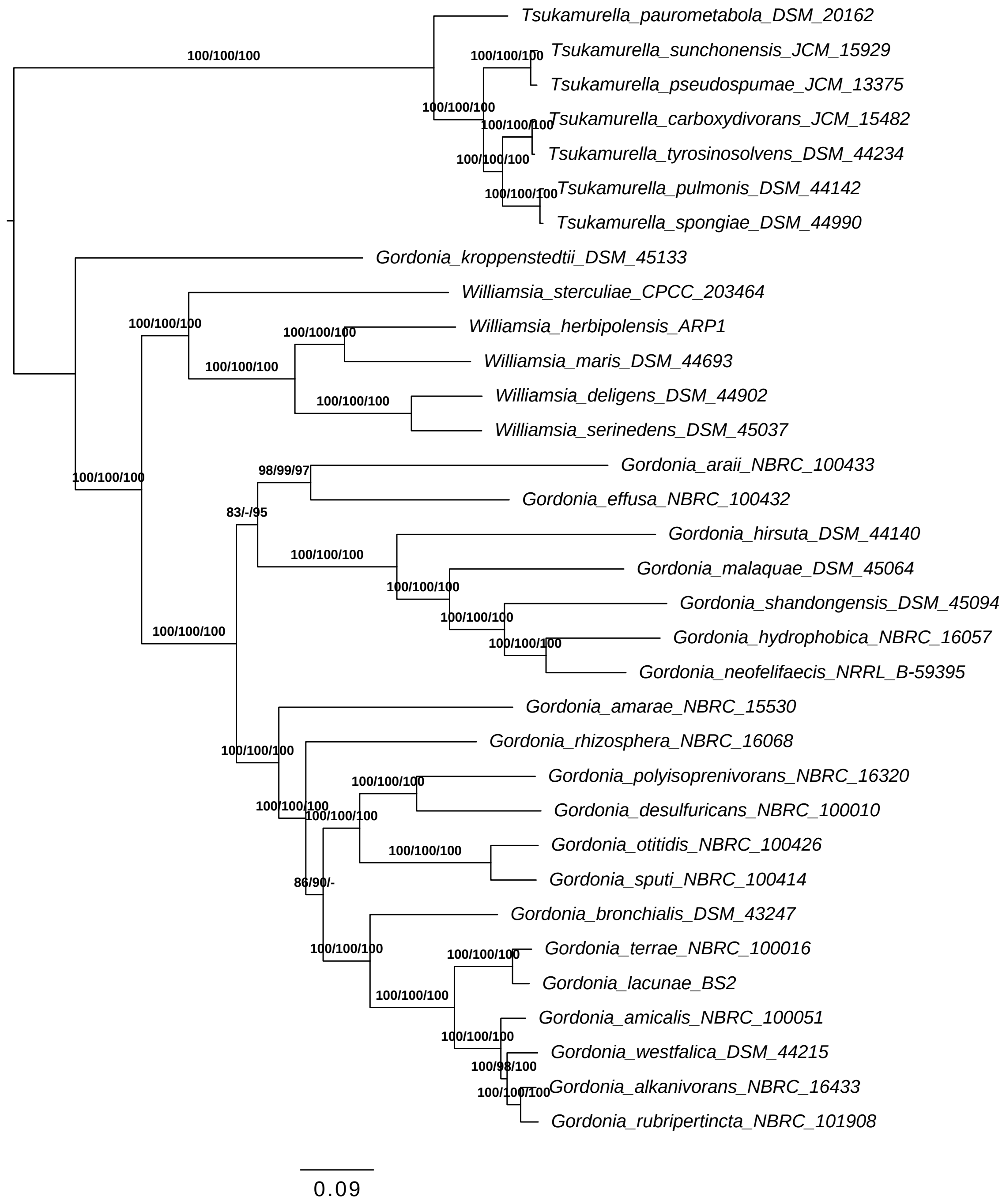

Figure 15: Phylogenetic tree inferred with RAxML from the *Gordonia* supermatrix including single-copy core genes. Branches are scaled in terms of expected number of changes per site. Numbers above branches are partition bootstrap support values from (i) RAxML analysis of the single-copy core genes; (ii) TNT analysis of the single-copy core genes; (iii) TNT analysis of the supermatrix that included single-copy genes that occurred in at least four of the genomes.

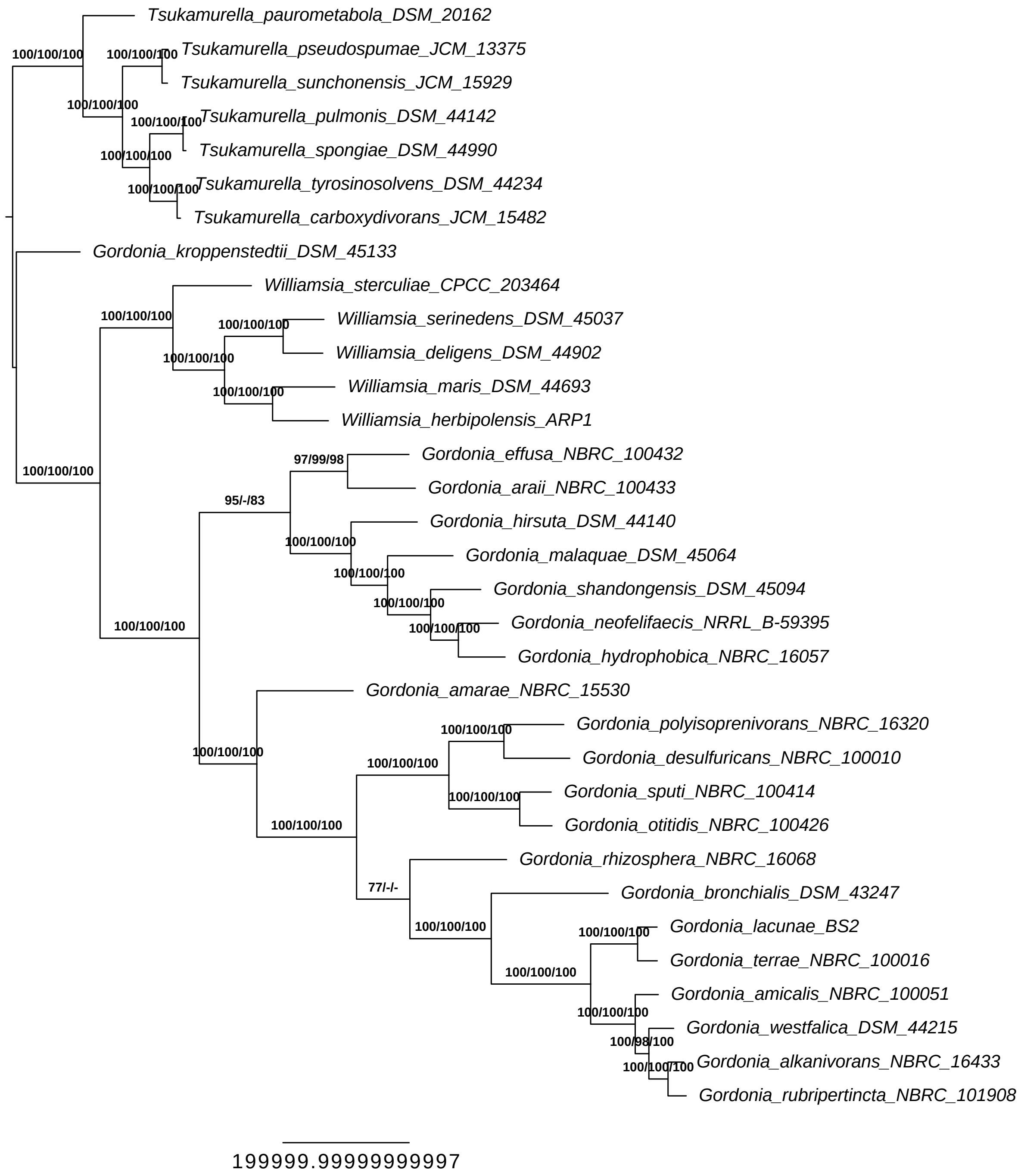

Figure 16: Phylogenetic tree inferred with TNT from the *Gordonia* supermatrix including single-copy genes that occurred in at least four of the genomes. Branches are scaled in terms of the minimum number of changes (ACCTRAN optimization). Numbers above branches are partition bootstrap support values from (i) TNT analysis of the supermatrix that included single-copy genes that occurred in at least four of the genomes; (ii) TNT analysis of the single-copy core genes; (iii) RAxML analysis of the single-copy core genes.

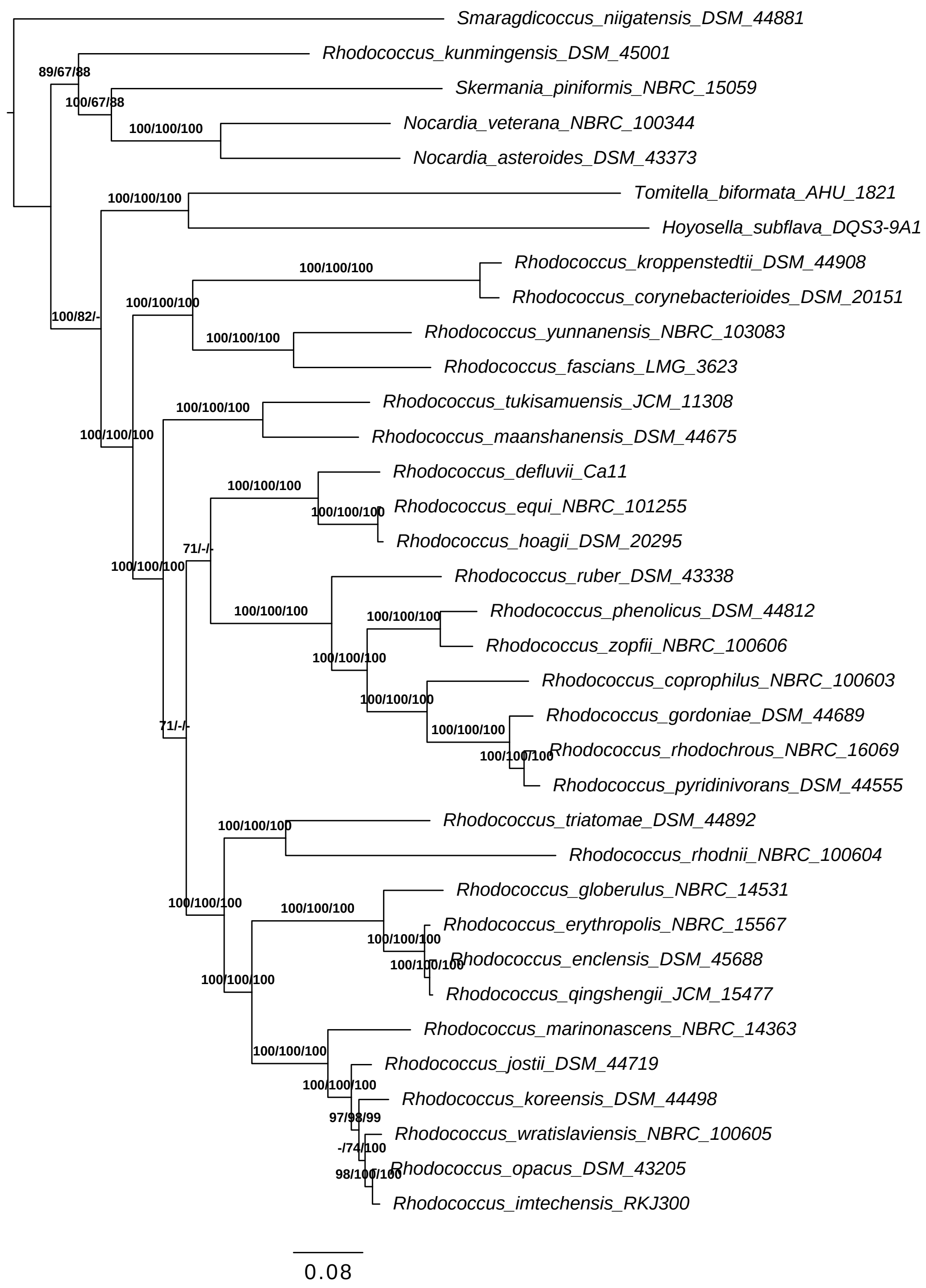

Figure 17: Phylogenetic tree inferred with RAxML from the *Rhodococcus* supermatrix including single-copy core genes. Branches are scaled in terms of expected number of changes per site. Numbers above branches are partition bootstrap support values from (i) RAxML analysis of the single-copy core genes; (ii) TNT analysis of the single-copy core genes; (iii) TNT analysis of the supermatrix that included single-copy genes that occurred in at least four of the genomes.

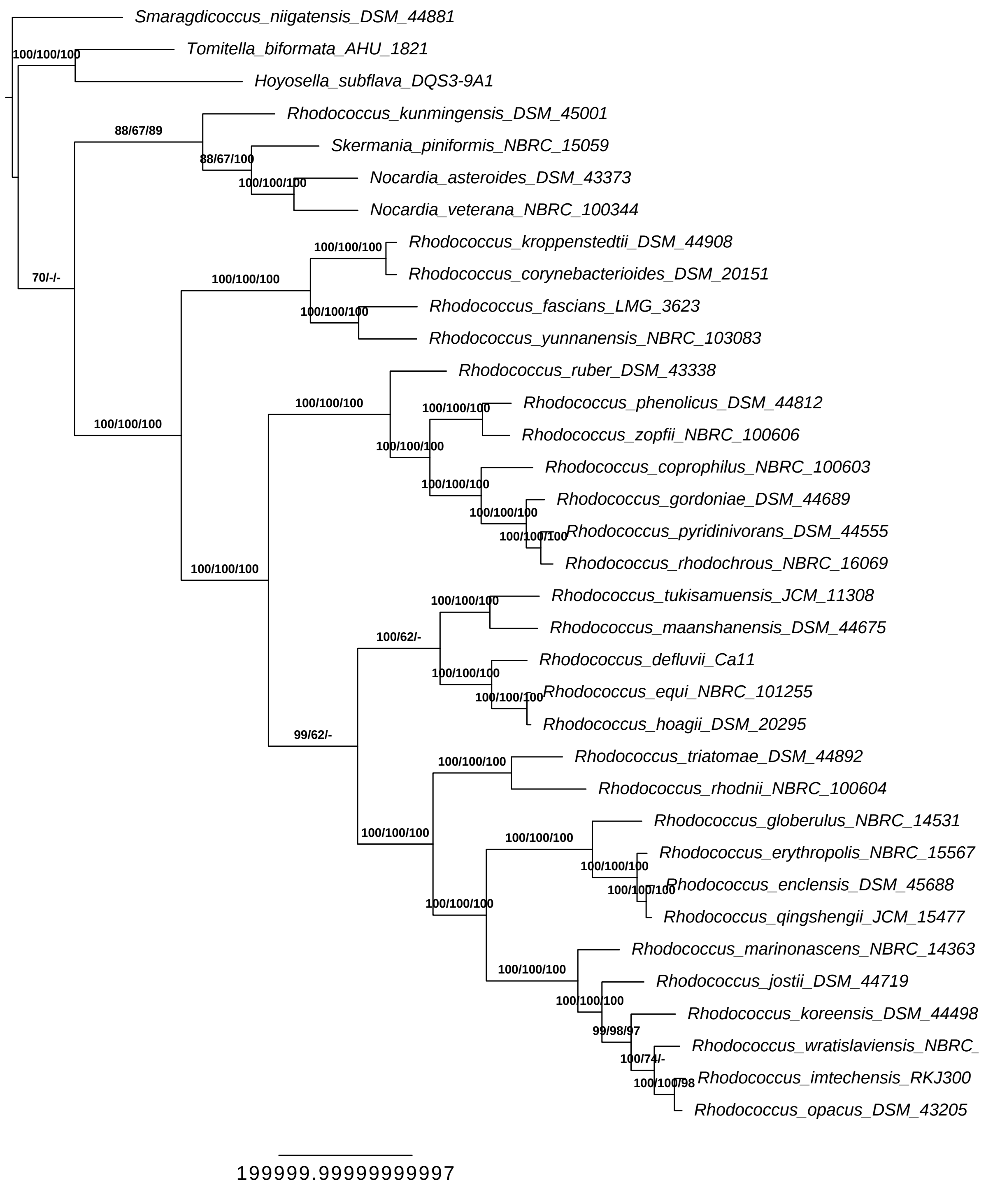

Figure 18: Phylogenetic tree inferred with TNT from the *Rhodococcus* supermatrix including single-copy genes that occurred in at least four of the genomes. Branches are scaled in terms of the minimum number of changes (ACCTRAN optimization). Numbers above branches are partition bootstrap support values from (i) TNT analysis of the supermatrix that included single-copy genes that occurred in at least four of the genomes; (ii) TNT analysis of the single-copy core genes; (iii) RAxML analysis of the single-copy core genes.

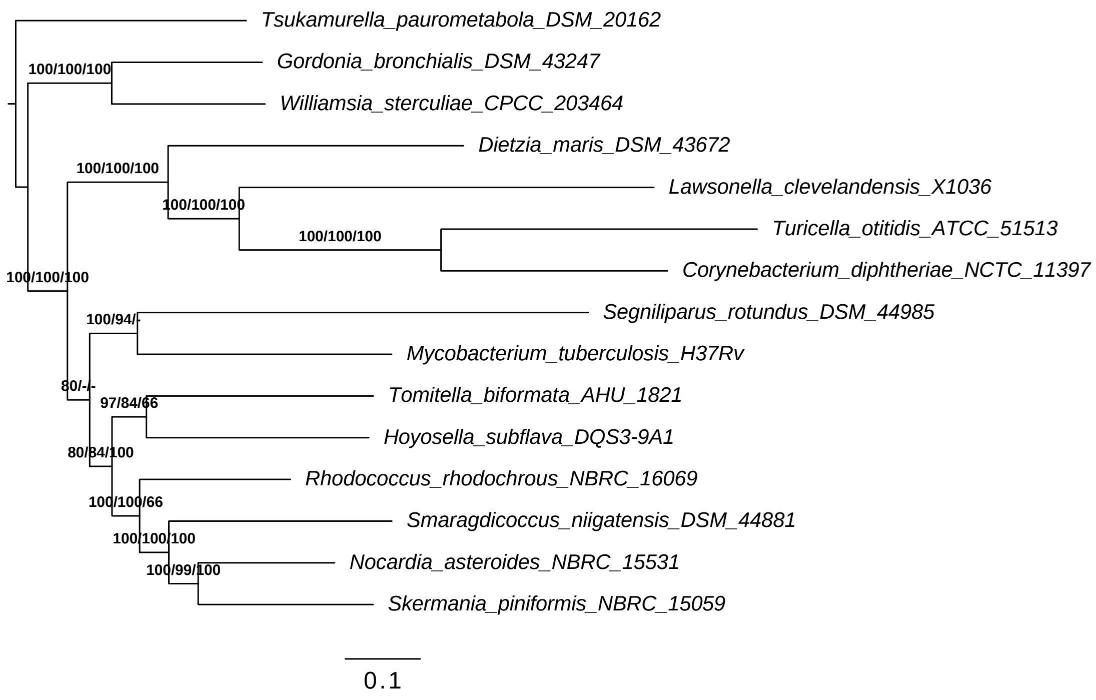

Figure 19: Phylogenetic tree inferred with RAxML from the *Corynebacterium* supermatrix including single-copy core genes. Branches are scaled in terms of expected number of changes per site. Numbers above branches are partition bootstrap support values from (i) RAxML analysis of the single-copy core genes; (ii) TNT analysis of the single-copy core genes; (iii) TNT analysis of the supermatrix that included single-copy genes that occurred in at least four of the genomes.

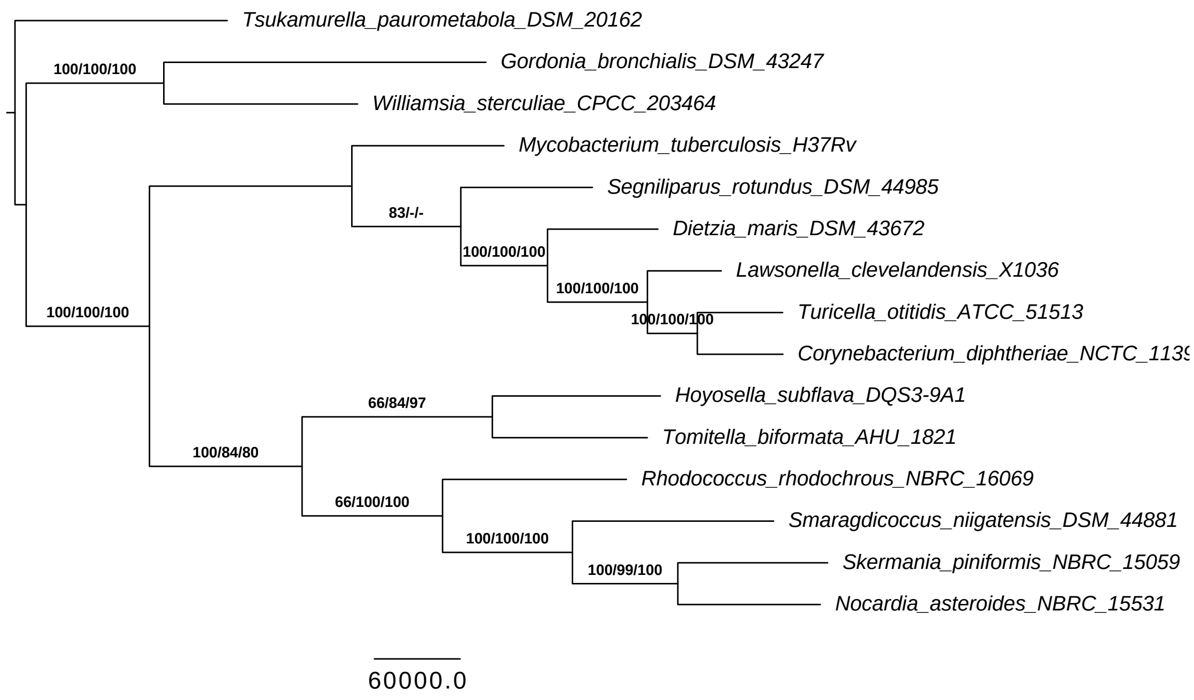

Figure 20: Phylogenetic tree inferred with TNT from the *Corynebacterium* supermatrix including single-copy genes that occurred in at least four of the genomes. Branches are scaled in terms of the minimum number of changes (ACCTRAN optimization). Numbers above branches are partition bootstrap support values from (i) TNT analysis of the supermatrix that included single-copy genes that occurred in at least four of the genomes; (ii) TNT analysis of the single-copy core genes; (iii) RAxML analysis of the single-copy core genes.
